# KRAS mutations are negatively correlated with immunity in colon cancer

**DOI:** 10.18632/aging.202182

**Published:** 2020-11-26

**Authors:** Xiaorui Fu, Xinyi Wang, Jinzhong Duanmu, Taiyuan Li, Qunguang Jiang

**Affiliations:** 1 Department of Gastrointestinal Surgery, The First Affiliated Hospital of Nanchang University, Nanchang, Jiangxi, People's Republic of China; 2 Queen Mary College, Medical Department, Nanchang University, Nanchang, Jiangxi, People's Republic of China

**Keywords:** KRAS mutations, immunity, colon cancer, tumor-infiltrating immune cells, inflammation

## Abstract

The heterogeneity of colon cancer tumors suggests that therapeutics targeting specific molecules may be effective in only a few patients. It is therefore necessary to explore gene mutations in colon cancer. In this study, we obtained colon cancer samples from The Cancer Genome Atlas, and the International Cancer Genome Consortium. We evaluated the landscape of somatic mutations in colon cancer and found that KRAS mutations, particularly rs121913529, were frequent and had prognostic value. Using ESTIMATE analysis, we observed that the KRAS-mutated group had higher tumor purity, lower immune score, and lower stromal score than the wild-type group. Through single-sample Gene Set Enrichment Analysis and Gene Set Enrichment Analysis, we found that KRAS mutations negatively correlated with enrichment levels of tumor infiltrating lymphocytes, inflammation, and cytolytic activities. HLA gene expression and checkpoint-related genes were also lower in the KRAS-mutated group. Finally, we found 24 immune-related genes that differed in expression between the KRAS-mutated and wild-type samples, which may provide clues to the mechanism of *KRAS*-related immune alteration. Our findings are indicative of the prognostic and predictive value of KRAS and illustrate the relationship between KRAS mutations and immune activity in colon cancer.

## INTRODUCTION

Colon cancer is the third leading cause of cancer deaths, with more than 1 million new cases diagnosed every year [1]. Heterogeneity is a characteristic of colon cancer whereby the pattern of mutations differ significantly among patients [2]. Mutations in essential genes can affect the proliferation, differentiation, apoptosis, survival capacity, and distant metastasis of tumor cells [3]. Thus, therapeutic methods that target specific biomolecules or genes are effective in a small fraction of patients. It is necessary to explore gene mutations and more potential therapeutic targets for colon cancer. *APC*, a tumor suppressor gene, is the most frequently mutated gene in patients with colon cancer and influences the Wnt/β-catenin pathway [4]. Mutated *APC* has been observed in early stage colon cancer and is correlated with clinical outcomes [5]. However, it was seldom detected in patients with late stage colon cancer and metastasis. In contrast, the *TP53* inactivating mutation is usually observed in more advanced tumors [6, 7]. Presently, RAS is the only predictive biomarker in the application of anti-EGFR agents to treat wild-type colon cancer [8, 9]. *KRAS* encodes a p21 protein, which couples with GTPase to transform GTP into GDP and regulates signaling pathways related to cellular growth and survival. When *KRAS* is mutated, the downstream signaling pathway (mitogen-activated protein kinase, MAPK) is activated, leading to cellular proliferation and tumor progression. In addition, *KRAS* mutations are predictive markers for breast, lung, ovarian, head/neck, and pancreatic cancers [4, 8, 9, 11]. For example, Jung et al. found that *KRAS* mutations were correlated with poor prognosis in patients with breast cancer, together with AKT signaling pathway activation, estrogen negative, and basal-like gene expression patterns. As BRAF is downstream of RAS in the MAPK/ERK signaling pathway, mutated BRAF is assumed to have the same resistance to the anti-EGFR agent as to the RAS-mutated colon tumor [10]. Furthermore, microsatellite instability (MSI) is another pathogenesis factor, and, if detected at an early stage, improves patient outcome [11, 12]. However, the mechanism of these gene mutations is unclear and personalized treatment requires further research on clinical biomarkers.

The minority of human colon cancers are genetically driven, including Lynch syndrome, familial adenomatous, and hamartomata’s polyposis [13]. The majority of colon cancer cases correlate with environmental and nonhereditary events, such as chronic inflammatory disease [14, 15]. Previous studies using mouse colon cancer models highlighted the importance of chronic inflammation in the development of colon cancer. In addition, these studies illustrated the mechanisms of inflammation-driven carcinogenesis in the intestine [16]. The infiltrating immune cells and their cytokines play a role in the inflammatory response. A higher neoantigen mutational load was positively correlated with T-lymphocyte infiltration and survival outcomes in patients with colon cancer [17]. Cytokines can be pro-inflammatory (IL-1, IL-6, IL-8, tumor-necrosis factor, transforming growth factor-β (TGF-β)) or anti-inflammatory (IL-1ra, IL-4, IL-10, IL-13) [18, 19]. Based on the successful utilization of immune checkpoint inhibitors, immunotherapy has gained grounds in clinical oncology practice in the last decade. Although patients with colon cancer have not benefited from immunotherapy, several studies have shown that colon tumors with high mutational burden may be potential targets of immune checkpoint inhibitors [20, 21]. Inhibition of MEK upregulates IFN-gamma-mediated human leukocyte antigen (HLA) and programmed death-1 receptor (PD-L1) expression in melanoma, colorectal, and breast cancers [22, 23]. The product of HLA genes—MHC protein—can also regulate the immune system [24]. There is an increasing role for PD-1 inhibition in MSI colon cancer, while the generalized activity of PD-1 inhibitors has not been seen in microsatellite stable (MSS) colon cancer [25, 26]. Thus, there is a need to study the relationship between specific genetic variants and immune events as well as alternative approaches to treat patients with different genetic characteristics.

In this study, we performed a comprehensive evaluation of somatic mutations in colon cancer. We found that *KRAS* mutations had a strong negative correlation with immunity and was of great prognostic value. We used single-sample Gene Set Enrichment Analysis (ssGSEA) and Gene Set Enrichment Analysis (GSEA) to identify the corresponding immune signatures of *KRAS* mutations and evaluated the relationship between *KRAS*-related pathways and immune cell infiltration. We compared the infiltration of immune cells, tumor mutational burden (TMB), HLA gene expression, and checkpoint-related genes between the *KRAS*-mutated and wild-type samples. Finally, in order to provide clues for the mechanism of *KRAS*-related immune alteration, we screened immune-related genes that differed in expression between the *KRAS*-mutated and wild-type samples.

## RESULTS

### The landscape of genetic mutations in colon cancer

**Figure 1:**
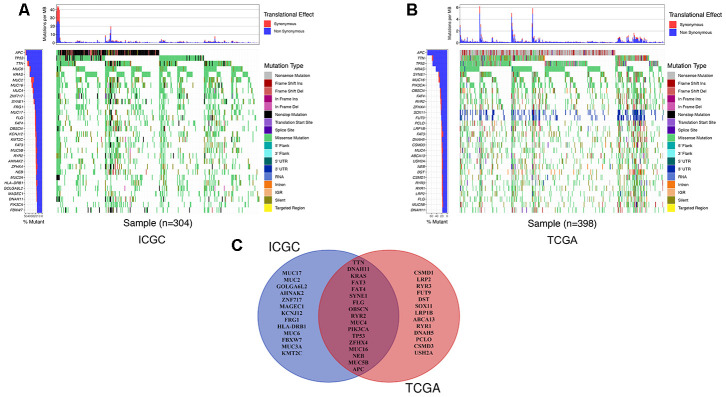
**The landscape of genetic mutations in colon cancer.** (**A**) The 30 most frequent mutations of samples in the ICGC database. The percentage of patients with mutations, translation effect (synonymous or non-synonymous), and mutation types were given. (**B**) The 30 most frequent mutations of samples in the TCGA database. (**C**) A Venn diagram of mutated genes. There were 17 genes that were members of the 30 most frequently mutated genes in the ICGC and TCGA databases.

**Figure 2:**
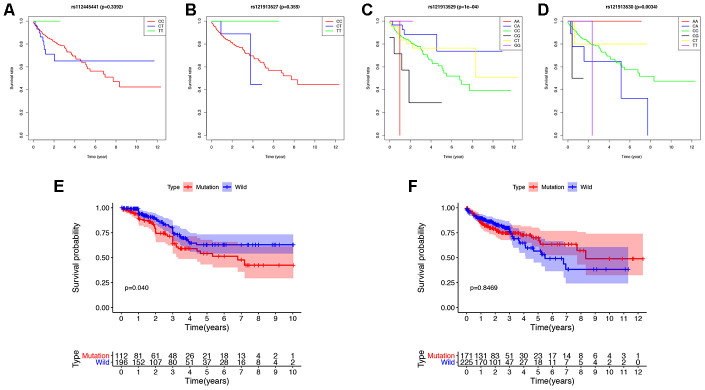
**Survival analysis of *KRAS* mutations in colon cancer.** (**A**–**D**) The survival rate of patients with different base-pairs in rs112445441, rs121913527, rs121913529, and rs121913530. (**E**, **F**) Comparison of the survival rates between *KRAS*-mutated and wild-type patients from the ICGC and TCGA databases. The difference in survival rate was statistically significant in ICGC (P=0.04), but not in TCGA (P=0.8469). In TCGA analysis, there were 171 and 225 patients with and without *KRAS* mutations, respectively. In ICGC analysis, there were 112 and 196 patients with and without *KRAS* mutations, respectively.

We detected the top thirty mutated genes in colon cancer samples from the ICGC database, and the top five of mutated genes were *APC*, *TP53*, *TTN*, *MUC6*, and *KRAS* (Figure 1A). We also detected the top thirty mutated genes in colon cancer samples from the The Cancer Genome Atlas (TCGA) database, and the top five mutated genes were *APC*, *TTN*, *TP53*, *KRAS*, and *SYNE1* (Figure 1B). Among the detected genes, 17 were members of the 30 most frequently mutated genes in the ICGC and TCGA databases (Figure 1C). The expression of some genes were significantly different in the mutated group than the wild-type group and included *APC* (p = 0.003), *DNAH11* (p = 0.021), *FAT3* (p = 0.031), *FAT4* (p = 0.002), *KRAS* (p = 0.039), *MUC5B* (p < 0.001), *PIK3CA* (p = 0.022), and *TP53* (p < 0.001) (Supplementary Figure 1). Next, we analyzed the mutational frequency of specific loci in the TCGA cohort. We found that rs121913529 in *KRAS* had the highest mutational frequency with 90 out of 399 patients having a mutation in this locus (Supplementary Table 2). Therefore, we predicted that *KRAS* mutations play an important role in colon cancer. We also performed survival analysis of four types of *KRAS* mutations whose mutation frequency were higher than 10/399: rs112445441 (p = 0.339), rs121913527 (p = 0.359), rs121913529 (p < 0.001), and rs121913530 (p = 0.003) (Figure 2A–2D). *KRAS*-mutated groups also showed worse survival outcomes compared to the wild-type groups in the ICGC cohort (p = 0.040) (Figure 2E–2F). These results indicate that *KRAS* mutations, particularly rs121913529, have prognostic value in colon cancer.

### KRAS mutations are negatively correlated with immune activities in colon cancer

**Figure 3:**
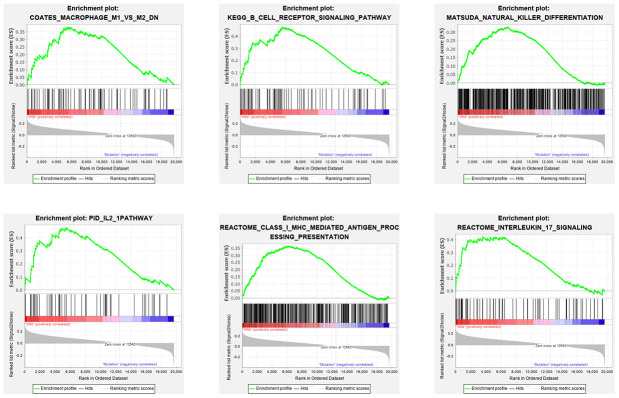
**Six immune pathways were enriched with *KRAS* mutations.** As shown in the enrichment plots, *KRAS* mutations were positively correlated with the immune pathways: macrophage M1 and M2, NK cell differentiation, class I MHC-mediated antigen processing, B cell receptor signaling, IL-2 and IL-17 pathways.

**Figure 4:**
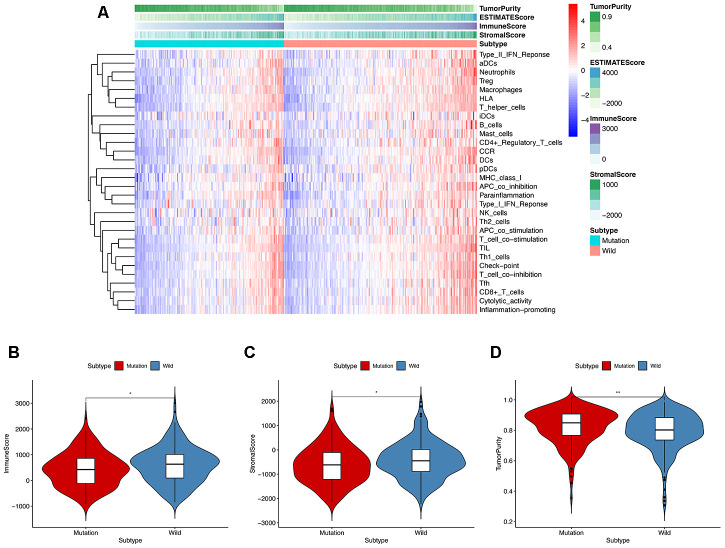
**ssGESA and ESTIMATE analysis of the relationship between *KRAS* mutations and immune activities.** (**A**) In ssGSEA, 30 immune-related pathways were incorporated to estimate the immune capacity of individual colon cancer samples. These gene sets were composed of immune cells and processes. The tumor purity, immune score, and stromal score are also shown in the heatmap. (**B**–**D**) Using the Mann-Whitney test, we found that the *KRAS*-mutated group was of lower immune and stromal score while its tumor purity was higher than the wild-type group.

**Figure 5:**
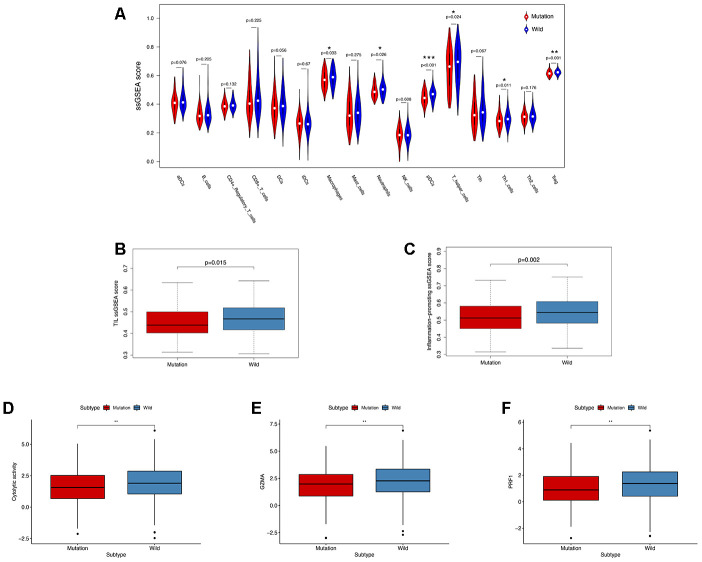
***KRAS*-mutated and wild-type groups differ in immune cell infiltration, inflammation, and cytolytic activities.** (**A**) Relative infiltration levels of 17 immune cells in the *KRAS*-mutated and wild-type groups. P values were calculated using a Mann-Whitney test. (**B**, **C**) By comparing the ssGSEA score of two immune signatures, the *KRAS*-mutated group showed lower enrichment levels of TIL and inflammation-promoting. (**D**–**F**) The KRAS-mutated group showed a lower level of cytolytic activities and GZMA and PRF1 expression (* P<0.05, ** P<0.01, *** P<0.001).

In order to explore the underlying mechanism of *KRAS* mutations in colon cancer, we performed GSEA to identify correlated pathways. We noticed that *KRAS* mutations had high enrichment in some immune signatures: macrophage M1 and M2, natural killer cell (NK cell) differentiation, class I MHC-mediated antigen processing, B-cell receptor signaling, IL-2, and IL-17 pathways (Figure 3). We assessed the immunity of tumor samples by applying the ssGSEA approach to the transcriptomes of TCGA colon cancer samples (Figure 4A). We incorporated 30 immune-related pathways and infiltrating cells to estimate the immune capacity of colon cancer tissues. We found significantly lower enrichment levels in 13 pathways within the *KRAS*-mutated group: pDCs, Treg, inflammation-promoting, Th1 cells, HLA, T cell co-stimulation, cytolytic activity, tumor infiltration lymphocyte (TIL), T cell co-inhibiting, T helper cells, neutrophils, macrophages, and checkpoint (Supplementary Table 3). Furthermore, we compared the tumor purity, immune score, and stromal score between the *KRAS*-mutated and wild-type groups. The *KRAS*-mutated group had lower immune and stromal scores while its tumor purity was higher than that in the wild-type group. This revealed that the *KRAS* mutation negatively correlated with immune activities (Figure 4B–4D). Moreover, we explored the correlation between *KRAS* mutation and specific immune signatures by analyzing expression levels of signature-related genes and immune cell infiltration. We compared the ssGSEA scores of 16 immune cell infiltration signatures between the *KRAS*-mutated and wild-type groups and found that the infiltration of macrophages (p = 0.033), neutrophils (p = 0.026), pDCs (p < 0.001), T-helper cells (p = 0.024), Th1 cells (p = 0.011), and Tregs (p = 0.001) were lower in *KRAS*-mutated group (Figure 5A). The TIL signature—composed of 117 genes—showed significantly higher enrichment in the wild-type than the *KRAS*-mutated groups (p = 0.015), and 64 out of 117 genes in this signature showed lower expression levels in the *KRAS*-mutated group (Figure 5B, Supplementary Table 4). As for the inflammation-promoting signature, the *KRAS*-mutated group showed lower enrichment and 9 out of 15 genes in this signature had decreased expression (p = 0.002, Figure 5C, Supplementary Table 5). Granzyme A (GZMA) and perforin 1 (PRF1) secreted by cytotoxic T-cells and NK cells are able to kill tumor cells [27]. GZMA is a tryptase that leads to caspase- independent apoptosis, while PRF1 is a pore-forming enzyme that facilitates the entry of granzymes into the target cells. Both effector molecules were considerably overexpressed upon CD8+ T cell activation [28]. The cytolytic activity was calculated as the mean of GZMA and PRF1 expression [29, 30]. The *KRAS*-mutated group had lower GZMA and PRF1 expression (Figure 5D–5F). These observations demonstrate that *KRAS* mutations are negatively correlated with immune cell infiltration, cytotoxic cell activity, and inflammatory response in colon cancer.

### Exploring the mechanism and function of *KRAS* mutation in immune activities

**Figure 6:**
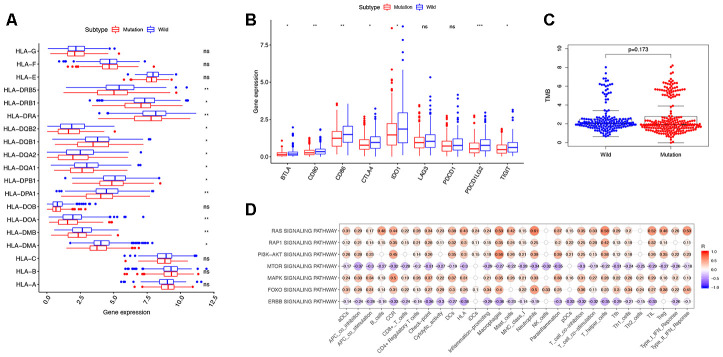
**Exploring the mechanism of *KRAS*-related immune alteration.** (**A**) 12 of the 19 HLA genes showed significantly lower expression levels in the *KRAS*-mutated group compared to the wild-type group. (**B**) The expression of 7 checkpoint-related genes (*BTLA*, *CD80*, *CD86*, *CTLA4*, *IDO1*, *PDCD1LG2*, and *TIGIT*) were lower in the *KRAS*-mutated group (* P<0.05, ** P<0.01, *** P<0.001). (**C**) Comparison of TMB between the *KRAS*-mutated and wild-type groups. (**D**) Spearman correlation analysis between 10 *KRAS*-related signaling pathways and 30 immune signatures.

**Figure 7:**
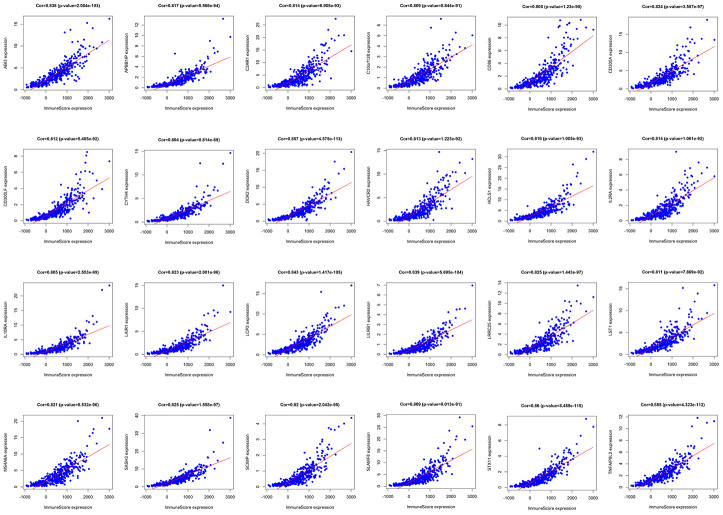
**Pearson correlation analysis of immune score and differentially expressed genes.** There were 24 differentially expressed genes that had a strong correlation with immune score, with cor>0.8 and P<0.05.

We analyzed TMB, HLA gene expression, and checkpoint-related genes in the *KRAS*-mutated and wild-type groups. Among the 19 HLA genes, 12 showed significantly lower expression levels in the *KRAS*-mutated group compared to the wild-type group (Figure 6A). For the checkpoint-related genes, we observed that *BTLA*, *CD80*, *CD86*, *CTLA4*, *IDO1*, *PDCD1LG2*, and *TIGIT* had decreased expression in the *KRAS*-mutated group (Figure 6B). Gene mutations can generate neoantigens that mediate anti-tumor immune activities, and TMB has also been shown to have a strong correlation with tumor immunity. However, there were no significant differences in TMB between the *KRAS*-mutated and wild-type groups. This suggests that TMB cannot explain their difference in immunity (Figure 6C). RAS-related pathways obtained from KEGG included RAP1, PI3K-ATK, mTOR, MAPK, FOXO, and ERBB signaling pathways. With the exception of mTOR and ERBB signaling pathways, most of the RAS-related pathways positively correlated with the immune signature. RAS (r = 0.61) and FOXO (r = 0.5) signaling pathways had strong positive correlations with neutrophils. RAS (r = 0.53) and PI3K-ATK signaling pathways (r = 0.56) exhibited a positive correlation with macrophages. There was also a positive correlation between the RAS signaling pathway and T-helper cells. Interestingly, neutrophils, macrophages, and T-helpers also showed differences in ssGSEA between the *KRAS*-mutated and wild-type groups. Finally, we used the Wilcoxon test to screen for differently expressed genes between the *KRAS*-mutated and wild-type groups, with FDR < 0.05. We calculated the Pearson correlation coefficients between these differentially expressed genes (Supplementary Table 6) and the immune score of every colon cancer sample from the TCGA datasets. We identified 24 genes, which had cor > 0.8 and p < 0.05 (Figure 7, Supplementary Table 7). The annotations of these genes are shown in Supplementary Table 8. *KRAS* mutations may affect the expression of these genes to further adjust the immune microenvironment.

## DISCUSSION

Based on the gene mutational landscape in colon cancer, we found that *KRAS* mutations ranked in the top five of mutated genes in the TCGA and ICGC databases. *KRAS* is a member of the RAS family, which are G-proteins involved in intracellular signaling [31]. The contribution of RAS to anti-apoptosis, proliferation, and metastasis ability of cancer cells has been well validated [32, 33]. These activities were achieved via activation of several downstream effector pathways such as canonical PI3K-AKT-mTOR and RAF-MEK-ERK kinase cascades [34]. Numerous studies indicated that *KRAS* mutations serve as prognostic and predictive biomarkers in multiple types of cancer, as it can provide information for patients’ survival outcomes and suggestions on the use of EGFR-inhibitors. Activating *KRAS* was one of the most frequent oncogenic mutations in early colon cancer, recorded in 27–43% of patients [35]. Ablation of the *KRAS* mutation in colon cells can lead to tumor regression in mice, suggesting its importance in colon carcinogenesis [36]. In our study, we observed that rs121913529 was the most frequent mutational locus in colon cancer samples from the TCGA database. In addition, rs121913530 and rs121913529 correlated negatively with the survival rate of colon cancer patients. As the prognostic value of rs121913530 has only been proven in breast cancer among Chinese people, further validations are needed [37]. Given the significant oncogenic function of *KRAS*, drugs targeting *KRAS* may provide a promising selection for tumor therapy.

Recently, Liao et al. showed that the *KRAS-IRF2* axis can drive immune suppression in colorectal cancer. A consensus molecular subtype classification provides some clues about the relationship between *KRAS* and immunity [38]. It was also reported that co-occurrence of *KRAS* and *TP53* played a role in activating anti-tumor immunity and immune escape [39]. Still, the genetic heterogeneity of *KRAS*-mutant tumors impedes the development of immunotherapy for patients. Recent studies suggest that patients with activating mutations in *KRAS* may benefit from a PD-1 blockade, but the clinical experiments involved lung cancers with unclear underlying mechanisms [40, 41]. Few research studies have systemically analyzed the effect of *KRAS* mutations on immune activity in colon cancer. Therefore, we analyzed the relationship among *KRAS* mutations, immune cells, and pathways. From our results, *KRAS* mutations have a strong negative correlation with the immune response and cell infiltration. By comparing the ssGSEA enrichment scores, we found that the *KRAS*-mutated group had lower enrichment levels of TIL, inflammation, and cytolytic activity. *KRAS*-mutated groups had lower enrichment levels of macrophages, NK-cell differentiation, class I MHC-mediated antigen processing, B-cell receptor signaling, IL-2 and IL-17 pathways in ssGSEA. The relative infiltration levels of macrophages, neutrophils, dendritic cells, T-helper and T-regulatory cells were also lower in the *KRAS*-mutated group. We evaluated the patients’ cytolytic activity by calculating the geometric mean of GZMA and PRF1 expression. GZMA can lead to apoptosis without cascade activation, and PRF1 can help the granzyme enter and kill tumor cells [29]. Up-regulation of these two genes caused CD8+ T-cells and NK cells to activate and anti-CTLA-4 or anti-PD-L1 treatment to induce productive immune responses in the body. Some checkpoint-related genes (*BTLA, CD80, CD86, CTLA4, IDO1, PDCD1LG2*, and *TIGIT*) had decreased expression in the *KRAS*-mutated group, providing potential opportunities for immunotherapy in colon cancer.

Recent studies demonstrated that TMB could serve as a predictive biomarker for immunotherapy. Neoantigens presented by tumor cells can activate the infiltration of CD8^+^ T-cells to recognize antigens and release cytolytic enzyme into tumor cells [42]. However, the correlation among gene mutations, TMB, and immune activities in colon cancer remains unknown. Our results showed no significant difference in TMB between the *KRAS*-mutated and wild-type groups. This indicated that TMB could not explain the relationship between *KRAS* mutations and the immune response. HLA gene expression was different between the *KRAS*-mutated and wild-type groups, supporting the role of HLA genes in regulating *KRAS*-related immune activities. To explore the mechanism of immune differences between the two groups, we identified 24 differentially expressed genes, which strongly correlated with the immune score. These genes may participate in the upstream or downstream pathways of *KRAS*-related immune alteration. For example, overexpression of CD86 is one of the most recognized characteristics of M1 macrophages and a predictive biomarker for immunotherapy [43]. Redente et al. reported that the number of macrophages was increased in the background of a mutated oncogenic *KRAS*, providing support for mutated *KRAS* directing macrophage infiltration in tumor tissue [44]. There is a need to further investigate the predictive value of *KRAS* for immune activity in colon cancer. Future research should detail the mechanism of how *KRAS* mutation and its downstream signaling pathways alter the immune activities and clinical phenotypes of colon cancer.

## CONCLUSIONS

In colon cancer, the *KRAS* gene was of high mutational frequency and rs121913529 was the most frequently mutated locus. Two loci of *KRAS* (rs121913529 and rs121913530) had prognostic value in patients with colon cancer. *KRAS* mutations had a strong negative correlation with TIL, inflammation, cytolytic activities, and HLA genes. Seven checkpoint-related genes (*BTLA,* *CD80, CD86, CTLA4, IDO1, PDCD1LG2,* and *TIGIT*) had decreased expression in the *KRAS*-mutated group, providing potential opportunities for immunotherapy in colon cancer. The *KRAS*-mutated group showed lower infiltration of macrophages, neutrophils, T-helper and T-regulatory cells. In order to explore the underlying mechanism, we also detected 24 immune-related genes that differed in expression in the *KRAS*-mutated and wild-type groups.

## MATERIALS AND METHODS

### Downloaded data

We obtained somatic mutation data and clinical information of colon cancer samples from the TCGA database (n=399) via the GDC data portal (https://portal.gdc.cancer.gov/repository) and ICGC database (n=321, http://dcc.icgc.org/releases/current/Projects). We downloaded the RNA-seq data (level 3, HTSeq-FPKM) of 473 colon cancer patients with clinical information from the TCGA database. The mutation data was paired with the RNA-seq data according to patient ID. The annotations of genes were obtained from the Uniprot database (https://www.uniprot.org/).

### Analysis of somatic gene mutations in colon cancer

For TCGA, we downloaded the “Masked Somatic Mutation” subtype of somatic mutation data and used the VarScan software for processing. We used an R package called “maftools” [45] to analyze and visualize the Mutation Annotation Format of somatic variants. We annotated TSV files containing somatic variant information from ICGC according to the hg19 reference genome. Both cohorts were visualized by the GenVisR package. The definition of TMB is the total number of coding errors of somatic genes per million bases, including base-pair substitutions, insertions, and deletions [20]. We counted all base-pair substitutions in the coding region of specific genes, except silent mutations that failed to alter amino acids. To calculate the TMB score of each sample, we divided the total number of mutations by the exome size (38 Mb). We analyzed the difference in overall survival rates between the mutated and wide-type groups using an R package called “survival.”

### Implementation of ssGSEA and GSEA

We performed ssGSEA to acquire the enrichment score for each immune-related pair [46] and sample using an R package called “GSVA” [47] (Supplementary Table 1). We obtained 30 immune gene sets from several literature sources, including immune cell types and functions [48], tumor-infiltrating lymphocytes (TILs) [49], proinflammatory [50], para-inflammation (PI) [51], cytokine and cytokine receptor (CCR) [52], human leukocyte antigen (HLA) [53], regulatory T (Treg) cells [54], and immune checkpoints [55]. The ssGSEA applied gene signatures expressed by immune cells and pathways to the colon cancer samples. The approach used in our study involved immune cells and pathways in innate and adaptive immunity. We used an R package called “ESTIMATE” to calculate the immune score, tumor purity, and stromal score of every tumor sample [56]. The stromal score is defined as the presence of stroma in tumor tissue. The immune score is defined as the infiltration of immune cells in tumor tissue. The tumor purity score is defined as tumor purity. We performed GSEA to explore the correlated pathways of *KRAS* mutation. Gene ontology gene sets “c2.cp.v7.0.symbols.gmt” obtained from the Molecular Signatures Database (MSigDB, http://software.broadinstitute.org/gsea/downloads.jsp) were used for the enrichment analysis. The enriched gene set was considered statistically significant when the p value was less than 0.05. We demonstrated the correlation between *KRAS* mutations and immune cell infiltration by comparing the immune cell infiltration in *KRAS*-mutated and wild-type groups.

### Correlation between the RAS-related pathway and immune activity in colon cancer

We obtained RAS-related pathways from KEGG. We analyzed the activities of RAS-related pathways by ssGSEA scores. We used a first order partial correlation to assess the correlation between immune signatures and RAS pathways [57]. We used the Spearman correlation test to evaluate the correlation with a significance threshold of p < 0.05.

### Statistical analysis

We used the Wilcoxon test to screen for gene expression differences between the *KRAS*-mutated and wild-type groups. We analyzed the relationship between differentially expressed genes and the immune score by calculating the Pearson correlation coefficients. The expression levels of checkpoint-related and HLA genes in the *KRAS*-mutated and wild-type groups were analyzed by the Mann–Whitney U test.

## Supplementary Material

Supplementary Figure 1

Supplementary Tables 1, 2 and 3

Supplementary Table 4

Supplementary Table 5

Supplementary Table 6

Supplementary Table 7

Supplementary Table 8
