# Supplementary material for: KRAS mutations are negatively correlated with immunity in colon cancer
Source: Aging (Albany NY). 2020 Nov 26;13(1):750–68. doi: 10.18632/aging.202182 (PMC7834984; doi:10.18632/aging.202182)
Supplement: Supplementary Figure 1 [file aging-13-202182-s001.pdf]

SUPPLEMENTARY FIGURE

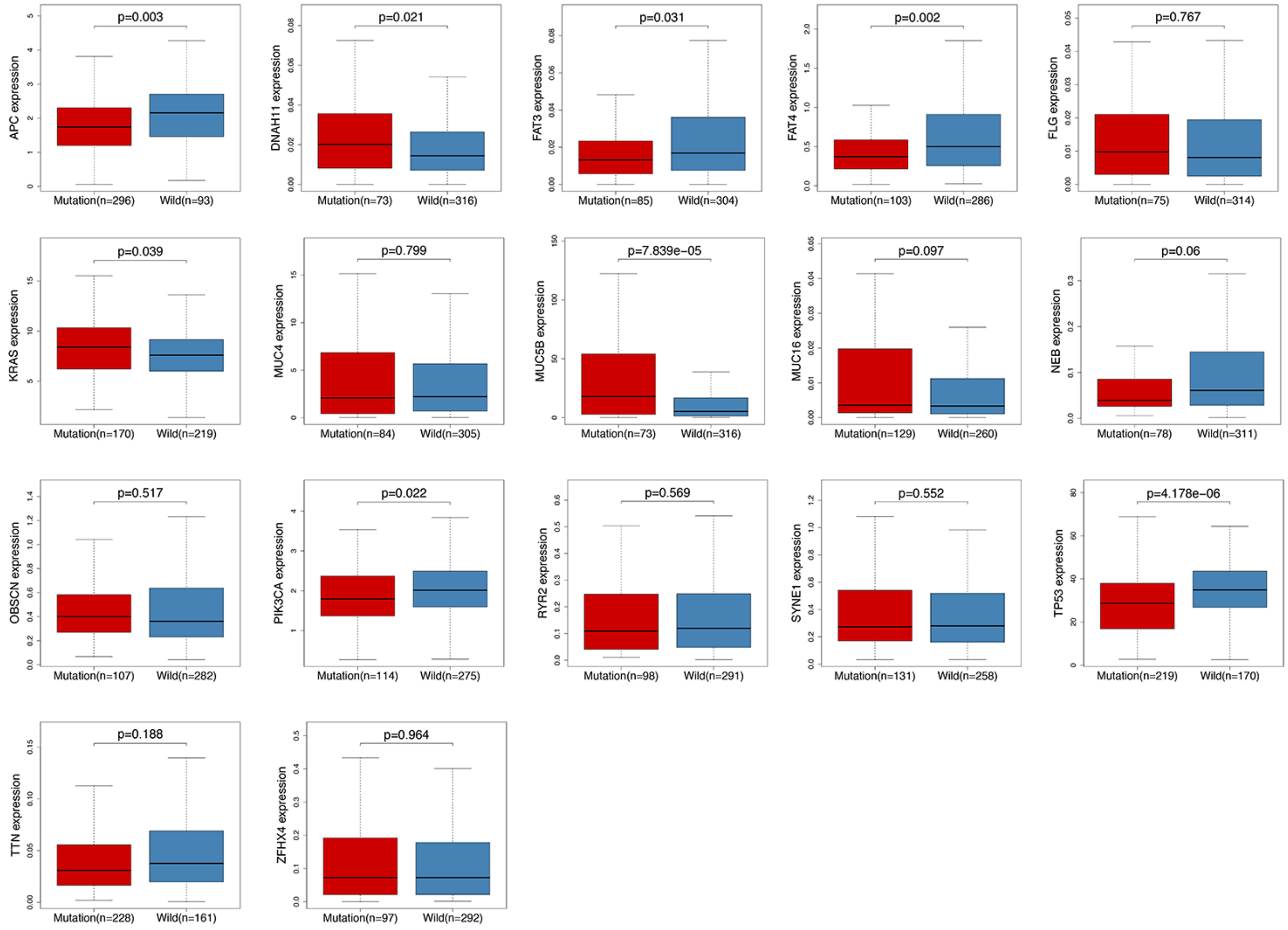

Supplementary Figure 1. Correlation between somatic mutations with gene expression. Among the 17 genes detected in Figure 1C, the mutation of 8 genes were correlated with their expression levels: *APC*, *DNAH11*, *FAT3*, *FAT4*, *KRAS*, *MUC5B*, *PIK3CA*, and *TP53*. The correlation analysis was performed based on TCGA.
