# Supplementary material for: KRAS mutations are negatively correlated with immunity in colon cancer
Source: Aging (Albany NY). 2020 Nov 26;13(1):750–68. doi: 10.18632/aging.202182 (PMC7834984; doi:10.18632/aging.202182)
Supplement: Supplementary Tables 1, 2 and 3 [file aging-13-202182-s002.pdf]

SUPPLEMENTARY TABLES

Supplementary Table 1. The genetic composition of 30 immune signatures.

aDCs	CD83;LAMP3;CCL1
APC_co_inhibition	C10orf54;CD274;LGALS9;PDCD1LG2;PVRL3
APC_co_stimulation	CD40;CD58;CD70;ICOSLG;SLAMF1;TNFSF14;TNFSF15;TNFSF18;TNFSF4;TNFSF8;TNFSF9
B_cells	BACH2;BANK1;BLK;BTLA;CD79A;CD79B;FCRL1;FCRL3;HVCN1;RALGPS2
CCR	CCL16;TPO;TGFBR2;CXCL2;CCL14;TGFBR3;IL11RA;CCL11;IL4I1;IL33;CXCL12;CXCL10;BMPER;BMP8A;CXCL11;IL21R;IL17B;TNFRSF9;ILF2;CX3CR1;CCR8;TNFSF12;CSF3;TNFSF4;BMP3;CX3CL1;BMP5;CXCR2;TNFRSF10D;BMP2;CXCL14;CCL28;CXCL3;BMP6;CCL21;CXCL9;CCL23;IL6;TNFRSF18;IL17RD;IL17D;IL27;CCL7;IL1R1;CXCR4;CXCR2P1;TGFB1I1;IFNGR1;IL9R;IL1RAPL1;IL11;CSF1;IL20RA;IL25;TNFRSF4;IL18;ILF3;CCL20;TNFRSF12A;IL6ST;CXCL13;IL12B;TNFRSF8;IL6R;BMPR2;IFNE;IL1RAPL2;IL3RA;BMP4;CCL24;TNFSF13B;CCR4;IL2RA;IL32;TNFRSF10C;IL22RA1;BMPR1A;CXCR5;CXCR3;IFNA8;IL17REL;IFNB1;IFNAR1;TNFRSF1B;CCL17;IFNL1;IL16;IL1RL1;ILK;CCL25;ILDR2;CXCR1;IL36RN;IL34;TGFB1;IFNG;IL19;ILKAP;BMP2K;CCR10;ILDR1;EPO;CCR7;IL17C;IL23A;CCR5;IL7;EPOR;CCL13;IL2RG;IL31RA;TNFAIP6;IFNL2;BMP1;IL12RB1;TNFAIP8;IL4R;TNFRSF6B;TNFAIP8L1;TNFRSF10B;IFNL3;CCL5;CXCL6;CXCL1;CCR3;TNFSF11;CSF1R;IL21;IL1RAP;IL12RB2;CCL1;IL17RA;CCR1;IL1RN;TNFRSF11B;TNFRSF14;IL13;IL2RB;BMP8B;CCL2;IL24;IL18RAP;TGFBI;TNFSF10;TNFRSF11A;CXCL5;IL5RA;TNFSF9;IL1RL2;TNFRSF13C;IL36G;IL15RA;TNFRSF21;CXCL8;IL22RA2;TNFAIP8L2;IL18R1;IFNLR1;CXCR6;CCL3L3;TNFRSF1A;IL17RE;IFNGR2;IL17RC;TNFAIP8L3;ILVBL;TGFBRAP1;CCL4L1;CSF2RA;CCRN4L;CCL26;TNFAIP1;CCRL2;IFNA10;TNFRSF17;IFNA13;IL20;IL18BP;CCL3L1;TNFSF12-TNFSF13;IL5;IL23R;IL26;TNF;TGFA;CSF2;IL1F10;CXCL17;TNFSF13;IFNA4;IL37;IL12A;IL7R;IFNA1;IL1A;IL4;IL2;CCL22;CSF3R;IL10;IFNK;TGFB2;IL1R2;IL1B;IL17F;IL27RA;IL15;TNFSF8;IL36B;XCL1;CXCL16;TNFRSF19;IL3;CCL3;IFNA2;BMPR1B;IFNA21;TNFSF18;CCL8;IL17RB;TNFRSF25;IL22;IL10RB;IFNAR2;CCL18;IFNA16;CSF2RB;IL36A;TNFAIP3;IL13RA2;IL13RA1;CCR9;TNFRSF10A;IFNA7;IFNW1;XCL2;TNFSF14;CCR2;BMP15;BMP10;CCL15;CCL14;TGFBR1;IFNA5;BMP7;IFNA14;IL20RB;IL10RA;IFNA17;CCR6;TGFB3;CCL15;CCL4;CCL27;TNFRSF13B;TNFAIP2;IL31;IL17A;TNFSF15;CCL19;IFNA6;IL9
CD8+_T_cells	CD8A
CD4+ Regulatory T cells	C15orf53;CTLA4;FOXP3;GPR15;IL32;IL4;IL5
Check-point	IDO1;LAG3;CTLA4;TNFRSF9;ICOS;CD80;PDCD1LG2;TIGIT;CD70;TNFSF9;ICOSLG;KIR3DL1;CD86;PDCD1;LAIR1;TNFRSF8;TNFSF15;TNFRSF14;IDO2;CD276;CD40;TNFRSF4;TNFSF14;HHLA2;CD244;CD274;HAVCR2;CD27;BTLA;LGALS9;TMIGD2;CD28;CD48;TNFRSF25;CD40LG;ADORA2A;VTCN1;CD160;CD44;TNFSF18;TNFRSF18;BTNL2;C10orf54;CD200R1;TNFSF4;CD200;NRP1
Cytolytic_activity	PRF1;GZMA
DCs	CCL17;CCL22;CD209;CCL13
HLA	HLA-E;HLA-DPB2;HLA-C HLA-J;HLA-DQB1;HLA-DQB2;HLA-DQA2;HLA-DQA1;HLA-A;HLA-DMA;HLA-DOB;HLA-DRB1;HLA-H;HLA-B;HLA-DRB5;HLA-DOA;HLA-DPB1;HLA-DRA;HLA-DRB6;HLA-L;HLA-F;HLA-G;HLA-DMB;HLA-DPA1
iDCs	CD1A;CD1E
Inflammation-promoting	CCL5;CD19;CD8B;CXCL10;CXCL13;CXCL9;GNLY;GZMB;IFNG;IL12A;IL12B;IRF1;PRF1;STAT1;TBX21
Macrophages	C11orf45;CD68;CLEC5A;CYBB;FUCA1;GPNMB;HS3ST2;LGMN;MMP9;TM4SF19
Mast_cells	CMA1;MS4A2;TPSAB1
MHC_class_I	B2M;HLA-A;TAP1
Neutrophils	EVI2B;HSD17B11;KDM6B;MEGF9;MNDA;NLRP12;PADI4;SELL;TRANK1;VNN3
NK_cells	KLRC1;KLRF1
Parainflammation	CXCL10;PLAT;CCND1;LGMN;PLAUR;AIM2;MMP7;ICAM1;MX2;CXCL9;ANXA1;TLR2;PLA2G2D;ITGA2;MX1;HMOX1;CD276;TIRAP;IL33;PTGES;TNFRSF12A;SCARB1;CD14;BLNK;IFIT3;RETNLB;IFIT2;ISG15;OAS2;REL;OAS3;CD44;PPARG;BST2;OAS1;NOX1;PLA2G2A;IFIT1;IFITM3;IL1RN
pDCs	CLEC4C;CXCR3;GZMB;IL3RA;IRF7;IRF8;LILRA4;PHEX;PLD4;PTCRA
T_cell_co-inhibition	BTLA;C10orf54;CD160;CD244;CD274;CTLA4;HAVCR2;LAG3;LAIR1;TIGIT
T_cell_co-stimulation	CD2;CD226;CD27;CD28;CD40LG;ICOS;SLAMF1;TNFRSF18;TNFRSF25;TNFRSF4;TNFRSF8;TNFRSF9;TNFSF14
T_helper_cells	CD4
Tfh	PDCD1;CXCL13;CXCR5
Th1_cells	IFNG;TBX21;CTLA4;STAT4;CD38;IL12RB2;LTA;CSF2
Th2_cells	PMCH;LAIR2;SMAD2;CXCR6;GATA3;IL26

TIL	ITM2C;CD38;THEMIS2;GLYR1;ICOS;F5;TIGIT;KLRD1;IRF4;PRKCQ;FCRL5;SIRPG;LPXN;IL2RG;CCL5;LCK;TRAF3IP3;CD86;MAL;LILRB1;DOK2;CD6;PAG1;LAX1;PLEK;PIK3CD;SLAMF1;XCL1;GPR171;XCL2;TBX21;CD2;CD53;KLHL6;SLAMF6;CD40;SIT1;TNFRSF4;CD79A;CD247;LCP2;CD3D;CD27;SH2D1A;FYB;ARHGAP30;ACAP1;CST7;CD3G;IL2RB;CD3E;FCRL3;CORO1A;ITK;TCL1A;CYBB;CSF2RB;IKZF1;NCF4;DOCK2;CCR2;PTPRC;PLAC8;NCKAP1L;IL7R;6-Sep;CD28;STAT4;CD8A;LY9;CD48;HCST;PTPRCAP;SASH3;ARHGAP25;LAT;TRAT1;IL10RA;PAX5;CCR7;DOCK11;PARVG;SPNS1;CD52;HCLS1;ARHGAP9;GIMAP6;PRKCB;MS4A1;GPR18;TBC1D10C;GVINP1;P2RY8;EVI2B;VAMP5;KLRK1;SELL;MPEG1;MS4A6A;ARHGAP15;MFNG;GZMK;SELPLG;TARP;GIMAP7;FAM65B;INPP5D;ITGA4;MZB1;GPSM3;STK10;CLEC2D;IL16;NLRC3;GIMAP5;GIMAP4;IFFO1;CFH;PVRIG;CFHR1
Treg	IL12RB2;TMPRSS6;CTSC;LAPTM4B;TFRC;RNF145;NETO2;ADAT2;CHST2;CTLA4;NFE2L3;LIMA1;IL1R2;ICOS;HSDL2;HTATIP2;FKBP1A;TIGIT;CCR8;LTA;SLC35F2;IL21R;AHCYL1;SOCS2;ETV7;BCL2L1;RRAGB;ACSL4;CHRNA6;BATF;LAX1;ADPRH;TNFRSF4;ANKRD10;CD274;CASP1;LY75;NPTN;SSTR3;GRSF1;CSF2RB;TMEM184C;NDFIP2;ZBTB38;ERI1;TRAF3;NAB1;HS3ST3B1;LAYN;JAK1;VDR;LEPROT;GCNT1;PTPRJ;IKZF2;CSF1;ENTPD1;TNFRSF18;METTL7A;KSR1;SSH1;CADM1;IL1R1;ACP5;CHST7;THADA;CD177;NFAT5;ZNF282;MAGEH1
Type_I_IFN_Reponse	DDX4;IFIT1;IFIT2;IFIT3;IRF7;ISG20;MX1;MX2;RSAD2;TNFSF10
Type_II_IFN_Reponse	GPR146;SELP;AHR

Supplementary Table 2. The mutational frequency of genes in the whole genome.

	Gene	Number
**rs121913529**	KRAS	90
**rs113488022**	BRAF	49
**rs112445441**	KRAS	31
**rs104886003**	PIK3CA	30
**rs28934578**	TP53	26
**rs121913332**	APC	25
**rs781215815**	RNF43	22
**rs121913530**	KRAS	19
**rs782665429**	DOCK3	18
**rs121913279**	PIK3CA	17
**rs764719749**	ACVR2A	15
**rs121913333**	APC	14
**rs11540652**	TP53	13
**rs772920507**	BMPR2	13
**rs121913527**	KRAS	13
**rs28934574**	TP53	12
**rs28934576**	TP53	12
**rs587781392**	APC	12
**rs121913273**	PIK3CA	12
**rs770033147**	SVIL	11
**rs121913331**	APC	11
**rs121913287**	PIK3CA	11
**rs397516436**	TP53	10
**rs62619935**	APC	10
**rs549924573**	RCVRN	10
**rs759765382**	RPL22	9
**rs763321097**	TEAD2	9
**rs372217972**	QKI	9
**rs767148651**	HNRNPL	8
**rs137854574**	APC	8
**rs759448855**	GLI1	8
**rs121912651**	TP53	8
**rs137854580**	APC	8
**rs121913343**	TP53	8
**rs199566425**	C9orf47	7
**rs377767347**	SMAD4	7
**rs778658185**	BCORL1	7
**rs756774416**	LMNTD2	7
**rs781677398**	ZDHHC8	7
**rs766438669**	CSMD3	7
**rs397515734**	APC	7
**rs775633847**	RAB28	7
**rs762648935**	MBD6	7
**rs143104828**	CSF2RA	6
**rs746563015**	CNTLN	6
**rs762448666**	ELMSAN1	6
**rs121913237**	NRAS	6
**rs121913329**	APC	6
**rs777980924**	FHOD3	6
**rs762805003**	CD93	6
**rs761047150**	C9orf131	6
**rs563361433**	ONECUT1	6
**rs748072217**	KIAA1024	6
**rs778388564**	PCDH19	6

**rs151073460**	FUS	6
**rs748349403**	SLC8A2	6
**rs765803753**	MICAL3	6
**rs748850271**	PHGR1	6

The table shows mutational frequency of locus more than 5.

Supplementary Table 3. The enrichment level of 30 immune signature in KRAS-mutated and wild-type groups were compared by Mann-Whitney test.

Immune Term	P Value
pDCs	**0.000284**
Treg	**0.000637**
Inflammation_promoting	**0.002**
Th1_cells	**0.011**
HLA	**0.012**
T_cell_co_stimulation	**0.014**
Cytolytic_activity	**0.015**
TIL	**0.015**
T_cell_co_inhibition	**0.023**
T_helper_cells	**0.024**
Neutrophils	**0.026**
Macrophages	**0.033**
Check_point	**0.037**
DCs	0.056
Tfh	0.067
CCR	0.069
aDCs	0.076
Type_II_IFN_Response	0.083
CD4+_Regulatory_T_cells	0.132
Type_I_IFN_Response	0.158
Th2_cells	0.176
B_cells	0.205
CD8+_T_Cells	0.225
Mast_cells	0.275
APC_co_stimulation	0.289
MHC_class_I	0.468
NK_cells	0.608
iDcs	0.67
Parainflammation	0.831
APC_co_inhibition	0.998
