# Supplementary material for: KRAS mutations are negatively correlated with immunity in colon cancer
Source: Aging (Albany NY). 2020 Nov 26;13(1):750–68. doi: 10.18632/aging.202182 (PMC7834984; doi:10.18632/aging.202182)
Supplement: Supplementary Table 4 [file aging-13-202182-s003.docx]

**Supplementary Table 4.** **Comparing the expression of genes in TIL signature between KRAS-mutated and wild-type groups.**

| Gene | conMean | treatMean | logFC | pValue |
| --- | --- | --- | --- | --- |
| CD40 | 4.189471 | 6.153315 | 0.554596 | **6.56E-06** |
| PLAC8 | 8.787574 | 14.25002 | 0.697427 | **4.75E-05** |
| CD38 | 0.619689 | 0.940506 | 0.601893 | **5.51E-05** |
| PAG1 | 2.189391 | 2.810327 | 0.360209 | **0.000221** |
| ARHGAP15 | 0.406826 | 0.548111 | 0.430055 | **0.000305** |
| STAT4 | 0.452884 | 0.631282 | 0.479144 | **0.000391** |
| PTPRC | 3.112474 | 4.587663 | 0.559697 | **0.000412** |
| LCP2 | 2.43026 | 3.04501 | 0.325337 | **0.000508** |
| CFH | 2.778433 | 3.481998 | 0.325644 | **0.00056** |
| GIMAP4 | 4.928094 | 6.63103 | 0.428203 | **0.000644** |
| SPNS1 | 0.933392 | 1.082394 | 0.21367 | **0.000871** |
| CD53 | 11.7707 | 15.26035 | 0.374588 | **0.001225** |
| MPEG1 | 4.709325 | 6.218754 | 0.401105 | **0.001319** |
| SLAMF1 | 0.476635 | 0.619543 | 0.37832 | **0.001388** |
| ICOS | 0.537819 | 0.714246 | 0.409302 | **0.001401** |
| CSF2RB | 2.338518 | 3.042802 | 0.379806 | **0.00141** |
| GIMAP6 | 2.386245 | 3.172865 | 0.411044 | **0.001516** |
| CD86 | 1.846501 | 2.457673 | 0.412499 | **0.001531** |
| ITGA4 | 1.15802 | 1.558512 | 0.42851 | **0.001666** |
| IL7R | 3.000151 | 4.162786 | 0.472514 | **0.001757** |
| MS4A6A | 3.019181 | 4.062844 | 0.428333 | **0.001899** |
| GPR18 | 0.282172 | 0.411736 | 0.545147 | **0.00198** |
| CYBB | 7.525273 | 10.20026 | 0.43879 | **0.002033** |
| PLEK | 5.060986 | 6.53685 | 0.369177 | **0.002202** |
| KLHL6 | 0.529618 | 0.691169 | 0.384087 | **0.002839** |
| VAMP5 | 15.3516 | 19.60255 | 0.352652 | **0.002961** |
| EVI2B | 3.474775 | 4.465424 | 0.361877 | **0.003152** |
| CD48 | 2.226152 | 3.084563 | 0.470514 | **0.003861** |
| CD3G | 0.66859 | 0.909157 | 0.443408 | **0.004068** |
| SELL | 2.020687 | 2.974593 | 0.557846 | **0.004104** |
| SIT1 | 1.821925 | 2.627552 | 0.528256 | **0.004188** |
| FYB | 1.622829 | 2.168145 | 0.41795 | **0.004249** |
| NCF4 | 5.895381 | 7.549679 | 0.35683 | **0.005462** |
| THEMIS2 | 3.467527 | 4.227035 | 0.285739 | **0.005762** |
| LAX1 | 0.533824 | 0.766854 | 0.522588 | **0.005959** |
| SH2D1A | 0.516921 | 0.750417 | 0.53775 | **0.006161** |
| CCR2 | 0.489912 | 0.678236 | 0.469266 | **0.006568** |
| TNFRSF4 | 2.083425 | 2.53445 | 0.282716 | **0.006568** |
| PRKCB | 0.50419 | 0.736228 | 0.546187 | **0.00679** |
| CD2 | 4.952975 | 6.559933 | 0.405386 | **0.007174** |
| IL10RA | 2.157754 | 2.763476 | 0.356954 | **0.008045** |
| INPP5D | 8.901643 | 7.73019 | -0.20357 | **0.008199** |
| GPR171 | 0.554292 | 0.769566 | 0.473397 | **0.008688** |
| IKZF1 | 0.805585 | 1.088455 | 0.434174 | **0.009428** |
| TIGIT | 0.574851 | 0.77648 | 0.43376 | **0.011401** |
| DOK2 | 2.790849 | 3.298913 | 0.241287 | **0.012979** |
| GIMAP7 | 3.612468 | 4.711989 | 0.383351 | **0.014414** |
| LILRB1 | 0.806866 | 0.987109 | 0.290881 | **0.014709** |
| IRF4 | 0.774582 | 0.989241 | 0.352903 | **0.015395** |
| FCRL5 | 0.268518 | 0.411345 | 0.61533 | **0.015472** |
| HCLS1 | 4.154831 | 5.006624 | 0.269048 | **0.015629** |
| KLRD1 | 0.145747 | 0.216606 | 0.571607 | **0.016413** |
| CD28 | 0.384722 | 0.498922 | 0.374998 | **0.017404** |
| CD247 | 0.992606 | 1.269027 | 0.354429 | **0.01749** |
| SASH3 | 4.009265 | 4.94913 | 0.303837 | **0.017534** |
| ITK | 0.360032 | 0.498554 | 0.469626 | **0.017841** |
| IL2RB | 2.668595 | 3.266459 | 0.291647 | **0.018063** |
| DOCK2 | 0.903279 | 1.161616 | 0.36289 | **0.022175** |
| NCKAP1L | 1.557218 | 1.971741 | 0.340499 | **0.022497** |
| TRAF3IP3 | 0.452298 | 0.605016 | 0.4197 | **0.022934** |
| GIMAP5 | 0.12015 | 0.164461 | 0.4529 | **0.030984** |
| PARVG | 0.75081 | 0.901385 | 0.263694 | **0.031344** |
| CD3D | 5.243576 | 6.618203 | 0.335888 | **0.034655** |
| GZMK | 0.779751 | 1.153175 | 0.564526 | **0.042739** |
| PAX5 | 0.360994 | 0.403169 | 0.159408 | 0.055442 |
| TBX21 | 0.247669 | 0.293948 | 0.247147 | 0.061036 |
| ARHGAP9 | 1.386989 | 1.656903 | 0.256533 | 0.064267 |
| F5 | 1.128275 | 1.845107 | 0.709586 | 0.068607 |
| SIRPG | 0.795898 | 1.026563 | 0.367166 | 0.070722 |
| P2RY8 | 0.888795 | 1.092472 | 0.297673 | 0.076626 |
| LY9 | 0.150725 | 0.195445 | 0.374843 | 0.076778 |
| TBC1D10C | 1.072392 | 1.332222 | 0.313002 | 0.077391 |
| IL16 | 0.634576 | 0.810794 | 0.353543 | 0.078629 |
| KLRK1 | 0.096408 | 0.100054 | 0.05356 | 0.078788 |
| XCL1 | 0.234201 | 0.328625 | 0.488695 | 0.08777 |
| CD3E | 3.593037 | 4.494177 | 0.322853 | 0.088433 |
| ARHGAP30 | 2.93746 | 3.56697 | 0.28013 | 0.090507 |
| NLRC3 | 0.586848 | 0.708892 | 0.272579 | 0.090682 |
| PVRIG | 0.057535 | 0.06948 | 0.272147 | 0.091142 |
| STK10 | 5.017511 | 4.832944 | -0.05407 | 0.092088 |
| CST7 | 6.516815 | 5.589782 | -0.22137 | 0.096047 |
| ARHGAP25 | 1.204956 | 1.427067 | 0.244073 | 0.096047 |
| MZB1 | 5.723666 | 7.736817 | 0.434801 | 0.101282 |
| MS4A1 | 0.559607 | 0.869832 | 0.636323 | 0.109837 |
| CD79A | 5.5342 | 7.698192 | 0.476145 | 0.130941 |
| TRAT1 | 0.233276 | 0.319863 | 0.455415 | 0.133041 |
| CD52 | 10.75573 | 13.26273 | 0.302273 | 0.139564 |
| CLEC2D | 0.559146 | 0.688304 | 0.299822 | 0.142035 |
| CD8A | 1.953257 | 2.520418 | 0.367781 | 0.15121 |
| SLAMF6 | 1.584088 | 1.72956 | 0.126753 | 0.174657 |
| CCL5 | 12.90831 | 15.46207 | 0.260433 | 0.186282 |
| FCRL3 | 0.149979 | 0.231942 | 0.629004 | 0.188725 |
| PIK3CD | 1.5763 | 1.821459 | 0.208553 | 0.222829 |
| SELPLG | 5.956935 | 6.477947 | 0.120966 | 0.236325 |
| PRKCQ | 1.383617 | 1.51556 | 0.131406 | 0.243285 |
| CCR7 | 1.132958 | 1.473794 | 0.37944 | 0.249634 |
| CD27 | 2.352356 | 2.787756 | 0.244998 | 0.251144 |
| HCST | 3.44191 | 3.781747 | 0.135843 | 0.261896 |
| 6-Sep | 4.148267 | 4.642381 | 0.162356 | 0.305458 |
| IFFO1 | 1.312398 | 1.443015 | 0.136881 | 0.373896 |
| ACAP1 | 1.164583 | 1.327589 | 0.188995 | 0.401064 |
| XCL2 | 0.354071 | 0.424048 | 0.260189 | 0.431298 |
| IL2RG | 38.68444 | 37.1754 | -0.0574 | 0.435292 |
| CD6 | 1.343842 | 1.52975 | 0.186932 | 0.470021 |
| GLYR1 | 21.51513 | 21.24718 | -0.01808 | 0.487081 |
| CFHR1 | 0.010951 | 0.011705 | 0.096009 | 0.614101 |
| DOCK11 | 3.479544 | 3.601205 | 0.049582 | 0.750885 |
| LCK | 4.33474 | 6.006219 | 0.470512 | 0.773931 |
| LAT | 0.122119 | 0.138512 | 0.181727 | 0.867564 |
| GPSM3 | 9.422548 | 9.717734 | 0.044503 | 0.878752 |
| CORO1A | 11.01737 | 11.04368 | 0.003441 | 0.899747 |
| MAL | 0.361765 | 0.415389 | 0.19941 | 0.900469 |
| ITM2C | 193.9482 | 194.3357 | 0.00288 | 0.914995 |
| MFNG | 3.015833 | 3.553872 | 0.236835 | 0.949956 |
| LPXN | 5.898188 | 5.680673 | -0.05421 | 0.979898 |
| TCL1A | 0.434867 | 0.51438 | 0.24226 | 0.980162 |
| PTPRCAP | 0 | 0 | NaN | NaN |
