# Supplementary material for: KRAS mutations are negatively correlated with immunity in colon cancer
Source: Aging (Albany NY). 2020 Nov 26;13(1):750–68. doi: 10.18632/aging.202182 (PMC7834984; doi:10.18632/aging.202182)
Supplement: Supplementary Table 5 [file aging-13-202182-s004.pdf]

SUPPLEMENTARY TABLE

Supplementary Table 5. Comparing the expression of genes in inflammation-activities signature between KRAS-mutated and wild-type groups.

Gene	WildMean	MutationMean	logFC	pValue
**IL12A**	0.282451	0.430636	0.608472	**3.36E-06**
**GZMB**	7.518245	13.26506	0.819164	**4.63E-06**
**CXCL9**	12.71954	17.27174	0.441366	**0.001911**
**CXCL10**	24.05325	29.22686	0.281063	**0.006641**
**PRF1**	3.191711	4.06926	0.350436	**0.009838**
**CXCL13**	4.056069	6.041906	0.574921	**0.020125**
**GNLY**	2.000802	2.894216	0.532595	**0.021077**
**IFNG**	0.27372	0.389159	0.507657	**0.028949**
**STAT1**	37.46403	44.23563	0.239703	**0.049644**
**TBX21**	0.247669	0.293948	0.247147	0.061036
**IRF1**	15.09839	17.20898	0.188767	0.064267
**CCL5**	12.90831	15.46207	0.260433	0.186282
**CD8B**	1.457315	1.674802	0.200678	0.279428
**CD19**	0.511267	0.742265	0.537859	0.283726
**IL12B**	0.033961	0.038595	0.184569	0.507014
