# Supplementary material for: KRAS mutations are negatively correlated with immunity in colon cancer
Source: Aging (Albany NY). 2020 Nov 26;13(1):750–68. doi: 10.18632/aging.202182 (PMC7834984; doi:10.18632/aging.202182)
Supplement: Supplementary Table 6 [file aging-13-202182-s005.docx]

**Supplementary Table 6. Detection of differentially expressed genes in KRAS-mutated and wild-type groups.**

| Gene | wildMeans | mutationMeans | logFC | p_value | FDR | Type |
| --- | --- | --- | --- | --- | --- | --- |
| LECT2 | 7.151376147 | 3.189349112 | -1.16496 | 0.019856 | 0.019856 | Downregulated |
| AC187653.1 | 11.03211009 | 5.372781065 | -1.03797 | 0.005153 | 0.005153 | Downregulated |
| PTF1A | 49.53211009 | 24.40236686 | -1.02134 | 0.000414 | 0.000414 | Downregulated |
| VSTM2B | 30.56880734 | 15.58579882 | -0.97183 | 0.002222 | 0.002222 | Downregulated |
| HIST2H4B | 6.665137615 | 3.473372781 | -0.9403 | 0.008667 | 0.008667 | Downregulated |
| ACCSL | 5.931192661 | 3.106508876 | -0.93303 | 0.047702 | 0.047702 | Downregulated |
| OR1E1 | 2.766055046 | 1.473372781 | -0.90871 | 0.016941 | 0.016941 | Downregulated |
| DNMT3L | 17.87614679 | 9.710059172 | -0.88048 | 0.002993 | 0.002993 | Downregulated |
| AC091980.2 | 6.197247706 | 3.408284024 | -0.86258 | 0.031345 | 0.031345 | Downregulated |
| SLC35G3 | 4.568807339 | 2.520710059 | -0.85799 | 0.005996 | 0.005996 | Downregulated |
| AL365273.2 | 3.871559633 | 2.147928994 | -0.84997 | 0.026142 | 0.026142 | Downregulated |
| C3orf84 | 2.633027523 | 1.467455621 | -0.84341 | 0.009074 | 0.009074 | Downregulated |
| MAGEA9B | 5.123853211 | 2.928994083 | -0.80682 | 0.041326 | 0.041326 | Downregulated |
| ANKRD62 | 29.94036697 | 17.18934911 | -0.80058 | 0.009217 | 0.009217 | Downregulated |
| KRT27 | 15.96788991 | 9.24260355 | -0.7888 | 0.00721 | 0.00721 | Downregulated |
| MYH2 | 28.16513761 | 16.34319527 | -0.78522 | 0.030018 | 0.030018 | Downregulated |
| GOLGA8G | 5.743119266 | 3.349112426 | -0.77806 | 0.029824 | 0.029824 | Downregulated |
| KRTAP13-2 | 14.12385321 | 8.301775148 | -0.76664 | 0.027371 | 0.027371 | Downregulated |
| OR9A2 | 2.385321101 | 1.449704142 | -0.71842 | 0.049092 | 0.049092 | Downregulated |
| AKAP4 | 92.67431193 | 56.72781065 | -0.70811 | 0.00014 | 0.00014 | Downregulated |
| PAPOLB | 15.08715596 | 9.24852071 | -0.70603 | 0.00827 | 0.00827 | Downregulated |
| C17orf105 | 32.26605505 | 19.90532544 | -0.69686 | 0.025339 | 0.025339 | Downregulated |
| ACOD1 | 67.28440367 | 41.65680473 | -0.69172 | 0.001698 | 0.001698 | Downregulated |
| CCDC177 | 37.10550459 | 23.23076923 | -0.6756 | 0.023153 | 0.023153 | Downregulated |
| HRH3 | 26.68807339 | 16.71597633 | -0.67497 | 0.009939 | 0.009939 | Downregulated |
| MLNR | 28.5733945 | 18.15976331 | -0.65393 | 0.020636 | 0.020636 | Downregulated |
| PLET1 | 16.67889908 | 10.65680473 | -0.64625 | 0.019893 | 0.019893 | Downregulated |
| NOTO | 14.86697248 | 9.532544379 | -0.64118 | 0.040462 | 0.040462 | Downregulated |
| MYH8 | 30.90825688 | 19.99408284 | -0.62842 | 0.024111 | 0.024111 | Downregulated |
| AMER2 | 31.74770642 | 20.5739645 | -0.62583 | 0.047045 | 0.047045 | Downregulated |
| DIO1 | 154.0458716 | 99.94674556 | -0.62413 | 4.32E-07 | 4.32E-07 | Downregulated |
| EPHA8 | 70.00458716 | 45.58579882 | -0.61887 | 0.001355 | 0.001355 | Downregulated |
| AL049844.1 | 23.67431193 | 15.4260355 | -0.61796 | 0.01988 | 0.01988 | Downregulated |
| GPR52 | 28.27522936 | 18.5443787 | -0.60856 | 0.035082 | 0.035082 | Downregulated |
| BTNL2 | 13.89908257 | 9.130177515 | -0.60627 | 0.049302 | 0.049302 | Downregulated |
| PSAPL1 | 50.00917431 | 33.10650888 | -0.59508 | 0.032614 | 0.032614 | Downregulated |
| KRT222 | 69.50917431 | 46.22485207 | -0.58853 | 0.001933 | 0.001933 | Downregulated |
| PAGE4 | 8.472477064 | 5.650887574 | -0.58431 | 0.018487 | 0.018487 | Downregulated |
| STARD6 | 18.92201835 | 12.71005917 | -0.5741 | 0.045579 | 0.045579 | Downregulated |
| RDH8 | 63.55963303 | 42.82248521 | -0.56974 | 0.002393 | 0.002393 | Downregulated |
| CHIA | 6.862385321 | 4.627218935 | -0.56856 | 0.010192 | 0.010192 | Downregulated |
| CENPVL3 | 1.798165138 | 1.213017751 | -0.56792 | 0.027745 | 0.027745 | Downregulated |
| IL21 | 30.88073394 | 20.85798817 | -0.56611 | 0.037369 | 0.037369 | Downregulated |
| FZD10 | 223.1788991 | 151.7988166 | -0.55604 | 4.78E-10 | 4.78E-10 | Downregulated |
| ETNPPL | 14.28899083 | 9.721893491 | -0.55559 | 0.024275 | 0.024275 | Downregulated |
| PITX3 | 42.22477064 | 28.85798817 | -0.54912 | 0.002825 | 0.002825 | Downregulated |
| LIPN | 66.68348624 | 46.12426036 | -0.5318 | 0.005722 | 0.005722 | Downregulated |
| OR2C1 | 50.87614679 | 35.20118343 | -0.53137 | 0.025176 | 0.025176 | Downregulated |
| IQCA1L | 5.752293578 | 3.982248521 | -0.53055 | 0.044826 | 0.044826 | Downregulated |
| MYH6 | 35.83486239 | 24.86982249 | -0.52697 | 0.026002 | 0.026002 | Downregulated |
| ENPP1 | 223.7522936 | 155.6213018 | -0.52386 | 2.81E-09 | 2.81E-09 | Downregulated |
| MC2R | 27.69266055 | 19.29585799 | -0.52121 | 0.048836 | 0.048836 | Downregulated |
| C11orf52 | 123.3577982 | 86.01183432 | -0.52024 | 0.000181 | 0.000181 | Downregulated |
| INSL5 | 55.72477064 | 38.90532544 | -0.51835 | 0.007703 | 0.007703 | Downregulated |
| SLC22A4 | 223.3853211 | 156.0946746 | -0.51711 | 4.39E-09 | 4.39E-09 | Downregulated |
| PATE1 | 1.903669725 | 1.331360947 | -0.51588 | 0.00412 | 0.00412 | Downregulated |
| ITPRIPL1 | 222.8669725 | 156.7633136 | -0.50759 | 8.15E-09 | 8.15E-09 | Downregulated |
| NPY2R | 35.18807339 | 24.80473373 | -0.50447 | 0.017841 | 0.017841 | Downregulated |
| LCLAT1 | 222.5733945 | 157.1420118 | -0.50221 | 1.15E-08 | 1.15E-08 | Downregulated |
| PPM1D | 222.3027523 | 157.4911243 | -0.49726 | 1.58E-08 | 1.58E-08 | Downregulated |
| HSPB3 | 102.3211009 | 72.61538462 | -0.49476 | 0.003428 | 0.003428 | Downregulated |
| OR3A3 | 1.45412844 | 1.035502959 | -0.48982 | 0.022693 | 0.022693 | Downregulated |
| OTOP3 | 16.06422018 | 11.44970414 | -0.48854 | 0.019332 | 0.019332 | Downregulated |
| MERTK | 221.8256881 | 158.1065089 | -0.48853 | 2.74E-08 | 2.74E-08 | Downregulated |
| PATE3 | 1.889908257 | 1.349112426 | -0.48631 | 0.014426 | 0.014426 | Downregulated |
| HACE1 | 221.5825688 | 158.4201183 | -0.48409 | 3.61E-08 | 3.61E-08 | Downregulated |
| FAM19A4 | 30.25229358 | 21.66863905 | -0.48144 | 0.038753 | 0.038753 | Downregulated |
| ZNF101 | 221.4266055 | 158.6213018 | -0.48124 | 4.31E-08 | 4.31E-08 | Downregulated |
| DAZL | 98.16055046 | 70.31952663 | -0.48122 | 0.001736 | 0.001736 | Downregulated |
| CLEC6A | 113.059633 | 81.00591716 | -0.48098 | 0.000459 | 0.000459 | Downregulated |
| ALX1 | 31.89908257 | 22.91715976 | -0.47709 | 0.041546 | 0.041546 | Downregulated |
| RAB39A | 190.4266055 | 136.8934911 | -0.47618 | 2.44E-06 | 2.44E-06 | Downregulated |
| MTDH | 221.0366972 | 159.1242604 | -0.47413 | 6.67E-08 | 6.67E-08 | Downregulated |
| NANP | 220.9770642 | 159.2011834 | -0.47305 | 7.13E-08 | 7.13E-08 | Downregulated |
| RNFT1 | 220.8348624 | 159.3846154 | -0.47046 | 8.35E-08 | 8.35E-08 | Downregulated |
| FADS6 | 126.8853211 | 91.73964497 | -0.46791 | 0.00075 | 0.00075 | Downregulated |
| ZNF844 | 220.3073394 | 160.0650888 | -0.46086 | 1.49E-07 | 1.49E-07 | Downregulated |
| ZNF492 | 113.3027523 | 82.32544379 | -0.46077 | 0.003029 | 0.003029 | Downregulated |
| SMIM18 | 73.04587156 | 53.21893491 | -0.45686 | 0.005325 | 0.005325 | Downregulated |
| ZNF304 | 220.0275229 | 160.4260355 | -0.45578 | 2.01E-07 | 2.01E-07 | Downregulated |
| IL20 | 58.83944954 | 42.96449704 | -0.45364 | 0.019289 | 0.019289 | Downregulated |
| RFX2 | 219.8073394 | 160.7100592 | -0.45178 | 2.55E-07 | 2.55E-07 | Downregulated |
| KLHL7 | 219.7844037 | 160.739645 | -0.45136 | 2.61E-07 | 2.61E-07 | Downregulated |
| ZDHHC19 | 144.0366972 | 105.4142012 | -0.45037 | 0.000423 | 0.000423 | Downregulated |
| LRRC14B | 54.78440367 | 40.1183432 | -0.4495 | 0.047186 | 0.047186 | Downregulated |
| ADAM22 | 219.646789 | 160.9171598 | -0.44887 | 3.02E-07 | 3.02E-07 | Downregulated |
| HRASLS | 110.5458716 | 81 | -0.44865 | 0.00115 | 0.00115 | Downregulated |
| NMT2 | 219.5137615 | 161.0887574 | -0.44646 | 3.48E-07 | 3.48E-07 | Downregulated |
| ZNF639 | 219.4541284 | 161.1656805 | -0.44537 | 3.70E-07 | 3.70E-07 | Downregulated |
| HS3ST4 | 84.2293578 | 61.92899408 | -0.44371 | 0.00863 | 0.00863 | Downregulated |
| DCUN1D4 | 219.2247706 | 161.4615385 | -0.44122 | 4.71E-07 | 4.71E-07 | Downregulated |
| TMEM236 | 219.087156 | 161.6390533 | -0.43873 | 5.43E-07 | 5.43E-07 | Downregulated |
| CDIP1 | 219.0550459 | 161.6804734 | -0.43815 | 5.62E-07 | 5.62E-07 | Downregulated |
| PCGF6 | 218.8669725 | 161.9230769 | -0.43475 | 6.82E-07 | 6.82E-07 | Downregulated |
| FAM216A | 218.8119266 | 161.9940828 | -0.43375 | 7.22E-07 | 7.22E-07 | Downregulated |
| SLC28A1 | 124.0550459 | 91.9408284 | -0.4322 | 0.001825 | 0.001825 | Downregulated |
| HIST1H2AI | 193.5504587 | 143.5443787 | -0.43121 | 1.04E-05 | 1.04E-05 | Downregulated |
| FAM180B | 55.65137615 | 41.27810651 | -0.43104 | 0.033693 | 0.033693 | Downregulated |
| KLRG2 | 154.440367 | 114.6094675 | -0.43032 | 0.00065 | 0.00065 | Downregulated |
| ACHE | 218.6146789 | 162.2485207 | -0.43018 | 8.83E-07 | 8.83E-07 | Downregulated |
| TBC1D31 | 218.5917431 | 162.2781065 | -0.42977 | 9.04E-07 | 9.04E-07 | Downregulated |
| VNN2 | 217.4678899 | 161.443787 | -0.42977 | 1.03E-06 | 1.03E-06 | Downregulated |
| DDHD2 | 218.4587156 | 162.4497041 | -0.42737 | 1.03E-06 | 1.03E-06 | Downregulated |
| CLEC4E | 212.7614679 | 158.4378698 | -0.42532 | 2.15E-06 | 2.15E-06 | Downregulated |
| GATA5 | 98.26605505 | 73.23076923 | -0.42424 | 0.002354 | 0.002354 | Downregulated |
| PPT1 | 218.2706422 | 162.6923077 | -0.42397 | 1.25E-06 | 1.25E-06 | Downregulated |
| WNT8B | 184.8119266 | 137.7988166 | -0.42349 | 3.01E-05 | 3.01E-05 | Downregulated |
| IL12A | 209.3073394 | 156.147929 | -0.42271 | 3.36E-06 | 3.36E-06 | Downregulated |
| TMEM74B | 218.1192661 | 162.887574 | -0.42124 | 1.46E-06 | 1.46E-06 | Downregulated |
| C15orf53 | 94.44495413 | 70.58579882 | -0.4201 | 0.013646 | 0.013646 | Downregulated |
| RD3L | 1.472477064 | 1.100591716 | -0.41997 | 0.016027 | 0.016027 | Downregulated |
| CYP26C1 | 73 | 54.59171598 | -0.41921 | 0.032651 | 0.032651 | Downregulated |
| RBL1 | 218.0045872 | 163.035503 | -0.41917 | 1.63E-06 | 1.63E-06 | Downregulated |
| EDEM2 | 217.5275229 | 162.6982249 | -0.419 | 1.55E-06 | 1.55E-06 | Downregulated |
| FAM218A | 122.7752294 | 91.87573964 | -0.41826 | 0.001287 | 0.001287 | Downregulated |
| CCDC117 | 217.9541284 | 163.1005917 | -0.41826 | 1.72E-06 | 1.72E-06 | Downregulated |
| THOC5 | 217.8807339 | 163.1952663 | -0.41694 | 1.85E-06 | 1.85E-06 | Downregulated |
| HELQ | 217.7798165 | 163.3254438 | -0.41512 | 2.04E-06 | 2.04E-06 | Downregulated |
| CLDN8 | 69.09174312 | 51.84023669 | -0.41444 | 0.015737 | 0.015737 | Downregulated |
| CTDSPL2 | 217.7018349 | 163.4260355 | -0.41372 | 2.20E-06 | 2.20E-06 | Downregulated |
| DDX27 | 217.6880734 | 163.443787 | -0.41347 | 2.23E-06 | 2.23E-06 | Downregulated |
| RBKS | 217.6743119 | 163.4615385 | -0.41322 | 2.27E-06 | 2.27E-06 | Downregulated |
| AP000781.2 | 84.9587156 | 63.82248521 | -0.4127 | 0.006768 | 0.006768 | Downregulated |
| PIGU | 217.6284404 | 163.5207101 | -0.41239 | 2.37E-06 | 2.37E-06 | Downregulated |
| TBCEL | 217.6284404 | 163.5207101 | -0.41239 | 2.37E-06 | 2.37E-06 | Downregulated |
| CNBD2 | 201.1376147 | 151.1538462 | -0.41217 | 1.19E-05 | 1.19E-05 | Downregulated |
| GABRR1 | 87.75688073 | 65.95266272 | -0.41208 | 0.024121 | 0.024121 | Downregulated |
| SRSF12 | 183.1743119 | 137.8106509 | -0.41053 | 5.70E-05 | 5.70E-05 | Downregulated |
| ARL13B | 217.3899083 | 163.8284024 | -0.4081 | 2.99E-06 | 2.99E-06 | Downregulated |
| FBXL20 | 217.3853211 | 163.8343195 | -0.40802 | 3.01E-06 | 3.01E-06 | Downregulated |
| ZNF625 | 94.13761468 | 70.98224852 | -0.40731 | 0.006629 | 0.006629 | Downregulated |
| SLC12A5 | 213.9266055 | 161.4615385 | -0.40593 | 4.71E-06 | 4.71E-06 | Downregulated |
| KCNS2 | 106.1743119 | 80.21301775 | -0.40453 | 0.004156 | 0.004156 | Downregulated |
| FADS3 | 217.1376147 | 164.1538462 | -0.40356 | 3.82E-06 | 3.82E-06 | Downregulated |
| CLEC1B | 65.18807339 | 49.29585799 | -0.40314 | 0.022604 | 0.022604 | Downregulated |
| RAI14 | 217.0504587 | 164.2662722 | -0.40199 | 4.15E-06 | 4.15E-06 | Downregulated |
| SMTNL2 | 213.7110092 | 161.739645 | -0.40199 | 5.81E-06 | 5.81E-06 | Downregulated |
| CLEC4D | 132.4449541 | 100.3846154 | -0.39985 | 0.00128 | 0.00128 | Downregulated |
| HIST2H3A | 1.724770642 | 1.307692308 | -0.39938 | 0.030567 | 0.030567 | Downregulated |
| ZNF543 | 216.793578 | 164.5976331 | -0.39738 | 5.31E-06 | 5.31E-06 | Downregulated |
| EEF2K | 216.7706422 | 164.6272189 | -0.39697 | 5.42E-06 | 5.42E-06 | Downregulated |
| WDR17 | 154.0642202 | 117.0177515 | -0.3968 | 0.000665 | 0.000665 | Downregulated |
| DBNDD2 | 216.7155963 | 164.6982249 | -0.39598 | 5.71E-06 | 5.71E-06 | Downregulated |
| PLCL1 | 216.6697248 | 164.7573964 | -0.39515 | 5.97E-06 | 5.97E-06 | Downregulated |
| WASF3 | 216.6513761 | 164.7810651 | -0.39482 | 6.07E-06 | 6.07E-06 | Downregulated |
| RWDD2A | 216.6513761 | 164.7810651 | -0.39482 | 6.07E-06 | 6.07E-06 | Downregulated |
| POFUT1 | 216.6330275 | 164.8047337 | -0.3945 | 6.18E-06 | 6.18E-06 | Downregulated |
| VNN3 | 189.646789 | 144.2899408 | -0.39434 | 5.52E-05 | 5.52E-05 | Downregulated |
| AC024270.1 | 216.6146789 | 164.8284024 | -0.39417 | 6.29E-06 | 6.29E-06 | Downregulated |
| C4orf50 | 85.94495413 | 65.40828402 | -0.39394 | 0.04323 | 0.04323 | Downregulated |
| TMOD2 | 216.5366972 | 164.9289941 | -0.39277 | 6.76E-06 | 6.76E-06 | Downregulated |
| PCSK1N | 197.9862385 | 150.8343195 | -0.39244 | 3.52E-05 | 3.52E-05 | Downregulated |
| TSPAN2 | 216.5091743 | 164.964497 | -0.39227 | 6.94E-06 | 6.94E-06 | Downregulated |
| SSTR2 | 216.4816514 | 165 | -0.39178 | 7.12E-06 | 7.12E-06 | Downregulated |
| MAFA | 129.5412844 | 98.80473373 | -0.39076 | 0.003222 | 0.003222 | Downregulated |
| ZYG11A | 132.9724771 | 101.4911243 | -0.38977 | 0.003188 | 0.003188 | Downregulated |
| RIMS2 | 104.1513761 | 79.64497041 | -0.38703 | 0.009594 | 0.009594 | Downregulated |
| RNF26 | 216.1880734 | 165.3786982 | -0.38651 | 9.37E-06 | 9.37E-06 | Downregulated |
| KDELC1 | 216.1697248 | 165.4023669 | -0.38618 | 9.53E-06 | 9.53E-06 | Downregulated |
| UPB1 | 188.2018349 | 144.0177515 | -0.38603 | 9.93E-05 | 9.93E-05 | Downregulated |
| CCNE2 | 216.059633 | 165.5443787 | -0.38421 | 1.05E-05 | 1.05E-05 | Downregulated |
| ZNF549 | 216.0366972 | 165.5739645 | -0.3838 | 1.08E-05 | 1.08E-05 | Downregulated |
| TPD52L2 | 216.0366972 | 165.5739645 | -0.3838 | 1.08E-05 | 1.08E-05 | Downregulated |
| KBTBD6 | 216.0275229 | 165.5857988 | -0.38364 | 1.09E-05 | 1.09E-05 | Downregulated |
| TUBD1 | 216.0137615 | 165.6035503 | -0.38339 | 1.10E-05 | 1.10E-05 | Downregulated |
| ZNF572 | 215.9816514 | 165.6449704 | -0.38281 | 1.13E-05 | 1.13E-05 | Downregulated |
| PNLIPRP1 | 150.646789 | 115.6745562 | -0.3811 | 0.001409 | 0.001409 | Downregulated |
| CNEP1R1 | 215.8715596 | 165.7869822 | -0.38084 | 1.25E-05 | 1.25E-05 | Downregulated |
| GDF10 | 151.5412844 | 116.4319527 | -0.38022 | 0.001442 | 0.001442 | Downregulated |
| RAD21 | 215.8256881 | 165.8461538 | -0.38002 | 1.31E-05 | 1.31E-05 | Downregulated |
| GID8 | 215.8211009 | 165.852071 | -0.37994 | 1.31E-05 | 1.31E-05 | Downregulated |
| ELOVL3 | 192.0642202 | 147.6153846 | -0.37975 | 0.000106 | 0.000106 | Downregulated |
| TMEM67 | 215.8073394 | 165.8698225 | -0.37969 | 1.33E-05 | 1.33E-05 | Downregulated |
| POLR2B | 214.9036697 | 165.1775148 | -0.37967 | 1.42E-05 | 1.42E-05 | Downregulated |
| ROS1 | 154.3027523 | 118.6390533 | -0.37918 | 0.000903 | 0.000903 | Downregulated |
| WRB | 215.7706422 | 165.9171598 | -0.37904 | 1.37E-05 | 1.37E-05 | Downregulated |
| VSTM1 | 108.6009174 | 83.50887574 | -0.37903 | 0.005915 | 0.005915 | Downregulated |
| TRIM37 | 215.7477064 | 165.9467456 | -0.37862 | 1.40E-05 | 1.40E-05 | Downregulated |
| SNIP1 | 215.733945 | 165.964497 | -0.37838 | 1.42E-05 | 1.42E-05 | Downregulated |
| KCNT1 | 196.1834862 | 150.9763314 | -0.37788 | 8.13E-05 | 8.13E-05 | Downregulated |
| FBXO3 | 215.6880734 | 166.0236686 | -0.37756 | 1.48E-05 | 1.48E-05 | Downregulated |
| LRRK2 | 212.3302752 | 163.5207101 | -0.37684 | 2.07E-05 | 2.07E-05 | Downregulated |
| HOOK3 | 215.5963303 | 166.1420118 | -0.37592 | 1.61E-05 | 1.61E-05 | Downregulated |
| CYP11B2 | 1.550458716 | 1.195266272 | -0.37536 | 0.028378 | 0.028378 | Downregulated |
| APPBP2 | 215.5550459 | 166.1952663 | -0.37518 | 1.67E-05 | 1.67E-05 | Downregulated |
| MFSD11 | 215.5458716 | 166.2071006 | -0.37501 | 1.68E-05 | 1.68E-05 | Downregulated |
| HYDIN | 118.8807339 | 91.78698225 | -0.37315 | 0.011808 | 0.011808 | Downregulated |
| CTNNBL1 | 215.412844 | 166.3786982 | -0.37263 | 1.90E-05 | 1.90E-05 | Downregulated |
| GBP6 | 121.3348624 | 93.73372781 | -0.37235 | 0.010159 | 0.010159 | Downregulated |
| TSPAN6 | 215.3899083 | 166.408284 | -0.37222 | 1.94E-05 | 1.94E-05 | Downregulated |
| OIT3 | 212.059633 | 163.8698225 | -0.37192 | 2.65E-05 | 2.65E-05 | Downregulated |
| TMEM82 | 210.9587156 | 163.0236686 | -0.37188 | 2.89E-05 | 2.89E-05 | Downregulated |
| DIRC2 | 215.3669725 | 166.4378698 | -0.37181 | 1.98E-05 | 1.98E-05 | Downregulated |
| GPR82 | 209.853211 | 162.1893491 | -0.3717 | 3.18E-05 | 3.18E-05 | Downregulated |
| KCNA2 | 145.6834862 | 112.6094675 | -0.37151 | 0.001757 | 0.001757 | Downregulated |
| EIF5 | 215.3073394 | 166.5147929 | -0.37075 | 2.09E-05 | 2.09E-05 | Downregulated |
| POTEE | 108.3577982 | 83.82248521 | -0.37039 | 0.033468 | 0.033468 | Downregulated |
| CLEC1A | 214.1330275 | 165.7455621 | -0.36954 | 2.44E-05 | 2.44E-05 | Downregulated |
| AIRE | 170.2293578 | 131.7928994 | -0.36921 | 0.000451 | 0.000451 | Downregulated |
| ZNF555 | 215.1880734 | 166.6686391 | -0.36862 | 2.32E-05 | 2.32E-05 | Downregulated |
| ZNF426 | 215.1834862 | 166.6745562 | -0.36853 | 2.33E-05 | 2.33E-05 | Downregulated |
| CHST15 | 215.1743119 | 166.6863905 | -0.36837 | 2.35E-05 | 2.35E-05 | Downregulated |
| CFL2 | 215.1697248 | 166.6923077 | -0.36829 | 2.36E-05 | 2.36E-05 | Downregulated |
| HOXC11 | 122.8715596 | 95.18934911 | -0.36828 | 0.021191 | 0.021191 | Downregulated |
| ZNF43 | 215.1376147 | 166.7337278 | -0.36771 | 2.43E-05 | 2.43E-05 | Downregulated |
| SCAMP5 | 215.1146789 | 166.7633136 | -0.3673 | 2.48E-05 | 2.48E-05 | Downregulated |
| CYP20A1 | 215.0917431 | 166.7928994 | -0.36689 | 2.53E-05 | 2.53E-05 | Downregulated |
| NDUFAF6 | 215.0642202 | 166.8284024 | -0.3664 | 2.59E-05 | 2.59E-05 | Downregulated |
| PNMA5 | 74.56422018 | 57.88757396 | -0.36523 | 0.043516 | 0.043516 | Downregulated |
| FOXI2 | 87.38990826 | 67.87573964 | -0.36457 | 0.037906 | 0.037906 | Downregulated |
| SEC22C | 214.9541284 | 166.9704142 | -0.36444 | 2.85E-05 | 2.85E-05 | Downregulated |
| MRPL49 | 214.9220183 | 167.0118343 | -0.36386 | 2.93E-05 | 2.93E-05 | Downregulated |
| ZNF2 | 214.3073394 | 166.5384615 | -0.36383 | 2.86E-05 | 2.86E-05 | Downregulated |
| AC106782.1 | 130.3394495 | 101.3195266 | -0.36336 | 0.005381 | 0.005381 | Downregulated |
| KIAA1586 | 214.8486239 | 167.1065089 | -0.36255 | 3.13E-05 | 3.13E-05 | Downregulated |
| SNURF | 126.0275229 | 98.06508876 | -0.36193 | 0.01614 | 0.01614 | Downregulated |
| SLC6A4 | 204.9541284 | 159.5266272 | -0.3615 | 7.35E-05 | 7.35E-05 | Downregulated |
| ACVR2A | 214.7706422 | 167.2071006 | -0.36116 | 3.35E-05 | 3.35E-05 | Downregulated |
| STARD7 | 214.7614679 | 167.2189349 | -0.361 | 3.38E-05 | 3.38E-05 | Downregulated |
| ATG14 | 214.7614679 | 167.2189349 | -0.361 | 3.38E-05 | 3.38E-05 | Downregulated |
| ST8SIA4 | 214.7385321 | 167.2485207 | -0.36059 | 3.45E-05 | 3.45E-05 | Downregulated |
| SLCO3A1 | 214.7110092 | 167.2840237 | -0.3601 | 3.53E-05 | 3.53E-05 | Downregulated |
| ZKSCAN2 | 214.6972477 | 167.3017751 | -0.35985 | 3.57E-05 | 3.57E-05 | Downregulated |
| ETAA1 | 214.6834862 | 167.3195266 | -0.35961 | 3.61E-05 | 3.61E-05 | Downregulated |
| SAMSN1 | 214.6743119 | 167.3313609 | -0.35944 | 3.64E-05 | 3.64E-05 | Downregulated |
| YTHDF1 | 214.6697248 | 167.3372781 | -0.35936 | 3.66E-05 | 3.66E-05 | Downregulated |
| TMEM133 | 214.6651376 | 167.3431953 | -0.35928 | 3.67E-05 | 3.67E-05 | Downregulated |
| RAD1 | 214.6422018 | 167.3727811 | -0.35887 | 3.75E-05 | 3.75E-05 | Downregulated |
| ABHD12 | 214.6055046 | 167.4201183 | -0.35821 | 3.87E-05 | 3.87E-05 | Downregulated |
| MME | 214.6055046 | 167.4201183 | -0.35821 | 3.87E-05 | 3.87E-05 | Downregulated |
| CNNM2 | 214.5366972 | 167.3964497 | -0.35796 | 3.86E-05 | 3.86E-05 | Downregulated |
| ARL11 | 214.587156 | 167.443787 | -0.35789 | 3.93E-05 | 3.93E-05 | Downregulated |
| IL34 | 214.587156 | 167.443787 | -0.35789 | 3.93E-05 | 3.93E-05 | Downregulated |
| PAPLN | 214.5688073 | 167.4674556 | -0.35756 | 3.99E-05 | 3.99E-05 | Downregulated |
| ZNF439 | 214.5321101 | 167.5147929 | -0.35691 | 4.12E-05 | 4.12E-05 | Downregulated |
| PAPOLG | 214.4908257 | 167.5680473 | -0.35617 | 4.27E-05 | 4.27E-05 | Downregulated |
| ZNF681 | 214.4587156 | 167.6094675 | -0.3556 | 4.39E-05 | 4.39E-05 | Downregulated |
| SNX21 | 214.4266055 | 167.6508876 | -0.35502 | 4.51E-05 | 4.51E-05 | Downregulated |
| SLC35G2 | 214.4220183 | 167.6568047 | -0.35494 | 4.53E-05 | 4.53E-05 | Downregulated |
| MPP5 | 214.412844 | 167.6686391 | -0.35478 | 4.57E-05 | 4.57E-05 | Downregulated |
| SGK3 | 214.3761468 | 167.7159763 | -0.35412 | 4.71E-05 | 4.71E-05 | Downregulated |
| MTBP | 214.3486239 | 167.7514793 | -0.35363 | 4.83E-05 | 4.83E-05 | Downregulated |
| GNG2 | 214.3256881 | 167.7810651 | -0.35322 | 4.92E-05 | 4.92E-05 | Downregulated |
| DENND6A | 214.3256881 | 167.7810651 | -0.35322 | 4.92E-05 | 4.92E-05 | Downregulated |
| ACTR3C | 214.2568807 | 167.8698225 | -0.352 | 5.22E-05 | 5.22E-05 | Downregulated |
| MDH1B | 204.4357798 | 160.1952663 | -0.35182 | 0.000111 | 0.000111 | Downregulated |
| NEU1 | 214.2293578 | 167.9053254 | -0.35151 | 5.34E-05 | 5.34E-05 | Downregulated |
| IL24 | 214.2110092 | 167.9289941 | -0.35118 | 5.43E-05 | 5.43E-05 | Downregulated |
| ZNF257 | 188.5642202 | 147.8284024 | -0.35113 | 0.00043 | 0.00043 | Downregulated |
| CD38 | 214.1926606 | 167.9526627 | -0.35085 | 5.51E-05 | 5.51E-05 | Downregulated |
| NUDT21 | 214.1834862 | 167.964497 | -0.35069 | 5.56E-05 | 5.56E-05 | Downregulated |
| FMO1 | 210.8853211 | 165.3846154 | -0.35063 | 7.23E-05 | 7.23E-05 | Downregulated |
| QKI | 214.1743119 | 167.9763314 | -0.35053 | 5.60E-05 | 5.60E-05 | Downregulated |
| DENND5B | 214.146789 | 168.0118343 | -0.35004 | 5.73E-05 | 5.73E-05 | Downregulated |
| TMIGD1 | 130.7293578 | 102.5976331 | -0.34959 | 0.003557 | 0.003557 | Downregulated |
| THAP9 | 214.1009174 | 168.0710059 | -0.34922 | 5.96E-05 | 5.96E-05 | Downregulated |
| 3-Sep | 214.0963303 | 168.0769231 | -0.34914 | 5.98E-05 | 5.98E-05 | Downregulated |
| NELFCD | 214.087156 | 168.0887574 | -0.34898 | 6.03E-05 | 6.03E-05 | Downregulated |
| HACD1 | 214.0366972 | 168.1538462 | -0.34808 | 6.29E-05 | 6.29E-05 | Downregulated |
| PHACTR2 | 214.0366972 | 168.1538462 | -0.34808 | 6.29E-05 | 6.29E-05 | Downregulated |
| ARMC8 | 214.0137615 | 168.183432 | -0.34767 | 6.41E-05 | 6.41E-05 | Downregulated |
| TMEM65 | 214.0091743 | 168.1893491 | -0.34759 | 6.44E-05 | 6.44E-05 | Downregulated |
| BMF | 214.0091743 | 168.1893491 | -0.34759 | 6.44E-05 | 6.44E-05 | Downregulated |
| SLITRK4 | 163.4449541 | 128.4556213 | -0.34753 | 0.001407 | 0.001407 | Downregulated |
| CAMKV | 184.2752294 | 144.8284024 | -0.34752 | 0.000598 | 0.000598 | Downregulated |
| ARHGAP42 | 213.9816514 | 168.2248521 | -0.3471 | 6.59E-05 | 6.59E-05 | Downregulated |
| VCPIP1 | 213.9541284 | 168.260355 | -0.34661 | 6.74E-05 | 6.74E-05 | Downregulated |
| HSD17B1 | 213.9449541 | 168.2721893 | -0.34644 | 6.80E-05 | 6.80E-05 | Downregulated |
| TKTL1 | 103.8027523 | 81.68047337 | -0.34578 | 0.016461 | 0.016461 | Downregulated |
| ADAM20 | 86.94954128 | 68.44378698 | -0.34526 | 0.047072 | 0.047072 | Downregulated |
| ING3 | 213.8669725 | 168.3727811 | -0.34505 | 7.26E-05 | 7.26E-05 | Downregulated |
| ATP6V1C2 | 213.7706422 | 168.4970414 | -0.34334 | 7.86E-05 | 7.86E-05 | Downregulated |
| NDUFAF7 | 213.766055 | 168.5029586 | -0.34326 | 7.90E-05 | 7.90E-05 | Downregulated |
| AGO1 | 213.7522936 | 168.5207101 | -0.34301 | 7.99E-05 | 7.99E-05 | Downregulated |
| RAD51B | 213.7155963 | 168.5680473 | -0.34236 | 8.23E-05 | 8.23E-05 | Downregulated |
| ZNF14 | 213.706422 | 168.5798817 | -0.3422 | 8.30E-05 | 8.30E-05 | Downregulated |
| AQP12A | 126.206422 | 99.58579882 | -0.34177 | 0.011952 | 0.011952 | Downregulated |
| PPP1CB | 212.9174312 | 168.035503 | -0.34153 | 8.67E-05 | 8.67E-05 | Downregulated |
| CCDC169 | 109.8853211 | 86.73372781 | -0.34133 | 0.0175 | 0.0175 | Downregulated |
| GPATCH11 | 213.6559633 | 168.6449704 | -0.3413 | 8.65E-05 | 8.65E-05 | Downregulated |
| MPP6 | 213.6513761 | 168.6508876 | -0.34122 | 8.69E-05 | 8.69E-05 | Downregulated |
| LGI4 | 213.6422018 | 168.6627219 | -0.34106 | 8.75E-05 | 8.75E-05 | Downregulated |
| INTS2 | 213.0825688 | 168.2248521 | -0.34102 | 8.51E-05 | 8.51E-05 | Downregulated |
| ZBTB8B | 91.81192661 | 72.4852071 | -0.34099 | 0.025946 | 0.025946 | Downregulated |
| NABP1 | 213.6376147 | 168.6686391 | -0.34097 | 8.78E-05 | 8.78E-05 | Downregulated |
| THAP1 | 213.6100917 | 168.704142 | -0.34048 | 8.99E-05 | 8.99E-05 | Downregulated |
| F2RL2 | 213.6100917 | 168.704142 | -0.34048 | 8.99E-05 | 8.99E-05 | Downregulated |
| INPP5B | 213.2568807 | 168.4378698 | -0.34038 | 8.69E-05 | 8.69E-05 | Downregulated |
| CAPN11 | 204.8944954 | 161.8402367 | -0.34031 | 0.000173 | 0.000173 | Downregulated |
| UBE2W | 213.5963303 | 168.7218935 | -0.34024 | 9.09E-05 | 9.09E-05 | Downregulated |
| KCNIP1 | 105.9541284 | 83.69822485 | -0.34017 | 0.036382 | 0.036382 | Downregulated |
| USP42 | 213.5917431 | 168.7278107 | -0.34016 | 9.13E-05 | 9.13E-05 | Downregulated |
| DDHD1 | 213.5504587 | 168.7810651 | -0.33942 | 9.44E-05 | 9.44E-05 | Downregulated |
| SERINC1 | 213.5458716 | 168.7869822 | -0.33934 | 9.48E-05 | 9.48E-05 | Downregulated |
| PCIF1 | 213.5366972 | 168.7988166 | -0.33918 | 9.55E-05 | 9.55E-05 | Downregulated |
| CALCRL | 213.5321101 | 168.8047337 | -0.3391 | 9.59E-05 | 9.59E-05 | Downregulated |
| WDCP | 212.7889908 | 168.3076923 | -0.33832 | 9.79E-05 | 9.79E-05 | Downregulated |
| GDPD4 | 139.9357798 | 110.704142 | -0.33806 | 0.018135 | 0.018135 | Downregulated |
| CCDC82 | 213.4724771 | 168.8816568 | -0.33804 | 0.000101 | 0.000101 | Downregulated |
| ADAM17 | 213.4724771 | 168.8816568 | -0.33804 | 0.000101 | 0.000101 | Downregulated |
| ZNF699 | 212.3394495 | 168.0591716 | -0.3374 | 0.000113 | 0.000113 | Downregulated |
| BMP8B | 213.3944954 | 168.9822485 | -0.33665 | 0.000107 | 0.000107 | Downregulated |
| SMOC1 | 213.3944954 | 168.9822485 | -0.33665 | 0.000107 | 0.000107 | Downregulated |
| TUSC3 | 213.3715596 | 169.0118343 | -0.33624 | 0.000109 | 0.000109 | Downregulated |
| SRSF6 | 213.3669725 | 169.0177515 | -0.33616 | 0.00011 | 0.00011 | Downregulated |
| ZNF547 | 211.146789 | 167.3195266 | -0.33564 | 0.000132 | 0.000132 | Downregulated |
| ZNF813 | 213.3302752 | 169.0650888 | -0.33551 | 0.000113 | 0.000113 | Downregulated |
| CCDC163 | 213.3211009 | 169.0769231 | -0.33535 | 0.000114 | 0.000114 | Downregulated |
| NCBP2L | 2.02293578 | 1.603550296 | -0.33518 | 0.046222 | 0.046222 | Downregulated |
| CCNYL1 | 213.2614679 | 169.1538462 | -0.33429 | 0.00012 | 0.00012 | Downregulated |
| KCNAB2 | 213.1697248 | 169.2721893 | -0.33266 | 0.000129 | 0.000129 | Downregulated |
| GRIN2C | 212.0688073 | 168.408284 | -0.33257 | 0.00014 | 0.00014 | Downregulated |
| SLC35E3 | 213.1330275 | 169.3195266 | -0.33201 | 0.000133 | 0.000133 | Downregulated |
| XRN1 | 213.1192661 | 169.3372781 | -0.33176 | 0.000134 | 0.000134 | Downregulated |
| TMEM151A | 195.9082569 | 155.704142 | -0.33137 | 0.000447 | 0.000447 | Downregulated |
| CYP7B1 | 210.8990826 | 167.6390533 | -0.33119 | 0.000161 | 0.000161 | Downregulated |
| RBMS1 | 213.087156 | 169.3786982 | -0.33119 | 0.000138 | 0.000138 | Downregulated |
| ERLEC1 | 213.0825688 | 169.3846154 | -0.33111 | 0.000138 | 0.000138 | Downregulated |
| TFEC | 213.0825688 | 169.3846154 | -0.33111 | 0.000138 | 0.000138 | Downregulated |
| PSMD11 | 213.0779817 | 169.3905325 | -0.33103 | 0.000139 | 0.000139 | Downregulated |
| HTRA3 | 213.0779817 | 169.3905325 | -0.33103 | 0.000139 | 0.000139 | Downregulated |
| TPP1 | 213.0733945 | 169.3964497 | -0.33095 | 0.000139 | 0.000139 | Downregulated |
| SCARB2 | 213.0550459 | 169.4201183 | -0.33062 | 0.000141 | 0.000141 | Downregulated |
| APELA | 191.6605505 | 152.4615385 | -0.33011 | 0.000571 | 0.000571 | Downregulated |
| HIST1H2AD | 203.2706422 | 161.6982249 | -0.3301 | 0.000281 | 0.000281 | Downregulated |
| S100A12 | 159.8348624 | 127.147929 | -0.33007 | 0.002546 | 0.002546 | Downregulated |
| ZNF670 | 213.0229358 | 169.4615385 | -0.33005 | 0.000145 | 0.000145 | Downregulated |
| OTOP2 | 80.44495413 | 64 | -0.32993 | 0.033971 | 0.033971 | Downregulated |
| GPR34 | 213.0045872 | 169.4852071 | -0.32973 | 0.000147 | 0.000147 | Downregulated |
| CYSLTR1 | 213 | 169.4911243 | -0.32964 | 0.000148 | 0.000148 | Downregulated |
| RASSF8 | 212.9862385 | 169.5088757 | -0.3294 | 0.00015 | 0.00015 | Downregulated |
| SHISA9 | 139.6146789 | 111.1183432 | -0.32935 | 0.01015 | 0.01015 | Downregulated |
| AOX1 | 208.6055046 | 166.0591716 | -0.32908 | 0.000206 | 0.000206 | Downregulated |
| EFHC1 | 212.9678899 | 169.5325444 | -0.32907 | 0.000152 | 0.000152 | Downregulated |
| PRDM13 | 98.08256881 | 78.08284024 | -0.32899 | 0.03893 | 0.03893 | Downregulated |
| BCL11B | 212.9357798 | 169.5739645 | -0.3285 | 0.000156 | 0.000156 | Downregulated |
| GTF2E1 | 212.9311927 | 169.5798817 | -0.32842 | 0.000156 | 0.000156 | Downregulated |
| ZNF724 | 212.9266055 | 169.5857988 | -0.32834 | 0.000157 | 0.000157 | Downregulated |
| GLCCI1 | 212.9266055 | 169.5857988 | -0.32834 | 0.000157 | 0.000157 | Downregulated |
| NAPB | 212.9220183 | 169.591716 | -0.32826 | 0.000157 | 0.000157 | Downregulated |
| TIFA | 212.9174312 | 169.5976331 | -0.32818 | 0.000158 | 0.000158 | Downregulated |
| SMG8 | 212.8944954 | 169.6272189 | -0.32777 | 0.000161 | 0.000161 | Downregulated |
| METTL6 | 212.8807339 | 169.6449704 | -0.32753 | 0.000163 | 0.000163 | Downregulated |
| NFATC2 | 212.8302752 | 169.7100592 | -0.32663 | 0.000169 | 0.000169 | Downregulated |
| RERGL | 86.51834862 | 69 | -0.32641 | 0.030593 | 0.030593 | Downregulated |
| OR2B6 | 126.5321101 | 100.9230769 | -0.32625 | 0.011661 | 0.011661 | Downregulated |
| DDX19B | 212.7981651 | 169.7514793 | -0.32606 | 0.000174 | 0.000174 | Downregulated |
| CHUK | 212.793578 | 169.7573964 | -0.32598 | 0.000175 | 0.000175 | Downregulated |
| GINS4 | 212.7844037 | 169.7692308 | -0.32582 | 0.000176 | 0.000176 | Downregulated |
| NOL9 | 212.7706422 | 169.7869822 | -0.32557 | 0.000178 | 0.000178 | Downregulated |
| C17orf80 | 212.7614679 | 169.7988166 | -0.32541 | 0.000179 | 0.000179 | Downregulated |
| PDRG1 | 212.7522936 | 169.8106509 | -0.32525 | 0.00018 | 0.00018 | Downregulated |
| ZBED9 | 152.1376147 | 121.4319527 | -0.32523 | 0.00426 | 0.00426 | Downregulated |
| ABCD2 | 196.6330275 | 156.964497 | -0.32507 | 0.000555 | 0.000555 | Downregulated |
| KCNF1 | 189.3302752 | 151.1420118 | -0.325 | 0.000867 | 0.000867 | Downregulated |
| CYSLTR2 | 204.059633 | 162.9171598 | -0.32485 | 0.000328 | 0.000328 | Downregulated |
| GYPE | 135.0504587 | 107.8343195 | -0.32468 | 0.009202 | 0.009202 | Downregulated |
| HIST1H3D | 212.7201835 | 169.852071 | -0.32468 | 0.000185 | 0.000185 | Downregulated |
| HILPDA | 212.7155963 | 169.8579882 | -0.3246 | 0.000186 | 0.000186 | Downregulated |
| P2RY13 | 212.7155963 | 169.8579882 | -0.3246 | 0.000186 | 0.000186 | Downregulated |
| GPR88 | 119.0045872 | 95.0295858 | -0.32457 | 0.013889 | 0.013889 | Downregulated |
| SOGA3 | 153.0366972 | 122.2071006 | -0.32455 | 0.0061 | 0.0061 | Downregulated |
| ZNF689 | 212.6972477 | 169.8816568 | -0.32427 | 0.000188 | 0.000188 | Downregulated |
| TRPC4AP | 212.6743119 | 169.9112426 | -0.32386 | 0.000192 | 0.000192 | Downregulated |
| CA4 | 194.4678899 | 155.3727811 | -0.3238 | 0.000577 | 0.000577 | Downregulated |
| RIT1 | 212.6651376 | 169.9230769 | -0.3237 | 0.000193 | 0.000193 | Downregulated |
| ATP6V1E1 | 212.6513761 | 169.9408284 | -0.32346 | 0.000195 | 0.000195 | Downregulated |
| PIPOX | 212.6055046 | 170 | -0.32264 | 0.000203 | 0.000203 | Downregulated |
| AGO4 | 212.6055046 | 170 | -0.32264 | 0.000203 | 0.000203 | Downregulated |
| TRAM1 | 212.6055046 | 170 | -0.32264 | 0.000203 | 0.000203 | Downregulated |
| C8orf37 | 211.5091743 | 169.1301775 | -0.32259 | 0.000219 | 0.000219 | Downregulated |
| PI15 | 203.9311927 | 163.0828402 | -0.32248 | 0.000367 | 0.000367 | Downregulated |
| PAG1 | 212.587156 | 170.0236686 | -0.32232 | 0.000206 | 0.000206 | Downregulated |
| ABI2 | 212.5779817 | 170.035503 | -0.32216 | 0.000207 | 0.000207 | Downregulated |
| SRRD | 212.5733945 | 170.0414201 | -0.32207 | 0.000208 | 0.000208 | Downregulated |
| PNLIP | 2.5 | 2 | -0.32193 | 0.04498 | 0.04498 | Downregulated |
| HIST1H3H | 212.559633 | 170.0591716 | -0.32183 | 0.00021 | 0.00021 | Downregulated |
| ECEL1 | 187.1009174 | 149.7159763 | -0.32159 | 0.000876 | 0.000876 | Downregulated |
| CGRRF1 | 212.5412844 | 170.0828402 | -0.32151 | 0.000213 | 0.000213 | Downregulated |
| AZIN1 | 212.5366972 | 170.0887574 | -0.32142 | 0.000214 | 0.000214 | Downregulated |
| GRPR | 189.146789 | 151.3786982 | -0.32134 | 0.00092 | 0.00092 | Downregulated |
| SLC30A10 | 109.3027523 | 87.4852071 | -0.32122 | 0.035267 | 0.035267 | Downregulated |
| HSF2 | 212.5091743 | 170.1242604 | -0.32094 | 0.000219 | 0.000219 | Downregulated |
| ZFP1 | 212.5091743 | 170.1242604 | -0.32094 | 0.000219 | 0.000219 | Downregulated |
| MAPK12 | 212.5 | 170.1360947 | -0.32077 | 0.00022 | 0.00022 | Downregulated |
| CPSF3 | 212.5 | 170.1360947 | -0.32077 | 0.00022 | 0.00022 | Downregulated |
| ADGRG5 | 212.4908257 | 170.147929 | -0.32061 | 0.000222 | 0.000222 | Downregulated |
| FLT3 | 190.1422018 | 152.2544379 | -0.32059 | 0.000846 | 0.000846 | Downregulated |
| C17orf67 | 212.4862385 | 170.1538462 | -0.32053 | 0.000223 | 0.000223 | Downregulated |
| DOCK4 | 212.4862385 | 170.1538462 | -0.32053 | 0.000223 | 0.000223 | Downregulated |
| ZFP92 | 211.3577982 | 169.3254438 | -0.31989 | 0.000247 | 0.000247 | Downregulated |
| IL5RA | 154.7018349 | 123.9467456 | -0.31977 | 0.004972 | 0.004972 | Downregulated |
| STKLD1 | 210.2568807 | 168.4674556 | -0.31968 | 0.000267 | 0.000267 | Downregulated |
| ZNF705E | 113.1834862 | 90.73372781 | -0.31895 | 0.024091 | 0.024091 | Downregulated |
| UTP18 | 212.3899083 | 170.2781065 | -0.31882 | 0.00024 | 0.00024 | Downregulated |
| CRY1 | 212.3715596 | 170.3017751 | -0.3185 | 0.000243 | 0.000243 | Downregulated |
| RDX | 212.3486239 | 170.3313609 | -0.31809 | 0.000248 | 0.000248 | Downregulated |
| ETNK2 | 212.3348624 | 170.3491124 | -0.31785 | 0.000251 | 0.000251 | Downregulated |
| XKR6 | 206.9036697 | 165.9940828 | -0.31783 | 0.000359 | 0.000359 | Downregulated |
| PEX12 | 212.3302752 | 170.3550296 | -0.31777 | 0.000251 | 0.000251 | Downregulated |
| USP39 | 212.3211009 | 170.3668639 | -0.3176 | 0.000253 | 0.000253 | Downregulated |
| SNX10 | 212.3165138 | 170.3727811 | -0.31752 | 0.000254 | 0.000254 | Downregulated |
| CXCL5 | 211.2155963 | 169.5088757 | -0.31736 | 0.000275 | 0.000275 | Downregulated |
| ART4 | 144.5229358 | 115.9881657 | -0.31732 | 0.00505 | 0.00505 | Downregulated |
| TEFM | 212.3027523 | 170.3905325 | -0.31728 | 0.000257 | 0.000257 | Downregulated |
| TCEA2 | 212.2889908 | 170.408284 | -0.31703 | 0.00026 | 0.00026 | Downregulated |
| MAP4K2 | 212.2752294 | 170.4260355 | -0.31679 | 0.000262 | 0.000262 | Downregulated |
| ERCC4 | 212.2431193 | 170.4674556 | -0.31622 | 0.000269 | 0.000269 | Downregulated |
| ZNF440 | 212.2385321 | 170.4733728 | -0.31614 | 0.00027 | 0.00027 | Downregulated |
| BBS4 | 212.2293578 | 170.4852071 | -0.31598 | 0.000272 | 0.000272 | Downregulated |
| B4GALT5 | 212.2293578 | 170.4852071 | -0.31598 | 0.000272 | 0.000272 | Downregulated |
| DPP10 | 155.4724771 | 124.9053254 | -0.31582 | 0.003998 | 0.003998 | Downregulated |
| SRP72 | 212.2110092 | 170.5088757 | -0.31565 | 0.000276 | 0.000276 | Downregulated |
| RPN2 | 212.206422 | 170.5147929 | -0.31557 | 0.000277 | 0.000277 | Downregulated |
| SGTB | 212.2018349 | 170.5207101 | -0.31549 | 0.000278 | 0.000278 | Downregulated |
| UGT2B10 | 117.9036697 | 94.74556213 | -0.31548 | 0.019978 | 0.019978 | Downregulated |
| ZNF788 | 212.1834862 | 170.5443787 | -0.31517 | 0.000282 | 0.000282 | Downregulated |
| CACNA2D4 | 212.1788991 | 170.5502959 | -0.31508 | 0.000283 | 0.000283 | Downregulated |
| ZNF518B | 212.1651376 | 170.5680473 | -0.31484 | 0.000286 | 0.000286 | Downregulated |
| FAM209B | 160.1330275 | 128.7455621 | -0.31475 | 0.004497 | 0.004497 | Downregulated |
| CLASP2 | 212.1559633 | 170.5798817 | -0.31468 | 0.000288 | 0.000288 | Downregulated |
| HIST1H4D | 160.1284404 | 128.7514793 | -0.31464 | 0.003891 | 0.003891 | Downregulated |
| TSPAN1 | 212.1513761 | 170.5857988 | -0.3146 | 0.000289 | 0.000289 | Downregulated |
| CLEC7A | 212.1513761 | 170.5857988 | -0.3146 | 0.000289 | 0.000289 | Downregulated |
| KAT7 | 212.146789 | 170.591716 | -0.31452 | 0.00029 | 0.00029 | Downregulated |
| MAGEB17 | 156.353211 | 125.7278107 | -0.31451 | 0.006651 | 0.006651 | Downregulated |
| PRAMEF12 | 1.279816514 | 1.029585799 | -0.31387 | 0.019073 | 0.019073 | Downregulated |
| LITAF | 212.1009174 | 170.6508876 | -0.3137 | 0.0003 | 0.0003 | Downregulated |
| YPEL2 | 212.0963303 | 170.6568047 | -0.31362 | 0.000301 | 0.000301 | Downregulated |
| EDN3 | 208.8211009 | 168.0473373 | -0.3134 | 0.000374 | 0.000374 | Downregulated |
| ARHGAP15 | 212.0825688 | 170.6745562 | -0.31338 | 0.000305 | 0.000305 | Downregulated |
| HIF3A | 212.0779817 | 170.6804734 | -0.3133 | 0.000306 | 0.000306 | Downregulated |
| LRCH2 | 209.8990826 | 168.9289941 | -0.31328 | 0.000351 | 0.000351 | Downregulated |
| THRB | 210.9816514 | 169.8106509 | -0.31319 | 0.00033 | 0.00033 | Downregulated |
| GSDMC | 202.3577982 | 162.8757396 | -0.31314 | 0.00056 | 0.00056 | Downregulated |
| MYO15A | 210.9770642 | 169.816568 | -0.31311 | 0.000331 | 0.000331 | Downregulated |
| PLA2G7 | 212.059633 | 170.704142 | -0.31297 | 0.00031 | 0.00031 | Downregulated |
| STXBP4 | 212.0550459 | 170.7100592 | -0.31289 | 0.000311 | 0.000311 | Downregulated |
| HBG1 | 1.47706422 | 1.189349112 | -0.31256 | 0.042281 | 0.042281 | Downregulated |
| PDCD1LG2 | 208.7706422 | 168.112426 | -0.31249 | 0.000389 | 0.000389 | Downregulated |
| ZNF331 | 212.0321101 | 170.739645 | -0.31248 | 0.000317 | 0.000317 | Downregulated |
| KIF3B | 212.0183486 | 170.7573964 | -0.31224 | 0.00032 | 0.00032 | Downregulated |
| GPR141 | 178.5321101 | 143.7988166 | -0.31213 | 0.002618 | 0.002618 | Downregulated |
| SNAI2 | 211.9954128 | 170.7869822 | -0.31184 | 0.000326 | 0.000326 | Downregulated |
| AKAP10 | 211.9954128 | 170.7869822 | -0.31184 | 0.000326 | 0.000326 | Downregulated |
| ARHGAP6 | 211.9816514 | 170.8047337 | -0.31159 | 0.000329 | 0.000329 | Downregulated |
| RERG | 211.9770642 | 170.8106509 | -0.31151 | 0.00033 | 0.00033 | Downregulated |
| CBARP | 211.9678899 | 170.8224852 | -0.31135 | 0.000333 | 0.000333 | Downregulated |
| LRP12 | 211.9587156 | 170.8343195 | -0.31119 | 0.000335 | 0.000335 | Downregulated |
| KCNS1 | 147.9036697 | 119.2130178 | -0.31112 | 0.008943 | 0.008943 | Downregulated |
| HPGD | 211.9449541 | 170.852071 | -0.31094 | 0.000339 | 0.000339 | Downregulated |
| HSFY2 | 1.431192661 | 1.153846154 | -0.31077 | 0.017142 | 0.017142 | Downregulated |
| KCNRG | 208.6743119 | 168.2366864 | -0.31076 | 0.000418 | 0.000418 | Downregulated |
| SCML2 | 211.9311927 | 170.8698225 | -0.3107 | 0.000342 | 0.000342 | Downregulated |
| FOXO1 | 211.9311927 | 170.8698225 | -0.3107 | 0.000342 | 0.000342 | Downregulated |
| KCNE1 | 144.266055 | 116.3195266 | -0.31064 | 0.006506 | 0.006506 | Downregulated |
| P2RY14 | 208.6605505 | 168.2544379 | -0.31051 | 0.000429 | 0.000429 | Downregulated |
| TCEANC | 211.9174312 | 170.887574 | -0.31045 | 0.000346 | 0.000346 | Downregulated |
| ARHGAP10 | 211.9082569 | 170.8994083 | -0.31029 | 0.000348 | 0.000348 | Downregulated |
| MYO16 | 199.0137615 | 160.5147929 | -0.31016 | 0.000766 | 0.000766 | Downregulated |
| ZBTB46 | 211.8944954 | 170.9171598 | -0.31005 | 0.000352 | 0.000352 | Downregulated |
| P2RY12 | 162.7798165 | 131.3136095 | -0.30991 | 0.004396 | 0.004396 | Downregulated |
| C21orf91 | 211.8669725 | 170.9526627 | -0.30956 | 0.000359 | 0.000359 | Downregulated |
| GRIN2A | 127.4449541 | 102.8343195 | -0.30955 | 0.017003 | 0.017003 | Downregulated |
| ZFP37 | 209.6880734 | 169.2011834 | -0.30951 | 0.000414 | 0.000414 | Downregulated |
| FPR2 | 198.9770642 | 160.5621302 | -0.30947 | 0.000771 | 0.000771 | Downregulated |
| DRC1 | 134.4908257 | 108.556213 | -0.30907 | 0.015543 | 0.015543 | Downregulated |
| PTPN1 | 211.8256881 | 171.0059172 | -0.30883 | 0.000371 | 0.000371 | Downregulated |
| PGF | 211.8211009 | 171.0118343 | -0.30875 | 0.000372 | 0.000372 | Downregulated |
| FCGR3B | 208.559633 | 168.3846154 | -0.3087 | 0.000458 | 0.000458 | Downregulated |
| P2RY6 | 211.7889908 | 171.0532544 | -0.30818 | 0.000381 | 0.000381 | Downregulated |
| ZNF267 | 211.7798165 | 171.0650888 | -0.30802 | 0.000384 | 0.000384 | Downregulated |
| CP | 210.6880734 | 170.1893491 | -0.30797 | 0.000413 | 0.000413 | Downregulated |
| WNT5A | 211.7706422 | 171.0769231 | -0.30786 | 0.000387 | 0.000387 | Downregulated |
| DGKE | 211.7706422 | 171.0769231 | -0.30786 | 0.000387 | 0.000387 | Downregulated |
| SLC7A10 | 107.3577982 | 86.73372781 | -0.30776 | 0.039106 | 0.039106 | Downregulated |
| TMEM68 | 211.7568807 | 171.0946746 | -0.30761 | 0.000391 | 0.000391 | Downregulated |
| STAT4 | 211.7568807 | 171.0946746 | -0.30761 | 0.000391 | 0.000391 | Downregulated |
| CLEC4A | 211.7431193 | 171.112426 | -0.30737 | 0.000395 | 0.000395 | Downregulated |
| FGF9 | 199.9220183 | 161.5621302 | -0.30735 | 0.000781 | 0.000781 | Downregulated |
| CDKN2B | 211.733945 | 171.1242604 | -0.30721 | 0.000398 | 0.000398 | Downregulated |
| BCAS2 | 211.733945 | 171.1242604 | -0.30721 | 0.000398 | 0.000398 | Downregulated |
| SNRK | 211.6972477 | 171.1715976 | -0.30656 | 0.000409 | 0.000409 | Downregulated |
| STC1 | 211.6972477 | 171.1715976 | -0.30656 | 0.000409 | 0.000409 | Downregulated |
| TMEM217 | 205.1788991 | 165.964497 | -0.30601 | 0.00062 | 0.00062 | Downregulated |
| TMEM170B | 211.646789 | 171.2366864 | -0.30567 | 0.000425 | 0.000425 | Downregulated |
| FLVCR2 | 211.646789 | 171.2366864 | -0.30567 | 0.000425 | 0.000425 | Downregulated |
| CHMP4B | 211.646789 | 171.2366864 | -0.30567 | 0.000425 | 0.000425 | Downregulated |
| GPR65 | 211.646789 | 171.2366864 | -0.30567 | 0.000425 | 0.000425 | Downregulated |
| MBTD1 | 211.6376147 | 171.2485207 | -0.3055 | 0.000428 | 0.000428 | Downregulated |
| MUSK | 125.8348624 | 101.8224852 | -0.30548 | 0.023883 | 0.023883 | Downregulated |
| TP53TG5 | 211.6238532 | 171.2662722 | -0.30526 | 0.000432 | 0.000432 | Downregulated |
| C2orf49 | 211.6192661 | 171.2721893 | -0.30518 | 0.000434 | 0.000434 | Downregulated |
| UVRAG | 211.5917431 | 171.3076923 | -0.30469 | 0.000443 | 0.000443 | Downregulated |
| MFSD8 | 211.5917431 | 171.3076923 | -0.30469 | 0.000443 | 0.000443 | Downregulated |
| CCDC36 | 191.4036697 | 154.964497 | -0.30468 | 0.001427 | 0.001427 | Downregulated |
| PELI2 | 211.5825688 | 171.3195266 | -0.30453 | 0.000446 | 0.000446 | Downregulated |
| TMEM132D | 123.2981651 | 99.84023669 | -0.30446 | 0.017619 | 0.017619 | Downregulated |
| TRPC1 | 211.5733945 | 171.3313609 | -0.30437 | 0.000449 | 0.000449 | Downregulated |
| AP002495.2 | 211.5688073 | 171.3372781 | -0.30429 | 0.00045 | 0.00045 | Downregulated |
| SIGLEC5 | 187.2568807 | 151.6627219 | -0.30415 | 0.00168 | 0.00168 | Downregulated |
| HCAR3 | 160.5779817 | 130.0650888 | -0.30404 | 0.004461 | 0.004461 | Downregulated |
| TCP10 | 136.9220183 | 110.9053254 | -0.30403 | 0.026351 | 0.026351 | Downregulated |
| TLR8 | 208.2798165 | 168.7455621 | -0.30367 | 0.000567 | 0.000567 | Downregulated |
| PLSCR4 | 211.5321101 | 171.3846154 | -0.30364 | 0.000463 | 0.000463 | Downregulated |
| ERCC3 | 211.5275229 | 171.3905325 | -0.30356 | 0.000464 | 0.000464 | Downregulated |
| NRXN2 | 211.5275229 | 171.3905325 | -0.30356 | 0.000464 | 0.000464 | Downregulated |
| FBXO33 | 211.5183486 | 171.4023669 | -0.3034 | 0.000468 | 0.000468 | Downregulated |
| DOC2A | 209.3440367 | 169.6449704 | -0.30336 | 0.000536 | 0.000536 | Downregulated |
| PHF5A | 211.5137615 | 171.408284 | -0.30331 | 0.000469 | 0.000469 | Downregulated |
| NIF3L1 | 211.5137615 | 171.408284 | -0.30331 | 0.000469 | 0.000469 | Downregulated |
| SS18L1 | 211.5091743 | 171.4142012 | -0.30323 | 0.000471 | 0.000471 | Downregulated |
| STX11 | 211.5091743 | 171.4142012 | -0.30323 | 0.000471 | 0.000471 | Downregulated |
| GUCY1B3 | 211.5045872 | 171.4201183 | -0.30315 | 0.000473 | 0.000473 | Downregulated |
| RAPGEF5 | 211.4954128 | 171.4319527 | -0.30299 | 0.000476 | 0.000476 | Downregulated |
| WIF1 | 127.4266055 | 103.3017751 | -0.3028 | 0.021474 | 0.021474 | Downregulated |
| GDAP2 | 211.4816514 | 171.4497041 | -0.30275 | 0.000481 | 0.000481 | Downregulated |
| HIST1H2AG | 211.4816514 | 171.4497041 | -0.30275 | 0.000481 | 0.000481 | Downregulated |
| BCL2L11 | 211.4770642 | 171.4556213 | -0.30267 | 0.000482 | 0.000482 | Downregulated |
| C3orf58 | 211.4724771 | 171.4615385 | -0.30258 | 0.000484 | 0.000484 | Downregulated |
| PRAC1 | 112.6834862 | 91.37869822 | -0.30235 | 0.03118 | 0.03118 | Downregulated |
| STEAP4 | 211.4357798 | 171.5088757 | -0.30194 | 0.000497 | 0.000497 | Downregulated |
| HIST1H4H | 210.3394495 | 170.6390533 | -0.30177 | 0.000534 | 0.000534 | Downregulated |
| REEP1 | 211.4174312 | 171.5325444 | -0.30161 | 0.000504 | 0.000504 | Downregulated |
| CRLF3 | 211.4036697 | 171.5502959 | -0.30137 | 0.00051 | 0.00051 | Downregulated |
| PPP2R3C | 211.3761468 | 171.5857988 | -0.30088 | 0.00052 | 0.00052 | Downregulated |
| CCDC138 | 211.3715596 | 171.591716 | -0.3008 | 0.000522 | 0.000522 | Downregulated |
| TP53INP1 | 211.3577982 | 171.6094675 | -0.30056 | 0.000527 | 0.000527 | Downregulated |
| FMO2 | 180.9541284 | 146.9940828 | -0.29987 | 0.002466 | 0.002466 | Downregulated |
| PAQR3 | 211.3119266 | 171.6686391 | -0.29975 | 0.000545 | 0.000545 | Downregulated |
| MMRN1 | 203.7522936 | 165.556213 | -0.2995 | 0.00087 | 0.00087 | Downregulated |
| RGS22 | 135.0091743 | 109.7100592 | -0.29936 | 0.017444 | 0.017444 | Downregulated |
| PGBD1 | 211.2844037 | 171.704142 | -0.29926 | 0.000557 | 0.000557 | Downregulated |
| LILRA5 | 211.2752294 | 171.7159763 | -0.2991 | 0.00056 | 0.00056 | Downregulated |
| KLHL11 | 211.2706422 | 171.7218935 | -0.29902 | 0.000562 | 0.000562 | Downregulated |
| RASGRP3 | 211.266055 | 171.7278107 | -0.29894 | 0.000564 | 0.000564 | Downregulated |
| RAB11FIP2 | 211.266055 | 171.7278107 | -0.29894 | 0.000564 | 0.000564 | Downregulated |
| FAM122C | 211.266055 | 171.7278107 | -0.29894 | 0.000564 | 0.000564 | Downregulated |
| TAF8 | 211.266055 | 171.7278107 | -0.29894 | 0.000564 | 0.000564 | Downregulated |
| AQP8 | 190.0733945 | 154.5088757 | -0.29887 | 0.001817 | 0.001817 | Downregulated |
| IL4I1 | 211.2614679 | 171.7337278 | -0.29886 | 0.000566 | 0.000566 | Downregulated |
| GPN1 | 211.2522936 | 171.7455621 | -0.29869 | 0.00057 | 0.00057 | Downregulated |
| NEMP2 | 211.2431193 | 171.7573964 | -0.29853 | 0.000574 | 0.000574 | Downregulated |
| LCP2 | 211.233945 | 171.7692308 | -0.29837 | 0.000578 | 0.000578 | Downregulated |
| SLC16A12 | 128.1192661 | 104.183432 | -0.29836 | 0.014042 | 0.014042 | Downregulated |
| MANBAL | 211.2293578 | 171.7751479 | -0.29829 | 0.00058 | 0.00058 | Downregulated |
| AL049779.1 | 108.6330275 | 88.34911243 | -0.29818 | 0.029713 | 0.029713 | Downregulated |
| FAM131A | 211.1926606 | 171.7810651 | -0.29799 | 0.000586 | 0.000586 | Downregulated |
| MKS1 | 211.2018349 | 171.8106509 | -0.2978 | 0.000592 | 0.000592 | Downregulated |
| C12orf45 | 211.2018349 | 171.8106509 | -0.2978 | 0.000592 | 0.000592 | Downregulated |
| TMEM199 | 211.2018349 | 171.8106509 | -0.2978 | 0.000592 | 0.000592 | Downregulated |
| OSGEPL1 | 211.1972477 | 171.816568 | -0.29772 | 0.000594 | 0.000594 | Downregulated |
| LZTFL1 | 211.1972477 | 171.816568 | -0.29772 | 0.000594 | 0.000594 | Downregulated |
| WDPCP | 211.1880734 | 171.8284024 | -0.29756 | 0.000598 | 0.000598 | Downregulated |
| MCMBP | 211.1834862 | 171.8343195 | -0.29748 | 0.0006 | 0.0006 | Downregulated |
| WNT10B | 210.0963303 | 170.9526627 | -0.29745 | 0.00064 | 0.00064 | Downregulated |
| SWT1 | 211.1697248 | 171.852071 | -0.29724 | 0.000606 | 0.000606 | Downregulated |
| HIST1H3F | 141.0688073 | 114.816568 | -0.29707 | 0.022469 | 0.022469 | Downregulated |
| INHA | 125.5504587 | 102.1893491 | -0.29702 | 0.024905 | 0.024905 | Downregulated |
| ADGRL4 | 211.1559633 | 171.8698225 | -0.29699 | 0.000612 | 0.000612 | Downregulated |
| ZYG11B | 211.1376147 | 171.8934911 | -0.29667 | 0.00062 | 0.00062 | Downregulated |
| RLN1 | 152.8119266 | 124.4378698 | -0.29633 | 0.009554 | 0.009554 | Downregulated |
| RCAN1 | 211.1146789 | 171.9230769 | -0.29626 | 0.000631 | 0.000631 | Downregulated |
| NFKBIA | 211.1146789 | 171.9230769 | -0.29626 | 0.000631 | 0.000631 | Downregulated |
| MKNK1 | 211.1146789 | 171.9230769 | -0.29626 | 0.000631 | 0.000631 | Downregulated |
| DDIT4L | 189.940367 | 154.6804734 | -0.29626 | 0.001702 | 0.001702 | Downregulated |
| ZNF829 | 207.8577982 | 169.2899408 | -0.2961 | 0.000768 | 0.000768 | Downregulated |
| RHEBL1 | 211.1009174 | 171.9408284 | -0.29602 | 0.000637 | 0.000637 | Downregulated |
| FAM49A | 211.0963303 | 171.9467456 | -0.29594 | 0.000639 | 0.000639 | Downregulated |
| HSF2BP | 207.8394495 | 169.3136095 | -0.29577 | 0.000779 | 0.000779 | Downregulated |
| ADGRE3 | 185.8119266 | 151.3786982 | -0.29568 | 0.002476 | 0.002476 | Downregulated |
| RELL1 | 211.0779817 | 171.9704142 | -0.29562 | 0.000648 | 0.000648 | Downregulated |
| GLDC | 209.9908257 | 171.0887574 | -0.29558 | 0.000692 | 0.000692 | Downregulated |
| APOC4-APOC2 | 115.6605505 | 94.24852071 | -0.29535 | 0.032406 | 0.032406 | Downregulated |
| NEDD4 | 211.0550459 | 172 | -0.29521 | 0.000659 | 0.000659 | Downregulated |
| ELF2 | 211.0458716 | 172.0118343 | -0.29505 | 0.000663 | 0.000663 | Downregulated |
| ZNF880 | 211.0366972 | 172.0236686 | -0.29489 | 0.000668 | 0.000668 | Downregulated |
| C3AR1 | 211.0229358 | 172.0414201 | -0.29464 | 0.000675 | 0.000675 | Downregulated |
| TFPI | 211.0137615 | 172.0532544 | -0.29448 | 0.000679 | 0.000679 | Downregulated |
| POLE2 | 211.0137615 | 172.0532544 | -0.29448 | 0.000679 | 0.000679 | Downregulated |
| DDIAS | 211.0137615 | 172.0532544 | -0.29448 | 0.000679 | 0.000679 | Downregulated |
| AARD | 157.3853211 | 128.3313609 | -0.29443 | 0.010121 | 0.010121 | Downregulated |
| CASS4 | 211 | 172.0710059 | -0.29424 | 0.000686 | 0.000686 | Downregulated |
| DENND4A | 210.9908257 | 172.0828402 | -0.29408 | 0.000691 | 0.000691 | Downregulated |
| MMP25 | 210.9816514 | 172.0946746 | -0.29392 | 0.000695 | 0.000695 | Downregulated |
| LIN54 | 210.9770642 | 172.1005917 | -0.29383 | 0.000698 | 0.000698 | Downregulated |
| SLC36A1 | 210.9678899 | 172.112426 | -0.29367 | 0.000702 | 0.000702 | Downregulated |
| KPNA5 | 210.9633028 | 172.1183432 | -0.29359 | 0.000705 | 0.000705 | Downregulated |
| IFNAR2 | 210.9633028 | 172.1183432 | -0.29359 | 0.000705 | 0.000705 | Downregulated |
| SERPINB7 | 133.9311927 | 109.2781065 | -0.29349 | 0.021044 | 0.021044 | Downregulated |
| MEFV | 207.6926606 | 169.5029586 | -0.29314 | 0.000861 | 0.000861 | Downregulated |
| BTN2A2 | 210.9357798 | 172.1538462 | -0.29311 | 0.000719 | 0.000719 | Downregulated |
| DCLRE1C | 210.9311927 | 172.1597633 | -0.29302 | 0.000721 | 0.000721 | Downregulated |
| PLAG1 | 209.8440367 | 171.2781065 | -0.29298 | 0.00077 | 0.00077 | Downregulated |
| DDX1 | 210.9266055 | 172.1656805 | -0.29294 | 0.000724 | 0.000724 | Downregulated |
| SYNRG | 210.912844 | 172.183432 | -0.2927 | 0.000731 | 0.000731 | Downregulated |
| BBS9 | 210.9082569 | 172.1893491 | -0.29262 | 0.000733 | 0.000733 | Downregulated |
| MCTP1 | 210.9082569 | 172.1893491 | -0.29262 | 0.000733 | 0.000733 | Downregulated |
| ST6GALNAC3 | 210.9036697 | 172.1952663 | -0.29254 | 0.000736 | 0.000736 | Downregulated |
| C1orf109 | 210.8990826 | 172.2011834 | -0.29246 | 0.000738 | 0.000738 | Downregulated |
| ATAD5 | 210.8899083 | 172.2130178 | -0.2923 | 0.000743 | 0.000743 | Downregulated |
| EMILIN3 | 193.8669725 | 158.3372781 | -0.29207 | 0.001964 | 0.001964 | Downregulated |
| GULP1 | 210.8761468 | 172.2307692 | -0.29205 | 0.000751 | 0.000751 | Downregulated |
| ZNF280B | 210.8715596 | 172.2366864 | -0.29197 | 0.000753 | 0.000753 | Downregulated |
| MYB | 210.8669725 | 172.2426036 | -0.29189 | 0.000756 | 0.000756 | Downregulated |
| CYLD | 210.8623853 | 172.2485207 | -0.29181 | 0.000758 | 0.000758 | Downregulated |
| LRRN4 | 187.6605505 | 153.295858 | -0.29181 | 0.002914 | 0.002914 | Downregulated |
| OSER1 | 210.8577982 | 172.2544379 | -0.29173 | 0.000761 | 0.000761 | Downregulated |
| C11orf71 | 210.8486239 | 172.2662722 | -0.29157 | 0.000766 | 0.000766 | Downregulated |
| ZNF772 | 208.6743119 | 170.5088757 | -0.29141 | 0.000871 | 0.000871 | Downregulated |
| FGL2 | 210.8394495 | 172.2781065 | -0.29141 | 0.000771 | 0.000771 | Downregulated |
| MEF2C | 210.8348624 | 172.2840237 | -0.29132 | 0.000774 | 0.000774 | Downregulated |
| N4BP2L1 | 210.8348624 | 172.2840237 | -0.29132 | 0.000774 | 0.000774 | Downregulated |
| KIAA1324L | 210.8302752 | 172.2899408 | -0.29124 | 0.000776 | 0.000776 | Downregulated |
| PDXP | 210.8256881 | 172.295858 | -0.29116 | 0.000779 | 0.000779 | Downregulated |
| RGS18 | 207.5733945 | 169.6568047 | -0.291 | 0.000938 | 0.000938 | Downregulated |
| AAR2 | 210.8119266 | 172.3136095 | -0.29092 | 0.000786 | 0.000786 | Downregulated |
| TMEM179B | 210.8073394 | 172.3195266 | -0.29084 | 0.000789 | 0.000789 | Downregulated |
| MOCS1 | 210.8027523 | 172.3254438 | -0.29076 | 0.000792 | 0.000792 | Downregulated |
| SNX16 | 210.7981651 | 172.3313609 | -0.29068 | 0.000794 | 0.000794 | Downregulated |
| CA5A | 130.3944954 | 106.6094675 | -0.29055 | 0.032743 | 0.032743 | Downregulated |
| GBP5 | 210.7844037 | 172.3491124 | -0.29043 | 0.000802 | 0.000802 | Downregulated |
| AMFR | 210.7752294 | 172.3609467 | -0.29027 | 0.000808 | 0.000808 | Downregulated |
| ZNF7 | 210.7752294 | 172.3609467 | -0.29027 | 0.000808 | 0.000808 | Downregulated |
| KLRG1 | 206.440367 | 168.852071 | -0.28997 | 0.001039 | 0.001039 | Downregulated |
| CERS4 | 210.7522936 | 172.3905325 | -0.28987 | 0.000821 | 0.000821 | Downregulated |
| TNIP3 | 210.7522936 | 172.3905325 | -0.28987 | 0.000821 | 0.000821 | Downregulated |
| IL2RA | 210.7477064 | 172.3964497 | -0.28979 | 0.000824 | 0.000824 | Downregulated |
| IPP | 210.7385321 | 172.408284 | -0.28963 | 0.000829 | 0.000829 | Downregulated |
| GP5 | 152.5366972 | 124.7928994 | -0.28962 | 0.012077 | 0.012077 | Downregulated |
| FAM104A | 210.7293578 | 172.4201183 | -0.28946 | 0.000835 | 0.000835 | Downregulated |
| AQP12B | 146.1422018 | 119.5798817 | -0.2894 | 0.016977 | 0.016977 | Downregulated |
| PDE6A | 207.4816514 | 169.7751479 | -0.28936 | 0.000996 | 0.000996 | Downregulated |
| TNFRSF9 | 210.7201835 | 172.4319527 | -0.2893 | 0.00084 | 0.00084 | Downregulated |
| ARNTL | 210.7201835 | 172.4319527 | -0.2893 | 0.00084 | 0.00084 | Downregulated |
| NKIRAS1 | 210.7110092 | 172.443787 | -0.28914 | 0.000846 | 0.000846 | Downregulated |
| CNRIP1 | 210.7110092 | 172.443787 | -0.28914 | 0.000846 | 0.000846 | Downregulated |
| SFMBT2 | 210.706422 | 172.4497041 | -0.28906 | 0.000849 | 0.000849 | Downregulated |
| POLR2C | 210.6926606 | 172.4674556 | -0.28882 | 0.000857 | 0.000857 | Downregulated |
| DUSP19 | 210.6880734 | 172.4733728 | -0.28873 | 0.00086 | 0.00086 | Downregulated |
| TMEM38A | 210.6834862 | 172.4792899 | -0.28865 | 0.000863 | 0.000863 | Downregulated |
| RTN4RL2 | 210.6788991 | 172.4852071 | -0.28857 | 0.000866 | 0.000866 | Downregulated |
| RTFDC1 | 210.6743119 | 172.4911243 | -0.28849 | 0.000868 | 0.000868 | Downregulated |
| SPNS1 | 210.6697248 | 172.4970414 | -0.28841 | 0.000871 | 0.000871 | Downregulated |
| ASIP | 174.4266055 | 142.8284024 | -0.28834 | 0.005482 | 0.005482 | Downregulated |
| HGF | 210.6651376 | 172.5029586 | -0.28833 | 0.000874 | 0.000874 | Downregulated |
| PUS10 | 210.6605505 | 172.5088757 | -0.28825 | 0.000877 | 0.000877 | Downregulated |
| TRPC6 | 210.6559633 | 172.5147929 | -0.28817 | 0.00088 | 0.00088 | Downregulated |
| NAALADL1 | 210.646789 | 172.5266272 | -0.28801 | 0.000886 | 0.000886 | Downregulated |
| TMEM86A | 210.6376147 | 172.5384615 | -0.28785 | 0.000892 | 0.000892 | Downregulated |
| TTLL9 | 199.9082569 | 163.8047337 | -0.28736 | 0.001635 | 0.001635 | Downregulated |
| CENPL | 210.6009174 | 172.5857988 | -0.2872 | 0.000915 | 0.000915 | Downregulated |
| PAPD5 | 210.5779817 | 172.6153846 | -0.28679 | 0.00093 | 0.00093 | Downregulated |
| GJC1 | 210.5688073 | 172.6272189 | -0.28663 | 0.000936 | 0.000936 | Downregulated |
| PKIB | 210.5642202 | 172.6331361 | -0.28655 | 0.00094 | 0.00094 | Downregulated |
| ZNF429 | 210.5458716 | 172.6568047 | -0.28623 | 0.000952 | 0.000952 | Downregulated |
| PRIM2 | 210.5458716 | 172.6568047 | -0.28623 | 0.000952 | 0.000952 | Downregulated |
| CHRNE | 209.1055046 | 171.4792899 | -0.2862 | 0.000995 | 0.000995 | Downregulated |
| CA5B | 210.5412844 | 172.6627219 | -0.28615 | 0.000955 | 0.000955 | Downregulated |
| ZNF821 | 210.5366972 | 172.6686391 | -0.28607 | 0.000958 | 0.000958 | Downregulated |
| PITPNB | 210.5366972 | 172.6686391 | -0.28607 | 0.000958 | 0.000958 | Downregulated |
| BDKRB2 | 210.5321101 | 172.6745562 | -0.28598 | 0.000961 | 0.000961 | Downregulated |
| SLC25A36 | 210.5321101 | 172.6745562 | -0.28598 | 0.000961 | 0.000961 | Downregulated |
| SCNN1B | 207.2706422 | 170.0473373 | -0.28558 | 0.001173 | 0.001173 | Downregulated |
| KMO | 208.3348624 | 170.9467456 | -0.28536 | 0.001113 | 0.001113 | Downregulated |
| CDC14A | 210.4954128 | 172.7218935 | -0.28534 | 0.000987 | 0.000987 | Downregulated |
| SPATA25 | 207.2568807 | 170.0650888 | -0.28533 | 0.00119 | 0.00119 | Downregulated |
| RNF115 | 210.4908257 | 172.7278107 | -0.28526 | 0.00099 | 0.00099 | Downregulated |
| KISS1R | 166.4770642 | 136.6331361 | -0.28502 | 0.006957 | 0.006957 | Downregulated |
| RSPO4 | 124.3119266 | 102.0295858 | -0.28498 | 0.036512 | 0.036512 | Downregulated |
| COMMD7 | 210.4678899 | 172.7573964 | -0.28485 | 0.001006 | 0.001006 | Downregulated |
| HPS3 | 210.4678899 | 172.7573964 | -0.28485 | 0.001006 | 0.001006 | Downregulated |
| XRN2 | 210.4678899 | 172.7573964 | -0.28485 | 0.001006 | 0.001006 | Downregulated |
| TIGD3 | 210.4633028 | 172.7633136 | -0.28477 | 0.001009 | 0.001009 | Downregulated |
| HERPUD1 | 210.4633028 | 172.7633136 | -0.28477 | 0.001009 | 0.001009 | Downregulated |
| HIST2H4A | 146.8623853 | 120.556213 | -0.28476 | 0.017484 | 0.017484 | Downregulated |
| NR5A2 | 210.4587156 | 172.7692308 | -0.28469 | 0.001013 | 0.001013 | Downregulated |
| EYA4 | 167.4220183 | 137.4497041 | -0.28459 | 0.007851 | 0.007851 | Downregulated |
| HBS1L | 210.4495413 | 172.7810651 | -0.28453 | 0.001019 | 0.001019 | Downregulated |
| CNKSR2 | 175.233945 | 143.8698225 | -0.28452 | 0.006007 | 0.006007 | Downregulated |
| APOBR | 210.4449541 | 172.7869822 | -0.28445 | 0.001023 | 0.001023 | Downregulated |
| SEC61A2 | 210.4220183 | 172.816568 | -0.28404 | 0.001039 | 0.001039 | Downregulated |
| RRAGC | 210.4174312 | 172.8224852 | -0.28396 | 0.001043 | 0.001043 | Downregulated |
| SLC10A7 | 210.412844 | 172.8284024 | -0.28388 | 0.001046 | 0.001046 | Downregulated |
| USP25 | 210.4082569 | 172.8343195 | -0.2838 | 0.00105 | 0.00105 | Downregulated |
| NPFFR2 | 93.27522936 | 76.62130178 | -0.28375 | 0.020393 | 0.020393 | Downregulated |
| GTDC1 | 210.4036697 | 172.8402367 | -0.28372 | 0.001053 | 0.001053 | Downregulated |
| HEBP1 | 210.3990826 | 172.8461538 | -0.28364 | 0.001056 | 0.001056 | Downregulated |
| ZNF804A | 176.1788991 | 144.739645 | -0.28358 | 0.005024 | 0.005024 | Downregulated |
| SEMA6D | 210.3853211 | 172.8639053 | -0.2834 | 0.001067 | 0.001067 | Downregulated |
| PABPC5 | 193.3899083 | 158.9526627 | -0.28292 | 0.002509 | 0.002509 | Downregulated |
| CLTC | 210.353211 | 172.9053254 | -0.28283 | 0.001091 | 0.001091 | Downregulated |
| HIST1H2BJ | 210.353211 | 172.9053254 | -0.28283 | 0.001091 | 0.001091 | Downregulated |
| MS4A14 | 203.9036697 | 167.6094675 | -0.28278 | 0.001565 | 0.001565 | Downregulated |
| VPS25 | 210.3394495 | 172.9230769 | -0.28259 | 0.001102 | 0.001102 | Downregulated |
| FAM126A | 210.3394495 | 172.9230769 | -0.28259 | 0.001102 | 0.001102 | Downregulated |
| IRAK2 | 210.3348624 | 172.9289941 | -0.28251 | 0.001105 | 0.001105 | Downregulated |
| SLC33A1 | 210.3348624 | 172.9289941 | -0.28251 | 0.001105 | 0.001105 | Downregulated |
| IKZF5 | 210.3348624 | 172.9289941 | -0.28251 | 0.001105 | 0.001105 | Downregulated |
| PALD1 | 210.3165138 | 172.9526627 | -0.28218 | 0.00112 | 0.00112 | Downregulated |
| PSME3 | 210.3165138 | 172.9526627 | -0.28218 | 0.00112 | 0.00112 | Downregulated |
| AKTIP | 210.3119266 | 172.9585799 | -0.2821 | 0.001123 | 0.001123 | Downregulated |
| CCDC89 | 197.5137615 | 162.4497041 | -0.28196 | 0.002135 | 0.002135 | Downregulated |
| R3HCC1L | 210.3027523 | 172.9704142 | -0.28194 | 0.001131 | 0.001131 | Downregulated |
| ACR | 111.2798165 | 91.52662722 | -0.28193 | 0.045518 | 0.045518 | Downregulated |
| SEC22A | 210.2981651 | 172.9763314 | -0.28186 | 0.001134 | 0.001134 | Downregulated |
| MOB1A | 210.2981651 | 172.9763314 | -0.28186 | 0.001134 | 0.001134 | Downregulated |
| PTPN7 | 210.2981651 | 172.9763314 | -0.28186 | 0.001134 | 0.001134 | Downregulated |
| LSM14B | 209.5688073 | 172.3786982 | -0.28184 | 0.001134 | 0.001134 | Downregulated |
| NKAPL | 153.1284404 | 125.9763314 | -0.28159 | 0.010885 | 0.010885 | Downregulated |
| TRIM23 | 210.2568807 | 173.0295858 | -0.28113 | 0.001168 | 0.001168 | Downregulated |
| FASTKD3 | 210.2522936 | 173.035503 | -0.28105 | 0.001171 | 0.001171 | Downregulated |
| XYLB | 210.2477064 | 173.0414201 | -0.28097 | 0.001175 | 0.001175 | Downregulated |
| CA1 | 186.087156 | 153.1715976 | -0.28083 | 0.003638 | 0.003638 | Downregulated |
| IGSF1 | 178.0275229 | 146.5502959 | -0.2807 | 0.005526 | 0.005526 | Downregulated |
| E2F6 | 210.2293578 | 173.0650888 | -0.28065 | 0.00119 | 0.00119 | Downregulated |
| FXYD1 | 185.059633 | 152.3491124 | -0.28061 | 0.004318 | 0.004318 | Downregulated |
| COL27A1 | 210.2155963 | 173.0828402 | -0.28041 | 0.001202 | 0.001202 | Downregulated |
| ZNF726 | 192.2201835 | 158.2721893 | -0.28035 | 0.002981 | 0.002981 | Downregulated |
| GAPT | 202.7018349 | 166.9112426 | -0.28028 | 0.00185 | 0.00185 | Downregulated |
| PTDSS1 | 210.2018349 | 173.1005917 | -0.28016 | 0.001214 | 0.001214 | Downregulated |
| M1AP | 176.0137615 | 144.9526627 | -0.28011 | 0.006101 | 0.006101 | Downregulated |
| CTCF | 210.1972477 | 173.1065089 | -0.28008 | 0.001218 | 0.001218 | Downregulated |
| TM6SF1 | 209.1146789 | 172.2189349 | -0.28005 | 0.001292 | 0.001292 | Downregulated |
| DHX36 | 210.1926606 | 173.112426 | -0.28 | 0.001221 | 0.001221 | Downregulated |
| C16orf72 | 210.1880734 | 173.1183432 | -0.27992 | 0.001225 | 0.001225 | Downregulated |
| MYBL1 | 210.1880734 | 173.1183432 | -0.27992 | 0.001225 | 0.001225 | Downregulated |
| AMPH | 208.0229358 | 171.3491124 | -0.2798 | 0.001383 | 0.001383 | Downregulated |
| CCDC88A | 210.1697248 | 173.1420118 | -0.2796 | 0.001241 | 0.001241 | Downregulated |
| ZNF570 | 210.1605505 | 173.1538462 | -0.27944 | 0.001249 | 0.001249 | Downregulated |
| PDE7A | 210.1559633 | 173.1597633 | -0.27936 | 0.001253 | 0.001253 | Downregulated |
| CHST2 | 210.1513761 | 173.1656805 | -0.27928 | 0.001257 | 0.001257 | Downregulated |
| ZNF347 | 210.1513761 | 173.1656805 | -0.27928 | 0.001257 | 0.001257 | Downregulated |
| GDAP1L1 | 151.1926606 | 124.5857988 | -0.27925 | 0.017628 | 0.017628 | Downregulated |
| SRC | 210.146789 | 173.1715976 | -0.2792 | 0.001261 | 0.001261 | Downregulated |
| PPP4R4 | 178.9495413 | 147.4674556 | -0.27916 | 0.004991 | 0.004991 | Downregulated |
| ZNF730 | 115.9541284 | 95.56213018 | -0.27904 | 0.030841 | 0.030841 | Downregulated |
| C18orf54 | 210.1376147 | 173.183432 | -0.27903 | 0.001269 | 0.001269 | Downregulated |
| VDAC1 | 210.1330275 | 173.1893491 | -0.27895 | 0.001273 | 0.001273 | Downregulated |
| ZNF853 | 210.1330275 | 173.1893491 | -0.27895 | 0.001273 | 0.001273 | Downregulated |
| KCNJ8 | 210.1284404 | 173.1952663 | -0.27887 | 0.001277 | 0.001277 | Downregulated |
| KCNJ15 | 207.9633028 | 171.4260355 | -0.27874 | 0.001437 | 0.001437 | Downregulated |
| ATP8B4 | 210.1100917 | 173.2189349 | -0.27855 | 0.001294 | 0.001294 | Downregulated |
| HTR3A | 133.3899083 | 109.9763314 | -0.27846 | 0.027907 | 0.027907 | Downregulated |
| KCNK13 | 204.7293578 | 168.7988166 | -0.27841 | 0.001722 | 0.001722 | Downregulated |
| ZNF93 | 210.1009174 | 173.2307692 | -0.27839 | 0.001302 | 0.001302 | Downregulated |
| NDUFAF5 | 210.1009174 | 173.2307692 | -0.27839 | 0.001302 | 0.001302 | Downregulated |
| TCEAL7 | 202.5963303 | 167.0473373 | -0.27835 | 0.001944 | 0.001944 | Downregulated |
| SLA | 210.0963303 | 173.2366864 | -0.27831 | 0.001306 | 0.001306 | Downregulated |
| PPM1E | 197.3119266 | 162.7100592 | -0.27817 | 0.002532 | 0.002532 | Downregulated |
| RNF152 | 210.087156 | 173.2485207 | -0.27814 | 0.001315 | 0.001315 | Downregulated |
| METTL2A | 210.0779817 | 173.260355 | -0.27798 | 0.001323 | 0.001323 | Downregulated |
| SHE | 210.0779817 | 173.260355 | -0.27798 | 0.001323 | 0.001323 | Downregulated |
| ICOS | 208.9954128 | 172.3727811 | -0.27794 | 0.001401 | 0.001401 | Downregulated |
| EVI2A | 210.0688073 | 173.2721893 | -0.27782 | 0.001332 | 0.001332 | Downregulated |
| CPNE3 | 210.0550459 | 173.2899408 | -0.27758 | 0.001344 | 0.001344 | Downregulated |
| ARHGEF6 | 210.0504587 | 173.295858 | -0.2775 | 0.001349 | 0.001349 | Downregulated |
| PTPRC | 210.0504587 | 173.295858 | -0.2775 | 0.001349 | 0.001349 | Downregulated |
| TMEM26 | 210.0458716 | 173.3017751 | -0.27742 | 0.001353 | 0.001353 | Downregulated |
| ZNF675 | 210.0458716 | 173.3017751 | -0.27742 | 0.001353 | 0.001353 | Downregulated |
| CD86 | 210.0458716 | 173.3017751 | -0.27742 | 0.001353 | 0.001353 | Downregulated |
| 2-Sep | 210.0458716 | 173.3017751 | -0.27742 | 0.001353 | 0.001353 | Downregulated |
| C6orf58 | 149.2844037 | 123.183432 | -0.27726 | 0.022681 | 0.022681 | Downregulated |
| MOSPD2 | 210.0321101 | 173.3195266 | -0.27718 | 0.001366 | 0.001366 | Downregulated |
| PLA2G12B | 166.1238532 | 137.0887574 | -0.27715 | 0.01079 | 0.01079 | Downregulated |
| STEAP1B | 189.9862385 | 156.7928994 | -0.27703 | 0.00387 | 0.00387 | Downregulated |
| HMGXB4 | 210.0091743 | 173.3491124 | -0.27677 | 0.001388 | 0.001388 | Downregulated |
| SLAMF1 | 210.0091743 | 173.3491124 | -0.27677 | 0.001388 | 0.001388 | Downregulated |
| CLEC2B | 210.0045872 | 173.3550296 | -0.27669 | 0.001392 | 0.001392 | Downregulated |
| SLC7A7 | 209.9954128 | 173.3668639 | -0.27653 | 0.001401 | 0.001401 | Downregulated |
| NEURL2 | 206.7568807 | 170.7100592 | -0.27639 | 0.001674 | 0.001674 | Downregulated |
| GAB3 | 209.9862385 | 173.3786982 | -0.27637 | 0.00141 | 0.00141 | Downregulated |
| MCM8 | 209.9770642 | 173.3905325 | -0.27621 | 0.001419 | 0.001419 | Downregulated |
| STAR | 159.4220183 | 131.6508876 | -0.27613 | 0.016599 | 0.016599 | Downregulated |
| TBC1D32 | 209.9724771 | 173.3964497 | -0.27613 | 0.001423 | 0.001423 | Downregulated |
| CHMP2B | 209.9724771 | 173.3964497 | -0.27613 | 0.001423 | 0.001423 | Downregulated |
| AC010422.6 | 209.9678899 | 173.4023669 | -0.27605 | 0.001428 | 0.001428 | Downregulated |
| EDNRA | 209.9678899 | 173.4023669 | -0.27605 | 0.001428 | 0.001428 | Downregulated |
| GPR180 | 209.9678899 | 173.4023669 | -0.27605 | 0.001428 | 0.001428 | Downregulated |
| STOX1 | 209.9678899 | 173.4023669 | -0.27605 | 0.001428 | 0.001428 | Downregulated |
| SMARCE1 | 209.9633028 | 173.408284 | -0.27596 | 0.001433 | 0.001433 | Downregulated |
| GPR137C | 207.7981651 | 171.6390533 | -0.27581 | 0.001615 | 0.001615 | Downregulated |
| USP37 | 209.9495413 | 173.4260355 | -0.27572 | 0.001446 | 0.001446 | Downregulated |
| RBMXL1 | 209.9449541 | 173.4319527 | -0.27564 | 0.001451 | 0.001451 | Downregulated |
| MAMSTR | 209.9449541 | 173.4319527 | -0.27564 | 0.001451 | 0.001451 | Downregulated |
| KLHL5 | 209.9311927 | 173.4497041 | -0.2754 | 0.001465 | 0.001465 | Downregulated |
| ABCC4 | 209.9311927 | 173.4497041 | -0.2754 | 0.001465 | 0.001465 | Downregulated |
| CPA3 | 208.8440367 | 172.5680473 | -0.27526 | 0.00156 | 0.00156 | Downregulated |
| RSBN1L | 209.9220183 | 173.4615385 | -0.27524 | 0.001474 | 0.001474 | Downregulated |
| GPR18 | 204.5458716 | 169.035503 | -0.2751 | 0.00198 | 0.00198 | Downregulated |
| CSF2RA | 209.912844 | 173.4733728 | -0.27508 | 0.001483 | 0.001483 | Downregulated |
| MLC1 | 192.9770642 | 159.4852071 | -0.27501 | 0.003362 | 0.003362 | Downregulated |
| SEC14L6 | 159.3623853 | 131.7278107 | -0.27475 | 0.018245 | 0.018245 | Downregulated |
| SLC15A1 | 184.766055 | 152.7278107 | -0.27474 | 0.004843 | 0.004843 | Downregulated |
| TBCCD1 | 209.8899083 | 173.5029586 | -0.27467 | 0.001507 | 0.001507 | Downregulated |
| SMPX | 126.4633028 | 104.5443787 | -0.2746 | 0.018948 | 0.018948 | Downregulated |
| ITGB3 | 209.8761468 | 173.5207101 | -0.27443 | 0.001521 | 0.001521 | Downregulated |
| CCDC102A | 209.8715596 | 173.5266272 | -0.27435 | 0.001526 | 0.001526 | Downregulated |
| TIPIN | 209.8715596 | 173.5266272 | -0.27435 | 0.001526 | 0.001526 | Downregulated |
| EML6 | 209.8669725 | 173.5325444 | -0.27427 | 0.001531 | 0.001531 | Downregulated |
| ZNF655 | 209.8623853 | 173.5384615 | -0.27419 | 0.001536 | 0.001536 | Downregulated |
| NR3C1 | 209.8348624 | 173.5739645 | -0.2737 | 0.001565 | 0.001565 | Downregulated |
| LOX | 209.8256881 | 173.5857988 | -0.27354 | 0.001575 | 0.001575 | Downregulated |
| TP73 | 209.8211009 | 173.591716 | -0.27346 | 0.00158 | 0.00158 | Downregulated |
| IL17RA | 209.8211009 | 173.591716 | -0.27346 | 0.00158 | 0.00158 | Downregulated |
| ZNF34 | 209.8165138 | 173.5976331 | -0.27338 | 0.001585 | 0.001585 | Downregulated |
| ZIK1 | 209.8165138 | 173.5976331 | -0.27338 | 0.001585 | 0.001585 | Downregulated |
| HBQ1 | 102.5688073 | 84.86390533 | -0.27337 | 0.037421 | 0.037421 | Downregulated |
| ANKRD13A | 209.8119266 | 173.6035503 | -0.2733 | 0.00159 | 0.00159 | Downregulated |
| FLCN | 209.8119266 | 173.6035503 | -0.2733 | 0.00159 | 0.00159 | Downregulated |
| HIST1H2AH | 141.0458716 | 116.7159763 | -0.27316 | 0.028348 | 0.028348 | Downregulated |
| ADAL | 209.8027523 | 173.6153846 | -0.27314 | 0.0016 | 0.0016 | Downregulated |
| MRC1 | 209.8027523 | 173.6153846 | -0.27314 | 0.0016 | 0.0016 | Downregulated |
| EDNRB | 209.7981651 | 173.6213018 | -0.27306 | 0.001605 | 0.001605 | Downregulated |
| SLC43A3 | 209.7981651 | 173.6213018 | -0.27306 | 0.001605 | 0.001605 | Downregulated |
| DDX52 | 209.793578 | 173.6272189 | -0.27298 | 0.00161 | 0.00161 | Downregulated |
| SSX2IP | 209.793578 | 173.6272189 | -0.27298 | 0.00161 | 0.00161 | Downregulated |
| PIGK | 209.7889908 | 173.6331361 | -0.2729 | 0.001615 | 0.001615 | Downregulated |
| KLHDC3 | 209.7889908 | 173.6331361 | -0.2729 | 0.001615 | 0.001615 | Downregulated |
| TNFSF10 | 209.7798165 | 173.6449704 | -0.27274 | 0.001625 | 0.001625 | Downregulated |
| FTO | 209.7752294 | 173.6508876 | -0.27265 | 0.00163 | 0.00163 | Downregulated |
| ZNF451 | 209.1880734 | 173.1656805 | -0.27265 | 0.00162 | 0.00162 | Downregulated |
| IGSF6 | 209.7706422 | 173.6568047 | -0.27257 | 0.001635 | 0.001635 | Downregulated |
| UBE2Z | 209.7706422 | 173.6568047 | -0.27257 | 0.001635 | 0.001635 | Downregulated |
| STAT3 | 209.766055 | 173.6627219 | -0.27249 | 0.00164 | 0.00164 | Downregulated |
| SEMA6A | 209.766055 | 173.6627219 | -0.27249 | 0.00164 | 0.00164 | Downregulated |
| OSTM1 | 209.766055 | 173.6627219 | -0.27249 | 0.00164 | 0.00164 | Downregulated |
| IL18RAP | 205.4678899 | 170.1065089 | -0.27247 | 0.002033 | 0.002033 | Downregulated |
| PPM1A | 209.7614679 | 173.6686391 | -0.27241 | 0.001646 | 0.001646 | Downregulated |
| FAM171B | 207.6055046 | 171.887574 | -0.27238 | 0.001841 | 0.001841 | Downregulated |
| CSRP2 | 209.7568807 | 173.6745562 | -0.27233 | 0.001651 | 0.001651 | Downregulated |
| SP4 | 209.7522936 | 173.6804734 | -0.27225 | 0.001656 | 0.001656 | Downregulated |
| TUBB1 | 200.1422018 | 165.7337278 | -0.27216 | 0.002706 | 0.002706 | Downregulated |
| TCTEX1D1 | 191.793578 | 158.8224852 | -0.27214 | 0.003825 | 0.003825 | Downregulated |
| ITGA4 | 209.7431193 | 173.6923077 | -0.27209 | 0.001666 | 0.001666 | Downregulated |
| GPR160 | 209.7431193 | 173.6923077 | -0.27209 | 0.001666 | 0.001666 | Downregulated |
| EFCAB6 | 207.5825688 | 171.9171598 | -0.27197 | 0.001864 | 0.001864 | Downregulated |
| KCND3 | 209.733945 | 173.704142 | -0.27193 | 0.001677 | 0.001677 | Downregulated |
| H2AFV | 209.733945 | 173.704142 | -0.27193 | 0.001677 | 0.001677 | Downregulated |
| NUDT3 | 209.7293578 | 173.7100592 | -0.27185 | 0.001682 | 0.001682 | Downregulated |
| GNG12 | 209.7293578 | 173.7100592 | -0.27185 | 0.001682 | 0.001682 | Downregulated |
| EIF6 | 209.7293578 | 173.7100592 | -0.27185 | 0.001682 | 0.001682 | Downregulated |
| POLI | 209.7201835 | 173.7218935 | -0.27169 | 0.001693 | 0.001693 | Downregulated |
| RNF166 | 209.6880734 | 173.7633136 | -0.27112 | 0.00173 | 0.00173 | Downregulated |
| ADAMTS5 | 209.6788991 | 173.7751479 | -0.27096 | 0.001741 | 0.001741 | Downregulated |
| SLC9B2 | 209.6743119 | 173.7810651 | -0.27088 | 0.001746 | 0.001746 | Downregulated |
| ZNF556 | 145.4082569 | 120.5266272 | -0.27076 | 0.027078 | 0.027078 | Downregulated |
| CIDEB | 136.5688073 | 113.2011834 | -0.27074 | 0.038126 | 0.038126 | Downregulated |
| C9orf72 | 209.6651376 | 173.7928994 | -0.27072 | 0.001757 | 0.001757 | Downregulated |
| HSDL1 | 209.6605505 | 173.7988166 | -0.27064 | 0.001763 | 0.001763 | Downregulated |
| NEURL3 | 209.6605505 | 173.7988166 | -0.27064 | 0.001763 | 0.001763 | Downregulated |
| HOMER1 | 209.6559633 | 173.8047337 | -0.27056 | 0.001768 | 0.001768 | Downregulated |
| PLXNC1 | 209.6513761 | 173.8106509 | -0.27048 | 0.001774 | 0.001774 | Downregulated |
| EML5 | 158.233945 | 131.1952663 | -0.27034 | 0.022961 | 0.022961 | Downregulated |
| OTULIN | 209.6422018 | 173.8224852 | -0.27031 | 0.001785 | 0.001785 | Downregulated |
| MNDA | 209.6376147 | 173.8284024 | -0.27023 | 0.00179 | 0.00179 | Downregulated |
| GABRB3 | 184.5275229 | 153.035503 | -0.26997 | 0.006002 | 0.006002 | Downregulated |
| SERAC1 | 209.6192661 | 173.852071 | -0.26991 | 0.001813 | 0.001813 | Downregulated |
| CIAO1 | 209.6100917 | 173.8639053 | -0.26975 | 0.001824 | 0.001824 | Downregulated |
| DRG1 | 209.6100917 | 173.8639053 | -0.26975 | 0.001824 | 0.001824 | Downregulated |
| ZCCHC11 | 209.6100917 | 173.8639053 | -0.26975 | 0.001824 | 0.001824 | Downregulated |
| ZNF677 | 208.5321101 | 172.9704142 | -0.26974 | 0.001929 | 0.001929 | Downregulated |
| FOXRED2 | 209.6055046 | 173.8698225 | -0.26967 | 0.00183 | 0.00183 | Downregulated |
| NOVA1 | 188.5825688 | 156.4319527 | -0.26966 | 0.004929 | 0.004929 | Downregulated |
| HIST1H4C | 159.1422018 | 132.0118343 | -0.26965 | 0.019309 | 0.019309 | Downregulated |
| SIGLEC8 | 195.8119266 | 162.4319527 | -0.26963 | 0.003609 | 0.003609 | Downregulated |
| TMEM97 | 209.6009174 | 173.8757396 | -0.26959 | 0.001836 | 0.001836 | Downregulated |
| MAST1 | 209.0733945 | 173.443787 | -0.26954 | 0.001799 | 0.001799 | Downregulated |
| C1orf52 | 209.5963303 | 173.8816568 | -0.26951 | 0.001841 | 0.001841 | Downregulated |
| LYPLA1 | 209.5917431 | 173.887574 | -0.26943 | 0.001847 | 0.001847 | Downregulated |
| TPX2 | 209.587156 | 173.8934911 | -0.26935 | 0.001853 | 0.001853 | Downregulated |
| TSN | 209.5825688 | 173.8994083 | -0.26927 | 0.001858 | 0.001858 | Downregulated |
| CCDC126 | 209.5825688 | 173.8994083 | -0.26927 | 0.001858 | 0.001858 | Downregulated |
| USP31 | 209.5779817 | 173.9053254 | -0.26919 | 0.001864 | 0.001864 | Downregulated |
| CCDC121 | 208.5 | 173.0118343 | -0.26918 | 0.001968 | 0.001968 | Downregulated |
| TCP11L1 | 209.5688073 | 173.9171598 | -0.26902 | 0.001876 | 0.001876 | Downregulated |
| CFAP43 | 206.3394495 | 171.2485207 | -0.26893 | 0.002209 | 0.002209 | Downregulated |
| LPAR4 | 162.8853211 | 135.1952663 | -0.26881 | 0.012027 | 0.012027 | Downregulated |
| PKLR | 183.4587156 | 152.2721893 | -0.2688 | 0.006838 | 0.006838 | Downregulated |
| DNAJC27 | 209.5504587 | 173.9408284 | -0.2687 | 0.001899 | 0.001899 | Downregulated |
| RBM48 | 209.5504587 | 173.9408284 | -0.2687 | 0.001899 | 0.001899 | Downregulated |
| ABCC9 | 209.5458716 | 173.9467456 | -0.26862 | 0.001905 | 0.001905 | Downregulated |
| DNAJB14 | 209.5412844 | 173.9526627 | -0.26854 | 0.001911 | 0.001911 | Downregulated |
| GNB4 | 209.5366972 | 173.9585799 | -0.26846 | 0.001917 | 0.001917 | Downregulated |
| CFAP20 | 209.5321101 | 173.964497 | -0.26838 | 0.001923 | 0.001923 | Downregulated |
| TFPI2 | 209.5321101 | 173.964497 | -0.26838 | 0.001923 | 0.001923 | Downregulated |
| BCAP31 | 209.5229358 | 173.9763314 | -0.26822 | 0.001935 | 0.001935 | Downregulated |
| FBXO9 | 209.5137615 | 173.9881657 | -0.26806 | 0.001947 | 0.001947 | Downregulated |
| ARHGAP19 | 209.5045872 | 174 | -0.26789 | 0.001959 | 0.001959 | Downregulated |
| CDK19 | 209.5 | 174.0059172 | -0.26781 | 0.001965 | 0.001965 | Downregulated |
| EME1 | 209.5 | 174.0059172 | -0.26781 | 0.001965 | 0.001965 | Downregulated |
| EIF4A3 | 209.5 | 174.0059172 | -0.26781 | 0.001965 | 0.001965 | Downregulated |
| RAD18 | 209.4908257 | 174.0177515 | -0.26765 | 0.001977 | 0.001977 | Downregulated |
| ZNF557 | 209.4908257 | 174.0177515 | -0.26765 | 0.001977 | 0.001977 | Downregulated |
| MICU3 | 191.559633 | 159.1242604 | -0.26764 | 0.004434 | 0.004434 | Downregulated |
| DUSP28 | 209.4862385 | 174.0236686 | -0.26757 | 0.001983 | 0.001983 | Downregulated |
| RASL12 | 209.4816514 | 174.0295858 | -0.26749 | 0.001989 | 0.001989 | Downregulated |
| CNKSR3 | 209.4770642 | 174.035503 | -0.26741 | 0.001996 | 0.001996 | Downregulated |
| PIGH | 209.4724771 | 174.0414201 | -0.26733 | 0.002002 | 0.002002 | Downregulated |
| CEPT1 | 209.4724771 | 174.0414201 | -0.26733 | 0.002002 | 0.002002 | Downregulated |
| ZNF143 | 209.4587156 | 174.0591716 | -0.26709 | 0.00202 | 0.00202 | Downregulated |
| ZCWPW2 | 196.7155963 | 163.4792899 | -0.267 | 0.003727 | 0.003727 | Downregulated |
| ZNF548 | 209.4495413 | 174.0710059 | -0.26693 | 0.002033 | 0.002033 | Downregulated |
| DNMT3B | 209.440367 | 174.0828402 | -0.26677 | 0.002045 | 0.002045 | Downregulated |
| NUB1 | 209.4357798 | 174.0887574 | -0.26668 | 0.002052 | 0.002052 | Downregulated |
| CKAP5 | 209.4311927 | 174.0946746 | -0.2666 | 0.002058 | 0.002058 | Downregulated |
| RCSD1 | 209.4266055 | 174.1005917 | -0.26652 | 0.002064 | 0.002064 | Downregulated |
| KIAA1462 | 209.4266055 | 174.1005917 | -0.26652 | 0.002064 | 0.002064 | Downregulated |
| BRMS1L | 209.4266055 | 174.1005917 | -0.26652 | 0.002064 | 0.002064 | Downregulated |
| SLAMF7 | 209.4220183 | 174.1065089 | -0.26644 | 0.002071 | 0.002071 | Downregulated |
| SLC4A7 | 209.4174312 | 174.112426 | -0.26636 | 0.002077 | 0.002077 | Downregulated |
| TADA2A | 209.412844 | 174.1183432 | -0.26628 | 0.002083 | 0.002083 | Downregulated |
| WISP1 | 209.4082569 | 174.1242604 | -0.2662 | 0.00209 | 0.00209 | Downregulated |
| ZNF334 | 194.5825688 | 161.8106509 | -0.26608 | 0.004055 | 0.004055 | Downregulated |
| GHRL | 204.0366972 | 169.6923077 | -0.26591 | 0.002755 | 0.002755 | Downregulated |
| ACTL6A | 209.3807339 | 174.1597633 | -0.26572 | 0.002129 | 0.002129 | Downregulated |
| UMAD1 | 209.3761468 | 174.1656805 | -0.26564 | 0.002135 | 0.002135 | Downregulated |
| CFH | 209.3715596 | 174.1715976 | -0.26556 | 0.002142 | 0.002142 | Downregulated |
| GPM6B | 209.3669725 | 174.1775148 | -0.26548 | 0.002148 | 0.002148 | Downregulated |
| HSD11B1 | 208.2844037 | 173.2899408 | -0.26537 | 0.002274 | 0.002274 | Downregulated |
| SGIP1 | 209.3577982 | 174.1893491 | -0.26531 | 0.002162 | 0.002162 | Downregulated |
| SLC1A3 | 209.3577982 | 174.1893491 | -0.26531 | 0.002162 | 0.002162 | Downregulated |
| HERC3 | 209.3348624 | 174.2189349 | -0.26491 | 0.002195 | 0.002195 | Downregulated |
| FAM19A1 | 176.2752294 | 146.7100592 | -0.26486 | 0.007456 | 0.007456 | Downregulated |
| RHOH | 209.3302752 | 174.2248521 | -0.26483 | 0.002202 | 0.002202 | Downregulated |
| C8orf33 | 209.3119266 | 174.2485207 | -0.26451 | 0.002229 | 0.002229 | Downregulated |
| HECTD2 | 209.2981651 | 174.2662722 | -0.26427 | 0.002249 | 0.002249 | Downregulated |
| RNASE2 | 202.8761468 | 168.9349112 | -0.26413 | 0.003023 | 0.003023 | Downregulated |
| C6orf201 | 153.3256881 | 127.6745562 | -0.26413 | 0.014331 | 0.014331 | Downregulated |
| NAA16 | 209.2844037 | 174.2840237 | -0.26402 | 0.00227 | 0.00227 | Downregulated |
| GTF3C3 | 209.2844037 | 174.2840237 | -0.26402 | 0.00227 | 0.00227 | Downregulated |
| GDE1 | 209.2844037 | 174.2840237 | -0.26402 | 0.00227 | 0.00227 | Downregulated |
| AC087289.3 | 128.6146789 | 107.112426 | -0.26393 | 0.045877 | 0.045877 | Downregulated |
| MIGA1 | 209.2706422 | 174.3017751 | -0.26378 | 0.002291 | 0.002291 | Downregulated |
| RANBP1 | 209.2614679 | 174.3136095 | -0.26362 | 0.002305 | 0.002305 | Downregulated |
| FAM209A | 157.940367 | 131.5739645 | -0.26351 | 0.018486 | 0.018486 | Downregulated |
| NINL | 209.2477064 | 174.3313609 | -0.26338 | 0.002326 | 0.002326 | Downregulated |
| FAM76B | 209.2431193 | 174.3372781 | -0.2633 | 0.002333 | 0.002333 | Downregulated |
| CD83 | 209.2431193 | 174.3372781 | -0.2633 | 0.002333 | 0.002333 | Downregulated |
| DNAJC5B | 201.7706422 | 168.112426 | -0.26329 | 0.00328 | 0.00328 | Downregulated |
| C16orf86 | 209.2385321 | 174.3431953 | -0.26322 | 0.00234 | 0.00234 | Downregulated |
| TMUB2 | 208.206422 | 173.4852071 | -0.2632 | 0.002457 | 0.002457 | Downregulated |
| EDA | 209.2201835 | 174.3668639 | -0.2629 | 0.002369 | 0.002369 | Downregulated |
| UBA5 | 209.2201835 | 174.3668639 | -0.2629 | 0.002369 | 0.002369 | Downregulated |
| ZNF845 | 208.5412844 | 173.816568 | -0.26277 | 0.002373 | 0.002373 | Downregulated |
| CXCR3 | 209.2110092 | 174.3786982 | -0.26273 | 0.002384 | 0.002384 | Downregulated |
| S100A8 | 209.206422 | 174.3846154 | -0.26265 | 0.002391 | 0.002391 | Downregulated |
| PLEKHB2 | 209.1926606 | 174.4023669 | -0.26241 | 0.002413 | 0.002413 | Downregulated |
| FUT11 | 209.1926606 | 174.4023669 | -0.26241 | 0.002413 | 0.002413 | Downregulated |
| CEP44 | 209.1926606 | 174.4023669 | -0.26241 | 0.002413 | 0.002413 | Downregulated |
| PTP4A1 | 209.1834862 | 174.4142012 | -0.26225 | 0.002427 | 0.002427 | Downregulated |
| FKBP1C | 205.9587156 | 171.739645 | -0.26213 | 0.002839 | 0.002839 | Downregulated |
| MAP7D2 | 208.0917431 | 173.5384615 | -0.26196 | 0.002579 | 0.002579 | Downregulated |
| GIMAP8 | 209.1651376 | 174.4378698 | -0.26193 | 0.002457 | 0.002457 | Downregulated |
| IL6R | 209.1651376 | 174.4378698 | -0.26193 | 0.002457 | 0.002457 | Downregulated |
| VPS50 | 209.1651376 | 174.4378698 | -0.26193 | 0.002457 | 0.002457 | Downregulated |
| RBSN | 209.1422018 | 174.4674556 | -0.26153 | 0.002495 | 0.002495 | Downregulated |
| RHOT1 | 209.1422018 | 174.4674556 | -0.26153 | 0.002495 | 0.002495 | Downregulated |
| TMEM144 | 209.1422018 | 174.4674556 | -0.26153 | 0.002495 | 0.002495 | Downregulated |
| APBB1 | 209.1238532 | 174.4911243 | -0.2612 | 0.002525 | 0.002525 | Downregulated |
| NRP1 | 209.1238532 | 174.4911243 | -0.2612 | 0.002525 | 0.002525 | Downregulated |
| ZKSCAN4 | 209.1192661 | 174.4970414 | -0.26112 | 0.002533 | 0.002533 | Downregulated |
| TEK | 209.1055046 | 174.5147929 | -0.26088 | 0.002556 | 0.002556 | Downregulated |
| YPEL1 | 209.0917431 | 174.5325444 | -0.26064 | 0.002579 | 0.002579 | Downregulated |
| PICALM | 209.0917431 | 174.5325444 | -0.26064 | 0.002579 | 0.002579 | Downregulated |
| PXMP4 | 209.0825688 | 174.5443787 | -0.26048 | 0.002594 | 0.002594 | Downregulated |
| CWC25 | 209.0825688 | 174.5443787 | -0.26048 | 0.002594 | 0.002594 | Downregulated |
| ZNF85 | 206.9266055 | 172.7633136 | -0.26032 | 0.002882 | 0.002882 | Downregulated |
| ZNF669 | 209.0733945 | 174.556213 | -0.26032 | 0.00261 | 0.00261 | Downregulated |
| TMOD1 | 209.0642202 | 174.5680473 | -0.26016 | 0.002626 | 0.002626 | Downregulated |
| MS4A7 | 209.0642202 | 174.5680473 | -0.26016 | 0.002626 | 0.002626 | Downregulated |
| NPAT | 209.0642202 | 174.5680473 | -0.26016 | 0.002626 | 0.002626 | Downregulated |
| DENND5A | 209.059633 | 174.5739645 | -0.26008 | 0.002634 | 0.002634 | Downregulated |
| ZNF620 | 209.059633 | 174.5739645 | -0.26008 | 0.002634 | 0.002634 | Downregulated |
| FAM110D | 209.059633 | 174.5739645 | -0.26008 | 0.002634 | 0.002634 | Downregulated |
| ZSWIM3 | 209.0550459 | 174.5798817 | -0.26 | 0.002642 | 0.002642 | Downregulated |
| VIPR2 | 199.4816514 | 166.5857988 | -0.25999 | 0.004248 | 0.004248 | Downregulated |
| XPNPEP2 | 205.8348624 | 171.8994083 | -0.25992 | 0.003091 | 0.003091 | Downregulated |
| MRPL45 | 209.0458716 | 174.591716 | -0.25983 | 0.002658 | 0.002658 | Downregulated |
| INPP5J | 209.0412844 | 174.5976331 | -0.25975 | 0.002666 | 0.002666 | Downregulated |
| PARD6B | 209.0412844 | 174.5976331 | -0.25975 | 0.002666 | 0.002666 | Downregulated |
| ZNF70 | 209.0321101 | 174.6094675 | -0.25959 | 0.002682 | 0.002682 | Downregulated |
| GALNT16 | 205.8073394 | 171.9349112 | -0.25943 | 0.003119 | 0.003119 | Downregulated |
| PDK1 | 209.0229358 | 174.6213018 | -0.25943 | 0.002698 | 0.002698 | Downregulated |
| DOLPP1 | 209.0183486 | 174.6272189 | -0.25935 | 0.002706 | 0.002706 | Downregulated |
| S1PR1 | 209.0183486 | 174.6272189 | -0.25935 | 0.002706 | 0.002706 | Downregulated |
| FCAR | 175.0229358 | 146.2307692 | -0.2593 | 0.009607 | 0.009607 | Downregulated |
| PALB2 | 209.0137615 | 174.6331361 | -0.25927 | 0.002714 | 0.002714 | Downregulated |
| TM2D1 | 209.0091743 | 174.6390533 | -0.25919 | 0.002723 | 0.002723 | Downregulated |
| RFLNB | 209 | 174.6508876 | -0.25903 | 0.002739 | 0.002739 | Downregulated |
| LRRC70 | 184.9862385 | 154.591716 | -0.25895 | 0.007653 | 0.007653 | Downregulated |
| RBM23 | 208.9954128 | 174.6568047 | -0.25895 | 0.002747 | 0.002747 | Downregulated |
| ST14 | 208.9816514 | 174.6745562 | -0.25871 | 0.002772 | 0.002772 | Downregulated |
| GPC6 | 208.9770642 | 174.6804734 | -0.25863 | 0.00278 | 0.00278 | Downregulated |
| MSANTD4 | 208.9724771 | 174.6863905 | -0.25855 | 0.002789 | 0.002789 | Downregulated |
| VPS37D | 208.9724771 | 174.6863905 | -0.25855 | 0.002789 | 0.002789 | Downregulated |
| SPCS1 | 208.9724771 | 174.6863905 | -0.25855 | 0.002789 | 0.002789 | Downregulated |
| CCR1 | 208.9633028 | 174.6982249 | -0.25838 | 0.002806 | 0.002806 | Downregulated |
| BLOC1S5 | 208.9633028 | 174.6982249 | -0.25838 | 0.002806 | 0.002806 | Downregulated |
| TNFSF13B | 208.9633028 | 174.6982249 | -0.25838 | 0.002806 | 0.002806 | Downregulated |
| ZNF286B | 205.7477064 | 172.0118343 | -0.25837 | 0.003242 | 0.003242 | Downregulated |
| IL31RA | 129.2614679 | 108.0710059 | -0.25831 | 0.047236 | 0.047236 | Downregulated |
| SNX13 | 208.9587156 | 174.704142 | -0.2583 | 0.002814 | 0.002814 | Downregulated |
| LMBRD1 | 208.9587156 | 174.704142 | -0.2583 | 0.002814 | 0.002814 | Downregulated |
| ST7L | 208.9541284 | 174.7100592 | -0.25822 | 0.002822 | 0.002822 | Downregulated |
| CIPC | 208.9495413 | 174.7159763 | -0.25814 | 0.002831 | 0.002831 | Downregulated |
| KLHL6 | 208.9449541 | 174.7218935 | -0.25806 | 0.002839 | 0.002839 | Downregulated |
| NOD1 | 208.9266055 | 174.7455621 | -0.25774 | 0.002874 | 0.002874 | Downregulated |
| CNOT6L | 208.9266055 | 174.7455621 | -0.25774 | 0.002874 | 0.002874 | Downregulated |
| TIMMDC1 | 208.9266055 | 174.7455621 | -0.25774 | 0.002874 | 0.002874 | Downregulated |
| SNX9 | 208.9220183 | 174.7514793 | -0.25766 | 0.002882 | 0.002882 | Downregulated |
| DNAJC8 | 208.9220183 | 174.7514793 | -0.25766 | 0.002882 | 0.002882 | Downregulated |
| TEX261 | 208.9174312 | 174.7573964 | -0.25758 | 0.002891 | 0.002891 | Downregulated |
| TSPYL4 | 208.912844 | 174.7633136 | -0.2575 | 0.0029 | 0.0029 | Downregulated |
| PPEF1 | 196.2018349 | 164.1420118 | -0.25739 | 0.004973 | 0.004973 | Downregulated |
| MMADHC | 208.9036697 | 174.7751479 | -0.25734 | 0.002917 | 0.002917 | Downregulated |
| PDZD7 | 208.8990826 | 174.7810651 | -0.25726 | 0.002926 | 0.002926 | Downregulated |
| CARNS1 | 208.8990826 | 174.7810651 | -0.25726 | 0.002926 | 0.002926 | Downregulated |
| AQP9 | 208.8944954 | 174.7869822 | -0.25718 | 0.002934 | 0.002934 | Downregulated |
| CSF2RB | 208.8944954 | 174.7869822 | -0.25718 | 0.002934 | 0.002934 | Downregulated |
| FCGR2B | 208.8944954 | 174.7869822 | -0.25718 | 0.002934 | 0.002934 | Downregulated |
| EID2B | 205.6743119 | 172.1065089 | -0.25706 | 0.003429 | 0.003429 | Downregulated |
| MAP3K8 | 208.8853211 | 174.7988166 | -0.25702 | 0.002952 | 0.002952 | Downregulated |
| ADGRF5 | 208.8807339 | 174.8047337 | -0.25694 | 0.002961 | 0.002961 | Downregulated |
| PDLIM3 | 208.8761468 | 174.8106509 | -0.25685 | 0.00297 | 0.00297 | Downregulated |
| ASB7 | 208.8761468 | 174.8106509 | -0.25685 | 0.00297 | 0.00297 | Downregulated |
| RABL3 | 208.8761468 | 174.8106509 | -0.25685 | 0.00297 | 0.00297 | Downregulated |
| PHLPP2 | 208.8761468 | 174.8106509 | -0.25685 | 0.00297 | 0.00297 | Downregulated |
| HM13 | 208.8761468 | 174.8106509 | -0.25685 | 0.00297 | 0.00297 | Downregulated |
| ALKBH8 | 208.6100917 | 174.5976331 | -0.25677 | 0.002926 | 0.002926 | Downregulated |
| TLR2 | 208.8715596 | 174.816568 | -0.25677 | 0.002978 | 0.002978 | Downregulated |
| CCR8 | 192.0229358 | 160.7159763 | -0.25677 | 0.006386 | 0.006386 | Downregulated |
| USP46 | 208.8669725 | 174.8224852 | -0.25669 | 0.002987 | 0.002987 | Downregulated |
| VAMP3 | 208.8669725 | 174.8224852 | -0.25669 | 0.002987 | 0.002987 | Downregulated |
| CLIC2 | 208.8623853 | 174.8284024 | -0.25661 | 0.002996 | 0.002996 | Downregulated |
| PM20D1 | 136.0412844 | 113.8816568 | -0.25651 | 0.03834 | 0.03834 | Downregulated |
| NSL1 | 208.8394495 | 174.8579882 | -0.25621 | 0.003041 | 0.003041 | Downregulated |
| MATN4 | 175.8577982 | 147.2485207 | -0.25616 | 0.012785 | 0.012785 | Downregulated |
| SLC38A9 | 208.8348624 | 174.8639053 | -0.25613 | 0.00305 | 0.00305 | Downregulated |
| XXYLT1 | 208.8302752 | 174.8698225 | -0.25605 | 0.003059 | 0.003059 | Downregulated |
| PNRC1 | 208.8302752 | 174.8698225 | -0.25605 | 0.003059 | 0.003059 | Downregulated |
| ATP13A4 | 198.2155963 | 165.9881657 | -0.25599 | 0.004862 | 0.004862 | Downregulated |
| IL7R | 208.8211009 | 174.8816568 | -0.25589 | 0.003078 | 0.003078 | Downregulated |
| HIST1H1D | 167.0825688 | 139.9289941 | -0.25587 | 0.014008 | 0.014008 | Downregulated |
| PKHD1L1 | 162.3073394 | 135.9408284 | -0.25575 | 0.019674 | 0.019674 | Downregulated |
| ADGRL2 | 208.8119266 | 174.8934911 | -0.25573 | 0.003096 | 0.003096 | Downregulated |
| FGR | 208.8073394 | 174.8994083 | -0.25565 | 0.003105 | 0.003105 | Downregulated |
| RAB2A | 208.8027523 | 174.9053254 | -0.25557 | 0.003114 | 0.003114 | Downregulated |
| PYGO1 | 207.7247706 | 174.0118343 | -0.25549 | 0.003285 | 0.003285 | Downregulated |
| MEIG1 | 168.0275229 | 140.7573964 | -0.25549 | 0.014837 | 0.014837 | Downregulated |
| SLC38A6 | 208.7981651 | 174.9112426 | -0.25549 | 0.003124 | 0.003124 | Downregulated |
| GNS | 208.7981651 | 174.9112426 | -0.25549 | 0.003124 | 0.003124 | Downregulated |
| CALU | 208.793578 | 174.9171598 | -0.25541 | 0.003133 | 0.003133 | Downregulated |
| DPP4 | 208.793578 | 174.9171598 | -0.25541 | 0.003133 | 0.003133 | Downregulated |
| UHRF1BP1L | 208.793578 | 174.9171598 | -0.25541 | 0.003133 | 0.003133 | Downregulated |
| AP1S2 | 208.7844037 | 174.9289941 | -0.25524 | 0.003152 | 0.003152 | Downregulated |
| FBXO5 | 208.7798165 | 174.9349112 | -0.25516 | 0.003161 | 0.003161 | Downregulated |
| PADI4 | 155.7201835 | 130.4792899 | -0.25514 | 0.020801 | 0.020801 | Downregulated |
| PLEKHA3 | 208.4036697 | 174.6272189 | -0.2551 | 0.003082 | 0.003082 | Downregulated |
| MYLK3 | 180.7706422 | 151.4733728 | -0.2551 | 0.009605 | 0.009605 | Downregulated |
| ZFP64 | 208.7752294 | 174.9408284 | -0.25508 | 0.00317 | 0.00317 | Downregulated |
| CREM | 208.7614679 | 174.9585799 | -0.25484 | 0.003199 | 0.003199 | Downregulated |
| LETM2 | 208.7568807 | 174.964497 | -0.25476 | 0.003208 | 0.003208 | Downregulated |
| SV2B | 195.0091743 | 163.4674556 | -0.25454 | 0.006067 | 0.006067 | Downregulated |
| CRADD | 208.7385321 | 174.9881657 | -0.25444 | 0.003246 | 0.003246 | Downregulated |
| ACSM5 | 153.8394495 | 128.9704142 | -0.25439 | 0.02373 | 0.02373 | Downregulated |
| CDC123 | 208.733945 | 174.9940828 | -0.25436 | 0.003256 | 0.003256 | Downregulated |
| PARVB | 208.733945 | 174.9940828 | -0.25436 | 0.003256 | 0.003256 | Downregulated |
| MEOX1 | 208.733945 | 174.9940828 | -0.25436 | 0.003256 | 0.003256 | Downregulated |
| ATP1A3 | 208.7293578 | 175 | -0.25428 | 0.003266 | 0.003266 | Downregulated |
| F7 | 178.733945 | 149.8579882 | -0.25422 | 0.010958 | 0.010958 | Downregulated |
| PROK2 | 163.1834862 | 136.8284024 | -0.25413 | 0.015425 | 0.015425 | Downregulated |
| RARB | 208.7201835 | 175.0118343 | -0.25412 | 0.003285 | 0.003285 | Downregulated |
| SLC35B4 | 208.7155963 | 175.0177515 | -0.25404 | 0.003295 | 0.003295 | Downregulated |
| ECM2 | 208.7110092 | 175.0236686 | -0.25396 | 0.003305 | 0.003305 | Downregulated |
| SLC2A12 | 207.6376147 | 174.1242604 | -0.25395 | 0.003474 | 0.003474 | Downregulated |
| RIPOR3 | 208.706422 | 175.0295858 | -0.25388 | 0.003314 | 0.003314 | Downregulated |
| CCP110 | 208.7018349 | 175.035503 | -0.2538 | 0.003324 | 0.003324 | Downregulated |
| ZNF708 | 208.6926606 | 175.0473373 | -0.25363 | 0.003344 | 0.003344 | Downregulated |
| C1orf216 | 208.6926606 | 175.0473373 | -0.25363 | 0.003344 | 0.003344 | Downregulated |
| SASS6 | 208.6880734 | 175.0532544 | -0.25355 | 0.003354 | 0.003354 | Downregulated |
| DNAJA2 | 208.6743119 | 175.0710059 | -0.25331 | 0.003384 | 0.003384 | Downregulated |
| METTL16 | 208.6743119 | 175.0710059 | -0.25331 | 0.003384 | 0.003384 | Downregulated |
| ZRANB1 | 208.6743119 | 175.0710059 | -0.25331 | 0.003384 | 0.003384 | Downregulated |
| GPR63 | 208.6743119 | 175.0710059 | -0.25331 | 0.003384 | 0.003384 | Downregulated |
| PDE1A | 208.6651376 | 175.0828402 | -0.25315 | 0.003404 | 0.003404 | Downregulated |
| CASC3 | 208.6651376 | 175.0828402 | -0.25315 | 0.003404 | 0.003404 | Downregulated |
| CLC | 158.4266055 | 132.9349112 | -0.25309 | 0.015277 | 0.015277 | Downregulated |
| FAM217B | 208.646789 | 175.1065089 | -0.25283 | 0.003444 | 0.003444 | Downregulated |
| GNB5 | 208.646789 | 175.1065089 | -0.25283 | 0.003444 | 0.003444 | Downregulated |
| ZNF569 | 206.5 | 173.3136095 | -0.25276 | 0.003805 | 0.003805 | Downregulated |
| KLHDC7B | 208.6422018 | 175.112426 | -0.25275 | 0.003454 | 0.003454 | Downregulated |
| ZNF878 | 190.7798165 | 160.1301775 | -0.25266 | 0.007151 | 0.007151 | Downregulated |
| VCPKMT | 208.6330275 | 175.1242604 | -0.25259 | 0.003474 | 0.003474 | Downregulated |
| PTP4A2 | 208.6330275 | 175.1242604 | -0.25259 | 0.003474 | 0.003474 | Downregulated |
| TTC7B | 208.6284404 | 175.1301775 | -0.25251 | 0.003485 | 0.003485 | Downregulated |
| POMK | 208.6284404 | 175.1301775 | -0.25251 | 0.003485 | 0.003485 | Downregulated |
| SIGLEC10 | 208.6284404 | 175.1301775 | -0.25251 | 0.003485 | 0.003485 | Downregulated |
| ASNSD1 | 208.6238532 | 175.1360947 | -0.25243 | 0.003495 | 0.003495 | Downregulated |
| TNFAIP8L3 | 208.6192661 | 175.1420118 | -0.25235 | 0.003505 | 0.003505 | Downregulated |
| AL161911.1 | 208.6192661 | 175.1420118 | -0.25235 | 0.003505 | 0.003505 | Downregulated |
| ATP6V0A1 | 208.6100917 | 175.1538462 | -0.25219 | 0.003526 | 0.003526 | Downregulated |
| CCDC15 | 208.6100917 | 175.1538462 | -0.25219 | 0.003526 | 0.003526 | Downregulated |
| BIN2 | 208.6055046 | 175.1597633 | -0.25211 | 0.003536 | 0.003536 | Downregulated |
| SLC25A17 | 208.6055046 | 175.1597633 | -0.25211 | 0.003536 | 0.003536 | Downregulated |
| SLC31A2 | 207.5321101 | 174.260355 | -0.25209 | 0.003706 | 0.003706 | Downregulated |
| CCDC178 | 119.9311927 | 100.7100592 | -0.252 | 0.048697 | 0.048697 | Downregulated |
| TBC1D9 | 208.587156 | 175.183432 | -0.25178 | 0.003578 | 0.003578 | Downregulated |
| BEST1 | 208.5779817 | 175.1952663 | -0.25162 | 0.003599 | 0.003599 | Downregulated |
| TNFSF18 | 181.5917431 | 152.5443787 | -0.25147 | 0.01121 | 0.01121 | Downregulated |
| USP2 | 208.5642202 | 175.2130178 | -0.25138 | 0.003631 | 0.003631 | Downregulated |
| AZI2 | 208.5642202 | 175.2130178 | -0.25138 | 0.003631 | 0.003631 | Downregulated |
| SLFN12L | 199.0091743 | 167.1952663 | -0.2513 | 0.005415 | 0.005415 | Downregulated |
| CNR2 | 169.7614679 | 142.6331361 | -0.2512 | 0.014146 | 0.014146 | Downregulated |
| SIRPB1 | 203.2201835 | 170.7455621 | -0.2512 | 0.004579 | 0.004579 | Downregulated |
| RNF121 | 208.5504587 | 175.2307692 | -0.25114 | 0.003663 | 0.003663 | Downregulated |
| NAALAD2 | 202.1559633 | 169.8639053 | -0.25109 | 0.004821 | 0.004821 | Downregulated |
| IDO1 | 208.5458716 | 175.2366864 | -0.25106 | 0.003674 | 0.003674 | Downregulated |
| PYGO2 | 208.5412844 | 175.2426036 | -0.25098 | 0.003684 | 0.003684 | Downregulated |
| VPS13B | 208.5366972 | 175.2485207 | -0.2509 | 0.003695 | 0.003695 | Downregulated |
| GPLD1 | 207.4633028 | 174.3491124 | -0.25088 | 0.003878 | 0.003878 | Downregulated |
| MTRF1 | 208.5321101 | 175.2544379 | -0.25082 | 0.003706 | 0.003706 | Downregulated |
| MFSD14A | 208.5321101 | 175.2544379 | -0.25082 | 0.003706 | 0.003706 | Downregulated |
| SCIMP | 208.5229358 | 175.2662722 | -0.25066 | 0.003728 | 0.003728 | Downregulated |
| HCAR2 | 200.0229358 | 168.1242604 | -0.25064 | 0.0054 | 0.0054 | Downregulated |
| ARCN1 | 208.5137615 | 175.2781065 | -0.2505 | 0.00375 | 0.00375 | Downregulated |
| TLR1 | 208.5091743 | 175.2840237 | -0.25042 | 0.003761 | 0.003761 | Downregulated |
| WDR24 | 208.5091743 | 175.2840237 | -0.25042 | 0.003761 | 0.003761 | Downregulated |
| SMARCD3 | 208.5045872 | 175.2899408 | -0.25034 | 0.003772 | 0.003772 | Downregulated |
| ENTPD1 | 208.5045872 | 175.2899408 | -0.25034 | 0.003772 | 0.003772 | Downregulated |
| BEX4 | 208.5 | 175.295858 | -0.25026 | 0.003783 | 0.003783 | Downregulated |
| MYNN | 208.4954128 | 175.3017751 | -0.25018 | 0.003794 | 0.003794 | Downregulated |
| SLC9A9 | 208.4908257 | 175.3076923 | -0.25009 | 0.003805 | 0.003805 | Downregulated |
| LRRC8C | 208.4908257 | 175.3076923 | -0.25009 | 0.003805 | 0.003805 | Downregulated |
| SLF2 | 208.4908257 | 175.3076923 | -0.25009 | 0.003805 | 0.003805 | Downregulated |
| VEGFC | 208.4862385 | 175.3136095 | -0.25001 | 0.003816 | 0.003816 | Downregulated |
| RAB3IL1 | 208.4724771 | 175.3313609 | -0.24977 | 0.003849 | 0.003849 | Downregulated |
| HSPB11 | 208.4724771 | 175.3313609 | -0.24977 | 0.003849 | 0.003849 | Downregulated |
| RAB8B | 208.4678899 | 175.3372781 | -0.24969 | 0.003861 | 0.003861 | Downregulated |
| MYCT1 | 208.4587156 | 175.3491124 | -0.24953 | 0.003883 | 0.003883 | Downregulated |
| ZNF674 | 208.4541284 | 175.3550296 | -0.24945 | 0.003895 | 0.003895 | Downregulated |
| EAF2 | 208.4495413 | 175.3609467 | -0.24937 | 0.003906 | 0.003906 | Downregulated |
| FAM46C | 208.4449541 | 175.3668639 | -0.24929 | 0.003917 | 0.003917 | Downregulated |
| GPAM | 208.4449541 | 175.3668639 | -0.24929 | 0.003917 | 0.003917 | Downregulated |
| PRDM16 | 208.440367 | 175.3727811 | -0.24921 | 0.003929 | 0.003929 | Downregulated |
| ADAMTSL1 | 208.4357798 | 175.3786982 | -0.24913 | 0.00394 | 0.00394 | Downregulated |
| SIK3 | 208.4357798 | 175.3786982 | -0.24913 | 0.00394 | 0.00394 | Downregulated |
| CCNJ | 208.4311927 | 175.3846154 | -0.24905 | 0.003952 | 0.003952 | Downregulated |
| PPFIA1 | 208.4311927 | 175.3846154 | -0.24905 | 0.003952 | 0.003952 | Downregulated |
| IFNG | 157.3165138 | 132.3786982 | -0.249 | 0.028949 | 0.028949 | Downregulated |
| TMEM99 | 208.4220183 | 175.3964497 | -0.24889 | 0.003975 | 0.003975 | Downregulated |
| NPTX2 | 208.4220183 | 175.3964497 | -0.24889 | 0.003975 | 0.003975 | Downregulated |
| NCF2 | 208.4220183 | 175.3964497 | -0.24889 | 0.003975 | 0.003975 | Downregulated |
| FAM20A | 208.4220183 | 175.3964497 | -0.24889 | 0.003975 | 0.003975 | Downregulated |
| HACD3 | 208.4174312 | 175.4023669 | -0.24881 | 0.003986 | 0.003986 | Downregulated |
| SGPL1 | 208.4174312 | 175.4023669 | -0.24881 | 0.003986 | 0.003986 | Downregulated |
| DCLRE1A | 208.4174312 | 175.4023669 | -0.24881 | 0.003986 | 0.003986 | Downregulated |
| FPR1 | 208.4174312 | 175.4023669 | -0.24881 | 0.003986 | 0.003986 | Downregulated |
| KIAA1257 | 208.412844 | 175.408284 | -0.24873 | 0.003998 | 0.003998 | Downregulated |
| GTPBP8 | 208.4082569 | 175.4142012 | -0.24865 | 0.00401 | 0.00401 | Downregulated |
| SLC35G1 | 208.4082569 | 175.4142012 | -0.24865 | 0.00401 | 0.00401 | Downregulated |
| F10 | 208.4036697 | 175.4201183 | -0.24857 | 0.004021 | 0.004021 | Downregulated |
| PDGFRA | 208.4036697 | 175.4201183 | -0.24857 | 0.004021 | 0.004021 | Downregulated |
| ZNF483 | 171.5779817 | 144.4260355 | -0.24853 | 0.018785 | 0.018785 | Downregulated |
| NXPE3 | 208.3944954 | 175.4319527 | -0.24841 | 0.004045 | 0.004045 | Downregulated |
| ZNF528 | 208.3944954 | 175.4319527 | -0.24841 | 0.004045 | 0.004045 | Downregulated |
| GABPB1 | 208.3944954 | 175.4319527 | -0.24841 | 0.004045 | 0.004045 | Downregulated |
| CD3G | 208.3853211 | 175.443787 | -0.24824 | 0.004068 | 0.004068 | Downregulated |
| GPR176 | 208.3853211 | 175.443787 | -0.24824 | 0.004068 | 0.004068 | Downregulated |
| CR1 | 199.8853211 | 168.3017751 | -0.24812 | 0.00581 | 0.00581 | Downregulated |
| NBR1 | 208.3715596 | 175.4615385 | -0.248 | 0.004104 | 0.004104 | Downregulated |
| DBR1 | 208.3715596 | 175.4615385 | -0.248 | 0.004104 | 0.004104 | Downregulated |
| PBX3 | 208.3669725 | 175.4674556 | -0.24792 | 0.004116 | 0.004116 | Downregulated |
| CD226 | 208.3623853 | 175.4733728 | -0.24784 | 0.004128 | 0.004128 | Downregulated |
| FLT1 | 208.353211 | 175.4852071 | -0.24768 | 0.004151 | 0.004151 | Downregulated |
| MBTPS1 | 207.6559633 | 174.8994083 | -0.24767 | 0.004188 | 0.004188 | Downregulated |
| GLT1D1 | 191.5412844 | 161.3372781 | -0.24758 | 0.007472 | 0.007472 | Downregulated |
| OSMR | 208.3440367 | 175.4970414 | -0.24752 | 0.004176 | 0.004176 | Downregulated |
| SLC2A6 | 208.3440367 | 175.4970414 | -0.24752 | 0.004176 | 0.004176 | Downregulated |
| KCNK12 | 192.5642202 | 162.2130178 | -0.24745 | 0.007483 | 0.007483 | Downregulated |
| ANGPT2 | 208.3394495 | 175.5029586 | -0.24744 | 0.004188 | 0.004188 | Downregulated |
| MED24 | 208.3302752 | 175.5147929 | -0.24728 | 0.004212 | 0.004212 | Downregulated |
| RLF | 208.3302752 | 175.5147929 | -0.24728 | 0.004212 | 0.004212 | Downregulated |
| CRBN | 208.3256881 | 175.5207101 | -0.2472 | 0.004224 | 0.004224 | Downregulated |
| TLR7 | 204.0550459 | 171.9289941 | -0.24715 | 0.005089 | 0.005089 | Downregulated |
| HIBADH | 208.3211009 | 175.5266272 | -0.24712 | 0.004236 | 0.004236 | Downregulated |
| NANOS1 | 206.1788991 | 173.7278107 | -0.24707 | 0.004645 | 0.004645 | Downregulated |
| TCP11L2 | 208.3119266 | 175.5384615 | -0.24696 | 0.004261 | 0.004261 | Downregulated |
| DHRS13 | 208.3119266 | 175.5384615 | -0.24696 | 0.004261 | 0.004261 | Downregulated |
| CRCP | 208.3073394 | 175.5443787 | -0.24688 | 0.004273 | 0.004273 | Downregulated |
| CNOT9 | 208.3073394 | 175.5443787 | -0.24688 | 0.004273 | 0.004273 | Downregulated |
| IL13RA2 | 202.9770642 | 171.0591716 | -0.24682 | 0.005325 | 0.005325 | Downregulated |
| SLBP | 208.3027523 | 175.5502959 | -0.2468 | 0.004286 | 0.004286 | Downregulated |
| TBPL1 | 208.3027523 | 175.5502959 | -0.2468 | 0.004286 | 0.004286 | Downregulated |
| MTRF1L | 208.2981651 | 175.556213 | -0.24672 | 0.004298 | 0.004298 | Downregulated |
| KATNAL1 | 208.2981651 | 175.556213 | -0.24672 | 0.004298 | 0.004298 | Downregulated |
| CMKLR1 | 208.293578 | 175.5621302 | -0.24664 | 0.00431 | 0.00431 | Downregulated |
| MAGEF1 | 208.293578 | 175.5621302 | -0.24664 | 0.00431 | 0.00431 | Downregulated |
| CSPG5 | 208.293578 | 175.5621302 | -0.24664 | 0.00431 | 0.00431 | Downregulated |
| STYX | 208.2798165 | 175.5798817 | -0.2464 | 0.004348 | 0.004348 | Downregulated |
| CD36 | 208.2752294 | 175.5857988 | -0.24632 | 0.00436 | 0.00436 | Downregulated |
| CCSAP | 208.2706422 | 175.591716 | -0.24623 | 0.004373 | 0.004373 | Downregulated |
| SCNN1G | 131.3715596 | 110.7633136 | -0.24617 | 0.030784 | 0.030784 | Downregulated |
| DNAJB4 | 208.266055 | 175.5976331 | -0.24615 | 0.004386 | 0.004386 | Downregulated |
| CREB1 | 208.266055 | 175.5976331 | -0.24615 | 0.004386 | 0.004386 | Downregulated |
| HIST1H2BO | 162.8256881 | 137.2899408 | -0.2461 | 0.019801 | 0.019801 | Downregulated |
| MAF | 208.2568807 | 175.6094675 | -0.24599 | 0.004411 | 0.004411 | Downregulated |
| DBT | 208.2568807 | 175.6094675 | -0.24599 | 0.004411 | 0.004411 | Downregulated |
| IGFLR1 | 208.2568807 | 175.6094675 | -0.24599 | 0.004411 | 0.004411 | Downregulated |
| ICAM3 | 207.1834862 | 174.7100592 | -0.24595 | 0.004625 | 0.004625 | Downregulated |
| EFTUD2 | 208.2522936 | 175.6153846 | -0.24591 | 0.004424 | 0.004424 | Downregulated |
| CHN1 | 208.2477064 | 175.6213018 | -0.24583 | 0.004436 | 0.004436 | Downregulated |
| CD59 | 208.2477064 | 175.6213018 | -0.24583 | 0.004436 | 0.004436 | Downregulated |
| SESTD1 | 208.233945 | 175.6390533 | -0.24559 | 0.004475 | 0.004475 | Downregulated |
| GRIN3A | 208.233945 | 175.6390533 | -0.24559 | 0.004475 | 0.004475 | Downregulated |
| SIKE1 | 208.2247706 | 175.6508876 | -0.24543 | 0.004501 | 0.004501 | Downregulated |
| ADGRE2 | 208.2247706 | 175.6508876 | -0.24543 | 0.004501 | 0.004501 | Downregulated |
| UBXN2B | 208.2247706 | 175.6508876 | -0.24543 | 0.004501 | 0.004501 | Downregulated |
| SLC12A6 | 208.2247706 | 175.6508876 | -0.24543 | 0.004501 | 0.004501 | Downregulated |
| ZNF433 | 208.2155963 | 175.6627219 | -0.24527 | 0.004527 | 0.004527 | Downregulated |
| DZIP1L | 208.2110092 | 175.6686391 | -0.24519 | 0.00454 | 0.00454 | Downregulated |
| UBR1 | 208.206422 | 175.6745562 | -0.24511 | 0.004553 | 0.004553 | Downregulated |
| CMTM8 | 208.206422 | 175.6745562 | -0.24511 | 0.004553 | 0.004553 | Downregulated |
| CENPQ | 208.206422 | 175.6745562 | -0.24511 | 0.004553 | 0.004553 | Downregulated |
| IKBKB | 208.2018349 | 175.6804734 | -0.24503 | 0.004566 | 0.004566 | Downregulated |
| ZNF791 | 208.2018349 | 175.6804734 | -0.24503 | 0.004566 | 0.004566 | Downregulated |
| RANBP3L | 139.0917431 | 117.3668639 | -0.24501 | 0.031775 | 0.031775 | Downregulated |
| ZFP30 | 208.1972477 | 175.6863905 | -0.24495 | 0.004579 | 0.004579 | Downregulated |
| NDRG2 | 208.1972477 | 175.6863905 | -0.24495 | 0.004579 | 0.004579 | Downregulated |
| TMEM206 | 208.1880734 | 175.6982249 | -0.24479 | 0.004605 | 0.004605 | Downregulated |
| PHTF2 | 208.1880734 | 175.6982249 | -0.24479 | 0.004605 | 0.004605 | Downregulated |
| TNNC1 | 207.1146789 | 174.7988166 | -0.24473 | 0.004821 | 0.004821 | Downregulated |
| C2orf42 | 208.1834862 | 175.704142 | -0.24471 | 0.004618 | 0.004618 | Downregulated |
| PEX26 | 208.1788991 | 175.7100592 | -0.24463 | 0.004632 | 0.004632 | Downregulated |
| MFSD14C | 208.1743119 | 175.7159763 | -0.24455 | 0.004645 | 0.004645 | Downregulated |
| CHN2 | 208.1743119 | 175.7159763 | -0.24455 | 0.004645 | 0.004645 | Downregulated |
| FOLH1 | 204.9678899 | 173.0177515 | -0.24448 | 0.005325 | 0.005325 | Downregulated |
| PTPRR | 208.1697248 | 175.7218935 | -0.24447 | 0.004658 | 0.004658 | Downregulated |
| GOLGA7 | 208.1651376 | 175.7278107 | -0.24439 | 0.004672 | 0.004672 | Downregulated |
| DBNL | 208.1376147 | 175.7633136 | -0.2439 | 0.004752 | 0.004752 | Downregulated |
| TYRP1 | 196.5183486 | 165.9526627 | -0.24389 | 0.007484 | 0.007484 | Downregulated |
| SIRPB2 | 208.1330275 | 175.7692308 | -0.24382 | 0.004766 | 0.004766 | Downregulated |
| THAP5 | 208.1330275 | 175.7692308 | -0.24382 | 0.004766 | 0.004766 | Downregulated |
| NME6 | 208.1284404 | 175.7751479 | -0.24374 | 0.00478 | 0.00478 | Downregulated |
| KLHL29 | 208.1284404 | 175.7751479 | -0.24374 | 0.00478 | 0.00478 | Downregulated |
| FLI1 | 208.1284404 | 175.7751479 | -0.24374 | 0.00478 | 0.00478 | Downregulated |
| DPYD | 208.1284404 | 175.7751479 | -0.24374 | 0.00478 | 0.00478 | Downregulated |
| FGF12 | 201.7431193 | 170.3964497 | -0.24362 | 0.00611 | 0.00611 | Downregulated |
| RAD9B | 202.7981651 | 171.2899408 | -0.2436 | 0.005983 | 0.005983 | Downregulated |
| ABCA1 | 208.1192661 | 175.7869822 | -0.24358 | 0.004807 | 0.004807 | Downregulated |
| ZNHIT3 | 208.1100917 | 175.7988166 | -0.24342 | 0.004835 | 0.004835 | Downregulated |
| RBMS3 | 208.1055046 | 175.8047337 | -0.24334 | 0.004848 | 0.004848 | Downregulated |
| DDX3X | 208.1009174 | 175.8106509 | -0.24326 | 0.004862 | 0.004862 | Downregulated |
| HACD4 | 208.0917431 | 175.8224852 | -0.2431 | 0.00489 | 0.00489 | Downregulated |
| PDP2 | 207.6330275 | 175.443787 | -0.24303 | 0.004766 | 0.004766 | Downregulated |
| GALNT10 | 208.087156 | 175.8284024 | -0.24302 | 0.004904 | 0.004904 | Downregulated |
| WDR43 | 208.087156 | 175.8284024 | -0.24302 | 0.004904 | 0.004904 | Downregulated |
| C6orf52 | 198.5550459 | 167.7810651 | -0.24296 | 0.007403 | 0.007403 | Downregulated |
| ZNF253 | 208.0825688 | 175.8343195 | -0.24294 | 0.004918 | 0.004918 | Downregulated |
| VMP1 | 208.0825688 | 175.8343195 | -0.24294 | 0.004918 | 0.004918 | Downregulated |
| BCAP29 | 208.0825688 | 175.8343195 | -0.24294 | 0.004918 | 0.004918 | Downregulated |
| MYO5A | 208.0779817 | 175.8402367 | -0.24286 | 0.004932 | 0.004932 | Downregulated |
| PROCR | 207.3302752 | 175.2130178 | -0.24282 | 0.004946 | 0.004946 | Downregulated |
| PRR16 | 208.0733945 | 175.8461538 | -0.24278 | 0.004946 | 0.004946 | Downregulated |
| C1D | 208.0688073 | 175.852071 | -0.2427 | 0.00496 | 0.00496 | Downregulated |
| BARD1 | 208.0688073 | 175.852071 | -0.2427 | 0.00496 | 0.00496 | Downregulated |
| CD47 | 208.0688073 | 175.852071 | -0.2427 | 0.00496 | 0.00496 | Downregulated |
| CAPZA1 | 208.0642202 | 175.8579882 | -0.24262 | 0.004974 | 0.004974 | Downregulated |
| IPO5 | 208.0642202 | 175.8579882 | -0.24262 | 0.004974 | 0.004974 | Downregulated |
| CAMTA1 | 208.059633 | 175.8639053 | -0.24254 | 0.004988 | 0.004988 | Downregulated |
| TM4SF18 | 208.059633 | 175.8639053 | -0.24254 | 0.004988 | 0.004988 | Downregulated |
| EXTL2 | 208.059633 | 175.8639053 | -0.24254 | 0.004988 | 0.004988 | Downregulated |
| MYO7A | 208.059633 | 175.8639053 | -0.24254 | 0.004988 | 0.004988 | Downregulated |
| PCDH17 | 208.059633 | 175.8639053 | -0.24254 | 0.004988 | 0.004988 | Downregulated |
| MEIOC | 201.6743119 | 170.4852071 | -0.24238 | 0.006477 | 0.006477 | Downregulated |
| EVI2B | 208.0412844 | 175.887574 | -0.24222 | 0.005046 | 0.005046 | Downregulated |
| ZNF506 | 208.0366972 | 175.8934911 | -0.24214 | 0.00506 | 0.00506 | Downregulated |
| DYNC2H1 | 208.0366972 | 175.8934911 | -0.24214 | 0.00506 | 0.00506 | Downregulated |
| POLR2K | 208.0321101 | 175.8994083 | -0.24206 | 0.005074 | 0.005074 | Downregulated |
| CFAP54 | 181.1238532 | 153.147929 | -0.24205 | 0.014107 | 0.014107 | Downregulated |
| ZBTB25 | 208.0275229 | 175.9053254 | -0.24198 | 0.005089 | 0.005089 | Downregulated |
| ARMCX1 | 208.0229358 | 175.9112426 | -0.24189 | 0.005103 | 0.005103 | Downregulated |
| PALMD | 208.0229358 | 175.9112426 | -0.24189 | 0.005103 | 0.005103 | Downregulated |
| COPB1 | 208.0183486 | 175.9171598 | -0.24181 | 0.005118 | 0.005118 | Downregulated |
| ISPD | 208.0137615 | 175.9230769 | -0.24173 | 0.005132 | 0.005132 | Downregulated |
| POLR3F | 208.0091743 | 175.9289941 | -0.24165 | 0.005147 | 0.005147 | Downregulated |
| WNT3 | 208.0091743 | 175.9289941 | -0.24165 | 0.005147 | 0.005147 | Downregulated |
| PIGW | 208.0091743 | 175.9289941 | -0.24165 | 0.005147 | 0.005147 | Downregulated |
| CPNE8 | 207.9954128 | 175.9467456 | -0.24141 | 0.005191 | 0.005191 | Downregulated |
| CERKL | 207.9908257 | 175.9526627 | -0.24133 | 0.005205 | 0.005205 | Downregulated |
| PIK3CA | 207.9862385 | 175.9585799 | -0.24125 | 0.00522 | 0.00522 | Downregulated |
| IFIT2 | 207.9770642 | 175.9704142 | -0.24109 | 0.00525 | 0.00525 | Downregulated |
| PIAS1 | 207.9724771 | 175.9763314 | -0.24101 | 0.005265 | 0.005265 | Downregulated |
| RNF13 | 207.9587156 | 175.9940828 | -0.24077 | 0.00531 | 0.00531 | Downregulated |
| SUCNR1 | 202.6376147 | 171.4970414 | -0.24072 | 0.006687 | 0.006687 | Downregulated |
| ALG9 | 207.9541284 | 176 | -0.24069 | 0.005325 | 0.005325 | Downregulated |
| CD84 | 207.9541284 | 176 | -0.24069 | 0.005325 | 0.005325 | Downregulated |
| PDE6G | 205.8165138 | 174.1952663 | -0.24065 | 0.00581 | 0.00581 | Downregulated |
| CYP17A1 | 132.0229358 | 111.739645 | -0.24065 | 0.049914 | 0.049914 | Downregulated |
| CHST12 | 207.9495413 | 176.0059172 | -0.24061 | 0.00534 | 0.00534 | Downregulated |
| MTF2 | 207.9495413 | 176.0059172 | -0.24061 | 0.00534 | 0.00534 | Downregulated |
| SLC9B1 | 203.6880734 | 172.4023669 | -0.24058 | 0.006335 | 0.006335 | Downregulated |
| FAM155B | 176.0917431 | 149.0473373 | -0.24056 | 0.0183 | 0.0183 | Downregulated |
| CTNS | 207.940367 | 176.0177515 | -0.24045 | 0.00537 | 0.00537 | Downregulated |
| WNK4 | 207.940367 | 176.0177515 | -0.24045 | 0.00537 | 0.00537 | Downregulated |
| ASTE1 | 207.940367 | 176.0177515 | -0.24045 | 0.00537 | 0.00537 | Downregulated |
| CD40 | 207.9357798 | 176.0236686 | -0.24037 | 0.005385 | 0.005385 | Downregulated |
| PPP1R21 | 207.9311927 | 176.0295858 | -0.24029 | 0.0054 | 0.0054 | Downregulated |
| CD80 | 202.6055046 | 171.5384615 | -0.24014 | 0.006724 | 0.006724 | Downregulated |
| IL11 | 206.853211 | 175.1360947 | -0.24013 | 0.005673 | 0.005673 | Downregulated |
| DUSP15 | 207.9220183 | 176.0414201 | -0.24013 | 0.005431 | 0.005431 | Downregulated |
| CCDC141 | 176.0688073 | 149.0769231 | -0.24008 | 0.018527 | 0.018527 | Downregulated |
| ADGRG3 | 207.9174312 | 176.0473373 | -0.24005 | 0.005446 | 0.005446 | Downregulated |
| FANCB | 207.912844 | 176.0532544 | -0.23997 | 0.005462 | 0.005462 | Downregulated |
| CLIP4 | 207.9082569 | 176.0591716 | -0.23989 | 0.005477 | 0.005477 | Downregulated |
| UBXN2A | 207.9036697 | 176.0650888 | -0.23981 | 0.005493 | 0.005493 | Downregulated |
| ZGRF1 | 207.8990826 | 176.0710059 | -0.23973 | 0.005508 | 0.005508 | Downregulated |
| ZNF490 | 207.8990826 | 176.0710059 | -0.23973 | 0.005508 | 0.005508 | Downregulated |
| ANKRD49 | 207.8944954 | 176.0769231 | -0.23965 | 0.005524 | 0.005524 | Downregulated |
| AFG1L | 207.8944954 | 176.0769231 | -0.23965 | 0.005524 | 0.005524 | Downregulated |
| PELI3 | 207.8899083 | 176.0828402 | -0.23957 | 0.005539 | 0.005539 | Downregulated |
| NEK1 | 207.8853211 | 176.0887574 | -0.23949 | 0.005555 | 0.005555 | Downregulated |
| CDS2 | 207.8853211 | 176.0887574 | -0.23949 | 0.005555 | 0.005555 | Downregulated |
| BATF3 | 207.8807339 | 176.0946746 | -0.2394 | 0.005571 | 0.005571 | Downregulated |
| EGFLAM | 207.8761468 | 176.1005917 | -0.23932 | 0.005586 | 0.005586 | Downregulated |
| ST3GAL6 | 207.8715596 | 176.1065089 | -0.23924 | 0.005602 | 0.005602 | Downregulated |
| RAB33A | 207.8715596 | 176.1065089 | -0.23924 | 0.005602 | 0.005602 | Downregulated |
| RNF114 | 207.8715596 | 176.1065089 | -0.23924 | 0.005602 | 0.005602 | Downregulated |
| AC003002.1 | 207.8669725 | 176.112426 | -0.23916 | 0.005618 | 0.005618 | Downregulated |
| ADAMTS3 | 203.6055046 | 172.5088757 | -0.23911 | 0.006734 | 0.006734 | Downregulated |
| ZEB2 | 207.5275229 | 175.8343195 | -0.23909 | 0.005618 | 0.005618 | Downregulated |
| CASP8AP2 | 207.8623853 | 176.1183432 | -0.23908 | 0.005634 | 0.005634 | Downregulated |
| TDRKH | 207.8623853 | 176.1183432 | -0.23908 | 0.005634 | 0.005634 | Downregulated |
| SH2D1A | 205.7247706 | 174.3136095 | -0.23903 | 0.006161 | 0.006161 | Downregulated |
| NUP35 | 207.8577982 | 176.1242604 | -0.239 | 0.005649 | 0.005649 | Downregulated |
| LACTB2 | 207.8577982 | 176.1242604 | -0.239 | 0.005649 | 0.005649 | Downregulated |
| LYSMD2 | 207.853211 | 176.1301775 | -0.23892 | 0.005665 | 0.005665 | Downregulated |
| TSPYL1 | 207.853211 | 176.1301775 | -0.23892 | 0.005665 | 0.005665 | Downregulated |
| OCLN | 207.853211 | 176.1301775 | -0.23892 | 0.005665 | 0.005665 | Downregulated |
| LYVE1 | 207.8486239 | 176.1360947 | -0.23884 | 0.005681 | 0.005681 | Downregulated |
| PTGS2 | 207.8486239 | 176.1360947 | -0.23884 | 0.005681 | 0.005681 | Downregulated |
| TAGAP | 207.8440367 | 176.1420118 | -0.23876 | 0.005697 | 0.005697 | Downregulated |
| NLRC4 | 207.8440367 | 176.1420118 | -0.23876 | 0.005697 | 0.005697 | Downregulated |
| C10orf128 | 207.8348624 | 176.1538462 | -0.2386 | 0.005729 | 0.005729 | Downregulated |
| GABARAPL2 | 207.8348624 | 176.1538462 | -0.2386 | 0.005729 | 0.005729 | Downregulated |
| SERPINA10 | 170.1376147 | 144.2130178 | -0.2385 | 0.018596 | 0.018596 | Downregulated |
| DNALI1 | 207.8256881 | 176.1656805 | -0.23844 | 0.005762 | 0.005762 | Downregulated |
| S1PR3 | 207.8211009 | 176.1715976 | -0.23836 | 0.005778 | 0.005778 | Downregulated |
| FYB | 207.8119266 | 176.183432 | -0.2382 | 0.00581 | 0.00581 | Downregulated |
| HS6ST2 | 206.7431193 | 175.2781065 | -0.23819 | 0.006059 | 0.006059 | Downregulated |
| LAX1 | 206.7431193 | 175.2781065 | -0.23819 | 0.006076 | 0.006076 | Downregulated |
| EPB41L5 | 207.8027523 | 176.1952663 | -0.23804 | 0.005843 | 0.005843 | Downregulated |
| TTC36 | 153.146789 | 129.8639053 | -0.23791 | 0.038224 | 0.038224 | Downregulated |
| DYNC1LI1 | 207.793578 | 176.2071006 | -0.23788 | 0.005876 | 0.005876 | Downregulated |
| TMEM39A | 207.3761468 | 175.8757396 | -0.23769 | 0.005745 | 0.005745 | Downregulated |
| FADS1 | 207.7798165 | 176.2248521 | -0.23764 | 0.005925 | 0.005925 | Downregulated |
| LEXM | 178.9082569 | 151.7514793 | -0.23751 | 0.017037 | 0.017037 | Downregulated |
| DCLK3 | 206.4082569 | 175.0828402 | -0.23746 | 0.006076 | 0.006076 | Downregulated |
| GTF2H1 | 207.7614679 | 176.2485207 | -0.23732 | 0.005992 | 0.005992 | Downregulated |
| ANKRD46 | 207.7614679 | 176.2485207 | -0.23732 | 0.005992 | 0.005992 | Downregulated |
| RASL10A | 205.6238532 | 174.443787 | -0.23725 | 0.006532 | 0.006532 | Downregulated |
| CCR2 | 205.6238532 | 174.443787 | -0.23725 | 0.006568 | 0.006568 | Downregulated |
| RASGEF1B | 207.7568807 | 176.2544379 | -0.23724 | 0.006009 | 0.006009 | Downregulated |
| DHX57 | 207.7568807 | 176.2544379 | -0.23724 | 0.006009 | 0.006009 | Downregulated |
| CCR4 | 207.7522936 | 176.260355 | -0.23716 | 0.006025 | 0.006025 | Downregulated |
| SERPINB1 | 207.7477064 | 176.2662722 | -0.23708 | 0.006042 | 0.006042 | Downregulated |
| DNAH17 | 207.7385321 | 176.2781065 | -0.23692 | 0.006076 | 0.006076 | Downregulated |
| CRKL | 207.2568807 | 175.8698225 | -0.23691 | 0.005975 | 0.005975 | Downregulated |
| EOGT | 207.733945 | 176.2840237 | -0.23684 | 0.006093 | 0.006093 | Downregulated |
| KLHDC2 | 207.733945 | 176.2840237 | -0.23684 | 0.006093 | 0.006093 | Downregulated |
| ITGB1BP1 | 207.7247706 | 176.295858 | -0.23667 | 0.006127 | 0.006127 | Downregulated |
| STIL | 207.7247706 | 176.295858 | -0.23667 | 0.006127 | 0.006127 | Downregulated |
| HIST1H2BF | 170.0504587 | 144.3254438 | -0.23664 | 0.021324 | 0.021324 | Downregulated |
| MFAP3 | 207.7201835 | 176.3017751 | -0.23659 | 0.006144 | 0.006144 | Downregulated |
| SUV39H2 | 207.7201835 | 176.3017751 | -0.23659 | 0.006144 | 0.006144 | Downregulated |
| DMD | 207.7155963 | 176.3076923 | -0.23651 | 0.006161 | 0.006161 | Downregulated |
| FRMD6 | 207.7155963 | 176.3076923 | -0.23651 | 0.006161 | 0.006161 | Downregulated |
| DTHD1 | 150.3486239 | 127.6153846 | -0.23651 | 0.040812 | 0.040812 | Downregulated |
| ZNF415 | 206.6376147 | 175.4142012 | -0.23634 | 0.006478 | 0.006478 | Downregulated |
| PLS1 | 207.6926606 | 176.3372781 | -0.23611 | 0.006248 | 0.006248 | Downregulated |
| GLB1L | 207.6926606 | 176.3372781 | -0.23611 | 0.006248 | 0.006248 | Downregulated |
| FBXO36 | 207.6926606 | 176.3372781 | -0.23611 | 0.006248 | 0.006248 | Downregulated |
| BAG4 | 207.6880734 | 176.3431953 | -0.23603 | 0.006265 | 0.006265 | Downregulated |
| IGSF23 | 186.8577982 | 158.6568047 | -0.23603 | 0.012609 | 0.012609 | Downregulated |
| KCNG2 | 167.1330275 | 141.9112426 | -0.23601 | 0.028947 | 0.028947 | Downregulated |
| TRPM6 | 207.6834862 | 176.3491124 | -0.23595 | 0.006283 | 0.006283 | Downregulated |
| CAMK1G | 198.1697248 | 168.2781065 | -0.23589 | 0.009069 | 0.009069 | Downregulated |
| SIT1 | 207.6743119 | 176.3609467 | -0.23579 | 0.006318 | 0.006318 | Downregulated |
| EGFL6 | 207.6697248 | 176.3668639 | -0.23571 | 0.006335 | 0.006335 | Downregulated |
| IL18R1 | 207.6651376 | 176.3727811 | -0.23563 | 0.006353 | 0.006353 | Downregulated |
| ZNF264 | 207.6651376 | 176.3727811 | -0.23563 | 0.006353 | 0.006353 | Downregulated |
| F12 | 207.6605505 | 176.3786982 | -0.23555 | 0.006371 | 0.006371 | Downregulated |
| MCM10 | 207.6559633 | 176.3846154 | -0.23547 | 0.006388 | 0.006388 | Downregulated |
| JAK3 | 207.6559633 | 176.3846154 | -0.23547 | 0.006388 | 0.006388 | Downregulated |
| STAMBP | 207.6559633 | 176.3846154 | -0.23547 | 0.006388 | 0.006388 | Downregulated |
| MYLK4 | 207.6513761 | 176.3905325 | -0.23539 | 0.006406 | 0.006406 | Downregulated |
| JAML | 207.6513761 | 176.3905325 | -0.23539 | 0.006406 | 0.006406 | Downregulated |
| BCAT1 | 207.646789 | 176.3964497 | -0.23531 | 0.006424 | 0.006424 | Downregulated |
| CYYR1 | 207.6422018 | 176.4023669 | -0.23523 | 0.006442 | 0.006442 | Downregulated |
| UBXN4 | 207.6422018 | 176.4023669 | -0.23523 | 0.006442 | 0.006442 | Downregulated |
| CLEC12A | 196.0504587 | 166.556213 | -0.23522 | 0.010141 | 0.010141 | Downregulated |
| ICE2 | 207.6376147 | 176.408284 | -0.23515 | 0.00646 | 0.00646 | Downregulated |
| CIB1 | 207.6330275 | 176.4142012 | -0.23507 | 0.006478 | 0.006478 | Downregulated |
| RCHY1 | 207.6330275 | 176.4142012 | -0.23507 | 0.006478 | 0.006478 | Downregulated |
| EPHA3 | 207.6330275 | 176.4142012 | -0.23507 | 0.006478 | 0.006478 | Downregulated |
| TRPC3 | 189.853211 | 161.3254438 | -0.23491 | 0.01253 | 0.01253 | Downregulated |
| PLPBP | 207.6238532 | 176.4260355 | -0.23491 | 0.006514 | 0.006514 | Downregulated |
| FGF10 | 182.766055 | 155.3076923 | -0.23487 | 0.016093 | 0.016093 | Downregulated |
| LIN52 | 207.6192661 | 176.4319527 | -0.23483 | 0.006532 | 0.006532 | Downregulated |
| CCDC152 | 203.3577982 | 172.8284024 | -0.23468 | 0.007765 | 0.007765 | Downregulated |
| GIMAP6 | 207.6100917 | 176.443787 | -0.23467 | 0.006568 | 0.006568 | Downregulated |
| TXNRD3 | 207.6100917 | 176.443787 | -0.23467 | 0.006568 | 0.006568 | Downregulated |
| EXT2 | 207.6055046 | 176.4497041 | -0.23459 | 0.006586 | 0.006586 | Downregulated |
| SMYD5 | 207.5963303 | 176.4615385 | -0.23443 | 0.006623 | 0.006623 | Downregulated |
| WWTR1 | 207.5917431 | 176.4674556 | -0.23435 | 0.006641 | 0.006641 | Downregulated |
| SLFN11 | 207.5917431 | 176.4674556 | -0.23435 | 0.006641 | 0.006641 | Downregulated |
| GPATCH3 | 207.5917431 | 176.4674556 | -0.23435 | 0.006641 | 0.006641 | Downregulated |
| QSER1 | 207.587156 | 176.4733728 | -0.23427 | 0.00666 | 0.00666 | Downregulated |
| LSM11 | 207.5825688 | 176.4792899 | -0.23419 | 0.006678 | 0.006678 | Downregulated |
| UBE2E2 | 207.5825688 | 176.4792899 | -0.23419 | 0.006678 | 0.006678 | Downregulated |
| LAIR1 | 207.5779817 | 176.4852071 | -0.23411 | 0.006696 | 0.006696 | Downregulated |
| NFATC3 | 207.5779817 | 176.4852071 | -0.23411 | 0.006696 | 0.006696 | Downregulated |
| NCK2 | 207.5688073 | 176.4970414 | -0.23395 | 0.006734 | 0.006734 | Downregulated |
| KCTD12 | 207.5688073 | 176.4970414 | -0.23395 | 0.006734 | 0.006734 | Downregulated |
| BTK | 207.5688073 | 176.4970414 | -0.23395 | 0.006734 | 0.006734 | Downregulated |
| P2RX7 | 207.5688073 | 176.4970414 | -0.23395 | 0.006734 | 0.006734 | Downregulated |
| KIAA1211 | 207.5688073 | 176.4970414 | -0.23395 | 0.006734 | 0.006734 | Downregulated |
| CFAP74 | 197.0183486 | 167.5325444 | -0.23389 | 0.009837 | 0.009837 | Downregulated |
| CEACAM4 | 187.7614679 | 159.6627219 | -0.23387 | 0.014171 | 0.014171 | Downregulated |
| PRKCB | 207.5550459 | 176.5147929 | -0.2337 | 0.00679 | 0.00679 | Downregulated |
| HINT1 | 207.5550459 | 176.5147929 | -0.2337 | 0.00679 | 0.00679 | Downregulated |
| AC072022.1 | 143.940367 | 122.4201183 | -0.23363 | 0.048136 | 0.048136 | Downregulated |
| ZNF273 | 207.5504587 | 176.5207101 | -0.23362 | 0.006808 | 0.006808 | Downregulated |
| EMSY | 207.5458716 | 176.5266272 | -0.23354 | 0.006827 | 0.006827 | Downregulated |
| RAD51D | 207.5366972 | 176.5384615 | -0.23338 | 0.006865 | 0.006865 | Downregulated |
| AC092835.1 | 174.7614679 | 148.6627219 | -0.23334 | 0.020632 | 0.020632 | Downregulated |
| AP4E1 | 207.5275229 | 176.5502959 | -0.23322 | 0.006903 | 0.006903 | Downregulated |
| RAB2B | 207.5275229 | 176.5502959 | -0.23322 | 0.006903 | 0.006903 | Downregulated |
| MAP6D1 | 207.5229358 | 176.556213 | -0.23314 | 0.006922 | 0.006922 | Downregulated |
| MCM4 | 207.5229358 | 176.556213 | -0.23314 | 0.006922 | 0.006922 | Downregulated |
| CYB5R4 | 207.5137615 | 176.5680473 | -0.23298 | 0.00696 | 0.00696 | Downregulated |
| SIGLEC14 | 182.6651376 | 155.4378698 | -0.23286 | 0.016013 | 0.016013 | Downregulated |
| ZMAT5 | 207.5045872 | 176.5798817 | -0.23282 | 0.006999 | 0.006999 | Downregulated |
| CLEC5A | 207.5045872 | 176.5798817 | -0.23282 | 0.006999 | 0.006999 | Downregulated |
| ARPC2 | 207.5045872 | 176.5798817 | -0.23282 | 0.006999 | 0.006999 | Downregulated |
| KIR2DL4 | 156.6192661 | 133.2781065 | -0.23282 | 0.038627 | 0.038627 | Downregulated |
| HTRA4 | 187.706422 | 159.7337278 | -0.23281 | 0.01461 | 0.01461 | Downregulated |
| PRKAR2B | 207.5 | 176.5857988 | -0.23274 | 0.007018 | 0.007018 | Downregulated |
| CXorf21 | 206.4266055 | 175.6863905 | -0.23263 | 0.007343 | 0.007343 | Downregulated |
| INTS4 | 207.4816514 | 176.6094675 | -0.23242 | 0.007095 | 0.007095 | Downregulated |
| MDH1 | 207.4770642 | 176.6153846 | -0.23234 | 0.007115 | 0.007115 | Downregulated |
| RAD54B | 207.4724771 | 176.6213018 | -0.23226 | 0.007134 | 0.007134 | Downregulated |
| CD1B | 167.912844 | 142.9585799 | -0.23212 | 0.021783 | 0.021783 | Downregulated |
| CSPP1 | 207.4633028 | 176.6331361 | -0.2321 | 0.007174 | 0.007174 | Downregulated |
| PCSK5 | 207.4633028 | 176.6331361 | -0.2321 | 0.007174 | 0.007174 | Downregulated |
| MAPK8 | 207.4587156 | 176.6390533 | -0.23202 | 0.007193 | 0.007193 | Downregulated |
| SLC5A6 | 207.4541284 | 176.6449704 | -0.23194 | 0.007213 | 0.007213 | Downregulated |
| FOXN2 | 207.4495413 | 176.6508876 | -0.23186 | 0.007233 | 0.007233 | Downregulated |
| LSAMP | 207.4495413 | 176.6508876 | -0.23186 | 0.007233 | 0.007233 | Downregulated |
| CSF3R | 207.440367 | 176.6627219 | -0.2317 | 0.007273 | 0.007273 | Downregulated |
| LRRC29 | 207.4266055 | 176.6804734 | -0.23146 | 0.007333 | 0.007333 | Downregulated |
| CLMP | 207.4220183 | 176.6863905 | -0.23138 | 0.007353 | 0.007353 | Downregulated |
| GPM6A | 151.0412844 | 128.6686391 | -0.23128 | 0.037851 | 0.037851 | Downregulated |
| RALB | 207.412844 | 176.6982249 | -0.23122 | 0.007393 | 0.007393 | Downregulated |
| P2RY10 | 198.9587156 | 169.4970414 | -0.23121 | 0.010182 | 0.010182 | Downregulated |
| KCNN3 | 207.4036697 | 176.7100592 | -0.23106 | 0.007434 | 0.007434 | Downregulated |
| DDX19A | 207.3990826 | 176.7159763 | -0.23098 | 0.007454 | 0.007454 | Downregulated |
| ZNF175 | 207.3990826 | 176.7159763 | -0.23098 | 0.007454 | 0.007454 | Downregulated |
| GLRA4 | 161.2018349 | 137.3668639 | -0.23083 | 0.036581 | 0.036581 | Downregulated |
| AMIGO1 | 207.3899083 | 176.7278107 | -0.23082 | 0.007495 | 0.007495 | Downregulated |
| NOX4 | 207.3899083 | 176.7278107 | -0.23082 | 0.007495 | 0.007495 | Downregulated |
| TMED4 | 207.3853211 | 176.7337278 | -0.23074 | 0.007516 | 0.007516 | Downregulated |
| VDAC3 | 207.3807339 | 176.739645 | -0.23066 | 0.007536 | 0.007536 | Downregulated |
| SHBG | 180.5550459 | 153.8816568 | -0.23062 | 0.0205 | 0.0205 | Downregulated |
| MAGEE1 | 206.3119266 | 175.8343195 | -0.23061 | 0.007872 | 0.007872 | Downregulated |
| PAMR1 | 207.3761468 | 176.7455621 | -0.23058 | 0.007557 | 0.007557 | Downregulated |
| HVCN1 | 207.3669725 | 176.7573964 | -0.23042 | 0.007598 | 0.007598 | Downregulated |
| SLC19A3 | 207.3486239 | 176.7810651 | -0.23009 | 0.007681 | 0.007681 | Downregulated |
| DNAJC6 | 207.3486239 | 176.7810651 | -0.23009 | 0.007681 | 0.007681 | Downregulated |
| HIST1H2BN | 204.1559633 | 174.0650888 | -0.23004 | 0.008642 | 0.008642 | Downregulated |
| TIFAB | 195.7706422 | 166.9171598 | -0.23003 | 0.011565 | 0.011565 | Downregulated |
| C17orf75 | 207.3440367 | 176.7869822 | -0.23001 | 0.007702 | 0.007702 | Downregulated |
| MCM6 | 207.3440367 | 176.7869822 | -0.23001 | 0.007702 | 0.007702 | Downregulated |
| BBC3 | 207.3394495 | 176.7928994 | -0.22993 | 0.007723 | 0.007723 | Downregulated |
| GPR171 | 204.146789 | 174.0769231 | -0.22988 | 0.008688 | 0.008688 | Downregulated |
| PHOSPHO2 | 207.3348624 | 176.7988166 | -0.22985 | 0.007744 | 0.007744 | Downregulated |
| ZNF586 | 207.3348624 | 176.7988166 | -0.22985 | 0.007744 | 0.007744 | Downregulated |
| MTO1 | 206.353211 | 175.9704142 | -0.22978 | 0.008056 | 0.008056 | Downregulated |
| ZNF487 | 207.3302752 | 176.8047337 | -0.22977 | 0.007766 | 0.007766 | Downregulated |
| GUCA2B | 152.8027523 | 130.3076923 | -0.22975 | 0.037588 | 0.037588 | Downregulated |
| HIST1H2BG | 206.2568807 | 175.9053254 | -0.22964 | 0.008132 | 0.008132 | Downregulated |
| LAYN | 207.3211009 | 176.816568 | -0.22961 | 0.007808 | 0.007808 | Downregulated |
| ZNF77 | 207.3211009 | 176.816568 | -0.22961 | 0.007808 | 0.007808 | Downregulated |
| LRRFIP2 | 207.3165138 | 176.8224852 | -0.22953 | 0.007829 | 0.007829 | Downregulated |
| MAP3K7 | 207.3119266 | 176.8284024 | -0.22945 | 0.00785 | 0.00785 | Downregulated |
| CLSTN2 | 206.2431193 | 175.9230769 | -0.2294 | 0.008188 | 0.008188 | Downregulated |
| ASAP1 | 207.3073394 | 176.8343195 | -0.22937 | 0.007872 | 0.007872 | Downregulated |
| SELL | 207.3073394 | 176.8343195 | -0.22937 | 0.007872 | 0.007872 | Downregulated |
| OLFML1 | 207.0045872 | 176.5798817 | -0.22934 | 0.007776 | 0.007776 | Downregulated |
| ZNF578 | 163.9678899 | 139.8698225 | -0.22933 | 0.034133 | 0.034133 | Downregulated |
| TNFRSF4 | 207.2981651 | 176.8461538 | -0.22921 | 0.007915 | 0.007915 | Downregulated |
| SFRP1 | 197.7981651 | 168.7573964 | -0.22908 | 0.011106 | 0.011106 | Downregulated |
| PDE6B | 205.1605505 | 175.0414201 | -0.22906 | 0.00863 | 0.00863 | Downregulated |
| EFCAB8 | 165.8577982 | 141.5088757 | -0.22905 | 0.028273 | 0.028273 | Downregulated |
| SYNE3 | 207.2889908 | 176.8579882 | -0.22905 | 0.007958 | 0.007958 | Downregulated |
| ZFP82 | 207.2889908 | 176.8579882 | -0.22905 | 0.007958 | 0.007958 | Downregulated |
| CCR3 | 181.4724771 | 154.8343195 | -0.22903 | 0.019765 | 0.019765 | Downregulated |
| TBC1D15 | 207.2844037 | 176.8639053 | -0.22897 | 0.00798 | 0.00798 | Downregulated |
| BMT2 | 207.2752294 | 176.8757396 | -0.22881 | 0.008023 | 0.008023 | Downregulated |
| KCTD7 | 207.2752294 | 176.8757396 | -0.22881 | 0.008023 | 0.008023 | Downregulated |
| PDK2 | 207.2706422 | 176.8816568 | -0.22873 | 0.008045 | 0.008045 | Downregulated |
| SMC1B | 184.4587156 | 157.4260355 | -0.22862 | 0.017917 | 0.017917 | Downregulated |
| PPP2R5D | 207.2614679 | 176.8934911 | -0.22857 | 0.008088 | 0.008088 | Downregulated |
| FOPNL | 207.2568807 | 176.8994083 | -0.22849 | 0.00811 | 0.00811 | Downregulated |
| ANKRD33B | 207.2477064 | 176.9112426 | -0.22833 | 0.008154 | 0.008154 | Downregulated |
| TMEM237 | 207.2477064 | 176.9112426 | -0.22833 | 0.008154 | 0.008154 | Downregulated |
| PCDHGB1 | 159.2155963 | 135.9112426 | -0.22832 | 0.03455 | 0.03455 | Downregulated |
| GNAI3 | 207.2431193 | 176.9171598 | -0.22825 | 0.008177 | 0.008177 | Downregulated |
| RHD | 162.0275229 | 138.3195266 | -0.22823 | 0.033232 | 0.033232 | Downregulated |
| PREX2 | 207.2385321 | 176.9230769 | -0.22817 | 0.008199 | 0.008199 | Downregulated |
| DIXDC1 | 206.5275229 | 176.3254438 | -0.22809 | 0.008199 | 0.008199 | Downregulated |
| MFSD4B | 207.233945 | 176.9289941 | -0.22809 | 0.008221 | 0.008221 | Downregulated |
| PHYHIPL | 175.4862385 | 149.8284024 | -0.22805 | 0.024277 | 0.024277 | Downregulated |
| CYP2J2 | 207.2293578 | 176.9349112 | -0.22801 | 0.008243 | 0.008243 | Downregulated |
| PCDH18 | 207.2293578 | 176.9349112 | -0.22801 | 0.008243 | 0.008243 | Downregulated |
| CCDC167 | 206.9220183 | 176.6863905 | -0.2279 | 0.008056 | 0.008056 | Downregulated |
| HSPBAP1 | 207.2155963 | 176.9526627 | -0.22777 | 0.00831 | 0.00831 | Downregulated |
| ENPP2 | 207.2155963 | 176.9526627 | -0.22777 | 0.00831 | 0.00831 | Downregulated |
| PILRA | 207.2155963 | 176.9526627 | -0.22777 | 0.00831 | 0.00831 | Downregulated |
| SNUPN | 207.2110092 | 176.9585799 | -0.22769 | 0.008333 | 0.008333 | Downregulated |
| HLA-DOA | 207.2110092 | 176.9585799 | -0.22769 | 0.008333 | 0.008333 | Downregulated |
| BICC1 | 207.2110092 | 176.9585799 | -0.22769 | 0.008333 | 0.008333 | Downregulated |
| EIF2AK3 | 207.206422 | 176.964497 | -0.22761 | 0.008355 | 0.008355 | Downregulated |
| DNAJC24 | 207.206422 | 176.964497 | -0.22761 | 0.008355 | 0.008355 | Downregulated |
| ZNF879 | 207.206422 | 176.964497 | -0.22761 | 0.008355 | 0.008355 | Downregulated |
| ZNF66 | 199.8027523 | 170.6508876 | -0.22753 | 0.011063 | 0.011063 | Downregulated |
| FAM90A1 | 190.4862385 | 162.6982249 | -0.22749 | 0.015507 | 0.015507 | Downregulated |
| KLF1 | 177.4266055 | 151.5443787 | -0.22748 | 0.021668 | 0.021668 | Downregulated |
| PGS1 | 207.1972477 | 176.9763314 | -0.22745 | 0.008401 | 0.008401 | Downregulated |
| PMPCB | 207.1972477 | 176.9763314 | -0.22745 | 0.008401 | 0.008401 | Downregulated |
| MEI1 | 207.1972477 | 176.9763314 | -0.22745 | 0.008401 | 0.008401 | Downregulated |
| AGL | 207.1926606 | 176.9822485 | -0.22737 | 0.008423 | 0.008423 | Downregulated |
| HPSE | 207.1926606 | 176.9822485 | -0.22737 | 0.008423 | 0.008423 | Downregulated |
| ACBD7 | 205.0642202 | 175.1656805 | -0.22736 | 0.009106 | 0.009106 | Downregulated |
| C5orf58 | 158.233945 | 135.1775148 | -0.2272 | 0.035171 | 0.035171 | Downregulated |
| ZNF660 | 206.1100917 | 176.0946746 | -0.22706 | 0.008865 | 0.008865 | Downregulated |
| C22orf15 | 167.6788991 | 143.260355 | -0.22706 | 0.033818 | 0.033818 | Downregulated |
| SSPO | 195.6100917 | 167.1242604 | -0.22706 | 0.013213 | 0.013213 | Downregulated |
| DCP2 | 207.1743119 | 177.0059172 | -0.22705 | 0.008515 | 0.008515 | Downregulated |
| OLR1 | 206.1055046 | 176.1005917 | -0.22698 | 0.008877 | 0.008877 | Downregulated |
| PARP16 | 207.1697248 | 177.0118343 | -0.22697 | 0.008538 | 0.008538 | Downregulated |
| C10orf76 | 207.1651376 | 177.0177515 | -0.22689 | 0.008561 | 0.008561 | Downregulated |
| VSIG4 | 207.1651376 | 177.0177515 | -0.22689 | 0.008561 | 0.008561 | Downregulated |
| DOK2 | 207.1559633 | 177.0295858 | -0.22673 | 0.008607 | 0.008607 | Downregulated |
| YPEL5 | 207.1513761 | 177.035503 | -0.22665 | 0.00863 | 0.00863 | Downregulated |
| FBXO48 | 207.1513761 | 177.035503 | -0.22665 | 0.00863 | 0.00863 | Downregulated |
| SAMD8 | 207.1513761 | 177.035503 | -0.22665 | 0.00863 | 0.00863 | Downregulated |
| POGLUT1 | 207.146789 | 177.0414201 | -0.22657 | 0.008653 | 0.008653 | Downregulated |
| ERO1B | 207.146789 | 177.0414201 | -0.22657 | 0.008653 | 0.008653 | Downregulated |
| STX5 | 207.1422018 | 177.0473373 | -0.22649 | 0.008677 | 0.008677 | Downregulated |
| ZNF57 | 207.1422018 | 177.0473373 | -0.22649 | 0.008677 | 0.008677 | Downregulated |
| RAD51C | 207.1376147 | 177.0532544 | -0.22641 | 0.0087 | 0.0087 | Downregulated |
| BPGM | 207.1330275 | 177.0591716 | -0.22633 | 0.008724 | 0.008724 | Downregulated |
| CCL21 | 206.0642202 | 176.1538462 | -0.22626 | 0.009082 | 0.009082 | Downregulated |
| THUMPD2 | 207.1192661 | 177.0769231 | -0.22609 | 0.008794 | 0.008794 | Downregulated |
| ST6GAL1 | 207.1146789 | 177.0828402 | -0.22601 | 0.008818 | 0.008818 | Downregulated |
| LCA5 | 207.1146789 | 177.0828402 | -0.22601 | 0.008818 | 0.008818 | Downregulated |
| PTBP2 | 207.1100917 | 177.0887574 | -0.22593 | 0.008842 | 0.008842 | Downregulated |
| ESR2 | 201.8119266 | 172.5621302 | -0.2259 | 0.010791 | 0.010791 | Downregulated |
| ANKS1B | 164.7614679 | 140.8816568 | -0.2259 | 0.030376 | 0.030376 | Downregulated |
| IL26 | 140.9816514 | 120.556213 | -0.2258 | 0.042153 | 0.042153 | Downregulated |
| ERMARD | 207.1009174 | 177.1005917 | -0.22576 | 0.008889 | 0.008889 | Downregulated |
| SMIM10 | 207.0963303 | 177.1065089 | -0.22568 | 0.008913 | 0.008913 | Downregulated |
| SKIDA1 | 190.3899083 | 162.8224852 | -0.22566 | 0.017356 | 0.017356 | Downregulated |
| TAF2 | 207.0917431 | 177.112426 | -0.2256 | 0.008937 | 0.008937 | Downregulated |
| EFHD1 | 207.0825688 | 177.1242604 | -0.22544 | 0.008985 | 0.008985 | Downregulated |
| GOSR1 | 207.0779817 | 177.1301775 | -0.22536 | 0.009009 | 0.009009 | Downregulated |
| FOXP3 | 207.0779817 | 177.1301775 | -0.22536 | 0.009009 | 0.009009 | Downregulated |
| LILRA1 | 200.7293578 | 171.704142 | -0.22533 | 0.011282 | 0.011282 | Downregulated |
| ARL17B | 193.4495413 | 165.4792899 | -0.22531 | 0.014266 | 0.014266 | Downregulated |
| OPN1SW | 206.0091743 | 176.2248521 | -0.22529 | 0.00939 | 0.00939 | Downregulated |
| MLLT10 | 207.0733945 | 177.1360947 | -0.22528 | 0.009033 | 0.009033 | Downregulated |
| CNOT1 | 207.0688073 | 177.1420118 | -0.2252 | 0.009058 | 0.009058 | Downregulated |
| ZNF573 | 207.0688073 | 177.1420118 | -0.2252 | 0.009058 | 0.009058 | Downregulated |
| LOXL3 | 207.0642202 | 177.147929 | -0.22512 | 0.009082 | 0.009082 | Downregulated |
| GINS3 | 207.0642202 | 177.147929 | -0.22512 | 0.009082 | 0.009082 | Downregulated |
| CHORDC1 | 207.0642202 | 177.147929 | -0.22512 | 0.009082 | 0.009082 | Downregulated |
| NAF1 | 207.0504587 | 177.1656805 | -0.22488 | 0.009155 | 0.009155 | Downregulated |
| GALNS | 207.0504587 | 177.1656805 | -0.22488 | 0.009155 | 0.009155 | Downregulated |
| SPDL1 | 207.0458716 | 177.1715976 | -0.2248 | 0.00918 | 0.00918 | Downregulated |
| BRIP1 | 207.0366972 | 177.183432 | -0.22464 | 0.009229 | 0.009229 | Downregulated |
| PIK3CG | 207.0366972 | 177.183432 | -0.22464 | 0.009229 | 0.009229 | Downregulated |
| HAL | 202.793578 | 173.556213 | -0.22461 | 0.010734 | 0.010734 | Downregulated |
| TSPAN11 | 207.0275229 | 177.1952663 | -0.22448 | 0.009278 | 0.009278 | Downregulated |
| GAN | 207.0275229 | 177.1952663 | -0.22448 | 0.009278 | 0.009278 | Downregulated |
| HCLS1 | 207.0229358 | 177.2011834 | -0.2244 | 0.009303 | 0.009303 | Downregulated |
| C2CD2 | 207.0229358 | 177.2011834 | -0.2244 | 0.009303 | 0.009303 | Downregulated |
| RPS6 | 207.0229358 | 177.2011834 | -0.2244 | 0.009303 | 0.009303 | Downregulated |
| HTR2A | 180.2431193 | 154.2840237 | -0.22436 | 0.022692 | 0.022692 | Downregulated |
| GRB14 | 194.4266055 | 166.4319527 | -0.22429 | 0.013487 | 0.013487 | Downregulated |
| CEBPA | 207.0137615 | 177.2130178 | -0.22424 | 0.009353 | 0.009353 | Downregulated |
| ZNF521 | 207.0137615 | 177.2130178 | -0.22424 | 0.009353 | 0.009353 | Downregulated |
| SLC25A47 | 164.6834862 | 140.9822485 | -0.22418 | 0.034767 | 0.034767 | Downregulated |
| IKZF1 | 207 | 177.2307692 | -0.224 | 0.009428 | 0.009428 | Downregulated |
| METTL24 | 159.0229358 | 136.1597633 | -0.22393 | 0.03798 | 0.03798 | Downregulated |
| CCL2 | 206.9954128 | 177.2366864 | -0.22392 | 0.009453 | 0.009453 | Downregulated |
| CDON | 206.9908257 | 177.2426036 | -0.22384 | 0.009478 | 0.009478 | Downregulated |
| APBB1IP | 206.9862385 | 177.2485207 | -0.22376 | 0.009504 | 0.009504 | Downregulated |
| DPY19L4 | 206.9816514 | 177.2544379 | -0.22368 | 0.009529 | 0.009529 | Downregulated |
| NRG1 | 204.8486239 | 175.443787 | -0.22355 | 0.010345 | 0.010345 | Downregulated |
| LILRB2 | 206.9724771 | 177.2662722 | -0.22352 | 0.00958 | 0.00958 | Downregulated |
| NRAS | 206.9678899 | 177.2721893 | -0.22344 | 0.009606 | 0.009606 | Downregulated |
| TNFRSF18 | 206.9678899 | 177.2721893 | -0.22344 | 0.009606 | 0.009606 | Downregulated |
| ANO5 | 190.266055 | 162.9822485 | -0.2233 | 0.01647 | 0.01647 | Downregulated |
| CEP152 | 206.9587156 | 177.2840237 | -0.22328 | 0.009657 | 0.009657 | Downregulated |
| TYW5 | 206.9541284 | 177.2899408 | -0.2232 | 0.009682 | 0.009682 | Downregulated |
| PRKDC | 206.9495413 | 177.295858 | -0.22312 | 0.009708 | 0.009708 | Downregulated |
| TAF3 | 206.9495413 | 177.295858 | -0.22312 | 0.009708 | 0.009708 | Downregulated |
| UBE2E1 | 206.9449541 | 177.3017751 | -0.22304 | 0.009734 | 0.009734 | Downregulated |
| HEATR6 | 206.9357798 | 177.3136095 | -0.22288 | 0.009786 | 0.009786 | Downregulated |
| PSMD3 | 206.9357798 | 177.3136095 | -0.22288 | 0.009786 | 0.009786 | Downregulated |
| DDX42 | 206.9266055 | 177.3254438 | -0.22272 | 0.009838 | 0.009838 | Downregulated |
| PARP2 | 206.9266055 | 177.3254438 | -0.22272 | 0.009838 | 0.009838 | Downregulated |
| ZNF778 | 206.9220183 | 177.3313609 | -0.22264 | 0.009864 | 0.009864 | Downregulated |
| REXO2 | 206.9220183 | 177.3313609 | -0.22264 | 0.009864 | 0.009864 | Downregulated |
| IPMK | 206.9174312 | 177.3372781 | -0.22256 | 0.00989 | 0.00989 | Downregulated |
| TRAPPC6B | 206.9174312 | 177.3372781 | -0.22256 | 0.00989 | 0.00989 | Downregulated |
| CNST | 206.912844 | 177.3431953 | -0.22248 | 0.009917 | 0.009917 | Downregulated |
| PRSS36 | 206.9082569 | 177.3491124 | -0.2224 | 0.009943 | 0.009943 | Downregulated |
| SNTB2 | 206.9036697 | 177.3550296 | -0.22232 | 0.009969 | 0.009969 | Downregulated |
| MTHFD2L | 206.8990826 | 177.3609467 | -0.22224 | 0.009996 | 0.009996 | Downregulated |
| PRDX3 | 206.8944954 | 177.3668639 | -0.22216 | 0.010022 | 0.010022 | Downregulated |
| F13A1 | 206.8899083 | 177.3727811 | -0.22208 | 0.010049 | 0.010049 | Downregulated |
| RELA | 206.8899083 | 177.3727811 | -0.22208 | 0.010049 | 0.010049 | Downregulated |
| CCDC127 | 206.8807339 | 177.3846154 | -0.22192 | 0.010102 | 0.010102 | Downregulated |
| TMEM45B | 206.8807339 | 177.3846154 | -0.22192 | 0.010102 | 0.010102 | Downregulated |
| GBGT1 | 206.8807339 | 177.3846154 | -0.22192 | 0.010102 | 0.010102 | Downregulated |
| PRDM10 | 206.8761468 | 177.3905325 | -0.22184 | 0.010129 | 0.010129 | Downregulated |
| TSHZ2 | 206.8623853 | 177.408284 | -0.2216 | 0.01021 | 0.01021 | Downregulated |
| DPM1 | 206.8577982 | 177.4142012 | -0.22152 | 0.010237 | 0.010237 | Downregulated |
| DERL2 | 206.8577982 | 177.4142012 | -0.22152 | 0.010237 | 0.010237 | Downregulated |
| FGF7 | 205.793578 | 176.5029586 | -0.22151 | 0.010636 | 0.010636 | Downregulated |
| GSTCD | 206.853211 | 177.4201183 | -0.22144 | 0.010264 | 0.010264 | Downregulated |
| SLC25A12 | 206.8440367 | 177.4319527 | -0.22128 | 0.010318 | 0.010318 | Downregulated |
| KCNIP3 | 206.8440367 | 177.4319527 | -0.22128 | 0.010318 | 0.010318 | Downregulated |
| HHEX | 206.8440367 | 177.4319527 | -0.22128 | 0.010318 | 0.010318 | Downregulated |
| FCN1 | 205.7798165 | 176.5207101 | -0.22126 | 0.010734 | 0.010734 | Downregulated |
| ABCG2 | 206.8394495 | 177.4378698 | -0.2212 | 0.010346 | 0.010346 | Downregulated |
| TNFAIP8L2 | 206.293578 | 176.9704142 | -0.22119 | 0.010183 | 0.010183 | Downregulated |
| SIRPA | 206.8348624 | 177.443787 | -0.22112 | 0.010373 | 0.010373 | Downregulated |
| THEMIS | 203.6513761 | 174.7159763 | -0.22109 | 0.011566 | 0.011566 | Downregulated |
| TRMT61B | 206.8256881 | 177.4556213 | -0.22096 | 0.010428 | 0.010428 | Downregulated |
| HCK | 206.8211009 | 177.4615385 | -0.22088 | 0.010455 | 0.010455 | Downregulated |
| ZNF563 | 206.8165138 | 177.4674556 | -0.2208 | 0.010483 | 0.010483 | Downregulated |
| CHDH | 206.8165138 | 177.4674556 | -0.2208 | 0.010483 | 0.010483 | Downregulated |
| ZNF383 | 206.8165138 | 177.4674556 | -0.2208 | 0.010483 | 0.010483 | Downregulated |
| MRPS18B | 206.8165138 | 177.4674556 | -0.2208 | 0.010483 | 0.010483 | Downregulated |
| LHFPL3 | 174.1559633 | 149.443787 | -0.22078 | 0.030438 | 0.030438 | Downregulated |
| AL136531.2 | 197.3348624 | 169.3550296 | -0.2206 | 0.014579 | 0.014579 | Downregulated |
| PDS5B | 206.8027523 | 177.4852071 | -0.22056 | 0.010566 | 0.010566 | Downregulated |
| TREM1 | 206.8027523 | 177.4852071 | -0.22056 | 0.010566 | 0.010566 | Downregulated |
| PMS1 | 206.8027523 | 177.4852071 | -0.22056 | 0.010566 | 0.010566 | Downregulated |
| ZNF665 | 205.7385321 | 176.5739645 | -0.22054 | 0.010976 | 0.010976 | Downregulated |
| CLN5 | 206.7981651 | 177.4911243 | -0.22048 | 0.010594 | 0.010594 | Downregulated |
| AGMAT | 206.793578 | 177.4970414 | -0.2204 | 0.010622 | 0.010622 | Downregulated |
| RGS9BP | 167.3669725 | 143.6627219 | -0.22033 | 0.041149 | 0.041149 | Downregulated |
| SDC3 | 206.7889908 | 177.5029586 | -0.22032 | 0.01065 | 0.01065 | Downregulated |
| STK3 | 206.7889908 | 177.5029586 | -0.22032 | 0.01065 | 0.01065 | Downregulated |
| MS4A6A | 206.7798165 | 177.5147929 | -0.22016 | 0.010706 | 0.010706 | Downregulated |
| TPMT | 206.7798165 | 177.5147929 | -0.22016 | 0.010706 | 0.010706 | Downregulated |
| NR4A3 | 206.7752294 | 177.5207101 | -0.22008 | 0.010734 | 0.010734 | Downregulated |
| RBM43 | 206.7706422 | 177.5266272 | -0.22 | 0.010762 | 0.010762 | Downregulated |
| PSMC2 | 206.7706422 | 177.5266272 | -0.22 | 0.010762 | 0.010762 | Downregulated |
| RPL22L1 | 206.766055 | 177.5325444 | -0.21992 | 0.010791 | 0.010791 | Downregulated |
| MAP4K5 | 206.766055 | 177.5325444 | -0.21992 | 0.010791 | 0.010791 | Downregulated |
| MB21D2 | 206.766055 | 177.5325444 | -0.21992 | 0.010791 | 0.010791 | Downregulated |
| TPK1 | 206.766055 | 177.5325444 | -0.21992 | 0.010791 | 0.010791 | Downregulated |
| GHDC | 206.7568807 | 177.5443787 | -0.21976 | 0.010848 | 0.010848 | Downregulated |
| METTL21A | 206.7568807 | 177.5443787 | -0.21976 | 0.010848 | 0.010848 | Downregulated |
| XPNPEP3 | 206.7568807 | 177.5443787 | -0.21976 | 0.010848 | 0.010848 | Downregulated |
| GCNT7 | 180.0137615 | 154.5798817 | -0.21975 | 0.024034 | 0.024034 | Downregulated |
| ZNF354A | 206.7522936 | 177.5502959 | -0.21968 | 0.010876 | 0.010876 | Downregulated |
| DHX35 | 206.7522936 | 177.5502959 | -0.21968 | 0.010876 | 0.010876 | Downregulated |
| ATG7 | 206.7522936 | 177.5502959 | -0.21968 | 0.010876 | 0.010876 | Downregulated |
| WDR47 | 206.4541284 | 177.295858 | -0.21966 | 0.010622 | 0.010622 | Downregulated |
| ZNF354C | 203.5688073 | 174.8224852 | -0.21963 | 0.012167 | 0.012167 | Downregulated |
| HIST1H4I | 206.7477064 | 177.556213 | -0.2196 | 0.010905 | 0.010905 | Downregulated |
| SLC5A4 | 165.4174312 | 142.0769231 | -0.21944 | 0.040455 | 0.040455 | Downregulated |
| LRRC40 | 206.7385321 | 177.5680473 | -0.21944 | 0.010962 | 0.010962 | Downregulated |
| VSTM2L | 204.6146789 | 175.7455621 | -0.21942 | 0.011825 | 0.011825 | Downregulated |
| BBS7 | 206.733945 | 177.5739645 | -0.21936 | 0.010991 | 0.010991 | Downregulated |
| VCAM1 | 206.7293578 | 177.5798817 | -0.21928 | 0.01102 | 0.01102 | Downregulated |
| VASH2 | 206.7293578 | 177.5798817 | -0.21928 | 0.01102 | 0.01102 | Downregulated |
| CD177 | 206.7293578 | 177.5798817 | -0.21928 | 0.01102 | 0.01102 | Downregulated |
| DIS3 | 206.7247706 | 177.5857988 | -0.2192 | 0.011049 | 0.011049 | Downregulated |
| FAM117A | 206.7110092 | 177.6035503 | -0.21895 | 0.011136 | 0.011136 | Downregulated |
| IFNGR1 | 206.7110092 | 177.6035503 | -0.21895 | 0.011136 | 0.011136 | Downregulated |
| TBC1D22B | 206.7110092 | 177.6035503 | -0.21895 | 0.011136 | 0.011136 | Downregulated |
| GIMAP7 | 206.706422 | 177.6094675 | -0.21887 | 0.011165 | 0.011165 | Downregulated |
| ZNF266 | 206.7018349 | 177.6153846 | -0.21879 | 0.011194 | 0.011194 | Downregulated |
| TMEM200C | 204.5779817 | 175.7928994 | -0.21877 | 0.012073 | 0.012073 | Downregulated |
| RGS17 | 203.5183486 | 174.887574 | -0.21873 | 0.012519 | 0.012519 | Downregulated |
| ORC4 | 206.6926606 | 177.6272189 | -0.21863 | 0.011253 | 0.011253 | Downregulated |
| ZNF671 | 206.6880734 | 177.6331361 | -0.21855 | 0.011282 | 0.011282 | Downregulated |
| ENPEP | 206.6880734 | 177.6331361 | -0.21855 | 0.011282 | 0.011282 | Downregulated |
| CTBS | 206.6834862 | 177.6390533 | -0.21847 | 0.011312 | 0.011312 | Downregulated |
| NTMT1 | 206.3899083 | 177.3964497 | -0.2184 | 0.011238 | 0.011238 | Downregulated |
| PHACTR3 | 202.440367 | 174.0118343 | -0.21831 | 0.01318 | 0.01318 | Downregulated |
| RPS6KA5 | 206.6697248 | 177.6568047 | -0.21823 | 0.011401 | 0.011401 | Downregulated |
| ZDHHC11B | 206.6697248 | 177.6568047 | -0.21823 | 0.011401 | 0.011401 | Downregulated |
| TIGIT | 206.6697248 | 177.6568047 | -0.21823 | 0.011401 | 0.011401 | Downregulated |
| N4BP2L2 | 206.6697248 | 177.6568047 | -0.21823 | 0.011401 | 0.011401 | Downregulated |
| LILRB4 | 206.6651376 | 177.6627219 | -0.21815 | 0.011431 | 0.011431 | Downregulated |
| SLC4A1AP | 206.6605505 | 177.6686391 | -0.21807 | 0.011461 | 0.011461 | Downregulated |
| SORBS1 | 206.6559633 | 177.6745562 | -0.21799 | 0.011491 | 0.011491 | Downregulated |
| RAPH1 | 206.6559633 | 177.6745562 | -0.21799 | 0.011491 | 0.011491 | Downregulated |
| NEGR1 | 206.646789 | 177.6863905 | -0.21783 | 0.011551 | 0.011551 | Downregulated |
| CPED1 | 206.646789 | 177.6863905 | -0.21783 | 0.011551 | 0.011551 | Downregulated |
| NAB2 | 206.646789 | 177.6863905 | -0.21783 | 0.011551 | 0.011551 | Downregulated |
| PREX1 | 206.646789 | 177.6863905 | -0.21783 | 0.011551 | 0.011551 | Downregulated |
| CYP4F22 | 171.0963303 | 147.1242604 | -0.21777 | 0.030776 | 0.030776 | Downregulated |
| HABP4 | 206.6422018 | 177.6923077 | -0.21775 | 0.011581 | 0.011581 | Downregulated |
| MORF4L1 | 206.6376147 | 177.6982249 | -0.21767 | 0.011611 | 0.011611 | Downregulated |
| NMNAT2 | 202.4036697 | 174.0591716 | -0.21766 | 0.013317 | 0.013317 | Downregulated |
| CD163 | 206.6330275 | 177.704142 | -0.21759 | 0.011641 | 0.011641 | Downregulated |
| ZNF454 | 184.8990826 | 159.0177515 | -0.21755 | 0.022593 | 0.022593 | Downregulated |
| HIST1H2BC | 205.5642202 | 176.7988166 | -0.21748 | 0.01212 | 0.01212 | Downregulated |
| PPIP5K2 | 206.6238532 | 177.7159763 | -0.21743 | 0.011702 | 0.011702 | Downregulated |
| HPS5 | 206.6192661 | 177.7218935 | -0.21735 | 0.011733 | 0.011733 | Downregulated |
| MILR1 | 206.6192661 | 177.7218935 | -0.21735 | 0.011733 | 0.011733 | Downregulated |
| ZHX1-C8orf76 | 206.6192661 | 177.7218935 | -0.21735 | 0.011733 | 0.011733 | Downregulated |
| C4orf46 | 206.6192661 | 177.7218935 | -0.21735 | 0.011733 | 0.011733 | Downregulated |
| IL6 | 206.6146789 | 177.7278107 | -0.21727 | 0.011763 | 0.011763 | Downregulated |
| ZNF254 | 206.6100917 | 177.7337278 | -0.21719 | 0.011794 | 0.011794 | Downregulated |
| CUL1 | 206.6100917 | 177.7337278 | -0.21719 | 0.011794 | 0.011794 | Downregulated |
| TMEM121 | 204.4862385 | 175.9112426 | -0.21716 | 0.012714 | 0.012714 | Downregulated |
| TIMP3 | 206.6009174 | 177.7455621 | -0.21703 | 0.011855 | 0.011855 | Downregulated |
| PKIA | 206.6009174 | 177.7455621 | -0.21703 | 0.011855 | 0.011855 | Downregulated |
| ERN2 | 206.6009174 | 177.7455621 | -0.21703 | 0.011855 | 0.011855 | Downregulated |
| TCP1 | 206.6009174 | 177.7455621 | -0.21703 | 0.011855 | 0.011855 | Downregulated |
| FAM163A | 190.9541284 | 164.2899408 | -0.21698 | 0.020047 | 0.020047 | Downregulated |
| MSR1 | 206.5963303 | 177.7514793 | -0.21695 | 0.011886 | 0.011886 | Downregulated |
| ECSCR | 206.5963303 | 177.7514793 | -0.21695 | 0.011886 | 0.011886 | Downregulated |
| KIN | 206.5963303 | 177.7514793 | -0.21695 | 0.011886 | 0.011886 | Downregulated |
| NSUN5 | 206.5963303 | 177.7514793 | -0.21695 | 0.011886 | 0.011886 | Downregulated |
| RFX7 | 206.5963303 | 177.7514793 | -0.21695 | 0.011886 | 0.011886 | Downregulated |
| EFCAB7 | 206.5917431 | 177.7573964 | -0.21687 | 0.011917 | 0.011917 | Downregulated |
| CDPF1 | 206.5917431 | 177.7573964 | -0.21687 | 0.011917 | 0.011917 | Downregulated |
| TAF4 | 206.587156 | 177.7633136 | -0.21679 | 0.011948 | 0.011948 | Downregulated |
| NSUN3 | 206.587156 | 177.7633136 | -0.21679 | 0.011948 | 0.011948 | Downregulated |
| DPF1 | 205.5229358 | 176.852071 | -0.21676 | 0.012406 | 0.012406 | Downregulated |
| CWF19L2 | 206.5825688 | 177.7692308 | -0.21671 | 0.011979 | 0.011979 | Downregulated |
| STAG1 | 206.5825688 | 177.7692308 | -0.21671 | 0.011979 | 0.011979 | Downregulated |
| PHOSPHO1 | 201.293578 | 173.2307692 | -0.21661 | 0.01445 | 0.01445 | Downregulated |
| SYNJ1 | 206.5642202 | 177.7928994 | -0.21639 | 0.012104 | 0.012104 | Downregulated |
| HEXA | 206.5642202 | 177.7928994 | -0.21639 | 0.012104 | 0.012104 | Downregulated |
| CD180 | 206.559633 | 177.7988166 | -0.21631 | 0.012136 | 0.012136 | Downregulated |
| S100A16 | 206.559633 | 177.7988166 | -0.21631 | 0.012136 | 0.012136 | Downregulated |
| AJAP1 | 193.9954128 | 166.9881657 | -0.21628 | 0.018696 | 0.018696 | Downregulated |
| NAP1L5 | 206.5550459 | 177.8047337 | -0.21623 | 0.012167 | 0.012167 | Downregulated |
| ZNF711 | 206.5550459 | 177.8047337 | -0.21623 | 0.012167 | 0.012167 | Downregulated |
| CDC40 | 206.5550459 | 177.8047337 | -0.21623 | 0.012167 | 0.012167 | Downregulated |
| RPL17 | 206.5550459 | 177.8047337 | -0.21623 | 0.012167 | 0.012167 | Downregulated |
| ZNF558 | 206.5550459 | 177.8047337 | -0.21623 | 0.012167 | 0.012167 | Downregulated |
| ARL10 | 206.5504587 | 177.8106509 | -0.21615 | 0.012199 | 0.012199 | Downregulated |
| VMAC | 206.5504587 | 177.8106509 | -0.21615 | 0.012199 | 0.012199 | Downregulated |
| CDH26 | 191.9311927 | 165.2307692 | -0.21611 | 0.018104 | 0.018104 | Downregulated |
| FDXACB1 | 206.5412844 | 177.8224852 | -0.21599 | 0.012262 | 0.012262 | Downregulated |
| SF3A3 | 206.5366972 | 177.8284024 | -0.21591 | 0.012294 | 0.012294 | Downregulated |
| DOCK10 | 206.5321101 | 177.8343195 | -0.21583 | 0.012326 | 0.012326 | Downregulated |
| NSUN4 | 206.5321101 | 177.8343195 | -0.21583 | 0.012326 | 0.012326 | Downregulated |
| KLHL4 | 191.912844 | 165.2544379 | -0.21576 | 0.020269 | 0.020269 | Downregulated |
| ZNF793 | 204.4036697 | 176.0177515 | -0.2157 | 0.013351 | 0.013351 | Downregulated |
| GPX7 | 206.5229358 | 177.8461538 | -0.21567 | 0.01239 | 0.01239 | Downregulated |
| HELZ | 206.5183486 | 177.852071 | -0.21559 | 0.012422 | 0.012422 | Downregulated |
| ZNF281 | 206.5183486 | 177.852071 | -0.21559 | 0.012422 | 0.012422 | Downregulated |
| MAP3K7CL | 206.5183486 | 177.852071 | -0.21559 | 0.012422 | 0.012422 | Downregulated |
| HTR7 | 171.9541284 | 148.1005917 | -0.21545 | 0.033735 | 0.033735 | Downregulated |
| ARIH1 | 206.5045872 | 177.8698225 | -0.21535 | 0.012519 | 0.012519 | Downregulated |
| GRAMD1C | 206.5 | 177.8757396 | -0.21527 | 0.012551 | 0.012551 | Downregulated |
| ZNF486 | 206.5 | 177.8757396 | -0.21527 | 0.012551 | 0.012551 | Downregulated |
| THEMIS2 | 206.5 | 177.8757396 | -0.21527 | 0.012551 | 0.012551 | Downregulated |
| ADAMTS1 | 206.5 | 177.8757396 | -0.21527 | 0.012551 | 0.012551 | Downregulated |
| LRRC57 | 206.4954128 | 177.8816568 | -0.21519 | 0.012583 | 0.012583 | Downregulated |
| HIGD2A | 206.4954128 | 177.8816568 | -0.21519 | 0.012583 | 0.012583 | Downregulated |
| FCRL5 | 200.1651376 | 172.4319527 | -0.21516 | 0.015472 | 0.015472 | Downregulated |
| KDR | 206.4862385 | 177.8934911 | -0.21503 | 0.012649 | 0.012649 | Downregulated |
| GATB | 206.3669725 | 177.7928994 | -0.21501 | 0.012502 | 0.012502 | Downregulated |
| FAM162B | 200.1559633 | 172.443787 | -0.215 | 0.01555 | 0.01555 | Downregulated |
| ABCA6 | 200.146789 | 172.4556213 | -0.21483 | 0.015866 | 0.015866 | Downregulated |
| LRIG2 | 206.4724771 | 177.9112426 | -0.21479 | 0.012747 | 0.012747 | Downregulated |
| IL1R1 | 206.4678899 | 177.9171598 | -0.21471 | 0.01278 | 0.01278 | Downregulated |
| TRPC4 | 203.2752294 | 175.2011834 | -0.21442 | 0.014286 | 0.014286 | Downregulated |
| MKL2 | 206.4495413 | 177.9408284 | -0.21439 | 0.012912 | 0.012912 | Downregulated |
| ZNF568 | 206.4449541 | 177.9467456 | -0.21431 | 0.012945 | 0.012945 | Downregulated |
| GALNT18 | 206.4449541 | 177.9467456 | -0.21431 | 0.012945 | 0.012945 | Downregulated |
| CDIPT | 206.440367 | 177.9526627 | -0.21423 | 0.012979 | 0.012979 | Downregulated |
| GPR183 | 206.4357798 | 177.9585799 | -0.21415 | 0.013012 | 0.013012 | Downregulated |
| FBXO11 | 206.4357798 | 177.9585799 | -0.21415 | 0.013012 | 0.013012 | Downregulated |
| TOR1AIP1 | 206.4357798 | 177.9585799 | -0.21415 | 0.013012 | 0.013012 | Downregulated |
| DNAJC5 | 206.4357798 | 177.9585799 | -0.21415 | 0.013012 | 0.013012 | Downregulated |
| CYTH3 | 206.4357798 | 177.9585799 | -0.21415 | 0.013012 | 0.013012 | Downregulated |
| RBBP4 | 206.4357798 | 177.9585799 | -0.21415 | 0.013012 | 0.013012 | Downregulated |
| NGLY1 | 206.4357798 | 177.9585799 | -0.21415 | 0.013012 | 0.013012 | Downregulated |
| ELOVL4 | 201.1559633 | 173.408284 | -0.21414 | 0.01559 | 0.01559 | Downregulated |
| VIPAS39 | 206.3211009 | 177.8639053 | -0.21412 | 0.012879 | 0.012879 | Downregulated |
| RPS10 | 206.4311927 | 177.964497 | -0.21407 | 0.013046 | 0.013046 | Downregulated |
| ARRB1 | 206.4311927 | 177.964497 | -0.21407 | 0.013046 | 0.013046 | Downregulated |
| SLA2 | 206.4311927 | 177.964497 | -0.21407 | 0.013046 | 0.013046 | Downregulated |
| PCNX4 | 206.0045872 | 177.6094675 | -0.21397 | 0.012829 | 0.012829 | Downregulated |
| UTP23 | 206.4220183 | 177.9763314 | -0.21391 | 0.013113 | 0.013113 | Downregulated |
| CD300E | 202.1926606 | 174.3313609 | -0.2139 | 0.015125 | 0.015125 | Downregulated |
| THAP11 | 206.412844 | 177.9881657 | -0.21375 | 0.013181 | 0.013181 | Downregulated |
| ZC3H15 | 206.412844 | 177.9881657 | -0.21375 | 0.013181 | 0.013181 | Downregulated |
| MED12L | 172.8394495 | 149.0473373 | -0.21366 | 0.039144 | 0.039144 | Downregulated |
| NIN | 206.4036697 | 178 | -0.21359 | 0.013248 | 0.013248 | Downregulated |
| S100A14 | 206.3990826 | 178.0059172 | -0.21351 | 0.013282 | 0.013282 | Downregulated |
| BCL2A1 | 206.3990826 | 178.0059172 | -0.21351 | 0.013282 | 0.013282 | Downregulated |
| DCAF11 | 206.3990826 | 178.0059172 | -0.21351 | 0.013282 | 0.013282 | Downregulated |
| C16orf87 | 206.3944954 | 178.0118343 | -0.21343 | 0.013317 | 0.013317 | Downregulated |
| SYNCRIP | 206.3944954 | 178.0118343 | -0.21343 | 0.013317 | 0.013317 | Downregulated |
| COA1 | 206.3944954 | 178.0118343 | -0.21343 | 0.013317 | 0.013317 | Downregulated |
| ST6GALNAC5 | 205.3256881 | 177.1065089 | -0.2133 | 0.013838 | 0.013838 | Downregulated |
| C11orf63 | 205.3256881 | 177.1065089 | -0.2133 | 0.013856 | 0.013856 | Downregulated |
| ZEB1 | 206.3807339 | 178.0295858 | -0.21319 | 0.013419 | 0.013419 | Downregulated |
| MIXL1 | 176.7201835 | 152.4556213 | -0.21308 | 0.031161 | 0.031161 | Downregulated |
| DSCR3 | 206.3715596 | 178.0414201 | -0.21303 | 0.013488 | 0.013488 | Downregulated |
| FAM26F | 206.3715596 | 178.0414201 | -0.21303 | 0.013488 | 0.013488 | Downregulated |
| PHTF1 | 206.3623853 | 178.0532544 | -0.21287 | 0.013558 | 0.013558 | Downregulated |
| FAM26E | 206.3577982 | 178.0591716 | -0.21279 | 0.013592 | 0.013592 | Downregulated |
| SP100 | 206.3577982 | 178.0591716 | -0.21279 | 0.013592 | 0.013592 | Downregulated |
| FAM175A | 206.3577982 | 178.0591716 | -0.21279 | 0.013592 | 0.013592 | Downregulated |
| SPG21 | 206.3440367 | 178.0769231 | -0.21255 | 0.013697 | 0.013697 | Downregulated |
| ORC6 | 206.3440367 | 178.0769231 | -0.21255 | 0.013697 | 0.013697 | Downregulated |
| CPPED1 | 206.3440367 | 178.0769231 | -0.21255 | 0.013697 | 0.013697 | Downregulated |
| RBM39 | 206.3440367 | 178.0769231 | -0.21255 | 0.013697 | 0.013697 | Downregulated |
| SRSF2 | 206.3394495 | 178.0828402 | -0.21247 | 0.013732 | 0.013732 | Downregulated |
| ARL8B | 206.3394495 | 178.0828402 | -0.21247 | 0.013732 | 0.013732 | Downregulated |
| KCTD3 | 206.3348624 | 178.0887574 | -0.21239 | 0.013767 | 0.013767 | Downregulated |
| C8orf48 | 177.6697248 | 153.3491124 | -0.21238 | 0.029484 | 0.029484 | Downregulated |
| ERC2 | 172.7752294 | 149.1301775 | -0.21232 | 0.04188 | 0.04188 | Downregulated |
| NEK4 | 206.3302752 | 178.0946746 | -0.21231 | 0.013803 | 0.013803 | Downregulated |
| FEZ2 | 206.3256881 | 178.1005917 | -0.21223 | 0.013838 | 0.013838 | Downregulated |
| ZNF852 | 206.3256881 | 178.1005917 | -0.21223 | 0.013838 | 0.013838 | Downregulated |
| TMPPE | 206.3211009 | 178.1065089 | -0.21215 | 0.013873 | 0.013873 | Downregulated |
| FERMT2 | 206.3165138 | 178.112426 | -0.21207 | 0.013909 | 0.013909 | Downregulated |
| PTGFR | 203.1422018 | 175.3727811 | -0.21207 | 0.015492 | 0.015492 | Downregulated |
| NLGN4X | 199.9908257 | 172.6568047 | -0.21203 | 0.017018 | 0.017018 | Downregulated |
| GAB1 | 206.3119266 | 178.1183432 | -0.21199 | 0.013944 | 0.013944 | Downregulated |
| SLX4IP | 206.3073394 | 178.1242604 | -0.21191 | 0.01398 | 0.01398 | Downregulated |
| EPS8 | 206.3073394 | 178.1242604 | -0.21191 | 0.01398 | 0.01398 | Downregulated |
| AKT3 | 206.2981651 | 178.1360947 | -0.21175 | 0.014051 | 0.014051 | Downregulated |
| LATS1 | 206.2981651 | 178.1360947 | -0.21175 | 0.014051 | 0.014051 | Downregulated |
| TMEM145 | 203.1238532 | 175.3964497 | -0.21174 | 0.015531 | 0.015531 | Downregulated |
| SLC26A11 | 206.293578 | 178.1420118 | -0.21167 | 0.014087 | 0.014087 | Downregulated |
| TECTA | 194.7752294 | 168.2011834 | -0.21162 | 0.020001 | 0.020001 | Downregulated |
| PAPPA | 206.2889908 | 178.147929 | -0.21159 | 0.014123 | 0.014123 | Downregulated |
| SUSD3 | 206.2889908 | 178.147929 | -0.21159 | 0.014123 | 0.014123 | Downregulated |
| CEP170 | 206.2889908 | 178.147929 | -0.21159 | 0.014123 | 0.014123 | Downregulated |
| ADAMTS12 | 206.2889908 | 178.147929 | -0.21159 | 0.014123 | 0.014123 | Downregulated |
| DHFR2 | 206.2889908 | 178.147929 | -0.21159 | 0.014123 | 0.014123 | Downregulated |
| CD300LF | 206.2844037 | 178.1538462 | -0.21151 | 0.014159 | 0.014159 | Downregulated |
| ERI2 | 206.2844037 | 178.1538462 | -0.21151 | 0.014159 | 0.014159 | Downregulated |
| RORA | 206.2752294 | 178.1656805 | -0.21135 | 0.014232 | 0.014232 | Downregulated |
| CXCR6 | 206.2706422 | 178.1715976 | -0.21127 | 0.014268 | 0.014268 | Downregulated |
| LIFR | 206.2706422 | 178.1715976 | -0.21127 | 0.014268 | 0.014268 | Downregulated |
| DCSTAMP | 165.9908257 | 143.3846154 | -0.21121 | 0.04243 | 0.04243 | Downregulated |
| METTL9 | 206.266055 | 178.1775148 | -0.21119 | 0.014304 | 0.014304 | Downregulated |
| SPACA6 | 206.266055 | 178.1775148 | -0.21119 | 0.014304 | 0.014304 | Downregulated |
| NCR3LG1 | 206.266055 | 178.1775148 | -0.21119 | 0.014304 | 0.014304 | Downregulated |
| EMCN | 206.2614679 | 178.183432 | -0.21111 | 0.014341 | 0.014341 | Downregulated |
| IKBIP | 206.2568807 | 178.1893491 | -0.21103 | 0.014377 | 0.014377 | Downregulated |
| MMS22L | 206.2568807 | 178.1893491 | -0.21103 | 0.014377 | 0.014377 | Downregulated |
| SHPRH | 206.2568807 | 178.1893491 | -0.21103 | 0.014377 | 0.014377 | Downregulated |
| TUBA8 | 181.5642202 | 156.8579882 | -0.21102 | 0.032866 | 0.032866 | Downregulated |
| SRSF3 | 206.2522936 | 178.1952663 | -0.21095 | 0.014414 | 0.014414 | Downregulated |
| ZNF747 | 206.2522936 | 178.1952663 | -0.21095 | 0.014414 | 0.014414 | Downregulated |
| ADAM23 | 205.1880734 | 177.2840237 | -0.21088 | 0.014935 | 0.014935 | Downregulated |
| UNC13A | 206.2431193 | 178.2071006 | -0.21079 | 0.014487 | 0.014487 | Downregulated |
| FPR3 | 206.2385321 | 178.2130178 | -0.21071 | 0.014524 | 0.014524 | Downregulated |
| SLAMF8 | 206.2385321 | 178.2130178 | -0.21071 | 0.014524 | 0.014524 | Downregulated |
| PDE8B | 206.2293578 | 178.2248521 | -0.21055 | 0.014598 | 0.014598 | Downregulated |
| BCAS4 | 206.2293578 | 178.2248521 | -0.21055 | 0.014598 | 0.014598 | Downregulated |
| PCNX1 | 206.2293578 | 178.2248521 | -0.21055 | 0.014598 | 0.014598 | Downregulated |
| PLSCR1 | 206.2247706 | 178.2307692 | -0.21047 | 0.014635 | 0.014635 | Downregulated |
| IGSF9B | 206.2247706 | 178.2307692 | -0.21047 | 0.014635 | 0.014635 | Downregulated |
| SLC50A1 | 206.2201835 | 178.2366864 | -0.21039 | 0.014672 | 0.014672 | Downregulated |
| LILRB1 | 206.2155963 | 178.2426036 | -0.21031 | 0.014709 | 0.014709 | Downregulated |
| IL10RA | 206.2155963 | 178.2426036 | -0.21031 | 0.014709 | 0.014709 | Downregulated |
| COMMD2 | 206.2110092 | 178.2485207 | -0.21023 | 0.014747 | 0.014747 | Downregulated |
| WLS | 206.206422 | 178.2544379 | -0.21015 | 0.014784 | 0.014784 | Downregulated |
| SLC13A3 | 206.206422 | 178.2544379 | -0.21015 | 0.014784 | 0.014784 | Downregulated |
| ROBO1 | 206.206422 | 178.2544379 | -0.21015 | 0.014784 | 0.014784 | Downregulated |
| CCDC102B | 206.1926606 | 178.2721893 | -0.20991 | 0.014897 | 0.014897 | Downregulated |
| PTPN5 | 171.6880734 | 148.443787 | -0.20987 | 0.04809 | 0.04809 | Downregulated |
| DOK5 | 203.0183486 | 175.5325444 | -0.20987 | 0.016578 | 0.016578 | Downregulated |
| DTYMK | 206.1880734 | 178.2781065 | -0.20983 | 0.014935 | 0.014935 | Downregulated |
| DNAJC3 | 206.1834862 | 178.2840237 | -0.20975 | 0.014973 | 0.014973 | Downregulated |
| NUP155 | 206.1834862 | 178.2840237 | -0.20975 | 0.014973 | 0.014973 | Downregulated |
| CXCL13 | 201.9587156 | 174.6331361 | -0.20973 | 0.017188 | 0.017188 | Downregulated |
| SRD5A1 | 206.1788991 | 178.2899408 | -0.20967 | 0.015011 | 0.015011 | Downregulated |
| CENPBD1 | 206.1788991 | 178.2899408 | -0.20967 | 0.015011 | 0.015011 | Downregulated |
| ZNF250 | 206.1788991 | 178.2899408 | -0.20967 | 0.015011 | 0.015011 | Downregulated |
| RASSF5 | 206.1743119 | 178.295858 | -0.20959 | 0.015049 | 0.015049 | Downregulated |
| AKIRIN2 | 206.1743119 | 178.295858 | -0.20959 | 0.015049 | 0.015049 | Downregulated |
| RBM7 | 206.1743119 | 178.295858 | -0.20959 | 0.015049 | 0.015049 | Downregulated |
| SNW1 | 206.1697248 | 178.3017751 | -0.20951 | 0.015087 | 0.015087 | Downregulated |
| GCSAML | 152.8577982 | 132.2011834 | -0.20946 | 0.042724 | 0.042724 | Downregulated |
| MASP2 | 205.1009174 | 177.3964497 | -0.20936 | 0.015688 | 0.015688 | Downregulated |
| GIT1 | 206.1605505 | 178.3136095 | -0.20935 | 0.015163 | 0.015163 | Downregulated |
| RASL10B | 206.1605505 | 178.3136095 | -0.20935 | 0.015163 | 0.015163 | Downregulated |
| CDKN1B | 206.1559633 | 178.3195266 | -0.20927 | 0.015201 | 0.015201 | Downregulated |
| TNFRSF17 | 185.4770642 | 160.4378698 | -0.20923 | 0.028428 | 0.028428 | Downregulated |
| ARHGEF33 | 185.4724771 | 160.443787 | -0.20914 | 0.029849 | 0.029849 | Downregulated |
| MEIS1 | 206.146789 | 178.3313609 | -0.20911 | 0.015278 | 0.015278 | Downregulated |
| TM4SF20 | 206.146789 | 178.3313609 | -0.20911 | 0.015278 | 0.015278 | Downregulated |
| SLC37A1 | 206.1422018 | 178.3372781 | -0.20903 | 0.015317 | 0.015317 | Downregulated |
| WNK3 | 191.5504587 | 165.7218935 | -0.20896 | 0.023318 | 0.023318 | Downregulated |
| CD96 | 206.1376147 | 178.3431953 | -0.20895 | 0.015356 | 0.015356 | Downregulated |
| C7orf25 | 205.0733945 | 177.4319527 | -0.20887 | 0.015907 | 0.015907 | Downregulated |
| TMEM218 | 206.1330275 | 178.3491124 | -0.20887 | 0.015395 | 0.015395 | Downregulated |
| APOBEC3A | 198.7706422 | 171.9822485 | -0.20885 | 0.019234 | 0.019234 | Downregulated |
| TMEM156 | 202.9541284 | 175.6153846 | -0.20873 | 0.017103 | 0.017103 | Downregulated |
| TCF4 | 206.1192661 | 178.3668639 | -0.20863 | 0.015511 | 0.015511 | Downregulated |
| SMYD4 | 206.1192661 | 178.3668639 | -0.20863 | 0.015511 | 0.015511 | Downregulated |
| SPRTN | 206.1146789 | 178.3727811 | -0.20855 | 0.015551 | 0.015551 | Downregulated |
| C16orf52 | 206.1100917 | 178.3786982 | -0.20847 | 0.01559 | 0.01559 | Downregulated |
| IL1RAP | 206.1100917 | 178.3786982 | -0.20847 | 0.01559 | 0.01559 | Downregulated |
| DPY19L2 | 189.4816514 | 163.9940828 | -0.20841 | 0.024514 | 0.024514 | Downregulated |
| RPS6KB1 | 206.0963303 | 178.3964497 | -0.20823 | 0.015708 | 0.015708 | Downregulated |
| FANCD2 | 206.0963303 | 178.3964497 | -0.20823 | 0.015708 | 0.015708 | Downregulated |
| TSTD3 | 202.9220183 | 175.6568047 | -0.20817 | 0.017339 | 0.017339 | Downregulated |
| ARHGAP44 | 206.087156 | 178.408284 | -0.20807 | 0.015787 | 0.015787 | Downregulated |
| ABCE1 | 206.087156 | 178.408284 | -0.20807 | 0.015787 | 0.015787 | Downregulated |
| ADCY1 | 206.087156 | 178.408284 | -0.20807 | 0.015787 | 0.015787 | Downregulated |
| ANAPC4 | 206.0825688 | 178.4142012 | -0.20799 | 0.015827 | 0.015827 | Downregulated |
| CTLA4 | 206.0779817 | 178.4201183 | -0.20791 | 0.015867 | 0.015867 | Downregulated |
| NCOA2 | 206.0779817 | 178.4201183 | -0.20791 | 0.015867 | 0.015867 | Downregulated |
| KLRD1 | 205.0183486 | 177.5029586 | -0.20791 | 0.016413 | 0.016413 | Downregulated |
| HSPB9 | 197.6743119 | 171.1538462 | -0.20783 | 0.020721 | 0.020721 | Downregulated |
| CNNM1 | 200.8027523 | 173.8639053 | -0.20782 | 0.018652 | 0.018652 | Downregulated |
| C11orf65 | 194.5688073 | 168.4674556 | -0.20781 | 0.022307 | 0.022307 | Downregulated |
| AGO3 | 206.0688073 | 178.4319527 | -0.20775 | 0.015947 | 0.015947 | Downregulated |
| CASP3 | 206.0688073 | 178.4319527 | -0.20775 | 0.015947 | 0.015947 | Downregulated |
| IRF4 | 206.0688073 | 178.4319527 | -0.20775 | 0.015947 | 0.015947 | Downregulated |
| KIAA1328 | 206.0642202 | 178.4378698 | -0.20767 | 0.015987 | 0.015987 | Downregulated |
| C14orf28 | 206.059633 | 178.443787 | -0.20759 | 0.016027 | 0.016027 | Downregulated |
| PCMT1 | 206.0550459 | 178.4497041 | -0.20751 | 0.016067 | 0.016067 | Downregulated |
| LST1 | 206.0550459 | 178.4497041 | -0.20751 | 0.016067 | 0.016067 | Downregulated |
| MRM2 | 206.0550459 | 178.4497041 | -0.20751 | 0.016067 | 0.016067 | Downregulated |
| FAAP24 | 206.0504587 | 178.4556213 | -0.20743 | 0.016107 | 0.016107 | Downregulated |
| TTL | 206.0412844 | 178.4674556 | -0.20727 | 0.016188 | 0.016188 | Downregulated |
| CA3 | 206.0366972 | 178.4733728 | -0.20719 | 0.016229 | 0.016229 | Downregulated |
| NFATC1 | 206.0366972 | 178.4733728 | -0.20719 | 0.016229 | 0.016229 | Downregulated |
| PANK2 | 206.0275229 | 178.4852071 | -0.20703 | 0.016311 | 0.016311 | Downregulated |
| ZFP69 | 206.0275229 | 178.4852071 | -0.20703 | 0.016311 | 0.016311 | Downregulated |
| HAVCR2 | 206.0229358 | 178.4911243 | -0.20695 | 0.016352 | 0.016352 | Downregulated |
| TRIM28 | 206.0229358 | 178.4911243 | -0.20695 | 0.016352 | 0.016352 | Downregulated |
| ITGA8 | 206.0229358 | 178.4911243 | -0.20695 | 0.016352 | 0.016352 | Downregulated |
| ASCC1 | 206.0183486 | 178.4970414 | -0.20687 | 0.016393 | 0.016393 | Downregulated |
| AAGAB | 206.0183486 | 178.4970414 | -0.20687 | 0.016393 | 0.016393 | Downregulated |
| EXOG | 206.0183486 | 178.4970414 | -0.20687 | 0.016393 | 0.016393 | Downregulated |
| ZNF100 | 206.0137615 | 178.5029586 | -0.20679 | 0.016434 | 0.016434 | Downregulated |
| GDF15 | 206.0091743 | 178.5088757 | -0.20671 | 0.016475 | 0.016475 | Downregulated |
| CNTLN | 206.0045872 | 178.5147929 | -0.20663 | 0.016516 | 0.016516 | Downregulated |
| FIGNL1 | 206.0045872 | 178.5147929 | -0.20663 | 0.016516 | 0.016516 | Downregulated |
| LIX1L | 206 | 178.5207101 | -0.20655 | 0.016557 | 0.016557 | Downregulated |
| RGL1 | 205.9954128 | 178.5266272 | -0.20647 | 0.016599 | 0.016599 | Downregulated |
| PRF1 | 205.9954128 | 178.5266272 | -0.20647 | 0.016599 | 0.016599 | Downregulated |
| DNAJC28 | 202.8256881 | 175.7810651 | -0.20646 | 0.018333 | 0.018333 | Downregulated |
| HAUS2 | 205.9908257 | 178.5325444 | -0.20639 | 0.01664 | 0.01664 | Downregulated |
| SUGCT | 205.9862385 | 178.5384615 | -0.20631 | 0.016682 | 0.016682 | Downregulated |
| UQCRH | 205.9862385 | 178.5384615 | -0.20631 | 0.016682 | 0.016682 | Downregulated |
| EBF1 | 205.9862385 | 178.5384615 | -0.20631 | 0.016682 | 0.016682 | Downregulated |
| PLA2R1 | 205.9816514 | 178.5443787 | -0.20623 | 0.016724 | 0.016724 | Downregulated |
| BASP1 | 205.9770642 | 178.5502959 | -0.20615 | 0.016765 | 0.016765 | Downregulated |
| ZBTB11 | 205.9770642 | 178.5502959 | -0.20615 | 0.016765 | 0.016765 | Downregulated |
| PABPC4L | 193.4495413 | 167.6923077 | -0.20614 | 0.024576 | 0.024576 | Downregulated |
| LIPC | 191.3990826 | 165.9171598 | -0.20612 | 0.026073 | 0.026073 | Downregulated |
| STAU2 | 205.9724771 | 178.556213 | -0.20607 | 0.016807 | 0.016807 | Downregulated |
| CADM1 | 205.9724771 | 178.556213 | -0.20607 | 0.016807 | 0.016807 | Downregulated |
| TTPA | 200.7018349 | 173.9940828 | -0.20602 | 0.020051 | 0.020051 | Downregulated |
| CD244 | 202.793578 | 175.8224852 | -0.20589 | 0.018652 | 0.018652 | Downregulated |
| GORASP2 | 205.9587156 | 178.5739645 | -0.20583 | 0.016934 | 0.016934 | Downregulated |
| MS4A4A | 205.9587156 | 178.5739645 | -0.20583 | 0.016934 | 0.016934 | Downregulated |
| CDH13 | 205.9541284 | 178.5798817 | -0.20575 | 0.016976 | 0.016976 | Downregulated |
| CREBRF | 205.9541284 | 178.5798817 | -0.20575 | 0.016976 | 0.016976 | Downregulated |
| FER1L5 | 170.5229358 | 147.8639053 | -0.2057 | 0.045797 | 0.045797 | Downregulated |
| SRGAP2C | 203.8348624 | 176.7514793 | -0.20568 | 0.018153 | 0.018153 | Downregulated |
| CD300A | 205.9495413 | 178.5857988 | -0.20567 | 0.017018 | 0.017018 | Downregulated |
| THBD | 205.9449541 | 178.591716 | -0.20559 | 0.017061 | 0.017061 | Downregulated |
| S100PBP | 205.940367 | 178.5976331 | -0.20551 | 0.017103 | 0.017103 | Downregulated |
| C22orf39 | 205.9311927 | 178.6094675 | -0.20535 | 0.017189 | 0.017189 | Downregulated |
| SAMD4A | 205.9266055 | 178.6153846 | -0.20527 | 0.017231 | 0.017231 | Downregulated |
| PLK4 | 205.9174312 | 178.6272189 | -0.20511 | 0.017317 | 0.017317 | Downregulated |
| MSRB3 | 205.9174312 | 178.6272189 | -0.20511 | 0.017317 | 0.017317 | Downregulated |
| POLR2G | 205.9174312 | 178.6272189 | -0.20511 | 0.017317 | 0.017317 | Downregulated |
| IKZF3 | 205.912844 | 178.6331361 | -0.20503 | 0.01736 | 0.01736 | Downregulated |
| LRFN5 | 172.4220183 | 149.5857988 | -0.20497 | 0.041375 | 0.041375 | Downregulated |
| CD28 | 205.9082569 | 178.6390533 | -0.20495 | 0.017404 | 0.017404 | Downregulated |
| SMURF2 | 205.9036697 | 178.6449704 | -0.20487 | 0.017447 | 0.017447 | Downregulated |
| ERG | 205.9036697 | 178.6449704 | -0.20487 | 0.017447 | 0.017447 | Downregulated |
| TIGD7 | 205.8990826 | 178.6508876 | -0.20479 | 0.01749 | 0.01749 | Downregulated |
| ZBTB41 | 205.8990826 | 178.6508876 | -0.20479 | 0.01749 | 0.01749 | Downregulated |
| DLK2 | 205.8944954 | 178.6568047 | -0.20471 | 0.017534 | 0.017534 | Downregulated |
| LRRN2 | 205.8944954 | 178.6568047 | -0.20471 | 0.017534 | 0.017534 | Downregulated |
| SAMD14 | 205.8944954 | 178.6568047 | -0.20471 | 0.017534 | 0.017534 | Downregulated |
| RECQL | 205.8899083 | 178.6627219 | -0.20463 | 0.017577 | 0.017577 | Downregulated |
| MCOLN2 | 205.8899083 | 178.6627219 | -0.20463 | 0.017577 | 0.017577 | Downregulated |
| TAOK1 | 205.8761468 | 178.6804734 | -0.20439 | 0.017709 | 0.017709 | Downregulated |
| CHMP5 | 205.8761468 | 178.6804734 | -0.20439 | 0.017709 | 0.017709 | Downregulated |
| PRRT3 | 205.8715596 | 178.6863905 | -0.20431 | 0.017753 | 0.017753 | Downregulated |
| HOXA1 | 205.8715596 | 178.6863905 | -0.20431 | 0.017753 | 0.017753 | Downregulated |
| RTN3 | 205.8715596 | 178.6863905 | -0.20431 | 0.017753 | 0.017753 | Downregulated |
| SENP2 | 205.8669725 | 178.6923077 | -0.20423 | 0.017797 | 0.017797 | Downregulated |
| AC079447.1 | 198.5137615 | 172.3136095 | -0.2042 | 0.022228 | 0.022228 | Downregulated |
| ITK | 205.8623853 | 178.6982249 | -0.20415 | 0.017841 | 0.017841 | Downregulated |
| ZC3H6 | 205.8623853 | 178.6982249 | -0.20415 | 0.017841 | 0.017841 | Downregulated |
| SEMA6C | 205.8623853 | 178.6982249 | -0.20415 | 0.017841 | 0.017841 | Downregulated |
| NEPRO | 205.0917431 | 178.035503 | -0.2041 | 0.018041 | 0.018041 | Downregulated |
| RICTOR | 205.8577982 | 178.704142 | -0.20408 | 0.017885 | 0.017885 | Downregulated |
| CLTCL1 | 205.8577982 | 178.704142 | -0.20408 | 0.017885 | 0.017885 | Downregulated |
| TTC34 | 195.3944954 | 169.6272189 | -0.20402 | 0.024871 | 0.024871 | Downregulated |
| SH3GL1 | 205.853211 | 178.7100592 | -0.204 | 0.017929 | 0.017929 | Downregulated |
| DIO2 | 205.853211 | 178.7100592 | -0.204 | 0.017929 | 0.017929 | Downregulated |
| SRSF7 | 205.8486239 | 178.7159763 | -0.20392 | 0.017974 | 0.017974 | Downregulated |
| SHISA2 | 205.8486239 | 178.7159763 | -0.20392 | 0.017974 | 0.017974 | Downregulated |
| SFMBT1 | 205.8440367 | 178.7218935 | -0.20384 | 0.018018 | 0.018018 | Downregulated |
| LHX6 | 205.8440367 | 178.7218935 | -0.20384 | 0.018018 | 0.018018 | Downregulated |
| ZNF737 | 205.8394495 | 178.7278107 | -0.20376 | 0.018063 | 0.018063 | Downregulated |
| RIMKLA | 205.8348624 | 178.7337278 | -0.20368 | 0.018108 | 0.018108 | Downregulated |
| USP15 | 205.8348624 | 178.7337278 | -0.20368 | 0.018108 | 0.018108 | Downregulated |
| KLHDC1 | 205.8348624 | 178.7337278 | -0.20368 | 0.018108 | 0.018108 | Downregulated |
| ARSD | 205.8302752 | 178.739645 | -0.2036 | 0.018153 | 0.018153 | Downregulated |
| CD247 | 205.8302752 | 178.739645 | -0.2036 | 0.018153 | 0.018153 | Downregulated |
| LTK | 205.8302752 | 178.739645 | -0.2036 | 0.018153 | 0.018153 | Downregulated |
| FEZ1 | 205.8211009 | 178.7514793 | -0.20344 | 0.018242 | 0.018242 | Downregulated |
| PHKG2 | 205.8165138 | 178.7573964 | -0.20336 | 0.018288 | 0.018288 | Downregulated |
| SENP1 | 205.8165138 | 178.7573964 | -0.20336 | 0.018288 | 0.018288 | Downregulated |
| ABHD1 | 200.5458716 | 174.1952663 | -0.20323 | 0.021646 | 0.021646 | Downregulated |
| CAP2 | 205.8073394 | 178.7692308 | -0.2032 | 0.018378 | 0.018378 | Downregulated |
| ZSCAN18 | 205.8027523 | 178.7751479 | -0.20312 | 0.018424 | 0.018424 | Downregulated |
| CCDC116 | 180.1697248 | 156.5147929 | -0.20306 | 0.032415 | 0.032415 | Downregulated |
| PKD2 | 205.7981651 | 178.7810651 | -0.20304 | 0.018469 | 0.018469 | Downregulated |
| C11orf57 | 205.793578 | 178.7869822 | -0.20296 | 0.018515 | 0.018515 | Downregulated |
| P2RX5-TAX1BP3 | 205.793578 | 178.7869822 | -0.20296 | 0.018515 | 0.018515 | Downregulated |
| XPO6 | 205.7889908 | 178.7928994 | -0.20288 | 0.01856 | 0.01856 | Downregulated |
| GFPT2 | 205.7889908 | 178.7928994 | -0.20288 | 0.01856 | 0.01856 | Downregulated |
| ZSCAN26 | 205.7889908 | 178.7928994 | -0.20288 | 0.01856 | 0.01856 | Downregulated |
| TMEM255A | 201.5688073 | 175.1360947 | -0.2028 | 0.021179 | 0.021179 | Downregulated |
| ADORA2A | 178.1788991 | 154.816568 | -0.20277 | 0.040635 | 0.040635 | Downregulated |
| PRRG3 | 196.3577982 | 170.6153846 | -0.20274 | 0.024607 | 0.024607 | Downregulated |
| PXYLP1 | 205.7798165 | 178.8047337 | -0.20272 | 0.018652 | 0.018652 | Downregulated |
| POLG2 | 205.7798165 | 178.8047337 | -0.20272 | 0.018652 | 0.018652 | Downregulated |
| ZNF684 | 205.7798165 | 178.8047337 | -0.20272 | 0.018652 | 0.018652 | Downregulated |
| PIK3R5 | 205.7752294 | 178.8106509 | -0.20264 | 0.018698 | 0.018698 | Downregulated |
| FAM184A | 186.1376147 | 161.7573964 | -0.20254 | 0.03402 | 0.03402 | Downregulated |
| TAF13 | 205.766055 | 178.8224852 | -0.20248 | 0.01879 | 0.01879 | Downregulated |
| CD302 | 205.766055 | 178.8224852 | -0.20248 | 0.01879 | 0.01879 | Downregulated |
| C8orf76 | 205.766055 | 178.8224852 | -0.20248 | 0.01879 | 0.01879 | Downregulated |
| CEACAM21 | 205.7614679 | 178.8284024 | -0.2024 | 0.018837 | 0.018837 | Downregulated |
| MAGT1 | 205.7614679 | 178.8284024 | -0.2024 | 0.018837 | 0.018837 | Downregulated |
| ITSN2 | 205.7568807 | 178.8343195 | -0.20232 | 0.018883 | 0.018883 | Downregulated |
| MPZL3 | 205.7522936 | 178.8402367 | -0.20224 | 0.01893 | 0.01893 | Downregulated |
| SIN3A | 205.7522936 | 178.8402367 | -0.20224 | 0.01893 | 0.01893 | Downregulated |
| RAP1B | 205.7522936 | 178.8402367 | -0.20224 | 0.01893 | 0.01893 | Downregulated |
| SETDB2 | 205.7477064 | 178.8461538 | -0.20216 | 0.018976 | 0.018976 | Downregulated |
| FCRL2 | 179.1330275 | 155.7159763 | -0.20211 | 0.037466 | 0.037466 | Downregulated |
| SLC1A7 | 205.7431193 | 178.852071 | -0.20208 | 0.019023 | 0.019023 | Downregulated |
| RAB5C | 205.7385321 | 178.8579882 | -0.202 | 0.01907 | 0.01907 | Downregulated |
| S100B | 205.7385321 | 178.8579882 | -0.202 | 0.01907 | 0.01907 | Downregulated |
| CKB | 205.733945 | 178.8639053 | -0.20192 | 0.019117 | 0.019117 | Downregulated |
| CHST10 | 205.733945 | 178.8639053 | -0.20192 | 0.019117 | 0.019117 | Downregulated |
| C8orf34 | 175.1880734 | 152.3195266 | -0.2018 | 0.0425 | 0.0425 | Downregulated |
| OMG | 180.1055046 | 156.5976331 | -0.20178 | 0.040732 | 0.040732 | Downregulated |
| AP3M2 | 205.7247706 | 178.8757396 | -0.20176 | 0.019211 | 0.019211 | Downregulated |
| FCHSD2 | 205.7247706 | 178.8757396 | -0.20176 | 0.019211 | 0.019211 | Downregulated |
| PCDHGB4 | 186.0963303 | 161.8106509 | -0.20174 | 0.032541 | 0.032541 | Downregulated |
| TXNDC16 | 205.7201835 | 178.8816568 | -0.20168 | 0.019258 | 0.019258 | Downregulated |
| ANKRD44 | 205.7201835 | 178.8816568 | -0.20168 | 0.019258 | 0.019258 | Downregulated |
| MRPL47 | 205.7155963 | 178.887574 | -0.2016 | 0.019305 | 0.019305 | Downregulated |
| SYT11 | 205.7155963 | 178.887574 | -0.2016 | 0.019305 | 0.019305 | Downregulated |
| ZNF619 | 205.7110092 | 178.8934911 | -0.20152 | 0.019353 | 0.019353 | Downregulated |
| SLC30A7 | 205.7110092 | 178.8934911 | -0.20152 | 0.019353 | 0.019353 | Downregulated |
| PSMG1 | 205.706422 | 178.8994083 | -0.20144 | 0.0194 | 0.0194 | Downregulated |
| EID2 | 205.706422 | 178.8994083 | -0.20144 | 0.0194 | 0.0194 | Downregulated |
| AAED1 | 205.706422 | 178.8994083 | -0.20144 | 0.0194 | 0.0194 | Downregulated |
| OLFML2A | 205.706422 | 178.8994083 | -0.20144 | 0.0194 | 0.0194 | Downregulated |
| AASDHPPT | 205.7018349 | 178.9053254 | -0.20136 | 0.019448 | 0.019448 | Downregulated |
| ZC2HC1A | 205.6972477 | 178.9112426 | -0.20128 | 0.019495 | 0.019495 | Downregulated |
| ZNF831 | 198.3486239 | 172.5266272 | -0.20122 | 0.024229 | 0.024229 | Downregulated |
| NCF1 | 205.6926606 | 178.9171598 | -0.2012 | 0.019543 | 0.019543 | Downregulated |
| ATAD3C | 205.6926606 | 178.9171598 | -0.2012 | 0.019543 | 0.019543 | Downregulated |
| STIP1 | 205.6926606 | 178.9171598 | -0.2012 | 0.019543 | 0.019543 | Downregulated |
| FMO4 | 205.6926606 | 178.9171598 | -0.2012 | 0.019543 | 0.019543 | Downregulated |
| KLHL28 | 205.6880734 | 178.9230769 | -0.20112 | 0.019591 | 0.019591 | Downregulated |
| HARBI1 | 205.6880734 | 178.9230769 | -0.20112 | 0.019591 | 0.019591 | Downregulated |
| KCNT2 | 183.0458716 | 159.2485207 | -0.20093 | 0.037061 | 0.037061 | Downregulated |
| ZBTB5 | 205.6697248 | 178.9467456 | -0.2008 | 0.019784 | 0.019784 | Downregulated |
| NDRG3 | 205.6651376 | 178.9526627 | -0.20072 | 0.019832 | 0.019832 | Downregulated |
| HMCN2 | 205.6651376 | 178.9526627 | -0.20072 | 0.019832 | 0.019832 | Downregulated |
| SGCE | 205.6651376 | 178.9526627 | -0.20072 | 0.019832 | 0.019832 | Downregulated |
| ST3GAL3 | 205.6605505 | 178.9585799 | -0.20064 | 0.019881 | 0.019881 | Downregulated |
| AKAP2 | 189.0688073 | 164.5266272 | -0.20059 | 0.031991 | 0.031991 | Downregulated |
| JAM2 | 205.6513761 | 178.9704142 | -0.20048 | 0.019978 | 0.019978 | Downregulated |
| UBR2 | 205.646789 | 178.9763314 | -0.2004 | 0.020027 | 0.020027 | Downregulated |
| ACTR8 | 205.6422018 | 178.9822485 | -0.20032 | 0.020076 | 0.020076 | Downregulated |
| COPRS | 205.6376147 | 178.9881657 | -0.20024 | 0.020125 | 0.020125 | Downregulated |
| HNF4A | 205.6376147 | 178.9881657 | -0.20024 | 0.020125 | 0.020125 | Downregulated |
| RASGRP4 | 205.6330275 | 178.9940828 | -0.20016 | 0.020174 | 0.020174 | Downregulated |
| IL2RB | 205.6330275 | 178.9940828 | -0.20016 | 0.020174 | 0.020174 | Downregulated |
| PTX3 | 201.4174312 | 175.3313609 | -0.2001 | 0.022824 | 0.022824 | Downregulated |
| LLGL1 | 205.6284404 | 179 | -0.20008 | 0.020223 | 0.020223 | Downregulated |
| MITF | 205.6284404 | 179 | -0.20008 | 0.020223 | 0.020223 | Downregulated |
| GREB1 | 205.6284404 | 179 | -0.20008 | 0.020223 | 0.020223 | Downregulated |
| SLC22A31 | 178.6238532 | 155.5088757 | -0.19993 | 0.042846 | 0.042846 | Downregulated |
| CRISPLD2 | 205.6192661 | 179.0118343 | -0.19992 | 0.020322 | 0.020322 | Downregulated |
| TMEM54 | 205.6146789 | 179.0177515 | -0.19984 | 0.020372 | 0.020372 | Downregulated |
| VPS37B | 205.6146789 | 179.0177515 | -0.19984 | 0.020372 | 0.020372 | Downregulated |
| TROVE2 | 205.6100917 | 179.0236686 | -0.19976 | 0.020421 | 0.020421 | Downregulated |
| MUC13 | 205.6100917 | 179.0236686 | -0.19976 | 0.020421 | 0.020421 | Downregulated |
| PRIMPOL | 205.6055046 | 179.0295858 | -0.19968 | 0.020471 | 0.020471 | Downregulated |
| AVPR1A | 205.6055046 | 179.0295858 | -0.19968 | 0.020471 | 0.020471 | Downregulated |
| PRMT9 | 205.6009174 | 179.035503 | -0.1996 | 0.020521 | 0.020521 | Downregulated |
| VWA7 | 205.5963303 | 179.0414201 | -0.19952 | 0.020571 | 0.020571 | Downregulated |
| MPP2 | 205.587156 | 179.0532544 | -0.19936 | 0.020671 | 0.020671 | Downregulated |
| TMEM98 | 205.587156 | 179.0532544 | -0.19936 | 0.020671 | 0.020671 | Downregulated |
| CHEK2 | 205.587156 | 179.0532544 | -0.19936 | 0.020671 | 0.020671 | Downregulated |
| TENM3 | 205.5779817 | 179.0650888 | -0.1992 | 0.020772 | 0.020772 | Downregulated |
| GNG11 | 205.5733945 | 179.0710059 | -0.19912 | 0.020823 | 0.020823 | Downregulated |
| SERINC3 | 205.5688073 | 179.0769231 | -0.19904 | 0.020873 | 0.020873 | Downregulated |
| DGKQ | 205.5688073 | 179.0769231 | -0.19904 | 0.020873 | 0.020873 | Downregulated |
| CCDC87 | 197.1880734 | 171.7810651 | -0.199 | 0.026698 | 0.026698 | Downregulated |
| FANCM | 205.5642202 | 179.0828402 | -0.19896 | 0.020924 | 0.020924 | Downregulated |
| ZNF493 | 205.559633 | 179.0887574 | -0.19888 | 0.020975 | 0.020975 | Downregulated |
| ZNF559 | 205.5550459 | 179.0946746 | -0.1988 | 0.021026 | 0.021026 | Downregulated |
| PPIL1 | 205.5504587 | 179.1005917 | -0.19872 | 0.021077 | 0.021077 | Downregulated |
| SLC25A34 | 204.4908257 | 178.183432 | -0.19867 | 0.021803 | 0.021803 | Downregulated |
| KANSL1 | 205.5412844 | 179.112426 | -0.19856 | 0.021179 | 0.021179 | Downregulated |
| LILRB5 | 205.5412844 | 179.112426 | -0.19856 | 0.021179 | 0.021179 | Downregulated |
| TSPYL5 | 205.5366972 | 179.1183432 | -0.19848 | 0.021231 | 0.021231 | Downregulated |
| FBXW9 | 205.5321101 | 179.1242604 | -0.1984 | 0.021282 | 0.021282 | Downregulated |
| FABP3 | 205.5321101 | 179.1242604 | -0.1984 | 0.021282 | 0.021282 | Downregulated |
| SLC9A3R1 | 205.5275229 | 179.1301775 | -0.19832 | 0.021334 | 0.021334 | Downregulated |
| RNF219 | 205.5275229 | 179.1301775 | -0.19832 | 0.021334 | 0.021334 | Downregulated |
| OSBPL11 | 205.5229358 | 179.1360947 | -0.19824 | 0.021386 | 0.021386 | Downregulated |
| ADGRD1 | 205.5229358 | 179.1360947 | -0.19824 | 0.021386 | 0.021386 | Downregulated |
| ARID1A | 205.5137615 | 179.147929 | -0.19808 | 0.021489 | 0.021489 | Downregulated |
| ZNF292 | 205.5137615 | 179.147929 | -0.19808 | 0.021489 | 0.021489 | Downregulated |
| WIPF3 | 203.3990826 | 177.3136095 | -0.19801 | 0.022934 | 0.022934 | Downregulated |
| HERC5 | 205.5091743 | 179.1538462 | -0.198 | 0.021542 | 0.021542 | Downregulated |
| LY96 | 204.4495413 | 178.2366864 | -0.19795 | 0.022282 | 0.022282 | Downregulated |
| NEU3 | 205.5045872 | 179.1597633 | -0.19792 | 0.021594 | 0.021594 | Downregulated |
| CYTL1 | 196.0917431 | 170.9585799 | -0.19788 | 0.027913 | 0.027913 | Downregulated |
| HMGN4 | 205.5 | 179.1656805 | -0.19784 | 0.021646 | 0.021646 | Downregulated |
| PLA2G15 | 205.4954128 | 179.1715976 | -0.19776 | 0.021698 | 0.021698 | Downregulated |
| SERPINB9 | 205.4908257 | 179.1775148 | -0.19768 | 0.021751 | 0.021751 | Downregulated |
| MOB1B | 205.4908257 | 179.1775148 | -0.19768 | 0.021751 | 0.021751 | Downregulated |
| TRMT1L | 205.4908257 | 179.1775148 | -0.19768 | 0.021751 | 0.021751 | Downregulated |
| RBM12 | 205.4908257 | 179.1775148 | -0.19768 | 0.021751 | 0.021751 | Downregulated |
| SLC17A7 | 192.9908257 | 168.2840237 | -0.19763 | 0.031485 | 0.031485 | Downregulated |
| ZNF540 | 202.3256881 | 176.4260355 | -0.19762 | 0.023886 | 0.023886 | Downregulated |
| BACE1 | 205.4862385 | 179.183432 | -0.1976 | 0.021803 | 0.021803 | Downregulated |
| MRGBP | 205.4862385 | 179.183432 | -0.1976 | 0.021803 | 0.021803 | Downregulated |
| BBX | 205.4816514 | 179.1893491 | -0.19752 | 0.021856 | 0.021856 | Downregulated |
| DOK6 | 204.4220183 | 178.2721893 | -0.19747 | 0.022579 | 0.022579 | Downregulated |
| PINLYP | 205.4770642 | 179.1952663 | -0.19744 | 0.021909 | 0.021909 | Downregulated |
| UBE2I | 205.4770642 | 179.1952663 | -0.19744 | 0.021909 | 0.021909 | Downregulated |
| TTC39C | 205.4770642 | 179.1952663 | -0.19744 | 0.021909 | 0.021909 | Downregulated |
| ZNF491 | 204.4174312 | 178.2781065 | -0.19739 | 0.02266 | 0.02266 | Downregulated |
| KLRB1 | 205.4724771 | 179.2011834 | -0.19736 | 0.021962 | 0.021962 | Downregulated |
| GUCY1A3 | 205.4724771 | 179.2011834 | -0.19736 | 0.021962 | 0.021962 | Downregulated |
| IL7 | 205.4724771 | 179.2011834 | -0.19736 | 0.021962 | 0.021962 | Downregulated |
| TMEM55A | 205.4633028 | 179.2130178 | -0.19721 | 0.022068 | 0.022068 | Downregulated |
| IVD | 205.4587156 | 179.2189349 | -0.19713 | 0.022121 | 0.022121 | Downregulated |
| DSEL | 205.4587156 | 179.2189349 | -0.19713 | 0.022121 | 0.022121 | Downregulated |
| DOCK2 | 205.4541284 | 179.2248521 | -0.19705 | 0.022175 | 0.022175 | Downregulated |
| MED6 | 205.4495413 | 179.2307692 | -0.19697 | 0.022228 | 0.022228 | Downregulated |
| EPOP | 205.4495413 | 179.2307692 | -0.19697 | 0.022228 | 0.022228 | Downregulated |
| LBR | 205.4449541 | 179.2366864 | -0.19689 | 0.022282 | 0.022282 | Downregulated |
| EID3 | 205.4449541 | 179.2366864 | -0.19689 | 0.022282 | 0.022282 | Downregulated |
| PLEKHA1 | 205.4449541 | 179.2366864 | -0.19689 | 0.022282 | 0.022282 | Downregulated |
| ZNF835 | 192.9495413 | 168.3372781 | -0.19687 | 0.032142 | 0.032142 | Downregulated |
| TGFB3 | 205.440367 | 179.2426036 | -0.19681 | 0.022336 | 0.022336 | Downregulated |
| PCK1 | 205.440367 | 179.2426036 | -0.19681 | 0.022336 | 0.022336 | Downregulated |
| DGCR6 | 205.440367 | 179.2426036 | -0.19681 | 0.022336 | 0.022336 | Downregulated |
| TFIP11 | 205.4357798 | 179.2485207 | -0.19673 | 0.022389 | 0.022389 | Downregulated |
| IL21R | 205.4266055 | 179.260355 | -0.19657 | 0.022497 | 0.022497 | Downregulated |
| PYHIN1 | 202.266055 | 176.5029586 | -0.19656 | 0.024725 | 0.024725 | Downregulated |
| LILRA2 | 201.2155963 | 175.591716 | -0.19652 | 0.025348 | 0.025348 | Downregulated |
| PRSS21 | 182.8165138 | 159.5443787 | -0.19644 | 0.043475 | 0.043475 | Downregulated |
| RASSF4 | 205.1192661 | 179.0295858 | -0.19626 | 0.022497 | 0.022497 | Downregulated |
| CYB5D2 | 205.4036697 | 179.2899408 | -0.19617 | 0.022769 | 0.022769 | Downregulated |
| WDR7 | 205.4036697 | 179.2899408 | -0.19617 | 0.022769 | 0.022769 | Downregulated |
| ADNP | 205.3990826 | 179.295858 | -0.19609 | 0.022824 | 0.022824 | Downregulated |
| COLGALT1 | 205.3944954 | 179.3017751 | -0.19601 | 0.022879 | 0.022879 | Downregulated |
| TRAF3IP3 | 205.3899083 | 179.3076923 | -0.19593 | 0.022934 | 0.022934 | Downregulated |
| MXRA7 | 205.3899083 | 179.3076923 | -0.19593 | 0.022934 | 0.022934 | Downregulated |
| ANGPTL1 | 191.8715596 | 167.5147929 | -0.19585 | 0.032252 | 0.032252 | Downregulated |
| ABHD18 | 205.3853211 | 179.3136095 | -0.19585 | 0.022989 | 0.022989 | Downregulated |
| MPZL1 | 205.3853211 | 179.3136095 | -0.19585 | 0.022989 | 0.022989 | Downregulated |
| LTA | 199.087156 | 173.8224852 | -0.19579 | 0.027589 | 0.027589 | Downregulated |
| ACBD4 | 205.3807339 | 179.3195266 | -0.19577 | 0.023044 | 0.023044 | Downregulated |
| SCN3B | 194.940367 | 170.2130178 | -0.19569 | 0.030277 | 0.030277 | Downregulated |
| CHRNA1 | 199.0779817 | 173.8343195 | -0.19562 | 0.027719 | 0.027719 | Downregulated |
| USP49 | 205.3715596 | 179.3313609 | -0.19561 | 0.023155 | 0.023155 | Downregulated |
| IER3 | 205.3715596 | 179.3313609 | -0.19561 | 0.023155 | 0.023155 | Downregulated |
| RALGPS2 | 204.5825688 | 178.6508876 | -0.19554 | 0.023406 | 0.023406 | Downregulated |
| CUL5 | 205.3669725 | 179.3372781 | -0.19553 | 0.02321 | 0.02321 | Downregulated |
| ZBTB10 | 205.3669725 | 179.3372781 | -0.19553 | 0.02321 | 0.02321 | Downregulated |
| KLHL8 | 205.3623853 | 179.3431953 | -0.19545 | 0.023266 | 0.023266 | Downregulated |
| TTC32 | 205.3623853 | 179.3431953 | -0.19545 | 0.023266 | 0.023266 | Downregulated |
| COMMD9 | 205.3623853 | 179.3431953 | -0.19545 | 0.023266 | 0.023266 | Downregulated |
| ZNF200 | 205.3623853 | 179.3431953 | -0.19545 | 0.023266 | 0.023266 | Downregulated |
| ADGRE1 | 202.2018349 | 176.5857988 | -0.19543 | 0.025559 | 0.025559 | Downregulated |
| BOD1 | 205.2431193 | 179.2485207 | -0.19537 | 0.023127 | 0.023127 | Downregulated |
| TMEM59 | 205.3577982 | 179.3491124 | -0.19537 | 0.023322 | 0.023322 | Downregulated |
| AK4 | 205.3577982 | 179.3491124 | -0.19537 | 0.023322 | 0.023322 | Downregulated |
| TWIST1 | 205.353211 | 179.3550296 | -0.19529 | 0.023378 | 0.023378 | Downregulated |
| RNF169 | 205.353211 | 179.3550296 | -0.19529 | 0.023378 | 0.023378 | Downregulated |
| SOS1 | 205.353211 | 179.3550296 | -0.19529 | 0.023378 | 0.023378 | Downregulated |
| EED | 205.353211 | 179.3550296 | -0.19529 | 0.023378 | 0.023378 | Downregulated |
| OBSL1 | 205.353211 | 179.3550296 | -0.19529 | 0.023378 | 0.023378 | Downregulated |
| CD48 | 205.3486239 | 179.3609467 | -0.19521 | 0.023434 | 0.023434 | Downregulated |
| PIK3C3 | 205.3486239 | 179.3609467 | -0.19521 | 0.023434 | 0.023434 | Downregulated |
| IQSEC3 | 205.3440367 | 179.3668639 | -0.19513 | 0.02349 | 0.02349 | Downregulated |
| PANX1 | 205.3440367 | 179.3668639 | -0.19513 | 0.02349 | 0.02349 | Downregulated |
| BCL6B | 205.3394495 | 179.3727811 | -0.19505 | 0.023546 | 0.023546 | Downregulated |
| ZFP90 | 205.3394495 | 179.3727811 | -0.19505 | 0.023546 | 0.023546 | Downregulated |
| RAB40B | 205.3302752 | 179.3846154 | -0.19489 | 0.023659 | 0.023659 | Downregulated |
| GIMAP5 | 195.9266055 | 171.1715976 | -0.19487 | 0.030984 | 0.030984 | Downregulated |
| TNRC6A | 205.3256881 | 179.3905325 | -0.19481 | 0.023716 | 0.023716 | Downregulated |
| OSGIN2 | 205.3256881 | 179.3905325 | -0.19481 | 0.023716 | 0.023716 | Downregulated |
| C1orf162 | 205.3211009 | 179.3964497 | -0.19473 | 0.023772 | 0.023772 | Downregulated |
| UBA2 | 205.3211009 | 179.3964497 | -0.19473 | 0.023772 | 0.023772 | Downregulated |
| MS4A2 | 200.0688073 | 174.8106509 | -0.1947 | 0.027237 | 0.027237 | Downregulated |
| CABLES2 | 205.3165138 | 179.4023669 | -0.19465 | 0.023829 | 0.023829 | Downregulated |
| BIRC5 | 205.3165138 | 179.4023669 | -0.19465 | 0.023829 | 0.023829 | Downregulated |
| SLC47A1 | 205.3165138 | 179.4023669 | -0.19465 | 0.023829 | 0.023829 | Downregulated |
| ENOX1 | 205.3073394 | 179.4142012 | -0.19449 | 0.023943 | 0.023943 | Downregulated |
| TTC14 | 205.3073394 | 179.4142012 | -0.19449 | 0.023943 | 0.023943 | Downregulated |
| CERS5 | 205.3073394 | 179.4142012 | -0.19449 | 0.023943 | 0.023943 | Downregulated |
| RCN2 | 205.3073394 | 179.4142012 | -0.19449 | 0.023943 | 0.023943 | Downregulated |
| TTBK2 | 205.3073394 | 179.4142012 | -0.19449 | 0.023943 | 0.023943 | Downregulated |
| FKBP5 | 205.2981651 | 179.4260355 | -0.19433 | 0.024058 | 0.024058 | Downregulated |
| HS1BP3 | 205.293578 | 179.4319527 | -0.19425 | 0.024115 | 0.024115 | Downregulated |
| MPHOSPH9 | 205.293578 | 179.4319527 | -0.19425 | 0.024115 | 0.024115 | Downregulated |
| SPAST | 205.293578 | 179.4319527 | -0.19425 | 0.024115 | 0.024115 | Downregulated |
| CUL3 | 205.293578 | 179.4319527 | -0.19425 | 0.024115 | 0.024115 | Downregulated |
| TTC5 | 205.293578 | 179.4319527 | -0.19425 | 0.024115 | 0.024115 | Downregulated |
| TACR1 | 175.793578 | 153.6508876 | -0.19423 | 0.047348 | 0.047348 | Downregulated |
| ITGAX | 205.2889908 | 179.4378698 | -0.19417 | 0.024172 | 0.024172 | Downregulated |
| CBFB | 205.2889908 | 179.4378698 | -0.19417 | 0.024172 | 0.024172 | Downregulated |
| RPAP2 | 205.2889908 | 179.4378698 | -0.19417 | 0.024172 | 0.024172 | Downregulated |
| PPP1CC | 205.2889908 | 179.4378698 | -0.19417 | 0.024172 | 0.024172 | Downregulated |
| CLEC10A | 204.2293578 | 178.5207101 | -0.1941 | 0.024961 | 0.024961 | Downregulated |
| FAP | 205.2844037 | 179.443787 | -0.19409 | 0.02423 | 0.02423 | Downregulated |
| PHC3 | 205.2844037 | 179.443787 | -0.19409 | 0.02423 | 0.02423 | Downregulated |
| RASGRP1 | 205.2844037 | 179.443787 | -0.19409 | 0.02423 | 0.02423 | Downregulated |
| CFAP69 | 205.2798165 | 179.4497041 | -0.19401 | 0.024288 | 0.024288 | Downregulated |
| NAGS | 205.2706422 | 179.4615385 | -0.19385 | 0.024404 | 0.024404 | Downregulated |
| CD209 | 205.2706422 | 179.4615385 | -0.19385 | 0.024404 | 0.024404 | Downregulated |
| REL | 205.2706422 | 179.4615385 | -0.19385 | 0.024404 | 0.024404 | Downregulated |
| SIGLEC9 | 205.2614679 | 179.4733728 | -0.19369 | 0.02452 | 0.02452 | Downregulated |
| VAMP4 | 205.2614679 | 179.4733728 | -0.19369 | 0.02452 | 0.02452 | Downregulated |
| YPEL4 | 196.8944954 | 172.1597633 | -0.19367 | 0.0307 | 0.0307 | Downregulated |
| TUB | 205.2522936 | 179.4852071 | -0.19353 | 0.024637 | 0.024637 | Downregulated |
| GPR84 | 204.1926606 | 178.5680473 | -0.19346 | 0.025438 | 0.025438 | Downregulated |
| CXCR1 | 193.7798165 | 169.4852071 | -0.19326 | 0.033798 | 0.033798 | Downregulated |
| ADGRG2 | 198.9449541 | 174.0059172 | -0.19323 | 0.029655 | 0.029655 | Downregulated |
| CAPN7 | 205.233945 | 179.5088757 | -0.19321 | 0.024872 | 0.024872 | Downregulated |
| ZBTB44 | 205.2247706 | 179.5207101 | -0.19305 | 0.02499 | 0.02499 | Downregulated |
| WBP2 | 205.2247706 | 179.5207101 | -0.19305 | 0.02499 | 0.02499 | Downregulated |
| BATF2 | 205.2247706 | 179.5207101 | -0.19305 | 0.02499 | 0.02499 | Downregulated |
| KMT5B | 205.2201835 | 179.5266272 | -0.19297 | 0.02505 | 0.02505 | Downregulated |
| BRSK2 | 204.1605505 | 178.6094675 | -0.1929 | 0.025893 | 0.025893 | Downregulated |
| MICU1 | 205.2155963 | 179.5325444 | -0.19289 | 0.025109 | 0.025109 | Downregulated |
| WDR53 | 205.206422 | 179.5443787 | -0.19274 | 0.025229 | 0.025229 | Downregulated |
| CYTH4 | 205.206422 | 179.5443787 | -0.19274 | 0.025229 | 0.025229 | Downregulated |
| CACNB1 | 205.206422 | 179.5443787 | -0.19274 | 0.025229 | 0.025229 | Downregulated |
| ST13 | 205.2018349 | 179.5502959 | -0.19266 | 0.025288 | 0.025288 | Downregulated |
| CCL17 | 194.7706422 | 170.4319527 | -0.19258 | 0.033722 | 0.033722 | Downregulated |
| GRK4 | 205.1972477 | 179.556213 | -0.19258 | 0.025348 | 0.025348 | Downregulated |
| DPP8 | 205.1972477 | 179.556213 | -0.19258 | 0.025348 | 0.025348 | Downregulated |
| TRIM9 | 198.9082569 | 174.0532544 | -0.19257 | 0.03 | 0.03 | Downregulated |
| MAP3K14 | 205.1926606 | 179.5621302 | -0.1925 | 0.025408 | 0.025408 | Downregulated |
| MRPL11 | 205.1926606 | 179.5621302 | -0.1925 | 0.025408 | 0.025408 | Downregulated |
| LCN2 | 205.1880734 | 179.5680473 | -0.19242 | 0.025469 | 0.025469 | Downregulated |
| SOCS5 | 205.1788991 | 179.5798817 | -0.19226 | 0.025589 | 0.025589 | Downregulated |
| RNF146 | 205.1788991 | 179.5798817 | -0.19226 | 0.025589 | 0.025589 | Downregulated |
| HELLS | 205.1788991 | 179.5798817 | -0.19226 | 0.025589 | 0.025589 | Downregulated |
| SRGAP2B | 203.0688073 | 177.739645 | -0.1922 | 0.027269 | 0.027269 | Downregulated |
| PLEKHA8 | 205.1743119 | 179.5857988 | -0.19218 | 0.02565 | 0.02565 | Downregulated |
| SRSF1 | 205.1697248 | 179.591716 | -0.1921 | 0.025711 | 0.025711 | Downregulated |
| TFDP1 | 205.1697248 | 179.591716 | -0.1921 | 0.025711 | 0.025711 | Downregulated |
| DCBLD1 | 205.1697248 | 179.591716 | -0.1921 | 0.025711 | 0.025711 | Downregulated |
| STX2 | 205.1651376 | 179.5976331 | -0.19202 | 0.025771 | 0.025771 | Downregulated |
| LDB2 | 205.0779817 | 179.5266272 | -0.19197 | 0.025771 | 0.025771 | Downregulated |
| CD160 | 195.7614679 | 171.3846154 | -0.19186 | 0.032963 | 0.032963 | Downregulated |
| CAV2 | 205.1559633 | 179.6094675 | -0.19186 | 0.025893 | 0.025893 | Downregulated |
| REXO5 | 205.1559633 | 179.6094675 | -0.19186 | 0.025893 | 0.025893 | Downregulated |
| RBM45 | 205.1559633 | 179.6094675 | -0.19186 | 0.025893 | 0.025893 | Downregulated |
| SHQ1 | 205.1513761 | 179.6153846 | -0.19178 | 0.025955 | 0.025955 | Downregulated |
| SENP7 | 205.146789 | 179.6213018 | -0.1917 | 0.026016 | 0.026016 | Downregulated |
| BHLHE22 | 204.0917431 | 178.6982249 | -0.19169 | 0.026824 | 0.026824 | Downregulated |
| PLEK | 204.5733945 | 179.1301775 | -0.19161 | 0.026016 | 0.026016 | Downregulated |
| ZNF19 | 205.1376147 | 179.6331361 | -0.19154 | 0.026139 | 0.026139 | Downregulated |
| FHL1 | 205.1376147 | 179.6331361 | -0.19154 | 0.026139 | 0.026139 | Downregulated |
| GBP4 | 205.1376147 | 179.6331361 | -0.19154 | 0.026139 | 0.026139 | Downregulated |
| FPGT | 205.1330275 | 179.6390533 | -0.19146 | 0.026201 | 0.026201 | Downregulated |
| GAR1 | 205.1330275 | 179.6390533 | -0.19146 | 0.026201 | 0.026201 | Downregulated |
| CD109 | 205.1284404 | 179.6449704 | -0.19138 | 0.026262 | 0.026262 | Downregulated |
| ZNF436 | 205.1238532 | 179.6508876 | -0.1913 | 0.026324 | 0.026324 | Downregulated |
| ZNF713 | 205.1238532 | 179.6508876 | -0.1913 | 0.026324 | 0.026324 | Downregulated |
| 4-Sep | 205.1238532 | 179.6508876 | -0.1913 | 0.026324 | 0.026324 | Downregulated |
| STAU1 | 205.1192661 | 179.6568047 | -0.19122 | 0.026386 | 0.026386 | Downregulated |
| IREB2 | 205.1192661 | 179.6568047 | -0.19122 | 0.026386 | 0.026386 | Downregulated |
| EXOC6B | 205.1100917 | 179.6686391 | -0.19106 | 0.026511 | 0.026511 | Downregulated |
| SERINC5 | 205.1009174 | 179.6804734 | -0.1909 | 0.026636 | 0.026636 | Downregulated |
| STX19 | 205.1009174 | 179.6804734 | -0.1909 | 0.026636 | 0.026636 | Downregulated |
| SIAH1 | 205.1009174 | 179.6804734 | -0.1909 | 0.026636 | 0.026636 | Downregulated |
| HSD17B6 | 205.1009174 | 179.6804734 | -0.1909 | 0.026636 | 0.026636 | Downregulated |
| EPX | 182.5321101 | 159.9112426 | -0.19088 | 0.048566 | 0.048566 | Downregulated |
| PLA2G2D | 187.5412844 | 164.3076923 | -0.19081 | 0.041847 | 0.041847 | Downregulated |
| C3orf18 | 205.0917431 | 179.6923077 | -0.19074 | 0.026761 | 0.026761 | Downregulated |
| NUF2 | 205.0917431 | 179.6923077 | -0.19074 | 0.026761 | 0.026761 | Downregulated |
| FAM13B | 205.087156 | 179.6982249 | -0.19066 | 0.026824 | 0.026824 | Downregulated |
| SLCO2B1 | 205.087156 | 179.6982249 | -0.19066 | 0.026824 | 0.026824 | Downregulated |
| CPSF7 | 205.0825688 | 179.704142 | -0.19058 | 0.026888 | 0.026888 | Downregulated |
| PLEKHO2 | 205.0825688 | 179.704142 | -0.19058 | 0.026888 | 0.026888 | Downregulated |
| IDH3A | 205.0825688 | 179.704142 | -0.19058 | 0.026888 | 0.026888 | Downregulated |
| CAPZA2 | 205.0779817 | 179.7100592 | -0.1905 | 0.026951 | 0.026951 | Downregulated |
| MRPS10 | 205.0779817 | 179.7100592 | -0.1905 | 0.026951 | 0.026951 | Downregulated |
| MYOZ3 | 186.5137615 | 163.4497041 | -0.19044 | 0.044543 | 0.044543 | Downregulated |
| ANKRD13B | 205.0733945 | 179.7159763 | -0.19042 | 0.027014 | 0.027014 | Downregulated |
| ARL2 | 205.0688073 | 179.7218935 | -0.19034 | 0.027078 | 0.027078 | Downregulated |
| GNGT2 | 205.059633 | 179.7337278 | -0.19018 | 0.027205 | 0.027205 | Downregulated |
| ETFB | 205.059633 | 179.7337278 | -0.19018 | 0.027205 | 0.027205 | Downregulated |
| EBI3 | 205.0550459 | 179.739645 | -0.1901 | 0.027269 | 0.027269 | Downregulated |
| USP10 | 205.0550459 | 179.739645 | -0.1901 | 0.027269 | 0.027269 | Downregulated |
| STX4 | 204 | 178.8284024 | -0.18999 | 0.028078 | 0.028078 | Downregulated |
| POLDIP3 | 205.0412844 | 179.7573964 | -0.18986 | 0.027461 | 0.027461 | Downregulated |
| CES2 | 205.0412844 | 179.7573964 | -0.18986 | 0.027461 | 0.027461 | Downregulated |
| MCEMP1 | 193.5917431 | 169.7278107 | -0.18979 | 0.036623 | 0.036623 | Downregulated |
| MAT2B | 205.0321101 | 179.7692308 | -0.1897 | 0.02759 | 0.02759 | Downregulated |
| GIMAP4 | 205.0321101 | 179.7692308 | -0.1897 | 0.02759 | 0.02759 | Downregulated |
| ATF5 | 205.0321101 | 179.7692308 | -0.1897 | 0.02759 | 0.02759 | Downregulated |
| RASSF2 | 205.0229358 | 179.7810651 | -0.18954 | 0.027719 | 0.027719 | Downregulated |
| ZNF420 | 205.0229358 | 179.7810651 | -0.18954 | 0.027719 | 0.027719 | Downregulated |
| BNIP3 | 205.0229358 | 179.7810651 | -0.18954 | 0.027719 | 0.027719 | Downregulated |
| VWCE | 205.0229358 | 179.7810651 | -0.18954 | 0.027719 | 0.027719 | Downregulated |
| HIC2 | 205.0229358 | 179.7810651 | -0.18954 | 0.027719 | 0.027719 | Downregulated |
| KIAA1429 | 205.0183486 | 179.7869822 | -0.18946 | 0.027784 | 0.027784 | Downregulated |
| EPCAM | 205.0183486 | 179.7869822 | -0.18946 | 0.027784 | 0.027784 | Downregulated |
| COL4A4 | 205.0137615 | 179.7928994 | -0.18938 | 0.027849 | 0.027849 | Downregulated |
| PAK1 | 205.0091743 | 179.7988166 | -0.1893 | 0.027914 | 0.027914 | Downregulated |
| DEGS2 | 205.0091743 | 179.7988166 | -0.1893 | 0.027914 | 0.027914 | Downregulated |
| EEF1D | 205.0045872 | 179.8047337 | -0.18923 | 0.02798 | 0.02798 | Downregulated |
| MAP3K4 | 205.0045872 | 179.8047337 | -0.18923 | 0.02798 | 0.02798 | Downregulated |
| NCOA5 | 205.0045872 | 179.8047337 | -0.18923 | 0.02798 | 0.02798 | Downregulated |
| CAMSAP2 | 205 | 179.8106509 | -0.18915 | 0.028045 | 0.028045 | Downregulated |
| TOGARAM2 | 202.6192661 | 177.7455621 | -0.18896 | 0.029724 | 0.029724 | Downregulated |
| USP32 | 204.9816514 | 179.8343195 | -0.18883 | 0.028308 | 0.028308 | Downregulated |
| EML1 | 204.9770642 | 179.8402367 | -0.18875 | 0.028374 | 0.028374 | Downregulated |
| WDHD1 | 204.9770642 | 179.8402367 | -0.18875 | 0.028374 | 0.028374 | Downregulated |
| C12orf60 | 202.8715596 | 177.9940828 | -0.18874 | 0.03 | 0.03 | Downregulated |
| SLC25A33 | 204.9724771 | 179.8461538 | -0.18867 | 0.02844 | 0.02844 | Downregulated |
| SSH2 | 204.9678899 | 179.852071 | -0.18859 | 0.028507 | 0.028507 | Downregulated |
| CCDC157 | 204.9678899 | 179.852071 | -0.18859 | 0.028507 | 0.028507 | Downregulated |
| SPTBN1 | 204.9633028 | 179.8579882 | -0.18851 | 0.028573 | 0.028573 | Downregulated |
| SUGT1 | 204.9633028 | 179.8579882 | -0.18851 | 0.028573 | 0.028573 | Downregulated |
| METTL1 | 204.9587156 | 179.8639053 | -0.18843 | 0.02864 | 0.02864 | Downregulated |
| ZNF718 | 204.9541284 | 179.8698225 | -0.18835 | 0.028707 | 0.028707 | Downregulated |
| E2F5 | 204.9541284 | 179.8698225 | -0.18835 | 0.028707 | 0.028707 | Downregulated |
| BTLA | 198.6697248 | 174.3609467 | -0.1883 | 0.0338 | 0.0338 | Downregulated |
| MTPN | 204.9495413 | 179.8757396 | -0.18827 | 0.028773 | 0.028773 | Downregulated |
| IFNLR1 | 204.9449541 | 179.8816568 | -0.18819 | 0.02884 | 0.02884 | Downregulated |
| ZBTB21 | 204.9449541 | 179.8816568 | -0.18819 | 0.02884 | 0.02884 | Downregulated |
| CHM | 204.9357798 | 179.8934911 | -0.18803 | 0.028975 | 0.028975 | Downregulated |
| CACNA2D1 | 204.0917431 | 179.1597633 | -0.18797 | 0.029211 | 0.029211 | Downregulated |
| FHL5 | 201.7752294 | 177.1360947 | -0.18789 | 0.031525 | 0.031525 | Downregulated |
| FCMR | 204.9266055 | 179.9053254 | -0.18787 | 0.02911 | 0.02911 | Downregulated |
| NXN | 204.9266055 | 179.9053254 | -0.18787 | 0.02911 | 0.02911 | Downregulated |
| GZMB | 204.9266055 | 179.9053254 | -0.18787 | 0.02911 | 0.02911 | Downregulated |
| C2orf76 | 204.9220183 | 179.9112426 | -0.18779 | 0.029178 | 0.029178 | Downregulated |
| PHF20L1 | 204.9174312 | 179.9171598 | -0.18771 | 0.029245 | 0.029245 | Downregulated |
| FCGR2A | 204.9174312 | 179.9171598 | -0.18771 | 0.029245 | 0.029245 | Downregulated |
| UBR3 | 204.912844 | 179.9230769 | -0.18763 | 0.029313 | 0.029313 | Downregulated |
| TMEM185A | 204.9082569 | 179.9289941 | -0.18755 | 0.029381 | 0.029381 | Downregulated |
| ITPR1 | 204.9082569 | 179.9289941 | -0.18755 | 0.029381 | 0.029381 | Downregulated |
| GPR146 | 204.9082569 | 179.9289941 | -0.18755 | 0.029381 | 0.029381 | Downregulated |
| NUDT11 | 198.6238532 | 174.4201183 | -0.18747 | 0.034576 | 0.034576 | Downregulated |
| ITM2B | 204.9036697 | 179.9349112 | -0.18747 | 0.02945 | 0.02945 | Downregulated |
| PMS2 | 204.9036697 | 179.9349112 | -0.18747 | 0.02945 | 0.02945 | Downregulated |
| THBS2 | 204.8990826 | 179.9408284 | -0.18739 | 0.029518 | 0.029518 | Downregulated |
| ZBED8 | 204.8990826 | 179.9408284 | -0.18739 | 0.029518 | 0.029518 | Downregulated |
| HIP1 | 204.8990826 | 179.9408284 | -0.18739 | 0.029518 | 0.029518 | Downregulated |
| MTA3 | 204.8944954 | 179.9467456 | -0.18731 | 0.029587 | 0.029587 | Downregulated |
| DNAJA3 | 204.8944954 | 179.9467456 | -0.18731 | 0.029587 | 0.029587 | Downregulated |
| BST1 | 204.8944954 | 179.9467456 | -0.18731 | 0.029587 | 0.029587 | Downregulated |
| MRPS27 | 204.8899083 | 179.9526627 | -0.18723 | 0.029655 | 0.029655 | Downregulated |
| GRB2 | 204.8899083 | 179.9526627 | -0.18723 | 0.029655 | 0.029655 | Downregulated |
| EI24 | 204.8853211 | 179.9585799 | -0.18715 | 0.029724 | 0.029724 | Downregulated |
| C20orf144 | 200.6880734 | 176.2721893 | -0.18715 | 0.033266 | 0.033266 | Downregulated |
| CHI3L2 | 201.7293578 | 177.1952663 | -0.18708 | 0.032367 | 0.032367 | Downregulated |
| FLVCR1 | 204.8807339 | 179.964497 | -0.18707 | 0.029793 | 0.029793 | Downregulated |
| ST6GALNAC1 | 204.8807339 | 179.964497 | -0.18707 | 0.029793 | 0.029793 | Downregulated |
| CXorf36 | 204.8807339 | 179.964497 | -0.18707 | 0.029793 | 0.029793 | Downregulated |
| POLE3 | 204.8761468 | 179.9704142 | -0.18699 | 0.029862 | 0.029862 | Downregulated |
| ZNF692 | 204.8715596 | 179.9763314 | -0.18691 | 0.029931 | 0.029931 | Downregulated |
| CTSA | 204.8715596 | 179.9763314 | -0.18691 | 0.029931 | 0.029931 | Downregulated |
| LILRA6 | 203.8165138 | 179.0532544 | -0.18688 | 0.030843 | 0.030843 | Downregulated |
| DOK4 | 204.8669725 | 179.9822485 | -0.18683 | 0.03 | 0.03 | Downregulated |
| DCLK1 | 204.8623853 | 179.9881657 | -0.18675 | 0.03007 | 0.03007 | Downregulated |
| EXO1 | 204.8623853 | 179.9881657 | -0.18675 | 0.03007 | 0.03007 | Downregulated |
| ACLY | 204.8577982 | 179.9940828 | -0.18667 | 0.03014 | 0.03014 | Downregulated |
| KPNA1 | 204.8577982 | 179.9940828 | -0.18667 | 0.03014 | 0.03014 | Downregulated |
| MGAM | 200.6605505 | 176.3076923 | -0.18666 | 0.033878 | 0.033878 | Downregulated |
| LRRC69 | 194.4449541 | 170.852071 | -0.18661 | 0.04 | 0.04 | Downregulated |
| HLA-DQA2 | 201.7018349 | 177.2307692 | -0.1866 | 0.032927 | 0.032927 | Downregulated |
| C8orf44 | 204.853211 | 180 | -0.18659 | 0.030209 | 0.030209 | Downregulated |
| UBASH3B | 204.853211 | 180 | -0.18659 | 0.030209 | 0.030209 | Downregulated |
| INMT | 204.8486239 | 180.0059172 | -0.18651 | 0.030279 | 0.030279 | Downregulated |
| ARL9 | 191.3623853 | 168.1715976 | -0.18637 | 0.042595 | 0.042595 | Downregulated |
| MFAP5 | 203.7844037 | 179.0946746 | -0.18632 | 0.031308 | 0.031308 | Downregulated |
| ZACN | 198.559633 | 174.5029586 | -0.18632 | 0.035931 | 0.035931 | Downregulated |
| MRPL3 | 204.8348624 | 180.0236686 | -0.18627 | 0.03049 | 0.03049 | Downregulated |
| UCKL1 | 204.8302752 | 180.0295858 | -0.18619 | 0.03056 | 0.03056 | Downregulated |
| EHMT2 | 204.8302752 | 180.0295858 | -0.18619 | 0.03056 | 0.03056 | Downregulated |
| PUM2 | 204.8256881 | 180.035503 | -0.18612 | 0.030631 | 0.030631 | Downregulated |
| BAALC | 197.5091743 | 173.6094675 | -0.18607 | 0.037288 | 0.037288 | Downregulated |
| STAMBPL1 | 204.8211009 | 180.0414201 | -0.18604 | 0.030701 | 0.030701 | Downregulated |
| CSNK2A3 | 196.4724771 | 172.704142 | -0.18602 | 0.037835 | 0.037835 | Downregulated |
| NME7 | 204.8165138 | 180.0473373 | -0.18596 | 0.030772 | 0.030772 | Downregulated |
| SNTA1 | 204.8165138 | 180.0473373 | -0.18596 | 0.030772 | 0.030772 | Downregulated |
| RAB9B | 202.7110092 | 178.2011834 | -0.18592 | 0.032515 | 0.032515 | Downregulated |
| HOPX | 204.8119266 | 180.0532544 | -0.18588 | 0.030843 | 0.030843 | Downregulated |
| EDIL3 | 204.8119266 | 180.0532544 | -0.18588 | 0.030843 | 0.030843 | Downregulated |
| TTC30B | 204.8119266 | 180.0532544 | -0.18588 | 0.030843 | 0.030843 | Downregulated |
| CEP97 | 204.8119266 | 180.0532544 | -0.18588 | 0.030843 | 0.030843 | Downregulated |
| BHLHA15 | 202.706422 | 178.2071006 | -0.18584 | 0.03259 | 0.03259 | Downregulated |
| WDFY3 | 204.8073394 | 180.0591716 | -0.1858 | 0.030914 | 0.030914 | Downregulated |
| TMBIM4 | 204.8027523 | 180.0650888 | -0.18572 | 0.030986 | 0.030986 | Downregulated |
| ZNF287 | 204.8027523 | 180.0650888 | -0.18572 | 0.030986 | 0.030986 | Downregulated |
| 8-Mar | 204.7981651 | 180.0710059 | -0.18564 | 0.031057 | 0.031057 | Downregulated |
| KIAA1958 | 204.7981651 | 180.0710059 | -0.18564 | 0.031057 | 0.031057 | Downregulated |
| ZNF107 | 204.7981651 | 180.0710059 | -0.18564 | 0.031057 | 0.031057 | Downregulated |
| LURAP1 | 191.7477064 | 168.6035503 | -0.18557 | 0.04144 | 0.04144 | Downregulated |
| C6orf47 | 204.793578 | 180.0769231 | -0.18556 | 0.031129 | 0.031129 | Downregulated |
| MINDY2 | 204.7889908 | 180.0828402 | -0.18548 | 0.0312 | 0.0312 | Downregulated |
| CHTF8 | 204.7844037 | 180.0887574 | -0.1854 | 0.031272 | 0.031272 | Downregulated |
| TUBGCP5 | 204.7844037 | 180.0887574 | -0.1854 | 0.031272 | 0.031272 | Downregulated |
| GEMIN2 | 204.7844037 | 180.0887574 | -0.1854 | 0.031272 | 0.031272 | Downregulated |
| MTMR7 | 202.6788991 | 178.2426036 | -0.18535 | 0.033191 | 0.033191 | Downregulated |
| PARVG | 204.7798165 | 180.0946746 | -0.18532 | 0.031344 | 0.031344 | Downregulated |
| ELK1 | 204.7798165 | 180.0946746 | -0.18532 | 0.031344 | 0.031344 | Downregulated |
| EFCC1 | 204.7752294 | 180.1005917 | -0.18524 | 0.031416 | 0.031416 | Downregulated |
| ARID4A | 204.7752294 | 180.1005917 | -0.18524 | 0.031416 | 0.031416 | Downregulated |
| ZNF90 | 198.4954128 | 174.5857988 | -0.18517 | 0.03733 | 0.03733 | Downregulated |
| IST1 | 204.766055 | 180.112426 | -0.18508 | 0.031561 | 0.031561 | Downregulated |
| ADA2 | 204.7614679 | 180.1183432 | -0.185 | 0.031634 | 0.031634 | Downregulated |
| TRAF6 | 204.7568807 | 180.1242604 | -0.18492 | 0.031706 | 0.031706 | Downregulated |
| FGD2 | 204.7568807 | 180.1242604 | -0.18492 | 0.031706 | 0.031706 | Downregulated |
| PCDH19 | 198.4770642 | 174.6094675 | -0.18484 | 0.037666 | 0.037666 | Downregulated |
| CD274 | 204.7522936 | 180.1301775 | -0.18484 | 0.031779 | 0.031779 | Downregulated |
| LRP3 | 204.7522936 | 180.1301775 | -0.18484 | 0.031779 | 0.031779 | Downregulated |
| CCNY | 204.7477064 | 180.1360947 | -0.18476 | 0.031852 | 0.031852 | Downregulated |
| KLHL24 | 204.7477064 | 180.1360947 | -0.18476 | 0.031852 | 0.031852 | Downregulated |
| BMP2K | 204.7477064 | 180.1360947 | -0.18476 | 0.031852 | 0.031852 | Downregulated |
| C4orf3 | 204.7477064 | 180.1360947 | -0.18476 | 0.031852 | 0.031852 | Downregulated |
| ZHX3 | 204.7477064 | 180.1360947 | -0.18476 | 0.031852 | 0.031852 | Downregulated |
| MEST | 204.7477064 | 180.1360947 | -0.18476 | 0.031852 | 0.031852 | Downregulated |
| PKD1L1 | 204.7477064 | 180.1360947 | -0.18476 | 0.031852 | 0.031852 | Downregulated |
| DCLRE1B | 204.7477064 | 180.1360947 | -0.18476 | 0.031852 | 0.031852 | Downregulated |
| MOXD1 | 204.7431193 | 180.1420118 | -0.18468 | 0.031925 | 0.031925 | Downregulated |
| HNRNPUL2 | 204.7431193 | 180.1420118 | -0.18468 | 0.031925 | 0.031925 | Downregulated |
| MTSS1 | 204.7385321 | 180.147929 | -0.1846 | 0.031998 | 0.031998 | Downregulated |
| MBP | 204.733945 | 180.1538462 | -0.18452 | 0.032072 | 0.032072 | Downregulated |
| ZNF764 | 204.733945 | 180.1538462 | -0.18452 | 0.032072 | 0.032072 | Downregulated |
| STOX2 | 204.7293578 | 180.1597633 | -0.18444 | 0.032145 | 0.032145 | Downregulated |
| C20orf202 | 197.6834862 | 173.964497 | -0.1844 | 0.038175 | 0.038175 | Downregulated |
| ARHGEF7 | 204.7247706 | 180.1656805 | -0.18436 | 0.032219 | 0.032219 | Downregulated |
| POP1 | 204.7247706 | 180.1656805 | -0.18436 | 0.032219 | 0.032219 | Downregulated |
| TDP2 | 204.7247706 | 180.1656805 | -0.18436 | 0.032219 | 0.032219 | Downregulated |
| MAN1C1 | 204.7201835 | 180.1715976 | -0.18428 | 0.032293 | 0.032293 | Downregulated |
| SCHIP1 | 198.4449541 | 174.6508876 | -0.18426 | 0.037498 | 0.037498 | Downregulated |
| DZIP1 | 204.7155963 | 180.1775148 | -0.1842 | 0.032367 | 0.032367 | Downregulated |
| CPXM2 | 204.7110092 | 180.183432 | -0.18412 | 0.032441 | 0.032441 | Downregulated |
| GPR4 | 204.7110092 | 180.183432 | -0.18412 | 0.032441 | 0.032441 | Downregulated |
| C15orf41 | 204.706422 | 180.1893491 | -0.18404 | 0.032515 | 0.032515 | Downregulated |
| SLC23A1 | 204.706422 | 180.1893491 | -0.18404 | 0.032515 | 0.032515 | Downregulated |
| ZNF624 | 204.706422 | 180.1893491 | -0.18404 | 0.032515 | 0.032515 | Downregulated |
| SPECC1L | 204.7018349 | 180.1952663 | -0.18396 | 0.03259 | 0.03259 | Downregulated |
| IRAK3 | 204.7018349 | 180.1952663 | -0.18396 | 0.03259 | 0.03259 | Downregulated |
| TIMM21 | 204.7018349 | 180.1952663 | -0.18396 | 0.03259 | 0.03259 | Downregulated |
| MAPK8IP2 | 204.6972477 | 180.2011834 | -0.18388 | 0.032664 | 0.032664 | Downregulated |
| UHRF1BP1 | 204.6926606 | 180.2071006 | -0.1838 | 0.032739 | 0.032739 | Downregulated |
| RFC2 | 204.6926606 | 180.2071006 | -0.1838 | 0.032739 | 0.032739 | Downregulated |
| MYBBP1A | 204.6880734 | 180.2130178 | -0.18372 | 0.032814 | 0.032814 | Downregulated |
| ZNF136 | 204.6880734 | 180.2130178 | -0.18372 | 0.032814 | 0.032814 | Downregulated |
| PTPN9 | 204.6880734 | 180.2130178 | -0.18372 | 0.032814 | 0.032814 | Downregulated |
| RALA | 204.6834862 | 180.2189349 | -0.18364 | 0.032889 | 0.032889 | Downregulated |
| DCUN1D2 | 204.6834862 | 180.2189349 | -0.18364 | 0.032889 | 0.032889 | Downregulated |
| C20orf96 | 204.6834862 | 180.2189349 | -0.18364 | 0.032889 | 0.032889 | Downregulated |
| MYO1F | 204.6834862 | 180.2189349 | -0.18364 | 0.032889 | 0.032889 | Downregulated |
| DDB1 | 204.6788991 | 180.2248521 | -0.18356 | 0.032964 | 0.032964 | Downregulated |
| MROH8 | 204.6788991 | 180.2248521 | -0.18356 | 0.032964 | 0.032964 | Downregulated |
| ICA1L | 204.6788991 | 180.2248521 | -0.18356 | 0.032964 | 0.032964 | Downregulated |
| GLIS1 | 192.2293578 | 169.2662722 | -0.18353 | 0.044454 | 0.044454 | Downregulated |
| ALOX15B | 202.5733945 | 178.3786982 | -0.1835 | 0.034813 | 0.034813 | Downregulated |
| IPO11 | 204.6697248 | 180.2366864 | -0.18341 | 0.033115 | 0.033115 | Downregulated |
| DENND2C | 204.6697248 | 180.2366864 | -0.18341 | 0.033115 | 0.033115 | Downregulated |
| DSE | 204.6651376 | 180.2426036 | -0.18333 | 0.033191 | 0.033191 | Downregulated |
| UBXN7 | 204.6651376 | 180.2426036 | -0.18333 | 0.033191 | 0.033191 | Downregulated |
| ZNF691 | 204.6651376 | 180.2426036 | -0.18333 | 0.033191 | 0.033191 | Downregulated |
| CASC10 | 200.4724771 | 176.5502959 | -0.18332 | 0.036832 | 0.036832 | Downregulated |
| CD300LB | 201.5137615 | 177.4733728 | -0.18328 | 0.036134 | 0.036134 | Downregulated |
| FCN3 | 204.6605505 | 180.2485207 | -0.18325 | 0.033266 | 0.033266 | Downregulated |
| WWC2 | 204.6605505 | 180.2485207 | -0.18325 | 0.033266 | 0.033266 | Downregulated |
| CPXM1 | 204.6605505 | 180.2485207 | -0.18325 | 0.033266 | 0.033266 | Downregulated |
| DIP2C | 204.6559633 | 180.2544379 | -0.18317 | 0.033342 | 0.033342 | Downregulated |
| UBE2J1 | 204.6559633 | 180.2544379 | -0.18317 | 0.033342 | 0.033342 | Downregulated |
| NOCT | 204.646789 | 180.2662722 | -0.18301 | 0.033495 | 0.033495 | Downregulated |
| C4orf32 | 204.646789 | 180.2662722 | -0.18301 | 0.033495 | 0.033495 | Downregulated |
| GNL3 | 204.646789 | 180.2662722 | -0.18301 | 0.033495 | 0.033495 | Downregulated |
| POLR2M | 204.646789 | 180.2662722 | -0.18301 | 0.033495 | 0.033495 | Downregulated |
| TRPM7 | 204.646789 | 180.2662722 | -0.18301 | 0.033495 | 0.033495 | Downregulated |
| KIF15 | 204.6422018 | 180.2721893 | -0.18293 | 0.033571 | 0.033571 | Downregulated |
| THAP6 | 204.6376147 | 180.2781065 | -0.18285 | 0.033647 | 0.033647 | Downregulated |
| HIST1H4E | 200.4449541 | 176.5857988 | -0.18284 | 0.037331 | 0.037331 | Downregulated |
| DHX9 | 204.6330275 | 180.2840237 | -0.18277 | 0.033724 | 0.033724 | Downregulated |
| ARL14EP | 204.6330275 | 180.2840237 | -0.18277 | 0.033724 | 0.033724 | Downregulated |
| TNFAIP6 | 204.6330275 | 180.2840237 | -0.18277 | 0.033724 | 0.033724 | Downregulated |
| LDHA | 204.6330275 | 180.2840237 | -0.18277 | 0.033724 | 0.033724 | Downregulated |
| TMX3 | 204.6284404 | 180.2899408 | -0.18269 | 0.033801 | 0.033801 | Downregulated |
| TTYH2 | 204.6284404 | 180.2899408 | -0.18269 | 0.033801 | 0.033801 | Downregulated |
| CHST11 | 204.6284404 | 180.2899408 | -0.18269 | 0.033801 | 0.033801 | Downregulated |
| CDKL2 | 189.1330275 | 166.6390533 | -0.18267 | 0.049796 | 0.049796 | Downregulated |
| MPL | 191.1605505 | 168.4319527 | -0.18262 | 0.048219 | 0.048219 | Downregulated |
| LGI2 | 204.6238532 | 180.295858 | -0.18261 | 0.033878 | 0.033878 | Downregulated |
| EDC3 | 204.6238532 | 180.295858 | -0.18261 | 0.033878 | 0.033878 | Downregulated |
| CNNM4 | 204.6238532 | 180.295858 | -0.18261 | 0.033878 | 0.033878 | Downregulated |
| FBXO17 | 204.6238532 | 180.295858 | -0.18261 | 0.033878 | 0.033878 | Downregulated |
| KDM3A | 204.6192661 | 180.3017751 | -0.18253 | 0.033955 | 0.033955 | Downregulated |
| RBP1 | 204.6146789 | 180.3076923 | -0.18245 | 0.034032 | 0.034032 | Downregulated |
| CEP95 | 204.6100917 | 180.3136095 | -0.18237 | 0.034109 | 0.034109 | Downregulated |
| SASH3 | 204.6009174 | 180.3254438 | -0.18221 | 0.034265 | 0.034265 | Downregulated |
| NCKAP1L | 204.6009174 | 180.3254438 | -0.18221 | 0.034265 | 0.034265 | Downregulated |
| SLC37A2 | 204.6009174 | 180.3254438 | -0.18221 | 0.034265 | 0.034265 | Downregulated |
| CREG1 | 204.6009174 | 180.3254438 | -0.18221 | 0.034265 | 0.034265 | Downregulated |
| LRCH1 | 204.5963303 | 180.3313609 | -0.18213 | 0.034343 | 0.034343 | Downregulated |
| CPSF2 | 204.5963303 | 180.3313609 | -0.18213 | 0.034343 | 0.034343 | Downregulated |
| GLS2 | 204.5917431 | 180.3372781 | -0.18205 | 0.034421 | 0.034421 | Downregulated |
| SYNE4 | 204.5917431 | 180.3372781 | -0.18205 | 0.034421 | 0.034421 | Downregulated |
| FGD1 | 204.5917431 | 180.3372781 | -0.18205 | 0.034421 | 0.034421 | Downregulated |
| NAA20 | 204.5917431 | 180.3372781 | -0.18205 | 0.034421 | 0.034421 | Downregulated |
| DDX20 | 204.5917431 | 180.3372781 | -0.18205 | 0.034421 | 0.034421 | Downregulated |
| LRRC8B | 204.587156 | 180.3431953 | -0.18197 | 0.034499 | 0.034499 | Downregulated |
| TSSK6 | 204.087156 | 179.9053254 | -0.18195 | 0.034421 | 0.034421 | Downregulated |
| KDM6A | 204.5825688 | 180.3491124 | -0.18189 | 0.034577 | 0.034577 | Downregulated |
| H2AFZ | 204.5825688 | 180.3491124 | -0.18189 | 0.034577 | 0.034577 | Downregulated |
| CIITA | 204.5779817 | 180.3550296 | -0.18181 | 0.034655 | 0.034655 | Downregulated |
| SIGLEC7 | 203.5229358 | 179.4319527 | -0.18175 | 0.035649 | 0.035649 | Downregulated |
| SLC35A5 | 204.5733945 | 180.3609467 | -0.18173 | 0.034734 | 0.034734 | Downregulated |
| RANBP9 | 204.5733945 | 180.3609467 | -0.18173 | 0.034734 | 0.034734 | Downregulated |
| PRKD3 | 204.5642202 | 180.3727811 | -0.18157 | 0.034892 | 0.034892 | Downregulated |
| DLC1 | 204.5642202 | 180.3727811 | -0.18157 | 0.034892 | 0.034892 | Downregulated |
| TMEM249 | 195.1926606 | 172.1183432 | -0.1815 | 0.043495 | 0.043495 | Downregulated |
| SEC23B | 204.559633 | 180.3786982 | -0.18149 | 0.034971 | 0.034971 | Downregulated |
| NLRP2 | 204.5550459 | 180.3846154 | -0.18141 | 0.03505 | 0.03505 | Downregulated |
| DGCR6L | 204.5550459 | 180.3846154 | -0.18141 | 0.03505 | 0.03505 | Downregulated |
| RRM2B | 204.5458716 | 180.3964497 | -0.18125 | 0.035209 | 0.035209 | Downregulated |
| YAF2 | 204.5412844 | 180.4023669 | -0.18117 | 0.035289 | 0.035289 | Downregulated |
| ZFP69B | 204.5412844 | 180.4023669 | -0.18117 | 0.035289 | 0.035289 | Downregulated |
| ATF4 | 204.5412844 | 180.4023669 | -0.18117 | 0.035289 | 0.035289 | Downregulated |
| RELL2 | 204.5366972 | 180.408284 | -0.18109 | 0.035368 | 0.035368 | Downregulated |
| APOM | 204.5366972 | 180.408284 | -0.18109 | 0.035368 | 0.035368 | Downregulated |
| TGFBR3 | 204.5366972 | 180.408284 | -0.18109 | 0.035368 | 0.035368 | Downregulated |
| EHD3 | 204.5366972 | 180.408284 | -0.18109 | 0.035368 | 0.035368 | Downregulated |
| IRF2 | 204.5321101 | 180.4142012 | -0.18101 | 0.035448 | 0.035448 | Downregulated |
| CLSPN | 204.5321101 | 180.4142012 | -0.18101 | 0.035448 | 0.035448 | Downregulated |
| CDC7 | 204.5321101 | 180.4142012 | -0.18101 | 0.035448 | 0.035448 | Downregulated |
| RSPO3 | 203.4770642 | 179.4911243 | -0.18095 | 0.036461 | 0.036461 | Downregulated |
| DDX18 | 204.5275229 | 180.4201183 | -0.18093 | 0.035528 | 0.035528 | Downregulated |
| CCZ1B | 204.5275229 | 180.4201183 | -0.18093 | 0.035528 | 0.035528 | Downregulated |
| CTH | 204.5275229 | 180.4201183 | -0.18093 | 0.035528 | 0.035528 | Downregulated |
| PLEKHO1 | 204.5275229 | 180.4201183 | -0.18093 | 0.035528 | 0.035528 | Downregulated |
| SLC1A1 | 204.5183486 | 180.4319527 | -0.18078 | 0.035689 | 0.035689 | Downregulated |
| RSBN1 | 204.5137615 | 180.4378698 | -0.1807 | 0.03577 | 0.03577 | Downregulated |
| OBSCN | 204.5137615 | 180.4378698 | -0.1807 | 0.03577 | 0.03577 | Downregulated |
| ARL2BP | 204.5137615 | 180.4378698 | -0.1807 | 0.03577 | 0.03577 | Downregulated |
| ABCA5 | 204.5137615 | 180.4378698 | -0.1807 | 0.03577 | 0.03577 | Downregulated |
| CEACAM3 | 200.3211009 | 176.7455621 | -0.18064 | 0.039649 | 0.039649 | Downregulated |
| TLK2 | 204.5091743 | 180.443787 | -0.18062 | 0.035851 | 0.035851 | Downregulated |
| HCN3 | 204.5091743 | 180.443787 | -0.18062 | 0.035851 | 0.035851 | Downregulated |
| LDAH | 204.5091743 | 180.443787 | -0.18062 | 0.035851 | 0.035851 | Downregulated |
| PXK | 204.5045872 | 180.4497041 | -0.18054 | 0.035931 | 0.035931 | Downregulated |
| B3GALNT1 | 204.5045872 | 180.4497041 | -0.18054 | 0.035931 | 0.035931 | Downregulated |
| JAZF1 | 204.5 | 180.4556213 | -0.18046 | 0.036013 | 0.036013 | Downregulated |
| APOBEC3G | 204.4954128 | 180.4615385 | -0.18038 | 0.036094 | 0.036094 | Downregulated |
| KAT2A | 204.4908257 | 180.4674556 | -0.1803 | 0.036175 | 0.036175 | Downregulated |
| FCGR1A | 204.4908257 | 180.4674556 | -0.1803 | 0.036175 | 0.036175 | Downregulated |
| VIP | 197.1880734 | 174.0236686 | -0.18029 | 0.042881 | 0.042881 | Downregulated |
| ZNF781 | 192.0458716 | 169.5029586 | -0.18014 | 0.049746 | 0.049746 | Downregulated |
| FZD4 | 204.4816514 | 180.4792899 | -0.18014 | 0.036338 | 0.036338 | Downregulated |
| ZNF626 | 201.3348624 | 177.704142 | -0.18012 | 0.039298 | 0.039298 | Downregulated |
| PDE8A | 204.4770642 | 180.4852071 | -0.18006 | 0.03642 | 0.03642 | Downregulated |
| ZBTB24 | 204.4770642 | 180.4852071 | -0.18006 | 0.03642 | 0.03642 | Downregulated |
| CCNI | 204.4724771 | 180.4911243 | -0.17998 | 0.036502 | 0.036502 | Downregulated |
| FNIP1 | 204.1651376 | 180.2248521 | -0.17994 | 0.036013 | 0.036013 | Downregulated |
| RAB25 | 204.4678899 | 180.4970414 | -0.1799 | 0.036584 | 0.036584 | Downregulated |
| MPEG1 | 204.4541284 | 180.5147929 | -0.17966 | 0.036832 | 0.036832 | Downregulated |
| POT1 | 204.4541284 | 180.5147929 | -0.17966 | 0.036832 | 0.036832 | Downregulated |
| MAP9 | 202.353211 | 178.6627219 | -0.17964 | 0.038863 | 0.038863 | Downregulated |
| MISP3 | 204.4495413 | 180.5207101 | -0.17958 | 0.036915 | 0.036915 | Downregulated |
| FAM69A | 204.4495413 | 180.5207101 | -0.17958 | 0.036915 | 0.036915 | Downregulated |
| F8 | 204.4449541 | 180.5266272 | -0.1795 | 0.036998 | 0.036998 | Downregulated |
| TM9SF4 | 204.440367 | 180.5325444 | -0.17942 | 0.037081 | 0.037081 | Downregulated |
| ERCC6L2 | 204.440367 | 180.5325444 | -0.17942 | 0.037081 | 0.037081 | Downregulated |
| GTF3C4 | 204.440367 | 180.5325444 | -0.17942 | 0.037081 | 0.037081 | Downregulated |
| TSSK4 | 201.2844037 | 177.7692308 | -0.17923 | 0.040136 | 0.040136 | Downregulated |
| DECR1 | 204.4220183 | 180.556213 | -0.1791 | 0.037415 | 0.037415 | Downregulated |
| LILRB3 | 204.4174312 | 180.5621302 | -0.17902 | 0.037499 | 0.037499 | Downregulated |
| CCR5 | 204.412844 | 180.5680473 | -0.17894 | 0.037583 | 0.037583 | Downregulated |
| GZMK | 195.0504587 | 172.3017751 | -0.17891 | 0.047449 | 0.047449 | Downregulated |
| TNFRSF1A | 204.4082569 | 180.5739645 | -0.17886 | 0.037667 | 0.037667 | Downregulated |
| ZNF366 | 201.1238532 | 177.6804734 | -0.1788 | 0.040583 | 0.040583 | Downregulated |
| MCM9 | 204.4036697 | 180.5798817 | -0.17878 | 0.037751 | 0.037751 | Downregulated |
| QRICH1 | 204.4036697 | 180.5798817 | -0.17878 | 0.037751 | 0.037751 | Downregulated |
| TAL1 | 204.3990826 | 180.5857988 | -0.1787 | 0.037836 | 0.037836 | Downregulated |
| TAF1B | 204.3990826 | 180.5857988 | -0.1787 | 0.037836 | 0.037836 | Downregulated |
| TAX1BP1 | 204.3990826 | 180.5857988 | -0.1787 | 0.037836 | 0.037836 | Downregulated |
| SOD1 | 204.3944954 | 180.591716 | -0.17862 | 0.037921 | 0.037921 | Downregulated |
| XRCC4 | 204.3944954 | 180.591716 | -0.17862 | 0.037921 | 0.037921 | Downregulated |
| PRKCD | 203.6376147 | 179.9230769 | -0.17862 | 0.038133 | 0.038133 | Downregulated |
| BCL2L1 | 204.3899083 | 180.5976331 | -0.17854 | 0.038005 | 0.038005 | Downregulated |
| KL | 202.2844037 | 178.7514793 | -0.17843 | 0.04018 | 0.04018 | Downregulated |
| SRI | 204.3807339 | 180.6094675 | -0.17839 | 0.038176 | 0.038176 | Downregulated |
| ANAPC10 | 204.3807339 | 180.6094675 | -0.17839 | 0.038176 | 0.038176 | Downregulated |
| GNLY | 204.3807339 | 180.6094675 | -0.17839 | 0.038176 | 0.038176 | Downregulated |
| PON3 | 201.233945 | 177.8343195 | -0.17834 | 0.041398 | 0.041398 | Downregulated |
| RAB33B | 204.3761468 | 180.6153846 | -0.17831 | 0.038261 | 0.038261 | Downregulated |
| ZC3H7A | 204.3761468 | 180.6153846 | -0.17831 | 0.038261 | 0.038261 | Downregulated |
| ARL6 | 204.3715596 | 180.6213018 | -0.17823 | 0.038347 | 0.038347 | Downregulated |
| CCDC58 | 204.3715596 | 180.6213018 | -0.17823 | 0.038347 | 0.038347 | Downregulated |
| CTSH | 204.3715596 | 180.6213018 | -0.17823 | 0.038347 | 0.038347 | Downregulated |
| RERE | 204.3669725 | 180.6272189 | -0.17815 | 0.038432 | 0.038432 | Downregulated |
| FAM204A | 204.3669725 | 180.6272189 | -0.17815 | 0.038432 | 0.038432 | Downregulated |
| C11orf74 | 204.3669725 | 180.6272189 | -0.17815 | 0.038432 | 0.038432 | Downregulated |
| CLCN6 | 204.3669725 | 180.6272189 | -0.17815 | 0.038432 | 0.038432 | Downregulated |
| PRR14L | 204.3623853 | 180.6331361 | -0.17807 | 0.038518 | 0.038518 | Downregulated |
| CEP128 | 204.3623853 | 180.6331361 | -0.17807 | 0.038518 | 0.038518 | Downregulated |
| ZNF396 | 204.3623853 | 180.6331361 | -0.17807 | 0.038518 | 0.038518 | Downregulated |
| IFT20 | 204.3623853 | 180.6331361 | -0.17807 | 0.038518 | 0.038518 | Downregulated |
| EMC7 | 204.3577982 | 180.6390533 | -0.17799 | 0.038604 | 0.038604 | Downregulated |
| ZNF484 | 204.3577982 | 180.6390533 | -0.17799 | 0.038604 | 0.038604 | Downregulated |
| EVI5 | 204.3577982 | 180.6390533 | -0.17799 | 0.038604 | 0.038604 | Downregulated |
| HNRNPA2B1 | 204.353211 | 180.6449704 | -0.17791 | 0.03869 | 0.03869 | Downregulated |
| FAM168B | 204.3440367 | 180.6568047 | -0.17775 | 0.038863 | 0.038863 | Downregulated |
| DSCC1 | 204.3440367 | 180.6568047 | -0.17775 | 0.038863 | 0.038863 | Downregulated |
| JAKMIP1 | 199.1146789 | 176.0414201 | -0.17768 | 0.044604 | 0.044604 | Downregulated |
| BAZ2A | 204.3394495 | 180.6627219 | -0.17767 | 0.03895 | 0.03895 | Downregulated |
| C12orf4 | 204.3394495 | 180.6627219 | -0.17767 | 0.03895 | 0.03895 | Downregulated |
| DCUN1D1 | 204.3394495 | 180.6627219 | -0.17767 | 0.03895 | 0.03895 | Downregulated |
| PRPF39 | 204.3348624 | 180.6686391 | -0.17759 | 0.039037 | 0.039037 | Downregulated |
| NSMCE1 | 204.3348624 | 180.6686391 | -0.17759 | 0.039037 | 0.039037 | Downregulated |
| LARGE1 | 204.3256881 | 180.6804734 | -0.17743 | 0.039211 | 0.039211 | Downregulated |
| PDGFC | 204.3211009 | 180.6863905 | -0.17735 | 0.039298 | 0.039298 | Downregulated |
| CEP85L | 204.3211009 | 180.6863905 | -0.17735 | 0.039298 | 0.039298 | Downregulated |
| KBTBD3 | 204.3211009 | 180.6863905 | -0.17735 | 0.039298 | 0.039298 | Downregulated |
| AMZ1 | 195.9908257 | 173.3254438 | -0.1773 | 0.048067 | 0.048067 | Downregulated |
| TSPAN18 | 204.3165138 | 180.6923077 | -0.17727 | 0.039385 | 0.039385 | Downregulated |
| METTL14 | 204.3165138 | 180.6923077 | -0.17727 | 0.039385 | 0.039385 | Downregulated |
| FRK | 203.5321101 | 180 | -0.17726 | 0.039958 | 0.039958 | Downregulated |
| SPCS3 | 204.3119266 | 180.6982249 | -0.17719 | 0.039473 | 0.039473 | Downregulated |
| PDE6D | 204.3119266 | 180.6982249 | -0.17719 | 0.039473 | 0.039473 | Downregulated |
| PPP2R2B | 197.0045872 | 174.260355 | -0.17698 | 0.047501 | 0.047501 | Downregulated |
| PPP1R3G | 204.2981651 | 180.7159763 | -0.17695 | 0.039737 | 0.039737 | Downregulated |
| WDSUB1 | 204.293578 | 180.7218935 | -0.17687 | 0.039825 | 0.039825 | Downregulated |
| PAICS | 204.293578 | 180.7218935 | -0.17687 | 0.039825 | 0.039825 | Downregulated |
| IFI30 | 204.2889908 | 180.7278107 | -0.17679 | 0.039914 | 0.039914 | Downregulated |
| EPHX1 | 204.2889908 | 180.7278107 | -0.17679 | 0.039914 | 0.039914 | Downregulated |
| ASB13 | 204.2844037 | 180.7337278 | -0.17671 | 0.040002 | 0.040002 | Downregulated |
| TACC3 | 204.2844037 | 180.7337278 | -0.17671 | 0.040002 | 0.040002 | Downregulated |
| GFOD1 | 204.2844037 | 180.7337278 | -0.17671 | 0.040002 | 0.040002 | Downregulated |
| TBC1D24 | 204.2844037 | 180.7337278 | -0.17671 | 0.040002 | 0.040002 | Downregulated |
| KRBA1 | 204.2844037 | 180.7337278 | -0.17671 | 0.040002 | 0.040002 | Downregulated |
| FSD1 | 204.2798165 | 180.739645 | -0.17663 | 0.040091 | 0.040091 | Downregulated |
| UBE2L3 | 204.2798165 | 180.739645 | -0.17663 | 0.040091 | 0.040091 | Downregulated |
| ZKSCAN3 | 204.2798165 | 180.739645 | -0.17663 | 0.040091 | 0.040091 | Downregulated |
| ZNF428 | 204.2752294 | 180.7455621 | -0.17655 | 0.04018 | 0.04018 | Downregulated |
| DPEP2 | 204.2706422 | 180.7514793 | -0.17647 | 0.040269 | 0.040269 | Downregulated |
| COX7C | 204.2706422 | 180.7514793 | -0.17647 | 0.040269 | 0.040269 | Downregulated |
| PPP2R5E | 204.2706422 | 180.7514793 | -0.17647 | 0.040269 | 0.040269 | Downregulated |
| TWIST2 | 202.1651376 | 178.9053254 | -0.17634 | 0.042553 | 0.042553 | Downregulated |
| RHOQ | 204.2614679 | 180.7633136 | -0.17632 | 0.040448 | 0.040448 | Downregulated |
| ZNF300 | 203.2110092 | 179.8343195 | -0.17631 | 0.041444 | 0.041444 | Downregulated |
| RETREG2 | 202.5688073 | 179.2721893 | -0.17626 | 0.041673 | 0.041673 | Downregulated |
| LIN9 | 204.2568807 | 180.7692308 | -0.17624 | 0.040538 | 0.040538 | Downregulated |
| SLC9A5 | 204.2522936 | 180.7751479 | -0.17616 | 0.040628 | 0.040628 | Downregulated |
| SMAD4 | 204.2477064 | 180.7810651 | -0.17608 | 0.040718 | 0.040718 | Downregulated |
| ACKR2 | 203.1972477 | 179.852071 | -0.17607 | 0.041765 | 0.041765 | Downregulated |
| DDN | 204.2431193 | 180.7869822 | -0.176 | 0.040808 | 0.040808 | Downregulated |
| ZNF20 | 200.0458716 | 177.1005917 | -0.17576 | 0.04524 | 0.04524 | Downregulated |
| ZNF701 | 204.2293578 | 180.8047337 | -0.17576 | 0.041079 | 0.041079 | Downregulated |
| ADAMTS6 | 204.2293578 | 180.8047337 | -0.17576 | 0.041079 | 0.041079 | Downregulated |
| TPCN2 | 204.2293578 | 180.8047337 | -0.17576 | 0.041079 | 0.041079 | Downregulated |
| ZKSCAN7 | 201.087156 | 178.0236686 | -0.17575 | 0.04441 | 0.04441 | Downregulated |
| DYSF | 204.2247706 | 180.8106509 | -0.17568 | 0.04117 | 0.04117 | Downregulated |
| KIAA0513 | 204.2247706 | 180.8106509 | -0.17568 | 0.04117 | 0.04117 | Downregulated |
| DDX59 | 204.2247706 | 180.8106509 | -0.17568 | 0.04117 | 0.04117 | Downregulated |
| VWF | 204.2247706 | 180.8106509 | -0.17568 | 0.04117 | 0.04117 | Downregulated |
| MUM1 | 204.2247706 | 180.8106509 | -0.17568 | 0.04117 | 0.04117 | Downregulated |
| AOC2 | 204.2201835 | 180.816568 | -0.1756 | 0.041261 | 0.041261 | Downregulated |
| BIRC7 | 197.9587156 | 175.2781065 | -0.17555 | 0.048276 | 0.048276 | Downregulated |
| SPG20 | 204.2155963 | 180.8224852 | -0.17552 | 0.041352 | 0.041352 | Downregulated |
| CES1 | 204.2155963 | 180.8224852 | -0.17552 | 0.041352 | 0.041352 | Downregulated |
| SMIM1 | 200.0321101 | 177.1183432 | -0.17552 | 0.046133 | 0.046133 | Downregulated |
| SLC45A4 | 204.2110092 | 180.8284024 | -0.17544 | 0.041444 | 0.041444 | Downregulated |
| GOLGA8B | 204.2018349 | 180.8402367 | -0.17528 | 0.041627 | 0.041627 | Downregulated |
| PARP11 | 204.2018349 | 180.8402367 | -0.17528 | 0.041627 | 0.041627 | Downregulated |
| AFAP1L1 | 204.2018349 | 180.8402367 | -0.17528 | 0.041627 | 0.041627 | Downregulated |
| EPC2 | 204.2018349 | 180.8402367 | -0.17528 | 0.041627 | 0.041627 | Downregulated |
| FAM160B1 | 204.2018349 | 180.8402367 | -0.17528 | 0.041627 | 0.041627 | Downregulated |
| SUMF2 | 204.1972477 | 180.8461538 | -0.1752 | 0.041719 | 0.041719 | Downregulated |
| CCNL1 | 204.1972477 | 180.8461538 | -0.1752 | 0.041719 | 0.041719 | Downregulated |
| NUP93 | 204.1972477 | 180.8461538 | -0.1752 | 0.041719 | 0.041719 | Downregulated |
| ZNF148 | 204.1880734 | 180.8579882 | -0.17504 | 0.041903 | 0.041903 | Downregulated |
| PDIA6 | 204.1880734 | 180.8579882 | -0.17504 | 0.041903 | 0.041903 | Downregulated |
| FNDC11 | 203.1376147 | 179.9289941 | -0.17503 | 0.042976 | 0.042976 | Downregulated |
| RAB29 | 204.1834862 | 180.8639053 | -0.17496 | 0.041995 | 0.041995 | Downregulated |
| TESK2 | 204.1834862 | 180.8639053 | -0.17496 | 0.041995 | 0.041995 | Downregulated |
| TCTE3 | 204.1788991 | 180.8698225 | -0.17488 | 0.042088 | 0.042088 | Downregulated |
| DYRK3 | 204.1697248 | 180.8816568 | -0.17472 | 0.042274 | 0.042274 | Downregulated |
| VNN1 | 204.1697248 | 180.8816568 | -0.17472 | 0.042274 | 0.042274 | Downregulated |
| NEK11 | 204.1697248 | 180.8816568 | -0.17472 | 0.042274 | 0.042274 | Downregulated |
| C17orf100 | 204.1697248 | 180.8816568 | -0.17472 | 0.042274 | 0.042274 | Downregulated |
| GAS1 | 204.1697248 | 180.8816568 | -0.17472 | 0.042274 | 0.042274 | Downregulated |
| CDADC1 | 204.1651376 | 180.887574 | -0.17464 | 0.042367 | 0.042367 | Downregulated |
| CFP | 204.1605505 | 180.8934911 | -0.17456 | 0.04246 | 0.04246 | Downregulated |
| PDZD3 | 204.1605505 | 180.8934911 | -0.17456 | 0.04246 | 0.04246 | Downregulated |
| GCNT2 | 204.1559633 | 180.8994083 | -0.17448 | 0.042553 | 0.042553 | Downregulated |
| GGCX | 204.1559633 | 180.8994083 | -0.17448 | 0.042553 | 0.042553 | Downregulated |
| ENTPD7 | 204.1559633 | 180.8994083 | -0.17448 | 0.042553 | 0.042553 | Downregulated |
| SOCS4 | 204.1513761 | 180.9053254 | -0.1744 | 0.042647 | 0.042647 | Downregulated |
| TFAP2D | 1.128440367 | 1 | -0.17433 | 0.018991 | 0.018991 | Downregulated |
| ATXN3 | 204.146789 | 180.9112426 | -0.17432 | 0.042741 | 0.042741 | Downregulated |
| CDH6 | 204.1422018 | 180.9171598 | -0.17425 | 0.042835 | 0.042835 | Downregulated |
| WWOX | 204.1422018 | 180.9171598 | -0.17425 | 0.042835 | 0.042835 | Downregulated |
| JMY | 204.1422018 | 180.9171598 | -0.17425 | 0.042835 | 0.042835 | Downregulated |
| SLC2A3 | 204.1376147 | 180.9230769 | -0.17417 | 0.042929 | 0.042929 | Downregulated |
| SIAE | 204.1330275 | 180.9289941 | -0.17409 | 0.043023 | 0.043023 | Downregulated |
| FBXO30 | 204.1284404 | 180.9349112 | -0.17401 | 0.043117 | 0.043117 | Downregulated |
| F2RL3 | 204.1284404 | 180.9349112 | -0.17401 | 0.043117 | 0.043117 | Downregulated |
| TMIGD3 | 204.1284404 | 180.9349112 | -0.17401 | 0.043117 | 0.043117 | Downregulated |
| TCF21 | 204.1284404 | 180.9349112 | -0.17401 | 0.043117 | 0.043117 | Downregulated |
| BLM | 204.1238532 | 180.9408284 | -0.17393 | 0.043212 | 0.043212 | Downregulated |
| PPM1M | 204.1238532 | 180.9408284 | -0.17393 | 0.043212 | 0.043212 | Downregulated |
| SELP | 198.9036697 | 176.3136095 | -0.17393 | 0.048746 | 0.048746 | Downregulated |
| BIRC3 | 203.5550459 | 180.4497041 | -0.17382 | 0.0426 | 0.0426 | Downregulated |
| TMEM47 | 204.1146789 | 180.9526627 | -0.17377 | 0.043402 | 0.043402 | Downregulated |
| ZNF501 | 204.1100917 | 180.9585799 | -0.17369 | 0.043497 | 0.043497 | Downregulated |
| HACL1 | 204.1100917 | 180.9585799 | -0.17369 | 0.043497 | 0.043497 | Downregulated |
| USP28 | 204.1100917 | 180.9585799 | -0.17369 | 0.043497 | 0.043497 | Downregulated |
| ZNF782 | 204.1055046 | 180.964497 | -0.17361 | 0.043592 | 0.043592 | Downregulated |
| ATG16L1 | 204.1055046 | 180.964497 | -0.17361 | 0.043592 | 0.043592 | Downregulated |
| JADE1 | 204.1009174 | 180.9704142 | -0.17353 | 0.043688 | 0.043688 | Downregulated |
| FBXO32 | 204.1009174 | 180.9704142 | -0.17353 | 0.043688 | 0.043688 | Downregulated |
| USP51 | 199.9174312 | 177.2662722 | -0.17349 | 0.048069 | 0.048069 | Downregulated |
| CCDC80 | 204.0917431 | 180.9822485 | -0.17337 | 0.043879 | 0.043879 | Downregulated |
| DUS4L | 204.0917431 | 180.9822485 | -0.17337 | 0.043879 | 0.043879 | Downregulated |
| GRAMD4 | 204.0917431 | 180.9822485 | -0.17337 | 0.043879 | 0.043879 | Downregulated |
| GLT8D2 | 204.087156 | 180.9881657 | -0.17329 | 0.043975 | 0.043975 | Downregulated |
| NIP7 | 204.087156 | 180.9881657 | -0.17329 | 0.043975 | 0.043975 | Downregulated |
| ZSCAN12 | 204.0825688 | 180.9940828 | -0.17321 | 0.044072 | 0.044072 | Downregulated |
| COL13A1 | 204.0825688 | 180.9940828 | -0.17321 | 0.044072 | 0.044072 | Downregulated |
| C20orf194 | 204.0779817 | 181 | -0.17313 | 0.044168 | 0.044168 | Downregulated |
| ZSCAN31 | 204.0779817 | 181 | -0.17313 | 0.044168 | 0.044168 | Downregulated |
| CA11 | 204.0733945 | 181.0059172 | -0.17305 | 0.044265 | 0.044265 | Downregulated |
| UNC5C | 204.0733945 | 181.0059172 | -0.17305 | 0.044265 | 0.044265 | Downregulated |
| HINT2 | 204.0733945 | 181.0059172 | -0.17305 | 0.044265 | 0.044265 | Downregulated |
| CEP135 | 204.059633 | 181.0236686 | -0.17281 | 0.044555 | 0.044555 | Downregulated |
| SLC29A1 | 204.059633 | 181.0236686 | -0.17281 | 0.044555 | 0.044555 | Downregulated |
| CSF1 | 204.059633 | 181.0236686 | -0.17281 | 0.044555 | 0.044555 | Downregulated |
| ABI3 | 204.0550459 | 181.0295858 | -0.17273 | 0.044653 | 0.044653 | Downregulated |
| STX16 | 204.0504587 | 181.035503 | -0.17265 | 0.04475 | 0.04475 | Downregulated |
| LRRN4CL | 204.0504587 | 181.035503 | -0.17265 | 0.04475 | 0.04475 | Downregulated |
| MYO5B | 204.0504587 | 181.035503 | -0.17265 | 0.04475 | 0.04475 | Downregulated |
| JAK1 | 204.0458716 | 181.0414201 | -0.17257 | 0.044848 | 0.044848 | Downregulated |
| ARFGAP1 | 203.266055 | 180.3846154 | -0.17229 | 0.045536 | 0.045536 | Downregulated |
| NPHP4 | 204.0229358 | 181.0710059 | -0.17218 | 0.045339 | 0.045339 | Downregulated |
| PIGL | 204.0183486 | 181.0769231 | -0.1721 | 0.045437 | 0.045437 | Downregulated |
| METTL27 | 204.0183486 | 181.0769231 | -0.1721 | 0.045437 | 0.045437 | Downregulated |
| RMDN2 | 204.0137615 | 181.0828402 | -0.17202 | 0.045536 | 0.045536 | Downregulated |
| MDGA1 | 204.0091743 | 181.0887574 | -0.17194 | 0.045635 | 0.045635 | Downregulated |
| MAP3K2 | 204.0091743 | 181.0887574 | -0.17194 | 0.045635 | 0.045635 | Downregulated |
| FMO3 | 201.912844 | 179.2307692 | -0.17191 | 0.047862 | 0.047862 | Downregulated |
| TNFSF11 | 204.0045872 | 181.0946746 | -0.17186 | 0.045735 | 0.045735 | Downregulated |
| BCKDK | 203.1972477 | 180.3786982 | -0.17185 | 0.045685 | 0.045685 | Downregulated |
| KAZN | 204 | 181.1005917 | -0.17178 | 0.045834 | 0.045834 | Downregulated |
| ICAM2 | 204 | 181.1005917 | -0.17178 | 0.045834 | 0.045834 | Downregulated |
| SLC29A2 | 204 | 181.1005917 | -0.17178 | 0.045834 | 0.045834 | Downregulated |
| EIF4ENIF1 | 204 | 181.1005917 | -0.17178 | 0.045834 | 0.045834 | Downregulated |
| P2RX1 | 203.9954128 | 181.1065089 | -0.1717 | 0.045934 | 0.045934 | Downregulated |
| MT-CO3 | 203.9908257 | 181.112426 | -0.17162 | 0.046034 | 0.046034 | Downregulated |
| FRS2 | 203.9862385 | 181.1183432 | -0.17154 | 0.046134 | 0.046134 | Downregulated |
| NDNF | 203.9862385 | 181.1183432 | -0.17154 | 0.046134 | 0.046134 | Downregulated |
| PPP1R3C | 203.9816514 | 181.1242604 | -0.17146 | 0.046234 | 0.046234 | Downregulated |
| RSRP1 | 203.9816514 | 181.1242604 | -0.17146 | 0.046234 | 0.046234 | Downregulated |
| MRAS | 203.9816514 | 181.1242604 | -0.17146 | 0.046234 | 0.046234 | Downregulated |
| ZNF738 | 203.9816514 | 181.1242604 | -0.17146 | 0.046234 | 0.046234 | Downregulated |
| FILIP1 | 203.9770642 | 181.1301775 | -0.17138 | 0.046334 | 0.046334 | Downregulated |
| MRPL53 | 203.9770642 | 181.1301775 | -0.17138 | 0.046334 | 0.046334 | Downregulated |
| PDIK1L | 203.9770642 | 181.1301775 | -0.17138 | 0.046334 | 0.046334 | Downregulated |
| VGLL3 | 203.9724771 | 181.1360947 | -0.1713 | 0.046435 | 0.046435 | Downregulated |
| TNF | 202.9220183 | 180.2071006 | -0.17127 | 0.047604 | 0.047604 | Downregulated |
| CCNG2 | 203.9678899 | 181.1420118 | -0.17122 | 0.046535 | 0.046535 | Downregulated |
| ATL1 | 203.9633028 | 181.147929 | -0.17114 | 0.046636 | 0.046636 | Downregulated |
| TBC1D8B | 203.9633028 | 181.147929 | -0.17114 | 0.046636 | 0.046636 | Downregulated |
| ENTPD6 | 203.9587156 | 181.1538462 | -0.17106 | 0.046738 | 0.046738 | Downregulated |
| SUSD2 | 203.9587156 | 181.1538462 | -0.17106 | 0.046738 | 0.046738 | Downregulated |
| SFXN5 | 203.9587156 | 181.1538462 | -0.17106 | 0.046738 | 0.046738 | Downregulated |
| SHISA4 | 203.9587156 | 181.1538462 | -0.17106 | 0.046738 | 0.046738 | Downregulated |
| BBOF1 | 203.9587156 | 181.1538462 | -0.17106 | 0.046738 | 0.046738 | Downregulated |
| CDC42EP2 | 203.9449541 | 181.1715976 | -0.17082 | 0.047042 | 0.047042 | Downregulated |
| YEATS2 | 203.9449541 | 181.1715976 | -0.17082 | 0.047042 | 0.047042 | Downregulated |
| POLR1C | 203.940367 | 181.1775148 | -0.17074 | 0.047144 | 0.047144 | Downregulated |
| MITD1 | 203.9357798 | 181.183432 | -0.17066 | 0.047246 | 0.047246 | Downregulated |
| SMARCAD1 | 203.9311927 | 181.1893491 | -0.17058 | 0.047348 | 0.047348 | Downregulated |
| NXT1 | 203.9311927 | 181.1893491 | -0.17058 | 0.047348 | 0.047348 | Downregulated |
| NR2C2 | 203.9266055 | 181.1952663 | -0.1705 | 0.04745 | 0.04745 | Downregulated |
| ANKRD28 | 203.9266055 | 181.1952663 | -0.1705 | 0.04745 | 0.04745 | Downregulated |
| PXMP2 | 203.9220183 | 181.2011834 | -0.17043 | 0.047553 | 0.047553 | Downregulated |
| ZNF736 | 203.9220183 | 181.2011834 | -0.17043 | 0.047553 | 0.047553 | Downregulated |
| ABI1 | 203.9174312 | 181.2071006 | -0.17035 | 0.047656 | 0.047656 | Downregulated |
| ETHE1 | 203.9174312 | 181.2071006 | -0.17035 | 0.047656 | 0.047656 | Downregulated |
| NDFIP2 | 203.9174312 | 181.2071006 | -0.17035 | 0.047656 | 0.047656 | Downregulated |
| CAVIN2 | 203.912844 | 181.2130178 | -0.17027 | 0.047759 | 0.047759 | Downregulated |
| ZNF680 | 203.9036697 | 181.2248521 | -0.17011 | 0.047965 | 0.047965 | Downregulated |
| GIN1 | 203.9036697 | 181.2248521 | -0.17011 | 0.047965 | 0.047965 | Downregulated |
| ZNF335 | 203.9036697 | 181.2248521 | -0.17011 | 0.047965 | 0.047965 | Downregulated |
| ADAMTS2 | 203.8990826 | 181.2307692 | -0.17003 | 0.048069 | 0.048069 | Downregulated |
| ZBTB26 | 203.8990826 | 181.2307692 | -0.17003 | 0.048069 | 0.048069 | Downregulated |
| TMEM200B | 203.8853211 | 181.2485207 | -0.16979 | 0.04838 | 0.04838 | Downregulated |
| B4GALT4 | 203.8853211 | 181.2485207 | -0.16979 | 0.04838 | 0.04838 | Downregulated |
| CEP57 | 203.8853211 | 181.2485207 | -0.16979 | 0.04838 | 0.04838 | Downregulated |
| VPS8 | 203.8853211 | 181.2485207 | -0.16979 | 0.04838 | 0.04838 | Downregulated |
| ZCCHC4 | 203.8807339 | 181.2544379 | -0.16971 | 0.048485 | 0.048485 | Downregulated |
| MSANTD2 | 203.8807339 | 181.2544379 | -0.16971 | 0.048485 | 0.048485 | Downregulated |
| IL32 | 203.8807339 | 181.2544379 | -0.16971 | 0.048485 | 0.048485 | Downregulated |
| FGD5 | 203.8807339 | 181.2544379 | -0.16971 | 0.048485 | 0.048485 | Downregulated |
| USP8 | 203.8761468 | 181.260355 | -0.16963 | 0.048589 | 0.048589 | Downregulated |
| EGFR | 203.8715596 | 181.2662722 | -0.16955 | 0.048694 | 0.048694 | Downregulated |
| DDC | 203.8715596 | 181.2662722 | -0.16955 | 0.048694 | 0.048694 | Downregulated |
| TBXA2R | 203.8669725 | 181.2721893 | -0.16947 | 0.048799 | 0.048799 | Downregulated |
| SMIM13 | 203.8669725 | 181.2721893 | -0.16947 | 0.048799 | 0.048799 | Downregulated |
| UBE2A | 203.8669725 | 181.2721893 | -0.16947 | 0.048799 | 0.048799 | Downregulated |
| TRAPPC8 | 203.8577982 | 181.2840237 | -0.16931 | 0.049009 | 0.049009 | Downregulated |
| YY1 | 203.853211 | 181.2899408 | -0.16923 | 0.049114 | 0.049114 | Downregulated |
| CSNK1G1 | 203.8486239 | 181.295858 | -0.16915 | 0.04922 | 0.04922 | Downregulated |
| TMPO | 203.8486239 | 181.295858 | -0.16915 | 0.04922 | 0.04922 | Downregulated |
| BVES | 203.8440367 | 181.3017751 | -0.16907 | 0.049326 | 0.049326 | Downregulated |
| IFIT1 | 203.8440367 | 181.3017751 | -0.16907 | 0.049326 | 0.049326 | Downregulated |
| KCTD18 | 203.8440367 | 181.3017751 | -0.16907 | 0.049326 | 0.049326 | Downregulated |
| PPP1R16B | 203.8394495 | 181.3076923 | -0.16899 | 0.049432 | 0.049432 | Downregulated |
| SELE | 203.8394495 | 181.3076923 | -0.16899 | 0.049432 | 0.049432 | Downregulated |
| NTNG2 | 203.8348624 | 181.3136095 | -0.16891 | 0.049538 | 0.049538 | Downregulated |
| LRRC25 | 203.8348624 | 181.3136095 | -0.16891 | 0.049538 | 0.049538 | Downregulated |
| ORMDL3 | 203.8302752 | 181.3195266 | -0.16883 | 0.049644 | 0.049644 | Downregulated |
| PATL2 | 203.3669725 | 180.9467456 | -0.16852 | 0.049751 | 0.049751 | Downregulated |
| ZNF645 | 1.096330275 | 1 | -0.13268 | 0.030114 | 0.030114 | Downregulated |
| GIMD1 | 1.068807339 | 1 | -0.096 | 0.048078 | 0.048078 | Downregulated |
| OR6N1 | 1 | 1.035502959 | 0.050332 | 0.048922 | 0.048922 | Upregulated |
| LCE3C | 1 | 1.035502959 | 0.050332 | 0.048922 | 0.048922 | Upregulated |
| OR7G3 | 1 | 1.035502959 | 0.050332 | 0.048922 | 0.048922 | Upregulated |
| OR5AP2 | 1 | 1.035502959 | 0.050332 | 0.048922 | 0.048922 | Upregulated |
| OR6C74 | 1 | 1.035502959 | 0.050332 | 0.048922 | 0.048922 | Upregulated |
| TMOD4 | 1 | 1.035502959 | 0.050332 | 0.048922 | 0.048922 | Upregulated |
| OR2T1 | 1 | 1.035502959 | 0.050332 | 0.048922 | 0.048922 | Upregulated |
| KRTAP9-7 | 1 | 1.035502959 | 0.050332 | 0.048922 | 0.048922 | Upregulated |
| PRAMEF19 | 1 | 1.059171598 | 0.082936 | 0.022742 | 0.022742 | Upregulated |
| KRTAP2-1 | 1 | 1.059171598 | 0.082936 | 0.022742 | 0.022742 | Upregulated |
| OR12D3 | 1.02293578 | 1.094674556 | 0.097786 | 0.049638 | 0.049638 | Upregulated |
| OR2Z1 | 1.02293578 | 1.094674556 | 0.097786 | 0.049638 | 0.049638 | Upregulated |
| C11orf40 | 1.018348624 | 1.100591716 | 0.112048 | 0.049143 | 0.049143 | Upregulated |
| KRTAP9-8 | 1 | 1.088757396 | 0.122683 | 0.010758 | 0.010758 | Upregulated |
| SPANXN1 | 1 | 1.088757396 | 0.122683 | 0.010758 | 0.010758 | Upregulated |
| PRAMEF18 | 1.009174312 | 1.112426036 | 0.140534 | 0.048164 | 0.048164 | Upregulated |
| SPDEF | 184.1788991 | 206.6686391 | 0.166212 | 0.049858 | 0.049858 | Upregulated |
| FAM220A | 184.1788991 | 206.6686391 | 0.166212 | 0.049858 | 0.049858 | Upregulated |
| APBA1 | 184.1743119 | 206.6745562 | 0.166289 | 0.049751 | 0.049751 | Upregulated |
| MSRA | 184.1697248 | 206.6804734 | 0.166366 | 0.049644 | 0.049644 | Upregulated |
| ILDR2 | 184.1513761 | 206.704142 | 0.166675 | 0.04922 | 0.04922 | Upregulated |
| AMOT | 184.1422018 | 206.7159763 | 0.16683 | 0.049009 | 0.049009 | Upregulated |
| ADH6 | 184.1422018 | 206.7159763 | 0.16683 | 0.049009 | 0.049009 | Upregulated |
| KCMF1 | 184.1422018 | 206.7159763 | 0.16683 | 0.049009 | 0.049009 | Upregulated |
| FYCO1 | 184.1238532 | 206.739645 | 0.167139 | 0.048589 | 0.048589 | Upregulated |
| FAM20B | 184.1146789 | 206.7514793 | 0.167293 | 0.04838 | 0.04838 | Upregulated |
| SLC13A4 | 184.1146789 | 206.7514793 | 0.167293 | 0.04838 | 0.04838 | Upregulated |
| PRSS33 | 183.1651376 | 205.6923077 | 0.167343 | 0.049485 | 0.049485 | Upregulated |
| OTUB2 | 184.1055046 | 206.7633136 | 0.167447 | 0.048172 | 0.048172 | Upregulated |
| CEP89 | 184.1055046 | 206.7633136 | 0.167447 | 0.048172 | 0.048172 | Upregulated |
| MYCL | 184.1055046 | 206.7633136 | 0.167447 | 0.048172 | 0.048172 | Upregulated |
| SCG5 | 184.0963303 | 206.7751479 | 0.167602 | 0.047965 | 0.047965 | Upregulated |
| CBFA2T3 | 184.0825688 | 206.7928994 | 0.167834 | 0.047656 | 0.047656 | Upregulated |
| LIPM | 184.0779817 | 206.7988166 | 0.167911 | 0.047553 | 0.047553 | Upregulated |
| TBCA | 184.0688073 | 206.8106509 | 0.168065 | 0.047348 | 0.047348 | Upregulated |
| PLEKHM1 | 184.059633 | 206.8224852 | 0.16822 | 0.047144 | 0.047144 | Upregulated |
| CPOX | 184.0504587 | 206.8343195 | 0.168374 | 0.04694 | 0.04694 | Upregulated |
| EPS8L3 | 184.0504587 | 206.8343195 | 0.168374 | 0.04694 | 0.04694 | Upregulated |
| TOM1 | 184.0458716 | 206.8402367 | 0.168451 | 0.046839 | 0.046839 | Upregulated |
| FUT3 | 184.0229358 | 206.8698225 | 0.168838 | 0.046334 | 0.046334 | Upregulated |
| ARL6IP1 | 184.0137615 | 206.8816568 | 0.168992 | 0.046134 | 0.046134 | Upregulated |
| ETV2 | 184.0091743 | 206.887574 | 0.169069 | 0.046034 | 0.046034 | Upregulated |
| RNF223 | 184.0045872 | 206.8934911 | 0.169147 | 0.045934 | 0.045934 | Upregulated |
| C7orf43 | 184.0045872 | 206.8934911 | 0.169147 | 0.045934 | 0.045934 | Upregulated |
| MATR3 | 184 | 206.8994083 | 0.169224 | 0.045834 | 0.045834 | Upregulated |
| RFX1 | 184 | 206.8994083 | 0.169224 | 0.045834 | 0.045834 | Upregulated |
| NRN1 | 184 | 206.8994083 | 0.169224 | 0.045834 | 0.045834 | Upregulated |
| ATP5B | 183.9954128 | 206.9053254 | 0.169301 | 0.045735 | 0.045735 | Upregulated |
| BSG | 183.9954128 | 206.9053254 | 0.169301 | 0.045735 | 0.045735 | Upregulated |
| FASTK | 183.9908257 | 206.9112426 | 0.169378 | 0.045635 | 0.045635 | Upregulated |
| ITGA9 | 183.9862385 | 206.9171598 | 0.169455 | 0.045536 | 0.045536 | Upregulated |
| C11orf84 | 183.9862385 | 206.9171598 | 0.169455 | 0.045536 | 0.045536 | Upregulated |
| TRRAP | 183.9862385 | 206.9171598 | 0.169455 | 0.045536 | 0.045536 | Upregulated |
| NT5DC3 | 183.9678899 | 206.9408284 | 0.169764 | 0.045142 | 0.045142 | Upregulated |
| PATJ | 183.9678899 | 206.9408284 | 0.169764 | 0.045142 | 0.045142 | Upregulated |
| IL1R2 | 183.9678899 | 206.9408284 | 0.169764 | 0.045142 | 0.045142 | Upregulated |
| PIH1D1 | 183.4587156 | 206.3786982 | 0.169839 | 0.04475 | 0.04475 | Upregulated |
| PIM3 | 183.9449541 | 206.9704142 | 0.17015 | 0.044653 | 0.044653 | Upregulated |
| LINC01207 | 183.9449541 | 206.9704142 | 0.17015 | 0.044653 | 0.044653 | Upregulated |
| SFXN1 | 183.9357798 | 206.9822485 | 0.170305 | 0.044458 | 0.044458 | Upregulated |
| THUMPD1 | 183.9311927 | 206.9881657 | 0.170382 | 0.044361 | 0.044361 | Upregulated |
| TTYH3 | 183.9311927 | 206.9881657 | 0.170382 | 0.044361 | 0.044361 | Upregulated |
| HSD17B8 | 183.9266055 | 206.9940828 | 0.170459 | 0.044265 | 0.044265 | Upregulated |
| THAP2 | 183.9174312 | 207.0059172 | 0.170614 | 0.044072 | 0.044072 | Upregulated |
| GINS2 | 183.912844 | 207.0118343 | 0.170691 | 0.043975 | 0.043975 | Upregulated |
| TLE4 | 183.9082569 | 207.0177515 | 0.170768 | 0.043879 | 0.043879 | Upregulated |
| ZMIZ1 | 183.9036697 | 207.0236686 | 0.170845 | 0.043783 | 0.043783 | Upregulated |
| TMEM86B | 183.9036697 | 207.0236686 | 0.170845 | 0.043783 | 0.043783 | Upregulated |
| CD151 | 183.8853211 | 207.0473373 | 0.171154 | 0.043402 | 0.043402 | Upregulated |
| MCRS1 | 183.8807339 | 207.0532544 | 0.171232 | 0.043307 | 0.043307 | Upregulated |
| EARS2 | 183.8761468 | 207.0591716 | 0.171309 | 0.043212 | 0.043212 | Upregulated |
| GTF2F1 | 183.8761468 | 207.0591716 | 0.171309 | 0.043212 | 0.043212 | Upregulated |
| EHMT1 | 183.8761468 | 207.0591716 | 0.171309 | 0.043212 | 0.043212 | Upregulated |
| C6orf120 | 183.8715596 | 207.0650888 | 0.171386 | 0.043117 | 0.043117 | Upregulated |
| PRSS8 | 183.8669725 | 207.0710059 | 0.171463 | 0.043023 | 0.043023 | Upregulated |
| CYBA | 183.8623853 | 207.0769231 | 0.17154 | 0.042929 | 0.042929 | Upregulated |
| BTBD6 | 183.8577982 | 207.0828402 | 0.171618 | 0.042835 | 0.042835 | Upregulated |
| KRT7 | 183.8440367 | 207.1005917 | 0.171849 | 0.042553 | 0.042553 | Upregulated |
| FBXO10 | 183.8348624 | 207.112426 | 0.172004 | 0.042367 | 0.042367 | Upregulated |
| INO80C | 183.8302752 | 207.1183432 | 0.172081 | 0.042274 | 0.042274 | Upregulated |
| SMAD9 | 183.8302752 | 207.1183432 | 0.172081 | 0.042274 | 0.042274 | Upregulated |
| GCNT3 | 183.8211009 | 207.1301775 | 0.172235 | 0.042088 | 0.042088 | Upregulated |
| HEATR3 | 183.8119266 | 207.1420118 | 0.17239 | 0.041903 | 0.041903 | Upregulated |
| CISH | 183.8073394 | 207.147929 | 0.172467 | 0.041811 | 0.041811 | Upregulated |
| KCTD1 | 183.8073394 | 207.147929 | 0.172467 | 0.041811 | 0.041811 | Upregulated |
| CHCHD6 | 183.7981651 | 207.1597633 | 0.172621 | 0.041627 | 0.041627 | Upregulated |
| C19orf54 | 183.793578 | 207.1656805 | 0.172699 | 0.041535 | 0.041535 | Upregulated |
| IL20RB | 182.8440367 | 206.1065089 | 0.172776 | 0.042506 | 0.042506 | Upregulated |
| RBPMS | 183.7798165 | 207.183432 | 0.17293 | 0.041261 | 0.041261 | Upregulated |
| CFAP36 | 183.7752294 | 207.1893491 | 0.173008 | 0.04117 | 0.04117 | Upregulated |
| SNRPE | 183.7752294 | 207.1893491 | 0.173008 | 0.04117 | 0.04117 | Upregulated |
| KDM4B | 183.7706422 | 207.1952663 | 0.173085 | 0.041079 | 0.041079 | Upregulated |
| CAPN5 | 183.766055 | 207.2011834 | 0.173162 | 0.040989 | 0.040989 | Upregulated |
| TNIK | 183.766055 | 207.2011834 | 0.173162 | 0.040989 | 0.040989 | Upregulated |
| ZYX | 183.7614679 | 207.2071006 | 0.173239 | 0.040898 | 0.040898 | Upregulated |
| NR4A2 | 183.7568807 | 207.2130178 | 0.173316 | 0.040808 | 0.040808 | Upregulated |
| NCOA6 | 183.7431193 | 207.2307692 | 0.173548 | 0.040538 | 0.040538 | Upregulated |
| CDC73 | 183.7385321 | 207.2366864 | 0.173625 | 0.040448 | 0.040448 | Upregulated |
| BLOC1S1 | 183.7385321 | 207.2366864 | 0.173625 | 0.040448 | 0.040448 | Upregulated |
| ANTXR2 | 183.733945 | 207.2426036 | 0.173702 | 0.040359 | 0.040359 | Upregulated |
| ZNF324 | 183.7293578 | 207.2485207 | 0.17378 | 0.040269 | 0.040269 | Upregulated |
| FOXO4 | 183.7247706 | 207.2544379 | 0.173857 | 0.04018 | 0.04018 | Upregulated |
| ARHGEF5 | 183.7110092 | 207.2721893 | 0.174088 | 0.039914 | 0.039914 | Upregulated |
| C12orf76 | 183.706422 | 207.2781065 | 0.174166 | 0.039825 | 0.039825 | Upregulated |
| FBXL8 | 183.706422 | 207.2781065 | 0.174166 | 0.039825 | 0.039825 | Upregulated |
| UGGT1 | 183.706422 | 207.2781065 | 0.174166 | 0.039825 | 0.039825 | Upregulated |
| SLC35E1 | 183.706422 | 207.2781065 | 0.174166 | 0.039825 | 0.039825 | Upregulated |
| TNNT1 | 180.8761468 | 204.0946746 | 0.174236 | 0.042787 | 0.042787 | Upregulated |
| ANKMY1 | 183.5137615 | 207.0710059 | 0.174237 | 0.039254 | 0.039254 | Upregulated |
| ABCA13 | 181.8119266 | 205.1597633 | 0.174301 | 0.041811 | 0.041811 | Upregulated |
| IQSEC1 | 183.6972477 | 207.2899408 | 0.17432 | 0.039649 | 0.039649 | Upregulated |
| MUC1 | 183.6926606 | 207.295858 | 0.174397 | 0.039561 | 0.039561 | Upregulated |
| IL1RL2 | 180.8623853 | 204.112426 | 0.174472 | 0.042366 | 0.042366 | Upregulated |
| TWISTNB | 183.6834862 | 207.3076923 | 0.174552 | 0.039385 | 0.039385 | Upregulated |
| LPAR6 | 183.6834862 | 207.3076923 | 0.174552 | 0.039385 | 0.039385 | Upregulated |
| DRAM1 | 183.6788991 | 207.3136095 | 0.174629 | 0.039298 | 0.039298 | Upregulated |
| C19orf48 | 183.6788991 | 207.3136095 | 0.174629 | 0.039298 | 0.039298 | Upregulated |
| FLRT3 | 183.6743119 | 207.3195266 | 0.174706 | 0.039211 | 0.039211 | Upregulated |
| NUDT18 | 183.6697248 | 207.3254438 | 0.174783 | 0.039124 | 0.039124 | Upregulated |
| RPL29 | 183.6697248 | 207.3254438 | 0.174783 | 0.039124 | 0.039124 | Upregulated |
| FAM169A | 183.6605505 | 207.3372781 | 0.174938 | 0.03895 | 0.03895 | Upregulated |
| WDR72 | 181.7706422 | 205.2130178 | 0.175003 | 0.040898 | 0.040898 | Upregulated |
| MSTO1 | 183.6513761 | 207.3491124 | 0.175092 | 0.038777 | 0.038777 | Upregulated |
| C2CD4B | 183.646789 | 207.3550296 | 0.175169 | 0.03869 | 0.03869 | Upregulated |
| PHPT1 | 183.646789 | 207.3550296 | 0.175169 | 0.03869 | 0.03869 | Upregulated |
| SIGLEC15 | 183.6330275 | 207.3727811 | 0.175401 | 0.038432 | 0.038432 | Upregulated |
| MRPS23 | 183.6330275 | 207.3727811 | 0.175401 | 0.038432 | 0.038432 | Upregulated |
| C18orf8 | 183.6146789 | 207.3964497 | 0.17571 | 0.03809 | 0.03809 | Upregulated |
| MIF4GD | 183.6100917 | 207.4023669 | 0.175787 | 0.038005 | 0.038005 | Upregulated |
| PITPNM3 | 183.6100917 | 207.4023669 | 0.175787 | 0.038005 | 0.038005 | Upregulated |
| CDK6 | 183.6055046 | 207.408284 | 0.175864 | 0.037921 | 0.037921 | Upregulated |
| CD5 | 183.6009174 | 207.4142012 | 0.175941 | 0.037836 | 0.037836 | Upregulated |
| SPATA12 | 183.5779817 | 207.443787 | 0.176327 | 0.037415 | 0.037415 | Upregulated |
| UPK3B | 179.8073394 | 203.2071006 | 0.176499 | 0.041261 | 0.041261 | Upregulated |
| PLA2G4B | 174.2293578 | 196.9289941 | 0.176688 | 0.046836 | 0.046836 | Upregulated |
| FAM83G | 183.5550459 | 207.4733728 | 0.176713 | 0.036998 | 0.036998 | Upregulated |
| COX7A2 | 183.5504587 | 207.4792899 | 0.176791 | 0.036915 | 0.036915 | Upregulated |
| RDH13 | 183.5458716 | 207.4852071 | 0.176868 | 0.036832 | 0.036832 | Upregulated |
| KMT2D | 183.5366972 | 207.4970414 | 0.177022 | 0.036667 | 0.036667 | Upregulated |
| FAM174B | 183.5321101 | 207.5029586 | 0.177099 | 0.036584 | 0.036584 | Upregulated |
| DRAXIN | 178.8348624 | 202.2011834 | 0.177163 | 0.041443 | 0.041443 | Upregulated |
| TRIM41 | 183.5275229 | 207.5088757 | 0.177177 | 0.036502 | 0.036502 | Upregulated |
| GAD1 | 181.6330275 | 205.3905325 | 0.177343 | 0.038176 | 0.038176 | Upregulated |
| SPOP | 183.5091743 | 207.5325444 | 0.177485 | 0.036175 | 0.036175 | Upregulated |
| VDR | 183.5045872 | 207.5384615 | 0.177563 | 0.036094 | 0.036094 | Upregulated |
| COX4I1 | 182.6146789 | 206.5384615 | 0.177608 | 0.037081 | 0.037081 | Upregulated |
| CKMT1A | 183.4908257 | 207.556213 | 0.177794 | 0.035851 | 0.035851 | Upregulated |
| MSX1 | 183.4862385 | 207.5621302 | 0.177871 | 0.03577 | 0.03577 | Upregulated |
| N6AMT1 | 183.4862385 | 207.5621302 | 0.177871 | 0.03577 | 0.03577 | Upregulated |
| SPINT1 | 183.4816514 | 207.5680473 | 0.177949 | 0.035689 | 0.035689 | Upregulated |
| RPL36A | 183.4816514 | 207.5680473 | 0.177949 | 0.035689 | 0.035689 | Upregulated |
| RAET1G | 178.7889908 | 202.260355 | 0.177956 | 0.040762 | 0.040762 | Upregulated |
| C14orf79 | 183.4678899 | 207.5857988 | 0.17818 | 0.035448 | 0.035448 | Upregulated |
| SIPA1L1 | 183.4587156 | 207.5976331 | 0.178335 | 0.035289 | 0.035289 | Upregulated |
| NBN | 183.4587156 | 207.5976331 | 0.178335 | 0.035289 | 0.035289 | Upregulated |
| AC007906.2 | 183.4541284 | 207.6035503 | 0.178412 | 0.035209 | 0.035209 | Upregulated |
| PLOD3 | 183.4541284 | 207.6035503 | 0.178412 | 0.035209 | 0.035209 | Upregulated |
| APBB3 | 183.4449541 | 207.6153846 | 0.178566 | 0.03505 | 0.03505 | Upregulated |
| SLC20A2 | 183.440367 | 207.6213018 | 0.178643 | 0.034971 | 0.034971 | Upregulated |
| ZCCHC10 | 183.440367 | 207.6213018 | 0.178643 | 0.034971 | 0.034971 | Upregulated |
| ZNF839 | 183.4266055 | 207.6390533 | 0.178875 | 0.034734 | 0.034734 | Upregulated |
| EDC4 | 183.4220183 | 207.6449704 | 0.178952 | 0.034655 | 0.034655 | Upregulated |
| DNAJC12 | 183.4220183 | 207.6449704 | 0.178952 | 0.034655 | 0.034655 | Upregulated |
| BAG1 | 183.4082569 | 207.6627219 | 0.179184 | 0.034421 | 0.034421 | Upregulated |
| WDFY2 | 183.4082569 | 207.6627219 | 0.179184 | 0.034421 | 0.034421 | Upregulated |
| OR5AN1 | 1.013761468 | 1.147928994 | 0.179315 | 0.023675 | 0.023675 | Upregulated |
| C19orf68 | 183.3899083 | 207.6863905 | 0.179492 | 0.034109 | 0.034109 | Upregulated |
| KMT5A | 182.733945 | 206.9526627 | 0.179556 | 0.033955 | 0.033955 | Upregulated |
| TRIM11 | 183.3853211 | 207.6923077 | 0.17957 | 0.034032 | 0.034032 | Upregulated |
| DISP3 | 174.0642202 | 197.1420118 | 0.179616 | 0.043304 | 0.043304 | Upregulated |
| NUDT16 | 183.3715596 | 207.7100592 | 0.179801 | 0.033801 | 0.033801 | Upregulated |
| CIT | 183.3669725 | 207.7159763 | 0.179878 | 0.033724 | 0.033724 | Upregulated |
| STRN4 | 183.3394495 | 207.7514793 | 0.180342 | 0.033266 | 0.033266 | Upregulated |
| AKNA | 183.3394495 | 207.7514793 | 0.180342 | 0.033266 | 0.033266 | Upregulated |
| ADIPOR2 | 183.3348624 | 207.7573964 | 0.180419 | 0.033191 | 0.033191 | Upregulated |
| MEF2D | 183.3302752 | 207.7633136 | 0.180496 | 0.033115 | 0.033115 | Upregulated |
| VCP | 183.3256881 | 207.7692308 | 0.180573 | 0.03304 | 0.03304 | Upregulated |
| BCL11A | 183.3256881 | 207.7692308 | 0.180573 | 0.03304 | 0.03304 | Upregulated |
| GRAP | 178.6330275 | 202.4615385 | 0.180649 | 0.037372 | 0.037372 | Upregulated |
| UQCR10 | 183.3165138 | 207.7810651 | 0.180727 | 0.032889 | 0.032889 | Upregulated |
| TPT1 | 183.3119266 | 207.7869822 | 0.180805 | 0.032814 | 0.032814 | Upregulated |
| MIER2 | 183.3073394 | 207.7928994 | 0.180882 | 0.032739 | 0.032739 | Upregulated |
| LAMB1 | 183.3073394 | 207.7928994 | 0.180882 | 0.032739 | 0.032739 | Upregulated |
| SOCS3 | 183.3027523 | 207.7988166 | 0.180959 | 0.032664 | 0.032664 | Upregulated |
| EDF1 | 183.3027523 | 207.7988166 | 0.180959 | 0.032664 | 0.032664 | Upregulated |
| AEBP1 | 183.2981651 | 207.8047337 | 0.181036 | 0.03259 | 0.03259 | Upregulated |
| ZNF384 | 183.2981651 | 207.8047337 | 0.181036 | 0.03259 | 0.03259 | Upregulated |
| SNRPA | 183.293578 | 207.8106509 | 0.181113 | 0.032515 | 0.032515 | Upregulated |
| SLC16A13 | 183.293578 | 207.8106509 | 0.181113 | 0.032515 | 0.032515 | Upregulated |
| ALDH1B1 | 183.2706422 | 207.8402367 | 0.181499 | 0.032145 | 0.032145 | Upregulated |
| NCALD | 183.266055 | 207.8461538 | 0.181576 | 0.032072 | 0.032072 | Upregulated |
| DLL1 | 183.2522936 | 207.8639053 | 0.181808 | 0.031852 | 0.031852 | Upregulated |
| GPN3 | 183.2522936 | 207.8639053 | 0.181808 | 0.031852 | 0.031852 | Upregulated |
| CORO2B | 182.3073394 | 206.7988166 | 0.181855 | 0.032664 | 0.032664 | Upregulated |
| POMT2 | 183.2431193 | 207.8757396 | 0.181962 | 0.031706 | 0.031706 | Upregulated |
| SLC52A3 | 183.2385321 | 207.8816568 | 0.18204 | 0.031634 | 0.031634 | Upregulated |
| BAK1 | 183.2110092 | 207.9171598 | 0.182503 | 0.0312 | 0.0312 | Upregulated |
| RASD1 | 183.206422 | 207.9230769 | 0.18258 | 0.031129 | 0.031129 | Upregulated |
| SELENOW | 183.1834862 | 207.9526627 | 0.182966 | 0.030772 | 0.030772 | Upregulated |
| RPL24 | 183.1834862 | 207.9526627 | 0.182966 | 0.030772 | 0.030772 | Upregulated |
| B3GNT2 | 183.1743119 | 207.964497 | 0.18312 | 0.030631 | 0.030631 | Upregulated |
| MLH1 | 183.1697248 | 207.9704142 | 0.183197 | 0.03056 | 0.03056 | Upregulated |
| KANSL2 | 183.1697248 | 207.9704142 | 0.183197 | 0.03056 | 0.03056 | Upregulated |
| COX20 | 183.1651376 | 207.9763314 | 0.183274 | 0.03049 | 0.03049 | Upregulated |
| POLL | 183.1559633 | 207.9881657 | 0.183429 | 0.030349 | 0.030349 | Upregulated |
| CRYBG2 | 183.1422018 | 208.0059172 | 0.18366 | 0.03014 | 0.03014 | Upregulated |
| SKAP1 | 183.1330275 | 208.0177515 | 0.183815 | 0.03 | 0.03 | Upregulated |
| BMX | 183.1284404 | 208.0236686 | 0.183892 | 0.029931 | 0.029931 | Upregulated |
| OSBPL5 | 183.1238532 | 208.0295858 | 0.183969 | 0.029862 | 0.029862 | Upregulated |
| MFSD9 | 183.1238532 | 208.0295858 | 0.183969 | 0.029862 | 0.029862 | Upregulated |
| VWA3B | 179.3669725 | 203.7751479 | 0.184064 | 0.033418 | 0.033418 | Upregulated |
| TNFRSF10D | 183.1146789 | 208.0414201 | 0.184123 | 0.029724 | 0.029724 | Upregulated |
| RTN2 | 183.1100917 | 208.0473373 | 0.184201 | 0.029655 | 0.029655 | Upregulated |
| ARPC1B | 183.1100917 | 208.0473373 | 0.184201 | 0.029655 | 0.029655 | Upregulated |
| BTF3 | 183.0963303 | 208.0650888 | 0.184432 | 0.02945 | 0.02945 | Upregulated |
| RSPH1 | 183.0963303 | 208.0650888 | 0.184432 | 0.02945 | 0.02945 | Upregulated |
| VAPB | 183.0917431 | 208.0710059 | 0.184509 | 0.029381 | 0.029381 | Upregulated |
| E2F3 | 183.087156 | 208.0769231 | 0.184586 | 0.029313 | 0.029313 | Upregulated |
| HOMEZ | 183.0688073 | 208.1005917 | 0.184895 | 0.029042 | 0.029042 | Upregulated |
| TRAPPC12 | 183.0504587 | 208.1242604 | 0.185204 | 0.028773 | 0.028773 | Upregulated |
| HNRNPUL2-BSCL2 | 180.233945 | 204.9230769 | 0.185212 | 0.031129 | 0.031129 | Upregulated |
| AMIGO2 | 183.0412844 | 208.1360947 | 0.185358 | 0.02864 | 0.02864 | Upregulated |
| AP2S1 | 182.3165138 | 207.3254438 | 0.185452 | 0.028607 | 0.028607 | Upregulated |
| DDO | 183.0275229 | 208.1538462 | 0.18559 | 0.02844 | 0.02844 | Upregulated |
| LRIG3 | 183.0229358 | 208.1597633 | 0.185667 | 0.028374 | 0.028374 | Upregulated |
| PFDN1 | 183.0137615 | 208.1715976 | 0.185821 | 0.028242 | 0.028242 | Upregulated |
| GPC3 | 183.0091743 | 208.1775148 | 0.185898 | 0.028176 | 0.028176 | Upregulated |
| SMG9 | 183.0045872 | 208.183432 | 0.185975 | 0.028111 | 0.028111 | Upregulated |
| UXT | 183 | 208.1893491 | 0.186053 | 0.028045 | 0.028045 | Upregulated |
| PHC2 | 183 | 208.1893491 | 0.186053 | 0.028045 | 0.028045 | Upregulated |
| ARF3 | 182.9862385 | 208.2071006 | 0.186284 | 0.027849 | 0.027849 | Upregulated |
| AVL9 | 182.9862385 | 208.2071006 | 0.186284 | 0.027849 | 0.027849 | Upregulated |
| SYNGR4 | 165.5275229 | 188.3431953 | 0.186293 | 0.04498 | 0.04498 | Upregulated |
| FAM222A | 182.9816514 | 208.2130178 | 0.186361 | 0.027784 | 0.027784 | Upregulated |
| TOMM40L | 182.9816514 | 208.2130178 | 0.186361 | 0.027784 | 0.027784 | Upregulated |
| S100A5 | 174.6009174 | 198.6804734 | 0.186389 | 0.035607 | 0.035607 | Upregulated |
| C14orf166 | 182.9770642 | 208.2189349 | 0.186438 | 0.027719 | 0.027719 | Upregulated |
| PARP6 | 182.9587156 | 208.2426036 | 0.186747 | 0.027461 | 0.027461 | Upregulated |
| NOSTRIN | 182.9541284 | 208.2485207 | 0.186824 | 0.027397 | 0.027397 | Upregulated |
| ATG3 | 182.9541284 | 208.2485207 | 0.186824 | 0.027397 | 0.027397 | Upregulated |
| CS | 182.9495413 | 208.2544379 | 0.186901 | 0.027333 | 0.027333 | Upregulated |
| RXRB | 182.9449541 | 208.260355 | 0.186979 | 0.027269 | 0.027269 | Upregulated |
| VEZT | 182.9449541 | 208.260355 | 0.186979 | 0.027269 | 0.027269 | Upregulated |
| AGAP5 | 169.0917431 | 192.4911243 | 0.186986 | 0.040621 | 0.040621 | Upregulated |
| LIMA1 | 182.940367 | 208.2662722 | 0.187056 | 0.027205 | 0.027205 | Upregulated |
| PRRG2 | 182.9266055 | 208.2840237 | 0.187287 | 0.027014 | 0.027014 | Upregulated |
| ZNF628 | 182.9220183 | 208.2899408 | 0.187364 | 0.026951 | 0.026951 | Upregulated |
| URB1 | 182.9174312 | 208.295858 | 0.187442 | 0.026888 | 0.026888 | Upregulated |
| SPEN | 182.9036697 | 208.3136095 | 0.187673 | 0.026699 | 0.026699 | Upregulated |
| ATF7 | 182.9036697 | 208.3136095 | 0.187673 | 0.026699 | 0.026699 | Upregulated |
| NOMO1 | 182.9036697 | 208.3136095 | 0.187673 | 0.026699 | 0.026699 | Upregulated |
| C19orf24 | 182.8990826 | 208.3195266 | 0.18775 | 0.026636 | 0.026636 | Upregulated |
| RPH3AL | 182.8944954 | 208.3254438 | 0.187827 | 0.026573 | 0.026573 | Upregulated |
| MMAB | 182.8899083 | 208.3313609 | 0.187905 | 0.026511 | 0.026511 | Upregulated |
| MTUS1 | 182.8899083 | 208.3313609 | 0.187905 | 0.026511 | 0.026511 | Upregulated |
| ACSS1 | 182.8899083 | 208.3313609 | 0.187905 | 0.026511 | 0.026511 | Upregulated |
| NUTM2G | 164.5412844 | 187.443787 | 0.188008 | 0.042724 | 0.042724 | Upregulated |
| POLE | 182.8807339 | 208.3431953 | 0.188059 | 0.026386 | 0.026386 | Upregulated |
| ZFYVE19 | 182.8715596 | 208.3550296 | 0.188213 | 0.026262 | 0.026262 | Upregulated |
| VWA5B2 | 175.412844 | 199.8698225 | 0.188306 | 0.032366 | 0.032366 | Upregulated |
| RPL35 | 182.8623853 | 208.3668639 | 0.188368 | 0.026139 | 0.026139 | Upregulated |
| CD46 | 182.8623853 | 208.3668639 | 0.188368 | 0.026139 | 0.026139 | Upregulated |
| PLK2 | 182.8440367 | 208.3905325 | 0.188676 | 0.025893 | 0.025893 | Upregulated |
| GPI | 182.8394495 | 208.3964497 | 0.188753 | 0.025832 | 0.025832 | Upregulated |
| KANSL1L | 182.8394495 | 208.3964497 | 0.188753 | 0.025832 | 0.025832 | Upregulated |
| LRCH4 | 182.8211009 | 208.4201183 | 0.189062 | 0.025589 | 0.025589 | Upregulated |
| TBC1D4 | 182.8027523 | 208.443787 | 0.189371 | 0.025348 | 0.025348 | Upregulated |
| PRR36 | 182.7981651 | 208.4497041 | 0.189448 | 0.025288 | 0.025288 | Upregulated |
| IL1A | 180.9082569 | 206.3254438 | 0.189663 | 0.026636 | 0.026636 | Upregulated |
| SMIM4 | 182.7844037 | 208.4674556 | 0.189679 | 0.025109 | 0.025109 | Upregulated |
| PA2G4 | 182.7844037 | 208.4674556 | 0.189679 | 0.025109 | 0.025109 | Upregulated |
| DPEP1 | 182.7798165 | 208.4733728 | 0.189756 | 0.02505 | 0.02505 | Upregulated |
| PPP1R9A | 182.7706422 | 208.4852071 | 0.189911 | 0.024931 | 0.024931 | Upregulated |
| METTL17 | 182.7706422 | 208.4852071 | 0.189911 | 0.024931 | 0.024931 | Upregulated |
| DAGLA | 182.7614679 | 208.4970414 | 0.190065 | 0.024813 | 0.024813 | Upregulated |
| EEF1G | 170.733945 | 194.7810651 | 0.190104 | 0.036457 | 0.036457 | Upregulated |
| CIC | 182.7568807 | 208.5029586 | 0.190142 | 0.024754 | 0.024754 | Upregulated |
| NACA | 182.7477064 | 208.5147929 | 0.190296 | 0.024637 | 0.024637 | Upregulated |
| TMEM5 | 182.7293578 | 208.5384615 | 0.190605 | 0.024404 | 0.024404 | Upregulated |
| TMSB10 | 182.7293578 | 208.5384615 | 0.190605 | 0.024404 | 0.024404 | Upregulated |
| LRRIQ4 | 181.7889908 | 207.4674556 | 0.19062 | 0.025109 | 0.025109 | Upregulated |
| ACBD6 | 182.7155963 | 208.556213 | 0.190837 | 0.02423 | 0.02423 | Upregulated |
| ULBP1 | 177.1100917 | 202.1715976 | 0.190934 | 0.02844 | 0.02844 | Upregulated |
| KIAA1522 | 182.7018349 | 208.5739645 | 0.191068 | 0.024058 | 0.024058 | Upregulated |
| AC092718.3 | 167.0504587 | 190.739645 | 0.191321 | 0.040438 | 0.040438 | Upregulated |
| RNH1 | 182.6834862 | 208.5976331 | 0.191377 | 0.023829 | 0.023829 | Upregulated |
| LPL | 182.6788991 | 208.6035503 | 0.191454 | 0.023772 | 0.023772 | Upregulated |
| CDC23 | 182.6788991 | 208.6035503 | 0.191454 | 0.023772 | 0.023772 | Upregulated |
| C2CD4D | 182.6788991 | 208.6035503 | 0.191454 | 0.023772 | 0.023772 | Upregulated |
| ILDR1 | 182.6788991 | 208.6035503 | 0.191454 | 0.023772 | 0.023772 | Upregulated |
| NEURL1B | 182.6697248 | 208.6153846 | 0.191608 | 0.023659 | 0.023659 | Upregulated |
| SUSD4 | 167.9311927 | 191.7928994 | 0.191679 | 0.034569 | 0.034569 | Upregulated |
| NUCB1 | 182.6559633 | 208.6331361 | 0.191839 | 0.02349 | 0.02349 | Upregulated |
| GCC1 | 182.6559633 | 208.6331361 | 0.191839 | 0.02349 | 0.02349 | Upregulated |
| SLC16A11 | 182.6422018 | 208.6508876 | 0.192071 | 0.023322 | 0.023322 | Upregulated |
| POC1B | 182.6422018 | 208.6508876 | 0.192071 | 0.023322 | 0.023322 | Upregulated |
| ACKR3 | 182.6422018 | 208.6508876 | 0.192071 | 0.023322 | 0.023322 | Upregulated |
| RNF187 | 182.6422018 | 208.6508876 | 0.192071 | 0.023322 | 0.023322 | Upregulated |
| ZNF524 | 182.6376147 | 208.6568047 | 0.192148 | 0.023266 | 0.023266 | Upregulated |
| BRD7 | 182.6376147 | 208.6568047 | 0.192148 | 0.023266 | 0.023266 | Upregulated |
| SPRYD3 | 182.6330275 | 208.6627219 | 0.192225 | 0.02321 | 0.02321 | Upregulated |
| LPCAT1 | 182.6238532 | 208.6745562 | 0.192379 | 0.023099 | 0.023099 | Upregulated |
| PRPF19 | 182.6238532 | 208.6745562 | 0.192379 | 0.023099 | 0.023099 | Upregulated |
| PIWIL4 | 182.6146789 | 208.6863905 | 0.192534 | 0.022989 | 0.022989 | Upregulated |
| ASPDH | 160.7431193 | 183.7159763 | 0.19272 | 0.039622 | 0.039622 | Upregulated |
| CHMP2A | 182.6009174 | 208.704142 | 0.192765 | 0.022824 | 0.022824 | Upregulated |
| SLC37A4 | 182.6009174 | 208.704142 | 0.192765 | 0.022824 | 0.022824 | Upregulated |
| WDR45 | 182.5963303 | 208.7100592 | 0.192842 | 0.022769 | 0.022769 | Upregulated |
| PSPH | 182.5963303 | 208.7100592 | 0.192842 | 0.022769 | 0.022769 | Upregulated |
| RAD23A | 182.5963303 | 208.7100592 | 0.192842 | 0.022769 | 0.022769 | Upregulated |
| ABHD14B | 182.5917431 | 208.7159763 | 0.19292 | 0.022715 | 0.022715 | Upregulated |
| ULBP2 | 182.5825688 | 208.7278107 | 0.193074 | 0.022606 | 0.022606 | Upregulated |
| DNAJC22 | 182.5733945 | 208.739645 | 0.193228 | 0.022497 | 0.022497 | Upregulated |
| TMA16 | 182.5688073 | 208.7455621 | 0.193305 | 0.022443 | 0.022443 | Upregulated |
| IFT57 | 182.559633 | 208.7573964 | 0.19346 | 0.022336 | 0.022336 | Upregulated |
| LRRC6 | 182.5504587 | 208.7692308 | 0.193614 | 0.022228 | 0.022228 | Upregulated |
| CALML4 | 182.5412844 | 208.7810651 | 0.193768 | 0.022121 | 0.022121 | Upregulated |
| UTP6 | 182.5366972 | 208.7869822 | 0.193845 | 0.022068 | 0.022068 | Upregulated |
| ARHGAP39 | 182.5366972 | 208.7869822 | 0.193845 | 0.022068 | 0.022068 | Upregulated |
| RYR2 | 182.5275229 | 208.7988166 | 0.194 | 0.021962 | 0.021962 | Upregulated |
| TMEM53 | 182.5229358 | 208.8047337 | 0.194077 | 0.021909 | 0.021909 | Upregulated |
| WFIKKN1 | 181.5825688 | 207.7337278 | 0.19411 | 0.022579 | 0.022579 | Upregulated |
| IFI27L1 | 182.5183486 | 208.8106509 | 0.194154 | 0.021856 | 0.021856 | Upregulated |
| RGCC | 182.5137615 | 208.816568 | 0.194231 | 0.021803 | 0.021803 | Upregulated |
| LTBR | 182.5091743 | 208.8224852 | 0.194308 | 0.021751 | 0.021751 | Upregulated |
| TG | 182.5045872 | 208.8284024 | 0.194385 | 0.021698 | 0.021698 | Upregulated |
| STK10 | 182.5 | 208.8343195 | 0.194462 | 0.021646 | 0.021646 | Upregulated |
| PPA1 | 182.5 | 208.8343195 | 0.194462 | 0.021646 | 0.021646 | Upregulated |
| MARS | 182.4954128 | 208.8402367 | 0.194539 | 0.021594 | 0.021594 | Upregulated |
| STAC3 | 182.4908257 | 208.8461538 | 0.194617 | 0.021542 | 0.021542 | Upregulated |
| TSR1 | 182.4862385 | 208.852071 | 0.194694 | 0.021489 | 0.021489 | Upregulated |
| SERINC2 | 182.4862385 | 208.852071 | 0.194694 | 0.021489 | 0.021489 | Upregulated |
| DDX23 | 182.4816514 | 208.8579882 | 0.194771 | 0.021438 | 0.021438 | Upregulated |
| TMPRSS3 | 182.4770642 | 208.8639053 | 0.194848 | 0.021386 | 0.021386 | Upregulated |
| ST6GALNAC2 | 182.4770642 | 208.8639053 | 0.194848 | 0.021386 | 0.021386 | Upregulated |
| CCNJL | 182.4587156 | 208.887574 | 0.195157 | 0.021179 | 0.021179 | Upregulated |
| NME1-NME2 | 180.5733945 | 206.7573964 | 0.195354 | 0.022336 | 0.022336 | Upregulated |
| DSG3 | 182.4449541 | 208.9053254 | 0.195388 | 0.021026 | 0.021026 | Upregulated |
| SLC18B1 | 182.4357798 | 208.9171598 | 0.195542 | 0.020924 | 0.020924 | Upregulated |
| DNAJB1 | 182.4311927 | 208.9230769 | 0.195619 | 0.020873 | 0.020873 | Upregulated |
| SKOR1 | 180.5504587 | 206.7869822 | 0.195743 | 0.022068 | 0.022068 | Upregulated |
| RBBP9 | 182.4082569 | 208.9526627 | 0.196005 | 0.020621 | 0.020621 | Upregulated |
| C22orf23 | 179.5917431 | 205.7514793 | 0.196182 | 0.022633 | 0.022633 | Upregulated |
| 9-Mar | 182.3853211 | 208.9822485 | 0.196391 | 0.020372 | 0.020372 | Upregulated |
| FFAR4 | 182.3761468 | 208.9940828 | 0.196545 | 0.020273 | 0.020273 | Upregulated |
| PABPC4 | 182.3761468 | 208.9940828 | 0.196545 | 0.020273 | 0.020273 | Upregulated |
| ACADVL | 182.3669725 | 209.0059172 | 0.196699 | 0.020174 | 0.020174 | Upregulated |
| AMH | 178.6284404 | 204.7278107 | 0.196745 | 0.022824 | 0.022824 | Upregulated |
| PCDHGA2 | 179.5504587 | 205.8047337 | 0.196887 | 0.021988 | 0.021988 | Upregulated |
| MROH6 | 182.3486239 | 209.0295858 | 0.197008 | 0.019978 | 0.019978 | Upregulated |
| PKP3 | 182.3348624 | 209.0473373 | 0.197239 | 0.019832 | 0.019832 | Upregulated |
| ODF3L2 | 164.9311927 | 189.112426 | 0.19738 | 0.032244 | 0.032244 | Upregulated |
| EFL1 | 182.3256881 | 209.0591716 | 0.197393 | 0.019735 | 0.019735 | Upregulated |
| DRD4 | 181.3761468 | 208 | 0.197599 | 0.020248 | 0.020248 | Upregulated |
| FGFBP1 | 182.3119266 | 209.0769231 | 0.197625 | 0.019591 | 0.019591 | Upregulated |
| TH | 162.2431193 | 186.0828402 | 0.197788 | 0.032349 | 0.032349 | Upregulated |
| ASIC1 | 182.293578 | 209.1005917 | 0.197933 | 0.0194 | 0.0194 | Upregulated |
| SERGEF | 182.2889908 | 209.1065089 | 0.198011 | 0.019353 | 0.019353 | Upregulated |
| PTRH1 | 163.1146789 | 187.1183432 | 0.198064 | 0.034132 | 0.034132 | Upregulated |
| LAMA5 | 182.266055 | 209.1360947 | 0.198396 | 0.019117 | 0.019117 | Upregulated |
| PPP4R1 | 182.2477064 | 209.1597633 | 0.198705 | 0.01893 | 0.01893 | Upregulated |
| FSIP2 | 182.2431193 | 209.1656805 | 0.198782 | 0.018883 | 0.018883 | Upregulated |
| MED18 | 182.2385321 | 209.1715976 | 0.198859 | 0.018837 | 0.018837 | Upregulated |
| JMJD7-PLA2G4B | 182.2247706 | 209.1893491 | 0.19909 | 0.018698 | 0.018698 | Upregulated |
| TRIOBP | 182.2201835 | 209.1952663 | 0.199167 | 0.018652 | 0.018652 | Upregulated |
| TAOK3 | 182.2155963 | 209.2011834 | 0.199245 | 0.018606 | 0.018606 | Upregulated |
| RHBDL2 | 182.1972477 | 209.2248521 | 0.199553 | 0.018424 | 0.018424 | Upregulated |
| MINOS1 | 182.1926606 | 209.2307692 | 0.19963 | 0.018378 | 0.018378 | Upregulated |
| WNT7B | 178.4541284 | 204.9526627 | 0.199737 | 0.020621 | 0.020621 | Upregulated |
| PTCD1 | 182.1743119 | 209.2544379 | 0.199939 | 0.018197 | 0.018197 | Upregulated |
| AUTS2 | 182.1697248 | 209.260355 | 0.200016 | 0.018153 | 0.018153 | Upregulated |
| UNC5CL | 182.1651376 | 209.2662722 | 0.200093 | 0.018108 | 0.018108 | Upregulated |
| SIX2 | 168.3623853 | 193.4319527 | 0.200256 | 0.028303 | 0.028303 | Upregulated |
| GMDS | 182.1513761 | 209.2840237 | 0.200324 | 0.017974 | 0.017974 | Upregulated |
| ALDH1L1 | 181.2110092 | 208.2130178 | 0.20039 | 0.018515 | 0.018515 | Upregulated |
| MYO3B | 168.3486239 | 193.4497041 | 0.200507 | 0.027617 | 0.027617 | Upregulated |
| C21orf2 | 182.1376147 | 209.3017751 | 0.200556 | 0.017841 | 0.017841 | Upregulated |
| CCND3 | 182.1330275 | 209.3076923 | 0.200633 | 0.017797 | 0.017797 | Upregulated |
| NDUFA3 | 182.1146789 | 209.3313609 | 0.200941 | 0.017621 | 0.017621 | Upregulated |
| AC023509.3 | 182.1146789 | 209.3313609 | 0.200941 | 0.017621 | 0.017621 | Upregulated |
| MECR | 182.1146789 | 209.3313609 | 0.200941 | 0.017621 | 0.017621 | Upregulated |
| TMCC1 | 182.1100917 | 209.3372781 | 0.201018 | 0.017577 | 0.017577 | Upregulated |
| TMEM94 | 181.4954128 | 208.6804734 | 0.201363 | 0.017512 | 0.017512 | Upregulated |
| COL5A2 | 182.087156 | 209.3668639 | 0.201404 | 0.01736 | 0.01736 | Upregulated |
| PTCD3 | 182.0825688 | 209.3727811 | 0.201481 | 0.017317 | 0.017317 | Upregulated |
| U2AF2 | 182.0779817 | 209.3786982 | 0.201558 | 0.017274 | 0.017274 | Upregulated |
| ADCK1 | 182.0504587 | 209.4142012 | 0.202021 | 0.017018 | 0.017018 | Upregulated |
| ATXN2 | 182.0412844 | 209.4260355 | 0.202175 | 0.016934 | 0.016934 | Upregulated |
| RAP1GAP | 182.0412844 | 209.4260355 | 0.202175 | 0.016934 | 0.016934 | Upregulated |
| KLHDC7A | 174.6100917 | 200.9053254 | 0.202379 | 0.021437 | 0.021437 | Upregulated |
| UBE3B | 182.0183486 | 209.4556213 | 0.202561 | 0.016724 | 0.016724 | Upregulated |
| RANBP17 | 182.0183486 | 209.4556213 | 0.202561 | 0.016724 | 0.016724 | Upregulated |
| LSG1 | 182.0045872 | 209.4733728 | 0.202792 | 0.016599 | 0.016599 | Upregulated |
| RNLS | 182.0045872 | 209.4733728 | 0.202792 | 0.016599 | 0.016599 | Upregulated |
| NECTIN4 | 182 | 209.4792899 | 0.202869 | 0.016557 | 0.016557 | Upregulated |
| ZFYVE21 | 182 | 209.4792899 | 0.202869 | 0.016557 | 0.016557 | Upregulated |
| TMEM184A | 181.9954128 | 209.4852071 | 0.202946 | 0.016516 | 0.016516 | Upregulated |
| GTF3C1 | 181.9908257 | 209.4911243 | 0.203023 | 0.016475 | 0.016475 | Upregulated |
| BRD9 | 181.9862385 | 209.4970414 | 0.203101 | 0.016434 | 0.016434 | Upregulated |
| KCNQ4 | 181.9862385 | 209.4970414 | 0.203101 | 0.016434 | 0.016434 | Upregulated |
| SCAF1 | 181.9816514 | 209.5029586 | 0.203178 | 0.016393 | 0.016393 | Upregulated |
| GLIPR1L2 | 154.9587156 | 178.4142012 | 0.203347 | 0.033832 | 0.033832 | Upregulated |
| RPS24 | 181.9587156 | 209.5325444 | 0.203563 | 0.016188 | 0.016188 | Upregulated |
| EIF4B | 181.9449541 | 209.5502959 | 0.203795 | 0.016067 | 0.016067 | Upregulated |
| C2orf82 | 181.940367 | 209.556213 | 0.203872 | 0.016027 | 0.016027 | Upregulated |
| UNC13D | 181.940367 | 209.556213 | 0.203872 | 0.016027 | 0.016027 | Upregulated |
| DNAH2 | 181.9311927 | 209.5680473 | 0.204026 | 0.015947 | 0.015947 | Upregulated |
| RAB26 | 181.9266055 | 209.5739645 | 0.204103 | 0.015907 | 0.015907 | Upregulated |
| PDE9A | 181.9266055 | 209.5739645 | 0.204103 | 0.015907 | 0.015907 | Upregulated |
| BSCL2 | 181.9174312 | 209.5857988 | 0.204257 | 0.015827 | 0.015827 | Upregulated |
| PTPRB | 181.912844 | 209.591716 | 0.204334 | 0.015787 | 0.015787 | Upregulated |
| ETV4 | 181.8807339 | 209.6331361 | 0.204874 | 0.015511 | 0.015511 | Upregulated |
| C9orf66 | 161.8577982 | 186.5798817 | 0.205067 | 0.030262 | 0.030262 | Upregulated |
| AKNAD1 | 165.3944954 | 190.6923077 | 0.205335 | 0.024982 | 0.024982 | Upregulated |
| TNFAIP3 | 181.8486239 | 209.6745562 | 0.205414 | 0.01524 | 0.01524 | Upregulated |
| C14orf80 | 181.8165138 | 209.7159763 | 0.205954 | 0.014973 | 0.014973 | Upregulated |
| FAM53B | 181.8073394 | 209.7278107 | 0.206108 | 0.014897 | 0.014897 | Upregulated |
| CCER2 | 155.6743119 | 179.6035503 | 0.206285 | 0.032216 | 0.032216 | Upregulated |
| TRIM8 | 181.7889908 | 209.7514793 | 0.206416 | 0.014747 | 0.014747 | Upregulated |
| TNFRSF21 | 181.7844037 | 209.7573964 | 0.206493 | 0.014709 | 0.014709 | Upregulated |
| CDC42BPG | 181.7844037 | 209.7573964 | 0.206493 | 0.014709 | 0.014709 | Upregulated |
| ABCG8 | 141.2981651 | 163.0414201 | 0.206496 | 0.048597 | 0.048597 | Upregulated |
| HSPA2 | 181.7752294 | 209.7692308 | 0.206647 | 0.014635 | 0.014635 | Upregulated |
| COPS7A | 181.7522936 | 209.7988166 | 0.207033 | 0.01445 | 0.01445 | Upregulated |
| HMGA2 | 181.7201835 | 209.8402367 | 0.207573 | 0.014195 | 0.014195 | Upregulated |
| NDUFB2 | 181.7155963 | 209.8461538 | 0.20765 | 0.014159 | 0.014159 | Upregulated |
| ABCB8 | 181.7155963 | 209.8461538 | 0.20765 | 0.014159 | 0.014159 | Upregulated |
| BCL2L14 | 181.706422 | 209.8579882 | 0.207804 | 0.014087 | 0.014087 | Upregulated |
| GTPBP4 | 181.6926606 | 209.8757396 | 0.208035 | 0.01398 | 0.01398 | Upregulated |
| CORO1B | 181.6834862 | 209.887574 | 0.208189 | 0.013909 | 0.013909 | Upregulated |
| IMPA1 | 181.6605505 | 209.9171598 | 0.208575 | 0.013732 | 0.013732 | Upregulated |
| NMNAT3 | 181.6559633 | 209.9230769 | 0.208652 | 0.013697 | 0.013697 | Upregulated |
| PGAM1 | 181.6238532 | 209.964497 | 0.209192 | 0.013454 | 0.013454 | Upregulated |
| REG3A | 171.4816514 | 198.2485207 | 0.209256 | 0.019517 | 0.019517 | Upregulated |
| CELSR3 | 181.6146789 | 209.9763314 | 0.209346 | 0.013385 | 0.013385 | Upregulated |
| TRIM16 | 181.6055046 | 209.9881657 | 0.2095 | 0.013317 | 0.013317 | Upregulated |
| DBP | 181.6009174 | 209.9940828 | 0.209577 | 0.013282 | 0.013282 | Upregulated |
| FAM160B2 | 181.5963303 | 210 | 0.209654 | 0.013248 | 0.013248 | Upregulated |
| CAPN3 | 179.7293578 | 207.8461538 | 0.20969 | 0.014195 | 0.014195 | Upregulated |
| GRIN1 | 180.6605505 | 208.9230769 | 0.20969 | 0.013697 | 0.013697 | Upregulated |
| RGS5 | 181.587156 | 210.0118343 | 0.209808 | 0.013181 | 0.013181 | Upregulated |
| FOSB | 181.587156 | 210.0118343 | 0.209808 | 0.013181 | 0.013181 | Upregulated |
| PYM1 | 181.5779817 | 210.0236686 | 0.209963 | 0.013113 | 0.013113 | Upregulated |
| TRIM16L | 181.5733945 | 210.0295858 | 0.21004 | 0.013079 | 0.013079 | Upregulated |
| RAB20 | 181.5458716 | 210.0650888 | 0.210502 | 0.012879 | 0.012879 | Upregulated |
| ZBTB22 | 181.5366972 | 210.0769231 | 0.210656 | 0.012813 | 0.012813 | Upregulated |
| RHOV | 181.5366972 | 210.0769231 | 0.210656 | 0.012813 | 0.012813 | Upregulated |
| TSPAN14 | 181.5183486 | 210.1005917 | 0.210965 | 0.012681 | 0.012681 | Upregulated |
| LRRC8E | 180.5825688 | 209.0236686 | 0.211008 | 0.01313 | 0.01313 | Upregulated |
| CDCA3 | 181.5137615 | 210.1065089 | 0.211042 | 0.012649 | 0.012649 | Upregulated |
| OAS3 | 181.5091743 | 210.112426 | 0.211119 | 0.012616 | 0.012616 | Upregulated |
| IER2 | 181.5045872 | 210.1183432 | 0.211196 | 0.012583 | 0.012583 | Upregulated |
| GAB2 | 181.5045872 | 210.1183432 | 0.211196 | 0.012583 | 0.012583 | Upregulated |
| MZT2B | 181.5 | 210.1242604 | 0.211273 | 0.012551 | 0.012551 | Upregulated |
| TWF1 | 180.8394495 | 209.3905325 | 0.211487 | 0.012502 | 0.012502 | Upregulated |
| ZNF488 | 180.5504587 | 209.0650888 | 0.21155 | 0.012896 | 0.012896 | Upregulated |
| ADGRE5 | 181.4770642 | 210.1538462 | 0.211659 | 0.01239 | 0.01239 | Upregulated |
| PPP6R1 | 181.4633028 | 210.1715976 | 0.21189 | 0.012294 | 0.012294 | Upregulated |
| TUBB3 | 181.4633028 | 210.1715976 | 0.21189 | 0.012294 | 0.012294 | Upregulated |
| CD63 | 181.4587156 | 210.1775148 | 0.211967 | 0.012262 | 0.012262 | Upregulated |
| AP5Z1 | 181.4495413 | 210.1893491 | 0.212121 | 0.012199 | 0.012199 | Upregulated |
| RNF183 | 181.4449541 | 210.1952663 | 0.212198 | 0.012167 | 0.012167 | Upregulated |
| PREP | 181.4174312 | 210.2307692 | 0.212661 | 0.011979 | 0.011979 | Upregulated |
| MTERF3 | 181.4174312 | 210.2307692 | 0.212661 | 0.011979 | 0.011979 | Upregulated |
| CTNNA1 | 181.4082569 | 210.2426036 | 0.212815 | 0.011917 | 0.011917 | Upregulated |
| KCNJ14 | 181.4036697 | 210.2485207 | 0.212892 | 0.011886 | 0.011886 | Upregulated |
| NOB1 | 181.3990826 | 210.2544379 | 0.212969 | 0.011855 | 0.011855 | Upregulated |
| IRF6 | 181.3990826 | 210.2544379 | 0.212969 | 0.011855 | 0.011855 | Upregulated |
| RHBDL3 | 174.0045872 | 201.6863905 | 0.212988 | 0.015472 | 0.015472 | Upregulated |
| MRPS24 | 181.3899083 | 210.2662722 | 0.213123 | 0.011794 | 0.011794 | Upregulated |
| SCEL | 165.8577982 | 192.2781065 | 0.213248 | 0.019181 | 0.019181 | Upregulated |
| CDC34 | 181.3669725 | 210.295858 | 0.213509 | 0.011641 | 0.011641 | Upregulated |
| ITGA2B | 176.7201835 | 204.9289941 | 0.213657 | 0.013662 | 0.013662 | Upregulated |
| PPP1R1C | 173.9633028 | 201.739645 | 0.213712 | 0.015124 | 0.015124 | Upregulated |
| SSNA1 | 181.3440367 | 210.3254438 | 0.213894 | 0.011491 | 0.011491 | Upregulated |
| RPLP2 | 181.3348624 | 210.3372781 | 0.214048 | 0.011431 | 0.011431 | Upregulated |
| C4BPB | 181.3211009 | 210.3550296 | 0.214279 | 0.011342 | 0.011342 | Upregulated |
| FANK1 | 181.2889908 | 210.3964497 | 0.214819 | 0.011136 | 0.011136 | Upregulated |
| ZDHHC12 | 181.2844037 | 210.4023669 | 0.214896 | 0.011107 | 0.011107 | Upregulated |
| MTERF2 | 181.2844037 | 210.4023669 | 0.214896 | 0.011107 | 0.011107 | Upregulated |
| GFM2 | 181.2706422 | 210.4201183 | 0.215127 | 0.01102 | 0.01102 | Upregulated |
| NACC2 | 181.2522936 | 210.443787 | 0.215436 | 0.010905 | 0.010905 | Upregulated |
| MAPT | 179.3715596 | 208.3076923 | 0.215765 | 0.011581 | 0.011581 | Upregulated |
| ALDH3B1 | 181.2201835 | 210.4852071 | 0.215975 | 0.010706 | 0.010706 | Upregulated |
| RAB35 | 181.2110092 | 210.4970414 | 0.216129 | 0.01065 | 0.01065 | Upregulated |
| SLC14A2 | 115.7798165 | 134.5147929 | 0.216381 | 0.047531 | 0.047531 | Upregulated |
| PROM2 | 181.1926606 | 210.5207101 | 0.216438 | 0.010538 | 0.010538 | Upregulated |
| DNASE1L2 | 181.1834862 | 210.5325444 | 0.216592 | 0.010483 | 0.010483 | Upregulated |
| ILF2 | 181.1697248 | 210.5502959 | 0.216823 | 0.0104 | 0.0104 | Upregulated |
| KIAA2012 | 152.5550459 | 177.3076923 | 0.216925 | 0.023335 | 0.023335 | Upregulated |
| PEBP1 | 181.1513761 | 210.5739645 | 0.217131 | 0.010291 | 0.010291 | Upregulated |
| SHH | 181.1422018 | 210.5857988 | 0.217285 | 0.010237 | 0.010237 | Upregulated |
| PQLC2L | 142.4220183 | 165.6331361 | 0.217819 | 0.035786 | 0.035786 | Upregulated |
| PLOD2 | 181.1055046 | 210.6331361 | 0.217902 | 0.010022 | 0.010022 | Upregulated |
| ANKS1A | 181.0963303 | 210.6449704 | 0.218056 | 0.009969 | 0.009969 | Upregulated |
| NUP214 | 181.087156 | 210.6568047 | 0.21821 | 0.009917 | 0.009917 | Upregulated |
| KAT14 | 181.0642202 | 210.6863905 | 0.218596 | 0.009786 | 0.009786 | Upregulated |
| WFS1 | 181.0550459 | 210.6982249 | 0.21875 | 0.009734 | 0.009734 | Upregulated |
| ADGRF1 | 176.412844 | 205.3254438 | 0.218957 | 0.011641 | 0.011641 | Upregulated |
| QRICH2 | 181.0412844 | 210.7159763 | 0.218981 | 0.009657 | 0.009657 | Upregulated |
| TMSB4Y | 126.3302752 | 147.0414201 | 0.219022 | 0.048999 | 0.048999 | Upregulated |
| RASSF3 | 181.0321101 | 210.7278107 | 0.219135 | 0.009606 | 0.009606 | Upregulated |
| NEURL1 | 181.0229358 | 210.739645 | 0.219289 | 0.009555 | 0.009555 | Upregulated |
| 3-Mar | 181.0137615 | 210.7514793 | 0.219443 | 0.009504 | 0.009504 | Upregulated |
| PCBP2 | 181.0091743 | 210.7573964 | 0.21952 | 0.009478 | 0.009478 | Upregulated |
| TFCP2L1 | 180.9954128 | 210.7751479 | 0.219752 | 0.009403 | 0.009403 | Upregulated |
| ARHGEF39 | 180.9770642 | 210.7988166 | 0.22006 | 0.009303 | 0.009303 | Upregulated |
| EXTL3 | 180.9633028 | 210.816568 | 0.220291 | 0.009229 | 0.009229 | Upregulated |
| CAMK1D | 180.9495413 | 210.8343195 | 0.220522 | 0.009155 | 0.009155 | Upregulated |
| MEGF8 | 180.9311927 | 210.8579882 | 0.220831 | 0.009058 | 0.009058 | Upregulated |
| IGFBP2 | 180.9266055 | 210.8639053 | 0.220908 | 0.009033 | 0.009033 | Upregulated |
| PSKH1 | 180.9220183 | 210.8698225 | 0.220985 | 0.009009 | 0.009009 | Upregulated |
| UQCC1 | 180.912844 | 210.8816568 | 0.221139 | 0.008961 | 0.008961 | Upregulated |
| TSPAN8 | 180.9082569 | 210.887574 | 0.221216 | 0.008937 | 0.008937 | Upregulated |
| GSTK1 | 180.8944954 | 210.9053254 | 0.221447 | 0.008865 | 0.008865 | Upregulated |
| HPX | 180.8807339 | 210.9230769 | 0.221678 | 0.008794 | 0.008794 | Upregulated |
| ALG1L2 | 178.0917431 | 207.6863905 | 0.221786 | 0.009786 | 0.009786 | Upregulated |
| ZNF579 | 180.8577982 | 210.9526627 | 0.222063 | 0.008677 | 0.008677 | Upregulated |
| TKT | 180.8486239 | 210.964497 | 0.222218 | 0.00863 | 0.00863 | Upregulated |
| SLC12A2 | 180.8348624 | 210.9822485 | 0.222449 | 0.008561 | 0.008561 | Upregulated |
| RGP1 | 180.8348624 | 210.9822485 | 0.222449 | 0.008561 | 0.008561 | Upregulated |
| ZNF710 | 180.8073394 | 211.0177515 | 0.222911 | 0.008423 | 0.008423 | Upregulated |
| TFAP2A | 180.8027523 | 211.0236686 | 0.222988 | 0.008401 | 0.008401 | Upregulated |
| LGALS4 | 180.7981651 | 211.0295858 | 0.223065 | 0.008378 | 0.008378 | Upregulated |
| PAX6 | 180.793578 | 211.035503 | 0.223142 | 0.008355 | 0.008355 | Upregulated |
| PTGES2 | 180.7889908 | 211.0414201 | 0.223219 | 0.008333 | 0.008333 | Upregulated |
| SRSF9 | 180.7889908 | 211.0414201 | 0.223219 | 0.008333 | 0.008333 | Upregulated |
| LARP1 | 180.7844037 | 211.0473373 | 0.223296 | 0.00831 | 0.00831 | Upregulated |
| HYAL1 | 180.7798165 | 211.0532544 | 0.223373 | 0.008288 | 0.008288 | Upregulated |
| NELFB | 180.7798165 | 211.0532544 | 0.223373 | 0.008288 | 0.008288 | Upregulated |
| IQCE | 180.7752294 | 211.0591716 | 0.223451 | 0.008266 | 0.008266 | Upregulated |
| EIF3F | 180.7706422 | 211.0650888 | 0.223528 | 0.008243 | 0.008243 | Upregulated |
| KCTD13 | 180.7522936 | 211.0887574 | 0.223836 | 0.008154 | 0.008154 | Upregulated |
| RPL3 | 180.7477064 | 211.0946746 | 0.223913 | 0.008132 | 0.008132 | Upregulated |
| BRPF3 | 180.7431193 | 211.1005917 | 0.22399 | 0.00811 | 0.00811 | Upregulated |
| RHOC | 180.7385321 | 211.1065089 | 0.224067 | 0.008088 | 0.008088 | Upregulated |
| ZNF575 | 180.733945 | 211.112426 | 0.224144 | 0.008067 | 0.008067 | Upregulated |
| TOM1L2 | 180.7247706 | 211.1242604 | 0.224298 | 0.008023 | 0.008023 | Upregulated |
| STYK1 | 180.7155963 | 211.1360947 | 0.224452 | 0.00798 | 0.00798 | Upregulated |
| RFXANK | 180.7018349 | 211.1538462 | 0.224683 | 0.007915 | 0.007915 | Upregulated |
| DCDC2 | 175.1605505 | 204.6863905 | 0.224737 | 0.009786 | 0.009786 | Upregulated |
| AC138811.2 | 131.5825688 | 153.7692308 | 0.224798 | 0.037767 | 0.037767 | Upregulated |
| PCLO | 178.8256881 | 209.0118343 | 0.225031 | 0.008469 | 0.008469 | Upregulated |
| MRPL17 | 180.6743119 | 211.1893491 | 0.225146 | 0.007787 | 0.007787 | Upregulated |
| PTGES3L-AARSD1 | 152.1422018 | 177.8402367 | 0.225161 | 0.020283 | 0.020283 | Upregulated |
| WSB2 | 180.6559633 | 211.2130178 | 0.225454 | 0.007702 | 0.007702 | Upregulated |
| IGFN1 | 166.0825688 | 194.1775148 | 0.225475 | 0.013042 | 0.013042 | Upregulated |
| HOXA7 | 180.646789 | 211.2248521 | 0.225608 | 0.00766 | 0.00766 | Upregulated |
| SLC28A3 | 180.6422018 | 211.2307692 | 0.225685 | 0.00764 | 0.00764 | Upregulated |
| RPS9 | 180.6422018 | 211.2307692 | 0.225685 | 0.00764 | 0.00764 | Upregulated |
| ASTN2 | 180.6284404 | 211.2485207 | 0.225916 | 0.007577 | 0.007577 | Upregulated |
| DOCK1 | 180.6284404 | 211.2485207 | 0.225916 | 0.007577 | 0.007577 | Upregulated |
| VSNL1 | 180.6238532 | 211.2544379 | 0.225993 | 0.007557 | 0.007557 | Upregulated |
| PGBD2 | 180.6146789 | 211.2662722 | 0.226147 | 0.007516 | 0.007516 | Upregulated |
| ITFG1 | 180.6146789 | 211.2662722 | 0.226147 | 0.007516 | 0.007516 | Upregulated |
| MBLAC1 | 180.5917431 | 211.295858 | 0.226533 | 0.007414 | 0.007414 | Upregulated |
| PEX2 | 180.5825688 | 211.3076923 | 0.226687 | 0.007373 | 0.007373 | Upregulated |
| DGKA | 180.5825688 | 211.3076923 | 0.226687 | 0.007373 | 0.007373 | Upregulated |
| ASB12 | 156.3577982 | 182.964497 | 0.226713 | 0.018861 | 0.018861 | Upregulated |
| CLDN14 | 178.7201835 | 209.147929 | 0.226821 | 0.007979 | 0.007979 | Upregulated |
| SERPINB6 | 180.5688073 | 211.3254438 | 0.226918 | 0.007313 | 0.007313 | Upregulated |
| CELSR1 | 180.5642202 | 211.3313609 | 0.226995 | 0.007293 | 0.007293 | Upregulated |
| GALE | 180.5504587 | 211.3491124 | 0.227226 | 0.007233 | 0.007233 | Upregulated |
| VDAC2 | 180.5458716 | 211.3550296 | 0.227303 | 0.007213 | 0.007213 | Upregulated |
| COL9A2 | 180.5366972 | 211.3668639 | 0.227457 | 0.007174 | 0.007174 | Upregulated |
| MSH3 | 180.5366972 | 211.3668639 | 0.227457 | 0.007174 | 0.007174 | Upregulated |
| CLRN3 | 180.5366972 | 211.3668639 | 0.227457 | 0.007174 | 0.007174 | Upregulated |
| TNS4 | 180.5229358 | 211.3846154 | 0.227688 | 0.007115 | 0.007115 | Upregulated |
| SHD | 180.5137615 | 211.3964497 | 0.227842 | 0.007076 | 0.007076 | Upregulated |
| NECAP1 | 180.5091743 | 211.4023669 | 0.227919 | 0.007057 | 0.007057 | Upregulated |
| TMEM131 | 180.5091743 | 211.4023669 | 0.227919 | 0.007057 | 0.007057 | Upregulated |
| MRPL24 | 180.4954128 | 211.4201183 | 0.22815 | 0.006999 | 0.006999 | Upregulated |
| ANXA6 | 180.4954128 | 211.4201183 | 0.22815 | 0.006999 | 0.006999 | Upregulated |
| DNAAF3 | 180.4816514 | 211.4378698 | 0.228382 | 0.006941 | 0.006941 | Upregulated |
| KRTAP4-1 | 165.0275229 | 193.3491124 | 0.228501 | 0.012974 | 0.012974 | Upregulated |
| NAT2 | 180.4541284 | 211.4733728 | 0.228844 | 0.006827 | 0.006827 | Upregulated |
| HDAC7 | 180.4357798 | 211.4970414 | 0.229152 | 0.006752 | 0.006752 | Upregulated |
| TGIF2LX | 1.256880734 | 1.473372781 | 0.229275 | 0.042824 | 0.042824 | Upregulated |
| LNX1 | 180.4174312 | 211.5207101 | 0.22946 | 0.006678 | 0.006678 | Upregulated |
| SPSB2 | 180.4036697 | 211.5384615 | 0.229691 | 0.006623 | 0.006623 | Upregulated |
| ABCC10 | 180.3990826 | 211.5443787 | 0.229768 | 0.006604 | 0.006604 | Upregulated |
| RBM19 | 180.3577982 | 211.5976331 | 0.230462 | 0.006442 | 0.006442 | Upregulated |
| DPY19L3 | 180.353211 | 211.6035503 | 0.230539 | 0.006424 | 0.006424 | Upregulated |
| SYNPR | 119.7293578 | 140.4792899 | 0.230581 | 0.044422 | 0.044422 | Upregulated |
| IPO4 | 180.3348624 | 211.6272189 | 0.230847 | 0.006353 | 0.006353 | Upregulated |
| ZNF653 | 180.3211009 | 211.6449704 | 0.231078 | 0.0063 | 0.0063 | Upregulated |
| AC012651.1 | 178.4678899 | 209.4733728 | 0.231102 | 0.006846 | 0.006846 | Upregulated |
| RFWD3 | 180.293578 | 211.6804734 | 0.23154 | 0.006196 | 0.006196 | Upregulated |
| KAZALD1 | 180.2844037 | 211.6923077 | 0.231694 | 0.006161 | 0.006161 | Upregulated |
| CC2D1A | 180.2706422 | 211.7100592 | 0.231925 | 0.00611 | 0.00611 | Upregulated |
| VSIG2 | 180.266055 | 211.7159763 | 0.232002 | 0.006093 | 0.006093 | Upregulated |
| CD44 | 180.266055 | 211.7159763 | 0.232002 | 0.006093 | 0.006093 | Upregulated |
| BRAP | 179.8073394 | 211.2248521 | 0.232328 | 0.005909 | 0.005909 | Upregulated |
| PGAP1 | 180.2431193 | 211.7455621 | 0.232388 | 0.006009 | 0.006009 | Upregulated |
| MZF1 | 180.2385321 | 211.7514793 | 0.232465 | 0.005992 | 0.005992 | Upregulated |
| OSBPL7 | 180.2385321 | 211.7514793 | 0.232465 | 0.005992 | 0.005992 | Upregulated |
| TNFSF15 | 180.2247706 | 211.7692308 | 0.232696 | 0.005942 | 0.005942 | Upregulated |
| SCNN1A | 180.2155963 | 211.7810651 | 0.23285 | 0.005909 | 0.005909 | Upregulated |
| KLF7 | 180.2110092 | 211.7869822 | 0.232927 | 0.005892 | 0.005892 | Upregulated |
| HK1 | 180.206422 | 211.7928994 | 0.233004 | 0.005876 | 0.005876 | Upregulated |
| CES3 | 180.206422 | 211.7928994 | 0.233004 | 0.005876 | 0.005876 | Upregulated |
| DNASE2B | 156.0321101 | 183.3846154 | 0.23303 | 0.015278 | 0.015278 | Upregulated |
| SRPK3 | 180.2018349 | 211.7988166 | 0.233081 | 0.005859 | 0.005859 | Upregulated |
| MLANA | 170.1376147 | 199.9822485 | 0.23317 | 0.009215 | 0.009215 | Upregulated |
| AP1G1 | 180.1926606 | 211.8106509 | 0.233235 | 0.005827 | 0.005827 | Upregulated |
| SUZ12 | 179.4036697 | 210.9053254 | 0.233386 | 0.005868 | 0.005868 | Upregulated |
| GMEB2 | 180.1788991 | 211.8284024 | 0.233466 | 0.005778 | 0.005778 | Upregulated |
| CNN2 | 180.1788991 | 211.8284024 | 0.233466 | 0.005778 | 0.005778 | Upregulated |
| ZNF446 | 180.1743119 | 211.8343195 | 0.233543 | 0.005762 | 0.005762 | Upregulated |
| IL20RA | 180.1743119 | 211.8343195 | 0.233543 | 0.005762 | 0.005762 | Upregulated |
| DDX11 | 180.1697248 | 211.8402367 | 0.23362 | 0.005745 | 0.005745 | Upregulated |
| INS-IGF2 | 1.036697248 | 1.218934911 | 0.233626 | 0.005933 | 0.005933 | Upregulated |
| RPL32 | 180.0275229 | 211.6745562 | 0.23363 | 0.005665 | 0.005665 | Upregulated |
| SMIM15 | 180.1513761 | 211.8639053 | 0.233928 | 0.005681 | 0.005681 | Upregulated |
| ABCG5 | 173.7110092 | 204.3076923 | 0.234054 | 0.00766 | 0.00766 | Upregulated |
| TCP10L | 151.6880734 | 178.4260355 | 0.234218 | 0.015874 | 0.015874 | Upregulated |
| NANS | 180.1330275 | 211.887574 | 0.234236 | 0.005618 | 0.005618 | Upregulated |
| GTPBP6 | 180.1238532 | 211.8994083 | 0.23439 | 0.005586 | 0.005586 | Upregulated |
| CCDC114 | 179.1972477 | 210.8106509 | 0.234399 | 0.005835 | 0.005835 | Upregulated |
| COX6B1 | 180.1100917 | 211.9171598 | 0.234621 | 0.005539 | 0.005539 | Upregulated |
| GNA14 | 180.0779817 | 211.9585799 | 0.235161 | 0.005431 | 0.005431 | Upregulated |
| TECPR1 | 180.0642202 | 211.9763314 | 0.235392 | 0.005385 | 0.005385 | Upregulated |
| SH2D7 | 166.412844 | 195.9467456 | 0.235695 | 0.009809 | 0.009809 | Upregulated |
| ERICH1 | 180.0458716 | 212 | 0.2357 | 0.005325 | 0.005325 | Upregulated |
| BCAN | 180.0366972 | 212.0118343 | 0.235854 | 0.005295 | 0.005295 | Upregulated |
| SHMT2 | 180.0366972 | 212.0118343 | 0.235854 | 0.005295 | 0.005295 | Upregulated |
| CSTF1 | 180.0275229 | 212.0236686 | 0.236008 | 0.005265 | 0.005265 | Upregulated |
| RCC2 | 180.0183486 | 212.035503 | 0.236162 | 0.005235 | 0.005235 | Upregulated |
| C1orf226 | 180.0045872 | 212.0532544 | 0.236393 | 0.005191 | 0.005191 | Upregulated |
| SPARCL1 | 179.9908257 | 212.0710059 | 0.236624 | 0.005147 | 0.005147 | Upregulated |
| RAE1 | 179.9862385 | 212.0769231 | 0.236701 | 0.005132 | 0.005132 | Upregulated |
| TOX | 179.9816514 | 212.0828402 | 0.236778 | 0.005118 | 0.005118 | Upregulated |
| PDAP1 | 179.9770642 | 212.0887574 | 0.236855 | 0.005103 | 0.005103 | Upregulated |
| MGAT5 | 179.9724771 | 212.0946746 | 0.236932 | 0.005089 | 0.005089 | Upregulated |
| RPL10 | 179.9587156 | 212.112426 | 0.237163 | 0.005046 | 0.005046 | Upregulated |
| HOXB9 | 179.9449541 | 212.1301775 | 0.237394 | 0.005003 | 0.005003 | Upregulated |
| WDR55 | 179.9449541 | 212.1301775 | 0.237394 | 0.005003 | 0.005003 | Upregulated |
| VEZF1 | 179.9311927 | 212.147929 | 0.237625 | 0.00496 | 0.00496 | Upregulated |
| JMJD7 | 161.0045872 | 189.8402367 | 0.237684 | 0.011393 | 0.011393 | Upregulated |
| BFSP1 | 179.9220183 | 212.1597633 | 0.237779 | 0.004932 | 0.004932 | Upregulated |
| TMPRSS13 | 179.9174312 | 212.1656805 | 0.237856 | 0.004918 | 0.004918 | Upregulated |
| TMEM229B | 179.912844 | 212.1715976 | 0.237933 | 0.004904 | 0.004904 | Upregulated |
| HSPBP1 | 179.8990826 | 212.1893491 | 0.238164 | 0.004862 | 0.004862 | Upregulated |
| NECTIN2 | 179.8853211 | 212.2071006 | 0.238395 | 0.004821 | 0.004821 | Upregulated |
| HENMT1 | 179.8761468 | 212.2189349 | 0.238549 | 0.004793 | 0.004793 | Upregulated |
| AES | 179.8577982 | 212.2426036 | 0.238858 | 0.004739 | 0.004739 | Upregulated |
| HR | 179.8577982 | 212.2426036 | 0.238858 | 0.004739 | 0.004739 | Upregulated |
| B3GNT6 | 165.3440367 | 195.1301775 | 0.238966 | 0.0093 | 0.0093 | Upregulated |
| GIF | 155.7247706 | 183.7810651 | 0.23899 | 0.013333 | 0.013333 | Upregulated |
| RHOBTB2 | 179.8119266 | 212.3017751 | 0.239628 | 0.004605 | 0.004605 | Upregulated |
| JDP2 | 179.8073394 | 212.3076923 | 0.239705 | 0.004592 | 0.004592 | Upregulated |
| TRIM68 | 179.8073394 | 212.3076923 | 0.239705 | 0.004592 | 0.004592 | Upregulated |
| KIF19 | 177.0366972 | 209.0473373 | 0.239781 | 0.00525 | 0.00525 | Upregulated |
| BANK1 | 178.8715596 | 211.2307692 | 0.239896 | 0.004766 | 0.004766 | Upregulated |
| RBM10 | 179.7706422 | 212.3550296 | 0.240321 | 0.004488 | 0.004488 | Upregulated |
| RILPL1 | 179.766055 | 212.3609467 | 0.240398 | 0.004475 | 0.004475 | Upregulated |
| PLEKHG3 | 179.7568807 | 212.3727811 | 0.240552 | 0.004449 | 0.004449 | Upregulated |
| PLEKHF2 | 179.7522936 | 212.3786982 | 0.240629 | 0.004436 | 0.004436 | Upregulated |
| ZNF444 | 179.7385321 | 212.3964497 | 0.24086 | 0.004398 | 0.004398 | Upregulated |
| FBP1 | 179.7385321 | 212.3964497 | 0.24086 | 0.004398 | 0.004398 | Upregulated |
| HABP2 | 134.853211 | 159.3727811 | 0.241015 | 0.023593 | 0.023593 | Upregulated |
| CCDC148 | 165.2155963 | 195.295858 | 0.241311 | 0.007912 | 0.007912 | Upregulated |
| MOB2 | 179.7018349 | 212.443787 | 0.241476 | 0.004298 | 0.004298 | Upregulated |
| LRRTM1 | 114.0688073 | 134.8994083 | 0.24198 | 0.04132 | 0.04132 | Upregulated |
| OS9 | 179.6513761 | 212.5088757 | 0.242323 | 0.004164 | 0.004164 | Upregulated |
| SLC22A2 | 138 | 163.2781065 | 0.242663 | 0.0158 | 0.0158 | Upregulated |
| ATP5G2 | 179.6284404 | 212.5384615 | 0.242708 | 0.004104 | 0.004104 | Upregulated |
| DCT | 111.8394495 | 132.3431953 | 0.242855 | 0.031883 | 0.031883 | Upregulated |
| EVX1 | 173.1880734 | 204.9822485 | 0.243159 | 0.005423 | 0.005423 | Upregulated |
| INF2 | 179.587156 | 212.591716 | 0.243401 | 0.003998 | 0.003998 | Upregulated |
| KLK6 | 177.7385321 | 210.4142012 | 0.243476 | 0.004373 | 0.004373 | Upregulated |
| GSDMA | 179.5825688 | 212.5976331 | 0.243478 | 0.003986 | 0.003986 | Upregulated |
| PRH1 | 154.6238532 | 183.0769231 | 0.243687 | 0.011868 | 0.011868 | Upregulated |
| MRFAP1 | 179.5642202 | 212.6213018 | 0.243786 | 0.00394 | 0.00394 | Upregulated |
| HGFAC | 101.8394495 | 120.5976331 | 0.243905 | 0.02503 | 0.02503 | Upregulated |
| ZBTB12 | 179.5550459 | 212.6331361 | 0.24394 | 0.003917 | 0.003917 | Upregulated |
| ADRM1 | 179.5458716 | 212.6449704 | 0.244094 | 0.003895 | 0.003895 | Upregulated |
| POLR3B | 179.5091743 | 212.6923077 | 0.24471 | 0.003805 | 0.003805 | Upregulated |
| MAD2L2 | 179.5045872 | 212.6982249 | 0.244787 | 0.003794 | 0.003794 | Upregulated |
| EVI5L | 179.5045872 | 212.6982249 | 0.244787 | 0.003794 | 0.003794 | Upregulated |
| MVK | 179.4770642 | 212.7337278 | 0.245249 | 0.003728 | 0.003728 | Upregulated |
| ADAMTS15 | 179.4587156 | 212.7573964 | 0.245557 | 0.003684 | 0.003684 | Upregulated |
| TMEM105 | 179.4495413 | 212.7692308 | 0.245711 | 0.003663 | 0.003663 | Upregulated |
| MKNK2 | 179.4357798 | 212.7869822 | 0.245942 | 0.003631 | 0.003631 | Upregulated |
| PLA2G4F | 179.4082569 | 212.8224852 | 0.246404 | 0.003568 | 0.003568 | Upregulated |
| CYSTM1 | 179.3944954 | 212.8402367 | 0.246635 | 0.003536 | 0.003536 | Upregulated |
| WASHC1 | 179.3807339 | 212.8579882 | 0.246866 | 0.003505 | 0.003505 | Upregulated |
| ZBTB7C | 179.3715596 | 212.8698225 | 0.24702 | 0.003485 | 0.003485 | Upregulated |
| ALPK3 | 179.3669725 | 212.8757396 | 0.247097 | 0.003474 | 0.003474 | Upregulated |
| AFAP1 | 179.3623853 | 212.8816568 | 0.247174 | 0.003464 | 0.003464 | Upregulated |
| KIF16B | 179.0137615 | 212.5502959 | 0.247734 | 0.003314 | 0.003314 | Upregulated |
| ERLIN2 | 179.2889908 | 212.9763314 | 0.248406 | 0.003305 | 0.003305 | Upregulated |
| STOML3 | 154.3761468 | 183.3964497 | 0.248516 | 0.010302 | 0.010302 | Upregulated |
| PLA2G3 | 133.6880734 | 158.8994083 | 0.249243 | 0.020725 | 0.020725 | Upregulated |
| PPP2R1A | 179.2155963 | 213.0710059 | 0.249638 | 0.003152 | 0.003152 | Upregulated |
| ZNF497 | 179.1972477 | 213.0946746 | 0.249946 | 0.003114 | 0.003114 | Upregulated |
| KRT75 | 137.6330275 | 163.7514793 | 0.250681 | 0.013707 | 0.013707 | Upregulated |
| FGF19 | 146.6559633 | 174.5029586 | 0.250816 | 0.0147 | 0.0147 | Upregulated |
| RAPGEF3 | 179.1376147 | 213.1715976 | 0.250947 | 0.002996 | 0.002996 | Upregulated |
| SLC6A7 | 179.1376147 | 213.1715976 | 0.250947 | 0.002996 | 0.002996 | Upregulated |
| NR1H2 | 179.1330275 | 213.1775148 | 0.251024 | 0.002987 | 0.002987 | Upregulated |
| DRP2 | 179.1284404 | 213.183432 | 0.251101 | 0.002978 | 0.002978 | Upregulated |
| ITLN2 | 111.5229358 | 132.7514793 | 0.251387 | 0.036068 | 0.036068 | Upregulated |
| PRUNE2 | 179.1009174 | 213.2189349 | 0.251563 | 0.002926 | 0.002926 | Upregulated |
| CWH43 | 141.6422018 | 168.6686391 | 0.251941 | 0.013839 | 0.013839 | Upregulated |
| CDK9 | 179.0688073 | 213.260355 | 0.252102 | 0.002865 | 0.002865 | Upregulated |
| KRT83 | 112.9495413 | 134.5266272 | 0.252213 | 0.025396 | 0.025396 | Upregulated |
| CYP39A1 | 179.0366972 | 213.3017751 | 0.252641 | 0.002806 | 0.002806 | Upregulated |
| SPINK2 | 124.1559633 | 147.9408284 | 0.252867 | 0.022433 | 0.022433 | Upregulated |
| USP5 | 178.9862385 | 213.3668639 | 0.253487 | 0.002714 | 0.002714 | Upregulated |
| SNX19 | 178.9770642 | 213.3786982 | 0.253641 | 0.002698 | 0.002698 | Upregulated |
| CCT3 | 178.9266055 | 213.443787 | 0.254488 | 0.00261 | 0.00261 | Upregulated |
| BTBD17 | 45.53669725 | 54.32544379 | 0.254598 | 0.034271 | 0.034271 | Upregulated |
| SHF | 178.8990826 | 213.4792899 | 0.25495 | 0.002563 | 0.002563 | Upregulated |
| FAM184B | 173.3990826 | 206.9585799 | 0.255246 | 0.003364 | 0.003364 | Upregulated |
| PCOTH | 168 | 200.5207101 | 0.25529 | 0.004513 | 0.004513 | Upregulated |
| EXD3 | 178.8761468 | 213.5088757 | 0.255335 | 0.002525 | 0.002525 | Upregulated |
| SLC25A32 | 178.853211 | 213.5384615 | 0.25572 | 0.002487 | 0.002487 | Upregulated |
| GABBR1 | 178.8348624 | 213.5621302 | 0.256028 | 0.002457 | 0.002457 | Upregulated |
| SPTBN5 | 178.8256881 | 213.5739645 | 0.256182 | 0.002442 | 0.002442 | Upregulated |
| TNFRSF11A | 178.7981651 | 213.6094675 | 0.256644 | 0.002398 | 0.002398 | Upregulated |
| RPS19 | 178.793578 | 213.6153846 | 0.256721 | 0.002391 | 0.002391 | Upregulated |
| UGT1A8 | 148.0321101 | 176.8757396 | 0.256826 | 0.008685 | 0.008685 | Upregulated |
| FXYD4 | 165.2477064 | 197.4497041 | 0.256855 | 0.00467 | 0.00467 | Upregulated |
| URGCP-MRPS24 | 86.74770642 | 103.6804734 | 0.257247 | 0.046555 | 0.046555 | Upregulated |
| UBQLNL | 153.9266055 | 183.9763314 | 0.257278 | 0.007627 | 0.007627 | Upregulated |
| C1QTNF3-AMACR | 91.27981651 | 109.1005917 | 0.257291 | 0.047671 | 0.047671 | Upregulated |
| PLCXD1 | 178.7522936 | 213.6686391 | 0.257413 | 0.002326 | 0.002326 | Upregulated |
| FRMPD1 | 159.0779817 | 190.1656805 | 0.257523 | 0.005396 | 0.005396 | Upregulated |
| KCNMB2 | 104.8669725 | 125.3609467 | 0.257528 | 0.043752 | 0.043752 | Upregulated |
| QSOX1 | 178.7431193 | 213.6804734 | 0.257567 | 0.002312 | 0.002312 | Upregulated |
| ATOH8 | 178.7385321 | 213.6863905 | 0.257644 | 0.002305 | 0.002305 | Upregulated |
| HGD | 178.733945 | 213.6923077 | 0.257721 | 0.002298 | 0.002298 | Upregulated |
| TTC9 | 178.7293578 | 213.6982249 | 0.257798 | 0.002291 | 0.002291 | Upregulated |
| TLE2 | 178.6972477 | 213.739645 | 0.258337 | 0.002243 | 0.002243 | Upregulated |
| ZNF296 | 178.6926606 | 213.7455621 | 0.258414 | 0.002236 | 0.002236 | Upregulated |
| LY6G6C | 172.3027523 | 206.1242604 | 0.258569 | 0.003133 | 0.003133 | Upregulated |
| ASIC4 | 127.7706422 | 152.8639053 | 0.258691 | 0.016264 | 0.016264 | Upregulated |
| CLCA2 | 115.6330275 | 138.3668639 | 0.258945 | 0.040644 | 0.040644 | Upregulated |
| FZD9 | 167.7798165 | 200.8047337 | 0.259224 | 0.003793 | 0.003793 | Upregulated |
| RPL41 | 178.6330275 | 213.8224852 | 0.259415 | 0.002148 | 0.002148 | Upregulated |
| CDK11A | 178.6055046 | 213.8579882 | 0.259877 | 0.002109 | 0.002109 | Upregulated |
| PCID2 | 178.5963303 | 213.8698225 | 0.26003 | 0.002096 | 0.002096 | Upregulated |
| NEK6 | 178.5917431 | 213.8757396 | 0.260107 | 0.00209 | 0.00209 | Upregulated |
| BICDL1 | 178.5688073 | 213.9053254 | 0.260492 | 0.002058 | 0.002058 | Upregulated |
| TTC9B | 171.2889908 | 205.1893491 | 0.260523 | 0.003152 | 0.003152 | Upregulated |
| SLC25A6 | 178.5504587 | 213.9289941 | 0.2608 | 0.002033 | 0.002033 | Upregulated |
| MYCNOS | 89.86697248 | 107.6745562 | 0.260814 | 0.032477 | 0.032477 | Upregulated |
| UBE2M | 178.4862385 | 214.0118343 | 0.261878 | 0.001947 | 0.001947 | Upregulated |
| FITM2 | 178.4770642 | 214.0236686 | 0.262032 | 0.001935 | 0.001935 | Upregulated |
| ALG1L | 178.4724771 | 214.0295858 | 0.262109 | 0.001929 | 0.001929 | Upregulated |
| DUSP27 | 172.0917431 | 206.3964497 | 0.26224 | 0.002789 | 0.002789 | Upregulated |
| CCDC120 | 178.4495413 | 214.0591716 | 0.262493 | 0.001899 | 0.001899 | Upregulated |
| B3GNTL1 | 178.4082569 | 214.112426 | 0.263186 | 0.001847 | 0.001847 | Upregulated |
| HNF4G | 178.4036697 | 214.1183432 | 0.263263 | 0.001841 | 0.001841 | Upregulated |
| GMPR | 178.3853211 | 214.1420118 | 0.263571 | 0.001818 | 0.001818 | Upregulated |
| C19orf44 | 178.3669725 | 214.1656805 | 0.263879 | 0.001796 | 0.001796 | Upregulated |
| TUBA4A | 178.3623853 | 214.1715976 | 0.263956 | 0.00179 | 0.00179 | Upregulated |
| CXCL17 | 138.6238532 | 166.4911243 | 0.26427 | 0.011477 | 0.011477 | Upregulated |
| HES4 | 178.3348624 | 214.2071006 | 0.264418 | 0.001757 | 0.001757 | Upregulated |
| ABLIM3 | 178.3302752 | 214.2130178 | 0.264495 | 0.001752 | 0.001752 | Upregulated |
| SCGB2A1 | 140.2293578 | 168.4615385 | 0.264631 | 0.009372 | 0.009372 | Upregulated |
| PTCHD4 | 162.1651376 | 194.8579882 | 0.264959 | 0.004321 | 0.004321 | Upregulated |
| DUOX1 | 178.2889908 | 214.2662722 | 0.265187 | 0.001703 | 0.001703 | Upregulated |
| ARHGEF37 | 178.2889908 | 214.2662722 | 0.265187 | 0.001703 | 0.001703 | Upregulated |
| ZSWIM1 | 178.2752294 | 214.2840237 | 0.265418 | 0.001687 | 0.001687 | Upregulated |
| PSMD9 | 178.2614679 | 214.3017751 | 0.265649 | 0.001672 | 0.001672 | Upregulated |
| LYPD6B | 178.2614679 | 214.3017751 | 0.265649 | 0.001672 | 0.001672 | Upregulated |
| VHL | 178.2431193 | 214.3254438 | 0.265957 | 0.001651 | 0.001651 | Upregulated |
| MORC4 | 178.233945 | 214.3372781 | 0.266111 | 0.00164 | 0.00164 | Upregulated |
| REN | 169.1651376 | 203.4615385 | 0.266324 | 0.002788 | 0.002788 | Upregulated |
| FAM91A1 | 178.206422 | 214.3727811 | 0.266572 | 0.00161 | 0.00161 | Upregulated |
| PTPA | 178.1834862 | 214.4023669 | 0.266957 | 0.001585 | 0.001585 | Upregulated |
| CARD6 | 178.1834862 | 214.4023669 | 0.266957 | 0.001585 | 0.001585 | Upregulated |
| OCIAD2 | 178.1834862 | 214.4023669 | 0.266957 | 0.001585 | 0.001585 | Upregulated |
| PHKA1 | 178.1651376 | 214.4260355 | 0.267265 | 0.001565 | 0.001565 | Upregulated |
| PRSS2 | 160.293578 | 192.9230769 | 0.267309 | 0.004283 | 0.004283 | Upregulated |
| MRPS2 | 178.1559633 | 214.4378698 | 0.267419 | 0.001555 | 0.001555 | Upregulated |
| TEX19 | 111.6513761 | 134.3905325 | 0.26743 | 0.032806 | 0.032806 | Upregulated |
| KNDC1 | 173.5733945 | 208.9881657 | 0.267875 | 0.001986 | 0.001986 | Upregulated |
| UBAP2 | 178.1146789 | 214.4911243 | 0.268112 | 0.001512 | 0.001512 | Upregulated |
| MAP3K10 | 178.1146789 | 214.4911243 | 0.268112 | 0.001512 | 0.001512 | Upregulated |
| RRBP1 | 178.0917431 | 214.5207101 | 0.268496 | 0.001488 | 0.001488 | Upregulated |
| CCDC71L | 178.0917431 | 214.5207101 | 0.268496 | 0.001488 | 0.001488 | Upregulated |
| DNAAF5 | 178.0642202 | 214.556213 | 0.268958 | 0.00146 | 0.00146 | Upregulated |
| DPP3 | 178.0550459 | 214.5680473 | 0.269112 | 0.001451 | 0.001451 | Upregulated |
| RPL18 | 178.0366972 | 214.591716 | 0.26942 | 0.001433 | 0.001433 | Upregulated |
| TMEM40 | 121.9449541 | 147 | 0.269586 | 0.026947 | 0.026947 | Upregulated |
| NPIPB7 | 82.61009174 | 99.59171598 | 0.269708 | 0.039367 | 0.039367 | Upregulated |
| CEACAM19 | 177.9862385 | 214.6568047 | 0.270266 | 0.001383 | 0.001383 | Upregulated |
| TCN1 | 176.1559633 | 212.4556213 | 0.270308 | 0.001545 | 0.001545 | Upregulated |
| KRT6B | 177.0504587 | 213.5798817 | 0.270615 | 0.001444 | 0.001444 | Upregulated |
| ZFP36L1 | 177.9633028 | 214.6863905 | 0.270651 | 0.001362 | 0.001362 | Upregulated |
| NPY4R | 148.1834862 | 178.7633136 | 0.270666 | 0.006246 | 0.006246 | Upregulated |
| MID1IP1 | 177.9587156 | 214.6923077 | 0.270728 | 0.001357 | 0.001357 | Upregulated |
| MED14 | 177.9266055 | 214.7337278 | 0.271267 | 0.001327 | 0.001327 | Upregulated |
| C12orf50 | 39.94954128 | 48.21893491 | 0.271421 | 0.021071 | 0.021071 | Upregulated |
| HOXD12 | 70.01376147 | 84.52662722 | 0.271767 | 0.029861 | 0.029861 | Upregulated |
| BIN3 | 177.8715596 | 214.8047337 | 0.27219 | 0.001277 | 0.001277 | Upregulated |
| LINC00238 | 131.0825688 | 158.3254438 | 0.272417 | 0.011014 | 0.011014 | Upregulated |
| UBR5 | 177.8577982 | 214.8224852 | 0.272421 | 0.001265 | 0.001265 | Upregulated |
| MAP3K6 | 177.8256881 | 214.8639053 | 0.272959 | 0.001237 | 0.001237 | Upregulated |
| AC008750.8 | 131.0504587 | 158.3668639 | 0.273148 | 0.009345 | 0.009345 | Upregulated |
| AIFM3 | 177.7981651 | 214.8994083 | 0.273421 | 0.001214 | 0.001214 | Upregulated |
| LAMTOR2 | 177.7844037 | 214.9171598 | 0.273652 | 0.001202 | 0.001202 | Upregulated |
| C14orf177 | 1.02293578 | 1.236686391 | 0.273764 | 0.03569 | 0.03569 | Upregulated |
| FAM221B | 154.766055 | 187.147929 | 0.27409 | 0.004109 | 0.004109 | Upregulated |
| FAM71E1 | 177.7568807 | 214.9526627 | 0.274114 | 0.001179 | 0.001179 | Upregulated |
| GPR37 | 177.7522936 | 214.9585799 | 0.274191 | 0.001175 | 0.001175 | Upregulated |
| TMEM92 | 177.6926606 | 215.035503 | 0.275191 | 0.001127 | 0.001127 | Upregulated |
| AVIL | 177.6880734 | 215.0414201 | 0.275268 | 0.001123 | 0.001123 | Upregulated |
| MVB12B | 177.6376147 | 215.1065089 | 0.276114 | 0.001084 | 0.001084 | Upregulated |
| GLYATL1 | 173.0917431 | 209.6094675 | 0.276167 | 0.001442 | 0.001442 | Upregulated |
| KIF21B | 177.6284404 | 215.1183432 | 0.276268 | 0.001077 | 0.001077 | Upregulated |
| RPL11 | 177.6100917 | 215.1420118 | 0.276576 | 0.001063 | 0.001063 | Upregulated |
| NUP205 | 177.6009174 | 215.1538462 | 0.27673 | 0.001056 | 0.001056 | Upregulated |
| RTP4 | 177.5504587 | 215.2189349 | 0.277576 | 0.001019 | 0.001019 | Upregulated |
| OAZ3 | 177.5412844 | 215.2307692 | 0.27773 | 0.001013 | 0.001013 | Upregulated |
| TMTC2 | 177.5412844 | 215.2307692 | 0.27773 | 0.001013 | 0.001013 | Upregulated |
| SLCO1B3 | 146.1513761 | 177.2248521 | 0.278118 | 0.003994 | 0.003994 | Upregulated |
| TMEM143 | 177.4862385 | 215.3017751 | 0.278653 | 0.000974 | 0.000974 | Upregulated |
| LYPD3 | 177.4724771 | 215.3195266 | 0.278884 | 0.000964 | 0.000964 | Upregulated |
| KCNB2 | 75.66972477 | 91.81656805 | 0.279038 | 0.042024 | 0.042024 | Upregulated |
| FAM214B | 177.4587156 | 215.3372781 | 0.279115 | 0.000955 | 0.000955 | Upregulated |
| LMX1B | 89.96330275 | 109.1715976 | 0.279189 | 0.048382 | 0.048382 | Upregulated |
| GALNT4 | 177.4357798 | 215.3668639 | 0.279499 | 0.00094 | 0.00094 | Upregulated |
| RPL6 | 177.4311927 | 215.3727811 | 0.279576 | 0.000936 | 0.000936 | Upregulated |
| ACSM1 | 175.587156 | 213.1893491 | 0.279948 | 0.001036 | 0.001036 | Upregulated |
| CYSRT1 | 177.3486239 | 215.4792899 | 0.280961 | 0.000883 | 0.000883 | Upregulated |
| GIPC1 | 177.3348624 | 215.4970414 | 0.281192 | 0.000874 | 0.000874 | Upregulated |
| GNGT1 | 81.02293578 | 98.5443787 | 0.282443 | 0.029984 | 0.029984 | Upregulated |
| ARSJ | 176.9266055 | 215.2485207 | 0.282852 | 0.000789 | 0.000789 | Upregulated |
| CDK5RAP1 | 177.233945 | 215.6272189 | 0.282884 | 0.000813 | 0.000813 | Upregulated |
| MYBPC1 | 169.9954128 | 206.8579882 | 0.283145 | 0.001257 | 0.001257 | Upregulated |
| SLC27A1 | 177.1743119 | 215.704142 | 0.283884 | 0.000779 | 0.000779 | Upregulated |
| RPRD1B | 177.1697248 | 215.7100592 | 0.283961 | 0.000776 | 0.000776 | Upregulated |
| CPA6 | 162.8761468 | 198.3136095 | 0.284008 | 0.002026 | 0.002026 | Upregulated |
| MTUS2 | 160.2568807 | 195.1420118 | 0.284138 | 0.002105 | 0.002105 | Upregulated |
| REP15 | 162.8577982 | 198.3372781 | 0.284343 | 0.002001 | 0.002001 | Upregulated |
| STAT6 | 177.1055046 | 215.7928994 | 0.285038 | 0.000741 | 0.000741 | Upregulated |
| ACPT | 137.646789 | 167.7514793 | 0.285355 | 0.004675 | 0.004675 | Upregulated |
| TNFSF9 | 177.0825688 | 215.8224852 | 0.285423 | 0.000729 | 0.000729 | Upregulated |
| C11orf49 | 177.0825688 | 215.8224852 | 0.285423 | 0.000729 | 0.000729 | Upregulated |
| ACOT8 | 177.0779817 | 215.8284024 | 0.2855 | 0.000726 | 0.000726 | Upregulated |
| MB | 177.0733945 | 215.8343195 | 0.285577 | 0.000724 | 0.000724 | Upregulated |
| MED30 | 177.0642202 | 215.8461538 | 0.285731 | 0.000719 | 0.000719 | Upregulated |
| CAPN14 | 148.2293578 | 180.7928994 | 0.286507 | 0.003743 | 0.003743 | Upregulated |
| ART3 | 168.0183486 | 204.9408284 | 0.286589 | 0.001285 | 0.001285 | Upregulated |
| RAB5B | 177 | 215.9289941 | 0.286808 | 0.000686 | 0.000686 | Upregulated |
| ANKFN1 | 149.8807339 | 182.8579882 | 0.286909 | 0.003355 | 0.003355 | Upregulated |
| GRM7 | 133.5688073 | 163.0118343 | 0.287394 | 0.006378 | 0.006378 | Upregulated |
| CYB5R2 | 176.9357798 | 216.0118343 | 0.287885 | 0.000655 | 0.000655 | Upregulated |
| AACS | 176.9266055 | 216.0236686 | 0.288038 | 0.00065 | 0.00065 | Upregulated |
| DDX54 | 176.9174312 | 216.035503 | 0.288192 | 0.000646 | 0.000646 | Upregulated |
| SYTL5 | 175.087156 | 213.8343195 | 0.28842 | 0.000726 | 0.000726 | Upregulated |
| SHC2 | 176.8990826 | 216.0591716 | 0.2885 | 0.000637 | 0.000637 | Upregulated |
| PRR13 | 176.8577982 | 216.112426 | 0.289192 | 0.000618 | 0.000618 | Upregulated |
| CFAP73 | 175.0412844 | 213.8934911 | 0.289197 | 0.0007 | 0.0007 | Upregulated |
| NTRK2 | 175.8807339 | 215.0887574 | 0.290335 | 0.000627 | 0.000627 | Upregulated |
| HSD3B1 | 124.8623853 | 152.7633136 | 0.290959 | 0.010263 | 0.010263 | Upregulated |
| KRT10 | 176.7201835 | 216.2899408 | 0.2915 | 0.000559 | 0.000559 | Upregulated |
| GABRB2 | 140.5825688 | 172.0710059 | 0.291586 | 0.003759 | 0.003759 | Upregulated |
| CORO7-PAM16 | 113.6100917 | 139.1420118 | 0.292467 | 0.009539 | 0.009539 | Upregulated |
| CHRM1 | 169.4266055 | 207.591716 | 0.293088 | 0.000851 | 0.000851 | Upregulated |
| ACOX2 | 176.6238532 | 216.4142012 | 0.293115 | 0.00052 | 0.00052 | Upregulated |
| GGT7 | 176.6100917 | 216.4319527 | 0.293346 | 0.000515 | 0.000515 | Upregulated |
| ZNF784 | 176.5458716 | 216.5147929 | 0.294423 | 0.000491 | 0.000491 | Upregulated |
| NAA60 | 176.5412844 | 216.5207101 | 0.294499 | 0.000489 | 0.000489 | Upregulated |
| ASXL1 | 176.5091743 | 216.5621302 | 0.295038 | 0.000477 | 0.000477 | Upregulated |
| IL23A | 176.5045872 | 216.5680473 | 0.295115 | 0.000476 | 0.000476 | Upregulated |
| RPS6KA2 | 176.4770642 | 216.6035503 | 0.295576 | 0.000466 | 0.000466 | Upregulated |
| CADPS | 176.4541284 | 216.6331361 | 0.295961 | 0.000458 | 0.000458 | Upregulated |
| SUOX | 176.4266055 | 216.6686391 | 0.296422 | 0.000449 | 0.000449 | Upregulated |
| SLITRK6 | 173.6926606 | 213.3609467 | 0.296759 | 0.000539 | 0.000539 | Upregulated |
| FIZ1 | 176.4036697 | 216.6982249 | 0.296807 | 0.000441 | 0.000441 | Upregulated |
| CDC42EP1 | 176.3623853 | 216.7514793 | 0.297499 | 0.000428 | 0.000428 | Upregulated |
| MVB12A | 176.3027523 | 216.8284024 | 0.298499 | 0.000409 | 0.000409 | Upregulated |
| TMEM71 | 176.2844037 | 216.852071 | 0.298806 | 0.000403 | 0.000403 | Upregulated |
| PPP2R2C | 174.4587156 | 214.6449704 | 0.299067 | 0.000458 | 0.000458 | Upregulated |
| RACK1 | 176.2568807 | 216.887574 | 0.299268 | 0.000395 | 0.000395 | Upregulated |
| DLEC1 | 176.2018349 | 216.9585799 | 0.300191 | 0.000379 | 0.000379 | Upregulated |
| FOXD4 | 160.2431193 | 197.3372781 | 0.300401 | 0.001205 | 0.001205 | Upregulated |
| RIPK2 | 176.1743119 | 216.9940828 | 0.300652 | 0.000371 | 0.000371 | Upregulated |
| FAM110A | 176.1743119 | 216.9940828 | 0.300652 | 0.000371 | 0.000371 | Upregulated |
| COPZ1 | 176.1559633 | 217.0177515 | 0.30096 | 0.000366 | 0.000366 | Upregulated |
| ARHGAP22 | 176.146789 | 217.0295858 | 0.301114 | 0.000363 | 0.000363 | Upregulated |
| C3orf52 | 175.3944954 | 216.1183432 | 0.301218 | 0.000369 | 0.000369 | Upregulated |
| MSLNL | 88.64678899 | 109.2485207 | 0.301473 | 0.019651 | 0.019651 | Upregulated |
| GRHL3 | 176.1100917 | 217.0769231 | 0.301729 | 0.000353 | 0.000353 | Upregulated |
| HAS3 | 176.0779817 | 217.1183432 | 0.302267 | 0.000345 | 0.000345 | Upregulated |
| FAM178B | 173.3440367 | 213.8106509 | 0.302696 | 0.000413 | 0.000413 | Upregulated |
| BHMG1 | 62.12385321 | 76.62721893 | 0.30271 | 0.036284 | 0.036284 | Upregulated |
| KRT81 | 160.9220183 | 198.6449704 | 0.303831 | 0.000925 | 0.000925 | Upregulated |
| SP8 | 136.7752294 | 168.8757396 | 0.304155 | 0.003286 | 0.003286 | Upregulated |
| CLEC18B | 121.2614679 | 149.7751479 | 0.304677 | 0.00608 | 0.00608 | Upregulated |
| DCP1B | 175.9266055 | 217.3136095 | 0.304805 | 0.000307 | 0.000307 | Upregulated |
| PADI3 | 158.2568807 | 195.5502959 | 0.305271 | 0.000999 | 0.000999 | Upregulated |
| KCNV2 | 113.8440367 | 140.6745562 | 0.305303 | 0.008907 | 0.008907 | Upregulated |
| FDFT1 | 175.8853211 | 217.3668639 | 0.305497 | 0.000297 | 0.000297 | Upregulated |
| ELFN2 | 164.3119266 | 203.0650888 | 0.305505 | 0.000708 | 0.000708 | Upregulated |
| IL36RN | 73.23853211 | 90.5147929 | 0.305551 | 0.035219 | 0.035219 | Upregulated |
| KLK7 | 161.6743119 | 199.8639053 | 0.305927 | 0.000791 | 0.000791 | Upregulated |
| HOXD13 | 159.9220183 | 197.7514793 | 0.30632 | 0.000879 | 0.000879 | Upregulated |
| MFSD10 | 175.8348624 | 217.4319527 | 0.306343 | 0.000286 | 0.000286 | Upregulated |
| GJB5 | 164.2614679 | 203.1301775 | 0.30641 | 0.000682 | 0.000682 | Upregulated |
| SLC6A14 | 173.1238532 | 214.0946746 | 0.306444 | 0.000349 | 0.000349 | Upregulated |
| KAAG1 | 64.19724771 | 79.40828402 | 0.306778 | 0.021654 | 0.021654 | Upregulated |
| TRIM50 | 82.19724771 | 101.6804734 | 0.306881 | 0.024331 | 0.024331 | Upregulated |
| CCDC149 | 175.7844037 | 217.4970414 | 0.307189 | 0.000275 | 0.000275 | Upregulated |
| TUBB4B | 175.7798165 | 217.5029586 | 0.307266 | 0.000274 | 0.000274 | Upregulated |
| DYRK4 | 175.7752294 | 217.5088757 | 0.307342 | 0.000273 | 0.000273 | Upregulated |
| C2orf70 | 175.7477064 | 217.5443787 | 0.307804 | 0.000267 | 0.000267 | Upregulated |
| ACY1 | 175.733945 | 217.5621302 | 0.308035 | 0.000264 | 0.000264 | Upregulated |
| UBTD2 | 175.7155963 | 217.5857988 | 0.308342 | 0.000261 | 0.000261 | Upregulated |
| ANKHD1-EIF4EBP3 | 175.6880734 | 217.6213018 | 0.308804 | 0.000255 | 0.000255 | Upregulated |
| ASCL5 | 170.2568807 | 211.0118343 | 0.309611 | 0.000371 | 0.000371 | Upregulated |
| CACNA2D2 | 175.6238532 | 217.704142 | 0.30988 | 0.000243 | 0.000243 | Upregulated |
| SLC48A1 | 175.6055046 | 217.7278107 | 0.310188 | 0.000239 | 0.000239 | Upregulated |
| KRT16 | 155.4220183 | 192.7278107 | 0.310374 | 0.000893 | 0.000893 | Upregulated |
| GFI1B | 135.6880734 | 168.2662722 | 0.310452 | 0.00239 | 0.00239 | Upregulated |
| CKAP4 | 175.5825688 | 217.7573964 | 0.310572 | 0.000235 | 0.000235 | Upregulated |
| SLC35B1 | 175.5733945 | 217.7692308 | 0.310726 | 0.000233 | 0.000233 | Upregulated |
| CA8 | 175.5412844 | 217.8106509 | 0.311264 | 0.000227 | 0.000227 | Upregulated |
| ARFGEF3 | 175.4954128 | 217.8698225 | 0.312033 | 0.000219 | 0.000219 | Upregulated |
| KLK10 | 175.4633028 | 217.9112426 | 0.312571 | 0.000214 | 0.000214 | Upregulated |
| DMPK | 175.3990826 | 217.9940828 | 0.313648 | 0.000203 | 0.000203 | Upregulated |
| VSIG8 | 164.7247706 | 204.7455621 | 0.313775 | 0.000466 | 0.000466 | Upregulated |
| AC120057.3 | 1.26146789 | 1.568047337 | 0.313866 | 0.024281 | 0.024281 | Upregulated |
| RPL36A-HNRNPH2 | 86.99541284 | 108.1538462 | 0.314074 | 0.022648 | 0.022648 | Upregulated |
| OPA3 | 175.3715596 | 218.0295858 | 0.314109 | 0.000199 | 0.000199 | Upregulated |
| TTC16 | 162.0321101 | 201.5976331 | 0.315199 | 0.000545 | 0.000545 | Upregulated |
| SPATA18 | 175.2889908 | 218.1360947 | 0.315493 | 0.000186 | 0.000186 | Upregulated |
| IRS4 | 1.302752294 | 1.621301775 | 0.31559 | 0.046876 | 0.046876 | Upregulated |
| GJB6 | 104.8853211 | 130.6094675 | 0.316447 | 0.00698 | 0.00698 | Upregulated |
| PADI1 | 175.1422018 | 218.3254438 | 0.317954 | 0.000166 | 0.000166 | Upregulated |
| ETV5 | 175.0963303 | 218.3846154 | 0.318722 | 0.00016 | 0.00016 | Upregulated |
| ABCA4 | 159.2431193 | 198.6272189 | 0.318832 | 0.000552 | 0.000552 | Upregulated |
| MAL2 | 175.0779817 | 218.408284 | 0.31903 | 0.000157 | 0.000157 | Upregulated |
| MROH7 | 171.4862385 | 213.9408284 | 0.319119 | 0.000212 | 0.000212 | Upregulated |
| TMC2 | 142.4541284 | 177.8579882 | 0.320228 | 0.001242 | 0.001242 | Upregulated |
| NYX | 9.027522936 | 11.27218935 | 0.320366 | 0.049416 | 0.049416 | Upregulated |
| CDK5R2 | 127.4220183 | 159.1360947 | 0.320647 | 0.002575 | 0.002575 | Upregulated |
| C1orf168 | 111.0825688 | 138.7514793 | 0.320871 | 0.008305 | 0.008305 | Upregulated |
| CCDC129 | 118.3256881 | 147.8994083 | 0.321853 | 0.00348 | 0.00348 | Upregulated |
| SOX13 | 174.8853211 | 218.6568047 | 0.322259 | 0.000135 | 0.000135 | Upregulated |
| HEPHL1 | 163.3577982 | 204.295858 | 0.322625 | 0.000325 | 0.000325 | Upregulated |
| PAX9 | 171.266055 | 214.2248521 | 0.322887 | 0.000178 | 0.000178 | Upregulated |
| ALDH3A1 | 174.8027523 | 218.7633136 | 0.323643 | 0.000126 | 0.000126 | Upregulated |
| IGFL4 | 172.1100917 | 215.4023669 | 0.323702 | 0.000159 | 0.000159 | Upregulated |
| SPRR2E | 21.02752294 | 26.32544379 | 0.324179 | 0.020307 | 0.020307 | Upregulated |
| BAX | 174.7706422 | 218.8047337 | 0.324181 | 0.000123 | 0.000123 | Upregulated |
| INHBE | 172.9495413 | 216.591716 | 0.324627 | 0.000141 | 0.000141 | Upregulated |
| AC137834.1 | 93.10091743 | 116.6390533 | 0.325184 | 0.019746 | 0.019746 | Upregulated |
| ARMC3 | 101.7522936 | 127.6331361 | 0.326942 | 0.010667 | 0.010667 | Upregulated |
| CACNB3 | 174.587156 | 219.0414201 | 0.327256 | 0.000106 | 0.000106 | Upregulated |
| PCED1B | 174.5366972 | 219.1065089 | 0.328102 | 0.000101 | 0.000101 | Upregulated |
| C5orf52 | 168.2981651 | 211.2899408 | 0.328205 | 0.000178 | 0.000178 | Upregulated |
| DOK7 | 174.5 | 219.1538462 | 0.328717 | 9.84E-05 | 9.84E-05 | Upregulated |
| AQP2 | 61.56422018 | 77.34911243 | 0.329293 | 0.012083 | 0.012083 | Upregulated |
| EHHADH | 174.4311927 | 219.2426036 | 0.32987 | 9.30E-05 | 9.30E-05 | Upregulated |
| IGFL1 | 92.94954128 | 116.8343195 | 0.329944 | 0.01594 | 0.01594 | Upregulated |
| SPRY2 | 174.3944954 | 219.2899408 | 0.330485 | 9.02E-05 | 9.02E-05 | Upregulated |
| KRTAP3-2 | 35.05504587 | 44.0887574 | 0.330789 | 0.036286 | 0.036286 | Upregulated |
| RPL12 | 174.3715596 | 219.3195266 | 0.33087 | 8.85E-05 | 8.85E-05 | Upregulated |
| SMLR1 | 112.8394495 | 141.9704142 | 0.331319 | 0.003108 | 0.003108 | Upregulated |
| SARS2 | 174.3119266 | 219.3964497 | 0.331869 | 8.42E-05 | 8.42E-05 | Upregulated |
| P2RY11 | 174.2844037 | 219.4319527 | 0.33233 | 8.23E-05 | 8.23E-05 | Upregulated |
| RIMBP2 | 171.5963303 | 216.0650888 | 0.332447 | 0.000105 | 0.000105 | Upregulated |
| IL17RD | 174.266055 | 219.4556213 | 0.332638 | 8.11E-05 | 8.11E-05 | Upregulated |
| NRAP | 116.4174312 | 146.6153846 | 0.332729 | 0.007165 | 0.007165 | Upregulated |
| LPA | 72.53669725 | 91.42011834 | 0.333801 | 0.008344 | 0.008344 | Upregulated |
| POU2F3 | 174.1559633 | 219.5976331 | 0.334483 | 7.40E-05 | 7.40E-05 | Upregulated |
| DTX1 | 174.0642202 | 219.7159763 | 0.33602 | 6.85E-05 | 6.85E-05 | Upregulated |
| ALCAM | 174.0642202 | 219.7159763 | 0.33602 | 6.85E-05 | 6.85E-05 | Upregulated |
| HNRNPA0 | 173.9862385 | 219.816568 | 0.337327 | 6.41E-05 | 6.41E-05 | Upregulated |
| TMEM61 | 170.3348624 | 215.4260355 | 0.338819 | 8.27E-05 | 8.27E-05 | Upregulated |
| AC004233.2 | 126.6284404 | 160.1597633 | 0.33891 | 0.00197 | 0.00197 | Upregulated |
| PLXNB3 | 171.087156 | 216.7218935 | 0.341113 | 6.82E-05 | 6.82E-05 | Upregulated |
| TAS2R38 | 153.7844037 | 194.8402367 | 0.341382 | 0.000285 | 0.000285 | Upregulated |
| USH1C | 173.5779817 | 220.3431953 | 0.344168 | 4.53E-05 | 4.53E-05 | Upregulated |
| CSMD3 | 63.89908257 | 81.17159763 | 0.34518 | 0.037438 | 0.037438 | Upregulated |
| GPR55 | 169.940367 | 215.9349112 | 0.345568 | 5.98E-05 | 5.98E-05 | Upregulated |
| CRACR2A | 173.4541284 | 220.5029586 | 0.346244 | 4.07E-05 | 4.07E-05 | Upregulated |
| NEDD8-MDP1 | 70.50458716 | 89.65680473 | 0.346696 | 0.010028 | 0.010028 | Upregulated |
| GP9 | 78.63761468 | 100.0828402 | 0.347903 | 0.009057 | 0.009057 | Upregulated |
| MAT1A | 170.6880734 | 217.2366864 | 0.347906 | 4.95E-05 | 4.95E-05 | Upregulated |
| GDF2 | 1.073394495 | 1.366863905 | 0.348689 | 0.025819 | 0.025819 | Upregulated |
| RIPPLY1 | 128.4908257 | 163.6331361 | 0.3488 | 0.000909 | 0.000909 | Upregulated |
| SLC26A9 | 124.6513761 | 158.8224852 | 0.349516 | 0.001613 | 0.001613 | Upregulated |
| MYRF | 173.2477064 | 220.7692308 | 0.349703 | 3.40E-05 | 3.40E-05 | Upregulated |
| CRYM | 173.2247706 | 220.7988166 | 0.350087 | 3.34E-05 | 3.34E-05 | Upregulated |
| SNRNP35 | 173.2018349 | 220.8284024 | 0.350472 | 3.27E-05 | 3.27E-05 | Upregulated |
| THEG | 39.29816514 | 50.13609467 | 0.351388 | 0.028449 | 0.028449 | Upregulated |
| NLRP9 | 81.54587156 | 104.0828402 | 0.352048 | 0.009154 | 0.009154 | Upregulated |
| EPHB4 | 173.0642202 | 221.0059172 | 0.352778 | 2.90E-05 | 2.90E-05 | Upregulated |
| CCDC192 | 170.3899083 | 217.6213018 | 0.35298 | 3.81E-05 | 3.81E-05 | Upregulated |
| C9 | 92.16972477 | 117.8402367 | 0.354467 | 0.00226 | 0.00226 | Upregulated |
| L1TD1 | 170.293578 | 217.7455621 | 0.354619 | 3.48E-05 | 3.48E-05 | Upregulated |
| DUSP4 | 172.9036697 | 221.2130178 | 0.355468 | 2.52E-05 | 2.52E-05 | Upregulated |
| OXGR1 | 169.2706422 | 216.7988166 | 0.357025 | 3.42E-05 | 3.42E-05 | Upregulated |
| TBC1D3L | 68.50917431 | 87.89940828 | 0.359556 | 0.049742 | 0.049742 | Upregulated |
| PPAN-P2RY11 | 28.69266055 | 36.81656805 | 0.359673 | 0.042197 | 0.042197 | Upregulated |
| KRTAP5-8 | 52.30733945 | 67.15976331 | 0.360584 | 0.043129 | 0.043129 | Upregulated |
| ATP1A1 | 172.4908257 | 221.7455621 | 0.362386 | 1.74E-05 | 1.74E-05 | Upregulated |
| CPSF4L | 143.6100917 | 184.6627219 | 0.362736 | 0.00022 | 0.00022 | Upregulated |
| LEMD1 | 170.6972477 | 219.4970414 | 0.362762 | 2.07E-05 | 2.07E-05 | Upregulated |
| CBLC | 172.440367 | 221.8106509 | 0.363231 | 1.66E-05 | 1.66E-05 | Upregulated |
| IFITM5 | 64.02752294 | 82.39053254 | 0.363786 | 0.026769 | 0.026769 | Upregulated |
| SEC14L4 | 110.0688073 | 141.8816568 | 0.366282 | 0.002248 | 0.002248 | Upregulated |
| TRAF3IP2 | 172.206422 | 222.112426 | 0.367151 | 1.35E-05 | 1.35E-05 | Upregulated |
| AGBL4 | 120.8990826 | 155.9585799 | 0.36736 | 0.000524 | 0.000524 | Upregulated |
| C10orf95 | 172.0963303 | 222.2544379 | 0.368996 | 1.22E-05 | 1.22E-05 | Upregulated |
| GJC2 | 172.0963303 | 222.2544379 | 0.368996 | 1.22E-05 | 1.22E-05 | Upregulated |
| KLK11 | 172.0412844 | 222.3254438 | 0.369918 | 1.16E-05 | 1.16E-05 | Upregulated |
| TEX45 | 172.0412844 | 222.3254438 | 0.369918 | 1.16E-05 | 1.16E-05 | Upregulated |
| OFCC1 | 58.56422018 | 75.85798817 | 0.373282 | 0.015086 | 0.015086 | Upregulated |
| GALNT8 | 171.7844037 | 222.6568047 | 0.374223 | 9.13E-06 | 9.13E-06 | Upregulated |
| OPRD1 | 157.9220183 | 204.704142 | 0.374328 | 4.04E-05 | 4.04E-05 | Upregulated |
| NUTM2F | 50.13302752 | 65.00591716 | 0.37481 | 0.022349 | 0.022349 | Upregulated |
| KCNN4 | 171.5825688 | 222.9171598 | 0.377605 | 7.56E-06 | 7.56E-06 | Upregulated |
| C11orf53 | 168.0321101 | 218.3964497 | 0.378212 | 1.10E-05 | 1.10E-05 | Upregulated |
| C19orf57 | 171.5137615 | 223.0059172 | 0.378758 | 7.09E-06 | 7.09E-06 | Upregulated |
| KRTAP10-2 | 16.36697248 | 21.33136095 | 0.382189 | 0.015606 | 0.015606 | Upregulated |
| C2orf66 | 120.2247706 | 156.8284024 | 0.383453 | 0.001021 | 0.001021 | Upregulated |
| FEZF1 | 129.9770642 | 169.6331361 | 0.384161 | 0.000284 | 0.000284 | Upregulated |
| RASAL1 | 171.146789 | 223.4792899 | 0.384907 | 5.01E-06 | 5.01E-06 | Upregulated |
| TRIM58 | 152.2981651 | 198.9112426 | 0.385226 | 4.78E-05 | 4.78E-05 | Upregulated |
| GAST | 57.29357798 | 74.85207101 | 0.385669 | 0.021317 | 0.021317 | Upregulated |
| NXF5 | 7.376146789 | 9.639053254 | 0.386024 | 0.046736 | 0.046736 | Upregulated |
| KLK15 | 163.1651376 | 213.4319527 | 0.387443 | 1.14E-05 | 1.14E-05 | Upregulated |
| NBPF6 | 70.61926606 | 92.4260355 | 0.388237 | 0.006142 | 0.006142 | Upregulated |
| CCDC155 | 42.17889908 | 55.24852071 | 0.389414 | 0.022263 | 0.022263 | Upregulated |
| AC245041.1 | 110.587156 | 144.8757396 | 0.389632 | 0.000954 | 0.000954 | Upregulated |
| OR51M1 | 1.119266055 | 1.467455621 | 0.390764 | 0.032372 | 0.032372 | Upregulated |
| ZNF560 | 27.32568807 | 35.84615385 | 0.391561 | 0.032704 | 0.032704 | Upregulated |
| MYEOV | 170.7477064 | 223.9940828 | 0.391594 | 3.42E-06 | 3.42E-06 | Upregulated |
| LCN1 | 67.13761468 | 88.23668639 | 0.394257 | 0.008642 | 0.008642 | Upregulated |
| ACADL | 42.51376147 | 55.94674556 | 0.396124 | 0.007977 | 0.007977 | Upregulated |
| LMOD3 | 153.2798165 | 201.9704142 | 0.397976 | 2.15E-05 | 2.15E-05 | Upregulated |
| AHSG | 97.92201835 | 129.1005917 | 0.39879 | 0.000758 | 0.000758 | Upregulated |
| PXN | 170.2706422 | 224.6094675 | 0.399589 | 2.15E-06 | 2.15E-06 | Upregulated |
| KCNV1 | 123.9816514 | 163.5739645 | 0.399817 | 0.000173 | 0.000173 | Upregulated |
| SBSPON | 170.2477064 | 224.6390533 | 0.399973 | 2.10E-06 | 2.10E-06 | Upregulated |
| RLBP1 | 98.52293578 | 130.0591716 | 0.400637 | 0.004262 | 0.004262 | Upregulated |
| MYL2 | 39.85779817 | 52.68047337 | 0.402406 | 0.015469 | 0.015469 | Upregulated |
| SPINK7 | 35.72477064 | 47.33136095 | 0.405872 | 0.019734 | 0.019734 | Upregulated |
| ERICH2 | 158.6926606 | 210.3136095 | 0.406307 | 5.99E-06 | 5.99E-06 | Upregulated |
| HES1 | 169.733945 | 225.3017751 | 0.408584 | 1.26E-06 | 1.26E-06 | Upregulated |
| ATP6V0A4 | 100.2247706 | 133.1005917 | 0.409278 | 0.000469 | 0.000469 | Upregulated |
| CACNA1C | 169.6834862 | 225.3668639 | 0.409429 | 1.20E-06 | 1.20E-06 | Upregulated |
| ARHGEF28 | 169.5963303 | 225.4792899 | 0.41089 | 1.09E-06 | 1.09E-06 | Upregulated |
| TRPM5 | 168.6055046 | 224.4733728 | 0.412893 | 1.10E-06 | 1.10E-06 | Upregulated |
| SH3TC2 | 169.4495413 | 225.6686391 | 0.41335 | 9.43E-07 | 9.43E-07 | Upregulated |
| DMRTA2 | 125.6330275 | 167.3195266 | 0.41339 | 0.000136 | 0.000136 | Upregulated |
| KRT18 | 169.3761468 | 225.7633136 | 0.41458 | 8.75E-07 | 8.75E-07 | Upregulated |
| NBPF4 | 104.0779817 | 138.7633136 | 0.414961 | 0.000689 | 0.000689 | Upregulated |
| EN2 | 113.8394495 | 151.8106509 | 0.415272 | 0.000708 | 0.000708 | Upregulated |
| RNASE13 | 43.09633028 | 57.47337278 | 0.415329 | 0.018546 | 0.018546 | Upregulated |
| HOXB4 | 169.1972477 | 225.9940828 | 0.417579 | 7.29E-07 | 7.29E-07 | Upregulated |
| TMEM211 | 162.6743119 | 217.3254438 | 0.417871 | 1.67E-06 | 1.67E-06 | Upregulated |
| KLK4 | 140.8073394 | 188.2781065 | 0.419143 | 2.09E-05 | 2.09E-05 | Upregulated |
| EDAR | 169.0963303 | 226.1242604 | 0.41927 | 6.57E-07 | 6.57E-07 | Upregulated |
| COLCA2 | 169 | 226.2485207 | 0.420885 | 5.95E-07 | 5.95E-07 | Upregulated |
| SLC18A1 | 159.5137615 | 213.6863905 | 0.421814 | 2.23E-06 | 2.23E-06 | Upregulated |
| KLK12 | 159.4311927 | 213.7928994 | 0.42328 | 2.11E-06 | 2.11E-06 | Upregulated |
| RNF10 | 168.8302752 | 226.4674556 | 0.42373 | 4.99E-07 | 4.99E-07 | Upregulated |
| SMYD1 | 97.05504587 | 130.2189349 | 0.424064 | 0.000755 | 0.000755 | Upregulated |
| SH2D6 | 165.3394495 | 221.8698225 | 0.424282 | 8.12E-07 | 8.12E-07 | Upregulated |
| ERP29 | 168.733945 | 226.591716 | 0.425345 | 4.51E-07 | 4.51E-07 | Upregulated |
| GNG13 | 123.5045872 | 166.1420118 | 0.427852 | 0.000118 | 0.000118 | Upregulated |
| FABP7 | 44.65137615 | 60.09467456 | 0.428533 | 0.037806 | 0.037806 | Upregulated |
| MTRNR2L11 | 18.83027523 | 25.34319527 | 0.428544 | 0.034949 | 0.034949 | Upregulated |
| PPIAL4C | 28.70183486 | 38.65680473 | 0.429579 | 0.019993 | 0.019993 | Upregulated |
| NOL4 | 100.1697248 | 134.9289941 | 0.429754 | 0.000323 | 0.000323 | Upregulated |
| TBX20 | 70.14220183 | 94.50887574 | 0.430167 | 0.007486 | 0.007486 | Upregulated |
| VASP | 168.3807339 | 227.0473373 | 0.431266 | 3.11E-07 | 3.11E-07 | Upregulated |
| 11-Mar | 11.96330275 | 16.14792899 | 0.432733 | 0.031524 | 0.031524 | Upregulated |
| TMPRSS11E | 58.31192661 | 78.85207101 | 0.435358 | 0.008203 | 0.008203 | Upregulated |
| CRB2 | 168.0917431 | 227.4201183 | 0.436111 | 2.29E-07 | 2.29E-07 | Upregulated |
| C8orf74 | 90.19724771 | 122.0532544 | 0.436355 | 0.000615 | 0.000615 | Upregulated |
| MROH7-TTC4 | 81.52752294 | 110.4142012 | 0.437567 | 0.001587 | 0.001587 | Upregulated |
| DKK4 | 155.2798165 | 210.3076923 | 0.437631 | 1.39E-06 | 1.39E-06 | Upregulated |
| TMC4 | 167.9266055 | 227.6331361 | 0.43888 | 1.92E-07 | 1.92E-07 | Upregulated |
| POU3F3 | 81.48165138 | 110.4733728 | 0.439152 | 0.001537 | 0.001537 | Upregulated |
| HCN1 | 132.7706422 | 180.112426 | 0.439962 | 3.24E-05 | 3.24E-05 | Upregulated |
| ALDH3B2 | 158.4862385 | 215.0118343 | 0.440058 | 7.96E-07 | 7.96E-07 | Upregulated |
| AGMO | 159.3165138 | 216.1656805 | 0.440242 | 6.98E-07 | 6.98E-07 | Upregulated |
| GPRC5D | 167.8302752 | 227.7573964 | 0.440495 | 1.73E-07 | 1.73E-07 | Upregulated |
| RP1 | 99.06422018 | 134.5976331 | 0.442217 | 0.000913 | 0.000913 | Upregulated |
| RAET1L | 165.9082569 | 225.6745562 | 0.443858 | 1.85E-07 | 1.85E-07 | Upregulated |
| NPB | 34.44036697 | 46.92307692 | 0.446197 | 0.015526 | 0.015526 | Upregulated |
| KLK5 | 40.06880734 | 54.61538462 | 0.446828 | 0.022425 | 0.022425 | Upregulated |
| ANKRD33 | 77.69724771 | 105.9289941 | 0.447162 | 0.00123 | 0.00123 | Upregulated |
| IL37 | 138.6192661 | 189.0177515 | 0.447394 | 7.71E-06 | 7.71E-06 | Upregulated |
| TRIM39-RPP21 | 20.7293578 | 28.27810651 | 0.44801 | 0.045759 | 0.045759 | Upregulated |
| SIX3 | 64.25229358 | 87.69822485 | 0.4488 | 0.003197 | 0.003197 | Upregulated |
| AQP6 | 154.5458716 | 211.2544379 | 0.450947 | 6.35E-07 | 6.35E-07 | Upregulated |
| KRT33A | 1.28440367 | 1.75739645 | 0.452341 | 0.026363 | 0.026363 | Upregulated |
| CPB1 | 45.59633028 | 62.40828402 | 0.45282 | 0.015082 | 0.015082 | Upregulated |
| SPAG6 | 39.10550459 | 53.65088757 | 0.45623 | 0.017097 | 0.017097 | Upregulated |
| NLRP10 | 1.325688073 | 1.822485207 | 0.459166 | 0.048113 | 0.048113 | Upregulated |
| FGF20 | 78.51834862 | 107.9881657 | 0.459771 | 0.000696 | 0.000696 | Upregulated |
| RFX6 | 85.69266055 | 117.9408284 | 0.46082 | 0.000601 | 0.000601 | Upregulated |
| ANKRD30B | 68.76605505 | 94.81656805 | 0.463443 | 0.0028 | 0.0028 | Upregulated |
| PLA2G4D | 162.1697248 | 223.6982249 | 0.464049 | 7.87E-08 | 7.87E-08 | Upregulated |
| USP41 | 95.67889908 | 131.9940828 | 0.464201 | 0.000267 | 0.000267 | Upregulated |
| LIPK | 14.63302752 | 20.19526627 | 0.464789 | 0.023298 | 0.023298 | Upregulated |
| CCDC60 | 133.8440367 | 184.852071 | 0.465818 | 7.90E-06 | 7.90E-06 | Upregulated |
| SLC9A4 | 98.11009174 | 135.8284024 | 0.469312 | 0.000239 | 0.000239 | Upregulated |
| C4orf22 | 9.133027523 | 12.64497041 | 0.469399 | 0.043879 | 0.043879 | Upregulated |
| CAPN12 | 165.9862385 | 230.1360947 | 0.471424 | 2.21E-08 | 2.21E-08 | Upregulated |
| C9orf57 | 50.28440367 | 69.76923077 | 0.47248 | 0.003154 | 0.003154 | Upregulated |
| CDH7 | 80.49082569 | 111.7514793 | 0.473398 | 0.000756 | 0.000756 | Upregulated |
| FGF21 | 25.19724771 | 35.0295858 | 0.475308 | 0.042966 | 0.042966 | Upregulated |
| FAIM2 | 165.6284404 | 230.5976331 | 0.477427 | 1.46E-08 | 1.46E-08 | Upregulated |
| CALHM1 | 66.78440367 | 93.00591716 | 0.477811 | 0.005486 | 0.005486 | Upregulated |
| FREM3 | 40.82110092 | 57 | 0.481647 | 0.013117 | 0.013117 | Upregulated |
| OR1C1 | 2.23853211 | 3.130177515 | 0.483691 | 0.020021 | 0.020021 | Upregulated |
| KLK14 | 151.559633 | 212.9112426 | 0.490367 | 7.30E-08 | 7.30E-08 | Upregulated |
| PAGE5 | 37.74311927 | 53.22485207 | 0.495886 | 0.013273 | 0.013273 | Upregulated |
| FAM71E2 | 3.532110092 | 4.982248521 | 0.496267 | 0.047439 | 0.047439 | Upregulated |
| DSG4 | 160.2018349 | 226.2366864 | 0.497942 | 8.15E-09 | 8.15E-09 | Upregulated |
| C14orf105 | 82.11009174 | 116.0650888 | 0.499303 | 0.000398 | 0.000398 | Upregulated |
| SPRR2A | 72.14220183 | 102.3668639 | 0.504833 | 7.69E-05 | 7.69E-05 | Upregulated |
| SPRR2G | 2.380733945 | 3.378698225 | 0.505061 | 0.045429 | 0.045429 | Upregulated |
| CALHM3 | 83.10550459 | 118.0177515 | 0.505988 | 0.00053 | 0.00053 | Upregulated |
| OR10H1 | 14.41284404 | 20.47928994 | 0.506811 | 0.044646 | 0.044646 | Upregulated |
| PLG | 18.55045872 | 26.4556213 | 0.512119 | 0.020141 | 0.020141 | Upregulated |
| C1orf141 | 24.86238532 | 35.46153846 | 0.51229 | 0.035682 | 0.035682 | Upregulated |
| KRTAP3-1 | 38.31192661 | 54.67455621 | 0.513076 | 0.003143 | 0.003143 | Upregulated |
| TMPRSS11D | 19.62385321 | 28.13609467 | 0.519814 | 0.011915 | 0.011915 | Upregulated |
| PRB2 | 32.41743119 | 46.47928994 | 0.519818 | 0.008455 | 0.008455 | Upregulated |
| OR13G1 | 1.78440367 | 2.562130178 | 0.521902 | 0.015624 | 0.015624 | Upregulated |
| PAX2 | 73.88990826 | 106.2071006 | 0.523431 | 0.000174 | 0.000174 | Upregulated |
| CIB4 | 55.48623853 | 79.82840237 | 0.524772 | 0.000307 | 0.000307 | Upregulated |
| KRT6C | 56.93119266 | 81.97633136 | 0.525988 | 0.000223 | 0.000223 | Upregulated |
| SUN5 | 1.293577982 | 1.863905325 | 0.526962 | 0.046251 | 0.046251 | Upregulated |
| TMEM189-UBE2V1 | 33.0733945 | 47.66272189 | 0.52719 | 0.010161 | 0.010161 | Upregulated |
| GSX1 | 15.02752294 | 21.69230769 | 0.529576 | 0.002106 | 0.002106 | Upregulated |
| OCM2 | 1.096330275 | 1.585798817 | 0.532527 | 0.00378 | 0.00378 | Upregulated |
| RGR | 64.80275229 | 94.1183432 | 0.538421 | 0.002014 | 0.002014 | Upregulated |
| PTPN13 | 161.8165138 | 235.5147929 | 0.541459 | 1.29E-10 | 1.29E-10 | Upregulated |
| KRT6A | 139.2155963 | 202.9526627 | 0.543822 | 1.42E-08 | 1.42E-08 | Upregulated |
| IVL | 18.60091743 | 27.14792899 | 0.545468 | 0.013947 | 0.013947 | Upregulated |
| KLK3 | 137.4633028 | 200.9822485 | 0.548022 | 1.41E-08 | 1.41E-08 | Upregulated |
| POTEC | 13.24311927 | 19.3964497 | 0.55055 | 0.012124 | 0.012124 | Upregulated |
| OR2W3 | 84.6146789 | 124.2662722 | 0.554455 | 7.80E-05 | 7.80E-05 | Upregulated |
| CLEC3A | 19.36697248 | 28.46745562 | 0.555715 | 0.030791 | 0.030791 | Upregulated |
| PAX7 | 20.49082569 | 30.17751479 | 0.558496 | 0.01294 | 0.01294 | Upregulated |
| KLK2 | 84.43119266 | 124.5029586 | 0.560332 | 1.83E-05 | 1.83E-05 | Upregulated |
| OR52B2 | 2.582568807 | 3.810650888 | 0.561231 | 0.030644 | 0.030644 | Upregulated |
| SPRR2D | 82.57798165 | 121.9585799 | 0.562562 | 4.89E-05 | 4.89E-05 | Upregulated |
| AMELY | 2.848623853 | 4.213017751 | 0.564589 | 0.044503 | 0.044503 | Upregulated |
| PRTN3 | 13.87155963 | 20.52071006 | 0.564951 | 0.046205 | 0.046205 | Upregulated |
| TBC1D3D | 5.536697248 | 8.230769231 | 0.572002 | 0.049538 | 0.049538 | Upregulated |
| KLK13 | 109.4770642 | 163.0828402 | 0.574976 | 3.80E-07 | 3.80E-07 | Upregulated |
| KRTAP1-5 | 18.39908257 | 27.40828402 | 0.574978 | 0.037246 | 0.037246 | Upregulated |
| FRMPD2 | 45.25688073 | 67.63905325 | 0.579719 | 0.000526 | 0.000526 | Upregulated |
| FAM170B | 6.091743119 | 9.136094675 | 0.584722 | 0.045717 | 0.045717 | Upregulated |
| DKK1 | 135.5642202 | 203.4319527 | 0.58557 | 1.21E-09 | 1.21E-09 | Upregulated |
| ARL14EPL | 6.229357798 | 9.378698225 | 0.590304 | 0.010986 | 0.010986 | Upregulated |
| XAGE3 | 24.1559633 | 36.37278107 | 0.59048 | 0.003909 | 0.003909 | Upregulated |
| CCDC70 | 22.29816514 | 33.6035503 | 0.591689 | 0.021214 | 0.021214 | Upregulated |
| SPRR1B | 63.96330275 | 96.64497041 | 0.59545 | 0.000132 | 0.000132 | Upregulated |
| LYZL4 | 33.24311927 | 50.53254438 | 0.604157 | 0.0002 | 0.0002 | Upregulated |
| PAX3 | 26.21100917 | 40.01775148 | 0.610467 | 0.034576 | 0.034576 | Upregulated |
| SPRR1A | 60.55045872 | 92.47337278 | 0.6109 | 0.000208 | 0.000208 | Upregulated |
| CSN2 | 5.311926606 | 8.130177515 | 0.614052 | 0.005535 | 0.005535 | Upregulated |
| OTP | 29.88073394 | 45.76331361 | 0.614976 | 0.006743 | 0.006743 | Upregulated |
| CCDC103 | 31.65137615 | 48.47928994 | 0.6151 | 0.001541 | 0.001541 | Upregulated |
| DMRTB1 | 2.206422018 | 3.384615385 | 0.617283 | 0.011762 | 0.011762 | Upregulated |
| OR13J1 | 32.26146789 | 49.73372781 | 0.624412 | 0.007292 | 0.007292 | Upregulated |
| HTR3E | 72.14678899 | 111.5384615 | 0.628534 | 4.50E-05 | 4.50E-05 | Upregulated |
| AL590560.1 | 26.94954128 | 41.85207101 | 0.635038 | 0.001285 | 0.001285 | Upregulated |
| BPIFB2 | 22.49541284 | 35.04733728 | 0.639674 | 0.007311 | 0.007311 | Upregulated |
| SPRR3 | 55.38073394 | 86.68047337 | 0.646323 | 5.83E-05 | 5.83E-05 | Upregulated |
| VGLL1 | 48.96788991 | 77.79881657 | 0.667912 | 0.000181 | 0.000181 | Upregulated |
| TMEM210 | 48.86697248 | 77.92899408 | 0.6733 | 0.00012 | 0.00012 | Upregulated |
| OR52E8 | 1.357798165 | 2.171597633 | 0.677488 | 0.009618 | 0.009618 | Upregulated |
| GPRC6A | 49.09174312 | 78.92307692 | 0.684967 | 1.90E-05 | 1.90E-05 | Upregulated |
| CGB5 | 18.97247706 | 30.5443787 | 0.686999 | 0.007239 | 0.007239 | Upregulated |
| CALML5 | 19.46788991 | 31.49704142 | 0.69412 | 0.006419 | 0.006419 | Upregulated |
| CRCT1 | 6.270642202 | 10.18343195 | 0.699539 | 0.00113 | 0.00113 | Upregulated |
| SP9 | 19.54587156 | 32.20118343 | 0.72025 | 0.002813 | 0.002813 | Upregulated |
| KRT34 | 24.81651376 | 40.89940828 | 0.72078 | 0.002714 | 0.002714 | Upregulated |
| OR8G5 | 2.591743119 | 4.289940828 | 0.727035 | 0.01211 | 0.01211 | Upregulated |
| CGB8 | 7.908256881 | 13.21301775 | 0.740528 | 0.015158 | 0.015158 | Upregulated |
| MMP26 | 5.591743119 | 9.366863905 | 0.744268 | 0.027429 | 0.027429 | Upregulated |
| CA6 | 36.80275229 | 62.18343195 | 0.756717 | 3.91E-05 | 3.91E-05 | Upregulated |
| RPTN | 8.720183486 | 14.73964497 | 0.757271 | 0.00982 | 0.00982 | Upregulated |
| OR14K1 | 2.013761468 | 3.420118343 | 0.764153 | 0.015684 | 0.015684 | Upregulated |
| KHDC3L | 1.458715596 | 2.485207101 | 0.768667 | 0.006313 | 0.006313 | Upregulated |
| FAM25A | 15.08256881 | 25.79289941 | 0.774092 | 0.000933 | 0.000933 | Upregulated |
| SERPINA11 | 45.13761468 | 77.66272189 | 0.782892 | 4.17E-06 | 4.17E-06 | Upregulated |
| IFNL3 | 8.201834862 | 14.36094675 | 0.808132 | 0.015409 | 0.015409 | Upregulated |
| DNTT | 6.444954128 | 11.28994083 | 0.808796 | 0.045137 | 0.045137 | Upregulated |
| FOXD4L6 | 7.697247706 | 13.4852071 | 0.808963 | 0.012881 | 0.012881 | Upregulated |
| KRT33B | 3.119266055 | 5.514792899 | 0.8221 | 0.007825 | 0.007825 | Upregulated |
| DPPA2 | 2.165137615 | 3.881656805 | 0.842214 | 0.003921 | 0.003921 | Upregulated |
| KRTAP16-1 | 4.004587156 | 7.24852071 | 0.856033 | 0.038511 | 0.038511 | Upregulated |
| OTOS | 2.376146789 | 4.319526627 | 0.862249 | 0.015698 | 0.015698 | Upregulated |
| KRT74 | 16.91743119 | 30.85207101 | 0.866857 | 0.000605 | 0.000605 | Upregulated |
| FOXI1 | 61.2293578 | 111.9349112 | 0.870365 | 2.54E-09 | 2.54E-09 | Upregulated |
| OR2T8 | 9.47706422 | 17.61538462 | 0.894324 | 0.000175 | 0.000175 | Upregulated |
| CCDC140 | 2.394495413 | 4.544378698 | 0.924361 | 0.004056 | 0.004056 | Upregulated |
| OR2T33 | 2.013761468 | 3.852071006 | 0.935741 | 0.002039 | 0.002039 | Upregulated |
| INS | 2.69266055 | 5.49112426 | 1.028069 | 0.013298 | 0.013298 | Upregulated |
| SPP2 | 2.440366972 | 5 | 1.03483 | 0.00886 | 0.00886 | Upregulated |
| ELSPBP1 | 4.95412844 | 10.18934911 | 1.040359 | 0.001088 | 0.001088 | Upregulated |
| VCX2 | 2.041284404 | 4.272189349 | 1.065498 | 0.000661 | 0.000661 | Upregulated |
| RGS21 | 7.133027523 | 15.22485207 | 1.093842 | 0.000901 | 0.000901 | Upregulated |
| OTX2 | 4.811926606 | 10.37278107 | 1.108116 | 7.42E-05 | 7.42E-05 | Upregulated |
| KRTAP1-1 | 9.417431193 | 20.62130178 | 1.13073 | 0.000572 | 0.000572 | Upregulated |
| HTR3C | 25.05963303 | 58 | 1.210688 | 1.01E-08 | 1.01E-08 | Upregulated |
| GNAT3 | 6.52293578 | 16.01183432 | 1.295545 | 0.000237 | 0.000237 | Upregulated |
| C1QL2 | 3.128440367 | 7.698224852 | 1.299082 | 0.001128 | 0.001128 | Upregulated |
| TAS1R2 | 1.637614679 | 4.562130178 | 1.478112 | 0.000681 | 0.000681 | Upregulated |

| Gene | wildMeans | mutationMeans | logFC | p_value | FDR | Type |
| --- | --- | --- | --- | --- | --- | --- |
| LECT2 | 7.151376147 | 3.189349112 | -1.16496 | 0.019856 | 0.019856 | Downregulated |
| AC187653.1 | 11.03211009 | 5.372781065 | -1.03797 | 0.005153 | 0.005153 | Downregulated |
| PTF1A | 49.53211009 | 24.40236686 | -1.02134 | 0.000414 | 0.000414 | Downregulated |
| VSTM2B | 30.56880734 | 15.58579882 | -0.97183 | 0.002222 | 0.002222 | Downregulated |
| HIST2H4B | 6.665137615 | 3.473372781 | -0.9403 | 0.008667 | 0.008667 | Downregulated |
| ACCSL | 5.931192661 | 3.106508876 | -0.93303 | 0.047702 | 0.047702 | Downregulated |
| OR1E1 | 2.766055046 | 1.473372781 | -0.90871 | 0.016941 | 0.016941 | Downregulated |
| DNMT3L | 17.87614679 | 9.710059172 | -0.88048 | 0.002993 | 0.002993 | Downregulated |
| AC091980.2 | 6.197247706 | 3.408284024 | -0.86258 | 0.031345 | 0.031345 | Downregulated |
| SLC35G3 | 4.568807339 | 2.520710059 | -0.85799 | 0.005996 | 0.005996 | Downregulated |
| AL365273.2 | 3.871559633 | 2.147928994 | -0.84997 | 0.026142 | 0.026142 | Downregulated |
| C3orf84 | 2.633027523 | 1.467455621 | -0.84341 | 0.009074 | 0.009074 | Downregulated |
| MAGEA9B | 5.123853211 | 2.928994083 | -0.80682 | 0.041326 | 0.041326 | Downregulated |
| ANKRD62 | 29.94036697 | 17.18934911 | -0.80058 | 0.009217 | 0.009217 | Downregulated |
| KRT27 | 15.96788991 | 9.24260355 | -0.7888 | 0.00721 | 0.00721 | Downregulated |
| MYH2 | 28.16513761 | 16.34319527 | -0.78522 | 0.030018 | 0.030018 | Downregulated |
| GOLGA8G | 5.743119266 | 3.349112426 | -0.77806 | 0.029824 | 0.029824 | Downregulated |
| KRTAP13-2 | 14.12385321 | 8.301775148 | -0.76664 | 0.027371 | 0.027371 | Downregulated |
| OR9A2 | 2.385321101 | 1.449704142 | -0.71842 | 0.049092 | 0.049092 | Downregulated |
| AKAP4 | 92.67431193 | 56.72781065 | -0.70811 | 0.00014 | 0.00014 | Downregulated |
| PAPOLB | 15.08715596 | 9.24852071 | -0.70603 | 0.00827 | 0.00827 | Downregulated |
| C17orf105 | 32.26605505 | 19.90532544 | -0.69686 | 0.025339 | 0.025339 | Downregulated |
| ACOD1 | 67.28440367 | 41.65680473 | -0.69172 | 0.001698 | 0.001698 | Downregulated |
| CCDC177 | 37.10550459 | 23.23076923 | -0.6756 | 0.023153 | 0.023153 | Downregulated |
| HRH3 | 26.68807339 | 16.71597633 | -0.67497 | 0.009939 | 0.009939 | Downregulated |
| MLNR | 28.5733945 | 18.15976331 | -0.65393 | 0.020636 | 0.020636 | Downregulated |
| PLET1 | 16.67889908 | 10.65680473 | -0.64625 | 0.019893 | 0.019893 | Downregulated |
| NOTO | 14.86697248 | 9.532544379 | -0.64118 | 0.040462 | 0.040462 | Downregulated |
| MYH8 | 30.90825688 | 19.99408284 | -0.62842 | 0.024111 | 0.024111 | Downregulated |
| AMER2 | 31.74770642 | 20.5739645 | -0.62583 | 0.047045 | 0.047045 | Downregulated |
| DIO1 | 154.0458716 | 99.94674556 | -0.62413 | 4.32E-07 | 4.32E-07 | Downregulated |
| EPHA8 | 70.00458716 | 45.58579882 | -0.61887 | 0.001355 | 0.001355 | Downregulated |
| AL049844.1 | 23.67431193 | 15.4260355 | -0.61796 | 0.01988 | 0.01988 | Downregulated |
| GPR52 | 28.27522936 | 18.5443787 | -0.60856 | 0.035082 | 0.035082 | Downregulated |
| BTNL2 | 13.89908257 | 9.130177515 | -0.60627 | 0.049302 | 0.049302 | Downregulated |
| PSAPL1 | 50.00917431 | 33.10650888 | -0.59508 | 0.032614 | 0.032614 | Downregulated |
| KRT222 | 69.50917431 | 46.22485207 | -0.58853 | 0.001933 | 0.001933 | Downregulated |
| PAGE4 | 8.472477064 | 5.650887574 | -0.58431 | 0.018487 | 0.018487 | Downregulated |
| STARD6 | 18.92201835 | 12.71005917 | -0.5741 | 0.045579 | 0.045579 | Downregulated |
| RDH8 | 63.55963303 | 42.82248521 | -0.56974 | 0.002393 | 0.002393 | Downregulated |
| CHIA | 6.862385321 | 4.627218935 | -0.56856 | 0.010192 | 0.010192 | Downregulated |
| CENPVL3 | 1.798165138 | 1.213017751 | -0.56792 | 0.027745 | 0.027745 | Downregulated |
| IL21 | 30.88073394 | 20.85798817 | -0.56611 | 0.037369 | 0.037369 | Downregulated |
| FZD10 | 223.1788991 | 151.7988166 | -0.55604 | 4.78E-10 | 4.78E-10 | Downregulated |
| ETNPPL | 14.28899083 | 9.721893491 | -0.55559 | 0.024275 | 0.024275 | Downregulated |
| PITX3 | 42.22477064 | 28.85798817 | -0.54912 | 0.002825 | 0.002825 | Downregulated |
| LIPN | 66.68348624 | 46.12426036 | -0.5318 | 0.005722 | 0.005722 | Downregulated |
| OR2C1 | 50.87614679 | 35.20118343 | -0.53137 | 0.025176 | 0.025176 | Downregulated |
| IQCA1L | 5.752293578 | 3.982248521 | -0.53055 | 0.044826 | 0.044826 | Downregulated |
| MYH6 | 35.83486239 | 24.86982249 | -0.52697 | 0.026002 | 0.026002 | Downregulated |
| ENPP1 | 223.7522936 | 155.6213018 | -0.52386 | 2.81E-09 | 2.81E-09 | Downregulated |
| MC2R | 27.69266055 | 19.29585799 | -0.52121 | 0.048836 | 0.048836 | Downregulated |
| C11orf52 | 123.3577982 | 86.01183432 | -0.52024 | 0.000181 | 0.000181 | Downregulated |
| INSL5 | 55.72477064 | 38.90532544 | -0.51835 | 0.007703 | 0.007703 | Downregulated |
| SLC22A4 | 223.3853211 | 156.0946746 | -0.51711 | 4.39E-09 | 4.39E-09 | Downregulated |
| PATE1 | 1.903669725 | 1.331360947 | -0.51588 | 0.00412 | 0.00412 | Downregulated |
| ITPRIPL1 | 222.8669725 | 156.7633136 | -0.50759 | 8.15E-09 | 8.15E-09 | Downregulated |
| NPY2R | 35.18807339 | 24.80473373 | -0.50447 | 0.017841 | 0.017841 | Downregulated |
| LCLAT1 | 222.5733945 | 157.1420118 | -0.50221 | 1.15E-08 | 1.15E-08 | Downregulated |
| PPM1D | 222.3027523 | 157.4911243 | -0.49726 | 1.58E-08 | 1.58E-08 | Downregulated |
| HSPB3 | 102.3211009 | 72.61538462 | -0.49476 | 0.003428 | 0.003428 | Downregulated |
| OR3A3 | 1.45412844 | 1.035502959 | -0.48982 | 0.022693 | 0.022693 | Downregulated |
| OTOP3 | 16.06422018 | 11.44970414 | -0.48854 | 0.019332 | 0.019332 | Downregulated |
| MERTK | 221.8256881 | 158.1065089 | -0.48853 | 2.74E-08 | 2.74E-08 | Downregulated |
| PATE3 | 1.889908257 | 1.349112426 | -0.48631 | 0.014426 | 0.014426 | Downregulated |
| HACE1 | 221.5825688 | 158.4201183 | -0.48409 | 3.61E-08 | 3.61E-08 | Downregulated |
| FAM19A4 | 30.25229358 | 21.66863905 | -0.48144 | 0.038753 | 0.038753 | Downregulated |
| ZNF101 | 221.4266055 | 158.6213018 | -0.48124 | 4.31E-08 | 4.31E-08 | Downregulated |
| DAZL | 98.16055046 | 70.31952663 | -0.48122 | 0.001736 | 0.001736 | Downregulated |
| CLEC6A | 113.059633 | 81.00591716 | -0.48098 | 0.000459 | 0.000459 | Downregulated |
| ALX1 | 31.89908257 | 22.91715976 | -0.47709 | 0.041546 | 0.041546 | Downregulated |
| RAB39A | 190.4266055 | 136.8934911 | -0.47618 | 2.44E-06 | 2.44E-06 | Downregulated |
| MTDH | 221.0366972 | 159.1242604 | -0.47413 | 6.67E-08 | 6.67E-08 | Downregulated |
| NANP | 220.9770642 | 159.2011834 | -0.47305 | 7.13E-08 | 7.13E-08 | Downregulated |
| RNFT1 | 220.8348624 | 159.3846154 | -0.47046 | 8.35E-08 | 8.35E-08 | Downregulated |
| FADS6 | 126.8853211 | 91.73964497 | -0.46791 | 0.00075 | 0.00075 | Downregulated |
| ZNF844 | 220.3073394 | 160.0650888 | -0.46086 | 1.49E-07 | 1.49E-07 | Downregulated |
| ZNF492 | 113.3027523 | 82.32544379 | -0.46077 | 0.003029 | 0.003029 | Downregulated |
| SMIM18 | 73.04587156 | 53.21893491 | -0.45686 | 0.005325 | 0.005325 | Downregulated |
| ZNF304 | 220.0275229 | 160.4260355 | -0.45578 | 2.01E-07 | 2.01E-07 | Downregulated |
| IL20 | 58.83944954 | 42.96449704 | -0.45364 | 0.019289 | 0.019289 | Downregulated |
| RFX2 | 219.8073394 | 160.7100592 | -0.45178 | 2.55E-07 | 2.55E-07 | Downregulated |
| KLHL7 | 219.7844037 | 160.739645 | -0.45136 | 2.61E-07 | 2.61E-07 | Downregulated |
| ZDHHC19 | 144.0366972 | 105.4142012 | -0.45037 | 0.000423 | 0.000423 | Downregulated |
| LRRC14B | 54.78440367 | 40.1183432 | -0.4495 | 0.047186 | 0.047186 | Downregulated |
| ADAM22 | 219.646789 | 160.9171598 | -0.44887 | 3.02E-07 | 3.02E-07 | Downregulated |
| HRASLS | 110.5458716 | 81 | -0.44865 | 0.00115 | 0.00115 | Downregulated |
| NMT2 | 219.5137615 | 161.0887574 | -0.44646 | 3.48E-07 | 3.48E-07 | Downregulated |
| ZNF639 | 219.4541284 | 161.1656805 | -0.44537 | 3.70E-07 | 3.70E-07 | Downregulated |
| HS3ST4 | 84.2293578 | 61.92899408 | -0.44371 | 0.00863 | 0.00863 | Downregulated |
| DCUN1D4 | 219.2247706 | 161.4615385 | -0.44122 | 4.71E-07 | 4.71E-07 | Downregulated |
| TMEM236 | 219.087156 | 161.6390533 | -0.43873 | 5.43E-07 | 5.43E-07 | Downregulated |
| CDIP1 | 219.0550459 | 161.6804734 | -0.43815 | 5.62E-07 | 5.62E-07 | Downregulated |
| PCGF6 | 218.8669725 | 161.9230769 | -0.43475 | 6.82E-07 | 6.82E-07 | Downregulated |
| FAM216A | 218.8119266 | 161.9940828 | -0.43375 | 7.22E-07 | 7.22E-07 | Downregulated |
| SLC28A1 | 124.0550459 | 91.9408284 | -0.4322 | 0.001825 | 0.001825 | Downregulated |
| HIST1H2AI | 193.5504587 | 143.5443787 | -0.43121 | 1.04E-05 | 1.04E-05 | Downregulated |
| FAM180B | 55.65137615 | 41.27810651 | -0.43104 | 0.033693 | 0.033693 | Downregulated |
| KLRG2 | 154.440367 | 114.6094675 | -0.43032 | 0.00065 | 0.00065 | Downregulated |
| ACHE | 218.6146789 | 162.2485207 | -0.43018 | 8.83E-07 | 8.83E-07 | Downregulated |
| TBC1D31 | 218.5917431 | 162.2781065 | -0.42977 | 9.04E-07 | 9.04E-07 | Downregulated |
| VNN2 | 217.4678899 | 161.443787 | -0.42977 | 1.03E-06 | 1.03E-06 | Downregulated |
| DDHD2 | 218.4587156 | 162.4497041 | -0.42737 | 1.03E-06 | 1.03E-06 | Downregulated |
| CLEC4E | 212.7614679 | 158.4378698 | -0.42532 | 2.15E-06 | 2.15E-06 | Downregulated |
| GATA5 | 98.26605505 | 73.23076923 | -0.42424 | 0.002354 | 0.002354 | Downregulated |
| PPT1 | 218.2706422 | 162.6923077 | -0.42397 | 1.25E-06 | 1.25E-06 | Downregulated |
| WNT8B | 184.8119266 | 137.7988166 | -0.42349 | 3.01E-05 | 3.01E-05 | Downregulated |
| IL12A | 209.3073394 | 156.147929 | -0.42271 | 3.36E-06 | 3.36E-06 | Downregulated |
| TMEM74B | 218.1192661 | 162.887574 | -0.42124 | 1.46E-06 | 1.46E-06 | Downregulated |
| C15orf53 | 94.44495413 | 70.58579882 | -0.4201 | 0.013646 | 0.013646 | Downregulated |
| RD3L | 1.472477064 | 1.100591716 | -0.41997 | 0.016027 | 0.016027 | Downregulated |
| CYP26C1 | 73 | 54.59171598 | -0.41921 | 0.032651 | 0.032651 | Downregulated |
| RBL1 | 218.0045872 | 163.035503 | -0.41917 | 1.63E-06 | 1.63E-06 | Downregulated |
| EDEM2 | 217.5275229 | 162.6982249 | -0.419 | 1.55E-06 | 1.55E-06 | Downregulated |
| FAM218A | 122.7752294 | 91.87573964 | -0.41826 | 0.001287 | 0.001287 | Downregulated |
| CCDC117 | 217.9541284 | 163.1005917 | -0.41826 | 1.72E-06 | 1.72E-06 | Downregulated |
| THOC5 | 217.8807339 | 163.1952663 | -0.41694 | 1.85E-06 | 1.85E-06 | Downregulated |
| HELQ | 217.7798165 | 163.3254438 | -0.41512 | 2.04E-06 | 2.04E-06 | Downregulated |
| CLDN8 | 69.09174312 | 51.84023669 | -0.41444 | 0.015737 | 0.015737 | Downregulated |
| CTDSPL2 | 217.7018349 | 163.4260355 | -0.41372 | 2.20E-06 | 2.20E-06 | Downregulated |
| DDX27 | 217.6880734 | 163.443787 | -0.41347 | 2.23E-06 | 2.23E-06 | Downregulated |
| RBKS | 217.6743119 | 163.4615385 | -0.41322 | 2.27E-06 | 2.27E-06 | Downregulated |
| AP000781.2 | 84.9587156 | 63.82248521 | -0.4127 | 0.006768 | 0.006768 | Downregulated |
| PIGU | 217.6284404 | 163.5207101 | -0.41239 | 2.37E-06 | 2.37E-06 | Downregulated |
| TBCEL | 217.6284404 | 163.5207101 | -0.41239 | 2.37E-06 | 2.37E-06 | Downregulated |
| CNBD2 | 201.1376147 | 151.1538462 | -0.41217 | 1.19E-05 | 1.19E-05 | Downregulated |
| GABRR1 | 87.75688073 | 65.95266272 | -0.41208 | 0.024121 | 0.024121 | Downregulated |
| SRSF12 | 183.1743119 | 137.8106509 | -0.41053 | 5.70E-05 | 5.70E-05 | Downregulated |
| ARL13B | 217.3899083 | 163.8284024 | -0.4081 | 2.99E-06 | 2.99E-06 | Downregulated |
| FBXL20 | 217.3853211 | 163.8343195 | -0.40802 | 3.01E-06 | 3.01E-06 | Downregulated |
| ZNF625 | 94.13761468 | 70.98224852 | -0.40731 | 0.006629 | 0.006629 | Downregulated |
| SLC12A5 | 213.9266055 | 161.4615385 | -0.40593 | 4.71E-06 | 4.71E-06 | Downregulated |
| KCNS2 | 106.1743119 | 80.21301775 | -0.40453 | 0.004156 | 0.004156 | Downregulated |
| FADS3 | 217.1376147 | 164.1538462 | -0.40356 | 3.82E-06 | 3.82E-06 | Downregulated |
| CLEC1B | 65.18807339 | 49.29585799 | -0.40314 | 0.022604 | 0.022604 | Downregulated |
| RAI14 | 217.0504587 | 164.2662722 | -0.40199 | 4.15E-06 | 4.15E-06 | Downregulated |
| SMTNL2 | 213.7110092 | 161.739645 | -0.40199 | 5.81E-06 | 5.81E-06 | Downregulated |
| CLEC4D | 132.4449541 | 100.3846154 | -0.39985 | 0.00128 | 0.00128 | Downregulated |
| HIST2H3A | 1.724770642 | 1.307692308 | -0.39938 | 0.030567 | 0.030567 | Downregulated |
| ZNF543 | 216.793578 | 164.5976331 | -0.39738 | 5.31E-06 | 5.31E-06 | Downregulated |
| EEF2K | 216.7706422 | 164.6272189 | -0.39697 | 5.42E-06 | 5.42E-06 | Downregulated |
| WDR17 | 154.0642202 | 117.0177515 | -0.3968 | 0.000665 | 0.000665 | Downregulated |
| DBNDD2 | 216.7155963 | 164.6982249 | -0.39598 | 5.71E-06 | 5.71E-06 | Downregulated |
| PLCL1 | 216.6697248 | 164.7573964 | -0.39515 | 5.97E-06 | 5.97E-06 | Downregulated |
| WASF3 | 216.6513761 | 164.7810651 | -0.39482 | 6.07E-06 | 6.07E-06 | Downregulated |
| RWDD2A | 216.6513761 | 164.7810651 | -0.39482 | 6.07E-06 | 6.07E-06 | Downregulated |
| POFUT1 | 216.6330275 | 164.8047337 | -0.3945 | 6.18E-06 | 6.18E-06 | Downregulated |
| VNN3 | 189.646789 | 144.2899408 | -0.39434 | 5.52E-05 | 5.52E-05 | Downregulated |
| AC024270.1 | 216.6146789 | 164.8284024 | -0.39417 | 6.29E-06 | 6.29E-06 | Downregulated |
| C4orf50 | 85.94495413 | 65.40828402 | -0.39394 | 0.04323 | 0.04323 | Downregulated |
| TMOD2 | 216.5366972 | 164.9289941 | -0.39277 | 6.76E-06 | 6.76E-06 | Downregulated |
| PCSK1N | 197.9862385 | 150.8343195 | -0.39244 | 3.52E-05 | 3.52E-05 | Downregulated |
| TSPAN2 | 216.5091743 | 164.964497 | -0.39227 | 6.94E-06 | 6.94E-06 | Downregulated |
| SSTR2 | 216.4816514 | 165 | -0.39178 | 7.12E-06 | 7.12E-06 | Downregulated |
| MAFA | 129.5412844 | 98.80473373 | -0.39076 | 0.003222 | 0.003222 | Downregulated |
| ZYG11A | 132.9724771 | 101.4911243 | -0.38977 | 0.003188 | 0.003188 | Downregulated |
| RIMS2 | 104.1513761 | 79.64497041 | -0.38703 | 0.009594 | 0.009594 | Downregulated |
| RNF26 | 216.1880734 | 165.3786982 | -0.38651 | 9.37E-06 | 9.37E-06 | Downregulated |
| KDELC1 | 216.1697248 | 165.4023669 | -0.38618 | 9.53E-06 | 9.53E-06 | Downregulated |
| UPB1 | 188.2018349 | 144.0177515 | -0.38603 | 9.93E-05 | 9.93E-05 | Downregulated |
| CCNE2 | 216.059633 | 165.5443787 | -0.38421 | 1.05E-05 | 1.05E-05 | Downregulated |
| ZNF549 | 216.0366972 | 165.5739645 | -0.3838 | 1.08E-05 | 1.08E-05 | Downregulated |
| TPD52L2 | 216.0366972 | 165.5739645 | -0.3838 | 1.08E-05 | 1.08E-05 | Downregulated |
| KBTBD6 | 216.0275229 | 165.5857988 | -0.38364 | 1.09E-05 | 1.09E-05 | Downregulated |
| TUBD1 | 216.0137615 | 165.6035503 | -0.38339 | 1.10E-05 | 1.10E-05 | Downregulated |
| ZNF572 | 215.9816514 | 165.6449704 | -0.38281 | 1.13E-05 | 1.13E-05 | Downregulated |
| PNLIPRP1 | 150.646789 | 115.6745562 | -0.3811 | 0.001409 | 0.001409 | Downregulated |
| CNEP1R1 | 215.8715596 | 165.7869822 | -0.38084 | 1.25E-05 | 1.25E-05 | Downregulated |
| GDF10 | 151.5412844 | 116.4319527 | -0.38022 | 0.001442 | 0.001442 | Downregulated |
| RAD21 | 215.8256881 | 165.8461538 | -0.38002 | 1.31E-05 | 1.31E-05 | Downregulated |
| GID8 | 215.8211009 | 165.852071 | -0.37994 | 1.31E-05 | 1.31E-05 | Downregulated |
| ELOVL3 | 192.0642202 | 147.6153846 | -0.37975 | 0.000106 | 0.000106 | Downregulated |
| TMEM67 | 215.8073394 | 165.8698225 | -0.37969 | 1.33E-05 | 1.33E-05 | Downregulated |
| POLR2B | 214.9036697 | 165.1775148 | -0.37967 | 1.42E-05 | 1.42E-05 | Downregulated |
| ROS1 | 154.3027523 | 118.6390533 | -0.37918 | 0.000903 | 0.000903 | Downregulated |
| WRB | 215.7706422 | 165.9171598 | -0.37904 | 1.37E-05 | 1.37E-05 | Downregulated |
| VSTM1 | 108.6009174 | 83.50887574 | -0.37903 | 0.005915 | 0.005915 | Downregulated |
| TRIM37 | 215.7477064 | 165.9467456 | -0.37862 | 1.40E-05 | 1.40E-05 | Downregulated |
| SNIP1 | 215.733945 | 165.964497 | -0.37838 | 1.42E-05 | 1.42E-05 | Downregulated |
| KCNT1 | 196.1834862 | 150.9763314 | -0.37788 | 8.13E-05 | 8.13E-05 | Downregulated |
| FBXO3 | 215.6880734 | 166.0236686 | -0.37756 | 1.48E-05 | 1.48E-05 | Downregulated |
| LRRK2 | 212.3302752 | 163.5207101 | -0.37684 | 2.07E-05 | 2.07E-05 | Downregulated |
| HOOK3 | 215.5963303 | 166.1420118 | -0.37592 | 1.61E-05 | 1.61E-05 | Downregulated |
| CYP11B2 | 1.550458716 | 1.195266272 | -0.37536 | 0.028378 | 0.028378 | Downregulated |
| APPBP2 | 215.5550459 | 166.1952663 | -0.37518 | 1.67E-05 | 1.67E-05 | Downregulated |
| MFSD11 | 215.5458716 | 166.2071006 | -0.37501 | 1.68E-05 | 1.68E-05 | Downregulated |
| HYDIN | 118.8807339 | 91.78698225 | -0.37315 | 0.011808 | 0.011808 | Downregulated |
| CTNNBL1 | 215.412844 | 166.3786982 | -0.37263 | 1.90E-05 | 1.90E-05 | Downregulated |
| GBP6 | 121.3348624 | 93.73372781 | -0.37235 | 0.010159 | 0.010159 | Downregulated |
| TSPAN6 | 215.3899083 | 166.408284 | -0.37222 | 1.94E-05 | 1.94E-05 | Downregulated |
| OIT3 | 212.059633 | 163.8698225 | -0.37192 | 2.65E-05 | 2.65E-05 | Downregulated |
| TMEM82 | 210.9587156 | 163.0236686 | -0.37188 | 2.89E-05 | 2.89E-05 | Downregulated |
| DIRC2 | 215.3669725 | 166.4378698 | -0.37181 | 1.98E-05 | 1.98E-05 | Downregulated |
| GPR82 | 209.853211 | 162.1893491 | -0.3717 | 3.18E-05 | 3.18E-05 | Downregulated |
| KCNA2 | 145.6834862 | 112.6094675 | -0.37151 | 0.001757 | 0.001757 | Downregulated |
| EIF5 | 215.3073394 | 166.5147929 | -0.37075 | 2.09E-05 | 2.09E-05 | Downregulated |
| POTEE | 108.3577982 | 83.82248521 | -0.37039 | 0.033468 | 0.033468 | Downregulated |
| CLEC1A | 214.1330275 | 165.7455621 | -0.36954 | 2.44E-05 | 2.44E-05 | Downregulated |
| AIRE | 170.2293578 | 131.7928994 | -0.36921 | 0.000451 | 0.000451 | Downregulated |
| ZNF555 | 215.1880734 | 166.6686391 | -0.36862 | 2.32E-05 | 2.32E-05 | Downregulated |
| ZNF426 | 215.1834862 | 166.6745562 | -0.36853 | 2.33E-05 | 2.33E-05 | Downregulated |
| CHST15 | 215.1743119 | 166.6863905 | -0.36837 | 2.35E-05 | 2.35E-05 | Downregulated |
| CFL2 | 215.1697248 | 166.6923077 | -0.36829 | 2.36E-05 | 2.36E-05 | Downregulated |
| HOXC11 | 122.8715596 | 95.18934911 | -0.36828 | 0.021191 | 0.021191 | Downregulated |
| ZNF43 | 215.1376147 | 166.7337278 | -0.36771 | 2.43E-05 | 2.43E-05 | Downregulated |
| SCAMP5 | 215.1146789 | 166.7633136 | -0.3673 | 2.48E-05 | 2.48E-05 | Downregulated |
| CYP20A1 | 215.0917431 | 166.7928994 | -0.36689 | 2.53E-05 | 2.53E-05 | Downregulated |
| NDUFAF6 | 215.0642202 | 166.8284024 | -0.3664 | 2.59E-05 | 2.59E-05 | Downregulated |
| PNMA5 | 74.56422018 | 57.88757396 | -0.36523 | 0.043516 | 0.043516 | Downregulated |
| FOXI2 | 87.38990826 | 67.87573964 | -0.36457 | 0.037906 | 0.037906 | Downregulated |
| SEC22C | 214.9541284 | 166.9704142 | -0.36444 | 2.85E-05 | 2.85E-05 | Downregulated |
| MRPL49 | 214.9220183 | 167.0118343 | -0.36386 | 2.93E-05 | 2.93E-05 | Downregulated |
| ZNF2 | 214.3073394 | 166.5384615 | -0.36383 | 2.86E-05 | 2.86E-05 | Downregulated |
| AC106782.1 | 130.3394495 | 101.3195266 | -0.36336 | 0.005381 | 0.005381 | Downregulated |
| KIAA1586 | 214.8486239 | 167.1065089 | -0.36255 | 3.13E-05 | 3.13E-05 | Downregulated |
| SNURF | 126.0275229 | 98.06508876 | -0.36193 | 0.01614 | 0.01614 | Downregulated |
| SLC6A4 | 204.9541284 | 159.5266272 | -0.3615 | 7.35E-05 | 7.35E-05 | Downregulated |
| ACVR2A | 214.7706422 | 167.2071006 | -0.36116 | 3.35E-05 | 3.35E-05 | Downregulated |
| STARD7 | 214.7614679 | 167.2189349 | -0.361 | 3.38E-05 | 3.38E-05 | Downregulated |
| ATG14 | 214.7614679 | 167.2189349 | -0.361 | 3.38E-05 | 3.38E-05 | Downregulated |
| ST8SIA4 | 214.7385321 | 167.2485207 | -0.36059 | 3.45E-05 | 3.45E-05 | Downregulated |
| SLCO3A1 | 214.7110092 | 167.2840237 | -0.3601 | 3.53E-05 | 3.53E-05 | Downregulated |
| ZKSCAN2 | 214.6972477 | 167.3017751 | -0.35985 | 3.57E-05 | 3.57E-05 | Downregulated |
| ETAA1 | 214.6834862 | 167.3195266 | -0.35961 | 3.61E-05 | 3.61E-05 | Downregulated |
| SAMSN1 | 214.6743119 | 167.3313609 | -0.35944 | 3.64E-05 | 3.64E-05 | Downregulated |
| YTHDF1 | 214.6697248 | 167.3372781 | -0.35936 | 3.66E-05 | 3.66E-05 | Downregulated |
| TMEM133 | 214.6651376 | 167.3431953 | -0.35928 | 3.67E-05 | 3.67E-05 | Downregulated |
| RAD1 | 214.6422018 | 167.3727811 | -0.35887 | 3.75E-05 | 3.75E-05 | Downregulated |
| ABHD12 | 214.6055046 | 167.4201183 | -0.35821 | 3.87E-05 | 3.87E-05 | Downregulated |
| MME | 214.6055046 | 167.4201183 | -0.35821 | 3.87E-05 | 3.87E-05 | Downregulated |
| CNNM2 | 214.5366972 | 167.3964497 | -0.35796 | 3.86E-05 | 3.86E-05 | Downregulated |
| ARL11 | 214.587156 | 167.443787 | -0.35789 | 3.93E-05 | 3.93E-05 | Downregulated |
| IL34 | 214.587156 | 167.443787 | -0.35789 | 3.93E-05 | 3.93E-05 | Downregulated |
| PAPLN | 214.5688073 | 167.4674556 | -0.35756 | 3.99E-05 | 3.99E-05 | Downregulated |
| ZNF439 | 214.5321101 | 167.5147929 | -0.35691 | 4.12E-05 | 4.12E-05 | Downregulated |
| PAPOLG | 214.4908257 | 167.5680473 | -0.35617 | 4.27E-05 | 4.27E-05 | Downregulated |
| ZNF681 | 214.4587156 | 167.6094675 | -0.3556 | 4.39E-05 | 4.39E-05 | Downregulated |
| SNX21 | 214.4266055 | 167.6508876 | -0.35502 | 4.51E-05 | 4.51E-05 | Downregulated |
| SLC35G2 | 214.4220183 | 167.6568047 | -0.35494 | 4.53E-05 | 4.53E-05 | Downregulated |
| MPP5 | 214.412844 | 167.6686391 | -0.35478 | 4.57E-05 | 4.57E-05 | Downregulated |
| SGK3 | 214.3761468 | 167.7159763 | -0.35412 | 4.71E-05 | 4.71E-05 | Downregulated |
| MTBP | 214.3486239 | 167.7514793 | -0.35363 | 4.83E-05 | 4.83E-05 | Downregulated |
| GNG2 | 214.3256881 | 167.7810651 | -0.35322 | 4.92E-05 | 4.92E-05 | Downregulated |
| DENND6A | 214.3256881 | 167.7810651 | -0.35322 | 4.92E-05 | 4.92E-05 | Downregulated |
| ACTR3C | 214.2568807 | 167.8698225 | -0.352 | 5.22E-05 | 5.22E-05 | Downregulated |
| MDH1B | 204.4357798 | 160.1952663 | -0.35182 | 0.000111 | 0.000111 | Downregulated |
| NEU1 | 214.2293578 | 167.9053254 | -0.35151 | 5.34E-05 | 5.34E-05 | Downregulated |
| IL24 | 214.2110092 | 167.9289941 | -0.35118 | 5.43E-05 | 5.43E-05 | Downregulated |
| ZNF257 | 188.5642202 | 147.8284024 | -0.35113 | 0.00043 | 0.00043 | Downregulated |
| CD38 | 214.1926606 | 167.9526627 | -0.35085 | 5.51E-05 | 5.51E-05 | Downregulated |
| NUDT21 | 214.1834862 | 167.964497 | -0.35069 | 5.56E-05 | 5.56E-05 | Downregulated |
| FMO1 | 210.8853211 | 165.3846154 | -0.35063 | 7.23E-05 | 7.23E-05 | Downregulated |
| QKI | 214.1743119 | 167.9763314 | -0.35053 | 5.60E-05 | 5.60E-05 | Downregulated |
| DENND5B | 214.146789 | 168.0118343 | -0.35004 | 5.73E-05 | 5.73E-05 | Downregulated |
| TMIGD1 | 130.7293578 | 102.5976331 | -0.34959 | 0.003557 | 0.003557 | Downregulated |
| THAP9 | 214.1009174 | 168.0710059 | -0.34922 | 5.96E-05 | 5.96E-05 | Downregulated |
| 3-Sep | 214.0963303 | 168.0769231 | -0.34914 | 5.98E-05 | 5.98E-05 | Downregulated |
| NELFCD | 214.087156 | 168.0887574 | -0.34898 | 6.03E-05 | 6.03E-05 | Downregulated |
| HACD1 | 214.0366972 | 168.1538462 | -0.34808 | 6.29E-05 | 6.29E-05 | Downregulated |
| PHACTR2 | 214.0366972 | 168.1538462 | -0.34808 | 6.29E-05 | 6.29E-05 | Downregulated |
| ARMC8 | 214.0137615 | 168.183432 | -0.34767 | 6.41E-05 | 6.41E-05 | Downregulated |
| TMEM65 | 214.0091743 | 168.1893491 | -0.34759 | 6.44E-05 | 6.44E-05 | Downregulated |
| BMF | 214.0091743 | 168.1893491 | -0.34759 | 6.44E-05 | 6.44E-05 | Downregulated |
| SLITRK4 | 163.4449541 | 128.4556213 | -0.34753 | 0.001407 | 0.001407 | Downregulated |
| CAMKV | 184.2752294 | 144.8284024 | -0.34752 | 0.000598 | 0.000598 | Downregulated |
| ARHGAP42 | 213.9816514 | 168.2248521 | -0.3471 | 6.59E-05 | 6.59E-05 | Downregulated |
| VCPIP1 | 213.9541284 | 168.260355 | -0.34661 | 6.74E-05 | 6.74E-05 | Downregulated |
| HSD17B1 | 213.9449541 | 168.2721893 | -0.34644 | 6.80E-05 | 6.80E-05 | Downregulated |
| TKTL1 | 103.8027523 | 81.68047337 | -0.34578 | 0.016461 | 0.016461 | Downregulated |
| ADAM20 | 86.94954128 | 68.44378698 | -0.34526 | 0.047072 | 0.047072 | Downregulated |
| ING3 | 213.8669725 | 168.3727811 | -0.34505 | 7.26E-05 | 7.26E-05 | Downregulated |
| ATP6V1C2 | 213.7706422 | 168.4970414 | -0.34334 | 7.86E-05 | 7.86E-05 | Downregulated |
| NDUFAF7 | 213.766055 | 168.5029586 | -0.34326 | 7.90E-05 | 7.90E-05 | Downregulated |
| AGO1 | 213.7522936 | 168.5207101 | -0.34301 | 7.99E-05 | 7.99E-05 | Downregulated |
| RAD51B | 213.7155963 | 168.5680473 | -0.34236 | 8.23E-05 | 8.23E-05 | Downregulated |
| ZNF14 | 213.706422 | 168.5798817 | -0.3422 | 8.30E-05 | 8.30E-05 | Downregulated |
| AQP12A | 126.206422 | 99.58579882 | -0.34177 | 0.011952 | 0.011952 | Downregulated |
| PPP1CB | 212.9174312 | 168.035503 | -0.34153 | 8.67E-05 | 8.67E-05 | Downregulated |
| CCDC169 | 109.8853211 | 86.73372781 | -0.34133 | 0.0175 | 0.0175 | Downregulated |
| GPATCH11 | 213.6559633 | 168.6449704 | -0.3413 | 8.65E-05 | 8.65E-05 | Downregulated |
| MPP6 | 213.6513761 | 168.6508876 | -0.34122 | 8.69E-05 | 8.69E-05 | Downregulated |
| LGI4 | 213.6422018 | 168.6627219 | -0.34106 | 8.75E-05 | 8.75E-05 | Downregulated |
| INTS2 | 213.0825688 | 168.2248521 | -0.34102 | 8.51E-05 | 8.51E-05 | Downregulated |
| ZBTB8B | 91.81192661 | 72.4852071 | -0.34099 | 0.025946 | 0.025946 | Downregulated |
| NABP1 | 213.6376147 | 168.6686391 | -0.34097 | 8.78E-05 | 8.78E-05 | Downregulated |
| THAP1 | 213.6100917 | 168.704142 | -0.34048 | 8.99E-05 | 8.99E-05 | Downregulated |
| F2RL2 | 213.6100917 | 168.704142 | -0.34048 | 8.99E-05 | 8.99E-05 | Downregulated |
| INPP5B | 213.2568807 | 168.4378698 | -0.34038 | 8.69E-05 | 8.69E-05 | Downregulated |
| CAPN11 | 204.8944954 | 161.8402367 | -0.34031 | 0.000173 | 0.000173 | Downregulated |
| UBE2W | 213.5963303 | 168.7218935 | -0.34024 | 9.09E-05 | 9.09E-05 | Downregulated |
| KCNIP1 | 105.9541284 | 83.69822485 | -0.34017 | 0.036382 | 0.036382 | Downregulated |
| USP42 | 213.5917431 | 168.7278107 | -0.34016 | 9.13E-05 | 9.13E-05 | Downregulated |
| DDHD1 | 213.5504587 | 168.7810651 | -0.33942 | 9.44E-05 | 9.44E-05 | Downregulated |
| SERINC1 | 213.5458716 | 168.7869822 | -0.33934 | 9.48E-05 | 9.48E-05 | Downregulated |
| PCIF1 | 213.5366972 | 168.7988166 | -0.33918 | 9.55E-05 | 9.55E-05 | Downregulated |
| CALCRL | 213.5321101 | 168.8047337 | -0.3391 | 9.59E-05 | 9.59E-05 | Downregulated |
| WDCP | 212.7889908 | 168.3076923 | -0.33832 | 9.79E-05 | 9.79E-05 | Downregulated |
| GDPD4 | 139.9357798 | 110.704142 | -0.33806 | 0.018135 | 0.018135 | Downregulated |
| CCDC82 | 213.4724771 | 168.8816568 | -0.33804 | 0.000101 | 0.000101 | Downregulated |
| ADAM17 | 213.4724771 | 168.8816568 | -0.33804 | 0.000101 | 0.000101 | Downregulated |
| ZNF699 | 212.3394495 | 168.0591716 | -0.3374 | 0.000113 | 0.000113 | Downregulated |
| BMP8B | 213.3944954 | 168.9822485 | -0.33665 | 0.000107 | 0.000107 | Downregulated |
| SMOC1 | 213.3944954 | 168.9822485 | -0.33665 | 0.000107 | 0.000107 | Downregulated |
| TUSC3 | 213.3715596 | 169.0118343 | -0.33624 | 0.000109 | 0.000109 | Downregulated |
| SRSF6 | 213.3669725 | 169.0177515 | -0.33616 | 0.00011 | 0.00011 | Downregulated |
| ZNF547 | 211.146789 | 167.3195266 | -0.33564 | 0.000132 | 0.000132 | Downregulated |
| ZNF813 | 213.3302752 | 169.0650888 | -0.33551 | 0.000113 | 0.000113 | Downregulated |
| CCDC163 | 213.3211009 | 169.0769231 | -0.33535 | 0.000114 | 0.000114 | Downregulated |
| NCBP2L | 2.02293578 | 1.603550296 | -0.33518 | 0.046222 | 0.046222 | Downregulated |
| CCNYL1 | 213.2614679 | 169.1538462 | -0.33429 | 0.00012 | 0.00012 | Downregulated |
| KCNAB2 | 213.1697248 | 169.2721893 | -0.33266 | 0.000129 | 0.000129 | Downregulated |
| GRIN2C | 212.0688073 | 168.408284 | -0.33257 | 0.00014 | 0.00014 | Downregulated |
| SLC35E3 | 213.1330275 | 169.3195266 | -0.33201 | 0.000133 | 0.000133 | Downregulated |
| XRN1 | 213.1192661 | 169.3372781 | -0.33176 | 0.000134 | 0.000134 | Downregulated |
| TMEM151A | 195.9082569 | 155.704142 | -0.33137 | 0.000447 | 0.000447 | Downregulated |
| CYP7B1 | 210.8990826 | 167.6390533 | -0.33119 | 0.000161 | 0.000161 | Downregulated |
| RBMS1 | 213.087156 | 169.3786982 | -0.33119 | 0.000138 | 0.000138 | Downregulated |
| ERLEC1 | 213.0825688 | 169.3846154 | -0.33111 | 0.000138 | 0.000138 | Downregulated |
| TFEC | 213.0825688 | 169.3846154 | -0.33111 | 0.000138 | 0.000138 | Downregulated |
| PSMD11 | 213.0779817 | 169.3905325 | -0.33103 | 0.000139 | 0.000139 | Downregulated |
| HTRA3 | 213.0779817 | 169.3905325 | -0.33103 | 0.000139 | 0.000139 | Downregulated |
| TPP1 | 213.0733945 | 169.3964497 | -0.33095 | 0.000139 | 0.000139 | Downregulated |
| SCARB2 | 213.0550459 | 169.4201183 | -0.33062 | 0.000141 | 0.000141 | Downregulated |
| APELA | 191.6605505 | 152.4615385 | -0.33011 | 0.000571 | 0.000571 | Downregulated |
| HIST1H2AD | 203.2706422 | 161.6982249 | -0.3301 | 0.000281 | 0.000281 | Downregulated |
| S100A12 | 159.8348624 | 127.147929 | -0.33007 | 0.002546 | 0.002546 | Downregulated |
| ZNF670 | 213.0229358 | 169.4615385 | -0.33005 | 0.000145 | 0.000145 | Downregulated |
| OTOP2 | 80.44495413 | 64 | -0.32993 | 0.033971 | 0.033971 | Downregulated |
| GPR34 | 213.0045872 | 169.4852071 | -0.32973 | 0.000147 | 0.000147 | Downregulated |
| CYSLTR1 | 213 | 169.4911243 | -0.32964 | 0.000148 | 0.000148 | Downregulated |
| RASSF8 | 212.9862385 | 169.5088757 | -0.3294 | 0.00015 | 0.00015 | Downregulated |
| SHISA9 | 139.6146789 | 111.1183432 | -0.32935 | 0.01015 | 0.01015 | Downregulated |
| AOX1 | 208.6055046 | 166.0591716 | -0.32908 | 0.000206 | 0.000206 | Downregulated |
| EFHC1 | 212.9678899 | 169.5325444 | -0.32907 | 0.000152 | 0.000152 | Downregulated |
| PRDM13 | 98.08256881 | 78.08284024 | -0.32899 | 0.03893 | 0.03893 | Downregulated |
| BCL11B | 212.9357798 | 169.5739645 | -0.3285 | 0.000156 | 0.000156 | Downregulated |
| GTF2E1 | 212.9311927 | 169.5798817 | -0.32842 | 0.000156 | 0.000156 | Downregulated |
| ZNF724 | 212.9266055 | 169.5857988 | -0.32834 | 0.000157 | 0.000157 | Downregulated |
| GLCCI1 | 212.9266055 | 169.5857988 | -0.32834 | 0.000157 | 0.000157 | Downregulated |
| NAPB | 212.9220183 | 169.591716 | -0.32826 | 0.000157 | 0.000157 | Downregulated |
| TIFA | 212.9174312 | 169.5976331 | -0.32818 | 0.000158 | 0.000158 | Downregulated |
| SMG8 | 212.8944954 | 169.6272189 | -0.32777 | 0.000161 | 0.000161 | Downregulated |
| METTL6 | 212.8807339 | 169.6449704 | -0.32753 | 0.000163 | 0.000163 | Downregulated |
| NFATC2 | 212.8302752 | 169.7100592 | -0.32663 | 0.000169 | 0.000169 | Downregulated |
| RERGL | 86.51834862 | 69 | -0.32641 | 0.030593 | 0.030593 | Downregulated |
| OR2B6 | 126.5321101 | 100.9230769 | -0.32625 | 0.011661 | 0.011661 | Downregulated |
| DDX19B | 212.7981651 | 169.7514793 | -0.32606 | 0.000174 | 0.000174 | Downregulated |
| CHUK | 212.793578 | 169.7573964 | -0.32598 | 0.000175 | 0.000175 | Downregulated |
| GINS4 | 212.7844037 | 169.7692308 | -0.32582 | 0.000176 | 0.000176 | Downregulated |
| NOL9 | 212.7706422 | 169.7869822 | -0.32557 | 0.000178 | 0.000178 | Downregulated |
| C17orf80 | 212.7614679 | 169.7988166 | -0.32541 | 0.000179 | 0.000179 | Downregulated |
| PDRG1 | 212.7522936 | 169.8106509 | -0.32525 | 0.00018 | 0.00018 | Downregulated |
| ZBED9 | 152.1376147 | 121.4319527 | -0.32523 | 0.00426 | 0.00426 | Downregulated |
| ABCD2 | 196.6330275 | 156.964497 | -0.32507 | 0.000555 | 0.000555 | Downregulated |
| KCNF1 | 189.3302752 | 151.1420118 | -0.325 | 0.000867 | 0.000867 | Downregulated |
| CYSLTR2 | 204.059633 | 162.9171598 | -0.32485 | 0.000328 | 0.000328 | Downregulated |
| GYPE | 135.0504587 | 107.8343195 | -0.32468 | 0.009202 | 0.009202 | Downregulated |
| HIST1H3D | 212.7201835 | 169.852071 | -0.32468 | 0.000185 | 0.000185 | Downregulated |
| HILPDA | 212.7155963 | 169.8579882 | -0.3246 | 0.000186 | 0.000186 | Downregulated |
| P2RY13 | 212.7155963 | 169.8579882 | -0.3246 | 0.000186 | 0.000186 | Downregulated |
| GPR88 | 119.0045872 | 95.0295858 | -0.32457 | 0.013889 | 0.013889 | Downregulated |
| SOGA3 | 153.0366972 | 122.2071006 | -0.32455 | 0.0061 | 0.0061 | Downregulated |
| ZNF689 | 212.6972477 | 169.8816568 | -0.32427 | 0.000188 | 0.000188 | Downregulated |
| TRPC4AP | 212.6743119 | 169.9112426 | -0.32386 | 0.000192 | 0.000192 | Downregulated |
| CA4 | 194.4678899 | 155.3727811 | -0.3238 | 0.000577 | 0.000577 | Downregulated |
| RIT1 | 212.6651376 | 169.9230769 | -0.3237 | 0.000193 | 0.000193 | Downregulated |
| ATP6V1E1 | 212.6513761 | 169.9408284 | -0.32346 | 0.000195 | 0.000195 | Downregulated |
| PIPOX | 212.6055046 | 170 | -0.32264 | 0.000203 | 0.000203 | Downregulated |
| AGO4 | 212.6055046 | 170 | -0.32264 | 0.000203 | 0.000203 | Downregulated |
| TRAM1 | 212.6055046 | 170 | -0.32264 | 0.000203 | 0.000203 | Downregulated |
| C8orf37 | 211.5091743 | 169.1301775 | -0.32259 | 0.000219 | 0.000219 | Downregulated |
| PI15 | 203.9311927 | 163.0828402 | -0.32248 | 0.000367 | 0.000367 | Downregulated |
| PAG1 | 212.587156 | 170.0236686 | -0.32232 | 0.000206 | 0.000206 | Downregulated |
| ABI2 | 212.5779817 | 170.035503 | -0.32216 | 0.000207 | 0.000207 | Downregulated |
| SRRD | 212.5733945 | 170.0414201 | -0.32207 | 0.000208 | 0.000208 | Downregulated |
| PNLIP | 2.5 | 2 | -0.32193 | 0.04498 | 0.04498 | Downregulated |
| HIST1H3H | 212.559633 | 170.0591716 | -0.32183 | 0.00021 | 0.00021 | Downregulated |
| ECEL1 | 187.1009174 | 149.7159763 | -0.32159 | 0.000876 | 0.000876 | Downregulated |
| CGRRF1 | 212.5412844 | 170.0828402 | -0.32151 | 0.000213 | 0.000213 | Downregulated |
| AZIN1 | 212.5366972 | 170.0887574 | -0.32142 | 0.000214 | 0.000214 | Downregulated |
| GRPR | 189.146789 | 151.3786982 | -0.32134 | 0.00092 | 0.00092 | Downregulated |
| SLC30A10 | 109.3027523 | 87.4852071 | -0.32122 | 0.035267 | 0.035267 | Downregulated |
| HSF2 | 212.5091743 | 170.1242604 | -0.32094 | 0.000219 | 0.000219 | Downregulated |
| ZFP1 | 212.5091743 | 170.1242604 | -0.32094 | 0.000219 | 0.000219 | Downregulated |
| MAPK12 | 212.5 | 170.1360947 | -0.32077 | 0.00022 | 0.00022 | Downregulated |
| CPSF3 | 212.5 | 170.1360947 | -0.32077 | 0.00022 | 0.00022 | Downregulated |
| ADGRG5 | 212.4908257 | 170.147929 | -0.32061 | 0.000222 | 0.000222 | Downregulated |
| FLT3 | 190.1422018 | 152.2544379 | -0.32059 | 0.000846 | 0.000846 | Downregulated |
| C17orf67 | 212.4862385 | 170.1538462 | -0.32053 | 0.000223 | 0.000223 | Downregulated |
| DOCK4 | 212.4862385 | 170.1538462 | -0.32053 | 0.000223 | 0.000223 | Downregulated |
| ZFP92 | 211.3577982 | 169.3254438 | -0.31989 | 0.000247 | 0.000247 | Downregulated |
| IL5RA | 154.7018349 | 123.9467456 | -0.31977 | 0.004972 | 0.004972 | Downregulated |
| STKLD1 | 210.2568807 | 168.4674556 | -0.31968 | 0.000267 | 0.000267 | Downregulated |
| ZNF705E | 113.1834862 | 90.73372781 | -0.31895 | 0.024091 | 0.024091 | Downregulated |
| UTP18 | 212.3899083 | 170.2781065 | -0.31882 | 0.00024 | 0.00024 | Downregulated |
| CRY1 | 212.3715596 | 170.3017751 | -0.3185 | 0.000243 | 0.000243 | Downregulated |
| RDX | 212.3486239 | 170.3313609 | -0.31809 | 0.000248 | 0.000248 | Downregulated |
| ETNK2 | 212.3348624 | 170.3491124 | -0.31785 | 0.000251 | 0.000251 | Downregulated |
| XKR6 | 206.9036697 | 165.9940828 | -0.31783 | 0.000359 | 0.000359 | Downregulated |
| PEX12 | 212.3302752 | 170.3550296 | -0.31777 | 0.000251 | 0.000251 | Downregulated |
| USP39 | 212.3211009 | 170.3668639 | -0.3176 | 0.000253 | 0.000253 | Downregulated |
| SNX10 | 212.3165138 | 170.3727811 | -0.31752 | 0.000254 | 0.000254 | Downregulated |
| CXCL5 | 211.2155963 | 169.5088757 | -0.31736 | 0.000275 | 0.000275 | Downregulated |
| ART4 | 144.5229358 | 115.9881657 | -0.31732 | 0.00505 | 0.00505 | Downregulated |
| TEFM | 212.3027523 | 170.3905325 | -0.31728 | 0.000257 | 0.000257 | Downregulated |
| TCEA2 | 212.2889908 | 170.408284 | -0.31703 | 0.00026 | 0.00026 | Downregulated |
| MAP4K2 | 212.2752294 | 170.4260355 | -0.31679 | 0.000262 | 0.000262 | Downregulated |
| ERCC4 | 212.2431193 | 170.4674556 | -0.31622 | 0.000269 | 0.000269 | Downregulated |
| ZNF440 | 212.2385321 | 170.4733728 | -0.31614 | 0.00027 | 0.00027 | Downregulated |
| BBS4 | 212.2293578 | 170.4852071 | -0.31598 | 0.000272 | 0.000272 | Downregulated |
| B4GALT5 | 212.2293578 | 170.4852071 | -0.31598 | 0.000272 | 0.000272 | Downregulated |
| DPP10 | 155.4724771 | 124.9053254 | -0.31582 | 0.003998 | 0.003998 | Downregulated |
| SRP72 | 212.2110092 | 170.5088757 | -0.31565 | 0.000276 | 0.000276 | Downregulated |
| RPN2 | 212.206422 | 170.5147929 | -0.31557 | 0.000277 | 0.000277 | Downregulated |
| SGTB | 212.2018349 | 170.5207101 | -0.31549 | 0.000278 | 0.000278 | Downregulated |
| UGT2B10 | 117.9036697 | 94.74556213 | -0.31548 | 0.019978 | 0.019978 | Downregulated |
| ZNF788 | 212.1834862 | 170.5443787 | -0.31517 | 0.000282 | 0.000282 | Downregulated |
| CACNA2D4 | 212.1788991 | 170.5502959 | -0.31508 | 0.000283 | 0.000283 | Downregulated |
| ZNF518B | 212.1651376 | 170.5680473 | -0.31484 | 0.000286 | 0.000286 | Downregulated |
| FAM209B | 160.1330275 | 128.7455621 | -0.31475 | 0.004497 | 0.004497 | Downregulated |
| CLASP2 | 212.1559633 | 170.5798817 | -0.31468 | 0.000288 | 0.000288 | Downregulated |
| HIST1H4D | 160.1284404 | 128.7514793 | -0.31464 | 0.003891 | 0.003891 | Downregulated |
| TSPAN1 | 212.1513761 | 170.5857988 | -0.3146 | 0.000289 | 0.000289 | Downregulated |
| CLEC7A | 212.1513761 | 170.5857988 | -0.3146 | 0.000289 | 0.000289 | Downregulated |
| KAT7 | 212.146789 | 170.591716 | -0.31452 | 0.00029 | 0.00029 | Downregulated |
| MAGEB17 | 156.353211 | 125.7278107 | -0.31451 | 0.006651 | 0.006651 | Downregulated |
| PRAMEF12 | 1.279816514 | 1.029585799 | -0.31387 | 0.019073 | 0.019073 | Downregulated |
| LITAF | 212.1009174 | 170.6508876 | -0.3137 | 0.0003 | 0.0003 | Downregulated |
| YPEL2 | 212.0963303 | 170.6568047 | -0.31362 | 0.000301 | 0.000301 | Downregulated |
| EDN3 | 208.8211009 | 168.0473373 | -0.3134 | 0.000374 | 0.000374 | Downregulated |
| ARHGAP15 | 212.0825688 | 170.6745562 | -0.31338 | 0.000305 | 0.000305 | Downregulated |
| HIF3A | 212.0779817 | 170.6804734 | -0.3133 | 0.000306 | 0.000306 | Downregulated |
| LRCH2 | 209.8990826 | 168.9289941 | -0.31328 | 0.000351 | 0.000351 | Downregulated |
| THRB | 210.9816514 | 169.8106509 | -0.31319 | 0.00033 | 0.00033 | Downregulated |
| GSDMC | 202.3577982 | 162.8757396 | -0.31314 | 0.00056 | 0.00056 | Downregulated |
| MYO15A | 210.9770642 | 169.816568 | -0.31311 | 0.000331 | 0.000331 | Downregulated |
| PLA2G7 | 212.059633 | 170.704142 | -0.31297 | 0.00031 | 0.00031 | Downregulated |
| STXBP4 | 212.0550459 | 170.7100592 | -0.31289 | 0.000311 | 0.000311 | Downregulated |
| HBG1 | 1.47706422 | 1.189349112 | -0.31256 | 0.042281 | 0.042281 | Downregulated |
| PDCD1LG2 | 208.7706422 | 168.112426 | -0.31249 | 0.000389 | 0.000389 | Downregulated |
| ZNF331 | 212.0321101 | 170.739645 | -0.31248 | 0.000317 | 0.000317 | Downregulated |
| KIF3B | 212.0183486 | 170.7573964 | -0.31224 | 0.00032 | 0.00032 | Downregulated |
| GPR141 | 178.5321101 | 143.7988166 | -0.31213 | 0.002618 | 0.002618 | Downregulated |
| SNAI2 | 211.9954128 | 170.7869822 | -0.31184 | 0.000326 | 0.000326 | Downregulated |
| AKAP10 | 211.9954128 | 170.7869822 | -0.31184 | 0.000326 | 0.000326 | Downregulated |
| ARHGAP6 | 211.9816514 | 170.8047337 | -0.31159 | 0.000329 | 0.000329 | Downregulated |
| RERG | 211.9770642 | 170.8106509 | -0.31151 | 0.00033 | 0.00033 | Downregulated |
| CBARP | 211.9678899 | 170.8224852 | -0.31135 | 0.000333 | 0.000333 | Downregulated |
| LRP12 | 211.9587156 | 170.8343195 | -0.31119 | 0.000335 | 0.000335 | Downregulated |
| KCNS1 | 147.9036697 | 119.2130178 | -0.31112 | 0.008943 | 0.008943 | Downregulated |
| HPGD | 211.9449541 | 170.852071 | -0.31094 | 0.000339 | 0.000339 | Downregulated |
| HSFY2 | 1.431192661 | 1.153846154 | -0.31077 | 0.017142 | 0.017142 | Downregulated |
| KCNRG | 208.6743119 | 168.2366864 | -0.31076 | 0.000418 | 0.000418 | Downregulated |
| SCML2 | 211.9311927 | 170.8698225 | -0.3107 | 0.000342 | 0.000342 | Downregulated |
| FOXO1 | 211.9311927 | 170.8698225 | -0.3107 | 0.000342 | 0.000342 | Downregulated |
| KCNE1 | 144.266055 | 116.3195266 | -0.31064 | 0.006506 | 0.006506 | Downregulated |
| P2RY14 | 208.6605505 | 168.2544379 | -0.31051 | 0.000429 | 0.000429 | Downregulated |
| TCEANC | 211.9174312 | 170.887574 | -0.31045 | 0.000346 | 0.000346 | Downregulated |
| ARHGAP10 | 211.9082569 | 170.8994083 | -0.31029 | 0.000348 | 0.000348 | Downregulated |
| MYO16 | 199.0137615 | 160.5147929 | -0.31016 | 0.000766 | 0.000766 | Downregulated |
| ZBTB46 | 211.8944954 | 170.9171598 | -0.31005 | 0.000352 | 0.000352 | Downregulated |
| P2RY12 | 162.7798165 | 131.3136095 | -0.30991 | 0.004396 | 0.004396 | Downregulated |
| C21orf91 | 211.8669725 | 170.9526627 | -0.30956 | 0.000359 | 0.000359 | Downregulated |
| GRIN2A | 127.4449541 | 102.8343195 | -0.30955 | 0.017003 | 0.017003 | Downregulated |
| ZFP37 | 209.6880734 | 169.2011834 | -0.30951 | 0.000414 | 0.000414 | Downregulated |
| FPR2 | 198.9770642 | 160.5621302 | -0.30947 | 0.000771 | 0.000771 | Downregulated |
| DRC1 | 134.4908257 | 108.556213 | -0.30907 | 0.015543 | 0.015543 | Downregulated |
| PTPN1 | 211.8256881 | 171.0059172 | -0.30883 | 0.000371 | 0.000371 | Downregulated |
| PGF | 211.8211009 | 171.0118343 | -0.30875 | 0.000372 | 0.000372 | Downregulated |
| FCGR3B | 208.559633 | 168.3846154 | -0.3087 | 0.000458 | 0.000458 | Downregulated |
| P2RY6 | 211.7889908 | 171.0532544 | -0.30818 | 0.000381 | 0.000381 | Downregulated |
| ZNF267 | 211.7798165 | 171.0650888 | -0.30802 | 0.000384 | 0.000384 | Downregulated |
| CP | 210.6880734 | 170.1893491 | -0.30797 | 0.000413 | 0.000413 | Downregulated |
| WNT5A | 211.7706422 | 171.0769231 | -0.30786 | 0.000387 | 0.000387 | Downregulated |
| DGKE | 211.7706422 | 171.0769231 | -0.30786 | 0.000387 | 0.000387 | Downregulated |
| SLC7A10 | 107.3577982 | 86.73372781 | -0.30776 | 0.039106 | 0.039106 | Downregulated |
| TMEM68 | 211.7568807 | 171.0946746 | -0.30761 | 0.000391 | 0.000391 | Downregulated |
| STAT4 | 211.7568807 | 171.0946746 | -0.30761 | 0.000391 | 0.000391 | Downregulated |
| CLEC4A | 211.7431193 | 171.112426 | -0.30737 | 0.000395 | 0.000395 | Downregulated |
| FGF9 | 199.9220183 | 161.5621302 | -0.30735 | 0.000781 | 0.000781 | Downregulated |
| CDKN2B | 211.733945 | 171.1242604 | -0.30721 | 0.000398 | 0.000398 | Downregulated |
| BCAS2 | 211.733945 | 171.1242604 | -0.30721 | 0.000398 | 0.000398 | Downregulated |
| SNRK | 211.6972477 | 171.1715976 | -0.30656 | 0.000409 | 0.000409 | Downregulated |
| STC1 | 211.6972477 | 171.1715976 | -0.30656 | 0.000409 | 0.000409 | Downregulated |
| TMEM217 | 205.1788991 | 165.964497 | -0.30601 | 0.00062 | 0.00062 | Downregulated |
| TMEM170B | 211.646789 | 171.2366864 | -0.30567 | 0.000425 | 0.000425 | Downregulated |
| FLVCR2 | 211.646789 | 171.2366864 | -0.30567 | 0.000425 | 0.000425 | Downregulated |
| CHMP4B | 211.646789 | 171.2366864 | -0.30567 | 0.000425 | 0.000425 | Downregulated |
| GPR65 | 211.646789 | 171.2366864 | -0.30567 | 0.000425 | 0.000425 | Downregulated |
| MBTD1 | 211.6376147 | 171.2485207 | -0.3055 | 0.000428 | 0.000428 | Downregulated |
| MUSK | 125.8348624 | 101.8224852 | -0.30548 | 0.023883 | 0.023883 | Downregulated |
| TP53TG5 | 211.6238532 | 171.2662722 | -0.30526 | 0.000432 | 0.000432 | Downregulated |
| C2orf49 | 211.6192661 | 171.2721893 | -0.30518 | 0.000434 | 0.000434 | Downregulated |
| UVRAG | 211.5917431 | 171.3076923 | -0.30469 | 0.000443 | 0.000443 | Downregulated |
| MFSD8 | 211.5917431 | 171.3076923 | -0.30469 | 0.000443 | 0.000443 | Downregulated |
| CCDC36 | 191.4036697 | 154.964497 | -0.30468 | 0.001427 | 0.001427 | Downregulated |
| PELI2 | 211.5825688 | 171.3195266 | -0.30453 | 0.000446 | 0.000446 | Downregulated |
| TMEM132D | 123.2981651 | 99.84023669 | -0.30446 | 0.017619 | 0.017619 | Downregulated |
| TRPC1 | 211.5733945 | 171.3313609 | -0.30437 | 0.000449 | 0.000449 | Downregulated |
| AP002495.2 | 211.5688073 | 171.3372781 | -0.30429 | 0.00045 | 0.00045 | Downregulated |
| SIGLEC5 | 187.2568807 | 151.6627219 | -0.30415 | 0.00168 | 0.00168 | Downregulated |
| HCAR3 | 160.5779817 | 130.0650888 | -0.30404 | 0.004461 | 0.004461 | Downregulated |
| TCP10 | 136.9220183 | 110.9053254 | -0.30403 | 0.026351 | 0.026351 | Downregulated |
| TLR8 | 208.2798165 | 168.7455621 | -0.30367 | 0.000567 | 0.000567 | Downregulated |
| PLSCR4 | 211.5321101 | 171.3846154 | -0.30364 | 0.000463 | 0.000463 | Downregulated |
| ERCC3 | 211.5275229 | 171.3905325 | -0.30356 | 0.000464 | 0.000464 | Downregulated |
| NRXN2 | 211.5275229 | 171.3905325 | -0.30356 | 0.000464 | 0.000464 | Downregulated |
| FBXO33 | 211.5183486 | 171.4023669 | -0.3034 | 0.000468 | 0.000468 | Downregulated |
| DOC2A | 209.3440367 | 169.6449704 | -0.30336 | 0.000536 | 0.000536 | Downregulated |
| PHF5A | 211.5137615 | 171.408284 | -0.30331 | 0.000469 | 0.000469 | Downregulated |
| NIF3L1 | 211.5137615 | 171.408284 | -0.30331 | 0.000469 | 0.000469 | Downregulated |
| SS18L1 | 211.5091743 | 171.4142012 | -0.30323 | 0.000471 | 0.000471 | Downregulated |
| STX11 | 211.5091743 | 171.4142012 | -0.30323 | 0.000471 | 0.000471 | Downregulated |
| GUCY1B3 | 211.5045872 | 171.4201183 | -0.30315 | 0.000473 | 0.000473 | Downregulated |
| RAPGEF5 | 211.4954128 | 171.4319527 | -0.30299 | 0.000476 | 0.000476 | Downregulated |
| WIF1 | 127.4266055 | 103.3017751 | -0.3028 | 0.021474 | 0.021474 | Downregulated |
| GDAP2 | 211.4816514 | 171.4497041 | -0.30275 | 0.000481 | 0.000481 | Downregulated |
| HIST1H2AG | 211.4816514 | 171.4497041 | -0.30275 | 0.000481 | 0.000481 | Downregulated |
| BCL2L11 | 211.4770642 | 171.4556213 | -0.30267 | 0.000482 | 0.000482 | Downregulated |
| C3orf58 | 211.4724771 | 171.4615385 | -0.30258 | 0.000484 | 0.000484 | Downregulated |
| PRAC1 | 112.6834862 | 91.37869822 | -0.30235 | 0.03118 | 0.03118 | Downregulated |
| STEAP4 | 211.4357798 | 171.5088757 | -0.30194 | 0.000497 | 0.000497 | Downregulated |
| HIST1H4H | 210.3394495 | 170.6390533 | -0.30177 | 0.000534 | 0.000534 | Downregulated |
| REEP1 | 211.4174312 | 171.5325444 | -0.30161 | 0.000504 | 0.000504 | Downregulated |
| CRLF3 | 211.4036697 | 171.5502959 | -0.30137 | 0.00051 | 0.00051 | Downregulated |
| PPP2R3C | 211.3761468 | 171.5857988 | -0.30088 | 0.00052 | 0.00052 | Downregulated |
| CCDC138 | 211.3715596 | 171.591716 | -0.3008 | 0.000522 | 0.000522 | Downregulated |
| TP53INP1 | 211.3577982 | 171.6094675 | -0.30056 | 0.000527 | 0.000527 | Downregulated |
| FMO2 | 180.9541284 | 146.9940828 | -0.29987 | 0.002466 | 0.002466 | Downregulated |
| PAQR3 | 211.3119266 | 171.6686391 | -0.29975 | 0.000545 | 0.000545 | Downregulated |
| MMRN1 | 203.7522936 | 165.556213 | -0.2995 | 0.00087 | 0.00087 | Downregulated |
| RGS22 | 135.0091743 | 109.7100592 | -0.29936 | 0.017444 | 0.017444 | Downregulated |
| PGBD1 | 211.2844037 | 171.704142 | -0.29926 | 0.000557 | 0.000557 | Downregulated |
| LILRA5 | 211.2752294 | 171.7159763 | -0.2991 | 0.00056 | 0.00056 | Downregulated |
| KLHL11 | 211.2706422 | 171.7218935 | -0.29902 | 0.000562 | 0.000562 | Downregulated |
| RASGRP3 | 211.266055 | 171.7278107 | -0.29894 | 0.000564 | 0.000564 | Downregulated |
| RAB11FIP2 | 211.266055 | 171.7278107 | -0.29894 | 0.000564 | 0.000564 | Downregulated |
| FAM122C | 211.266055 | 171.7278107 | -0.29894 | 0.000564 | 0.000564 | Downregulated |
| TAF8 | 211.266055 | 171.7278107 | -0.29894 | 0.000564 | 0.000564 | Downregulated |
| AQP8 | 190.0733945 | 154.5088757 | -0.29887 | 0.001817 | 0.001817 | Downregulated |
| IL4I1 | 211.2614679 | 171.7337278 | -0.29886 | 0.000566 | 0.000566 | Downregulated |
[truncated: 305,558 more chars]
