# Supplementary material for: KRAS mutations are negatively correlated with immunity in colon cancer
Source: Aging (Albany NY). 2020 Nov 26;13(1):750–68. doi: 10.18632/aging.202182 (PMC7834984; doi:10.18632/aging.202182)
Supplement: Supplementary Table 7 [file aging-13-202182-s006.docx]

**Supplementary Table 7 The correlation coefficients of differential expressed genes and immune score.**

| Gene | cor | pvalue |
| --- | --- | --- |
| STX11 | 0.86 | 8.46E-115 |
| DOK2 | 0.857 | 4.57E-113 |
| TNFAIP8L2 | 0.855 | 4.32E-112 |
| LCP2 | 0.843 | 1.42E-105 |
| LILRB1 | 0.839 | 5.69E-104 |
| ABI3 | 0.838 | 2.08E-103 |
| LRRC25 | 0.825 | 1.44E-97 |
| SASH3 | 0.825 | 1.56E-97 |
| CD300A | 0.824 | 3.59E-97 |
| LAIR1 | 0.823 | 2.00E-96 |
| MS4A6A | 0.821 | 8.53E-96 |
| SCIMP | 0.82 | 2.04E-95 |
| APBB1IP | 0.817 | 5.57E-94 |
| HCLS1 | 0.816 | 1.00E-93 |
| C3AR1 | 0.814 | 6.91E-93 |
| IL2RA | 0.814 | 1.06E-92 |
| HAVCR2 | 0.813 | 1.22E-92 |
| CD300LF | 0.812 | 5.49E-92 |
| LST1 | 0.811 | 7.87E-92 |
| SLAMF8 | 0.809 | 8.01E-91 |
| C10orf128 | 0.809 | 8.84E-91 |
| CD86 | 0.808 | 1.23E-90 |
| IL10RA | 0.805 | 2.55E-89 |
| CYTH4 | 0.804 | 8.81E-89 |
| MYO1F | 0.8 | 2.42E-87 |
| HCK | 0.799 | 5.65E-87 |
| CMKLR1 | 0.798 | 7.82E-87 |
| MPEG1 | 0.798 | 1.63E-86 |
| GNGT2 | 0.797 | 2.94E-86 |
| CD48 | 0.797 | 3.74E-86 |
| SLA | 0.795 | 1.89E-85 |
| LILRB4 | 0.792 | 1.95E-84 |
| SIGLEC7 | 0.786 | 1.78E-82 |
| BTK | 0.785 | 3.67E-82 |
| NCKAP1L | 0.784 | 8.69E-82 |
| FGR | 0.784 | 1.13E-81 |
| LILRB2 | 0.784 | 1.25E-81 |
| MS4A4A | 0.781 | 9.90E-81 |
| GIMAP6 | 0.78 | 1.52E-80 |
| FGD2 | 0.778 | 6.91E-80 |
| CCR5 | 0.778 | 9.79E-80 |
| PLEKHO2 | 0.778 | 1.04E-79 |
| CCR1 | 0.777 | 1.98E-79 |
| SIGLEC10 | 0.777 | 3.12E-79 |
| NCF1 | 0.775 | 1.25E-78 |
| PILRA | 0.774 | 1.62E-78 |
| PIK3R5 | 0.772 | 1.20E-77 |
| GIMAP4 | 0.771 | 1.55E-77 |
| SIGLEC9 | 0.771 | 2.68E-77 |
| TNFRSF9 | 0.769 | 8.78E-77 |
| KCNK13 | 0.768 | 1.26E-76 |
| SLAMF7 | 0.768 | 1.83E-76 |
| CD96 | 0.768 | 2.04E-76 |
| CLEC7A | 0.768 | 2.31E-76 |
| SLAMF1 | 0.767 | 3.90E-76 |
| PDCD1LG2 | 0.766 | 6.69E-76 |
| EVI2B | 0.766 | 7.56E-76 |
| CD3G | 0.765 | 1.14E-75 |
| TAGAP | 0.758 | 2.39E-73 |
| FPR3 | 0.757 | 4.10E-73 |
| THEMIS2 | 0.756 | 6.67E-73 |
| IL21R | 0.754 | 3.66E-72 |
| CD247 | 0.753 | 4.09E-72 |
| RHOH | 0.751 | 1.61E-71 |
| DOCK2 | 0.751 | 2.84E-71 |
| ARHGAP15 | 0.75 | 5.68E-71 |
| IKZF1 | 0.748 | 1.27E-70 |
| PARVG | 0.747 | 3.11E-70 |
| CSF2RB | 0.746 | 6.75E-70 |
| IGSF6 | 0.745 | 9.51E-70 |
| FLI1 | 0.743 | 2.96E-69 |
| IL2RB | 0.743 | 4.81E-69 |
| FCGR1A | 0.74 | 3.37E-68 |
| JAML | 0.74 | 3.56E-68 |
| SAMSN1 | 0.739 | 6.41E-68 |
| P2RY10 | 0.738 | 7.09E-68 |
| EVI2A | 0.738 | 1.13E-67 |
| TNFSF13B | 0.737 | 1.59E-67 |
| CSF1 | 0.737 | 2.36E-67 |
| P2RY13 | 0.736 | 2.96E-67 |
| CXCR6 | 0.736 | 3.89E-67 |
| FYB | 0.735 | 5.67E-67 |
| SIRPB2 | 0.734 | 9.21E-67 |
| HVCN1 | 0.734 | 1.15E-66 |
| FAM49A | 0.733 | 2.24E-66 |
| ICOS | 0.732 | 3.97E-66 |
| PLA2G7 | 0.732 | 5.07E-66 |
| MNDA | 0.73 | 1.39E-65 |
| CHST2 | 0.729 | 1.77E-65 |
| SLA2 | 0.729 | 1.79E-65 |
| GPR65 | 0.729 | 2.11E-65 |
| C1orf162 | 0.726 | 1.54E-64 |
| MS4A7 | 0.725 | 2.89E-64 |
| GAB3 | 0.722 | 1.21E-63 |
| DPEP2 | 0.722 | 1.64E-63 |
| VSIG4 | 0.721 | 2.01E-63 |
| LY96 | 0.719 | 7.35E-63 |
| MILR1 | 0.719 | 9.37E-63 |
| PLEK | 0.718 | 1.29E-62 |
| TM6SF1 | 0.718 | 1.41E-62 |
| ITGAX | 0.718 | 1.79E-62 |
| CXorf21 | 0.718 | 1.88E-62 |
| CASS4 | 0.717 | 3.34E-62 |
| CD226 | 0.716 | 5.99E-62 |
| TIGIT | 0.715 | 7.47E-62 |
| CD209 | 0.713 | 2.35E-61 |
| CD180 | 0.712 | 4.47E-61 |
| FCGR2B | 0.712 | 5.32E-61 |
| PTPRC | 0.711 | 7.32E-61 |
| SH2D1A | 0.711 | 8.32E-61 |
| ARHGEF6 | 0.71 | 1.00E-60 |
| SLC31A2 | 0.707 | 5.72E-60 |
| LILRA2 | 0.707 | 5.95E-60 |
| RCSD1 | 0.706 | 1.54E-59 |
| RASSF5 | 0.705 | 1.68E-59 |
| CD84 | 0.704 | 2.93E-59 |
| FAM20A | 0.702 | 8.90E-59 |
| KLHL6 | 0.702 | 9.66E-59 |
| PPM1M | 0.701 | 2.10E-58 |
| STAT4 | 0.7 | 3.48E-58 |
| CCR2 | 0.699 | 4.61E-58 |
| ADGRE2 | 0.699 | 4.72E-58 |
| LILRA1 | 0.699 | 5.72E-58 |
| GPR183 | 0.698 | 1.07E-57 |
| FOXP3 | 0.697 | 1.37E-57 |
| GIMAP8 | 0.697 | 1.48E-57 |
| FGL2 | 0.696 | 2.98E-57 |
| SIGLEC14 | 0.695 | 3.16E-57 |
| NCF2 | 0.695 | 4.26E-57 |
| TIFAB | 0.693 | 1.17E-56 |
| ADA2 | 0.693 | 1.41E-56 |
| TLR8 | 0.691 | 3.34E-56 |
| BIN2 | 0.691 | 4.10E-56 |
| PPP1R16B | 0.69 | 5.84E-56 |
| CLEC4A | 0.689 | 9.56E-56 |
| JAK3 | 0.689 | 1.06E-55 |
| GPR141 | 0.688 | 1.41E-55 |
| FCGR2A | 0.688 | 1.77E-55 |
| RASGRP4 | 0.687 | 1.95E-55 |
| CYSLTR1 | 0.686 | 4.15E-55 |
| RASSF2 | 0.685 | 5.55E-55 |
| TLR7 | 0.684 | 8.50E-55 |
| MAF | 0.684 | 9.46E-55 |
| FAM26F | 0.684 | 1.16E-54 |
| LTA | 0.683 | 1.66E-54 |
| CFP | 0.681 | 3.81E-54 |
| RASSF4 | 0.681 | 3.91E-54 |
| GPR84 | 0.679 | 1.02E-53 |
| SFMBT2 | 0.678 | 1.65E-53 |
| PLEKHO1 | 0.678 | 1.67E-53 |
| IL4I1 | 0.677 | 3.85E-53 |
| CD28 | 0.676 | 4.36E-53 |
| DOCK10 | 0.676 | 5.14E-53 |
| LILRB3 | 0.675 | 1.07E-52 |
| THEMIS | 0.674 | 1.12E-52 |
| GIMAP7 | 0.673 | 1.79E-52 |
| RASGRP1 | 0.673 | 2.45E-52 |
| EBI3 | 0.672 | 4.54E-52 |
| PLXNC1 | 0.671 | 4.61E-52 |
| SLC9A9 | 0.668 | 2.13E-51 |
| KLRD1 | 0.667 | 3.12E-51 |
| TMIGD3 | 0.667 | 3.67E-51 |
| CTLA4 | 0.666 | 6.02E-51 |
| TMEM86A | 0.665 | 8.80E-51 |
| P2RX7 | 0.664 | 1.25E-50 |
| LRRC8C | 0.663 | 2.88E-50 |
| PREX1 | 0.662 | 3.02E-50 |
| GPR171 | 0.662 | 3.94E-50 |
| ABCD2 | 0.66 | 1.04E-49 |
| RAB33A | 0.657 | 3.10E-49 |
| LILRB5 | 0.657 | 4.32E-49 |
| CCR4 | 0.656 | 4.56E-49 |
| SIGLEC5 | 0.656 | 6.61E-49 |
| TFEC | 0.655 | 7.09E-49 |
| TRAF3IP3 | 0.654 | 1.30E-48 |
| RGS18 | 0.654 | 1.48E-48 |
| PYHIN1 | 0.654 | 1.64E-48 |
| NLRC4 | 0.652 | 3.33E-48 |
| GNG2 | 0.651 | 4.21E-48 |
| CYSLTR2 | 0.649 | 1.00E-47 |
| GPR34 | 0.649 | 1.27E-47 |
| PDE6G | 0.647 | 2.56E-47 |
| CLEC4E | 0.647 | 2.74E-47 |
| CHST11 | 0.647 | 3.02E-47 |
| JAKMIP1 | 0.647 | 3.13E-47 |
| KMO | 0.646 | 3.80E-47 |
| DPYD | 0.646 | 4.31E-47 |
| CD80 | 0.645 | 5.92E-47 |
| MRAS | 0.645 | 7.04E-47 |
| ST8SIA4 | 0.645 | 7.85E-47 |
| IFI30 | 0.641 | 4.36E-46 |
| CLEC10A | 0.64 | 6.47E-46 |
| SELL | 0.639 | 8.39E-46 |
| PIK3CG | 0.638 | 1.19E-45 |
| CLIC2 | 0.637 | 1.85E-45 |
| PRKCB | 0.637 | 2.36E-45 |
| LILRA6 | 0.636 | 2.59E-45 |
| MYO5A | 0.635 | 4.39E-45 |
| CD38 | 0.634 | 7.54E-45 |
| ITGA4 | 0.632 | 1.72E-44 |
| P2RY6 | 0.632 | 1.78E-44 |
| VCAM1 | 0.632 | 1.82E-44 |
| CD83 | 0.631 | 2.39E-44 |
| ZEB2 | 0.63 | 3.20E-44 |
| LILRA5 | 0.63 | 3.54E-44 |
| SIRPB1 | 0.628 | 6.95E-44 |
| TLR1 | 0.628 | 8.63E-44 |
| IKZF3 | 0.627 | 9.54E-44 |
| ITK | 0.627 | 1.02E-43 |
| CD300LB | 0.627 | 1.26E-43 |
| APOBEC3G | 0.626 | 1.59E-43 |
| BASP1 | 0.624 | 4.25E-43 |
| CCR8 | 0.622 | 8.49E-43 |
| MSR1 | 0.622 | 9.06E-43 |
| SLC1A3 | 0.622 | 9.54E-43 |
| IGFLR1 | 0.621 | 1.06E-42 |
| CAMK1G | 0.621 | 1.37E-42 |
| GIMAP5 | 0.621 | 1.41E-42 |
| GAPT | 0.62 | 1.57E-42 |
| PRF1 | 0.619 | 2.77E-42 |
| GZMK | 0.619 | 3.16E-42 |
| DRAM1 | 0.618 | 3.41E-42 |
| FCN1 | 0.618 | 4.26E-42 |
| GNB4 | 0.616 | 7.01E-42 |
| NRP1 | 0.614 | 1.73E-41 |
| ZIK1 | 0.614 | 1.85E-41 |
| TNFRSF4 | 0.614 | 1.95E-41 |
| DNAJC5B | 0.613 | 2.36E-41 |
| CCL2 | 0.613 | 3.00E-41 |
| CNRIP1 | 0.611 | 5.97E-41 |
| IL18RAP | 0.611 | 6.14E-41 |
| CEACAM4 | 0.61 | 7.39E-41 |
| SOCS3 | 0.607 | 2.70E-40 |
| LAX1 | 0.607 | 2.76E-40 |
| MEI1 | 0.604 | 6.39E-40 |
| CLEC6A | 0.602 | 1.64E-39 |
| SIGLEC8 | 0.599 | 5.60E-39 |
| DSE | 0.598 | 6.02E-39 |
| CXCL13 | 0.598 | 6.06E-39 |
| NFATC1 | 0.598 | 6.11E-39 |
| CLEC1A | 0.597 | 9.38E-39 |
| ANKRD44 | 0.595 | 2.08E-38 |
| RASGRP3 | 0.595 | 2.22E-38 |
| MRC1 | 0.594 | 2.47E-38 |
| SYT11 | 0.594 | 3.12E-38 |
| CFH | 0.592 | 5.59E-38 |
| NXPE3 | 0.592 | 6.38E-38 |
| THBD | 0.591 | 7.54E-38 |
| TMEM156 | 0.59 | 1.09E-37 |
| CD163 | 0.589 | 1.74E-37 |
| KLRG1 | 0.589 | 1.77E-37 |
| IRF4 | 0.583 | 1.41E-36 |
| RNASE2 | 0.583 | 1.44E-36 |
| NR3C1 | 0.582 | 1.81E-36 |
| RAB8B | 0.581 | 2.96E-36 |
| WISP1 | 0.58 | 3.53E-36 |
| FPR1 | 0.579 | 4.59E-36 |
| CHST15 | 0.578 | 7.63E-36 |
| MS4A14 | 0.577 | 8.81E-36 |
| SLCO2B1 | 0.577 | 9.92E-36 |
| GPR176 | 0.575 | 2.00E-35 |
| CYLD | 0.574 | 2.57E-35 |
| TNFRSF18 | 0.571 | 8.53E-35 |
| KLRB1 | 0.57 | 1.03E-34 |
| QKI | 0.568 | 2.17E-34 |
| DOK5 | 0.567 | 2.86E-34 |
| IL1R1 | 0.567 | 2.90E-34 |
| ENTPD1 | 0.567 | 3.03E-34 |
| PRR16 | 0.566 | 4.04E-34 |
| OLFML1 | 0.566 | 4.24E-34 |
| CSF2RA | 0.564 | 6.74E-34 |
| ICAM3 | 0.563 | 8.29E-34 |
| CSF3R | 0.563 | 8.73E-34 |
| TMEM26 | 0.563 | 1.09E-33 |
| IFNG | 0.562 | 1.14E-33 |
| RFLNB | 0.561 | 1.99E-33 |
| CLIP4 | 0.559 | 3.39E-33 |
| TLR2 | 0.556 | 9.61E-33 |
| FLVCR2 | 0.554 | 1.64E-32 |
| S1PR1 | 0.552 | 3.00E-32 |
| ADAMTS2 | 0.55 | 5.28E-32 |
| OSMR | 0.549 | 6.69E-32 |
| TWIST2 | 0.549 | 8.07E-32 |
| ZNF831 | 0.548 | 9.32E-32 |
| IL7R | 0.548 | 9.87E-32 |
| CIITA | 0.548 | 1.02E-31 |
| P2RY12 | 0.548 | 1.19E-31 |
| ATP8B4 | 0.547 | 1.36E-31 |
| MDGA1 | 0.546 | 1.73E-31 |
| GPC6 | 0.546 | 1.73E-31 |
| HTRA4 | 0.546 | 1.84E-31 |
| GFPT2 | 0.546 | 1.88E-31 |
| SLFN12L | 0.545 | 2.19E-31 |
| SYNE3 | 0.545 | 2.71E-31 |
| TNFAIP3 | 0.544 | 3.94E-31 |
| S100B | 0.543 | 4.51E-31 |
| CLMP | 0.543 | 4.54E-31 |
| KCNJ8 | 0.543 | 4.75E-31 |
| KCNN3 | 0.543 | 5.09E-31 |
| ST6GALNAC5 | 0.542 | 6.68E-31 |
| TREM1 | 0.539 | 1.61E-30 |
| BTLA | 0.538 | 2.24E-30 |
| CEACAM21 | 0.537 | 2.96E-30 |
| SLC7A7 | 0.536 | 3.78E-30 |
| CLEC2B | 0.535 | 4.96E-30 |
| BHLHE22 | 0.534 | 5.82E-30 |
| LAYN | 0.534 | 7.39E-30 |
| F13A1 | 0.534 | 7.43E-30 |
| UBE2E2 | 0.533 | 8.12E-30 |
| ADGRE3 | 0.532 | 1.25E-29 |
| ZNF366 | 0.531 | 1.63E-29 |
| CRISPLD2 | 0.53 | 2.21E-29 |
| CCDC88A | 0.528 | 3.21E-29 |
| BCL2A1 | 0.528 | 3.41E-29 |
| ADGRG3 | 0.528 | 4.12E-29 |
| HLA-DQA2 | 0.528 | 4.17E-29 |
| OLR1 | 0.527 | 4.59E-29 |
| GNLY | 0.527 | 4.89E-29 |
| ST3GAL6 | 0.527 | 5.28E-29 |
| CLEC5A | 0.527 | 5.39E-29 |
| CR1 | 0.526 | 6.23E-29 |
| PLA2G2D | 0.524 | 1.05E-28 |
| MEOX1 | 0.522 | 2.15E-28 |
| VEGFC | 0.521 | 2.81E-28 |
| HSD11B1 | 0.52 | 3.01E-28 |
| DOCK4 | 0.52 | 3.04E-28 |
| BTN2A2 | 0.519 | 4.57E-28 |
| SHE | 0.519 | 5.06E-28 |
| STK10 | 0.518 | 5.61E-28 |
| MEFV | 0.518 | 5.69E-28 |
| BCL6B | 0.518 | 6.73E-28 |
| SUCNR1 | 0.517 | 7.70E-28 |
| VWF | 0.516 | 9.41E-28 |
| 4-Sep | 0.516 | 1.07E-27 |
| SLFN11 | 0.515 | 1.31E-27 |
| SNAI2 | 0.515 | 1.42E-27 |
| SERPINB9 | 0.515 | 1.44E-27 |
| FAM26E | 0.514 | 1.70E-27 |
| ZNF521 | 0.512 | 3.38E-27 |
| GNG11 | 0.512 | 3.38E-27 |
| GLT1D1 | 0.511 | 3.70E-27 |
| MOXD1 | 0.511 | 4.12E-27 |
| FCRL5 | 0.511 | 4.35E-27 |
| APOBEC3A | 0.51 | 5.39E-27 |
| CYTL1 | 0.508 | 9.51E-27 |
| ADGRE1 | 0.506 | 1.38E-26 |
| PRKAR2B | 0.506 | 1.40E-26 |
| TEK | 0.506 | 1.55E-26 |
| ADGRL4 | 0.504 | 2.56E-26 |
| CYYR1 | 0.503 | 2.98E-26 |
| SLC47A1 | 0.503 | 3.02E-26 |
| FCGR3B | 0.503 | 3.48E-26 |
| DYSF | 0.502 | 4.71E-26 |
| CD274 | 0.501 | 5.82E-26 |
| HACD4 | 0.501 | 5.90E-26 |
| GBP4 | 0.5 | 6.47E-26 |
| TTYH2 | 0.5 | 7.74E-26 |
| TNF | 0.5 | 8.07E-26 |
| CHI3L2 | 0.499 | 9.79E-26 |
| YPEL4 | 0.499 | 1.04E-25 |
| KLHDC7B | 0.498 | 1.10E-25 |
| CD40 | 0.497 | 1.37E-25 |
| EHD3 | 0.497 | 1.49E-25 |
| IL18R1 | 0.497 | 1.56E-25 |
| C20orf194 | 0.495 | 2.31E-25 |
| CPA3 | 0.494 | 3.17E-25 |
| TMEM255A | 0.494 | 3.21E-25 |
| CREM | 0.494 | 3.38E-25 |
| CCDC80 | 0.493 | 4.11E-25 |
| NTNG2 | 0.493 | 4.67E-25 |
| MMP25 | 0.492 | 4.88E-25 |
| ADAMTS1 | 0.492 | 4.96E-25 |
| FGF7 | 0.492 | 5.15E-25 |
| KLHL5 | 0.491 | 6.78E-25 |
| PINLYP | 0.491 | 6.99E-25 |
| LITAF | 0.491 | 7.32E-25 |
| TCF4 | 0.491 | 7.35E-25 |
| SELP | 0.491 | 7.65E-25 |
| SV2B | 0.491 | 7.99E-25 |
| CYP7B1 | 0.49 | 8.44E-25 |
| FMO1 | 0.489 | 1.23E-24 |
| SNX10 | 0.488 | 1.70E-24 |
| CALCRL | 0.487 | 1.81E-24 |
| GUCY1B3 | 0.486 | 2.49E-24 |
| ZBTB46 | 0.486 | 2.79E-24 |
| DZIP1 | 0.485 | 2.86E-24 |
| ECSCR | 0.485 | 2.91E-24 |
| CERKL | 0.485 | 3.31E-24 |
| IL5RA | 0.485 | 3.45E-24 |
| LOX | 0.485 | 3.53E-24 |
| LOXL3 | 0.484 | 3.64E-24 |
| SLC2A3 | 0.484 | 3.91E-24 |
| MITF | 0.484 | 4.01E-24 |
| NFKBIA | 0.484 | 4.30E-24 |
| FEZ1 | 0.484 | 4.49E-24 |
| DLC1 | 0.483 | 5.11E-24 |
| CPXM1 | 0.483 | 5.27E-24 |
| DZIP1L | 0.482 | 7.20E-24 |
| LIX1L | 0.481 | 7.62E-24 |
| IFNAR2 | 0.481 | 8.18E-24 |
| CD300E | 0.481 | 8.34E-24 |
| PDE1A | 0.481 | 8.76E-24 |
| FAP | 0.481 | 8.85E-24 |
| CHN1 | 0.481 | 8.98E-24 |
| ERG | 0.481 | 9.31E-24 |
| KIR2DL4 | 0.48 | 9.94E-24 |
| IL6R | 0.48 | 1.06E-23 |
| AFAP1L1 | 0.479 | 1.49E-23 |
| GLT8D2 | 0.478 | 1.72E-23 |
| AEBP1 | 0.477 | 2.12E-23 |
| FUT11 | 0.476 | 2.55E-23 |
| ADGRL2 | 0.476 | 2.69E-23 |
| DSEL | 0.476 | 2.74E-23 |
| KCNJ15 | 0.476 | 2.89E-23 |
| THBS2 | 0.475 | 3.30E-23 |
| VSTM1 | 0.475 | 3.42E-23 |
| SIRPA | 0.475 | 3.66E-23 |
| FAM110D | 0.475 | 3.77E-23 |
| DCLK1 | 0.474 | 4.10E-23 |
| TSPYL5 | 0.474 | 4.13E-23 |
| SLC2A6 | 0.474 | 4.87E-23 |
| IKBIP | 0.474 | 4.90E-23 |
| CTNS | 0.472 | 8.04E-23 |
| CCL21 | 0.47 | 1.05E-22 |
| PKD2 | 0.469 | 1.41E-22 |
| ZNF671 | 0.469 | 1.45E-22 |
| CLEC12A | 0.469 | 1.57E-22 |
| TNFAIP6 | 0.467 | 2.46E-22 |
| PCDH17 | 0.466 | 2.75E-22 |
| TNFRSF17 | 0.465 | 3.60E-22 |
| WDR7 | 0.465 | 3.94E-22 |
| ABCA6 | 0.464 | 4.24E-22 |
| NOX4 | 0.464 | 4.71E-22 |
| FCHSD2 | 0.464 | 4.90E-22 |
| JAM2 | 0.462 | 7.29E-22 |
| IL17RA | 0.462 | 7.89E-22 |
| BCAT1 | 0.461 | 8.85E-22 |
| TRPC6 | 0.461 | 9.42E-22 |
| SPG20 | 0.461 | 9.57E-22 |
| PABPC5 | 0.461 | 9.58E-22 |
| BICC1 | 0.46 | 1.13E-21 |
| CXorf36 | 0.46 | 1.18E-21 |
| HLA-DOA | 0.459 | 1.33E-21 |
| ACOD1 | 0.459 | 1.58E-21 |
| MYCT1 | 0.458 | 1.70E-21 |
| GAS1 | 0.457 | 2.34E-21 |
| LDB2 | 0.457 | 2.38E-21 |
| ECM2 | 0.457 | 2.39E-21 |
| PTGFR | 0.457 | 2.44E-21 |
| P2RX1 | 0.456 | 2.78E-21 |
| TBC1D9 | 0.456 | 3.02E-21 |
| CCL17 | 0.455 | 3.25E-21 |
| CFAP54 | 0.455 | 3.56E-21 |
| ARNTL | 0.455 | 3.62E-21 |
| TCTEX1D1 | 0.455 | 3.82E-21 |
| FZD4 | 0.455 | 3.82E-21 |
| GPR4 | 0.454 | 4.31E-21 |
| TP53INP1 | 0.454 | 4.38E-21 |
| AKT3 | 0.454 | 4.81E-21 |
| UBASH3B | 0.453 | 5.69E-21 |
| PAMR1 | 0.452 | 6.60E-21 |
| ADGRF5 | 0.452 | 6.87E-21 |
| AQP9 | 0.452 | 7.04E-21 |
| CEP85L | 0.449 | 1.27E-20 |
| NXN | 0.449 | 1.28E-20 |
| BATF3 | 0.448 | 1.57E-20 |
| KIAA1462 | 0.448 | 1.70E-20 |
| IL21 | 0.448 | 1.70E-20 |
| SGTB | 0.447 | 1.89E-20 |
| JAZF1 | 0.447 | 1.93E-20 |
| LPAR4 | 0.447 | 2.17E-20 |
| MEF2C | 0.446 | 2.50E-20 |
| GBP5 | 0.446 | 2.75E-20 |
| CCDC102B | 0.445 | 2.96E-20 |
| TBXA2R | 0.445 | 3.54E-20 |
| FMO3 | 0.444 | 3.62E-20 |
| ITPR1 | 0.444 | 3.72E-20 |
| ADAMTS3 | 0.443 | 4.51E-20 |
| PTX3 | 0.443 | 4.87E-20 |
| ZNF677 | 0.442 | 6.48E-20 |
| VGLL3 | 0.44 | 8.45E-20 |
| GNS | 0.439 | 1.05E-19 |
| PALMD | 0.439 | 1.19E-19 |
| ADAMTS12 | 0.438 | 1.32E-19 |
| CSRP2 | 0.438 | 1.36E-19 |
| BAALC | 0.437 | 1.62E-19 |
| LRRK2 | 0.436 | 2.02E-19 |
| LYVE1 | 0.436 | 2.06E-19 |
| HTR2A | 0.436 | 2.26E-19 |
| DCSTAMP | 0.435 | 2.43E-19 |
| RIPOR3 | 0.435 | 2.49E-19 |
| FCAR | 0.435 | 2.51E-19 |
| F2RL3 | 0.434 | 3.55E-19 |
| FCMR | 0.433 | 4.45E-19 |
| RGL1 | 0.433 | 4.45E-19 |
| PPP2R2B | 0.432 | 4.68E-19 |
| GNB5 | 0.432 | 4.86E-19 |
| GUCY1A3 | 0.431 | 5.56E-19 |
| FRMD6 | 0.431 | 5.80E-19 |
| CD244 | 0.431 | 6.04E-19 |
| DAZL | 0.431 | 6.27E-19 |
| VNN2 | 0.43 | 7.13E-19 |
| PDGFRA | 0.43 | 7.93E-19 |
| HOPX | 0.429 | 1.01E-18 |
| IQSEC3 | 0.428 | 1.12E-18 |
| ENOX1 | 0.428 | 1.14E-18 |
| PREX2 | 0.428 | 1.21E-18 |
| SNRK | 0.427 | 1.39E-18 |
| CD1B | 0.426 | 1.85E-18 |
| COL5A2 | 0.425 | 1.98E-18 |
| MCEMP1 | 0.425 | 2.24E-18 |
| HTR7 | 0.425 | 2.26E-18 |
| EGFLAM | 0.424 | 2.46E-18 |
| TGFB3 | 0.423 | 3.05E-18 |
| SP100 | 0.423 | 3.17E-18 |
| MS4A2 | 0.423 | 3.34E-18 |
| BEST1 | 0.422 | 3.61E-18 |
| RNF166 | 0.422 | 3.74E-18 |
| HHEX | 0.422 | 3.76E-18 |
| ABCC9 | 0.422 | 3.88E-18 |
| RAB3IL1 | 0.421 | 4.24E-18 |
| MCTP1 | 0.421 | 4.59E-18 |
| FHL5 | 0.418 | 8.14E-18 |
| SGIP1 | 0.418 | 8.48E-18 |
| C1orf216 | 0.418 | 9.25E-18 |
| TENM3 | 0.418 | 9.26E-18 |
| ANKRD33B | 0.417 | 9.64E-18 |
| ELOVL4 | 0.416 | 1.28E-17 |
| HPSE | 0.416 | 1.30E-17 |
| KDR | 0.415 | 1.60E-17 |
| MAP3K8 | 0.413 | 2.19E-17 |
| FCRL2 | 0.411 | 3.03E-17 |
| IRAK3 | 0.411 | 3.25E-17 |
| DENND5A | 0.411 | 3.49E-17 |
| FGF10 | 0.411 | 3.58E-17 |
| INMT | 0.41 | 4.42E-17 |
| BCL2L11 | 0.409 | 5.15E-17 |
| FPR2 | 0.409 | 5.34E-17 |
| STAT3 | 0.408 | 5.43E-17 |
| AVPR1A | 0.408 | 5.51E-17 |
| CLEC4D | 0.408 | 6.42E-17 |
| MFAP5 | 0.406 | 8.27E-17 |
| ARMCX1 | 0.403 | 1.44E-16 |
| MAN1C1 | 0.403 | 1.65E-16 |
| S1PR3 | 0.402 | 1.78E-16 |
| EBF1 | 0.402 | 1.78E-16 |
| CEP170 | 0.402 | 1.80E-16 |
| STEAP1B | 0.401 | 2.37E-16 |
| TIMP3 | 0.4 | 2.46E-16 |
| TNFSF10 | 0.399 | 3.21E-16 |
| RTP4 | 0.399 | 3.26E-16 |
| RAI14 | 0.399 | 3.38E-16 |
| STC1 | 0.399 | 3.43E-16 |
| FERMT2 | 0.398 | 3.73E-16 |
| SMARCD3 | 0.398 | 3.94E-16 |
| TSHZ2 | 0.398 | 4.00E-16 |
| TNFRSF1A | 0.398 | 4.06E-16 |
| HERPUD1 | 0.397 | 4.35E-16 |
| NOCT | 0.397 | 4.63E-16 |
| SGCE | 0.397 | 4.73E-16 |
| LLGL1 | 0.397 | 4.87E-16 |
| FAM46C | 0.396 | 5.90E-16 |
| TSPAN11 | 0.395 | 6.27E-16 |
| CCDC36 | 0.395 | 6.31E-16 |
| UNC5C | 0.395 | 7.16E-16 |
| SRGAP2C | 0.394 | 7.33E-16 |
| MIXL1 | 0.394 | 8.22E-16 |
| RORA | 0.393 | 9.01E-16 |
| IFIT2 | 0.393 | 9.37E-16 |
| LIFR | 0.391 | 1.48E-15 |
| TOGARAM2 | 0.39 | 1.55E-15 |
| C18orf8 | 0.39 | 1.69E-15 |
| GCSAML | 0.39 | 1.75E-15 |
| ZNF804A | 0.389 | 1.92E-15 |
| LRRN4CL | 0.389 | 2.13E-15 |
| FCN3 | 0.389 | 2.15E-15 |
| PATL2 | 0.388 | 2.33E-15 |
| CACNA2D1 | 0.388 | 2.44E-15 |
| HEXA | 0.388 | 2.48E-15 |
| DNAJB4 | 0.387 | 2.74E-15 |
| TCEAL7 | 0.385 | 3.73E-15 |
| ZNF626 | 0.385 | 4.10E-15 |
| ADAM23 | 0.384 | 4.63E-15 |
| RBMS3 | 0.383 | 5.46E-15 |
| ENPP2 | 0.383 | 5.62E-15 |
| STEAP4 | 0.383 | 6.12E-15 |
| PABPC4L | 0.381 | 7.45E-15 |
| ABCA1 | 0.381 | 7.71E-15 |
| DYRK3 | 0.38 | 9.26E-15 |
| EMCN | 0.38 | 1.03E-14 |
| WWTR1 | 0.379 | 1.19E-14 |
| DTHD1 | 0.379 | 1.25E-14 |
| LRFN5 | 0.378 | 1.45E-14 |
| OAS3 | 0.378 | 1.46E-14 |
| SRGAP2B | 0.377 | 1.71E-14 |
| TCP11L1 | 0.376 | 1.78E-14 |
| RELL1 | 0.376 | 1.78E-14 |
| MICU3 | 0.376 | 1.92E-14 |
| MBP | 0.376 | 2.06E-14 |
| SELE | 0.375 | 2.15E-14 |
| PDGFC | 0.374 | 2.84E-14 |
| SLC12A6 | 0.374 | 2.89E-14 |
| PLSCR4 | 0.373 | 3.48E-14 |
| TPP1 | 0.372 | 3.96E-14 |
| CNR2 | 0.372 | 4.06E-14 |
| ATG7 | 0.371 | 4.30E-14 |
| CPNE8 | 0.371 | 4.60E-14 |
| AAED1 | 0.37 | 5.15E-14 |
| CYP4F22 | 0.37 | 5.26E-14 |
| ZEB1 | 0.37 | 5.40E-14 |
| EDIL3 | 0.369 | 6.03E-14 |
| KCTD12 | 0.369 | 6.19E-14 |
| RAB35 | 0.369 | 6.23E-14 |
| PAPLN | 0.369 | 6.52E-14 |
| TRPC3 | 0.369 | 6.52E-14 |
| ADAMTS5 | 0.369 | 6.70E-14 |
| RARB | 0.368 | 7.46E-14 |
| CXCR1 | 0.367 | 8.51E-14 |
| ACSM5 | 0.367 | 8.98E-14 |
| GSDMC | 0.366 | 1.06E-13 |
| CIDEB | 0.366 | 1.07E-13 |
| BANK1 | 0.365 | 1.14E-13 |
| CD59 | 0.365 | 1.20E-13 |
| CLC | 0.365 | 1.27E-13 |
| KLHL4 | 0.365 | 1.30E-13 |
| ADGRD1 | 0.365 | 1.32E-13 |
| FGD5 | 0.364 | 1.37E-13 |
| RCAN1 | 0.364 | 1.43E-13 |
| CCDC178 | 0.363 | 1.81E-13 |
| SPARCL1 | 0.362 | 2.13E-13 |
| DIO2 | 0.362 | 2.14E-13 |
| C16orf86 | 0.362 | 2.15E-13 |
| AMZ1 | 0.361 | 2.29E-13 |
| CEACAM3 | 0.361 | 2.31E-13 |
| PDE6B | 0.361 | 2.33E-13 |
| KCNT2 | 0.36 | 2.70E-13 |
| CALU | 0.36 | 2.92E-13 |
| OSTM1 | 0.359 | 3.11E-13 |
| NR4A3 | 0.359 | 3.13E-13 |
| GPX7 | 0.359 | 3.22E-13 |
| KCNE1 | 0.358 | 3.77E-13 |
| ITGB3 | 0.358 | 3.93E-13 |
| KCNK12 | 0.358 | 4.02E-13 |
| MEIS1 | 0.357 | 4.30E-13 |
| LYSMD2 | 0.357 | 4.37E-13 |
| SAMD4A | 0.357 | 4.59E-13 |
| ZNF304 | 0.357 | 4.80E-13 |
| RAP1B | 0.355 | 6.39E-13 |
| DCBLD1 | 0.354 | 6.95E-13 |
| HCAR2 | 0.354 | 7.80E-13 |
| HTRA3 | 0.353 | 7.83E-13 |
| FLT1 | 0.353 | 7.89E-13 |
| RECQL | 0.351 | 1.10E-12 |
| PNRC1 | 0.35 | 1.27E-12 |
| HGF | 0.35 | 1.35E-12 |
| ARHGAP10 | 0.35 | 1.39E-12 |
| CASC10 | 0.348 | 1.82E-12 |
| PDLIM3 | 0.348 | 1.85E-12 |
| FAM171B | 0.348 | 1.87E-12 |
| NKAPL | 0.348 | 1.90E-12 |
| IL6 | 0.348 | 1.92E-12 |
| GYPE | 0.348 | 1.98E-12 |
| EPHA3 | 0.346 | 2.42E-12 |
| HCAR3 | 0.346 | 2.44E-12 |
| ANXA6 | 0.346 | 2.48E-12 |
| LURAP1 | 0.346 | 2.48E-12 |
| CCR3 | 0.346 | 2.72E-12 |
| FOXO1 | 0.346 | 2.72E-12 |
| VNN3 | 0.344 | 3.25E-12 |
| TCF21 | 0.344 | 3.25E-12 |
| GRIN3A | 0.344 | 3.34E-12 |
| PHTF2 | 0.343 | 3.83E-12 |
| SFRP1 | 0.342 | 4.72E-12 |
| SUSD2 | 0.342 | 4.78E-12 |
| SYNJ1 | 0.342 | 4.85E-12 |
| AOX1 | 0.342 | 4.97E-12 |
| TWIST1 | 0.341 | 5.06E-12 |
| EID3 | 0.341 | 5.21E-12 |
| P2RY14 | 0.341 | 5.23E-12 |
| CPXM2 | 0.341 | 5.55E-12 |
| EGFL6 | 0.34 | 5.98E-12 |
| FAM19A1 | 0.34 | 6.50E-12 |
| IL24 | 0.339 | 6.88E-12 |
| HPS5 | 0.339 | 7.48E-12 |
| IL32 | 0.339 | 7.76E-12 |
| PAPPA | 0.338 | 8.01E-12 |
| ELOVL3 | 0.338 | 8.29E-12 |
| GPR88 | 0.337 | 9.33E-12 |
| TAL1 | 0.337 | 9.45E-12 |
| FLCN | 0.337 | 9.51E-12 |
| ITPRIPL1 | 0.337 | 1.00E-11 |
| PBX3 | 0.337 | 1.00E-11 |
| PIK3C3 | 0.337 | 1.01E-11 |
| FAM126A | 0.337 | 1.03E-11 |
| LIPN | 0.335 | 1.24E-11 |
| TLE4 | 0.335 | 1.25E-11 |
| ZNF436 | 0.334 | 1.50E-11 |
| AJAP1 | 0.334 | 1.52E-11 |
| BIRC3 | 0.334 | 1.57E-11 |
| TMEM200B | 0.333 | 1.72E-11 |
| DPY19L2 | 0.333 | 1.88E-11 |
| TMX3 | 0.333 | 1.90E-11 |
| ESR2 | 0.332 | 1.94E-11 |
| BATF2 | 0.332 | 1.95E-11 |
| AKNA | 0.332 | 2.08E-11 |
| FAM162B | 0.332 | 2.10E-11 |
| SMAD4 | 0.332 | 2.15E-11 |
| ZNF835 | 0.332 | 2.20E-11 |
| SLC38A6 | 0.332 | 2.20E-11 |
| ZNF454 | 0.33 | 2.62E-11 |
| COL13A1 | 0.329 | 3.10E-11 |
| LHX6 | 0.329 | 3.18E-11 |
| ICAM2 | 0.329 | 3.25E-11 |
| C8orf48 | 0.328 | 3.51E-11 |
| RERGL | 0.327 | 4.48E-11 |
| SGPL1 | 0.327 | 4.55E-11 |
| APBB1 | 0.326 | 5.09E-11 |
| KRT222 | 0.324 | 6.61E-11 |
| EID2B | 0.324 | 6.79E-11 |
| PCSK5 | 0.323 | 7.38E-11 |
| NIN | 0.323 | 7.45E-11 |
| NLGN4X | 0.323 | 7.52E-11 |
| IRF2 | 0.323 | 7.67E-11 |
| DOK6 | 0.322 | 8.32E-11 |
| ZNF781 | 0.322 | 8.39E-11 |
| MSRB3 | 0.322 | 9.25E-11 |
| PROK2 | 0.321 | 9.46E-11 |
| FMO2 | 0.321 | 1.04E-10 |
| MXRA7 | 0.321 | 1.05E-10 |
| HERC5 | 0.32 | 1.13E-10 |
| ENPEP | 0.32 | 1.13E-10 |
| CDKN2B | 0.319 | 1.30E-10 |
| CD160 | 0.318 | 1.61E-10 |
| LSAMP | 0.317 | 1.87E-10 |
| RHOQ | 0.316 | 1.90E-10 |
| GHRL | 0.315 | 2.18E-10 |
| TECTA | 0.315 | 2.25E-10 |
| LRCH2 | 0.315 | 2.38E-10 |
| PANX1 | 0.314 | 2.52E-10 |
| EML1 | 0.314 | 2.62E-10 |
| PPT1 | 0.314 | 2.63E-10 |
| MINDY2 | 0.314 | 2.75E-10 |
| PPP4R1 | 0.314 | 2.75E-10 |
| SCHIP1 | 0.313 | 2.91E-10 |
| TNFSF18 | 0.313 | 2.94E-10 |
| TUB | 0.313 | 2.96E-10 |
| CD36 | 0.313 | 2.97E-10 |
| TMEM229B | 0.313 | 3.08E-10 |
| TIFA | 0.313 | 3.10E-10 |
| MKNK1 | 0.313 | 3.20E-10 |
| GPR18 | 0.313 | 3.20E-10 |
| MUSK | 0.312 | 3.44E-10 |
| RERG | 0.312 | 3.44E-10 |
| RNF152 | 0.311 | 3.74E-10 |
| TCP11L2 | 0.31 | 4.45E-10 |
| MFAP3 | 0.31 | 4.47E-10 |
| ZNF331 | 0.31 | 4.77E-10 |
| CARD6 | 0.31 | 4.85E-10 |
| PADI4 | 0.309 | 5.01E-10 |
| ACKR3 | 0.307 | 6.95E-10 |
| RASL12 | 0.307 | 7.31E-10 |
| ANKRD13A | 0.306 | 8.08E-10 |
| RBMS1 | 0.306 | 8.25E-10 |
| RPS6KA2 | 0.305 | 9.33E-10 |
| ATF5 | 0.305 | 9.45E-10 |
| NEGR1 | 0.304 | 1.04E-09 |
| RHEBL1 | 0.304 | 1.05E-09 |
| IFNGR1 | 0.303 | 1.19E-09 |
| CCDC117 | 0.302 | 1.41E-09 |
| ZFP92 | 0.301 | 1.55E-09 |
| WNK3 | 0.3 | 1.62E-09 |
| WDR47 | 0.3 | 1.66E-09 |
| C4orf3 | 0.3 | 1.72E-09 |
| IDO1 | 0.299 | 2.01E-09 |
| KCND3 | 0.299 | 2.01E-09 |
| KCNG2 | 0.299 | 2.04E-09 |
| ETV5 | 0.298 | 2.31E-09 |
| C21orf91 | 0.298 | 2.31E-09 |
| SLC17A7 | 0.297 | 2.45E-09 |
| TACR1 | 0.297 | 2.57E-09 |
| PPEF1 | 0.297 | 2.64E-09 |
| TMEM47 | 0.297 | 2.68E-09 |
| ADAMTSL1 | 0.295 | 3.46E-09 |
| ANTXR2 | 0.294 | 3.51E-09 |
| GBP6 | 0.294 | 3.53E-09 |
| C4orf32 | 0.294 | 3.83E-09 |
| BMF | 0.294 | 3.87E-09 |
| LRRC70 | 0.294 | 3.91E-09 |
| S100A8 | 0.294 | 3.91E-09 |
| B3GALNT1 | 0.293 | 3.98E-09 |
| SLC43A3 | 0.293 | 3.98E-09 |
| TMEM121 | 0.293 | 4.09E-09 |
| USP15 | 0.293 | 4.12E-09 |
| MAP3K7CL | 0.293 | 4.20E-09 |
| EOGT | 0.292 | 5.07E-09 |
| CHST12 | 0.29 | 5.96E-09 |
| RASSF8 | 0.29 | 6.08E-09 |
| BPGM | 0.29 | 6.11E-09 |
| CLEC1B | 0.29 | 6.38E-09 |
| S100A12 | 0.29 | 6.40E-09 |
| F8 | 0.29 | 6.43E-09 |
| EAF2 | 0.289 | 6.90E-09 |
| MYOZ3 | 0.288 | 7.57E-09 |
| MAPK12 | 0.288 | 7.80E-09 |
| ITM2B | 0.288 | 8.18E-09 |
| ENTPD7 | 0.288 | 8.37E-09 |
| DTX1 | 0.287 | 8.56E-09 |
| CHRNE | 0.287 | 8.79E-09 |
| STX5 | 0.287 | 9.05E-09 |
| ARL10 | 0.287 | 9.16E-09 |
| TUSC3 | 0.286 | 9.64E-09 |
| SHISA4 | 0.286 | 1.01E-08 |
| LRRN2 | 0.286 | 1.07E-08 |
| ZMIZ1 | 0.285 | 1.08E-08 |
| TMEM217 | 0.285 | 1.08E-08 |
| IQSEC1 | 0.285 | 1.10E-08 |
| DIXDC1 | 0.285 | 1.11E-08 |
| IL1RAP | 0.285 | 1.17E-08 |
| PRRT3 | 0.284 | 1.28E-08 |
| PTPN5 | 0.284 | 1.33E-08 |
| PLK2 | 0.283 | 1.43E-08 |
| ARPC2 | 0.283 | 1.50E-08 |
| PLEKHB2 | 0.283 | 1.50E-08 |
| COL4A4 | 0.282 | 1.65E-08 |
| BHLHA15 | 0.282 | 1.67E-08 |
| TRPC1 | 0.282 | 1.76E-08 |
| PITX3 | 0.281 | 1.77E-08 |
| GPR146 | 0.281 | 1.82E-08 |
| LCA5 | 0.281 | 1.89E-08 |
| MAP3K6 | 0.28 | 2.00E-08 |
| ZNF281 | 0.28 | 2.12E-08 |
| ABLIM3 | 0.28 | 2.17E-08 |
| NUB1 | 0.28 | 2.23E-08 |
| RAB2B | 0.279 | 2.52E-08 |
| BDKRB2 | 0.278 | 2.55E-08 |
| METTL24 | 0.278 | 2.61E-08 |
| HK1 | 0.277 | 2.88E-08 |
| FAM160B1 | 0.277 | 3.05E-08 |
| TMOD1 | 0.277 | 3.12E-08 |
| MAT2B | 0.276 | 3.31E-08 |
| MORF4L1 | 0.276 | 3.55E-08 |
| PCNX1 | 0.275 | 3.80E-08 |
| PELI3 | 0.274 | 4.15E-08 |
| CXCR3 | 0.274 | 4.15E-08 |
| INPP5B | 0.274 | 4.19E-08 |
| JAK1 | 0.274 | 4.39E-08 |
| NUDT11 | 0.274 | 4.49E-08 |
| NAP1L5 | 0.273 | 4.57E-08 |
| SEC14L6 | 0.273 | 4.65E-08 |
| AC072022.1 | 0.273 | 4.82E-08 |
| SLC5A4 | 0.271 | 5.80E-08 |
| DIP2C | 0.271 | 5.86E-08 |
| CAV2 | 0.271 | 5.95E-08 |
| SAMD14 | 0.271 | 6.05E-08 |
| FHL1 | 0.271 | 6.10E-08 |
| VAMP3 | 0.27 | 6.93E-08 |
| PLA2G15 | 0.269 | 7.77E-08 |
| CCDC102A | 0.268 | 8.39E-08 |
| GLIS1 | 0.268 | 8.78E-08 |
| RDX | 0.267 | 9.31E-08 |
| SUGCT | 0.267 | 9.60E-08 |
| LGI2 | 0.266 | 1.04E-07 |
| GALNT16 | 0.265 | 1.17E-07 |
| PGAM1 | 0.265 | 1.23E-07 |
| TRPC4 | 0.265 | 1.23E-07 |
| BBS4 | 0.265 | 1.27E-07 |
| TNIP3 | 0.265 | 1.27E-07 |
| XRN1 | 0.264 | 1.29E-07 |
| ARL2BP | 0.264 | 1.43E-07 |
| KCNIP1 | 0.263 | 1.45E-07 |
| HDAC7 | 0.263 | 1.57E-07 |
| FKBP5 | 0.263 | 1.60E-07 |
| KIAA1328 | 0.262 | 1.62E-07 |
| KDELC1 | 0.262 | 1.63E-07 |
| MGAM | 0.262 | 1.66E-07 |
| ACKR2 | 0.262 | 1.71E-07 |
| KIAA0513 | 0.262 | 1.71E-07 |
| CRADD | 0.262 | 1.73E-07 |
| PM20D1 | 0.261 | 1.83E-07 |
| CADM1 | 0.261 | 1.94E-07 |
| MEF2D | 0.261 | 2.00E-07 |
| TMEM200C | 0.26 | 2.02E-07 |
| GLCCI1 | 0.26 | 2.12E-07 |
| PKHD1L1 | 0.259 | 2.27E-07 |
| EDNRB | 0.259 | 2.34E-07 |
| ROBO1 | 0.259 | 2.35E-07 |
| GPR82 | 0.259 | 2.43E-07 |
| PHTF1 | 0.259 | 2.44E-07 |
| RASGEF1B | 0.258 | 2.58E-07 |
| KATNAL1 | 0.258 | 2.65E-07 |
| CPED1 | 0.258 | 2.79E-07 |
| OLFML2A | 0.256 | 3.19E-07 |
| CCDC71L | 0.256 | 3.22E-07 |
| GREB1 | 0.256 | 3.38E-07 |
| ST3GAL3 | 0.256 | 3.40E-07 |
| NEK1 | 0.254 | 3.99E-07 |
| RIT1 | 0.253 | 4.40E-07 |
| CDH13 | 0.253 | 4.42E-07 |
| TMOD2 | 0.253 | 4.78E-07 |
| C3orf18 | 0.252 | 4.89E-07 |
| CA11 | 0.252 | 4.97E-07 |
| PPP1R3C | 0.252 | 5.16E-07 |
| BVES | 0.251 | 5.40E-07 |
| PTPN7 | 0.251 | 5.69E-07 |
| SERINC1 | 0.251 | 5.85E-07 |
| RBMXL1 | 0.25 | 6.23E-07 |
| DENND5B | 0.25 | 6.28E-07 |
| ZNF699 | 0.249 | 6.70E-07 |
| ARHGAP22 | 0.249 | 6.91E-07 |
| TFPI | 0.249 | 7.08E-07 |
| HECTD2 | 0.248 | 7.79E-07 |
| SIT1 | 0.248 | 7.97E-07 |
| KCNS2 | 0.247 | 8.25E-07 |
| STX2 | 0.247 | 8.37E-07 |
| BTNL2 | 0.247 | 8.64E-07 |
| EFHD1 | 0.247 | 8.67E-07 |
| GPATCH3 | 0.246 | 9.23E-07 |
| NUCB1 | 0.246 | 9.44E-07 |
| LRP12 | 0.246 | 9.60E-07 |
| AP000781.2 | 0.246 | 1.01E-06 |
| TRIM8 | 0.245 | 1.02E-06 |
| ECEL1 | 0.245 | 1.05E-06 |
| GJC1 | 0.245 | 1.09E-06 |
| BBOF1 | 0.245 | 1.11E-06 |
| ADORA2A | 0.244 | 1.13E-06 |
| GULP1 | 0.244 | 1.14E-06 |
| BMP2K | 0.244 | 1.15E-06 |
| CNNM2 | 0.244 | 1.24E-06 |
| CRY1 | 0.244 | 1.24E-06 |
| SMIM10 | 0.243 | 1.26E-06 |
| FILIP1 | 0.242 | 1.42E-06 |
| CD109 | 0.242 | 1.47E-06 |
| CA5B | 0.242 | 1.49E-06 |
| ITGA8 | 0.242 | 1.51E-06 |
| YPEL5 | 0.241 | 1.58E-06 |
| CAP2 | 0.241 | 1.59E-06 |
| FGF12 | 0.241 | 1.60E-06 |
| IL11 | 0.241 | 1.66E-06 |
| KLHDC1 | 0.24 | 1.73E-06 |
| CYB5D2 | 0.239 | 1.89E-06 |
| MAP3K14 | 0.239 | 1.95E-06 |
| PGF | 0.239 | 2.03E-06 |
| COLGALT1 | 0.238 | 2.21E-06 |
| HSD17B6 | 0.238 | 2.23E-06 |
| KIAA1211 | 0.238 | 2.24E-06 |
| ZNF543 | 0.237 | 2.33E-06 |
| CFL2 | 0.237 | 2.34E-06 |
| ANGPT2 | 0.237 | 2.36E-06 |
| SSTR2 | 0.237 | 2.44E-06 |
| CD302 | 0.236 | 2.81E-06 |
| C8orf34 | 0.235 | 2.87E-06 |
| STOX2 | 0.235 | 2.97E-06 |
| MMRN1 | 0.234 | 3.18E-06 |
| GJB6 | 0.234 | 3.29E-06 |
| TFPI2 | 0.233 | 3.51E-06 |
| DERL2 | 0.232 | 3.84E-06 |
| AP1S2 | 0.232 | 3.85E-06 |
| CGRRF1 | 0.232 | 3.93E-06 |
| ARRB1 | 0.231 | 4.57E-06 |
| SLC37A2 | 0.23 | 4.65E-06 |
| ADGRG5 | 0.229 | 5.29E-06 |
| GBGT1 | 0.228 | 5.74E-06 |
| CCNG2 | 0.228 | 5.89E-06 |
| BACE1 | 0.227 | 6.18E-06 |
| PYGO1 | 0.227 | 6.26E-06 |
| ZNF175 | 0.227 | 6.38E-06 |
| RTN4RL2 | 0.227 | 6.42E-06 |
| TRAPPC8 | 0.227 | 6.51E-06 |
| C15orf53 | 0.227 | 6.61E-06 |
| RBM43 | 0.226 | 6.81E-06 |
| P2RX5-TAX1BP3 | 0.226 | 6.97E-06 |
| TESK2 | 0.226 | 7.21E-06 |
| RSPO3 | 0.226 | 7.47E-06 |
| TOR1AIP1 | 0.225 | 7.57E-06 |
| DRAXIN | 0.225 | 7.83E-06 |
| SLC16A12 | 0.225 | 7.94E-06 |
| SPRYD3 | 0.225 | 7.98E-06 |
| ZNF264 | 0.225 | 8.06E-06 |
| AOC2 | 0.224 | 8.27E-06 |
| RRAGC | 0.224 | 8.31E-06 |
| VIPR2 | 0.224 | 8.65E-06 |
| CAVIN2 | 0.224 | 8.93E-06 |
| CDH6 | 0.222 | 9.98E-06 |
| WWC2 | 0.222 | 9.99E-06 |
| PCED1B | 0.222 | 1.00E-05 |
| MICU1 | 0.222 | 1.01E-05 |
| FBXO32 | 0.222 | 1.03E-05 |
| STAC3 | 0.222 | 1.05E-05 |
| SDC3 | 0.221 | 1.11E-05 |
| KCNAB2 | 0.221 | 1.13E-05 |
| CIC | 0.22 | 1.21E-05 |
| GRK4 | 0.219 | 1.34E-05 |
| RBM23 | 0.219 | 1.40E-05 |
| CXCL5 | 0.218 | 1.46E-05 |
| KRBA1 | 0.218 | 1.46E-05 |
| GRB2 | 0.218 | 1.55E-05 |
| CORO2B | 0.217 | 1.58E-05 |
| HIP1 | 0.216 | 1.78E-05 |
| TTC5 | 0.216 | 1.80E-05 |
| BAK1 | 0.216 | 1.83E-05 |
| C12orf4 | 0.216 | 1.84E-05 |
| FAM13B | 0.216 | 1.90E-05 |
| NUDT18 | 0.215 | 1.92E-05 |
| ADAMTS6 | 0.214 | 2.11E-05 |
| CD63 | 0.214 | 2.13E-05 |
| PTPRR | 0.214 | 2.16E-05 |
| ZNF101 | 0.214 | 2.25E-05 |
| TBCEL | 0.213 | 2.38E-05 |
| SLC18B1 | 0.213 | 2.48E-05 |
| PI15 | 0.212 | 2.52E-05 |
| TTC7B | 0.212 | 2.61E-05 |
| CYTH3 | 0.212 | 2.64E-05 |
| MYO16 | 0.212 | 2.65E-05 |
| SNTB2 | 0.211 | 2.83E-05 |
| FBXO33 | 0.211 | 2.88E-05 |
| ARL9 | 0.211 | 2.91E-05 |
| LRIG3 | 0.21 | 2.99E-05 |
| TRIM23 | 0.21 | 3.03E-05 |
| ATL1 | 0.209 | 3.30E-05 |
| C4orf50 | 0.209 | 3.38E-05 |
| HTR3A | 0.209 | 3.54E-05 |
| FAM180B | 0.208 | 3.60E-05 |
| NECAP1 | 0.208 | 3.74E-05 |
| PXK | 0.208 | 3.76E-05 |
| WNT5A | 0.208 | 3.79E-05 |
| PCDH18 | 0.208 | 3.84E-05 |
| CDC42EP1 | 0.207 | 3.90E-05 |
| TP73 | 0.207 | 4.02E-05 |
| OPN1SW | 0.206 | 4.45E-05 |
| KRT27 | 0.206 | 4.50E-05 |
| IL31RA | 0.206 | 4.51E-05 |
| DNAAF3 | 0.205 | 4.75E-05 |
| PTGS2 | 0.205 | 5.02E-05 |
| ZNF788 | 0.205 | 5.05E-05 |
| RAPGEF3 | 0.204 | 5.26E-05 |
| HELQ | 0.204 | 5.37E-05 |
| PLCL1 | 0.204 | 5.45E-05 |
| ZNF143 | 0.203 | 5.54E-05 |
| JADE1 | 0.203 | 5.70E-05 |
| C18orf54 | 0.203 | 5.71E-05 |
| MAFA | 0.203 | 5.99E-05 |
| FAM163A | 0.203 | 6.00E-05 |
| RTN2 | 0.202 | 6.20E-05 |
| HAL | 0.202 | 6.27E-05 |
| ANKS1B | 0.202 | 6.28E-05 |
| RAB39A | 0.202 | 6.40E-05 |
| VIP | 0.201 | 7.01E-05 |
| ARSJ | 0.2 | 7.24E-05 |
| CYBA | 0.2 | 7.49E-05 |
| USP51 | 0.199 | 8.09E-05 |
| CCDC89 | 0.199 | 8.13E-05 |
| SIK3 | 0.199 | 8.21E-05 |
| SESTD1 | 0.198 | 8.52E-05 |
| UBE2Z | 0.198 | 8.71E-05 |
| NPFFR2 | 0.198 | 8.72E-05 |
| UBE2J1 | 0.198 | 9.00E-05 |
| SEMA6C | 0.198 | 9.14E-05 |
| DDHD1 | 0.196 | 0.0001 |
| SEC22C | 0.196 | 0.000101 |
| PIAS1 | 0.196 | 0.000106 |
| CD5 | 0.196 | 0.000108 |
| TIMM21 | 0.196 | 0.000108 |
| AKAP2 | 0.195 | 0.000108 |
| IFIT1 | 0.195 | 0.000109 |
| SMYD4 | 0.195 | 0.000114 |
| TTBK2 | 0.195 | 0.000115 |
| ZNF267 | 0.194 | 0.000124 |
| MPP2 | 0.194 | 0.000126 |
| MERTK | 0.193 | 0.000134 |
| ZYX | 0.192 | 0.000141 |
| ZNF491 | 0.192 | 0.000143 |
| SLC12A5 | 0.192 | 0.000145 |
| SKIDA1 | 0.191 | 0.000152 |
| DUSP4 | 0.191 | 0.000154 |
| NACC2 | 0.191 | 0.000154 |
| GCNT2 | 0.191 | 0.000159 |
| PTPN9 | 0.19 | 0.000165 |
| SRSF12 | 0.19 | 0.000165 |
| DYNC2H1 | 0.19 | 0.000165 |
| HACE1 | 0.19 | 0.000171 |
| SCEL | 0.19 | 0.000171 |
| NCALD | 0.189 | 0.00018 |
| BST1 | 0.189 | 0.000181 |
| DCLK3 | 0.189 | 0.000184 |
| EMSY | 0.188 | 0.000192 |
| LYPD3 | 0.188 | 0.000196 |
| IL20 | 0.188 | 0.000201 |
| TFIP11 | 0.188 | 0.000207 |
| ATP1A3 | 0.187 | 0.000209 |
| TM4SF18 | 0.187 | 0.00021 |
| 8-Mar | 0.187 | 0.000213 |
| CAPN11 | 0.187 | 0.000213 |
| AGO1 | 0.187 | 0.000214 |
| PICALM | 0.187 | 0.000217 |
| GPR63 | 0.187 | 0.000219 |
| ARHGAP19 | 0.187 | 0.00022 |
| CSNK1G1 | 0.187 | 0.000223 |
| DENND4A | 0.185 | 0.00025 |
| NFATC3 | 0.185 | 0.000252 |
| RANBP3L | 0.185 | 0.00026 |
| TNFSF11 | 0.184 | 0.000265 |
| STARD6 | 0.184 | 0.000282 |
| SLC36A1 | 0.183 | 0.000287 |
| GP5 | 0.183 | 0.000288 |
| HRASLS | 0.183 | 0.000292 |
| ZFP82 | 0.183 | 0.000295 |
| SLC7A10 | 0.183 | 0.000303 |
| SLC35G2 | 0.182 | 0.000311 |
| PGBD1 | 0.182 | 0.00032 |
| SLC25A34 | 0.182 | 0.000326 |
| CCSAP | 0.182 | 0.000327 |
| C10orf76 | 0.181 | 0.000334 |
| BAZ2A | 0.181 | 0.000337 |
| TMPRSS3 | 0.181 | 0.000346 |
| NAB2 | 0.181 | 0.000354 |
| CAPN14 | 0.18 | 0.000368 |
| CBARP | 0.18 | 0.000369 |
| UVRAG | 0.18 | 0.000378 |
| ZFP36L1 | 0.179 | 0.000391 |
| CHST10 | 0.179 | 0.000399 |
| NPY2R | 0.179 | 0.000403 |
| CERS5 | 0.179 | 0.000412 |
| ZNF540 | 0.178 | 0.000419 |
| SNIP1 | 0.177 | 0.000457 |
| FBXO10 | 0.177 | 0.000476 |
| ARL13B | 0.177 | 0.000477 |
| ST6GALNAC3 | 0.176 | 0.000498 |
| LPCAT1 | 0.176 | 0.000499 |
| PPP2R3C | 0.176 | 0.000515 |
| WDFY2 | 0.176 | 0.000521 |
| AL365273.2 | 0.175 | 0.000524 |
| CDC42EP2 | 0.175 | 0.000529 |
| NKIRAS1 | 0.175 | 0.000531 |
| F2RL2 | 0.175 | 0.000535 |
| FOXI2 | 0.175 | 0.000539 |
| KLHL28 | 0.175 | 0.000545 |
| S100PBP | 0.174 | 0.00057 |
| NECTIN2 | 0.174 | 0.00057 |
| MLC1 | 0.174 | 0.000576 |
| CHUK | 0.174 | 0.00059 |
| NOL9 | 0.173 | 0.00061 |
| BEX4 | 0.173 | 0.000615 |
| MAD2L2 | 0.173 | 0.000619 |
| B3GNT2 | 0.173 | 0.000622 |
| FOSB | 0.173 | 0.000628 |
| SIN3A | 0.173 | 0.000636 |
| CD177 | 0.172 | 0.000657 |
| IL26 | 0.172 | 0.000663 |
| ZNF624 | 0.172 | 0.000677 |
| ZKSCAN7 | 0.172 | 0.000682 |
| LPAR6 | 0.172 | 0.000689 |
| M1AP | 0.172 | 0.000691 |
| 3-Sep | 0.171 | 0.000706 |
| IGFL1 | 0.171 | 0.000713 |
| CTSH | 0.171 | 0.000725 |
| BBX | 0.171 | 0.00074 |
| PAG1 | 0.17 | 0.000763 |
| TTC9B | 0.17 | 0.00077 |
| TNFSF9 | 0.17 | 0.000798 |
| FTO | 0.17 | 0.000799 |
| ADAM22 | 0.169 | 0.000822 |
| SP4 | 0.169 | 0.000834 |
| SLC35B4 | 0.169 | 0.000843 |
| HES4 | 0.169 | 0.000858 |
| MFSD14A | 0.168 | 0.000896 |
| PRDM16 | 0.168 | 0.000918 |
| QRICH1 | 0.168 | 0.000928 |
| CDON | 0.168 | 0.000938 |
| TFAP2A | 0.167 | 0.00097 |
| AP4E1 | 0.167 | 0.000972 |
| GPR137C | 0.167 | 0.000992 |
| CEP135 | 0.166 | 0.001034 |
| CEP97 | 0.166 | 0.001049 |
| MYEOV | 0.166 | 0.00105 |
| ERO1B | 0.166 | 0.001067 |
| CASP3 | 0.165 | 0.001096 |
| KRT16 | 0.165 | 0.001136 |
| ATP6V1E1 | 0.165 | 0.001155 |
| TMTC2 | 0.164 | 0.001226 |
| SOS1 | 0.163 | 0.001303 |
| SCN3B | 0.162 | 0.001339 |
| TBC1D15 | 0.162 | 0.001383 |
| ANGPTL1 | 0.161 | 0.001448 |
| ZNF70 | 0.16 | 0.001628 |
| KLK10 | 0.16 | 0.001631 |
| KCTD1 | 0.16 | 0.001639 |
| GNAI3 | 0.159 | 0.001659 |
| ULBP2 | 0.159 | 0.001755 |
| AKAP10 | 0.158 | 0.001778 |
| GRIN2A | 0.158 | 0.001792 |
| CDC14A | 0.158 | 0.001795 |
| GJB5 | 0.158 | 0.001801 |
| TOM1 | 0.158 | 0.001809 |
| AZI2 | 0.158 | 0.001812 |
| PRKD3 | 0.158 | 0.001817 |
| TTYH3 | 0.158 | 0.001825 |
| YPEL2 | 0.158 | 0.001827 |
| MYH2 | 0.158 | 0.001851 |
| TSPAN18 | 0.158 | 0.001862 |
| ZNF569 | 0.158 | 0.001881 |
| TOM1L2 | 0.158 | 0.001884 |
| LARGE1 | 0.157 | 0.001894 |
| FAM53B | 0.157 | 0.001922 |
| FRS2 | 0.157 | 0.001929 |
| CLCA2 | 0.157 | 0.001932 |
| SLC30A7 | 0.157 | 0.001973 |
| MYO7A | 0.156 | 0.00205 |
| APOC4-APOC2 | 0.156 | 0.002095 |
| ACR | 0.156 | 0.002124 |
| WDPCP | 0.156 | 0.002131 |
| PLSCR1 | 0.155 | 0.002184 |
| C16orf72 | 0.155 | 0.002196 |
| ZNF568 | 0.155 | 0.00226 |
| CCDC152 | 0.155 | 0.002265 |
| RILPL1 | 0.155 | 0.00229 |
| LPL | 0.154 | 0.002308 |
| TPK1 | 0.154 | 0.002328 |
| FADS3 | 0.154 | 0.00236 |
| CNTLN | 0.154 | 0.002364 |
| RHOBTB2 | 0.154 | 0.002366 |
| SLC35A5 | 0.153 | 0.002478 |
| CREBRF | 0.153 | 0.002491 |
| RRM2B | 0.153 | 0.002549 |
| FAM214B | 0.153 | 0.002588 |
| FOLH1 | 0.153 | 0.002624 |
| SLC35E1 | 0.152 | 0.002704 |
| RAET1G | 0.152 | 0.002742 |
| AC008750.8 | 0.152 | 0.002776 |
| TTC39C | 0.151 | 0.002879 |
| ZNF396 | 0.151 | 0.002939 |
| RBM7 | 0.151 | 0.002945 |
| PHOSPHO1 | 0.15 | 0.003105 |
| CNEP1R1 | 0.15 | 0.003137 |
| KRT6A | 0.15 | 0.003145 |
| R3HCC1L | 0.15 | 0.003153 |
| SOX13 | 0.15 | 0.003158 |
| CTBS | 0.15 | 0.003168 |
| HMGN4 | 0.15 | 0.003169 |
| PDK1 | 0.149 | 0.00325 |
| TAF13 | 0.149 | 0.003327 |
| MED18 | 0.149 | 0.003338 |
| ART4 | 0.149 | 0.003371 |
| TBCCD1 | 0.149 | 0.003396 |
| SLC35E3 | 0.148 | 0.00359 |
| JDP2 | 0.148 | 0.003614 |
| TMPRSS11D | 0.147 | 0.003638 |
| ZNF829 | 0.147 | 0.003655 |
| BMX | 0.147 | 0.003699 |
| KRT6C | 0.147 | 0.003705 |
| IL7 | 0.147 | 0.003709 |
| BMT2 | 0.147 | 0.003741 |
| PDE8B | 0.147 | 0.003757 |
| RGS5 | 0.147 | 0.003846 |
| PELI2 | 0.146 | 0.003919 |
| DNAJC28 | 0.146 | 0.003919 |
| MB21D2 | 0.146 | 0.003921 |
| BCKDK | 0.146 | 0.003992 |
| EDNRA | 0.145 | 0.004199 |
| RGCC | 0.145 | 0.004214 |
| PHC2 | 0.145 | 0.00422 |
| CNN2 | 0.145 | 0.00422 |
| PKIB | 0.145 | 0.004233 |
| CLSPN | 0.145 | 0.004258 |
| PPP6R1 | 0.145 | 0.004266 |
| WNT7B | 0.145 | 0.004333 |
| AARD | 0.144 | 0.004461 |
| SUSD4 | 0.144 | 0.004533 |
| ADAM17 | 0.144 | 0.004584 |
| CD44 | 0.143 | 0.00476 |
| ACCSL | 0.143 | 0.004767 |
| IL36RN | 0.143 | 0.004793 |
| ERCC4 | 0.143 | 0.004805 |
| CKAP4 | 0.143 | 0.004819 |
| TPCN2 | 0.143 | 0.004832 |
| CHIA | 0.143 | 0.004912 |
| DLL1 | 0.143 | 0.004946 |
| CCNYL1 | 0.142 | 0.004997 |
| CRCT1 | 0.142 | 0.005048 |
| SPRR2E | 0.142 | 0.00506 |
| CPPED1 | 0.142 | 0.00513 |
| RWDD2A | 0.142 | 0.005149 |
| ZRANB1 | 0.142 | 0.00528 |
| COMMD9 | 0.141 | 0.005321 |
| NANOS1 | 0.141 | 0.005389 |
| UNC13D | 0.141 | 0.005462 |
| SEMA6D | 0.141 | 0.00547 |
| SPRR2D | 0.14 | 0.005883 |
| SPRR2G | 0.14 | 0.005891 |
| PIK3CA | 0.14 | 0.005893 |
| FAM19A4 | 0.14 | 0.005927 |
| EML5 | 0.14 | 0.005947 |
| ATP5B | 0.14 | 0.005951 |
| ZNF772 | 0.14 | 0.005963 |
| DPF1 | 0.139 | 0.006117 |
| PRMT9 | 0.139 | 0.006126 |
| RIPK2 | 0.139 | 0.00619 |
| MORC4 | 0.139 | 0.006196 |
| ZBTB22 | 0.139 | 0.006216 |
| WDFY3 | 0.139 | 0.006293 |
| FLT3 | 0.138 | 0.006498 |
| CYB5R4 | 0.138 | 0.006686 |
| HSD17B1 | 0.138 | 0.006736 |
| IVL | 0.137 | 0.006806 |
| AMIGO2 | 0.137 | 0.006858 |
| ZDHHC19 | 0.137 | 0.00693 |
| KRT81 | 0.137 | 0.00697 |
| RNF115 | 0.137 | 0.007002 |
| BBS9 | 0.137 | 0.007015 |
| ARID1A | 0.137 | 0.00709 |
| RAB29 | 0.137 | 0.007129 |
| APELA | 0.137 | 0.007141 |
| TTL | 0.137 | 0.007145 |
| STYX | 0.136 | 0.007186 |
| CLCN6 | 0.136 | 0.007397 |
| SLF2 | 0.136 | 0.007492 |
| SPRR2A | 0.135 | 0.007717 |
| RNF121 | 0.135 | 0.007787 |
| TRAM1 | 0.135 | 0.007911 |
| C17orf105 | 0.135 | 0.008004 |
| ERLEC1 | 0.135 | 0.008013 |
| AGO4 | 0.135 | 0.008033 |
| SLITRK4 | 0.135 | 0.008046 |
| SPCS3 | 0.134 | 0.008181 |
| KCNB2 | 0.134 | 0.008231 |
| RNF146 | 0.134 | 0.008381 |
| GGCX | 0.134 | 0.008451 |
| RAB33B | 0.134 | 0.008498 |
| KRT6B | 0.133 | 0.008586 |
| POMK | 0.133 | 0.008635 |
| C17orf67 | 0.133 | 0.008883 |
| KISS1R | 0.133 | 0.00894 |
| TNNT1 | 0.132 | 0.009154 |
| SPINK2 | 0.132 | 0.009184 |
| SOGA3 | 0.132 | 0.009237 |
| SPRR1B | 0.132 | 0.009293 |
| DCLRE1B | 0.132 | 0.009425 |
| SYNRG | 0.132 | 0.009492 |
| UHRF1BP1L | 0.132 | 0.009583 |
| MYL2 | 0.132 | 0.009589 |
| RHOC | 0.131 | 0.009637 |
| XXYLT1 | 0.131 | 0.01014 |
| POLDIP3 | 0.13 | 0.010239 |
| FAM209B | 0.13 | 0.0103 |
| WNT10B | 0.129 | 0.010799 |
| CP | 0.129 | 0.010856 |
| FYCO1 | 0.129 | 0.010933 |
| RCHY1 | 0.129 | 0.010941 |
| IDH3A | 0.129 | 0.010982 |
| GPLD1 | 0.129 | 0.010985 |
| DBR1 | 0.129 | 0.011258 |
| ZNF136 | 0.128 | 0.01144 |
| ZNF354C | 0.128 | 0.011585 |
| SLC6A14 | 0.128 | 0.011601 |
| GRAP | 0.128 | 0.011639 |
| MAPK8IP2 | 0.128 | 0.011685 |
| NOVA1 | 0.128 | 0.011784 |
| TSPYL1 | 0.128 | 0.01194 |
| COPS7A | 0.128 | 0.011991 |
| CDK9 | 0.127 | 0.012403 |
| ACHE | 0.127 | 0.012559 |
| HOOK3 | 0.127 | 0.012586 |
| PDXP | 0.127 | 0.012637 |
| ZNF726 | 0.126 | 0.012941 |
| WSB2 | 0.126 | 0.013147 |
| MOB1A | 0.126 | 0.013237 |
| DSCR3 | 0.126 | 0.013248 |
| ZNF484 | 0.126 | 0.013355 |
| GCNT3 | 0.126 | 0.013434 |
| SPATA18 | 0.125 | 0.013811 |
| EYA4 | 0.125 | 0.014011 |
| PPM1A | 0.125 | 0.014054 |
| TSPAN14 | 0.125 | 0.014227 |
| REL | 0.124 | 0.01429 |
| EXTL3 | 0.124 | 0.014404 |
| C3orf84 | 0.124 | 0.014769 |
| APPBP2 | 0.124 | 0.014775 |
| CARNS1 | 0.124 | 0.014994 |
| CHTF8 | 0.123 | 0.015139 |
| CFAP43 | 0.123 | 0.015168 |
| UBR2 | 0.123 | 0.015236 |
| FAM170B | 0.123 | 0.015391 |
| DIO1 | 0.123 | 0.015402 |
| CEP128 | 0.123 | 0.015429 |
| KLF1 | 0.123 | 0.015454 |
| PRTN3 | 0.123 | 0.015618 |
| OMG | 0.123 | 0.015622 |
| BTBD17 | 0.123 | 0.015753 |
| BIRC7 | 0.123 | 0.015808 |
| ZNF689 | 0.122 | 0.016188 |
| DGKA | 0.122 | 0.016373 |
| VWA3B | 0.122 | 0.016481 |
| OTOP2 | 0.121 | 0.017282 |
| RDH8 | 0.121 | 0.017384 |
| KRT7 | 0.121 | 0.017595 |
| IL1A | 0.12 | 0.017812 |
| PCNX4 | 0.12 | 0.018032 |
| GUCA2B | 0.12 | 0.018138 |
| RNF13 | 0.12 | 0.018166 |
| KLK13 | 0.12 | 0.018264 |
| LTK | 0.119 | 0.018696 |
| MPHOSPH9 | 0.119 | 0.018821 |
| SFXN1 | 0.119 | 0.019037 |
| PARP11 | 0.119 | 0.01915 |
| TSPAN2 | 0.119 | 0.019206 |
| POU3F3 | 0.118 | 0.019728 |
| MYH8 | 0.118 | 0.019745 |
| MSRA | 0.118 | 0.019794 |
| OTOP3 | 0.118 | 0.019809 |
| C2CD2 | 0.118 | 0.019875 |
| PSAPL1 | 0.118 | 0.020241 |
| NRG1 | 0.118 | 0.020519 |
| AFAP1 | 0.118 | 0.020638 |
| RHD | 0.118 | 0.020718 |
| TMIGD1 | 0.118 | 0.020733 |
| ZBTB25 | 0.117 | 0.021005 |
| RAD9B | 0.117 | 0.021013 |
| ZNF287 | 0.117 | 0.02115 |
| EIF2AK3 | 0.117 | 0.021273 |
| CNOT6L | 0.117 | 0.021366 |
| ZNF625 | 0.117 | 0.021511 |
| C12orf60 | 0.117 | 0.021655 |
| CACNA2D4 | 0.117 | 0.021875 |
| TECPR1 | 0.116 | 0.0223 |
| GABARAPL2 | 0.116 | 0.022329 |
| MKL2 | 0.116 | 0.0224 |
| ALOX15B | 0.116 | 0.022409 |
| C9orf72 | 0.116 | 0.022679 |
| ETNPPL | 0.116 | 0.023034 |
| PPM1D | 0.116 | 0.023037 |
| SPACA6 | 0.115 | 0.023116 |
| HRH3 | 0.115 | 0.023236 |
| AMPH | 0.115 | 0.023726 |
| RELA | 0.115 | 0.023844 |
| MAPK8 | 0.115 | 0.024211 |
| FADS1 | 0.114 | 0.024355 |
| HBG1 | 0.114 | 0.024482 |
| SOCS5 | 0.114 | 0.024602 |
| TFAP2D | 0.114 | 0.0247 |
| TMPPE | 0.114 | 0.024948 |
| IL12A | 0.114 | 0.025023 |
| FOXD4 | 0.114 | 0.025102 |
| RGS17 | 0.113 | 0.025868 |
| FXYD1 | 0.113 | 0.025934 |
| NR4A2 | 0.113 | 0.025983 |
| LETM2 | 0.113 | 0.026193 |
| RAPH1 | 0.113 | 0.026455 |
| CA4 | 0.113 | 0.026547 |
| MED12L | 0.112 | 0.027137 |
| PLOD2 | 0.112 | 0.027389 |
| SLC4A7 | 0.112 | 0.027532 |
| SENP7 | 0.112 | 0.027664 |
| CYSRT1 | 0.112 | 0.027853 |
| HMCN2 | 0.112 | 0.027918 |
| ARPC1B | 0.112 | 0.028004 |
| IL34 | 0.111 | 0.028315 |
| THOC5 | 0.111 | 0.028389 |
| FSD1 | 0.111 | 0.028453 |
| VSTM2B | 0.111 | 0.028693 |
| ST6GALNAC2 | 0.111 | 0.028894 |
| KLK5 | 0.111 | 0.028943 |
| USP32 | 0.111 | 0.029162 |
| PLA2G3 | 0.111 | 0.02958 |
| FOXI1 | 0.111 | 0.029742 |
| NAALADL1 | 0.11 | 0.029809 |
| EMILIN3 | 0.11 | 0.029829 |
| RNH1 | 0.11 | 0.029956 |
| RNF169 | 0.11 | 0.030074 |
| LCE3C | 0.11 | 0.030113 |
| AC138811.2 | 0.11 | 0.030138 |
| UBR1 | 0.11 | 0.030286 |
| SLC22A4 | 0.11 | 0.030753 |
| FAM209A | 0.109 | 0.031612 |
| POLI | 0.109 | 0.03176 |
| CSPG5 | 0.109 | 0.031993 |
| IL1R2 | 0.109 | 0.032181 |
| PIM3 | 0.109 | 0.032182 |
| FAM160B2 | 0.108 | 0.033012 |
| PTPN13 | 0.108 | 0.0333 |
| DUOX1 | 0.108 | 0.033558 |
| PCDHGB1 | 0.108 | 0.033829 |
| PPP4R4 | 0.108 | 0.03404 |
| GATA5 | 0.108 | 0.034043 |
| CGB8 | 0.108 | 0.034192 |
| ALCAM | 0.108 | 0.03434 |
| EDN3 | 0.107 | 0.034603 |
| CCDC163 | 0.107 | 0.034894 |
| NBN | 0.107 | 0.035312 |
| IRAK2 | 0.107 | 0.035325 |
| C9orf66 | 0.107 | 0.036193 |
| ADGRE5 | 0.106 | 0.036674 |
| ZNF428 | 0.106 | 0.036895 |
| TMEM151A | 0.106 | 0.036936 |
| SPAG6 | 0.106 | 0.037073 |
| MYRF | 0.106 | 0.037294 |
| NSL1 | 0.106 | 0.037898 |
| CCND3 | 0.105 | 0.038972 |
| ADCY1 | 0.105 | 0.039311 |
| TAOK3 | 0.105 | 0.039557 |
| THAP11 | 0.104 | 0.040028 |
| TAF3 | 0.104 | 0.040089 |
| ZYG11A | 0.104 | 0.040423 |
| RPL17 | 0.104 | 0.040427 |
| TRAPPC6B | 0.104 | 0.040854 |
| SCARB2 | 0.104 | 0.040966 |
| CYP26C1 | 0.104 | 0.041256 |
| SLC1A1 | 0.103 | 0.04231 |
| TOX | 0.103 | 0.042547 |
| SHISA2 | 0.103 | 0.042969 |
| RFX7 | 0.103 | 0.043333 |
| ZCWPW2 | 0.103 | 0.043402 |
| DPP4 | 0.103 | 0.043413 |
| CD47 | 0.103 | 0.043554 |
| TSR1 | 0.103 | 0.043859 |
| C6orf201 | 0.102 | 0.043932 |
| EXTL2 | 0.102 | 0.044105 |
| AL161911.1 | 0.102 | 0.044999 |
| TRAF6 | 0.102 | 0.045022 |
| RAB9B | 0.102 | 0.045262 |
| ZNF384 | 0.102 | 0.04527 |
| GRIN2C | 0.102 | 0.045308 |
| CSMD3 | 0.102 | 0.045675 |
| CGB5 | 0.101 | 0.046133 |
| NR1H2 | 0.101 | 0.046735 |
| TGIF2LX | 0.101 | 0.046799 |
| VIPAS39 | 0.101 | 0.046873 |
| CDC73 | 0.101 | 0.0469 |
| STAG1 | 0.101 | 0.047144 |
| TMEM40 | 0.101 | 0.047175 |
| PDE6D | 0.101 | 0.047269 |
| WDR24 | 0.101 | 0.047392 |
| PAQR3 | 0.1 | 0.049879 |
| DOCK1 | 0.1 | 0.050453 |
| RANBP9 | 0.099 | 0.05134 |
| AC106782.1 | 0.099 | 0.051534 |
| SPRR3 | 0.099 | 0.051632 |
| DNAH2 | 0.099 | 0.051882 |
| N4BP2L1 | 0.099 | 0.0519 |
| VEZT | 0.099 | 0.052048 |
| LRRC40 | 0.099 | 0.052184 |
| LCN2 | 0.098 | 0.053187 |
| PPP1R21 | 0.098 | 0.053482 |
| PLET1 | 0.098 | 0.053578 |
| ZNF555 | 0.098 | 0.05398 |
| NTRK2 | 0.098 | 0.05453 |
| PAPOLG | 0.098 | 0.054587 |
| RASL10A | 0.098 | 0.054629 |
| ZNF483 | 0.098 | 0.054832 |
| C22orf23 | 0.098 | 0.055041 |
| KCNMB2 | 0.097 | 0.055457 |
| C11orf63 | 0.097 | 0.055738 |
| ARHGAP42 | 0.097 | 0.05599 |
| TRIM9 | 0.097 | 0.05606 |
| VWA5B2 | 0.097 | 0.056166 |
| PHF5A | 0.097 | 0.056281 |
| ASB7 | 0.097 | 0.056541 |
| KIAA1586 | 0.097 | 0.056597 |
| YPEL1 | 0.097 | 0.057115 |
| C19orf54 | 0.097 | 0.057283 |
| GPM6B | 0.097 | 0.057352 |
| ABI1 | 0.096 | 0.059118 |
| RLF | 0.096 | 0.059131 |
| C2CD4B | 0.096 | 0.059458 |
| TUBB3 | 0.096 | 0.060049 |
| CLTCL1 | 0.096 | 0.060051 |
| FLRT3 | 0.095 | 0.06071 |
| PRSS21 | 0.095 | 0.060859 |
| SLC30A10 | 0.095 | 0.061521 |
| C5orf58 | 0.095 | 0.061983 |
| ABCG2 | 0.095 | 0.062621 |
| MUC1 | 0.094 | 0.063733 |
| FKBP1C | 0.094 | 0.064135 |
| HPS3 | 0.094 | 0.064155 |
| YAF2 | 0.094 | 0.064547 |
| NT5DC3 | 0.094 | 0.065676 |
| CNKSR2 | 0.093 | 0.066996 |
| SENP1 | 0.093 | 0.06714 |
| SLC9A5 | 0.093 | 0.067402 |
| VNN1 | 0.093 | 0.067435 |
| SLC26A11 | 0.093 | 0.067713 |
| WNT8B | 0.093 | 0.068289 |
| GAB2 | 0.092 | 0.069125 |
| MAP9 | 0.092 | 0.069155 |
| MEGF8 | 0.092 | 0.069218 |
| SWT1 | 0.092 | 0.070629 |
| AC079447.1 | 0.092 | 0.070841 |
| C22orf15 | 0.092 | 0.071023 |
| TTLL9 | 0.092 | 0.071173 |
| C3orf58 | 0.092 | 0.071579 |
| AL136531.2 | 0.091 | 0.072656 |
| UPK3B | 0.091 | 0.072669 |
| GALNT18 | 0.091 | 0.073095 |
| SPINK7 | 0.091 | 0.073337 |
| SERPINB7 | 0.091 | 0.073358 |
| FEZ2 | 0.091 | 0.073544 |
| RALB | 0.091 | 0.073579 |
| OTULIN | 0.091 | 0.073922 |
| PTPA | 0.091 | 0.07396 |
| CTDSPL2 | 0.091 | 0.07415 |
| GABRB3 | 0.091 | 0.075167 |
| GFI1B | 0.09 | 0.076086 |
| CAMSAP2 | 0.09 | 0.076155 |
| ZNF628 | 0.09 | 0.076864 |
| AMER2 | 0.09 | 0.077221 |
| IL23A | 0.09 | 0.077295 |
| HYDIN | 0.09 | 0.07736 |
| MCM9 | 0.09 | 0.077821 |
| PRDM10 | 0.09 | 0.077824 |
| IGSF1 | 0.089 | 0.078685 |
| MRFAP1 | 0.089 | 0.079339 |
| KCNRG | 0.089 | 0.079699 |
| DCP2 | 0.089 | 0.079924 |
| GPM6A | 0.089 | 0.080003 |
| TOMM40L | 0.089 | 0.080042 |
| CDK5R2 | 0.089 | 0.080069 |
| CAPZA1 | 0.089 | 0.080089 |
| GPN3 | 0.089 | 0.080412 |
| OR2B6 | 0.089 | 0.080769 |
| CCDC141 | 0.089 | 0.081054 |
| FAM218A | 0.089 | 0.081078 |
| SMC1B | 0.089 | 0.081556 |
| IGSF9B | 0.088 | 0.08218 |
| SMIM18 | 0.088 | 0.082452 |
| RAB5C | 0.088 | 0.084111 |
| ITSN2 | 0.088 | 0.084944 |
| NEMP2 | 0.088 | 0.085397 |
| ELSPBP1 | 0.087 | 0.085997 |
| CDIP1 | 0.087 | 0.086084 |
| DBNL | 0.087 | 0.086196 |
| USP5 | 0.087 | 0.086943 |
| ARL8B | 0.087 | 0.087084 |
| TCEA2 | 0.087 | 0.087965 |
| IL13RA2 | 0.087 | 0.08797 |
| CREB1 | 0.087 | 0.088035 |
| TIGD7 | 0.087 | 0.088822 |
| CBFA2T3 | 0.086 | 0.089662 |
| ZNF557 | 0.086 | 0.090985 |
| GALNS | 0.086 | 0.09114 |
| SLC27A1 | 0.086 | 0.09126 |
| NFATC2 | 0.086 | 0.091273 |
| TMEM145 | 0.086 | 0.091964 |
| NAALAD2 | 0.086 | 0.092415 |
| TYRP1 | 0.086 | 0.092422 |
| KPNA5 | 0.085 | 0.093775 |
| IMPA1 | 0.085 | 0.094822 |
| TROVE2 | 0.085 | 0.094938 |
| DIRC2 | 0.085 | 0.095492 |
| C4orf22 | 0.085 | 0.095611 |
| RGS22 | 0.085 | 0.096344 |
| NPHP4 | 0.084 | 0.097993 |
| EXOC6B | 0.084 | 0.098365 |
| SLC28A3 | 0.084 | 0.099005 |
| BAG4 | 0.084 | 0.099654 |
| OR6N1 | 0.084 | 0.099658 |
| SCNN1B | 0.084 | 0.100581 |
| NAGS | 0.084 | 0.100789 |
| METTL16 | 0.083 | 0.101715 |
| CLDN8 | 0.083 | 0.101885 |
| FAM69A | 0.083 | 0.102061 |
| PANK2 | 0.083 | 0.102396 |
| S100A16 | 0.083 | 0.103054 |
| CENPVL3 | 0.083 | 0.103589 |
| KLHL11 | 0.083 | 0.103606 |
| SAMD8 | 0.083 | 0.104515 |
| IREB2 | 0.082 | 0.105359 |
| HOXC11 | 0.082 | 0.105411 |
| CCDC177 | 0.082 | 0.106299 |
| FBXO17 | 0.082 | 0.106552 |
| METTL9 | 0.082 | 0.107806 |
| SPTBN1 | 0.082 | 0.108318 |
| CCDC87 | 0.082 | 0.108586 |
| ZNF490 | 0.081 | 0.109818 |
| NME7 | 0.081 | 0.110363 |
| EVI5L | 0.081 | 0.110493 |
| DUSP19 | 0.081 | 0.110862 |
| NABP1 | 0.081 | 0.111019 |
| ZNF559 | 0.081 | 0.111275 |
| SORBS1 | 0.081 | 0.111532 |
| ZNF619 | 0.081 | 0.112161 |
| OBSL1 | 0.081 | 0.112812 |
| NOD1 | 0.08 | 0.114456 |
| COPB1 | 0.08 | 0.114761 |
| HOXB4 | 0.08 | 0.115053 |
| TNFRSF11A | 0.08 | 0.117273 |
| NUDT3 | 0.08 | 0.117989 |
| PRSS36 | 0.079 | 0.118959 |
| ZNF778 | 0.079 | 0.118961 |
| C6orf52 | 0.079 | 0.120192 |
| FOXN2 | 0.079 | 0.120748 |
| TGFBR3 | 0.079 | 0.121496 |
| ELF2 | 0.079 | 0.121839 |
| WDR17 | 0.079 | 0.122307 |
| SLC9B2 | 0.079 | 0.122929 |
| GPC3 | 0.078 | 0.123609 |
| SPECC1L | 0.078 | 0.124196 |
| NPAT | 0.078 | 0.125433 |
| PSMD9 | 0.078 | 0.126682 |
| ABCG8 | 0.078 | 0.127318 |
| GZMB | 0.077 | 0.128179 |
| SRSF9 | 0.077 | 0.13044 |
| MRPL49 | 0.077 | 0.130583 |
| CCP110 | 0.077 | 0.1315 |
| MAPT | 0.077 | 0.131668 |
| ZNF286B | 0.077 | 0.132054 |
| NRXN2 | 0.076 | 0.133871 |
| TMEM237 | 0.076 | 0.134063 |
| VPS37D | 0.076 | 0.135227 |
| DISP3 | 0.076 | 0.135866 |
| GAD1 | 0.076 | 0.136138 |
| LAMB1 | 0.076 | 0.136704 |
| FAM122C | 0.076 | 0.13699 |
| TSPAN1 | 0.076 | 0.137187 |
| CCDC126 | 0.076 | 0.137983 |
| HERC3 | 0.075 | 0.13829 |
| 3-Mar | 0.075 | 0.139541 |
| PDIA6 | 0.075 | 0.139652 |
| FBP1 | 0.075 | 0.141009 |
| TSPYL4 | 0.075 | 0.141601 |
| ALX1 | 0.075 | 0.142855 |
| TMEM179B | 0.074 | 0.144351 |
| C2orf42 | 0.074 | 0.145003 |
| CRLF3 | 0.074 | 0.145274 |
| ANO5 | 0.074 | 0.147869 |
| ITGB1BP1 | 0.074 | 0.14828 |
| LIPK | 0.073 | 0.149309 |
| DGCR6L | 0.073 | 0.14946 |
| CCNI | 0.073 | 0.149889 |
| ZNF645 | 0.073 | 0.150466 |
| UBTD2 | 0.073 | 0.150825 |
| TMEM39A | 0.073 | 0.151165 |
| ZNF845 | 0.072 | 0.1569 |
| AGO3 | 0.072 | 0.157675 |
| HPGD | 0.072 | 0.157829 |
| TSSK4 | 0.072 | 0.15784 |
| CHMP2B | 0.072 | 0.159094 |
| ELFN2 | 0.072 | 0.159453 |
| DRC1 | 0.072 | 0.159867 |
| SIGLEC15 | 0.071 | 0.161577 |
| HIF3A | 0.071 | 0.162246 |
| CYB5R2 | 0.071 | 0.16333 |
| ZSCAN26 | 0.071 | 0.163538 |
| THAP1 | 0.071 | 0.163942 |
| BBS7 | 0.071 | 0.164122 |
| VEZF1 | 0.07 | 0.166387 |
| INHBE | 0.07 | 0.167085 |
| CA1 | 0.07 | 0.168125 |
| CHMP5 | 0.07 | 0.16939 |
| ZNF549 | 0.07 | 0.171482 |
| AKIRIN2 | 0.07 | 0.171493 |
| ZNF660 | 0.07 | 0.172019 |
| MCMBP | 0.069 | 0.175841 |
| ARL6 | 0.069 | 0.176412 |
| MAP6D1 | 0.069 | 0.176502 |
| KCTD7 | 0.069 | 0.177683 |
| SMURF2 | 0.069 | 0.177855 |
| ZNF821 | 0.068 | 0.178767 |
| LIN52 | 0.068 | 0.179302 |
| GPR55 | 0.068 | 0.17953 |
| IPO11 | 0.068 | 0.181205 |
| CLDN14 | 0.068 | 0.181401 |
| ATP13A4 | 0.068 | 0.18311 |
| AC092835.1 | 0.068 | 0.183703 |
| PAX9 | 0.067 | 0.186762 |
| TRIM16 | 0.067 | 0.188715 |
| VPS13B | 0.067 | 0.188853 |
| ZNF528 | 0.067 | 0.188888 |
| SPRR1A | 0.067 | 0.188981 |
| FAM25A | 0.067 | 0.190293 |
| TNFAIP8L3 | 0.067 | 0.191477 |
| EMC7 | 0.066 | 0.192795 |
| MYBPC1 | 0.066 | 0.193094 |
| UNC13A | 0.066 | 0.193435 |
| AC024270.1 | 0.066 | 0.193584 |
| HACD1 | 0.066 | 0.194232 |
| FNDC11 | 0.066 | 0.194256 |
| EPS8 | 0.066 | 0.194983 |
| MOCS1 | 0.066 | 0.195169 |
| GDAP1L1 | 0.066 | 0.1955 |
| MAST1 | 0.066 | 0.196219 |
| ARCN1 | 0.066 | 0.19628 |
| CNNM1 | 0.066 | 0.198399 |
| DENND6A | 0.065 | 0.199795 |
| ARMC8 | 0.065 | 0.199936 |
| RIMS2 | 0.065 | 0.200219 |
| DYRK4 | 0.065 | 0.200324 |
| KLK7 | 0.065 | 0.201103 |
| ACADL | 0.065 | 0.202369 |
| AP2S1 | 0.065 | 0.203033 |
| PRB2 | 0.065 | 0.203731 |
| TTC36 | 0.065 | 0.204613 |
| KLK11 | 0.064 | 0.205911 |
| ACBD7 | 0.064 | 0.20642 |
| GIMD1 | 0.064 | 0.206596 |
| DPP8 | 0.064 | 0.206811 |
| GPAM | 0.064 | 0.208749 |
| SCG5 | 0.064 | 0.212031 |
| HABP2 | 0.064 | 0.212061 |
| PAPOLB | 0.063 | 0.212864 |
| HIST1H4C | 0.063 | 0.21306 |
| ERC2 | 0.063 | 0.215426 |
| GLS2 | 0.063 | 0.216214 |
| ALDH3B2 | 0.063 | 0.216932 |
| IL20RB | 0.063 | 0.21826 |
| HEBP1 | 0.063 | 0.218941 |
| VAMP4 | 0.063 | 0.219339 |
| FAM90A1 | 0.063 | 0.219454 |
| GIN1 | 0.062 | 0.220457 |
| ZBTB11 | 0.062 | 0.220726 |
| FGF9 | 0.062 | 0.221883 |
| PTPN1 | 0.062 | 0.2249 |
| LIMA1 | 0.062 | 0.225272 |
| OR2C1 | 0.062 | 0.225401 |
| TMEM144 | 0.062 | 0.226143 |
| TMEM218 | 0.062 | 0.226866 |
| FBXO30 | 0.062 | 0.22702 |
| RASD1 | 0.062 | 0.227117 |
| SH3GL1 | 0.061 | 0.227596 |
| EID2 | 0.061 | 0.228809 |
| FAM175A | 0.061 | 0.229183 |
| ZNF586 | 0.061 | 0.229673 |
| KLHDC3 | 0.061 | 0.230797 |
| FAM184A | 0.061 | 0.230832 |
| DNAJC24 | 0.061 | 0.2326 |
| OS9 | 0.061 | 0.2338 |
| ADAM20 | 0.06 | 0.235079 |
| PAX2 | 0.06 | 0.235195 |
| DNAJB14 | 0.06 | 0.235552 |
| SERAC1 | 0.06 | 0.236254 |
| MPL | 0.06 | 0.236264 |
| PPP2R5E | 0.06 | 0.236558 |
| TAF8 | 0.06 | 0.237882 |
| RSBN1 | 0.06 | 0.238035 |
| LIN54 | 0.06 | 0.238833 |
| FAM131A | 0.059 | 0.243146 |
| INSL5 | 0.059 | 0.24443 |
| MIF4GD | 0.059 | 0.245081 |
| INHA | 0.059 | 0.246167 |
| HIST1H4H | 0.059 | 0.246478 |
| TMEM59 | 0.059 | 0.246769 |
| RBSN | 0.059 | 0.247017 |
| SLCO3A1 | 0.059 | 0.24714 |
| HEATR6 | 0.059 | 0.247611 |
| DHRS13 | 0.058 | 0.251664 |
| ACVR2A | 0.058 | 0.252407 |
| NEURL3 | 0.058 | 0.255146 |
| TMSB10 | 0.058 | 0.257086 |
| BCAP29 | 0.058 | 0.258984 |
| USP2 | 0.057 | 0.25977 |
| ZFP37 | 0.057 | 0.260043 |
| LMX1B | 0.057 | 0.260956 |
| ARIH1 | 0.057 | 0.261402 |
| EIF4A3 | 0.057 | 0.261688 |
| UBE2E1 | 0.057 | 0.261768 |
| ABCG5 | 0.057 | 0.262009 |
| STAR | 0.057 | 0.262392 |
| COL9A2 | 0.057 | 0.262842 |
| RPS6KA5 | 0.057 | 0.264745 |
| IFI27L1 | 0.057 | 0.264806 |
| KANSL2 | 0.057 | 0.265433 |
| LRCH4 | 0.057 | 0.266115 |
| HIST1H3D | 0.057 | 0.266123 |
| HEPHL1 | 0.057 | 0.266842 |
| GNA14 | 0.056 | 0.267612 |
| CA8 | 0.056 | 0.269357 |
| AL049779.1 | 0.056 | 0.270127 |
| PRR14L | 0.056 | 0.271272 |
| CCDC127 | 0.056 | 0.273063 |
| ZFYVE19 | 0.056 | 0.274234 |
| EFCC1 | 0.056 | 0.274873 |
| FBXO11 | 0.056 | 0.275311 |
| REXO2 | 0.056 | 0.275423 |
| HAS3 | 0.055 | 0.277487 |
| CREG1 | 0.055 | 0.284672 |
| MAP3K2 | 0.055 | 0.28484 |
| GPR52 | 0.054 | 0.286029 |
| ASTN2 | 0.054 | 0.287179 |
| POLR2B | 0.054 | 0.287238 |
| MYLK3 | 0.054 | 0.287815 |
| USP8 | 0.054 | 0.288314 |
| CAPZA2 | 0.054 | 0.289693 |
| KLK6 | 0.054 | 0.290641 |
| POTEE | 0.054 | 0.292024 |
| ZNF548 | 0.054 | 0.293505 |
| ZNF257 | 0.053 | 0.294437 |
| ASCC1 | 0.053 | 0.294629 |
| MARS | 0.053 | 0.299021 |
| CDC40 | 0.053 | 0.299982 |
| SLC14A2 | 0.053 | 0.301046 |
| ARHGEF37 | 0.053 | 0.301324 |
| LRRTM1 | 0.053 | 0.301549 |
| GNG12 | 0.053 | 0.301679 |
| DNTT | 0.052 | 0.30327 |
| MIER2 | 0.052 | 0.304101 |
| MAP3K10 | 0.052 | 0.305104 |
| SLC9B1 | 0.052 | 0.305351 |
| NLRP2 | 0.052 | 0.305569 |
| HIST1H4D | 0.052 | 0.307495 |
| SLC37A1 | 0.052 | 0.307683 |
| SEMA6A | 0.052 | 0.308188 |
| AP1G1 | 0.052 | 0.308812 |
| OR2T8 | 0.052 | 0.310236 |
| ASAP1 | 0.051 | 0.317916 |
| DLK2 | 0.051 | 0.318351 |
| CXCL17 | 0.051 | 0.318815 |
| TTC9 | 0.051 | 0.319815 |
| FABP7 | 0.05 | 0.323625 |
| PRDX3 | 0.05 | 0.323942 |
| CALML5 | 0.05 | 0.328178 |
| MDH1B | 0.05 | 0.328999 |
| SPEN | 0.05 | 0.330928 |
| SIPA1L1 | 0.049 | 0.332177 |
| HIST1H2BJ | 0.049 | 0.334431 |
| RAPGEF5 | 0.049 | 0.336088 |
| ZNF655 | 0.049 | 0.33696 |
| ARL14EPL | 0.049 | 0.337755 |
| SHMT2 | 0.049 | 0.339067 |
| ATG14 | 0.049 | 0.34019 |
| HIST1H1D | 0.049 | 0.340775 |
| CCER2 | 0.048 | 0.341987 |
| KLHDC2 | 0.048 | 0.350256 |
| DNMT3L | 0.048 | 0.350747 |
| ZNF570 | 0.048 | 0.350841 |
| IFNL3 | 0.048 | 0.351361 |
| OSBPL11 | 0.047 | 0.351498 |
| ZNF14 | 0.047 | 0.352557 |
| SPNS1 | 0.047 | 0.352579 |
| ZNF684 | 0.047 | 0.353734 |
| C12orf76 | 0.047 | 0.355552 |
| METTL14 | 0.047 | 0.357136 |
| OR2W3 | 0.047 | 0.357275 |
| SHPRH | 0.047 | 0.359361 |
| TRIM16L | 0.047 | 0.360779 |
| ZKSCAN3 | 0.047 | 0.361527 |
| PRDM13 | 0.046 | 0.364546 |
| AP3M2 | 0.046 | 0.36594 |
| UHRF1BP1 | 0.046 | 0.367101 |
| TCP10L | 0.046 | 0.368811 |
| SLCO1B3 | 0.046 | 0.369264 |
| CALHM1 | 0.046 | 0.369642 |
| AC120057.3 | 0.046 | 0.370099 |
| VCPKMT | 0.046 | 0.371333 |
| NSUN4 | 0.045 | 0.37319 |
| ASIC4 | 0.045 | 0.373345 |
| EXT2 | 0.045 | 0.374142 |
| HIST1H2BO | 0.045 | 0.375188 |
| KLF7 | 0.045 | 0.376161 |
| C9 | 0.045 | 0.377479 |
| WASF3 | 0.045 | 0.377611 |
| KRTAP1-5 | 0.044 | 0.382829 |
| PPM1E | 0.044 | 0.384327 |
| PLEKHM1 | 0.044 | 0.385103 |
| VSNL1 | 0.044 | 0.38563 |
| TMEM61 | 0.044 | 0.387406 |
| AHSG | 0.044 | 0.388488 |
| DNAJC8 | 0.044 | 0.390863 |
| SMYD1 | 0.043 | 0.396838 |
| RFX6 | 0.043 | 0.397067 |
| LRRN4 | 0.043 | 0.399248 |
| BTBD6 | 0.043 | 0.400653 |
| SCNN1G | 0.043 | 0.402499 |
| PTBP2 | 0.043 | 0.402668 |
| RBPMS | 0.043 | 0.404108 |
| PTP4A2 | 0.042 | 0.404865 |
| PPP1CC | 0.042 | 0.406041 |
| HIST2H4B | 0.042 | 0.406471 |
| MAP4K5 | 0.042 | 0.406915 |
| SKOR1 | 0.042 | 0.407769 |
| EVI5 | 0.042 | 0.408056 |
| HIST1H2BC | 0.042 | 0.408337 |
| PRRG3 | 0.042 | 0.409553 |
| AMH | 0.042 | 0.409603 |
| ARID4A | 0.042 | 0.410189 |
| ZNF426 | 0.042 | 0.412396 |
| TUBA8 | 0.042 | 0.412884 |
| LRRC29 | 0.042 | 0.413096 |
| QSOX1 | 0.042 | 0.413661 |
| DENND2C | 0.042 | 0.413804 |
| DNAJB1 | 0.042 | 0.413898 |
| HSFY2 | 0.041 | 0.418112 |
| AGL | 0.041 | 0.422793 |
| ATP6V0A4 | 0.041 | 0.423987 |
| PRIMPOL | 0.04 | 0.428258 |
| ZBTB21 | 0.04 | 0.429478 |
| LATS1 | 0.04 | 0.430705 |
| ZNF724 | 0.04 | 0.4316 |
| AFG1L | 0.04 | 0.433188 |
| KCNIP3 | 0.04 | 0.433221 |
| SH2D7 | 0.04 | 0.435398 |
| C14orf28 | 0.04 | 0.436064 |
| FBXO9 | 0.04 | 0.437394 |
| LRP3 | 0.04 | 0.437662 |
| TRIM58 | 0.039 | 0.440983 |
| CALHM3 | 0.039 | 0.442703 |
| CSN2 | 0.039 | 0.443101 |
| SIX3 | 0.039 | 0.443222 |
| PEX2 | 0.039 | 0.444248 |
| AC091980.2 | 0.039 | 0.449243 |
| EED | 0.038 | 0.450935 |
| LGI4 | 0.038 | 0.452228 |
| ARF3 | 0.038 | 0.45377 |
| SNURF | 0.038 | 0.454606 |
| LMBRD1 | 0.038 | 0.456261 |
| RPL22L1 | 0.038 | 0.457757 |
| ZNF573 | 0.038 | 0.457819 |
| ANKRD62 | 0.038 | 0.458475 |
| ZNF880 | 0.038 | 0.461045 |
| SMARCAD1 | 0.037 | 0.462928 |
| FOXRED2 | 0.037 | 0.463315 |
| OR2T33 | 0.037 | 0.464115 |
| CIB1 | 0.037 | 0.465797 |
| GNAT3 | 0.037 | 0.466343 |
| MTMR7 | 0.037 | 0.46653 |
| TMPRSS11E | 0.037 | 0.466746 |
| AC010422.6 | 0.037 | 0.47106 |
| ZFP1 | 0.037 | 0.473242 |
| SLC10A7 | 0.037 | 0.473813 |
| SKAP1 | 0.036 | 0.476099 |
| KDM4B | 0.036 | 0.477411 |
| STYK1 | 0.036 | 0.478916 |
| DYNC1LI1 | 0.036 | 0.479843 |
| CCDC116 | 0.036 | 0.481647 |
| RAET1L | 0.036 | 0.482651 |
| UBQLNL | 0.036 | 0.483386 |
| HIST1H2BF | 0.035 | 0.487486 |
| GFOD1 | 0.035 | 0.488036 |
| ETHE1 | 0.035 | 0.48896 |
| PKIA | 0.035 | 0.490199 |
| SIAE | 0.035 | 0.490498 |
| LRCH1 | 0.035 | 0.490869 |
| RFX2 | 0.035 | 0.492155 |
| SNX9 | 0.035 | 0.496253 |
| EML6 | 0.035 | 0.49685 |
| HIST1H2AI | 0.035 | 0.497747 |
| XKR6 | 0.035 | 0.497774 |
| FAM117A | 0.034 | 0.498957 |
| BMP8B | 0.034 | 0.504368 |
| HIST1H2AG | 0.034 | 0.509523 |
| PADI3 | 0.034 | 0.509769 |
| ZFP90 | 0.034 | 0.509861 |
| 11-Mar | 0.034 | 0.509922 |
| KCNS1 | 0.034 | 0.510119 |
| AAGAB | 0.033 | 0.514445 |
| TIGD3 | 0.033 | 0.517244 |
| TUBB1 | 0.033 | 0.521123 |
| PLEKHF2 | 0.033 | 0.521795 |
| CNST | 0.033 | 0.522007 |
| MTUS1 | 0.033 | 0.522518 |
| ZNF383 | 0.032 | 0.524538 |
| PQLC2L | 0.032 | 0.526068 |
| RICTOR | 0.032 | 0.526626 |
| ATG3 | 0.032 | 0.527585 |
| THAP5 | 0.032 | 0.529923 |
| WRB | 0.032 | 0.532055 |
| HIST1H2BG | 0.032 | 0.534063 |
| B4GALT5 | 0.032 | 0.536138 |
| GDPD4 | 0.031 | 0.537028 |
| HAUS2 | 0.031 | 0.540557 |
| ADAMTS15 | 0.031 | 0.541766 |
| ZC3H7A | 0.031 | 0.542313 |
| SLC50A1 | 0.031 | 0.544093 |
| FRMPD1 | 0.031 | 0.546325 |
| C1orf168 | 0.031 | 0.547355 |
| ZNF518B | 0.031 | 0.548004 |
| BRMS1L | 0.031 | 0.548767 |
| MEIOC | 0.03 | 0.550475 |
| OTOS | 0.03 | 0.551192 |
| NEURL1 | 0.03 | 0.551877 |
| ZNF488 | 0.03 | 0.555065 |
| AQP2 | 0.03 | 0.556419 |
| RXRB | 0.03 | 0.558889 |
| GPRC6A | 0.03 | 0.5598 |
| LAMA5 | 0.03 | 0.560883 |
| THEG | 0.03 | 0.56124 |
| CDPF1 | 0.029 | 0.563592 |
| OPA3 | 0.029 | 0.574515 |
| RAB20 | 0.029 | 0.57497 |
| LCN1 | 0.028 | 0.578042 |
| FFAR4 | 0.028 | 0.578062 |
| SSH2 | 0.028 | 0.582353 |
| INO80C | 0.028 | 0.583027 |
| ZC2HC1A | 0.028 | 0.586088 |
| CPOX | 0.028 | 0.586128 |
| HTR3C | 0.028 | 0.588447 |
| HIST1H3H | 0.027 | 0.589674 |
| FAM174B | 0.027 | 0.590202 |
| HIST1H2AD | 0.027 | 0.590737 |
| AC187653.1 | 0.027 | 0.591456 |
| USP37 | 0.027 | 0.593086 |
| PIGK | 0.027 | 0.59387 |
| TNFRSF10D | 0.027 | 0.59455 |
| SERPINB1 | 0.027 | 0.595036 |
| VPS37B | 0.027 | 0.596385 |
| HYAL1 | 0.027 | 0.597356 |
| EHMT1 | 0.027 | 0.598652 |
| HABP4 | 0.027 | 0.600423 |
| MC2R | 0.027 | 0.60136 |
| DNAJC27 | 0.027 | 0.602107 |
| RPAP2 | 0.027 | 0.602921 |
| RBBP4 | 0.026 | 0.603683 |
| TMEM189-UBE2V1 | 0.026 | 0.606593 |
| EXOG | 0.026 | 0.608002 |
| MYH6 | 0.026 | 0.609472 |
| FANCM | 0.026 | 0.609557 |
| SRP72 | 0.026 | 0.610804 |
| DNAJC3 | 0.026 | 0.61295 |
| TNS4 | 0.026 | 0.615631 |
| TKTL1 | 0.026 | 0.615858 |
| EEF2K | 0.025 | 0.617244 |
| ZNF844 | 0.025 | 0.619542 |
| RPTN | 0.025 | 0.622015 |
| PPFIA1 | 0.025 | 0.623008 |
| FGD1 | 0.025 | 0.623904 |
| CEPT1 | 0.025 | 0.62628 |
| MPP5 | 0.025 | 0.628137 |
| GTDC1 | 0.024 | 0.631067 |
| KHDC3L | 0.024 | 0.635882 |
| MLNR | 0.024 | 0.637905 |
| OR14K1 | 0.024 | 0.644365 |
| MAP3K4 | 0.024 | 0.644677 |
| RAB26 | 0.023 | 0.645661 |
| ZNF878 | 0.023 | 0.646363 |
| ARHGEF33 | 0.023 | 0.647171 |
| VASP | 0.023 | 0.647635 |
| RAD1 | 0.023 | 0.64796 |
| DPPA2 | 0.023 | 0.648238 |
| MYO15A | 0.023 | 0.648518 |
| DSG3 | 0.023 | 0.649435 |
| AQP8 | 0.023 | 0.653791 |
| ANKRD33 | 0.023 | 0.655151 |
| DDHD2 | 0.023 | 0.656108 |
| TMPO | 0.022 | 0.659268 |
| PXN | 0.022 | 0.660246 |
| KRTAP16-1 | 0.022 | 0.660323 |
| OR13G1 | 0.022 | 0.663525 |
| KRT34 | 0.022 | 0.672459 |
| DDX3X | 0.021 | 0.673968 |
| HBQ1 | 0.021 | 0.676756 |
| KIAA1324L | 0.021 | 0.679862 |
| MAP4K2 | 0.021 | 0.680553 |
| SP9 | 0.021 | 0.680986 |
| ZNF669 | 0.021 | 0.681181 |
| MMADHC | 0.021 | 0.681368 |
| GTPBP8 | 0.021 | 0.685941 |
| GIF | 0.02 | 0.687672 |
| ATXN3 | 0.02 | 0.69005 |
| COMMD2 | 0.02 | 0.690287 |
| GMPR | 0.02 | 0.694658 |
| HIST2H4A | 0.02 | 0.695436 |
| MFSD11 | 0.02 | 0.699576 |
| FAM216A | 0.02 | 0.701151 |
| NCBP2L | 0.02 | 0.701702 |
| POTEC | 0.02 | 0.701963 |
| DMRTA2 | 0.019 | 0.70281 |
| GRM7 | 0.019 | 0.70361 |
| SYNGR4 | 0.019 | 0.705651 |
| MBTPS1 | 0.019 | 0.711058 |
| AMFR | 0.019 | 0.715147 |
| ZNF853 | 0.019 | 0.715535 |
| ZFYVE21 | 0.019 | 0.716558 |
| PRAMEF12 | 0.018 | 0.717187 |
| C1orf52 | 0.018 | 0.719272 |
| GSTCD | 0.018 | 0.722167 |
| MYBL1 | 0.018 | 0.72332 |
| UBXN2B | 0.018 | 0.729457 |
| CBFB | 0.017 | 0.732393 |
| USP28 | 0.017 | 0.735781 |
| MIGA1 | 0.017 | 0.737501 |
| MUM1 | 0.017 | 0.738093 |
| NELFB | 0.017 | 0.738752 |
| AGBL4 | 0.017 | 0.739725 |
| GAST | 0.017 | 0.74246 |
| ANKRD30B | 0.017 | 0.742741 |
| IRS4 | 0.017 | 0.746055 |
| TAOK1 | 0.016 | 0.74746 |
| ACADVL | 0.016 | 0.747626 |
| BHMG1 | 0.016 | 0.748193 |
| GPATCH11 | 0.016 | 0.749318 |
| TMEM82 | 0.016 | 0.749592 |
| VCP | 0.016 | 0.751985 |
| HSPA2 | 0.016 | 0.752729 |
| CDK19 | 0.016 | 0.753393 |
| HIST1H2BN | 0.016 | 0.753756 |
| HIST1H4E | 0.016 | 0.75413 |
| SLC45A4 | 0.016 | 0.756338 |
| WDR45 | 0.016 | 0.75906 |
| TAS1R2 | 0.015 | 0.76176 |
| NEDD8-MDP1 | 0.015 | 0.76368 |
| ACTR8 | 0.015 | 0.764635 |
| EIF5 | 0.015 | 0.765851 |
| TMEM133 | 0.015 | 0.768581 |
| SNX19 | 0.015 | 0.771559 |
| MOSPD2 | 0.015 | 0.773546 |
| C6orf58 | 0.015 | 0.775916 |
| ITFG1 | 0.014 | 0.777653 |
| MTPN | 0.014 | 0.779871 |
| GABPB1 | 0.014 | 0.78202 |
| SPRTN | 0.014 | 0.785204 |
| HOMER1 | 0.014 | 0.785367 |
| ZNF501 | 0.014 | 0.789368 |
| PITPNB | 0.014 | 0.791112 |
| RALGPS2 | 0.014 | 0.791124 |
| KDM6A | 0.013 | 0.791377 |
| 2-Sep | 0.013 | 0.792838 |
| C12orf50 | 0.013 | 0.794418 |
| DDX23 | 0.013 | 0.795611 |
| ZNF420 | 0.013 | 0.800242 |
| BSCL2 | 0.013 | 0.80204 |
| SBSPON | 0.013 | 0.802384 |
| WBP2 | 0.013 | 0.803096 |
| MATN4 | 0.013 | 0.803629 |
| SOCS4 | 0.013 | 0.803646 |
| C1orf226 | 0.013 | 0.803738 |
| TMEM86B | 0.013 | 0.803977 |
| MYCNOS | 0.013 | 0.805074 |
| CSNK2A3 | 0.012 | 0.808031 |
| KCNF1 | 0.012 | 0.811296 |
| FBXO36 | 0.012 | 0.812226 |
| SPDEF | 0.012 | 0.81399 |
| PRH1 | 0.012 | 0.814133 |
| KLK14 | 0.012 | 0.814909 |
| HTR3E | 0.012 | 0.816016 |
| SLC35G3 | 0.012 | 0.81615 |
| FBXL20 | 0.012 | 0.816236 |
| MSANTD4 | 0.012 | 0.818725 |
| ZNF347 | 0.012 | 0.819179 |
| SMIM13 | 0.011 | 0.82249 |
| DKK1 | 0.011 | 0.822805 |
| ZBTB7C | 0.011 | 0.823647 |
| CCDC157 | 0.011 | 0.823683 |
| APOBR | 0.011 | 0.824062 |
| TRIM50 | 0.011 | 0.828281 |
| TMEM236 | 0.011 | 0.828492 |
| ARHGEF28 | 0.011 | 0.829381 |
| ZYG11B | 0.011 | 0.831164 |
| GPR37 | 0.011 | 0.831826 |
| BPIFB2 | 0.011 | 0.832454 |
| SRRD | 0.011 | 0.834242 |
| C14orf177 | 0.011 | 0.834508 |
| SLC26A9 | 0.011 | 0.834563 |
| AKAP4 | 0.011 | 0.835698 |
| FDXACB1 | 0.011 | 0.836066 |
| UBXN7 | 0.011 | 0.836485 |
| CPSF2 | 0.01 | 0.842555 |
| EXO1 | 0.01 | 0.844186 |
| RP1 | 0.01 | 0.844227 |
| PROCR | 0.01 | 0.846763 |
| HIST1H2AH | 0.01 | 0.851316 |
| IL37 | 0.01 | 0.851563 |
| PPAN-P2RY11 | 0.009 | 0.853778 |
| C4orf46 | 0.009 | 0.85444 |
| EDEM2 | 0.009 | 0.855498 |
| KLHL7 | 0.009 | 0.85596 |
| UPB1 | 0.009 | 0.860341 |
| STX4 | 0.009 | 0.862511 |
| FAM20B | 0.009 | 0.865657 |
| GDE1 | 0.009 | 0.866477 |
| C8orf37 | 0.009 | 0.867094 |
| ZCCHC11 | 0.008 | 0.868698 |
| MPZL1 | 0.008 | 0.869373 |
| OR51M1 | 0.008 | 0.869924 |
| ERCC6L2 | 0.008 | 0.871527 |
| REG3A | 0.008 | 0.873652 |
| CASP8AP2 | 0.008 | 0.878848 |
| POLR3B | 0.007 | 0.883892 |
| PARVB | 0.007 | 0.884497 |
| GPR180 | 0.007 | 0.886018 |
| C4BPB | 0.007 | 0.886739 |
| SCGB2A1 | 0.007 | 0.887774 |
| ENPP1 | 0.007 | 0.888037 |
| POMT2 | 0.007 | 0.895446 |
| PGS1 | 0.007 | 0.896079 |
| ICA1L | 0.007 | 0.896168 |
| FXYD4 | 0.006 | 0.899027 |
| JMY | 0.006 | 0.899046 |
| STAT6 | 0.006 | 0.900079 |
| RERE | 0.006 | 0.90051 |
| RD3L | 0.006 | 0.900713 |
| ZMAT5 | 0.006 | 0.9024 |
| BICDL1 | 0.006 | 0.904995 |
| OTX2 | 0.006 | 0.908779 |
| BIN3 | 0.006 | 0.910544 |
| ING3 | 0.006 | 0.911432 |
| MAGEA9B | 0.006 | 0.912766 |
| KRTAP10-2 | 0.005 | 0.915163 |
| KIF19 | 0.005 | 0.917486 |
| FAM110A | 0.005 | 0.920413 |
| TMOD4 | 0.005 | 0.921045 |
| TMEM206 | 0.005 | 0.922285 |
| GRIN1 | 0.005 | 0.92245 |
| NLRP9 | 0.005 | 0.922678 |
| LRRC14B | 0.005 | 0.922786 |
| GATB | 0.005 | 0.923038 |
| CCDC155 | 0.005 | 0.923421 |
| ART3 | 0.005 | 0.92361 |
| SLC29A1 | 0.005 | 0.925263 |
| TM4SF20 | 0.005 | 0.927419 |
| ROS1 | 0.005 | 0.928265 |
| PCDHGA2 | 0.004 | 0.930386 |
| AC004233.2 | 0.004 | 0.930843 |
| RHBDL3 | 0.004 | 0.940797 |
| CORO1B | 0.004 | 0.94277 |
| GLRA4 | 0.004 | 0.944895 |
| USP31 | 0.003 | 0.94645 |
| WIPF3 | 0.003 | 0.949909 |
| ETV2 | 0.003 | 0.950128 |
| C11orf52 | 0.003 | 0.952654 |
| IQCA1L | 0.003 | 0.95279 |
| OR5AN1 | 0.003 | 0.952916 |
| KRT33A | 0.003 | 0.95763 |
| FGFBP1 | 0.003 | 0.959527 |
| KMT2D | 0.003 | 0.960406 |
| TRIM37 | 0.002 | 0.964429 |
| SIX2 | 0.002 | 0.965435 |
| PAGE4 | 0.002 | 0.967 |
| STK3 | 0.002 | 0.967506 |
| PCDHGB4 | 0.002 | 0.968415 |
| NCOA2 | 0.002 | 0.968515 |
| OR1C1 | 0.002 | 0.970283 |
| ZNF560 | 0.002 | 0.970609 |
| SERPINA11 | 0.002 | 0.972362 |
| MAP3K7 | 0.002 | 0.973677 |
| DNASE2B | 0.001 | 0.979997 |
| EPC2 | 0.001 | 0.981054 |
| ZNF547 | 0.001 | 0.98252 |
| ALDH3B1 | 0.001 | 0.982745 |
| C16orf52 | 0.001 | 0.983501 |
| CDS2 | 0.001 | 0.983555 |
| PPP2R5D | 0.001 | 0.984773 |
| NLRP10 | 0.001 | 0.984994 |
| DCT | 0.001 | 0.987211 |
| OR9A2 | 0.001 | 0.988605 |
| PRAMEF19 | 0.001 | 0.989564 |
| IFITM5 | 0.001 | 0.9902 |
| MROH8 | 0.001 | 0.991574 |
| KRTAP3-1 | 0 | 0.992839 |
| SERPINB6 | 0 | 0.993202 |
| RGP1 | 0 | 0.994082 |
| ZNF266 | 0 | 0.99465 |
| RIMBP2 | 0 | 0.994976 |
| ADGRF1 | 0 | 0.994977 |
| DNAJC12 | 0 | 0.995327 |
| GDAP2 | 0 | 0.995721 |
| KRT33B | 0 | 0.995792 |
| MTRNR2L11 | 0 | 0.996257 |
| TCN1 | 0 | 0.996566 |
| DEGS2 | 0 | 0.996641 |
| TCEANC | 0 | 0.996933 |
| NSUN3 | 0 | 0.997448 |
| POLR2G | 0 | 0.998712 |
| ZNF440 | 0 | 0.998758 |
| DNALI1 | 0 | 0.999618 |
| CNNM4 | -0.001 | 0.978199 |
| WDHD1 | -0.001 | 0.978648 |
| TMEM45B | -0.001 | 0.978728 |
| RGS21 | -0.001 | 0.978782 |
| ITGA9 | -0.001 | 0.978904 |
| UBE3B | -0.001 | 0.97977 |
| RYR2 | -0.001 | 0.983007 |
| CYP17A1 | -0.001 | 0.989793 |
| SHF | -0.001 | 0.991991 |
| MYO5B | -0.002 | 0.961398 |
| OR7G3 | -0.002 | 0.961959 |
| ICE2 | -0.002 | 0.970424 |
| USP46 | -0.003 | 0.945412 |
| NRN1 | -0.003 | 0.945847 |
| SSPO | -0.003 | 0.946117 |
| CYP11B2 | -0.003 | 0.946582 |
| DCAF11 | -0.003 | 0.953551 |
| FEZF1 | -0.003 | 0.955012 |
| CCNJL | -0.003 | 0.955291 |
| FRMPD2 | -0.003 | 0.955428 |
| DCUN1D4 | -0.003 | 0.95853 |
| QRICH2 | -0.003 | 0.959589 |
| ZNF718 | -0.003 | 0.960699 |
| FSIP2 | -0.004 | 0.930401 |
| GP9 | -0.004 | 0.932835 |
| F12 | -0.004 | 0.933493 |
| SCAMP5 | -0.004 | 0.933524 |
| PSKH1 | -0.004 | 0.933839 |
| ZSCAN31 | -0.004 | 0.934307 |
| AMELY | -0.004 | 0.935223 |
| ST6GALNAC1 | -0.004 | 0.940225 |
| VHL | -0.004 | 0.943188 |
| UBE2W | -0.004 | 0.944466 |
| CDKL2 | -0.004 | 0.944973 |
| RNF10 | -0.005 | 0.916563 |
| VASH2 | -0.005 | 0.917103 |
| CYSTM1 | -0.005 | 0.918858 |
| TIPIN | -0.005 | 0.918966 |
| LYZL4 | -0.005 | 0.919649 |
| OR2Z1 | -0.005 | 0.920609 |
| C1QL2 | -0.005 | 0.921466 |
| FREM3 | -0.005 | 0.921808 |
| GLIPR1L2 | -0.005 | 0.924183 |
| SPANXN1 | -0.005 | 0.925104 |
| STIL | -0.005 | 0.926697 |
| INTS4 | -0.005 | 0.927108 |
| DGCR6 | -0.005 | 0.927568 |
| GDF2 | -0.005 | 0.92792 |
| DCLRE1C | -0.006 | 0.901822 |
| PATE3 | -0.006 | 0.902515 |
| C11orf40 | -0.006 | 0.90546 |
| SHQ1 | -0.006 | 0.906229 |
| LEXM | -0.006 | 0.907552 |
| TUBGCP5 | -0.006 | 0.907895 |
| GNG13 | -0.006 | 0.912582 |
| FAM104A | -0.006 | 0.913564 |
| OR5AP2 | -0.006 | 0.914042 |
| LDHA | -0.007 | 0.883495 |
| COX7A2 | -0.007 | 0.884175 |
| HMGXB4 | -0.007 | 0.885519 |
| OR12D3 | -0.007 | 0.886705 |
| DDB1 | -0.007 | 0.887276 |
| XPO6 | -0.007 | 0.887836 |
| MTUS2 | -0.007 | 0.889065 |
| SLC9A3R1 | -0.007 | 0.890064 |
| GABRB2 | -0.007 | 0.890378 |
| ZBTB8B | -0.007 | 0.893335 |
| RAP1GAP | -0.007 | 0.893948 |
| KCNA2 | -0.007 | 0.894048 |
| ZNF879 | -0.007 | 0.894542 |
| ZNF324 | -0.007 | 0.896684 |
| FAM71E2 | -0.008 | 0.870157 |
| HS3ST4 | -0.008 | 0.870245 |
| TWF1 | -0.008 | 0.870694 |
| OR2T1 | -0.008 | 0.870861 |
| DBP | -0.008 | 0.871384 |
| MTDH | -0.008 | 0.873542 |
| DCUN1D1 | -0.008 | 0.874416 |
| SHBG | -0.008 | 0.8759 |
| SEC23B | -0.008 | 0.87592 |
| PRUNE2 | -0.008 | 0.879995 |
| CACNA1C | -0.008 | 0.88059 |
| OR1E1 | -0.008 | 0.882991 |
| PLXNB3 | -0.009 | 0.853655 |
| MOB2 | -0.009 | 0.858611 |
| EIF4ENIF1 | -0.009 | 0.860344 |
| ZNF670 | -0.009 | 0.861366 |
| UQCR10 | -0.009 | 0.862113 |
| ZNF292 | -0.009 | 0.862698 |
| NXF5 | -0.009 | 0.863125 |
| PRAMEF18 | -0.009 | 0.863533 |
| BCAS2 | -0.009 | 0.865223 |
| CLTC | -0.009 | 0.865538 |
| USP25 | -0.009 | 0.866803 |
| ZNF300 | -0.01 | 0.837401 |
| NPTX2 | -0.01 | 0.838871 |
| SHC2 | -0.01 | 0.840547 |
| PHACTR2 | -0.01 | 0.845246 |
| OTUB2 | -0.01 | 0.845457 |
| MMP26 | -0.01 | 0.846052 |
| SMIM1 | -0.01 | 0.846626 |
| FOXD4L6 | -0.01 | 0.847688 |
| PPIP5K2 | -0.01 | 0.8477 |
| ZBTB41 | -0.01 | 0.847907 |
| FDFT1 | -0.01 | 0.848259 |
| UBXN2A | -0.01 | 0.850794 |
| SPP2 | -0.01 | 0.851725 |
| P2RY11 | -0.011 | 0.822867 |
| CISH | -0.011 | 0.826794 |
| RNF26 | -0.011 | 0.829087 |
| C6orf120 | -0.011 | 0.832611 |
| TIMMDC1 | -0.011 | 0.832632 |
| EPHA8 | -0.011 | 0.833031 |
| APBB3 | -0.011 | 0.833283 |
| SPOP | -0.011 | 0.833479 |
| KRTAP2-1 | -0.012 | 0.808263 |
| MKNK2 | -0.012 | 0.810011 |
| UGT2B10 | -0.012 | 0.810249 |
| MFSD4B | -0.012 | 0.81283 |
| MPP6 | -0.012 | 0.813435 |
| RFX1 | -0.012 | 0.814592 |
| KCNV1 | -0.012 | 0.815104 |
| PADI1 | -0.012 | 0.815645 |
| MTRF1L | -0.012 | 0.818338 |
| LYPLA1 | -0.012 | 0.818452 |
| LCLAT1 | -0.012 | 0.819987 |
| RPH3AL | -0.012 | 0.820456 |
| PPP1R1C | -0.013 | 0.792582 |
| RSPO4 | -0.013 | 0.792595 |
| WDCP | -0.013 | 0.797379 |
| OBSCN | -0.013 | 0.798813 |
| OR3A3 | -0.013 | 0.804507 |
| AGMO | -0.013 | 0.805144 |
| CDH7 | -0.013 | 0.806152 |
| NRAS | -0.014 | 0.779083 |
| RMDN2 | -0.014 | 0.781129 |
| TMEM38A | -0.014 | 0.781424 |
| SLITRK6 | -0.014 | 0.784715 |
| GABBR1 | -0.014 | 0.787068 |
| FER1L5 | -0.014 | 0.78843 |
| OR52B2 | -0.014 | 0.789295 |
| KRTAP9-8 | -0.015 | 0.762538 |
| CCNY | -0.015 | 0.763118 |
| CTCF | -0.015 | 0.764027 |
| INS-IGF2 | -0.015 | 0.765474 |
| ZNF710 | -0.015 | 0.766696 |
| APBA1 | -0.015 | 0.766717 |
| TMEM92 | -0.015 | 0.766789 |
| CCDC149 | -0.015 | 0.767756 |
| ZNF764 | -0.015 | 0.768288 |
| PDZD7 | -0.015 | 0.768403 |
| UBE2M | -0.015 | 0.769289 |
| TMEM71 | -0.015 | 0.77053 |
| CCDC140 | -0.015 | 0.771074 |
| EGFR | -0.015 | 0.772181 |
| SUN5 | -0.015 | 0.77461 |
| ABCA4 | -0.016 | 0.746807 |
| MAGEE1 | -0.016 | 0.747113 |
| ITGA2B | -0.016 | 0.749972 |
| NEK4 | -0.016 | 0.750944 |
| CCDC70 | -0.016 | 0.759455 |
| PATE1 | -0.016 | 0.760601 |
| VCX2 | -0.017 | 0.732072 |
| PIH1D1 | -0.017 | 0.732172 |
| KBTBD3 | -0.017 | 0.732664 |
| HGFAC | -0.017 | 0.733734 |
| FBXO3 | -0.017 | 0.73396 |
| CENPL | -0.017 | 0.735576 |
| GINS3 | -0.017 | 0.736358 |
| EPX | -0.017 | 0.737695 |
| FAM169A | -0.017 | 0.737973 |
| TXNDC16 | -0.017 | 0.739982 |
| RIMKLA | -0.017 | 0.741243 |
| TMEM185A | -0.017 | 0.74387 |
| ZNF148 | -0.017 | 0.744838 |
| SMOC1 | -0.017 | 0.745777 |
| GOLGA7 | -0.017 | 0.746003 |
| ZNF639 | -0.017 | 0.74624 |
| XRCC4 | -0.018 | 0.716791 |
| ZKSCAN2 | -0.018 | 0.71794 |
| MCM6 | -0.018 | 0.718039 |
| PSMC2 | -0.018 | 0.719081 |
| SPG21 | -0.018 | 0.720568 |
| ZNF492 | -0.018 | 0.720959 |
| CCDC148 | -0.018 | 0.721256 |
| PHC3 | -0.018 | 0.721318 |
| OTP | -0.018 | 0.721425 |
| TMEM132D | -0.018 | 0.722632 |
| CPB1 | -0.018 | 0.725726 |
| ZCCHC10 | -0.018 | 0.725967 |
| CCDC114 | -0.018 | 0.726849 |
| VPS8 | -0.018 | 0.731368 |
| MAGT1 | -0.019 | 0.703059 |
| ILDR2 | -0.019 | 0.704062 |
| CRKL | -0.019 | 0.7049 |
| MCM10 | -0.019 | 0.711709 |
| POLR2M | -0.019 | 0.712 |
| MYO3B | -0.019 | 0.714124 |
| MCOLN2 | -0.019 | 0.715032 |
| GTF3C1 | -0.019 | 0.715316 |
| KLHL24 | -0.02 | 0.689051 |
| TBC1D8B | -0.02 | 0.690674 |
| PAX3 | -0.02 | 0.691869 |
| CHRNA1 | -0.02 | 0.694814 |
| CLASP2 | -0.02 | 0.697251 |
| H2AFZ | -0.021 | 0.674398 |
| COL27A1 | -0.021 | 0.677786 |
| RAD51C | -0.021 | 0.677935 |
| PON3 | -0.021 | 0.68163 |
| NOL4 | -0.021 | 0.681664 |
| GLDC | -0.021 | 0.686885 |
| GFM2 | -0.022 | 0.659471 |
| KPNA1 | -0.022 | 0.659716 |
| PPIAL4C | -0.022 | 0.660868 |
| CUL5 | -0.022 | 0.661721 |
| ZNF691 | -0.022 | 0.663896 |
| ARL11 | -0.022 | 0.664103 |
| TNNC1 | -0.022 | 0.665003 |
| RPL41 | -0.022 | 0.665046 |
| REN | -0.022 | 0.671332 |
| SMG8 | -0.023 | 0.645028 |
| PHLPP2 | -0.023 | 0.645339 |
| PRKCD | -0.023 | 0.646216 |
| GRAMD1C | -0.023 | 0.648559 |
| ARL17B | -0.023 | 0.651138 |
| SLC22A2 | -0.023 | 0.655924 |
| ZBED9 | -0.024 | 0.632781 |
| DKK4 | -0.024 | 0.634976 |
| ZNF793 | -0.024 | 0.635438 |
| HIST1H4I | -0.024 | 0.638844 |
| HELZ | -0.024 | 0.640208 |
| CDIPT | -0.024 | 0.640515 |
| NR5A2 | -0.025 | 0.617584 |
| ZNF415 | -0.025 | 0.623482 |
| FOXO4 | -0.025 | 0.623855 |
| POGLUT1 | -0.025 | 0.627127 |
| ALPK3 | -0.025 | 0.629042 |
| NRAP | -0.025 | 0.629173 |
| OR8G5 | -0.026 | 0.60585 |
| RHOV | -0.026 | 0.606682 |
| CTSA | -0.026 | 0.611769 |
| CERS4 | -0.026 | 0.61498 |
| LY6G6C | -0.026 | 0.615768 |
| EFL1 | -0.026 | 0.616657 |
| ZBTB5 | -0.026 | 0.616775 |
| SLC35G1 | -0.027 | 0.594347 |
| CNOT9 | -0.027 | 0.595302 |
| LECT2 | -0.027 | 0.596184 |
| C11orf65 | -0.027 | 0.596502 |
| PLA2G4D | -0.027 | 0.599484 |
| MBLAC1 | -0.027 | 0.600068 |
| STXBP4 | -0.027 | 0.601313 |
| CFAP73 | -0.027 | 0.603221 |
| ALDH1L1 | -0.028 | 0.577254 |
| BLOC1S1 | -0.028 | 0.578435 |
| AACS | -0.028 | 0.579444 |
| TMSB4Y | -0.028 | 0.579843 |
| OCM2 | -0.028 | 0.580461 |
| RBBP9 | -0.028 | 0.581444 |
| CCDC82 | -0.028 | 0.586416 |
| ZNF791 | -0.029 | 0.566481 |
| ABI2 | -0.029 | 0.567863 |
| DUSP15 | -0.029 | 0.568335 |
| ZBTB44 | -0.029 | 0.568592 |
| PPP1CB | -0.029 | 0.569946 |
| KLK12 | -0.029 | 0.570423 |
| SF3A3 | -0.029 | 0.572087 |
| PDK2 | -0.029 | 0.572839 |
| PHPT1 | -0.03 | 0.550615 |
| LRRC8E | -0.03 | 0.550683 |
| TTC16 | -0.03 | 0.550846 |
| TNFSF15 | -0.03 | 0.551591 |
| AES | -0.03 | 0.552819 |
| GEMIN2 | -0.03 | 0.55283 |
| SFMBT1 | -0.03 | 0.553577 |
| PARP2 | -0.03 | 0.556315 |
| KRT10 | -0.03 | 0.559398 |
| TNRC6A | -0.03 | 0.560326 |
| OR10H1 | -0.03 | 0.561206 |
| DNAJA2 | -0.03 | 0.561839 |
| EDC3 | -0.03 | 0.562485 |
| DDX59 | -0.03 | 0.562496 |
| ASPDH | -0.031 | 0.53674 |
| SLC25A17 | -0.031 | 0.538937 |
| SNTA1 | -0.031 | 0.539394 |
| STKLD1 | -0.031 | 0.540003 |
| TMEM55A | -0.031 | 0.540329 |
| OPRD1 | -0.031 | 0.542535 |
| GOLGA8G | -0.031 | 0.54362 |
| KRTAP13-2 | -0.031 | 0.544182 |
| ZNF451 | -0.031 | 0.5455 |
| HIST2H3A | -0.031 | 0.548037 |
| SNX21 | -0.032 | 0.524243 |
| PARP6 | -0.032 | 0.52466 |
| ITLN2 | -0.032 | 0.52595 |
| NMNAT2 | -0.032 | 0.526487 |
| PLEKHA3 | -0.032 | 0.53078 |
| BAG1 | -0.033 | 0.511184 |
| RNFT1 | -0.033 | 0.511688 |
| TRPM6 | -0.033 | 0.513672 |
| CHM | -0.033 | 0.514111 |
| TBC1D32 | -0.033 | 0.51471 |
| IKZF5 | -0.033 | 0.515475 |
| ELK1 | -0.033 | 0.516098 |
| TEX19 | -0.033 | 0.518245 |
| VSIG2 | -0.033 | 0.518796 |
| MDH1 | -0.033 | 0.520296 |
| SYNPR | -0.033 | 0.522034 |
| SRSF7 | -0.033 | 0.522035 |
| RNF183 | -0.033 | 0.52242 |
| GSX1 | -0.033 | 0.523236 |
| B3GNT6 | -0.033 | 0.523445 |
| ZNF705E | -0.034 | 0.498635 |
| WFIKKN1 | -0.034 | 0.503425 |
| CEP89 | -0.034 | 0.504552 |
| RIPPLY1 | -0.034 | 0.505715 |
| CA5A | -0.034 | 0.506591 |
| PITPNM3 | -0.034 | 0.507313 |
| TRIM41 | -0.034 | 0.509107 |
| DRD4 | -0.034 | 0.510837 |
| HINT1 | -0.035 | 0.486881 |
| EN2 | -0.035 | 0.487112 |
| PDE7A | -0.035 | 0.491931 |
| POLE3 | -0.035 | 0.493872 |
| MROH6 | -0.035 | 0.494145 |
| KRTAP1-1 | -0.035 | 0.49448 |
| PRSS2 | -0.035 | 0.494801 |
| AC023509.3 | -0.035 | 0.496948 |
| AP002495.2 | -0.036 | 0.474304 |
| CNBD2 | -0.036 | 0.476822 |
| CFAP69 | -0.036 | 0.478296 |
| TRPM7 | -0.036 | 0.478544 |
| ETAA1 | -0.036 | 0.479084 |
| AVIL | -0.036 | 0.482026 |
| RPS6KB1 | -0.036 | 0.48266 |
| USP42 | -0.036 | 0.483165 |
| RSPH1 | -0.036 | 0.483413 |
| ANKRD28 | -0.036 | 0.48495 |
| FABP3 | -0.036 | 0.485913 |
| OAZ3 | -0.037 | 0.463017 |
| FBXO5 | -0.037 | 0.463969 |
| C20orf202 | -0.037 | 0.465848 |
| HSPB9 | -0.037 | 0.468716 |
| ATF7 | -0.037 | 0.472973 |
| ZNF85 | -0.037 | 0.473966 |
| FNIP1 | -0.038 | 0.450278 |
| SH2D6 | -0.038 | 0.452779 |
| TNIK | -0.038 | 0.454559 |
| MRPS2 | -0.038 | 0.456331 |
| DRP2 | -0.038 | 0.457383 |
| TRAF3IP2 | -0.038 | 0.457894 |
| CACNB1 | -0.038 | 0.458714 |
| LINC01207 | -0.038 | 0.45915 |
| NR2C2 | -0.038 | 0.459947 |
| IST1 | -0.038 | 0.460295 |
| RBP1 | -0.038 | 0.460406 |
| MB | -0.039 | 0.442087 |
| CCDC103 | -0.039 | 0.442415 |
| VSIG8 | -0.039 | 0.442953 |
| AC003002.1 | -0.039 | 0.44324 |
| C14orf80 | -0.039 | 0.443674 |
| BRAP | -0.039 | 0.443843 |
| HIST1H3F | -0.039 | 0.444278 |
| GJC2 | -0.039 | 0.444392 |
| PKD1L1 | -0.039 | 0.444552 |
| C19orf24 | -0.039 | 0.445355 |
| VGLL1 | -0.039 | 0.445588 |
| LIPC | -0.039 | 0.448389 |
| KANSL1L | -0.04 | 0.428678 |
| ZNF486 | -0.04 | 0.4314 |
| GINS4 | -0.04 | 0.432239 |
| GTF2E1 | -0.04 | 0.433166 |
| ZSCAN18 | -0.04 | 0.433885 |
| ARHGAP44 | -0.04 | 0.437836 |
| C1D | -0.041 | 0.417279 |
| SNUPN | -0.041 | 0.418594 |
| TTC34 | -0.041 | 0.420455 |
| UBE2A | -0.041 | 0.422151 |
| RBKS | -0.041 | 0.423449 |
| ZNF852 | -0.041 | 0.425446 |
| EFHC1 | -0.042 | 0.404683 |
| ZNF439 | -0.042 | 0.406908 |
| CHRM1 | -0.042 | 0.410244 |
| C22orf39 | -0.042 | 0.410321 |
| TBX20 | -0.042 | 0.410526 |
| HMGA2 | -0.043 | 0.395551 |
| DDIT4L | -0.043 | 0.396562 |
| GTF3C4 | -0.043 | 0.396977 |
| WDR72 | -0.043 | 0.397135 |
| PCGF6 | -0.043 | 0.399304 |
| ZNF575 | -0.043 | 0.400144 |
| DBT | -0.043 | 0.401541 |
| ZNF2 | -0.043 | 0.40324 |
| MLLT10 | -0.043 | 0.403794 |
| AL590560.1 | -0.044 | 0.382777 |
| AC245041.1 | -0.044 | 0.383224 |
| OSBPL5 | -0.044 | 0.383251 |
| TRIM39-RPP21 | -0.044 | 0.383926 |
| ETNK2 | -0.044 | 0.384694 |
| SIKE1 | -0.044 | 0.385227 |
| PTRH1 | -0.044 | 0.385229 |
| CHORDC1 | -0.044 | 0.387348 |
| BARD1 | -0.044 | 0.389615 |
| UNC5CL | -0.044 | 0.390297 |
| KCNV2 | -0.044 | 0.392545 |
| GSDMA | -0.044 | 0.393339 |
| KL | -0.045 | 0.375556 |
| RNASE13 | -0.045 | 0.376582 |
| ABCC4 | -0.045 | 0.37713 |
| AIRE | -0.045 | 0.378334 |
| YEATS2 | -0.045 | 0.378578 |
| DMRTB1 | -0.045 | 0.379706 |
| CDC7 | -0.045 | 0.379713 |
| IL17RD | -0.045 | 0.382155 |
| UMAD1 | -0.045 | 0.382243 |
| ZNF429 | -0.045 | 0.382489 |
| GORASP2 | -0.046 | 0.361654 |
| C1orf141 | -0.046 | 0.363145 |
| NOTO | -0.046 | 0.368988 |
| DDIAS | -0.046 | 0.369873 |
| KNDC1 | -0.046 | 0.370421 |
| SLC25A12 | -0.046 | 0.371126 |
| VWCE | -0.047 | 0.351757 |
| DHX36 | -0.047 | 0.352491 |
| CIB4 | -0.047 | 0.355741 |
| GPRC5D | -0.047 | 0.355949 |
| KLHDC7A | -0.047 | 0.357594 |
| SMLR1 | -0.047 | 0.35973 |
| RETREG2 | -0.047 | 0.360603 |
| L1TD1 | -0.048 | 0.341527 |
| ABHD18 | -0.048 | 0.342332 |
| HNF4G | -0.048 | 0.34238 |
| S100A14 | -0.048 | 0.343242 |
| TNFRSF21 | -0.048 | 0.345206 |
| ACPT | -0.048 | 0.345811 |
| ZKSCAN4 | -0.048 | 0.346731 |
| DMPK | -0.048 | 0.34705 |
| OR6C74 | -0.048 | 0.347116 |
| PHACTR3 | -0.049 | 0.332156 |
| IPP | -0.049 | 0.332475 |
| IER3 | -0.049 | 0.333288 |
| PPP1R3G | -0.049 | 0.335775 |
| FGF21 | -0.049 | 0.33883 |
| CPSF7 | -0.049 | 0.338968 |
| MYNN | -0.049 | 0.3397 |
| PAX6 | -0.049 | 0.33987 |
| LIN9 | -0.049 | 0.340209 |
| BNIP3 | -0.049 | 0.341076 |
| VCPIP1 | -0.05 | 0.322427 |
| FPGT | -0.05 | 0.323765 |
| PTGES2 | -0.05 | 0.325595 |
| KRTAP9-7 | -0.05 | 0.326262 |
| CA6 | -0.05 | 0.327384 |
| SSNA1 | -0.05 | 0.328674 |
| TMEM170B | -0.05 | 0.328757 |
| ABCA5 | -0.05 | 0.329401 |
| VPS50 | -0.05 | 0.330883 |
| SLBP | -0.05 | 0.330937 |
| SHISA9 | -0.051 | 0.313218 |
| PIGH | -0.051 | 0.315017 |
| TUBD1 | -0.051 | 0.316778 |
| ARL6IP1 | -0.051 | 0.317101 |
| POLE2 | -0.051 | 0.317708 |
| LZTFL1 | -0.051 | 0.318273 |
| DDX20 | -0.051 | 0.319758 |
| LRRC8B | -0.051 | 0.320181 |
| PNLIP | -0.051 | 0.321145 |
| SEC22A | -0.052 | 0.303642 |
| DNAH17 | -0.052 | 0.305866 |
| MZF1 | -0.052 | 0.306891 |
| POC1B | -0.052 | 0.307904 |
| ZC3H6 | -0.052 | 0.308384 |
| SLC18A1 | -0.052 | 0.30886 |
| NEURL1B | -0.052 | 0.309033 |
| CAMK1D | -0.052 | 0.310937 |
| ACSM1 | -0.052 | 0.310988 |
| PTCHD4 | -0.053 | 0.293871 |
| HARBI1 | -0.053 | 0.296114 |
| MTSS1 | -0.053 | 0.298291 |
| GOLGA8B | -0.053 | 0.298675 |
| CRYBG2 | -0.053 | 0.30206 |
| CLEC3A | -0.053 | 0.302767 |
| DGKE | -0.054 | 0.285863 |
| NEK11 | -0.054 | 0.286083 |
| NIF3L1 | -0.054 | 0.288288 |
| ZNF556 | -0.054 | 0.288817 |
| TP53TG5 | -0.054 | 0.291997 |
| CS | -0.054 | 0.292198 |
| MSLNL | -0.054 | 0.293154 |
| ARMC3 | -0.055 | 0.277837 |
| PTP4A1 | -0.055 | 0.278892 |
| KRT18 | -0.055 | 0.279578 |
| CRBN | -0.055 | 0.279786 |
| MLANA | -0.055 | 0.280119 |
| ZNF730 | -0.055 | 0.283247 |
| GLB1L | -0.055 | 0.284145 |
| SLC6A7 | -0.056 | 0.268224 |
| PRSS33 | -0.056 | 0.268561 |
| HBS1L | -0.056 | 0.268971 |
| NDUFAF5 | -0.056 | 0.272317 |
| TMC2 | -0.056 | 0.272586 |
| PCMT1 | -0.056 | 0.273261 |
| TMEM210 | -0.056 | 0.273696 |
| METTL27 | -0.057 | 0.260512 |
| CCDC129 | -0.057 | 0.261694 |
| VSTM2L | -0.057 | 0.261864 |
| HS6ST2 | -0.057 | 0.264622 |
| OR52E8 | -0.057 | 0.264637 |
| DDX11 | -0.057 | 0.266996 |
| TRAPPC12 | -0.058 | 0.251729 |
| TRIM28 | -0.058 | 0.25224 |
| KDM3A | -0.058 | 0.254254 |
| FZD9 | -0.058 | 0.255341 |
| GIPC1 | -0.058 | 0.256189 |
| SLC28A1 | -0.058 | 0.257781 |
| BCL11B | -0.058 | 0.257798 |
| UGGT1 | -0.058 | 0.257874 |
| ZNF653 | -0.059 | 0.242954 |
| NMT2 | -0.059 | 0.243032 |
| EDA | -0.059 | 0.244625 |
| CDK6 | -0.059 | 0.245639 |
| FANCD2 | -0.059 | 0.246765 |
| CAPN7 | -0.059 | 0.246886 |
| PXMP2 | -0.059 | 0.247327 |
| DHX9 | -0.059 | 0.248314 |
| GALNT10 | -0.059 | 0.24922 |
| HNRNPUL2-BSCL2 | -0.059 | 0.249464 |
| IPMK | -0.06 | 0.236373 |
| HSF2 | -0.06 | 0.236548 |
| REP15 | -0.06 | 0.2378 |
| TRMT1L | -0.06 | 0.239582 |
| MCRS1 | -0.06 | 0.240038 |
| PNMA5 | -0.06 | 0.240852 |
| LACTB2 | -0.061 | 0.22913 |
| PAGE5 | -0.061 | 0.229502 |
| CENPQ | -0.061 | 0.230393 |
| RSBN1L | -0.061 | 0.232735 |
| QSER1 | -0.061 | 0.232777 |
| HOXD13 | -0.062 | 0.220039 |
| FAM83G | -0.062 | 0.221228 |
| SUSD3 | -0.062 | 0.22198 |
| MASP2 | -0.062 | 0.222091 |
| TMEM54 | -0.062 | 0.222269 |
| FAM204A | -0.062 | 0.22301 |
| ZNF100 | -0.062 | 0.22442 |
| JMJD7 | -0.062 | 0.224757 |
| ARHGAP6 | -0.062 | 0.225543 |
| CLN5 | -0.062 | 0.226488 |
| CCDC169 | -0.062 | 0.226909 |
| RHBDL2 | -0.062 | 0.22694 |
| IGFN1 | -0.062 | 0.227362 |
| ZNF296 | -0.063 | 0.213338 |
| SLC38A9 | -0.063 | 0.213487 |
| STOML3 | -0.063 | 0.213748 |
| C17orf100 | -0.063 | 0.215173 |
| ARL14EP | -0.063 | 0.215226 |
| MYLK4 | -0.063 | 0.215583 |
| SEC61A2 | -0.063 | 0.217052 |
| SOD1 | -0.063 | 0.217612 |
| ABCB8 | -0.063 | 0.217699 |
| MVB12A | -0.064 | 0.206273 |
| HACD3 | -0.064 | 0.207594 |
| KRT83 | -0.064 | 0.207651 |
| CORO7-PAM16 | -0.064 | 0.207858 |
| SNW1 | -0.064 | 0.20819 |
| CWF19L2 | -0.064 | 0.208933 |
| PPIL1 | -0.064 | 0.209171 |
| ERICH1 | -0.064 | 0.209656 |
| ACLY | -0.064 | 0.210418 |
| TAX1BP1 | -0.064 | 0.211057 |
| HELLS | -0.064 | 0.211681 |
| MMS22L | -0.064 | 0.211837 |
| NPIPB7 | -0.064 | 0.21236 |
| PDE9A | -0.065 | 0.198707 |
| TMEM249 | -0.065 | 0.200013 |
| ASIP | -0.065 | 0.200483 |
| INTS2 | -0.065 | 0.20179 |
| KLK4 | -0.065 | 0.202973 |
| PEX26 | -0.065 | 0.204381 |
| SYTL5 | -0.066 | 0.19426 |
| EHMT2 | -0.066 | 0.194637 |
| VDAC3 | -0.066 | 0.195233 |
| GCNT7 | -0.066 | 0.195619 |
| PGBD2 | -0.066 | 0.196375 |
| CD151 | -0.066 | 0.198238 |
| TMEM74B | -0.067 | 0.187201 |
| REXO5 | -0.067 | 0.187495 |
| FAM178B | -0.067 | 0.188676 |
| IKBKB | -0.067 | 0.190715 |
| AZIN1 | -0.067 | 0.191269 |
| UBXN4 | -0.068 | 0.178704 |
| BBC3 | -0.068 | 0.179559 |
| C19orf44 | -0.068 | 0.180265 |
| RAB11FIP2 | -0.068 | 0.180268 |
| NUDT16 | -0.068 | 0.180269 |
| NDNF | -0.068 | 0.180404 |
| TDP2 | -0.068 | 0.181265 |
| ZNF701 | -0.068 | 0.181658 |
| SSX2IP | -0.068 | 0.182516 |
| FGF19 | -0.068 | 0.18263 |
| TPMT | -0.068 | 0.183226 |
| POU2F3 | -0.068 | 0.183648 |
| XRN2 | -0.068 | 0.184249 |
| FADS6 | -0.069 | 0.172781 |
| RAD51B | -0.069 | 0.173494 |
| RCN2 | -0.069 | 0.174277 |
| SP8 | -0.069 | 0.174351 |
| S100A5 | -0.069 | 0.175912 |
| AQP12A | -0.069 | 0.176473 |
| NYX | -0.069 | 0.176507 |
| VWA7 | -0.069 | 0.178181 |
| ATG16L1 | -0.069 | 0.178686 |
| HACL1 | -0.07 | 0.167177 |
| SPDL1 | -0.07 | 0.167336 |
| GTF2H1 | -0.07 | 0.167897 |
| NECTIN4 | -0.07 | 0.169114 |
| DDO | -0.07 | 0.169248 |
| OSGIN2 | -0.07 | 0.169295 |
| CAPN5 | -0.07 | 0.169526 |
| PCSK1N | -0.071 | 0.160841 |
| MUC13 | -0.071 | 0.160848 |
| EFCAB8 | -0.071 | 0.161687 |
| VMP1 | -0.071 | 0.162279 |
| PDE6A | -0.071 | 0.162372 |
| MAT1A | -0.071 | 0.162737 |
| ZNF708 | -0.071 | 0.16328 |
| RBM48 | -0.071 | 0.163418 |
| BRD9 | -0.071 | 0.163635 |
| ZGRF1 | -0.071 | 0.163918 |
| GABRR1 | -0.071 | 0.164019 |
| KANSL1 | -0.071 | 0.164065 |
| PTF1A | -0.071 | 0.165253 |
| RAB5B | -0.071 | 0.165636 |
| UBE2L3 | -0.071 | 0.166026 |
| FBXW9 | -0.072 | 0.156968 |
| TM2D1 | -0.072 | 0.157583 |
| FAM76B | -0.072 | 0.157938 |
| BRIP1 | -0.072 | 0.158176 |
| IFT20 | -0.072 | 0.158177 |
| ATXN2 | -0.072 | 0.158498 |
| PHYHIPL | -0.072 | 0.158887 |
| NEK6 | -0.072 | 0.159115 |
| ERMARD | -0.072 | 0.159182 |
| RPS9 | -0.073 | 0.14923 |
| ILF2 | -0.073 | 0.149713 |
| FANCB | -0.073 | 0.149718 |
| COA1 | -0.073 | 0.150184 |
| CRACR2A | -0.073 | 0.150343 |
| SRI | -0.073 | 0.150637 |
| MME | -0.073 | 0.151109 |
| STAMBPL1 | -0.073 | 0.151865 |
| FUT3 | -0.073 | 0.152081 |
| SMG9 | -0.073 | 0.153592 |
| MOB1B | -0.073 | 0.153664 |
| UBR3 | -0.074 | 0.144192 |
| KLRG2 | -0.074 | 0.145131 |
| ZACN | -0.074 | 0.145874 |
| PLA2R1 | -0.074 | 0.146705 |
| CAPN12 | -0.074 | 0.147482 |
| CPNE3 | -0.074 | 0.148787 |
| DMD | -0.075 | 0.139006 |
| KAZN | -0.075 | 0.139447 |
| GRAMD4 | -0.075 | 0.139758 |
| OFCC1 | -0.075 | 0.140607 |
| PLCXD1 | -0.075 | 0.141744 |
| ZBTB12 | -0.075 | 0.141793 |
| POT1 | -0.075 | 0.142912 |
| POLL | -0.076 | 0.133347 |
| HSPB3 | -0.076 | 0.133391 |
| C2orf82 | -0.076 | 0.133779 |
| GALNT8 | -0.076 | 0.133811 |
| ERN2 | -0.076 | 0.134202 |
| SERINC2 | -0.076 | 0.134699 |
| ZDHHC12 | -0.076 | 0.134917 |
| NXT1 | -0.076 | 0.1351 |
| C14orf105 | -0.076 | 0.135826 |
| TUBB4B | -0.076 | 0.136121 |
| TMEM65 | -0.076 | 0.136713 |
| KLK2 | -0.076 | 0.136877 |
| SCAF1 | -0.076 | 0.137223 |
| EFCAB7 | -0.076 | 0.137266 |
| STARD7 | -0.076 | 0.137361 |
| ERCC3 | -0.077 | 0.128201 |
| SGK3 | -0.077 | 0.128564 |
| DOC2A | -0.077 | 0.130455 |
| HSD3B1 | -0.077 | 0.130572 |
| CLSTN2 | -0.077 | 0.130784 |
| KRT74 | -0.077 | 0.130876 |
| DHFR2 | -0.077 | 0.131843 |
| LSM11 | -0.077 | 0.132995 |
| PEX12 | -0.078 | 0.1233 |
| FASTKD3 | -0.078 | 0.124182 |
| GPN1 | -0.078 | 0.124975 |
| ZNF354A | -0.078 | 0.124992 |
| ZNF578 | -0.078 | 0.125053 |
| THRB | -0.078 | 0.125578 |
| CAPN3 | -0.078 | 0.126137 |
| KRT75 | -0.078 | 0.127207 |
| CLEC18B | -0.079 | 0.118493 |
| SLC25A47 | -0.079 | 0.118996 |
| STIP1 | -0.079 | 0.11916 |
| C5orf52 | -0.079 | 0.119921 |
| SPAST | -0.079 | 0.121664 |
| SNX16 | -0.079 | 0.121727 |
| CELSR1 | -0.079 | 0.122025 |
| KLK3 | -0.079 | 0.122128 |
| CCNJ | -0.079 | 0.122271 |
| DCDC2 | -0.079 | 0.122337 |
| SLC20A2 | -0.079 | 0.123024 |
| ADAL | -0.079 | 0.123104 |
| DRG1 | -0.08 | 0.114618 |
| ZNF524 | -0.08 | 0.114741 |
| DOK4 | -0.08 | 0.115296 |
| AKTIP | -0.08 | 0.116194 |
| RNF187 | -0.08 | 0.116417 |
| LRRIQ4 | -0.08 | 0.116844 |
| EDC4 | -0.08 | 0.117388 |
| ABCA13 | -0.081 | 0.11014 |
| RGS9BP | -0.081 | 0.111174 |
| THUMPD1 | -0.081 | 0.111459 |
| LYPD6B | -0.081 | 0.113781 |
| ZNF747 | -0.081 | 0.113861 |
| ZNF93 | -0.082 | 0.105312 |
| HNRNPA0 | -0.082 | 0.106076 |
| SPCS1 | -0.082 | 0.106332 |
| HIC2 | -0.082 | 0.10688 |
| DCP1B | -0.082 | 0.107296 |
| ST6GAL1 | -0.082 | 0.108054 |
| RASSF3 | -0.082 | 0.108514 |
| CACNA2D2 | -0.082 | 0.108594 |
| ZNF579 | -0.083 | 0.101359 |
| ZNF497 | -0.083 | 0.101664 |
| KCNT1 | -0.083 | 0.102129 |
| ANAPC10 | -0.083 | 0.102764 |
| CCZ1B | -0.083 | 0.103703 |
| PIGL | -0.084 | 0.097044 |
| ISPD | -0.084 | 0.097706 |
| PYGO2 | -0.084 | 0.09779 |
| LHFPL3 | -0.084 | 0.098303 |
| DNASE1L2 | -0.084 | 0.099493 |
| SLC35B1 | -0.085 | 0.093547 |
| TBPL1 | -0.085 | 0.093945 |
| PAX7 | -0.085 | 0.094732 |
| KAAG1 | -0.085 | 0.095791 |
| ZNF839 | -0.085 | 0.096128 |
| RAD21 | -0.086 | 0.089338 |
| SMAD9 | -0.086 | 0.091129 |
| HINT2 | -0.086 | 0.091649 |
| ZBED8 | -0.086 | 0.092609 |
| CA3 | -0.087 | 0.08633 |
| PRIM2 | -0.087 | 0.087241 |
| SMTNL2 | -0.087 | 0.087485 |
| CFAP20 | -0.087 | 0.087567 |
| LRRC57 | -0.087 | 0.087995 |
| RELL2 | -0.087 | 0.088263 |
| ADIPOR2 | -0.087 | 0.088412 |
| TPD52L2 | -0.087 | 0.088497 |
| B4GALT4 | -0.088 | 0.082372 |
| SLC22A31 | -0.088 | 0.082395 |
| MRPS10 | -0.088 | 0.082797 |
| ZNF433 | -0.089 | 0.079238 |
| ZNF200 | -0.089 | 0.079243 |
| GRHL3 | -0.089 | 0.080287 |
| OR13J1 | -0.089 | 0.081376 |
| DOK7 | -0.089 | 0.082002 |
| PLK4 | -0.09 | 0.075562 |
| PDIK1L | -0.09 | 0.07621 |
| SLC2A12 | -0.09 | 0.076554 |
| DHX57 | -0.09 | 0.076884 |
| NPY4R | -0.09 | 0.077139 |
| TMEM131 | -0.09 | 0.077657 |
| DCLRE1A | -0.09 | 0.077834 |
| PRAC1 | -0.09 | 0.078019 |
| CIPC | -0.09 | 0.078055 |
| KIAA1522 | -0.09 | 0.078316 |
| FAM91A1 | -0.09 | 0.078402 |
| CCDC60 | -0.091 | 0.072203 |
| ZNF813 | -0.091 | 0.072486 |
| CENPBD1 | -0.091 | 0.073319 |
| LEMD1 | -0.091 | 0.074265 |
| C9orf57 | -0.091 | 0.074434 |
| BSG | -0.091 | 0.074445 |
| GDF10 | -0.091 | 0.074545 |
| CNOT1 | -0.091 | 0.074622 |
| TCP1 | -0.091 | 0.075064 |
| SLC9A4 | -0.092 | 0.069367 |
| THAP9 | -0.092 | 0.069411 |
| PYM1 | -0.092 | 0.070014 |
| FAM222A | -0.092 | 0.070522 |
| ZNF43 | -0.092 | 0.07074 |
| ZNF784 | -0.092 | 0.071071 |
| MYBBP1A | -0.092 | 0.071124 |
| PRRG2 | -0.092 | 0.072016 |
| AMOT | -0.093 | 0.066157 |
| CDH26 | -0.093 | 0.067641 |
| TAF2 | -0.093 | 0.067887 |
| TSTD3 | -0.093 | 0.068017 |
| C1QTNF3-AMACR | -0.093 | 0.06841 |
| MCM4 | -0.093 | 0.068649 |
| PLAG1 | -0.094 | 0.063432 |
| VMAC | -0.094 | 0.063519 |
| CES3 | -0.094 | 0.06378 |
| ZFP30 | -0.094 | 0.064208 |
| TRRAP | -0.094 | 0.064624 |
| SNX13 | -0.094 | 0.064982 |
| CYP20A1 | -0.094 | 0.065405 |
| KRTAP3-2 | -0.095 | 0.061527 |
| TACC3 | -0.095 | 0.061841 |
| TBC1D24 | -0.095 | 0.062173 |
| MRPS24 | -0.095 | 0.0623 |
| AQP6 | -0.095 | 0.062494 |
| MTF2 | -0.095 | 0.06269 |
| HSD17B8 | -0.095 | 0.062804 |
| C2orf66 | -0.095 | 0.062878 |
| RALA | -0.095 | 0.06308 |
| PCDH19 | -0.095 | 0.063148 |
| OCIAD2 | -0.095 | 0.063212 |
| C11orf53 | -0.096 | 0.057891 |
| ORC4 | -0.096 | 0.058158 |
| ZNF713 | -0.096 | 0.058234 |
| ZNF738 | -0.096 | 0.058262 |
| SLC23A1 | -0.096 | 0.058781 |
| BCAS4 | -0.096 | 0.059418 |
| RABL3 | -0.096 | 0.060108 |
| CES2 | -0.096 | 0.060525 |
| PCK1 | -0.097 | 0.055413 |
| TMEM143 | -0.097 | 0.05547 |
| C16orf87 | -0.097 | 0.055579 |
| NAA60 | -0.097 | 0.05642 |
| TEFM | -0.097 | 0.056422 |
| NINL | -0.097 | 0.057011 |
| STRN4 | -0.097 | 0.05706 |
| PDS5B | -0.098 | 0.053077 |
| C19orf68 | -0.098 | 0.053243 |
| AMIGO1 | -0.098 | 0.053385 |
| SLC25A6 | -0.098 | 0.05346 |
| CEP44 | -0.098 | 0.053875 |
| HSPBP1 | -0.098 | 0.054159 |
| ORMDL3 | -0.098 | 0.054248 |
| E2F6 | -0.098 | 0.05446 |
| C2CD4D | -0.098 | 0.054837 |
| TBC1D22B | -0.098 | 0.054913 |
| LPA | -0.099 | 0.050855 |
| ZNF675 | -0.099 | 0.050962 |
| TMUB2 | -0.099 | 0.051939 |
| DUSP28 | -0.099 | 0.052313 |
| ARFGAP1 | -0.099 | 0.052388 |
| ARHGEF7 | -0.099 | 0.052682 |
| USP49 | -0.099 | 0.052784 |
| ZSCAN12 | -0.1 | 0.048949 |
| NGLY1 | -0.1 | 0.049308 |
| SLC25A32 | -0.1 | 0.049574 |
| RFC2 | -0.1 | 0.049729 |
| FRK | -0.1 | 0.050025 |
| TMEM99 | -0.1 | 0.050339 |
| CCNE2 | -0.101 | 0.046122 |
| METTL17 | -0.101 | 0.046826 |
| CRYM | -0.101 | 0.047009 |
| SPINT1 | -0.101 | 0.04703 |
| SCNN1A | -0.101 | 0.047593 |
| PLEKHA1 | -0.101 | 0.047616 |
| EVX1 | -0.101 | 0.047704 |
| CNKSR3 | -0.101 | 0.047744 |
| KRTAP5-8 | -0.101 | 0.048042 |
| EFCAB6 | -0.102 | 0.044107 |
| CFAP74 | -0.102 | 0.045463 |
| DNAJC6 | -0.102 | 0.045474 |
| WDR55 | -0.102 | 0.045501 |
| SLC16A13 | -0.102 | 0.045553 |
| PTPRB | -0.102 | 0.04584 |
| CDKN1B | -0.103 | 0.042017 |
| NUP155 | -0.103 | 0.042051 |
| NAF1 | -0.103 | 0.042448 |
| SLC12A2 | -0.103 | 0.042942 |
| CFAP36 | -0.103 | 0.04304 |
| ASB12 | -0.103 | 0.043089 |
| SPTBN5 | -0.103 | 0.043204 |
| ZNF493 | -0.103 | 0.043218 |
| CAMKV | -0.103 | 0.043448 |
| FIZ1 | -0.103 | 0.04352 |
| MRPS27 | -0.103 | 0.043539 |
| FGF20 | -0.104 | 0.039995 |
| GPI | -0.104 | 0.040729 |
| ZNF334 | -0.104 | 0.041331 |
| PLG | -0.104 | 0.041448 |
| ZNF90 | -0.104 | 0.041528 |
| KCTD3 | -0.105 | 0.038084 |
| AP5Z1 | -0.105 | 0.038391 |
| PLS1 | -0.105 | 0.03858 |
| HSPB11 | -0.105 | 0.03938 |
| LDAH | -0.105 | 0.039583 |
| FAAP24 | -0.105 | 0.039617 |
| USP41 | -0.105 | 0.039864 |
| RCC2 | -0.106 | 0.036566 |
| LTBR | -0.106 | 0.036609 |
| C7orf43 | -0.106 | 0.036802 |
| C17orf80 | -0.106 | 0.037016 |
| ADH6 | -0.106 | 0.037538 |
| LINC00238 | -0.106 | 0.037795 |
| DPP10 | -0.107 | 0.034647 |
| BIRC5 | -0.107 | 0.034696 |
| UGT1A8 | -0.107 | 0.035394 |
| GALNT4 | -0.107 | 0.035756 |
| ATOH8 | -0.107 | 0.035912 |
| NAPB | -0.108 | 0.032942 |
| NEU1 | -0.108 | 0.032997 |
| POLR2C | -0.108 | 0.033008 |
| CWH43 | -0.108 | 0.033462 |
| NPB | -0.108 | 0.033809 |
| METTL6 | -0.108 | 0.03393 |
| 9-Mar | -0.108 | 0.034262 |
| KLK15 | -0.109 | 0.031333 |
| HOXD12 | -0.109 | 0.031526 |
| ANKS1A | -0.109 | 0.031823 |
| RASL10B | -0.109 | 0.031858 |
| HSPBAP1 | -0.109 | 0.032298 |
| ARFGEF3 | -0.109 | 0.03259 |
| ZNF665 | -0.11 | 0.029814 |
| INS | -0.11 | 0.030149 |
| FAM220A | -0.11 | 0.030366 |
| SUV39H2 | -0.11 | 0.030669 |
| PIPOX | -0.11 | 0.030686 |
| RPL35 | -0.11 | 0.030785 |
| ARL2 | -0.11 | 0.030793 |
| PRPF39 | -0.11 | 0.031138 |
| EIF4B | -0.111 | 0.028643 |
| KIAA1429 | -0.111 | 0.028918 |
| STAU2 | -0.111 | 0.028923 |
| POLR3F | -0.111 | 0.029057 |
| C7orf25 | -0.111 | 0.029452 |
| UTP18 | -0.111 | 0.029654 |
| AC012651.1 | -0.111 | 0.02972 |
| PALB2 | -0.112 | 0.027252 |
| VDR | -0.112 | 0.027381 |
| ATP6V0A1 | -0.112 | 0.027381 |
| TCP10 | -0.112 | 0.027535 |
| C11orf57 | -0.112 | 0.027661 |
| TXNRD3 | -0.112 | 0.027836 |
| C19orf57 | -0.112 | 0.027872 |
| ZDHHC11B | -0.113 | 0.025671 |
| DLEC1 | -0.113 | 0.025772 |
| NUDT21 | -0.113 | 0.025885 |
| SLC33A1 | -0.113 | 0.026082 |
| SUMF2 | -0.113 | 0.026565 |
| SRD5A1 | -0.113 | 0.026746 |
| ASNSD1 | -0.114 | 0.024351 |
| ADGRG2 | -0.114 | 0.024938 |
| ZNF487 | -0.114 | 0.02507 |
| COPZ1 | -0.114 | 0.025112 |
| PPP2R1A | -0.114 | 0.0254 |
| NUP93 | -0.114 | 0.025431 |
| BRSK2 | -0.115 | 0.023245 |
| AC087289.3 | -0.115 | 0.023287 |
| OIT3 | -0.115 | 0.023541 |
| ERI2 | -0.115 | 0.023649 |
| INF2 | -0.115 | 0.023789 |
| DDX19A | -0.115 | 0.023901 |
| ACTR3C | -0.116 | 0.022045 |
| NBPF6 | -0.116 | 0.022674 |
| ZNF335 | -0.117 | 0.020883 |
| MTO1 | -0.117 | 0.021122 |
| TSPAN8 | -0.117 | 0.021258 |
| NEDD4 | -0.117 | 0.021599 |
| C8orf74 | -0.117 | 0.021684 |
| MSX1 | -0.117 | 0.021723 |
| RPL10 | -0.117 | 0.021839 |
| F10 | -0.118 | 0.019781 |
| NDRG2 | -0.118 | 0.019825 |
| ABCE1 | -0.118 | 0.019962 |
| ZNF506 | -0.118 | 0.020058 |
| ERP29 | -0.118 | 0.020369 |
| WDSUB1 | -0.119 | 0.018755 |
| NUP205 | -0.119 | 0.018865 |
| XPNPEP2 | -0.119 | 0.018989 |
| C14orf166 | -0.119 | 0.019007 |
| GMDS | -0.119 | 0.019068 |
| RAB2A | -0.119 | 0.019165 |
| KCNQ4 | -0.119 | 0.019181 |
| ZBTB24 | -0.119 | 0.01934 |
| NDUFA3 | -0.119 | 0.019388 |
| PSME3 | -0.12 | 0.017738 |
| APOM | -0.12 | 0.017874 |
| ALKBH8 | -0.12 | 0.018016 |
| PLPBP | -0.12 | 0.018083 |
| HR | -0.12 | 0.018298 |
| SELENOW | -0.12 | 0.018507 |
| SUZ12 | -0.12 | 0.018663 |
| TMEM67 | -0.121 | 0.01679 |
| TRMT61B | -0.121 | 0.016792 |
| MED6 | -0.121 | 0.016949 |
| TDRKH | -0.121 | 0.017075 |
| TMEM98 | -0.121 | 0.017136 |
| ST7L | -0.121 | 0.017481 |
| GNGT1 | -0.122 | 0.016029 |
| NANP | -0.122 | 0.016215 |
| COX4I1 | -0.122 | 0.016297 |
| CIT | -0.122 | 0.016328 |
| GTF2F1 | -0.122 | 0.016495 |
| SLC48A1 | -0.122 | 0.016518 |
| PPP1R9A | -0.122 | 0.016572 |
| AC007906.2 | -0.122 | 0.016597 |
| BLOC1S5 | -0.122 | 0.01676 |
| GTPBP6 | -0.123 | 0.015468 |
| C21orf2 | -0.123 | 0.015482 |
| SRSF3 | -0.124 | 0.014491 |
| MID1IP1 | -0.124 | 0.014542 |
| BCL2L14 | -0.124 | 0.014579 |
| FANK1 | -0.124 | 0.014808 |
| KLHL29 | -0.124 | 0.01483 |
| CTNNA1 | -0.124 | 0.014869 |
| RLBP1 | -0.124 | 0.01502 |
| CWC25 | -0.125 | 0.013596 |
| ATAD3C | -0.125 | 0.013711 |
| SLC15A1 | -0.125 | 0.013734 |
| PARP16 | -0.125 | 0.013821 |
| ETFB | -0.125 | 0.01397 |
| F7 | -0.125 | 0.014016 |
| NDFIP2 | -0.125 | 0.014058 |
| SLC1A7 | -0.125 | 0.014167 |
| MFSD10 | -0.125 | 0.01417 |
| RLN1 | -0.125 | 0.014215 |
| ANKFN1 | -0.126 | 0.012821 |
| UBA5 | -0.126 | 0.012899 |
| CASC3 | -0.126 | 0.013082 |
| DECR1 | -0.126 | 0.013276 |
| CCDC15 | -0.126 | 0.01347 |
| DNAJC22 | -0.127 | 0.012132 |
| RNLS | -0.127 | 0.012216 |
| SPRY2 | -0.127 | 0.012238 |
| TAF1B | -0.127 | 0.012389 |
| PLEKHG3 | -0.127 | 0.0124 |
| DIS3 | -0.127 | 0.012448 |
| RPL18 | -0.127 | 0.012464 |
| MAMSTR | -0.127 | 0.012569 |
| ABHD12 | -0.128 | 0.011451 |
| NEPRO | -0.128 | 0.011462 |
| TRIOBP | -0.128 | 0.011471 |
| GDF15 | -0.128 | 0.011477 |
| RBM12 | -0.128 | 0.011515 |
| TMEM211 | -0.128 | 0.011597 |
| FZD10 | -0.128 | 0.011642 |
| THAP6 | -0.128 | 0.011936 |
| DSG4 | -0.128 | 0.012015 |
| WWOX | -0.129 | 0.010868 |
| RHOT1 | -0.129 | 0.010888 |
| TYW5 | -0.129 | 0.010906 |
| ZNF737 | -0.129 | 0.011074 |
| BCL2L1 | -0.129 | 0.011372 |
| SERPINA10 | -0.13 | 0.010261 |
| TSSK6 | -0.13 | 0.010494 |
| C2orf49 | -0.13 | 0.010498 |
| UCKL1 | -0.13 | 0.010501 |
| C19orf48 | -0.13 | 0.01056 |
| FBXO48 | -0.13 | 0.010624 |
| GAB1 | -0.131 | 0.009614 |
| KIF16B | -0.131 | 0.009707 |
| PSMD3 | -0.131 | 0.009767 |
| ZNF254 | -0.131 | 0.009804 |
| PLA2G12B | -0.131 | 0.009852 |
| TUBA4A | -0.131 | 0.009946 |
| ZFP69B | -0.131 | 0.010117 |
| ZFP64 | -0.132 | 0.009108 |
| RPS19 | -0.132 | 0.009112 |
| HM13 | -0.132 | 0.009308 |
| CCDC138 | -0.132 | 0.009325 |
| PKLR | -0.132 | 0.009561 |
| IGFBP2 | -0.132 | 0.009563 |
| HSDL1 | -0.133 | 0.0086 |
| VPS25 | -0.133 | 0.008626 |
| TMEM53 | -0.133 | 0.00865 |
| DGKQ | -0.133 | 0.00871 |
| EPHX1 | -0.133 | 0.008841 |
| IVD | -0.133 | 0.008864 |
| HOXA1 | -0.133 | 0.009029 |
| FMO4 | -0.134 | 0.008141 |
| FASTK | -0.134 | 0.008238 |
| C8orf76 | -0.134 | 0.008241 |
| WLS | -0.134 | 0.008248 |
| LRIG2 | -0.134 | 0.008356 |
| SETDB2 | -0.134 | 0.008376 |
| CES1 | -0.134 | 0.00841 |
| SLC13A4 | -0.134 | 0.008436 |
| NANS | -0.134 | 0.008456 |
| LAMTOR2 | -0.134 | 0.008459 |
| ACSS1 | -0.134 | 0.008518 |
| XAGE3 | -0.135 | 0.007723 |
| TMCC1 | -0.135 | 0.007738 |
| RNF223 | -0.135 | 0.00787 |
| NUTM2G | -0.135 | 0.007997 |
| KMT5B | -0.135 | 0.007998 |
| THAP2 | -0.135 | 0.008009 |
| WFS1 | -0.135 | 0.00802 |
| COLCA2 | -0.135 | 0.008056 |
| VDAC2 | -0.136 | 0.007199 |
| KAT7 | -0.136 | 0.00735 |
| PTDSS1 | -0.136 | 0.007415 |
| ATAD5 | -0.136 | 0.007462 |
| RGR | -0.136 | 0.007579 |
| ZNF34 | -0.137 | 0.006767 |
| CCNL1 | -0.137 | 0.006784 |
| HSF2BP | -0.137 | 0.006899 |
| EPOP | -0.137 | 0.006907 |
| RFWD3 | -0.137 | 0.00701 |
| MRPL11 | -0.137 | 0.007036 |
| AL049844.1 | -0.138 | 0.006375 |
| MFSD8 | -0.138 | 0.006425 |
| AC092718.3 | -0.138 | 0.006445 |
| HCN1 | -0.138 | 0.006529 |
| MRPL47 | -0.138 | 0.006565 |
| GTF3C3 | -0.138 | 0.006605 |
| MANBAL | -0.138 | 0.006647 |
| GLYATL1 | -0.138 | 0.006729 |
| WIF1 | -0.139 | 0.005995 |
| FOPNL | -0.139 | 0.00608 |
| C15orf41 | -0.139 | 0.006106 |
| AASDHPPT | -0.139 | 0.006151 |
| CDC23 | -0.139 | 0.006261 |
| RTN3 | -0.139 | 0.006294 |
| PCLO | -0.14 | 0.005671 |
| PLOD3 | -0.14 | 0.005704 |
| IER2 | -0.14 | 0.005829 |
| TBC1D31 | -0.14 | 0.005858 |
| RFXANK | -0.141 | 0.005322 |
| PRKDC | -0.141 | 0.005344 |
| PMS2 | -0.141 | 0.005357 |
| ZNF674 | -0.141 | 0.005556 |
| UBR5 | -0.141 | 0.005561 |
| BLM | -0.141 | 0.005582 |
| YY1 | -0.141 | 0.005604 |
| KCTD18 | -0.142 | 0.005096 |
| PPP2R2C | -0.142 | 0.005155 |
| MTHFD2L | -0.142 | 0.005194 |
| CEP57 | -0.142 | 0.005236 |
| METTL2A | -0.142 | 0.005236 |
| STOX1 | -0.142 | 0.005264 |
| TRPM5 | -0.142 | 0.005268 |
| OSER1 | -0.143 | 0.004692 |
| CACNB3 | -0.143 | 0.004705 |
| ZNF20 | -0.143 | 0.004751 |
| CTH | -0.143 | 0.004763 |
| NEU3 | -0.143 | 0.004787 |
| MCM8 | -0.143 | 0.004918 |
| H2AFV | -0.143 | 0.004935 |
| PIGW | -0.144 | 0.004442 |
| C2orf76 | -0.144 | 0.004454 |
| SLC4A1AP | -0.144 | 0.004485 |
| POLR2K | -0.144 | 0.004519 |
| DPP3 | -0.144 | 0.00454 |
| ALDH3A1 | -0.144 | 0.004614 |
| ABCC10 | -0.145 | 0.004288 |
| TFDP1 | -0.145 | 0.004313 |
| IGSF23 | -0.145 | 0.004381 |
| CKB | -0.146 | 0.003881 |
| PSMD11 | -0.146 | 0.003886 |
| NUP35 | -0.146 | 0.00392 |
| PDE8A | -0.146 | 0.003948 |
| MLH1 | -0.146 | 0.003965 |
| KBTBD6 | -0.146 | 0.00399 |
| ACTL6A | -0.146 | 0.003996 |
| SMARCE1 | -0.146 | 0.004028 |
| SLX4IP | -0.146 | 0.004039 |
| PUS10 | -0.147 | 0.003652 |
| TTC30B | -0.147 | 0.003791 |
| EPHB4 | -0.147 | 0.003796 |
| BAX | -0.148 | 0.003512 |
| XPNPEP3 | -0.148 | 0.00353 |
| UBA2 | -0.148 | 0.003553 |
| CEP152 | -0.148 | 0.003567 |
| URGCP-MRPS24 | -0.148 | 0.003577 |
| RBM45 | -0.148 | 0.003583 |
| EFTUD2 | -0.148 | 0.003621 |
| ATP6V1C2 | -0.149 | 0.003203 |
| KMT5A | -0.149 | 0.003256 |
| ABHD1 | -0.149 | 0.003352 |
| NAT2 | -0.149 | 0.003373 |
| DDX54 | -0.15 | 0.003118 |
| MRPS23 | -0.15 | 0.00313 |
| MED30 | -0.15 | 0.003188 |
| RASAL1 | -0.15 | 0.003192 |
| CAMTA1 | -0.151 | 0.00283 |
| NME6 | -0.151 | 0.002944 |
| CEBPA | -0.152 | 0.002631 |
| SYNCRIP | -0.152 | 0.002668 |
| GAN | -0.152 | 0.002707 |
| KCTD13 | -0.152 | 0.002738 |
| KIF21B | -0.152 | 0.00274 |
| REEP1 | -0.152 | 0.002748 |
| CUL1 | -0.153 | 0.002493 |
| SPATA12 | -0.153 | 0.002511 |
| C1orf109 | -0.153 | 0.002536 |
| PPA1 | -0.153 | 0.002566 |
| MAGEF1 | -0.153 | 0.002574 |
| ANAPC4 | -0.153 | 0.002602 |
| DDN | -0.154 | 0.002334 |
| PCIF1 | -0.154 | 0.002363 |
| SYNE4 | -0.154 | 0.002376 |
| SRPK3 | -0.154 | 0.002397 |
| PGAP1 | -0.154 | 0.00244 |
| RANBP17 | -0.155 | 0.002182 |
| SENP2 | -0.155 | 0.002214 |
| DSCC1 | -0.155 | 0.002229 |
| LGALS4 | -0.155 | 0.002244 |
| ASXL1 | -0.155 | 0.002254 |
| SH3TC2 | -0.155 | 0.002271 |
| TRPC4AP | -0.155 | 0.002274 |
| BRPF3 | -0.155 | 0.002278 |
| PCOTH | -0.155 | 0.002279 |
| GRPR | -0.156 | 0.002037 |
| DBNDD2 | -0.156 | 0.002086 |
| MSH3 | -0.156 | 0.002111 |
| SEC14L4 | -0.156 | 0.002145 |
| PXYLP1 | -0.157 | 0.001892 |
| MYCL | -0.157 | 0.001892 |
| TWISTNB | -0.157 | 0.001898 |
| ZNF681 | -0.157 | 0.001945 |
| SLC13A3 | -0.157 | 0.002015 |
| RPS6 | -0.158 | 0.001762 |
| TBC1D3D | -0.158 | 0.001784 |
| SERINC3 | -0.158 | 0.001815 |
| NDUFAF7 | -0.158 | 0.001854 |
| NCR3LG1 | -0.158 | 0.001856 |
| NBPF4 | -0.158 | 0.00186 |
| IFT57 | -0.158 | 0.001862 |
| PRR36 | -0.158 | 0.001885 |
| ASB13 | -0.159 | 0.001646 |
| TH | -0.159 | 0.001688 |
| SUOX | -0.159 | 0.001715 |
| CDC34 | -0.159 | 0.001721 |
| TTC14 | -0.159 | 0.001739 |
| DTYMK | -0.159 | 0.001755 |
| PHF20L1 | -0.16 | 0.001558 |
| RSRP1 | -0.16 | 0.001571 |
| MITD1 | -0.16 | 0.001612 |
| ZFP69 | -0.16 | 0.001626 |
| C11orf84 | -0.16 | 0.001633 |
| PAK1 | -0.16 | 0.001637 |
| C11orf71 | -0.16 | 0.001638 |
| ODF3L2 | -0.161 | 0.001437 |
| TPT1 | -0.161 | 0.001491 |
| SLC52A3 | -0.161 | 0.001515 |
| EI24 | -0.162 | 0.00136 |
| U2AF2 | -0.162 | 0.001394 |
| DDX18 | -0.162 | 0.001426 |
| EME1 | -0.162 | 0.001429 |
| IL20RA | -0.163 | 0.001266 |
| SLC19A3 | -0.163 | 0.001267 |
| MSTO1 | -0.163 | 0.001267 |
| DOLPP1 | -0.163 | 0.001267 |
| SMPX | -0.163 | 0.001278 |
| ZNF620 | -0.163 | 0.001293 |
| EARS2 | -0.163 | 0.001337 |
| PHKA1 | -0.164 | 0.001199 |
| UBE2I | -0.164 | 0.00123 |
| PARD6B | -0.164 | 0.001237 |
| NUTM2F | -0.165 | 0.001129 |
| ALDH1B1 | -0.165 | 0.001136 |
| ZNF66 | -0.165 | 0.00114 |
| NBR1 | -0.165 | 0.001158 |
| PDZD3 | -0.166 | 0.001028 |
| ZNHIT3 | -0.166 | 0.001072 |
| MGAT5 | -0.167 | 0.000943 |
| PLA2G4B | -0.167 | 0.000961 |
| HGD | -0.167 | 0.000974 |
| CCDC192 | -0.168 | 0.000881 |
| CHMP2A | -0.168 | 0.000881 |
| HILPDA | -0.168 | 0.000885 |
| ERLIN2 | -0.168 | 0.00089 |
| RPRD1B | -0.168 | 0.000929 |
| CUL3 | -0.168 | 0.00093 |
| C11orf74 | -0.168 | 0.000937 |
| METTL21A | -0.169 | 0.000818 |
| IQCE | -0.169 | 0.000839 |
| WNT3 | -0.169 | 0.000849 |
| OCLN | -0.169 | 0.000871 |
| CABLES2 | -0.17 | 0.000775 |
| URB1 | -0.17 | 0.00079 |
| KLHL8 | -0.17 | 0.000794 |
| MECR | -0.17 | 0.000797 |
| ZNF680 | -0.17 | 0.000805 |
| USP39 | -0.171 | 0.00071 |
| NME1-NME2 | -0.171 | 0.000719 |
| RAB40B | -0.171 | 0.000731 |
| FIGNL1 | -0.171 | 0.000737 |
| RACK1 | -0.171 | 0.00075 |
| DUSP27 | -0.172 | 0.000658 |
| TMBIM4 | -0.172 | 0.000659 |
| GINS2 | -0.172 | 0.000664 |
| MAL2 | -0.172 | 0.000669 |
| MED24 | -0.172 | 0.000681 |
| SRSF2 | -0.172 | 0.000696 |
| AQP12B | -0.172 | 0.000703 |
| MFSD14C | -0.173 | 0.000614 |
| ZNF253 | -0.173 | 0.000627 |
| ZNF558 | -0.173 | 0.000636 |
| IFNLR1 | -0.173 | 0.000643 |
| TLE2 | -0.173 | 0.000647 |
| HOMEZ | -0.173 | 0.000652 |
| SASS6 | -0.173 | 0.000654 |
| ALG1L2 | -0.174 | 0.00059 |
| CDC123 | -0.175 | 0.000525 |
| RRBP1 | -0.175 | 0.000539 |
| C20orf96 | -0.175 | 0.000545 |
| CDCA3 | -0.175 | 0.000545 |
| PMS1 | -0.175 | 0.000547 |
| MVB12B | -0.175 | 0.000553 |
| SLC16A11 | -0.175 | 0.000554 |
| C6orf47 | -0.175 | 0.000558 |
| NSMCE1 | -0.176 | 0.000488 |
| C10orf95 | -0.176 | 0.000502 |
| POLE | -0.176 | 0.000503 |
| DPY19L4 | -0.176 | 0.000507 |
| MED14 | -0.176 | 0.00052 |
| GRB14 | -0.177 | 0.000457 |
| N6AMT1 | -0.178 | 0.000428 |
| ADRM1 | -0.178 | 0.000431 |
| AC137834.1 | -0.178 | 0.000435 |
| ANKRD49 | -0.178 | 0.000438 |
| PMPCB | -0.179 | 0.00039 |
| TRIM11 | -0.179 | 0.000393 |
| MRPL45 | -0.179 | 0.000404 |
| ACOT8 | -0.179 | 0.00041 |
| GSTK1 | -0.18 | 0.000362 |
| ANKRD13B | -0.18 | 0.000362 |
| WASHC1 | -0.18 | 0.000365 |
| MTRF1 | -0.18 | 0.000376 |
| NTMT1 | -0.18 | 0.000379 |
| SLC25A33 | -0.18 | 0.000384 |
| COX6B1 | -0.18 | 0.000386 |
| RANBP1 | -0.181 | 0.000333 |
| NAA16 | -0.181 | 0.000334 |
| MAP7D2 | -0.181 | 0.000336 |
| ANKHD1-EIF4EBP3 | -0.181 | 0.000338 |
| ST14 | -0.181 | 0.000348 |
| KCMF1 | -0.181 | 0.000349 |
| EXD3 | -0.181 | 0.000351 |
| NIP7 | -0.182 | 0.000311 |
| PDRG1 | -0.182 | 0.000312 |
| PSMG1 | -0.182 | 0.000314 |
| DPEP1 | -0.182 | 0.000315 |
| ASTE1 | -0.182 | 0.000328 |
| ZCCHC4 | -0.182 | 0.000329 |
| MRPL53 | -0.183 | 0.000286 |
| ULBP1 | -0.183 | 0.000289 |
| BOD1 | -0.183 | 0.000294 |
| N4BP2L2 | -0.183 | 0.000301 |
| RAD51D | -0.184 | 0.000266 |
| C17orf75 | -0.184 | 0.000267 |
| EDAR | -0.184 | 0.000277 |
| FAM221B | -0.184 | 0.000277 |
| CDC42BPG | -0.184 | 0.000278 |
| EDF1 | -0.185 | 0.000249 |
| LRRC69 | -0.185 | 0.000253 |
| IPO5 | -0.185 | 0.000253 |
| KIN | -0.185 | 0.000254 |
| ENTPD6 | -0.185 | 0.000258 |
| TLK2 | -0.186 | 0.000226 |
| ZNF107 | -0.186 | 0.000227 |
| ZHX1-C8orf76 | -0.186 | 0.00023 |
| ZNF280B | -0.186 | 0.000238 |
| KIAA2012 | -0.187 | 0.00021 |
| SLC6A4 | -0.187 | 0.000222 |
| ACBD4 | -0.188 | 0.000195 |
| NOMO1 | -0.188 | 0.000202 |
| DPY19L3 | -0.188 | 0.000205 |
| LIPM | -0.188 | 0.000205 |
| COMMD7 | -0.188 | 0.000206 |
| RDH13 | -0.188 | 0.000206 |
| C3orf52 | -0.188 | 0.000207 |
| ASCL5 | -0.189 | 0.000184 |
| C14orf79 | -0.189 | 0.000185 |
| DDX52 | -0.189 | 0.000185 |
| CCDC167 | -0.189 | 0.00019 |
| PCBP2 | -0.19 | 0.000165 |
| MAGEB17 | -0.19 | 0.000165 |
| UTP23 | -0.19 | 0.000166 |
| SPSB2 | -0.19 | 0.000167 |
| C8orf33 | -0.191 | 0.000156 |
| TMEM199 | -0.191 | 0.000159 |
| CDADC1 | -0.191 | 0.000159 |
| ZSWIM3 | -0.191 | 0.000162 |
| FAM155B | -0.192 | 0.000139 |
| MEIG1 | -0.192 | 0.000148 |
| FAM217B | -0.192 | 0.00015 |
| OSBPL7 | -0.193 | 0.000128 |
| ZNF77 | -0.193 | 0.000131 |
| NUP214 | -0.193 | 0.000138 |
| PTCD3 | -0.193 | 0.000138 |
| TEX261 | -0.194 | 0.000118 |
| FAIM2 | -0.194 | 0.000118 |
| NAA20 | -0.194 | 0.000118 |
| DCUN1D2 | -0.194 | 0.000119 |
| MTA3 | -0.194 | 0.000119 |
| TMEM94 | -0.194 | 0.000121 |
| C20orf144 | -0.194 | 0.000124 |
| WDR53 | -0.194 | 0.000125 |
| AGAP5 | -0.196 | 1.00E-04 |
| GOSR1 | -0.196 | 0.0001 |
| CPSF4L | -0.196 | 0.000101 |
| RPLP2 | -0.196 | 0.000101 |
| ZNF572 | -0.196 | 0.000102 |
| UTP6 | -0.196 | 0.000103 |
| CPSF3 | -0.196 | 0.000105 |
| NUF2 | -0.196 | 0.000105 |
| GCC1 | -0.196 | 0.000107 |
| CCDC121 | -0.196 | 0.000107 |
| CHN2 | -0.196 | 0.000108 |
| RBM10 | -0.196 | 0.000108 |
| EHHADH | -0.196 | 0.000108 |
| SLC25A36 | -0.197 | 9.25E-05 |
| IRF6 | -0.197 | 9.34E-05 |
| GAR1 | -0.197 | 9.36E-05 |
| OXGR1 | -0.197 | 9.55E-05 |
| FAM184B | -0.197 | 9.83E-05 |
| MTBP | -0.198 | 8.90E-05 |
| PLEKHA8 | -0.198 | 8.93E-05 |
| SNRNP35 | -0.199 | 8.21E-05 |
| COPRS | -0.199 | 8.27E-05 |
| ATF4 | -0.199 | 8.40E-05 |
| DNAJC5 | -0.2 | 7.18E-05 |
| PROM2 | -0.2 | 7.34E-05 |
| GGT7 | -0.2 | 7.35E-05 |
| ZNF563 | -0.2 | 7.56E-05 |
| PUM2 | -0.2 | 7.59E-05 |
| PABPC4 | -0.2 | 7.68E-05 |
| PATJ | -0.201 | 6.59E-05 |
| CDK11A | -0.201 | 6.80E-05 |
| PREP | -0.201 | 6.92E-05 |
| NEURL2 | -0.202 | 6.05E-05 |
| MPZL3 | -0.202 | 6.19E-05 |
| TRIM68 | -0.202 | 6.28E-05 |
| B3GNTL1 | -0.202 | 6.53E-05 |
| LRRFIP2 | -0.203 | 5.64E-05 |
| THUMPD2 | -0.204 | 5.24E-05 |
| MSANTD2 | -0.204 | 5.27E-05 |
| PHKG2 | -0.204 | 5.30E-05 |
| POLR1C | -0.204 | 5.33E-05 |
| PSPH | -0.204 | 5.38E-05 |
| FITM2 | -0.204 | 5.43E-05 |
| CIAO1 | -0.205 | 4.73E-05 |
| PAPD5 | -0.205 | 4.81E-05 |
| ZNF736 | -0.205 | 4.92E-05 |
| ZBTB26 | -0.205 | 4.97E-05 |
| SRSF1 | -0.206 | 4.33E-05 |
| MRPL17 | -0.206 | 4.50E-05 |
| HEATR3 | -0.206 | 4.51E-05 |
| PRR13 | -0.206 | 4.59E-05 |
| ZNF250 | -0.207 | 4.02E-05 |
| POP1 | -0.207 | 4.11E-05 |
| KIF15 | -0.207 | 4.13E-05 |
| SUGT1 | -0.208 | 3.78E-05 |
| DDX42 | -0.208 | 3.81E-05 |
| SFXN5 | -0.208 | 3.82E-05 |
| TMEM5 | -0.209 | 3.28E-05 |
| TSN | -0.209 | 3.40E-05 |
| ZHX3 | -0.209 | 3.45E-05 |
| RPL29 | -0.211 | 2.75E-05 |
| PNLIPRP1 | -0.211 | 2.77E-05 |
| DDX19B | -0.211 | 2.84E-05 |
| AK4 | -0.212 | 2.53E-05 |
| GHDC | -0.212 | 2.59E-05 |
| TADA2A | -0.213 | 2.32E-05 |
| CSPP1 | -0.213 | 2.39E-05 |
| E2F5 | -0.214 | 2.09E-05 |
| CADPS | -0.215 | 1.94E-05 |
| DDX1 | -0.215 | 2.05E-05 |
| KIF3B | -0.215 | 2.08E-05 |
| MVK | -0.215 | 2.08E-05 |
| FAM168B | -0.216 | 1.83E-05 |
| BTF3 | -0.217 | 1.73E-05 |
| KIAA1257 | -0.217 | 1.73E-05 |
| C2orf70 | -0.218 | 1.48E-05 |
| TBC1D4 | -0.218 | 1.49E-05 |
| RPS10 | -0.218 | 1.50E-05 |
| SHD | -0.218 | 1.52E-05 |
| MTERF3 | -0.218 | 1.53E-05 |
| NMNAT3 | -0.218 | 1.57E-05 |
| HENMT1 | -0.219 | 1.32E-05 |
| NDUFAF6 | -0.219 | 1.34E-05 |
| CD46 | -0.219 | 1.38E-05 |
| CSTF1 | -0.219 | 1.39E-05 |
| UXT | -0.219 | 1.40E-05 |
| HNRNPUL2 | -0.219 | 1.41E-05 |
| JMJD7-PLA2G4B | -0.22 | 1.21E-05 |
| TTPA | -0.22 | 1.21E-05 |
| MRPS18B | -0.22 | 1.24E-05 |
| HOXA7 | -0.22 | 1.26E-05 |
| ALG9 | -0.22 | 1.31E-05 |
| LMOD3 | -0.221 | 1.09E-05 |
| ANKRD46 | -0.221 | 1.13E-05 |
| OSGEPL1 | -0.221 | 1.18E-05 |
| ZNF446 | -0.221 | 1.18E-05 |
| UQCRH | -0.222 | 9.95E-06 |
| CHEK2 | -0.222 | 9.99E-06 |
| CKAP5 | -0.222 | 1.07E-05 |
| TBC1D3L | -0.222 | 1.09E-05 |
| HCN3 | -0.223 | 9.39E-06 |
| IL1RL2 | -0.223 | 9.70E-06 |
| HS1BP3 | -0.223 | 9.72E-06 |
| PFDN1 | -0.223 | 9.76E-06 |
| LARP1 | -0.223 | 9.92E-06 |
| NDRG3 | -0.224 | 8.53E-06 |
| WDR43 | -0.224 | 8.56E-06 |
| KRTAP4-1 | -0.224 | 8.61E-06 |
| SCML2 | -0.224 | 8.66E-06 |
| RNF219 | -0.225 | 7.48E-06 |
| NCOA5 | -0.225 | 7.58E-06 |
| TMA16 | -0.225 | 7.63E-06 |
| CEACAM19 | -0.225 | 7.75E-06 |
| TMEM68 | -0.225 | 7.92E-06 |
| ZNF7 | -0.225 | 7.96E-06 |
| RPL24 | -0.225 | 8.05E-06 |
| PCID2 | -0.225 | 8.09E-06 |
| KAT14 | -0.225 | 8.11E-06 |
| HIBADH | -0.225 | 8.11E-06 |
| TMPRSS13 | -0.226 | 6.84E-06 |
| RAD18 | -0.226 | 7.17E-06 |
| ZNF273 | -0.226 | 7.46E-06 |
| MMAB | -0.226 | 7.47E-06 |
| CELSR3 | -0.227 | 6.57E-06 |
| FBXL8 | -0.228 | 5.72E-06 |
| SRC | -0.23 | 4.65E-06 |
| MINOS1 | -0.23 | 4.75E-06 |
| CRB2 | -0.23 | 4.78E-06 |
| MBTD1 | -0.23 | 4.87E-06 |
| MISP3 | -0.23 | 4.90E-06 |
| CHDH | -0.231 | 4.18E-06 |
| GTPBP4 | -0.231 | 4.20E-06 |
| RBM19 | -0.231 | 4.21E-06 |
| LNX1 | -0.231 | 4.31E-06 |
| CKMT1A | -0.231 | 4.37E-06 |
| CCDC120 | -0.231 | 4.41E-06 |
| LRRC6 | -0.231 | 4.44E-06 |
| SPATA25 | -0.231 | 4.50E-06 |
| PKP3 | -0.232 | 3.80E-06 |
| MATR3 | -0.232 | 3.83E-06 |
| RBL1 | -0.232 | 3.86E-06 |
| SMIM4 | -0.233 | 3.63E-06 |
| PRSS8 | -0.234 | 3.28E-06 |
| AKNAD1 | -0.234 | 3.33E-06 |
| E2F3 | -0.234 | 3.34E-06 |
| TG | -0.235 | 2.89E-06 |
| CPA6 | -0.235 | 2.91E-06 |
| EPB41L5 | -0.235 | 2.98E-06 |
| MROH7 | -0.235 | 3.07E-06 |
| RAD54B | -0.236 | 2.55E-06 |
| GPR160 | -0.236 | 2.66E-06 |
| LBR | -0.236 | 2.67E-06 |
| KAZALD1 | -0.236 | 2.77E-06 |
| BCAN | -0.237 | 2.39E-06 |
| DNMT3B | -0.237 | 2.46E-06 |
| CYP2J2 | -0.237 | 2.50E-06 |
| RAB25 | -0.238 | 2.11E-06 |
| INPP5J | -0.238 | 2.11E-06 |
| ANKMY1 | -0.238 | 2.20E-06 |
| PTCD1 | -0.239 | 2.05E-06 |
| DNAAF5 | -0.239 | 2.08E-06 |
| GALE | -0.24 | 1.86E-06 |
| MFSD9 | -0.241 | 1.56E-06 |
| STAMBP | -0.241 | 1.61E-06 |
| ZC3H15 | -0.242 | 1.39E-06 |
| IGFL4 | -0.242 | 1.42E-06 |
| HIGD2A | -0.242 | 1.44E-06 |
| ZNF57 | -0.243 | 1.25E-06 |
| TMED4 | -0.244 | 1.14E-06 |
| TTC32 | -0.244 | 1.19E-06 |
| RPN2 | -0.244 | 1.19E-06 |
| CTNNBL1 | -0.244 | 1.23E-06 |
| MRM2 | -0.245 | 1.08E-06 |
| CBLC | -0.245 | 1.08E-06 |
| SIAH1 | -0.246 | 9.98E-07 |
| HPX | -0.247 | 8.40E-07 |
| RPL3 | -0.247 | 8.40E-07 |
| TSPAN6 | -0.247 | 8.41E-07 |
| ZSWIM1 | -0.247 | 8.77E-07 |
| SMIM15 | -0.247 | 8.83E-07 |
| ZNF711 | -0.248 | 7.79E-07 |
| SERGEF | -0.249 | 6.93E-07 |
| COX7C | -0.25 | 6.45E-07 |
| PHOSPHO2 | -0.25 | 6.54E-07 |
| ST13 | -0.251 | 5.98E-07 |
| MROH7-TTC4 | -0.253 | 4.49E-07 |
| PTGES3L-AARSD1 | -0.253 | 4.65E-07 |
| BFSP1 | -0.254 | 4.15E-07 |
| UBAP2 | -0.254 | 4.18E-07 |
| ALG1L | -0.254 | 4.30E-07 |
| NCK2 | -0.254 | 4.33E-07 |
| SRSF6 | -0.255 | 3.66E-07 |
| PDP2 | -0.255 | 3.89E-07 |
| NELFCD | -0.256 | 3.16E-07 |
| NDUFB2 | -0.256 | 3.20E-07 |
| MZT2B | -0.256 | 3.22E-07 |
| ILDR1 | -0.256 | 3.45E-07 |
| TAS2R38 | -0.257 | 2.82E-07 |
| ACBD6 | -0.257 | 2.83E-07 |
| EIF3F | -0.257 | 2.85E-07 |
| NCOA6 | -0.257 | 3.04E-07 |
| DHX35 | -0.258 | 2.57E-07 |
| MKS1 | -0.258 | 2.75E-07 |
| TBCA | -0.259 | 2.29E-07 |
| PALD1 | -0.259 | 2.29E-07 |
| DAGLA | -0.259 | 2.34E-07 |
| STAU1 | -0.259 | 2.46E-07 |
| TEX45 | -0.26 | 2.10E-07 |
| MRPL3 | -0.261 | 1.83E-07 |
| ORC6 | -0.261 | 1.89E-07 |
| ERICH2 | -0.261 | 1.93E-07 |
| CCT3 | -0.261 | 1.96E-07 |
| ARSD | -0.261 | 1.98E-07 |
| PIWIL4 | -0.263 | 1.49E-07 |
| ZBTB10 | -0.263 | 1.52E-07 |
| ACY1 | -0.263 | 1.52E-07 |
| VAPB | -0.263 | 1.58E-07 |
| CHCHD6 | -0.263 | 1.59E-07 |
| GMEB2 | -0.264 | 1.44E-07 |
| NOSTRIN | -0.265 | 1.23E-07 |
| HES1 | -0.265 | 1.28E-07 |
| ABHD14B | -0.266 | 1.04E-07 |
| TKT | -0.266 | 1.09E-07 |
| SARS2 | -0.267 | 9.27E-08 |
| CHMP4B | -0.267 | 9.32E-08 |
| GIT1 | -0.267 | 9.48E-08 |
| BCAP31 | -0.267 | 1.01E-07 |
| FLVCR1 | -0.268 | 8.26E-08 |
| RTFDC1 | -0.268 | 8.39E-08 |
| YTHDF1 | -0.268 | 9.01E-08 |
| METTL1 | -0.269 | 7.42E-08 |
| AIFM3 | -0.269 | 7.63E-08 |
| PEBP1 | -0.269 | 7.82E-08 |
| EIF6 | -0.269 | 7.86E-08 |
| ARHGEF39 | -0.269 | 7.97E-08 |
| SERINC5 | -0.269 | 8.14E-08 |
| ZNF782 | -0.27 | 6.51E-08 |
| ARHGAP39 | -0.27 | 6.57E-08 |
| KCNJ14 | -0.27 | 6.65E-08 |
| GID8 | -0.27 | 6.93E-08 |
| MRGBP | -0.27 | 7.18E-08 |
| SS18L1 | -0.27 | 7.25E-08 |
| AGMAT | -0.271 | 6.17E-08 |
| SLC37A4 | -0.272 | 5.19E-08 |
| UQCC1 | -0.272 | 5.39E-08 |
| USP10 | -0.272 | 5.68E-08 |
| DPM1 | -0.274 | 4.06E-08 |
| RPL12 | -0.275 | 3.88E-08 |
| CYP39A1 | -0.275 | 3.91E-08 |
| PRPF19 | -0.276 | 3.42E-08 |
| KCNN4 | -0.276 | 3.45E-08 |
| RAE1 | -0.277 | 2.98E-08 |
| CALML4 | -0.277 | 3.06E-08 |
| HNRNPA2B1 | -0.278 | 2.55E-08 |
| MT-CO3 | -0.278 | 2.67E-08 |
| ATP5G2 | -0.278 | 2.79E-08 |
| AAR2 | -0.28 | 2.03E-08 |
| CLRN3 | -0.28 | 2.07E-08 |
| AUTS2 | -0.28 | 2.10E-08 |
| MRPL24 | -0.281 | 1.84E-08 |
| EEF1D | -0.282 | 1.58E-08 |
| ACOX2 | -0.283 | 1.41E-08 |
| C12orf45 | -0.283 | 1.45E-08 |
| ASIC1 | -0.283 | 1.46E-08 |
| RAD23A | -0.283 | 1.51E-08 |
| DNAJA3 | -0.284 | 1.26E-08 |
| POFUT1 | -0.286 | 1.07E-08 |
| SHH | -0.287 | 9.11E-09 |
| POLG2 | -0.288 | 7.75E-09 |
| HNF4A | -0.288 | 8.19E-09 |
| XYLB | -0.289 | 7.12E-09 |
| NSUN5 | -0.29 | 5.86E-09 |
| PXMP4 | -0.29 | 6.35E-09 |
| RNF114 | -0.29 | 6.38E-09 |
| MYB | -0.291 | 5.21E-09 |
| TM9SF4 | -0.293 | 4.13E-09 |
| ZNF19 | -0.293 | 4.44E-09 |
| RPL32 | -0.294 | 3.62E-09 |
| C8orf44 | -0.294 | 3.70E-09 |
| SNRPA | -0.295 | 3.17E-09 |
| RPL36A-HNRNPH2 | -0.297 | 2.59E-09 |
| DDC | -0.299 | 1.85E-09 |
| SLC29A2 | -0.302 | 1.27E-09 |
| LSG1 | -0.303 | 1.16E-09 |
| AVL9 | -0.305 | 9.44E-10 |
| ATP1A1 | -0.306 | 8.09E-10 |
| C11orf49 | -0.306 | 8.13E-10 |
| PA2G4 | -0.308 | 5.80E-10 |
| KIAA1958 | -0.308 | 5.82E-10 |
| NOB1 | -0.308 | 6.03E-10 |
| TMEM97 | -0.31 | 4.28E-10 |
| TCTE3 | -0.311 | 3.81E-10 |
| PLA2G4F | -0.313 | 2.91E-10 |
| LSM14B | -0.314 | 2.52E-10 |
| TMC4 | -0.314 | 2.75E-10 |
| CMTM8 | -0.315 | 2.45E-10 |
| TAF4 | -0.317 | 1.65E-10 |
| ZNF444 | -0.319 | 1.28E-10 |
| ADNP | -0.32 | 1.20E-10 |
| EEF1G | -0.321 | 1.03E-10 |
| BRD7 | -0.322 | 9.11E-11 |
| CC2D1A | -0.323 | 7.41E-11 |
| MEST | -0.327 | 4.22E-11 |
| STX16 | -0.327 | 4.39E-11 |
| IPO4 | -0.328 | 3.70E-11 |
| CEP95 | -0.328 | 3.70E-11 |
| HOXB9 | -0.328 | 3.81E-11 |
| PDAP1 | -0.329 | 3.09E-11 |
| SNRPE | -0.33 | 2.63E-11 |
| RBM39 | -0.332 | 1.96E-11 |
| GNL3 | -0.334 | 1.49E-11 |
| TPX2 | -0.334 | 1.62E-11 |
| PIGU | -0.336 | 1.15E-11 |
| CRCP | -0.336 | 1.22E-11 |
| PAICS | -0.337 | 9.46E-12 |
| DUS4L | -0.337 | 1.03E-11 |
| ADCK1 | -0.34 | 6.30E-12 |
| TMEM184A | -0.341 | 5.34E-12 |
| STX19 | -0.343 | 3.93E-12 |
| SLC5A6 | -0.345 | 2.80E-12 |
| FAM71E1 | -0.351 | 1.21E-12 |
| EPS8L3 | -0.352 | 9.26E-13 |
| TMEM105 | -0.354 | 7.75E-13 |
| TFCP2L1 | -0.356 | 5.55E-13 |
| RPL6 | -0.357 | 4.38E-13 |
| CDK5RAP1 | -0.359 | 3.38E-13 |
| MTERF2 | -0.359 | 3.48E-13 |
| RPL11 | -0.362 | 1.98E-13 |
| DDX27 | -0.365 | 1.17E-13 |
| USH1C | -0.367 | 8.75E-14 |
| NACA | -0.37 | 5.61E-14 |
| EPCAM | -0.372 | 3.87E-14 |
| ARHGEF5 | -0.374 | 2.74E-14 |
| ZNF692 | -0.375 | 2.14E-14 |
| SMYD5 | -0.383 | 5.77E-15 |
| COX20 | -0.386 | 3.28E-15 |
| WNK4 | -0.392 | 1.12E-15 |
| CCDC58 | -0.394 | 7.56E-16 |
| BCL11A | -0.396 | 5.28E-16 |
| RPS24 | -0.398 | 4.19E-16 |
| VDAC1 | -0.404 | 1.21E-16 |
| KAT2A | -0.408 | 6.45E-17 |
| ETV4 | -0.421 | 4.82E-18 |
| RPL36A | -0.434 | 3.54E-19 |
