# Supplementary material for: KRAS mutations are negatively correlated with immunity in colon cancer
Source: Aging (Albany NY). 2020 Nov 26;13(1):750–68. doi: 10.18632/aging.202182 (PMC7834984; doi:10.18632/aging.202182)
Supplement: Supplementary Table 8 [file aging-13-202182-s007.pdf]

SUPPLEMENTARY TABLE

Supplementary Table 8. The annotations of differentially expressed genes with strong correlation with immune score.

Gene names	Entry	Protein names	Length
STX11	O75558	Syntaxin-11	287
DOK2	O60496	Docking protein 2 (Downstream of tyrosine kinase 2)	412
TNFAIP8L2	Q6P589	Tumor necrosis factor alpha-induced protein 8-like protein 2	184
LCP2	Q13094	Lymphocyte cytosolic protein 2	533
LILRB1	Q8NHL6	Leukocyte immunoglobulin-like receptor subfamily B member 1	650
ABI3	Q9P2A4	ABI gene family member 3	366
LRRC25	Q8N386	Leucine-rich repeat-containing protein 25	305
SASH3	O75995	SAM and SH3 domain-containing protein 3	380
CD300A	Q9UGN4	CMRF35-like molecule 8	299
LAIR1	Q6GTX8	Leukocyte-associated immunoglobulin-like receptor 1	287
MS4A6A	Q9H2W1	Membrane-spanning 4-domains subfamily A member 6A	248
SCIMP	Q6UWF3	SLP adapter and CSK-interacting membrane protein	145
APBB1IP	Q7Z5R6	Amyloid beta A4 precursor protein-binding family B member 1-interacting protein	666
HCLS1	P14317	Hematopoietic lineage cell-specific protein	486
C3AR1	Q16581	C3a anaphylatoxin chemotactic receptor	482
IL2RA	P01589	Interleukin-2 receptor subunit alpha	272
HAVCR2	Q8TDQ0	Hepatitis A virus cellular receptor 2	301
CD300LF	Q8TDQ1	CMRF35-like molecule 1 (Immune receptor expressed on myeloid cells 1)	290
LST1	O00453	Leukocyte-specific transcript 1 protein	97
LST1	Q9Y6L6	Solute carrier organic anion transporter family member 1B1	691
SLAMF8	Q9P0V8	SLAM family member 8 (B-lymphocyte activator macrophage expressed)	285
C10orf128	Q5T292	Transmembrane protein 273	105
CD86	P42081	T-lymphocyte activation antigen CD86	329
IL10RA	Q13651	Interleukin-10 receptor subunit alpha	578
CYTH4	Q9UIA0	Cytohesin-4	394
